# Earthquake hazard and risk analysis for natural and induced seismicity: towards objective assessments in the face of uncertainty

**DOI:** 10.1007/s10518-022-01357-4

**Published:** 2022-04-22

**Authors:** Julian J. Bommer

**Affiliations:** Civil and Environmental Engineering Department, Imperial College London, South Kensington Campus, London, SW7 2AZ UK

**Keywords:** Earthquake hazards, Seismic hazard analysis, Seismic risk, Epistemic uncertainty, Induced seismicity, Seismic risk mitigation

## Abstract

The fundamental objective of earthquake engineering is to protect lives and livelihoods through the reduction of seismic risk. Directly or indirectly, this generally requires quantification of the risk, for which quantification of the seismic hazard is required as a basic input. Over the last several decades, the practice of seismic hazard analysis has evolved enormously, firstly with the introduction of a rational framework for handling the apparent randomness in earthquake processes, which also enabled risk assessments to consider both the severity and likelihood of earthquake effects. The next major evolutionary step was the identification of epistemic uncertainties related to incomplete knowledge, and the formulation of frameworks for both their quantification and their incorporation into hazard assessments. Despite these advances in the practice of seismic hazard analysis, it is not uncommon for the acceptance of seismic hazard estimates to be hindered by invalid comparisons, resistance to new information that challenges prevailing views, and attachment to previous estimates of the hazard. The challenge of achieving impartial acceptance of seismic hazard and risk estimates becomes even more acute in the case of earthquakes attributed to human activities. A more rational evaluation of seismic hazard and risk due to induced earthquakes may be facilitated by adopting, with appropriate adaptations, the advances in risk quantification and risk mitigation developed for natural seismicity. While such practices may provide an impartial starting point for decision making regarding risk mitigation measures, the most promising avenue to achieve broad societal acceptance of the risks associated with induced earthquakes is through effective regulation, which needs to be transparent, independent, and informed by risk considerations based on both sound seismological science and reliable earthquake engineering.

## Introduction

The study of earthquakes serves many noble purposes, starting with humankind’s need to understand the planet on which we live and the causes of these calamitous events that challenge the very idea of residing on *terra firma*. Throughout history, peoples living in seismically active regions have formulated explanations for earthquakes, attributing their occurrence to the actions to disgruntled deities, mythical creatures or, later on, the Aristotelian view that earthquakes are caused by winds trapped and heated within a cavernous Earth (which is echoed in Shakespeare’s *Henry IV, Part 1*). While it is easy for us to look on these worldviews as quaint or pitifully ignorant, our modern understanding of earthquakes and their origins is very recent (when my own father studied geology as part of his civil engineering education, the framework of plate tectonics for understanding geological events had yet to be formulated and published). The discipline of seismology has advanced enormously during the last century or so, and our understanding of earthquakes continues to grow. The study of seismicity was instrumental in understanding plate tectonics and the analysis of seismic waves recorded on sensitive instruments all over the world has revealed, like global X-rays, the interior structure of our planet. As well as such advances in science, the development of seismology has also brought very tangible societal benefits, one of the most laudable being to distinguish the signals generated by underground tests of nuclear weapons from those generated by earthquakes, which made a comprehensive test ban treaty possible (Bolt 1976).

The most compelling reason to study earthquakes, however, must now be to mitigate their devastating impacts on people and on societies. A great deal of effort has been invested in developing predictions of earthquakes, since with sufficient prior warning, evacuations could prevent loss of life and injury. There have been some remarkable successes, most notably the prediction of the February 1975 Haicheng earthquake in China (Adams 1976); however, the following year, the Tangshan earthquake on 28 July occurred without warning and took the lives of several hundreds of thousands of people. More recently, there has been a focus on earthquake early warning systems (e.g., Gasparini et al. 2007), which can provide between seconds and tens of seconds of advance warning that can allow life-saving actions to be taken. However, whether strong ground shaking is predicted a few seconds or even a few days ahead of time, the built environment will still be exposed to the effects of the earthquake. Consequently, the most effective and reliable approach to protecting individuals and societies from the impact of earthquakes is through seismically resistant design and construction.

**Fig. 1:**
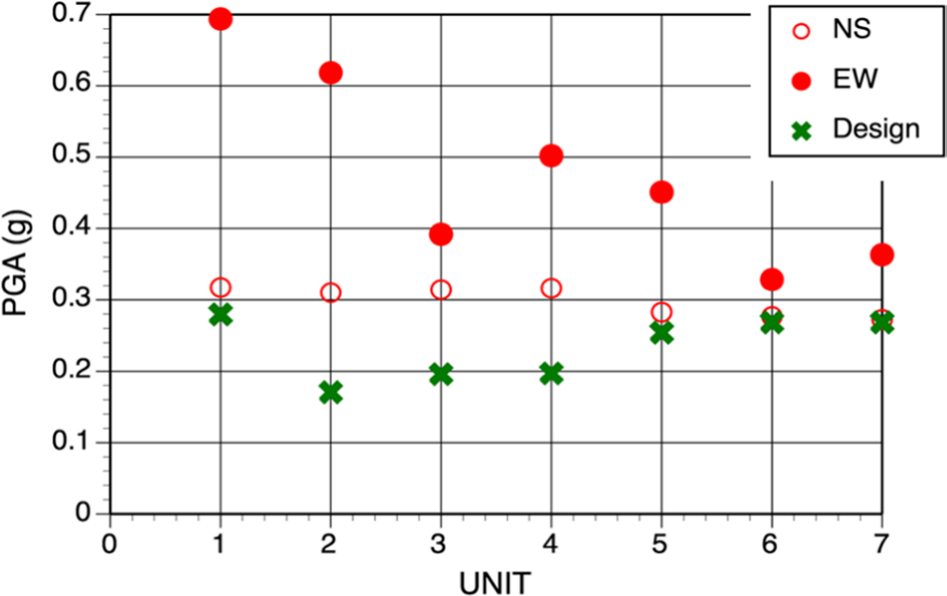
Recorded values of horizontal peak ground acceleration (PGA) at each unit of the Kashiwazaki-Kawira NPP during the 16 July 2007 Niigata Chūetsu earthquake (courtesy of Dr Norm Abrahamson)

To be cost effective in the face of limited resources, earthquake-resistant design first requires quantification of the expected levels of loading due to possible future earthquakes. Although not always made explicit, to demonstrate that the design is effective in providing the target levels of safety requires the analysis of the consequences of potential earthquake scenarios, for which the expected shaking levels are also required. The practice of assessing earthquake actions has progressed enormously over the last half century, especially in terms of identifying and quantifying uncertainties related to the location, magnitude, and frequency of future earthquakes, and to the levels of ground shaking that these will generate at a given location. The benefit of incorporating these uncertainties into the estimates of ground shaking levels is that the uncertainty can be taken into account in the definition of the design accelerations. This is not to say that seismic safety relies entirely on estimating the ‘correct’ level of seismic loading: additional margin is included in structural design, as has been clearly demonstrated by the safe performance of three different nuclear power plants in recent years. In July 2007, the magnitude 6.6 Niigata Chūetsu earthquake in western Japan occurred very close to the Kashiwazaki-Kawira nuclear power plant (NPP). At all seven reactor units, recorded accelerations exceeded the design motions (Fig. 1) without leading to any loss of radioactive containment. The magnitude 9.0 Tōhoku earthquake in March 2011 on the opposite coast of Japan generated motions at the Fukushima Daiichi NPP that also exceeded the design accelerations (Grant et al. 2017); the ensuing tsunami led to a severe nuclear accident at the plant, but the plant withstood the ground shaking without distress. A few months later, motions recorded at the North Anna NPP due to the **M** 5.8 Mineral, Virginia, USA earthquake also exceeded design acceleration levels without causing damage (Graizer et al. 2013).

Seismic safety in critical structures such as NPPs depends therefore on both the margins of resistance above the nominal design accelerations and the degree to which the estimates of the site demand, to which the design motions are referenced, reflect the uncertainty in their assessment. Therefore, for a nuclear regulator, capture of uncertainty in the assessment of seismic shaking levels provides assurance regarding the provision of adequate safety. However, the inclusion of large degrees of uncertainty can be viewed quite differently by other groups. For example, since inclusion of uncertainty generally leads to higher estimates of the accelerations (in theory broader uncertainty bands could lead to lower accelerations, but in practice it tends to push estimates in the opposite direction), owners and operators of these facilities may be averse to the inclusion of large intervals of uncertainty, especially if these are viewed as unnecessarily wide. For the public, capture of broad ranges of uncertainty in the estimates of earthquake hazard could be interpreted either way: on the one hand, it could be viewed positively as nuclear safety being enhanced through consideration of events that are stronger than what has been previously observed, whereas on the other hand, it could be seen as evidence that the science is too unsure to inform rational decision making and, in the face of such unknowns, safety cannot be guaranteed. The challenge therefore is two-fold: to develop impartial quantification of earthquake hazard and risk, and for these estimates to then be objectively accepted as the baseline for decision making regarding the management of the risk. This article discusses important advances in the estimation of earthquake hazard, and also explores, with concrete examples from practice, why impartial hazard estimates are sometimes met with stern— or even belligerent—resistance.

**Fig. 2:**
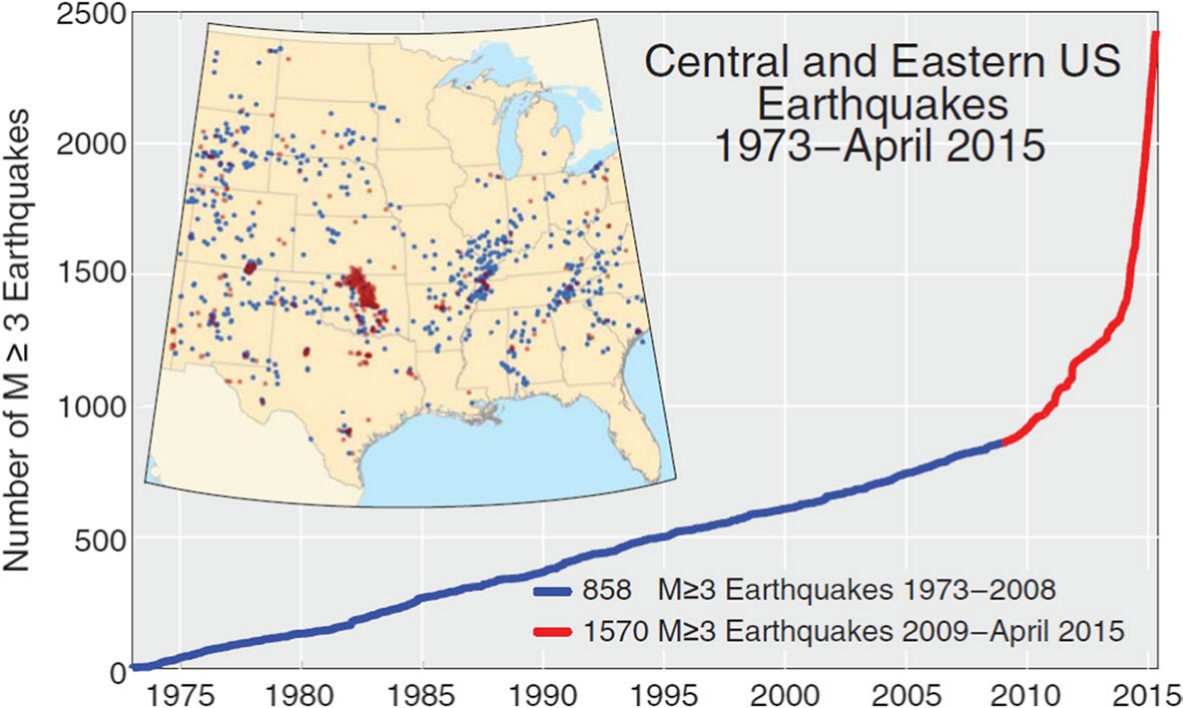
Increase in seismicity in the Central and Eastern United States from 2009 to 2015 related to hydrocarbon production (Rubinstein and Babaie Mahani 2015)

In recent years, earthquakes related to human activities—and generally referred to as induced seismicity—have attracted a great deal of scientific and societal attention. This has been driven primarily by more frequent occurrence of earthquakes of anthropogenic origin; a prime example being the remarkable increase in seismicity in the states of Oklahoma, Kentucky, and Texas, which has been related to hydrocarbon production (Fig. 2). However, the profile of induced seismicity in public debate, the media, and government policy has also been heightened by the controversy related to some of the industrial activities that have been shown to cause induced earthquakes, particularly hydraulic fracturing or fracking.

The seismic hazard (shaking levels) and risk (damage) due to induced seismicity can be estimated using the procedures that have been developed for natural seismicity, with appropriate adjustments for the distinct characteristics of induced earthquakes. The frameworks that have been developed for estimating seismic hazard due to natural earthquakes should be taken advantage of in the field of induced seismicity given that the controversy surrounding these cases often makes it imperative to correctly identify the degrees of uncertainty. Equally important, however, is to bring into the quantification of induced seismic hazard an engineering perspective that relates the hazard to risk. I make the case in this article that to date the assessment of induced seismic hazard has often not quantified uncertainty well and, perhaps more importantly, has failed to relate the hazard to a rational quantification of risk. These shortcomings are particularly important because the challenges of the hazard estimates being accepted by different groups are often particularly acute, much more so than is the case of natural seismicity. A key question that the article sets out to address is whether it is possible for robust estimates of seismic hazard associated with potential induced earthquakes to be adopted at face value. This leads to the question of whether the hazard estimates can be used as a starting point in discussions surrounding the rational management of the associated risk and its balance with the benefits of the industrial activity with the potential to cause seismic activity. This article discusses a number of case histories in which such objectivity was glaringly absent, and also explores options that might facilitate the impartial acceptance of estimates of induced seismic hazard.

The focus of this paper, as its title indicates, is to promote objectivity in the assessment of seismic hazard and risk for both natural and induced earthquakes. Assessment therefore refers to two different processes, reflecting the focus of this article on the balance of these two aspects noted above: (1) the estimation of possible or expected levels of earthquake shaking; and (2) the interpretation or evaluation of these estimates as a reliable basis for risk mitigation. Despite this deliberate ambiguity in the use of the word assessment, clear and consistent terminology is actually of great importance, for which reason the article starts with brief definitions of the key concepts embedded in the title: the meaning of hazard and risk (Sect. 1.1), and then the nature of uncertainty (Sect. 1.2). This introduction then concludes with a brief overview of the paper (Sect. 1.3).

### Seismic hazard and seismic risk

Seismic risk refers to undesirable consequences of earthquakes, which include death, injury, physical damage to buildings and infrastructure, interruption of business and social activities, and the direct and indirect costs associated with such outcomes. In a generic sense, risk can be defined as the possibility of such consequences occurring at a given location due to potential future earthquakes. In a more formal probabilistic framework, seismic risk is quantified by both the severity of a given metric of loss and the annual frequency or probability of that level of loss being exceeded.

Seismic hazard refers to the potentially damaging effects of earthquakes, the primary example being strong ground shaking (the full range of earthquake effects is discussed in Sect. 2). Again, in a generic sense, seismic hazard can be thought of as the possibility of strong shaking—measured, for example, by a specific level of peak ground acceleration (PGA)—occurring at a given location. In a probabilistic framework, the hazard is the probability or annual frequency of exceedance of different levels of the chosen measure of the vibratory ground motion.

Seismic hazard does not automatically create seismic risk: an earthquake in an entirely unpopulated region or in the middle of the ocean (remote from any submarine cables) will not constitute a risk: except, potentially, to any passing marine vessel (Ambraseys 1985). Risk only arises when there are buildings or infrastructure (such as transport networks, ports and harbours, energy generation and distribution systems, dams, pipelines, etc.) present at the locations affected by the shaking. The elements of the built environment that could be affected by earthquakes are referred to collectively as the exposure.

**Fig. 3:**
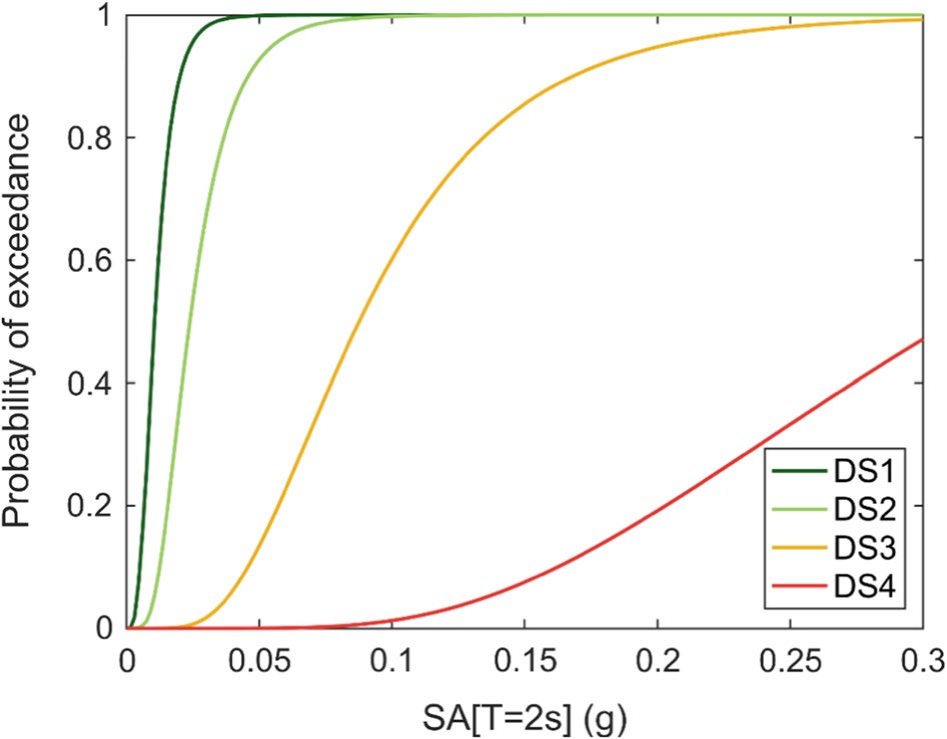
Fragility curves for a specific type of building, indicating the probability of exceeding different damage states as a function of spectral acceleration at a period of 2 s (Edwards et al. 2021)

For a given element of exposure, the seismic risk is controlled in the first instance by the degree of damage that could be inflicted by an earthquake. This depends on the strength of the possible ground shaking at the site (the hazard) and how much damage the structure is likely to suffer under different levels of ground shaking, which is referred to as the fragility. Damage is often generally defined by discrete damage states, such as those specified in the European Macroseismic Scale (Grünthal 1998): DS1 is negligible to slight (slight non-structural damage, no structural damage), DS2 is moderate (slight structural damage, moderate non-structural damage), DS3 is substantial to heavy (moderate structural damage, heavy non-structural damage), DS4 is very heavy (heavy structural damage, very heavy non-structural damage), and DS5 is extensive (very heavy structural damage or collapse). An example set of fragility functions for a given building type is shown in Fig. 3.

**Fig. 4:**
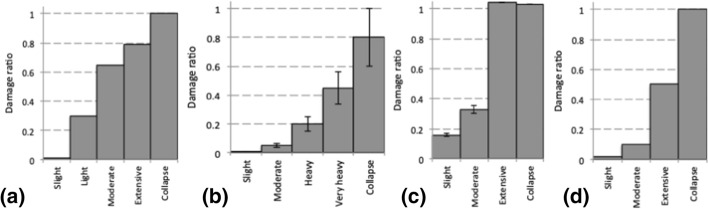
Examples of consequence functions that translate damage states to damage or cost ratios, from **a** Italy, **b** Greece, **c** Turkey and **d** California, (Silva et al. 2015)

Risk is generally quantified by metrics that more readily communicate the impact than the degree of structural and non-structural damage, such as the number of injured inhabitants or the direct costs of the damage. To translate the physical damage into other metrics requires a consequence function. Figure 4 shows examples of such functions that convert different damage states to costs, defined by damage ratios or cost ratios that are simply the cost of repairing the damage normalised by the cost of replacing the building. In some risk analyses, the fragility and consequence functions are merged so that risk metrics such as cost ratios or loss of life are predicted directly as a function of the ground shaking level; such functions are referred to as vulnerability curves. The choice to use fragility or vulnerability curves depends on the purpose of the risk study: to design structural strengthening schemes, insight is required regarding the expected physical damage, whereas for insurance purposes, the expected costs of earthquake damage may suffice.

**Fig. 5:**
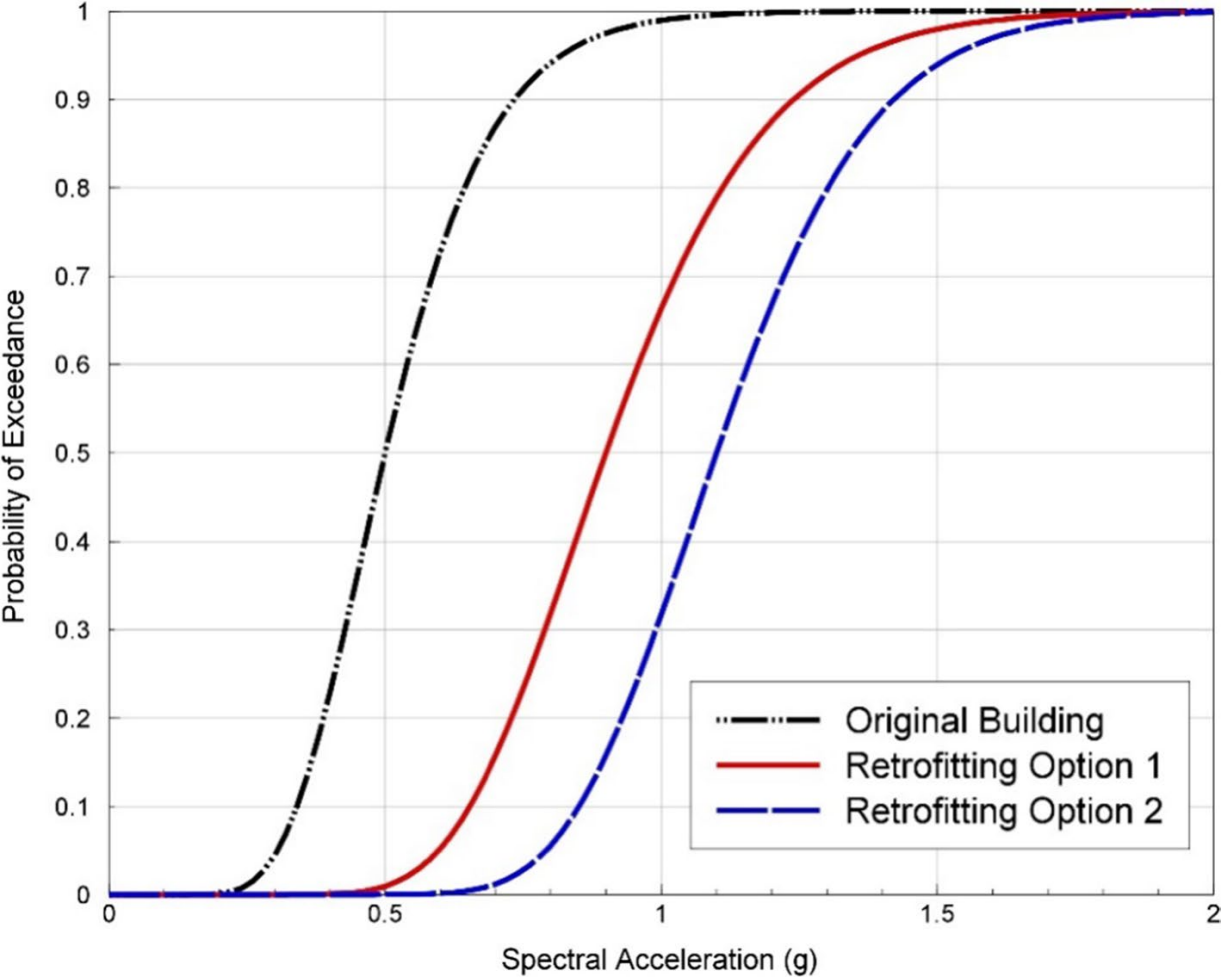
Illustration of the effect of seismic strengthening measures on fragility curves for a specific building type and damage state (Bommer et al. 2015a)

**Fig. 6:**
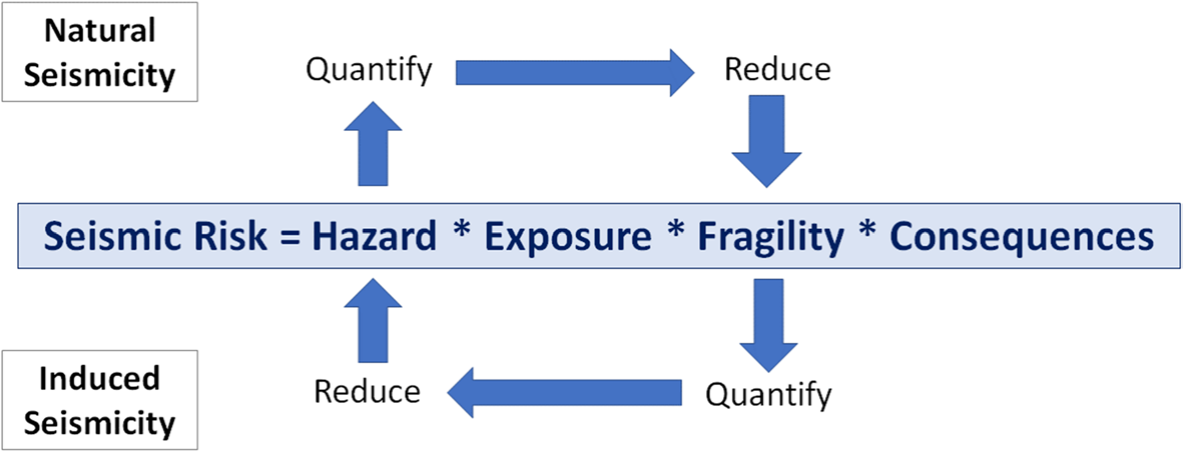
Schematic illustration of the classical approaches for mitigating seismic risk due natural and induced earthquakes by controlling different elements of the risk; in practice, explicit consideration of the exposure and its fragility has often been absent in the management of induced seismicity, replaced instead by vague notions of what levels of hazard are acceptable

Referring back to the earlier discussion, earthquake engineering for natural (or tectonic) seismicity generally seeks to reduce seismic risk to acceptable levels by first quantifying the hazard and then providing sufficient structural resistance to reduce the fragility (i.e., move the curves to the right, as shown in Fig. 5) such that the convolution of hazard and fragility will result in tolerable levels of damage. This does not necessarily mean no damage since designing all structures to resist all levels of earthquake loading without structural damage would be prohibitively expensive. The structural performance targets will generally be related to the consequences of structural damage or failure: single-family dwellings are designed to avoid collapse and preserve life safety; hospitals and other emergency services to avoid damage that would interrupt their operation; and nuclear power plants to avoid any structural damage that could jeopardise the containment of radioactivity. Earthquake engineering in this context is a collaboration between Earth scientists (engineering seismologists) who quantify the hazard and earthquake engineers (both structural and geotechnical) who then provide the required levels of seismic resistance in design. Until now, the way that the risk due to induced seismicity has been managed is very different and has been largely driven by Earth science: implicit assumptions are made regarding the exposure and its fragility, and the risk is then mitigated through schemes to either reduce the hazard at the location of the buildings by either relocating the operations (i.e., changing the exposure) or by controlling the induced seismicity. These two contrasting approaches are illustrated schematically in Fig. 6.

### Randomness and uncertainty

The assessment of earthquake hazard and risk can never be an exact science. Tectonic earthquakes are the result of geological processes that unfold over millennia, yet we have detailed observations covering just a few decades. The first seismographs came into operation around the turn of the twentieth century, but good global coverage by more sensitive instruments came many decades later. This has obvious implications for models of future earthquake activity that are based on extrapolations from observations of the past. Historical studies can extend the earthquake record back much further in time in some regions, albeit with reduced reliability regarding the characteristics of the events, and geological studies can extend the record for larger earthquakes over much longer intervals at specific locations. The first recordings of strong ground shaking were obtained in California in the early 1930s, but networks of similar instruments were installed much later in other parts of the world—the first European strong-motion recordings were registered more than three decades later. Even in those regions where such recordings are now abundant, different researchers derive models that yield different predictions. Consequently, seismic hazard analysis is invariably conducted with appreciable levels of uncertainty, and the same applies to risk analysis since there are uncertainties in every element of the model.

Faced with these uncertainties, there are two challenges for earthquake hazard and risk assessment: on the one hand, to gather data and to derive models that can reduce (or eliminate) the uncertainty, and, on the other hand, to ensure that the remaining uncertainty is identified, quantified, and incorporated into the hazard and risk analyses. In this regard, it is very helpful to distinguish those uncertainties that can, at least in theory, be reduced through the acquisition of new information, and those uncertainties that are effectively irreducible. The former are referred to as *epistemic* uncertainties, coming from the Greek word *ἐπιστήμη* which literally means science or knowledge*,* as they are related to our incomplete knowledge. The term uncertainty traditionally referred to this type of unknown, but the adjective epistemic is now generally applied to avoid ambiguity since the term uncertainty has often also been applied to randomness. Randomness, now usually referred to as aleatory variability (from *alea*, Latin for dice), is thought of as inherent to the process or phenomenon and, consequently, irreducible. In reality, it is more accurate to refer to apparent randomness since it is always characterised by the distribution of data points relative to a specific model (e.g., Strasser et al. 2009; Stafford 2015), and consequently can be reduced by developing models that include the dependence of the predicted parameter on other variables. Consider, for example, a model that predicts ground accelerations as a function of earthquake size (magnitude) and the distance of the recording site from the source of the earthquake. The residuals of the recorded accelerations relative to the predictions define the aleatory variability in the predictions, but this variability will be appreciably reduced if the nature of the surface geology at the recording sites is taken into account, even if this is just a simple distinction between rock and soil sites (Boore 2004). In effect, such a modification to the model isolates an epistemic uncertainty—the nature of the recording site and its influence on the ground acceleration—and thus removes it from the apparent randomness; this, in turn, creates the necessity, when applying the model, to obtain additional information, namely the nature of the surface geology at the target site.

Aleatory variability is generally measured from residuals of data relative to the selected model and is characterised by a statistical distribution. The quantification of epistemic uncertainty requires expert judgement (as discussed in Sect. 6) and is represented in the form of alternative models or distributions of values for model parameters. As is explained in Sect. 3, aleatory variability and epistemic uncertainty are handled differently in seismic hazard analysis and also influence the results in quite distinct ways. What is indispensable is that both types be recognised, quantified and incorporated into the estimation of earthquake hazard and risk.

### Overview of the paper

Following this Introduction, the paper is structured in two parts that deal with natural earthquakes and induced seismicity, with the focus in both parts being the quest for objectivity in the assessment of their associated hazard.

Part I addresses natural earthquakes of tectonic origin, starting with a brief overview of the hazards associated with earthquakes (Sect. 2) followed by an overview of seismic hazard assessment, explaining how it incorporates aleatory variability in earthquake processes, as well as highlighting how hazard is always defined, explicitly or implicitly, in the context of risk (Sect. 3). Section 4 then discusses features of good practice in seismic hazard analysis that can be expected to facilitate acceptance of the result, emphasising especially the importance of capturing epistemic uncertainties. Section 5 discusses the construction of input models for seismic hazard analysis, highlighting recent developments that facilitate the representation of epistemic uncertainty in these inputs. Section 6 then discusses the role of expert judgement in the characterisation of epistemic uncertainty and the evolution of processes to organise multiple expert assessments for this objective. Part I concludes with a discussion of cases in which the outcomes of seismic hazard assessments have met with opposition (Sect. 7), illustrating that undertaking an impartial and robust hazard analysis does not always mean that the results will be treated objectively.

Part II addresses induced seismicity, for which objectivity in hazard and risk assessments can be far more elusive. The discussion begins with a brief overview of induced seismicity and some basic definitions, followed by a discussion of how induced earthquakes can be distinguished from natural earthquakes (Sect. 8), including some examples of when making this distinction has become controversial. Section 9 discusses seismic hazard and risk analysis for induced earthquakes through adaptation of the approaches that have been developed for natural seismicity, including the characterisation of uncertainties. Section 10 then discusses the mitigation of induced seismic risk, explaining the use of traffic light protocols (TLP) as the primary tool used in the scheme illustrated in Fig. 6, but also making the case for induced seismic risk to be managed in the same way as seismic risk due to tectonic earthquakes. Section 11 addresses the fact that for induced seismicity, there is often concern and focus on earthquakes of magnitudes that would generally be given little attention were they of natural origin, by reviewing the smallest tectonic earthquakes that have been known to cause damage. This then leads into Sect. 12 and four case histories of induced earthquakes that did have far-reaching consequences, despite their small magnitude. In every case it is shown that the consequences of the induced seismicity were not driven by physical damage caused by the ground shaking but by other non-technical factors, each one illustrating a failure to objectively quantify and rationally manage the perceived seismic risk. Part II closes with a discussion of the implications of the issues and case histories presented in terms of achieving objective and rational responses to earthquake risk arising from induced seismicity. A number of ideas are put forward that could contribute to a more balanced and objective response to induced earthquakes.

The paper then closes with a brief Discussion and Conclusions section that brings together the key messages from both Part I and Part II.

Finally, a few words are in order regarding the audience to which the paper is addressed. The article is addressed in the first instance to seismologists and engineers, since both of these disciplines are vital to the effective mitigation of earthquake risk (and, I shall argue, the contribution from earthquake engineering to confronting the challenges of induced seismicity has been largely lacking to date). However, if both impartial quantification of earthquake hazard and risk, and objective evaluation of hazard and risk estimates in the formulation of policy are to be achieved, other players need to be involved in the discussions, particularly regulators and operators from the energy sector, who may not have expertise in the field of Earth sciences or earthquake engineering. Consequently, the paper begins with a presentation of some fundamentals so that it can be read as a standalone document by non-specialists, as well as the usual readership of the *Bulletin of Earthquake Engineering*. Readers in the latter category may therefore wish to jump over Sects. 2 and 3 (and may feel that they should have been given a similar warning regarding Sect. 1.1 and 1.2).

Part I: Natural Seismicity

## Earthquakes and seismic hazards

**Fig. 7:**
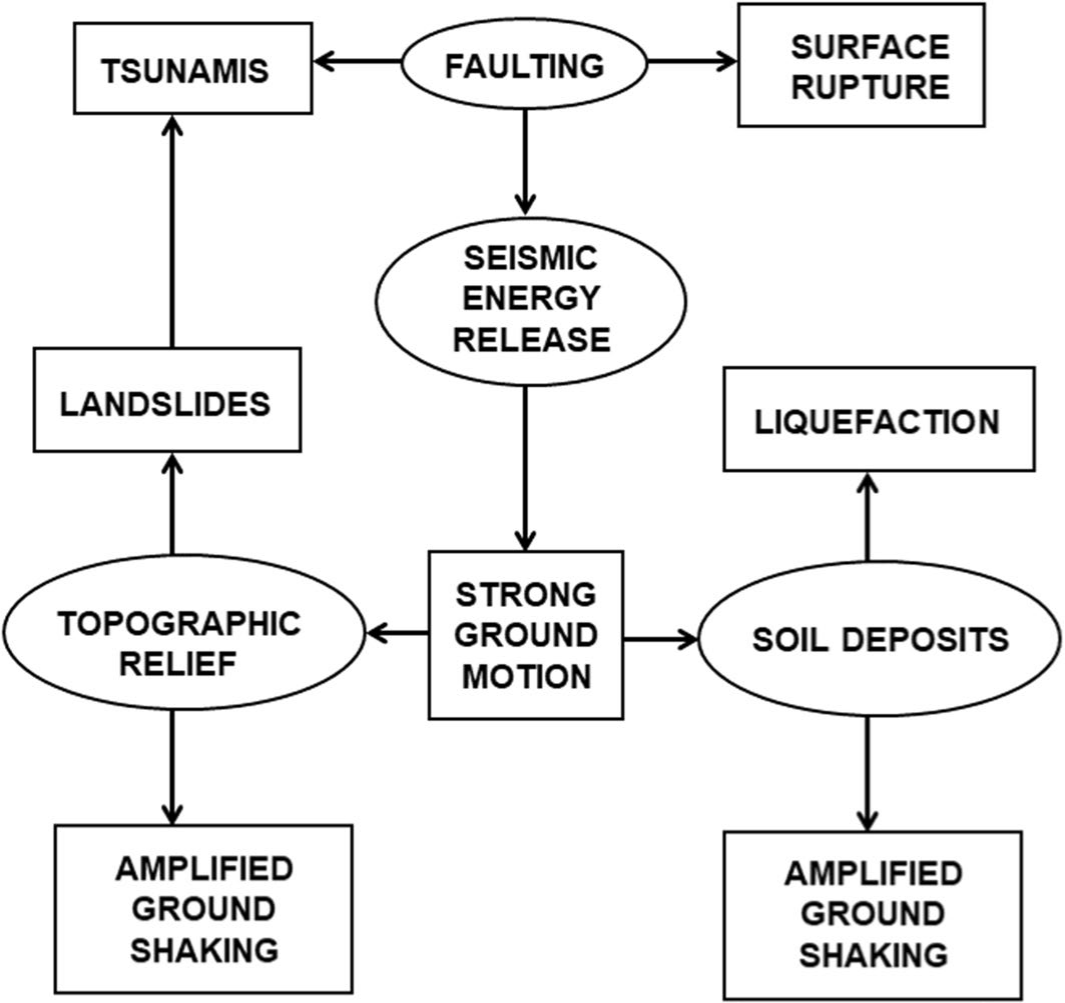
Earthquake processes and their interaction with the natural environment (ellipses) and the resulting seismic hazard (rectangles); adapted from Bommer and Boore (2005)

An earthquake is the abrupt rupture of a geological fault, initiating at a point referred to as the focus or hypocentre, the projection of which on the Earth’s surface is the epicentre. The displacement of the fault relaxes the surrounding crustal rocks, releasing accumulated strain energy that radiates from the fault rupture in the form of seismic waves whose passage causes ground shaking. Figure 7 illustrates the different hazards that can result from the occurrence of an earthquake.

### Fault ruptures

As illustrated in Fig. 7, there are two important hazards directly associated with the fault rupture that is the source of the earthquake: surface fault rupture and tsunami.

#### Surface rupture

**Fig. 8:**
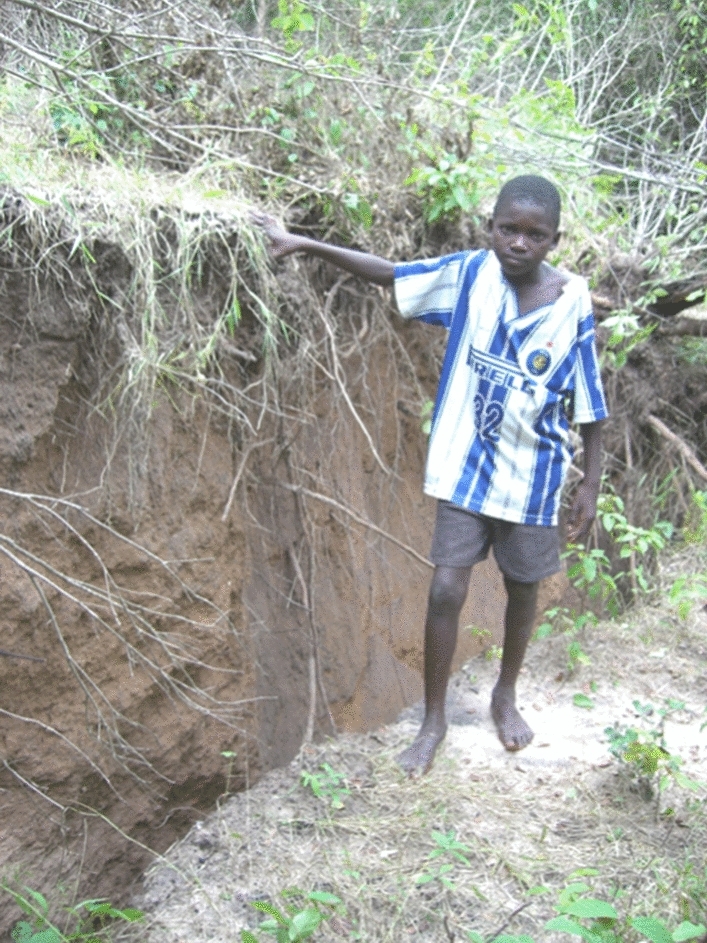
Normal-faulting scarp created by the 2006 Machaze **M** 7 earthquake in Mozambique, which occurred towards the southern end of the East African Rift (Fenton and Bommer 2006). The boy is standing on the hanging block (i.e., the fault dips under his feet) that has moved downwards in the earthquake

**Fig. 9:**
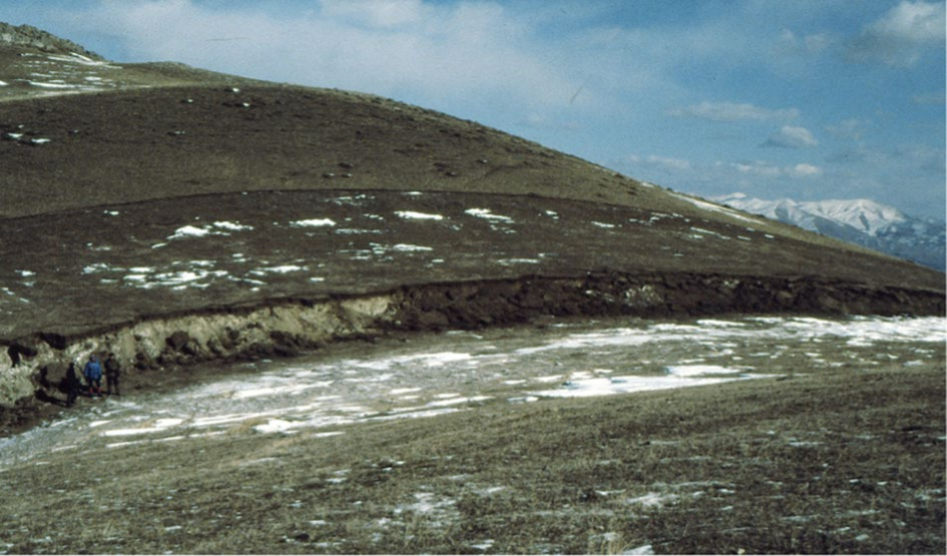
Reverse-faulting scarp in Armenia following the Spitak earthquake of 1988, in the Caucasus mountains (Bommer and Ambraseys 1989). The three people to the left of the figure are on the foot wall (the fault dips away from them) and the hanging wall has moved upwards

The dimensions of fault ruptures grow exponentially with earthquake magnitude, as does the slip on the fault that accompanies the rupture (e.g., Wells and Coppersmith 1994; Strasser et al. 2010; Leonard 2014; Skarlatoudis et al. 2015; Thingbaijam et al. 2017). Similarly, the probability of the rupture reaching the ground surface—at which point it can pose a very serious threat to any structure that straddles the fault trace—also grows with magnitude (e.g., Youngs et al. 2003). The sense of the fault displacement is controlled by the fault geometry and the tectonic stress field in the region: predominantly vertical movement is dip-slip and horizontal motion is strike-slip. Vertical motion is referred to as normal in regions of tectonic extension (Fig. 8) and reverse in regions of compression (Fig. 9).

**Fig. 10:**
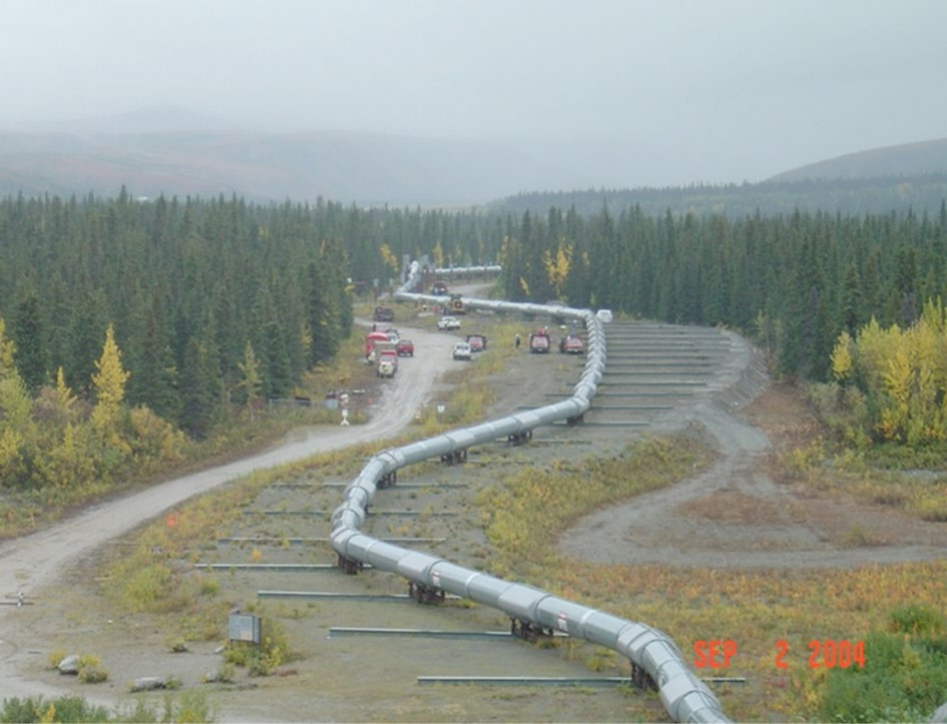
The Trans-Alaska pipeline crossing of the Denali fault, restored to its original configuration following the 2003 Denali earthquake to be able to withstand right-lateral displacement in future earthquakes (Image courtesy of Lloyd S Cluff)

The risk objective in the assessment of surface rupture hazard is generally to avoid locations where this hazard could manifest (in other words, to mitigate the risk by changing the exposure). For safety–critical structures such as nuclear power plants (NPPs), the presence of a fault capable of generating surface rupture would normally be an exclusionary criterion that would disqualify the site. Meehan (1984) relates the story of several potential NPP sites in California that were eventually abandoned when excavations for their foundations revealed the presence of active geological faults. For extended lifeline infrastructure, however, such as roads, bridges, and pipelines, it is often impossible to avoid crossing active fault traces and in such circumstances the focus moves to quantifying the sense and amplitude of potential surface slip, and to allow for this in the design. An outstanding example of successful structural design against surface fault rupture is the Trans-Alaskan Oil Pipeline, a story brilliantly recounted by the late Lloyd Cluff in his Mallet-Milne lecture of 2011. The pipeline crosses the Denali fault and was designed to accommodate up to 6 m of horizontal displacement and 1.5 m of vertical offset. The design was tested in November 2003 by a magnitude **M** 7.9 earthquake associated with a 336-km rupture on the Denali fault, with a maximum slip of 8.8 m. In the area where the pipeline crosses the fault trace, it was freely supported on wide sleepers to allow it to slip and thus avoid the compressional forces that would have been induced by the right-lateral strike-slip motion (Fig. 10). No damage occurred at all and not a drop of oil was spilt and thus a major environmental disaster was avoided: the pipeline transports 2.2 million barrels of crude oil a day. Failure of the pipeline would also have had severe economic consequences since at the time it transported 17% of US crude oil supply and accounted for 80% of Alaska’s economy.

There are also numerous examples of earth dams built across fault traces—the favourable topography allowing the creation of a reservoir often being the consequence of the faults—and designed to accommodate future fault offset (e.g., Allen and Cluff 2000; Mejía 2013). There have also been some spectacular failures causes by fault rupture, such as the Shih-Kang dam that was destroyed by the fault rupture associated with the 199 Chi-Chi earthquake in Taiwan (e.g., Faccioli et al., 2006).

**Fig. 11:**
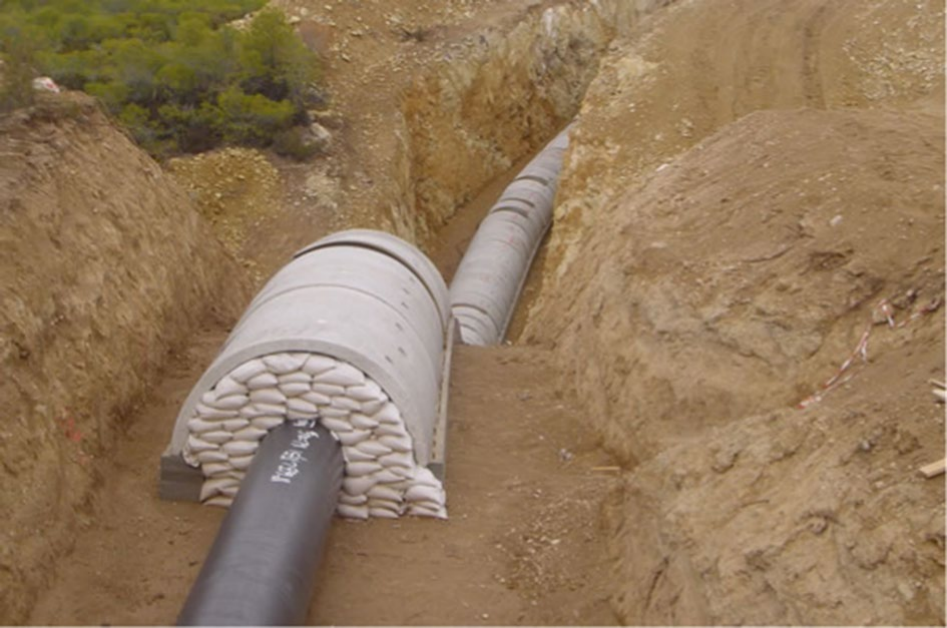
Construction of high pressure gas pipeline from Megara to Corinth, Greece: where the pipeline crosses active faults, it is encased to prevent damage due to fault slip (Image courtesy of Professor George Bouckovalas, NTUA http://users.ntua.gr/gbouck/proj-photos/megara.html)

Accommodating vertical offset associated with dip-slip faults can be even more challenging, but innovative engineering solutions can be found. Figure 11, for example, shows a detail of a high-pressure gas pipeline in Greece at a location where it crosses the trace of a dip-slip fault, and design measures have been added to allow the pipeline to accommodate potential fault slip without compromising the integrity of the conduit.

#### Tsunami

When a surface fault rupture occurs in the seabed, and especially for a reverse or thrust (a reverse fault of shallow dip) rupture typical of subduction zones, the displacement of a large body of water above the fault can create a gravity wave of small amplitude and great wavelength that travels across the ocean surface at a velocity equal to $\sqrt{gd}$, where *g* is the acceleration due to gravity (9.81 m/s^2^) and *d* is the depth of the ocean. As the wave approaches the shore, the speed of the wave reduces with the water depth and the wave height grows to maintain the momentum, creating what is called a *tsunami*, which is a Japanese word meaning ‘harbour wave’. Tsunamis can be the most destructive of all earthquake effects, as was seen in the 2004 Boxing Day **M** 9.2 earthquake that originated off the coast of Indonesia (e.g., Fujii and Satake 2007) and caused loss of life as far away as East Africa (Obura 2006), and the tsunami that followed the 2011 Tōhoku **M** 9.0 earthquake in Japan (e.g., Saito et al. 2011), which caused the loss of 20,000 lives. As indicated in Fig. 7, tsunamis can also be generated by submarine landslides (e.g., Ward 2001; Harbitz et al. 2006; Gusman et al. 2019), an outstanding example of which was the Storegga slide in the North Sea, assumed to have been triggered by an earthquake, that generated a tsunami that inundated areas along the east coast of Scotland (e.g., Dawson et al. 1988).

The estimation of tsunami hazard generally focuses on potential wave heights and run-up, the latter referring to the highest elevation on land to which the water rises. Such parameters can inform design or preventative measures, including elevated platforms and evacuation routes. Insufficient sea wall height at the Fukushima Daiichi NPP in Japan led to inundation of the plant due to the tsunami that followed the Tōhoku earthquake, leading to a severe nuclear accident despite the fact that the plant had survived the preceding ground shaking without serious damage. There can be significant scope for reducing loss of life due to tsunami through early warning systems that alert coastal populations to an impending wave arrival following a major earthquake (e.g., Selva et al. 2021); for tsunami the lead times can be much longer than early warning systems for ground shaking, for which reason these can be of great benefit.

### Ground shaking

On a global scale, most earthquake destruction is caused by the strong shaking of the ground associated with the passage of seismic waves, and this shaking is also the trigger for the collateral geotechnical hazards discussed in Sect. 2.3. The focus of most seismic hazard assessments is to quantify possible levels of ground shaking, which provides the basis for earthquake-resistant structural design.

#### Intensity

**Fig. 12:**
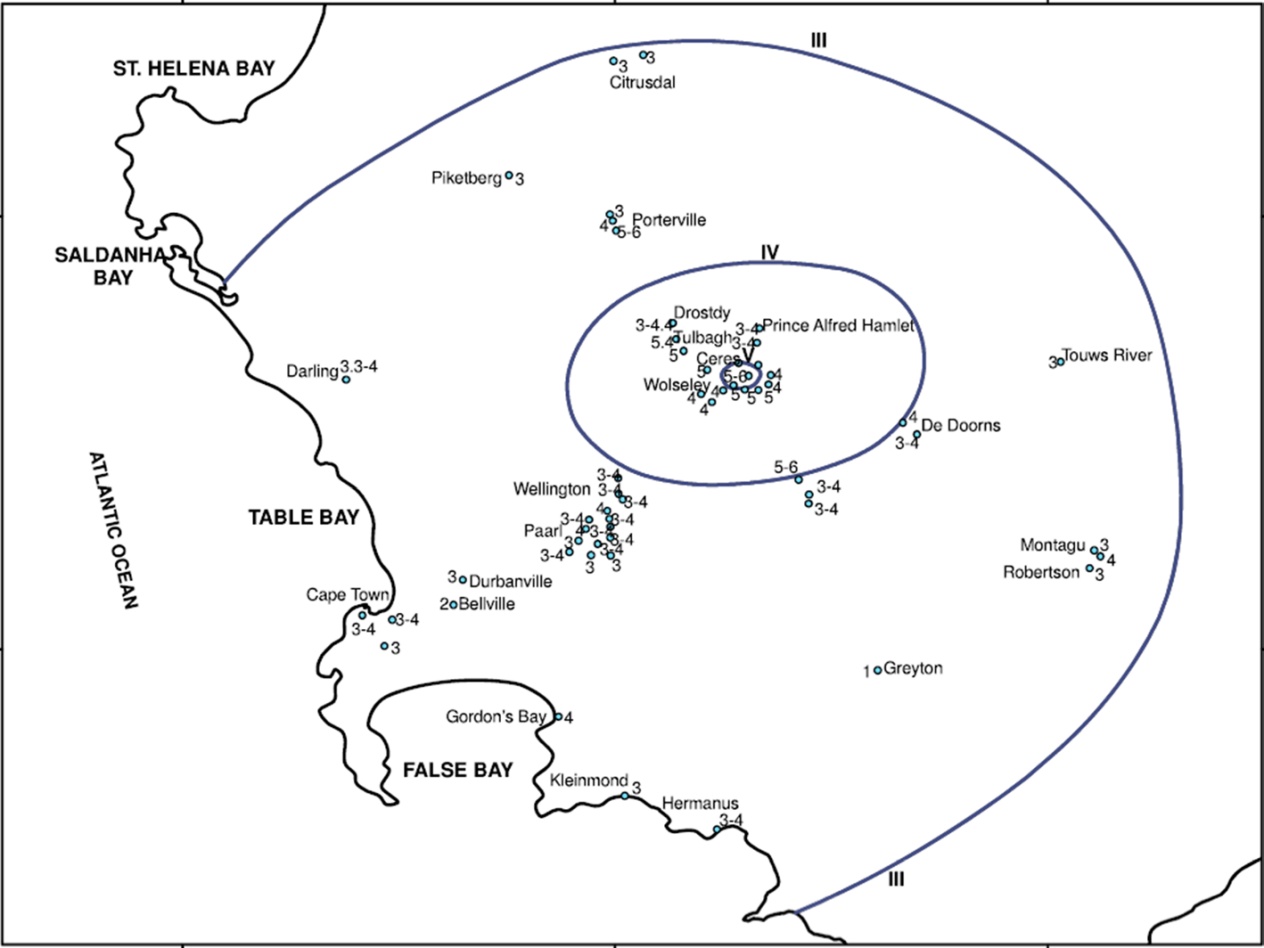
Isoseismal map for an earthquake in South Africa (Midzi et al. 2013). The IDPs for individual locations are shown in Arabic numerals

Macroseismic intensity is a parameter that reflects the strength of the ground shaking at a given location, inferred from observations rather than instrumental measurements. There are several scales of intensity, the most widely used defining 12 degrees of intensity (Musson et al. 2010), such as the European Macroseismic Scale, or EMS (Grünthal 1998). For the lower degrees of intensity, the indicators are primarily related to the response of humans and to the movement of objects during the earthquakes; as the intensity increases, the indicators are increasingly related to the extent of damage in buildings of different strength. The intensity assigned to a specific location should be based on the modal observation and is often referred to as an intensity data point (IDP). Contours can be drawn around IDPs and these are called isoseismals, which enclose areas of equal intensity. The intensity is generally written as a Roman numeral, which reinforces that notion that it is an index and should be treated as an integer value. An isoseismal map, such as the one shown in Fig. 12, conveys both the maximum strength of the earthquake shaking and the area over which the earthquake was felt, and provides a very useful overview of an earthquake. Intensity can be very useful for a number of purposes, including the inference of source location and size for earthquakes that occurred prior to the dawn of instrumental seismology (e.g., Strasser et al. 2015). However, for the purposes of engineering design to mitigate seismic risk, intensity is of little use and recourse is made to instrumental recordings of the strong ground shaking.

#### Accelerograms and ground-motion parameters

**Fig. 13:**
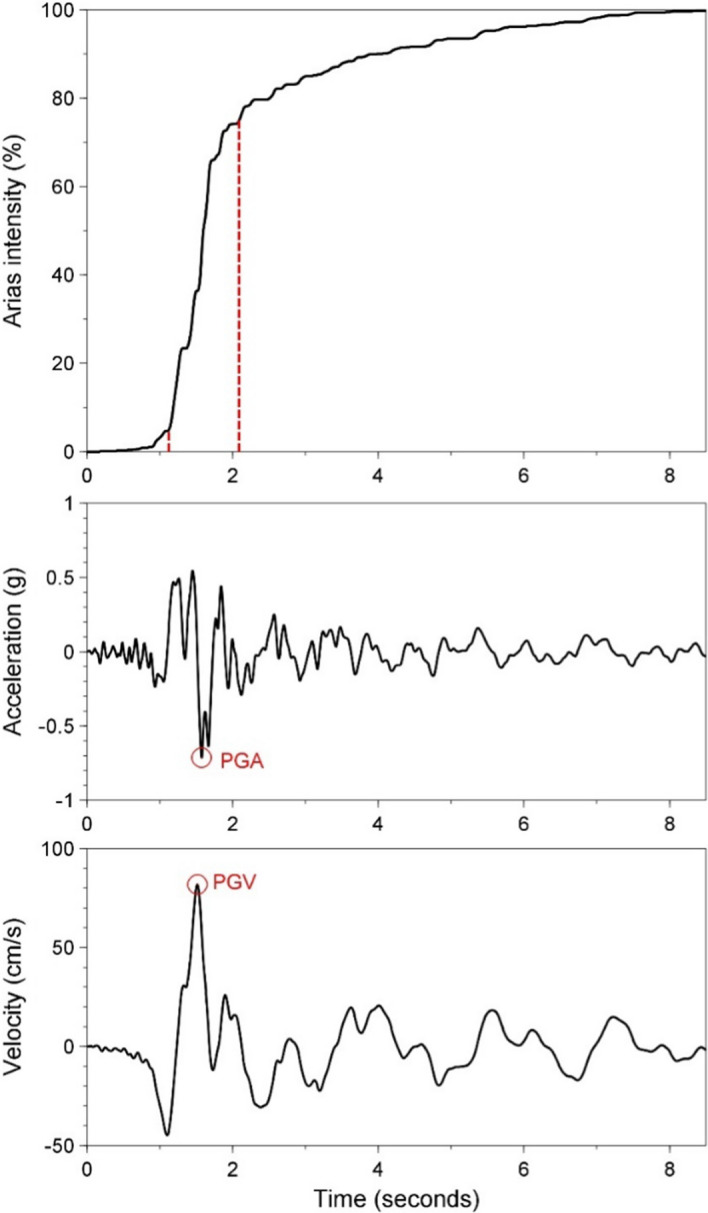
The acceleration and velocity time-series from the recording at the CIG station of the **M** 5.7 San Salvador, El Salvador, earthquake of October 1986. The upper plot shows the accumulation of Arias intensity and the significant duration (of 0.96 s) based on the interval between obtaining 5% and 75% of the total Arias intensity

The development and installation of instruments capable of recording the strong ground shaking caused by earthquakes was a very significant step in the evolution of earthquake engineering since it allowed the detailed characterisation of these motions as input to structural analysis and design. The instruments are called accelerographs since they generate a record of the ground acceleration against time, which is known as an accelerogram. Many different parameters are used to characterise accelerograms, each of which captures a different feature of the shaking. The mostly widely used parameter is the peak ground acceleration, PGA, which is simply the largest absolute amplitude on the accelerogram. Integration of the accelerogram over time generates the velocity time-history, from which the peak ground velocity, PGV, is measured in the same way (Fig. 13). In many ways, PGV is a superior indicator of the strength of the shaking to PGA (Bommer and Alarcón 2006).

Another indicator of the strength of the shaking is the Arias intensity, which is proportional to the integral of the acceleration squared over time (Fig. 13). Arias intensity has been found to be a good indicator of the capacity of ground shaking to trigger instability in both natural and man-made slopes (Jibson and Keefer 1993; Harper and Wilson 1995; Armstrong et al. 2021).

The duration of shaking or number of cycles of motion can also be important parameters to characterise the shaking. Numerous definitions have been proposed for the measurement of both of these parameters (Bommer and Martinez-Pereira 1999; Hancock and Bommer 2005). The most commonly used measure of duration is called the significant duration and it is based on the accumulation of Arias intensity, defined as the time elapsed between reaching 5% and 75% or 95% of the total. Figure 13 illustrates this measure of duration.

**Fig. 14:**
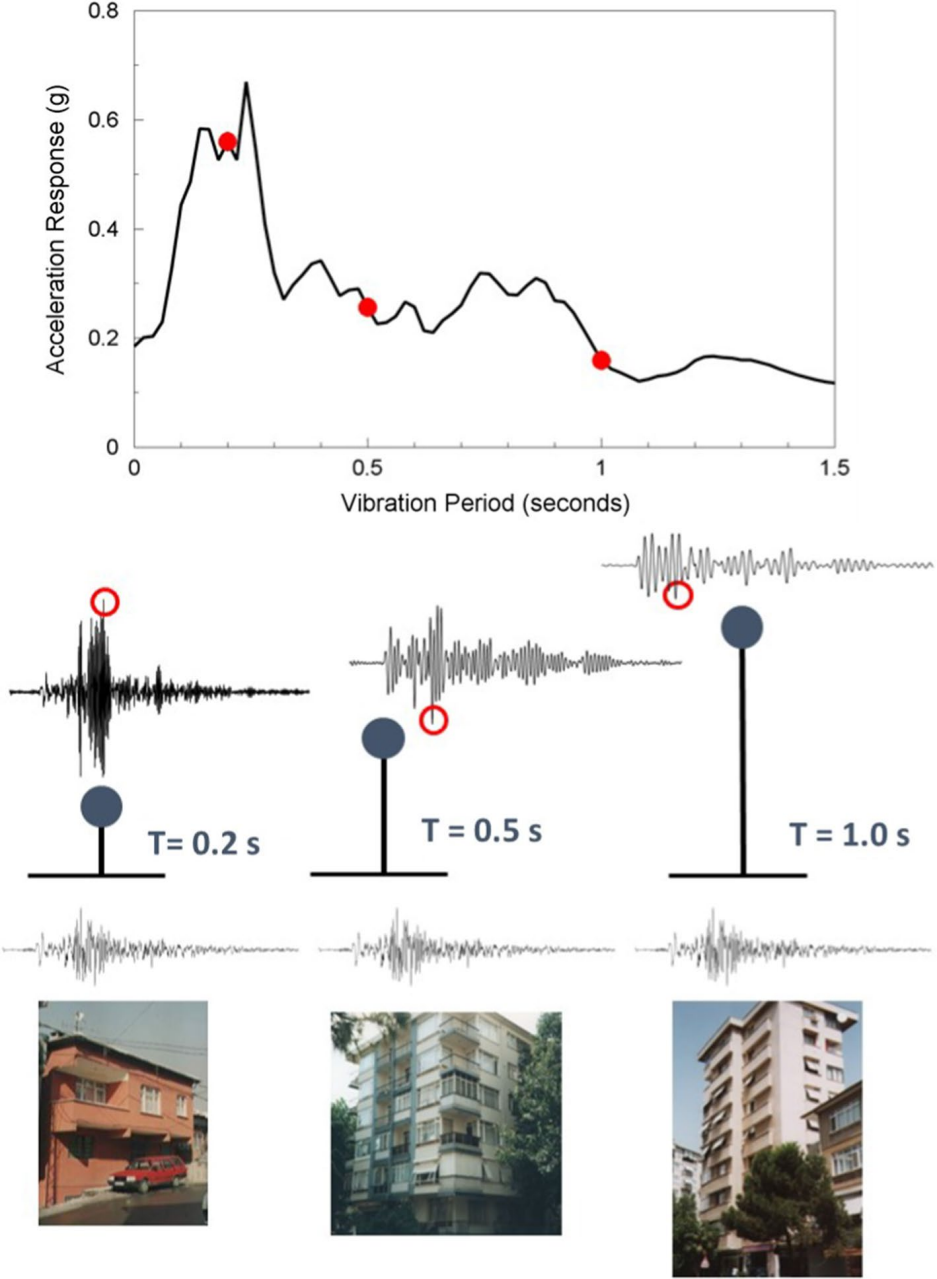
The concept of the acceleration response spectrum: structures (lowest row) are represented as equivalent single-degree-of-freedom oscillators characterised by their natural period of vibration and equivalent viscous damping (middle row), which are then excited by the chosen accelerogram and the response of the mass calculated. The maximum response is plotted against the period of the oscillator and the complete response spectrum of the accelerogram is constructed by repeating for a large number of closely-spaced periods; building photographs from Spence et al. (2003)

The response of a structure to earthquake shaking depends to a large extent on the natural vibration frequency of the structure and the frequency content of the motion. As a crude rule-of-thumb, the natural vibration period of a reinforced concrete structure can be estimated as the number of storeys divided by 10, although this can also be calculated more accurately considering the height and other characteristics of the structure (Crowley and Pinho 2010). The response spectrum is a representation of the maximum response experienced by single-degree-of-freedom oscillators with a given level of damping (usually assumed to be 5% of critical) to a specific earthquake motion. The concept of the response spectrum is illustrated in Fig. 14. The response spectrum is the basic representation of ground motions used in all seismic design, and all seismic design codes specify a response spectrum as a function of location and site characteristics. The response spectrum can be scaled for damping ratios other than the nominal 5% of critical although the scaling factors depend not only on the target damping value, but also on the duration or number of cycles of motion (Bommer and Mendis 2005; Stafford et al. 2008a).

#### Ground-motion prediction models

**Fig. 15:**
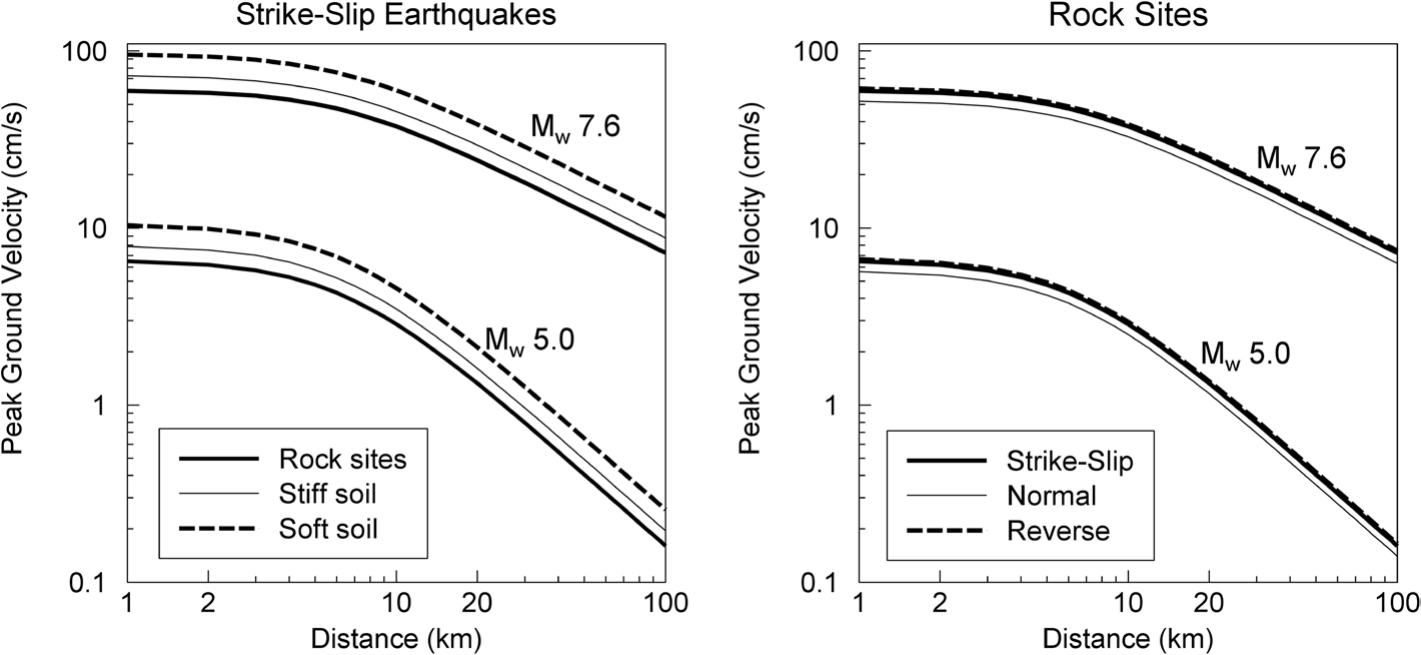
Predictions of PGV as a function of distance for two magnitudes showing the influence of site classification (left) and style-of-faulting (right) (Akkar and Bommer 2010)

An essential element of any seismic hazard assessment is a model to estimate the value of the ground-motion parameter of interest at a particular location as a result of a specified earthquake scenario. The models reflect the influence of the source of the earthquake (the energy release), the path to the site of interest (the propagation of the seismic waves), and the characteristics of the site itself (soft near-surface layers will modify the amplitude and frequency of the waves). The parameters that are always included in such a model are magnitude (source), distance from the source to the site (path), and a characterisation of the site. Early models used distance from the epicentre (R_epi_) or the hypocentre (R_hyp_) but these distance metrics ignore the dimensions of the fault rupture and therefore are not an accurate measure of the separation from the source for sites close to larger earthquakes associated with extended fault ruptures. More commonly used metrics in modern models are the distance to the closest point on the fault rupture (R_rup_) or the shortest horizontal distance to the projection of the fault rupture onto the Earth’s surface, which is known as the Joyner-Boore distance (Joyner and Boore 1981) or R_jb_. Site effects were originally represented by classes, sometimes as simple as distinguishing between ‘rock’ and ‘soil’, but nowadays are generally represented by explicit inclusion of the parameter V_S30_, which is the shear-wave velocity (which is a measure of the site stiffness) corresponding to the travel time of vertically propagating shear waves over the uppermost 30 m at the site. The reference depth of 30 m was selected because of the relative abundance of borehole data to this depth rather than any particular geophysical significance. The modelling of site effects has sometimes included additional parameters to represent the depth of sediments, such as Z_1.0_ or Z_2.5_ (the depths at which shear-wave velocities of 1.0 and 2.5 km/s are encountered). The more advanced models also include the non-linear response of soft soil sites for large-amplitude motions, often constrained by site response models developed separately (Walling et al. 2008; Seyhan and Stewart 2014). Another parameter that is frequently included is the style-of-faulting, SoF (e.g., Bommer et al. 2003). Figure 15 shows an example of predictions from a model for PGV, showing the influence of magnitude, distance, site classification and style-of-faulting.

**Fig. 16:**
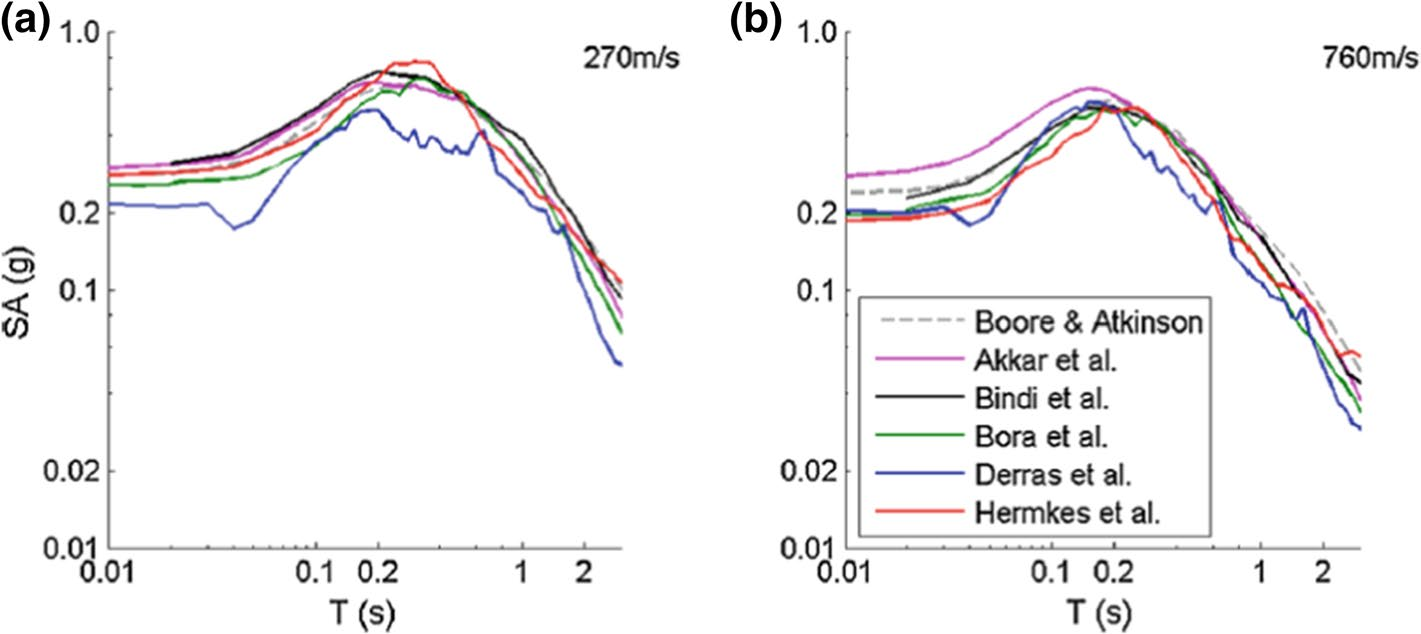
Acceleration response spectra predicted by five European models and one from California for sites with **a** V_S30_ = 270 m/s and **b** V_S30_ = 760 m/s for an earthquake of **M** 7 at 10 km (Douglas et al. 2014a)

By developing a series of predictive models for response spectral accelerations at a number of closely spaced oscillator periods, complete response spectra can be predicted for a given scenario. Figure 16 shows predicted response spectra for rock and soil sites at 10 km from a magnitude **M** 7 earthquake obtained from a suite of predictive models derived for Europe and the Mediterranean region, compared with the predictions from the Californian model of Boore and Atkinson (2008), which was shown to provide a good fit to European strong-motion data (Stafford et al. 2008b). The range of periods for which reliable response spectral ordinates can be generated depends on the signal-to-noise ratio of the accelerograms, especially for records obtained by older, analogue instruments, although processing is generally still required for modern digital recordings as well (Boore and Bommer 2005). The maximum usable response period of a processed record depends on the filters applied to remove those parts of the signal that are considered excessively noisy (Akkar and Bommer 2006).

There are many different approaches to developing predictive models for different ground-motion parameters (Douglas and Aochi 2008) but the most commonly used are regression on empirical datasets of ground-motion recordings, and stochastic simulations based on seismological theory (e.g., Boore 2003). The former is generally used in regions with abundant datasets of accelerograms, whereas simulations are generally used in regions with sparse data, where recordings from smaller earthquakes are used to infer the parameters used in the simulations. Stochastic simulations can also be used to adjust empirical models developed in a data-rich region for application to another region with less data, which preserves the advantages of empirical models (see Sect. 5.2). A common misconception regarding empirical models is that their objective is to reproduce as accurately as possible the observational data. The purpose of the models is rather to provide reliable predictions for all magnitude-distance combinations that may be considered in seismic hazard assessments, including those that represent extrapolations beyond the limits of the data. The empirical data provides vital constraint on the models, but the model derivation may also invoke external constraints obtained from simulations or independent analyses.

At this point, a note is in order regarding terminology. Predictive models for ground-motion parameters were originally referred to as attenuation relations (or even attenuation laws), which is no longer considered an appropriate name since the models describe the scaling of ground-motion amplitudes with magnitude as well as the attenuation with distance. This recognition prompted the adoption of the term ground motion prediction equations or GMPEs. More recently, there has been a tendency to move to the use of ground motion prediction models (GMPMs) or simply ground motion models (GMMs); in the remainder of this article, GMM is used.

**Fig. 17:**
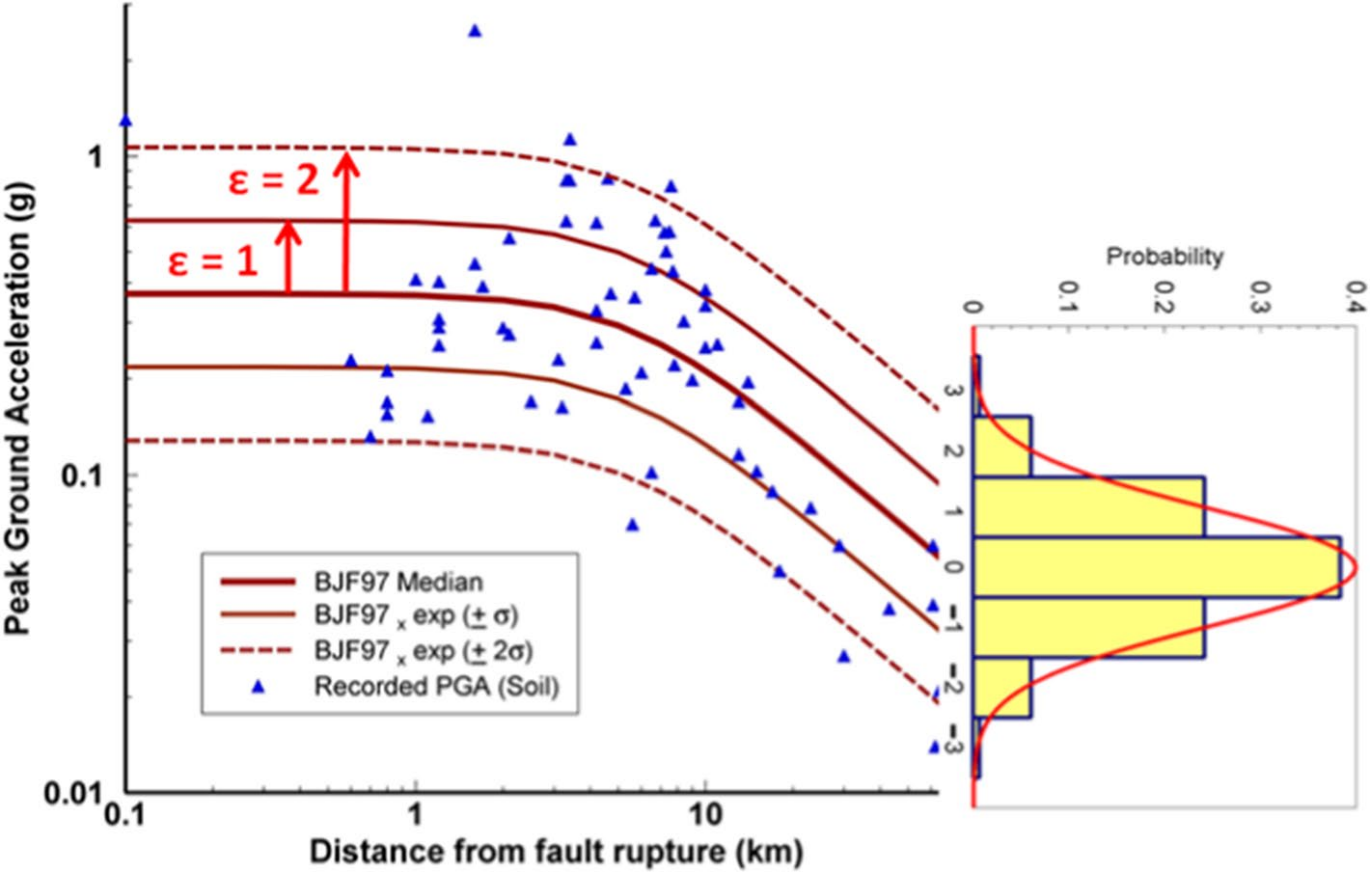
Recorded PGA values at soil sites from the 2004 Parkfield earthquake in California, compared to predictions from the California GMM of Boore et al. (1997), illustrating the Gaussian distribution of the logarithmic residuals. Adapted from Bommer and Abrahamson (2006)

Predicted curves such as those shown in Figs. 15 and 16 paint an incomplete picture of GMMs. When an empirical GMM is derived, the data always displays considerable scatter with respect to the predictions (Fig. 17). For a given model, this scatter is interpreted as aleatory variability. When the regressions are performed on the logarithmic values of the ground-motion parameter, the residuals—observed minus predicted values—are found to be normally distributed (e.g., Jayaram and Baker 2008). The distribution of the residuals can therefore be characterised by the standard deviation of these logarithmic residuals, which is generally represented by the Greek letter $\sigma$ (sigma). Consequently, GMMs do not predict unique values of the chosen ground-motion parameter, *Y*, for a given scenario, but rather a distribution of values:

1 $$\mathrm{log}\left(Y\right)=f\left(M,R,{V}_{S30}, SoF\right)+\varepsilon \sigma $$

where $\varepsilon$ is the number of standard deviations above or below the mean (Fig. 17). If $\varepsilon$ is set to zero, the GMM predicts median values of Y, which have a 50% probability of being exceeded for the specified scenario; setting $\varepsilon =1$ yields the mean-plus-one-standard deviation value, which will be appreciably higher and have only a 16% probability of being exceeded.

Typical values of the standard deviation of logarithmic ground-motion residuals are generally such that 84-percentile values of motion are between 80 and 100% larger than the median predictions. The expansion of ground-motion datasets and the development of more sophisticated models has not resulted in any marked reduction of sigma values (Strasser et al., 2009); indeed, the values associated with recent models are often larger than those that were obtained for earlier models (e.g., Joyner and Boore 1981; Ambraseys et al. 1996) but this may be the result of early datasets being insufficiently large to capture the full distribution of the residuals. Progress in reducing sigma values has been made by decomposition of the variability into different components, which begins with separating the total sigma into between-event ($\tau$) and within-event ($\phi$) components, which are related by the following expression:

2 $$\sigma = \sqrt{{\tau }^{2}+{\phi }^{2}}$$

**Fig. 18:**
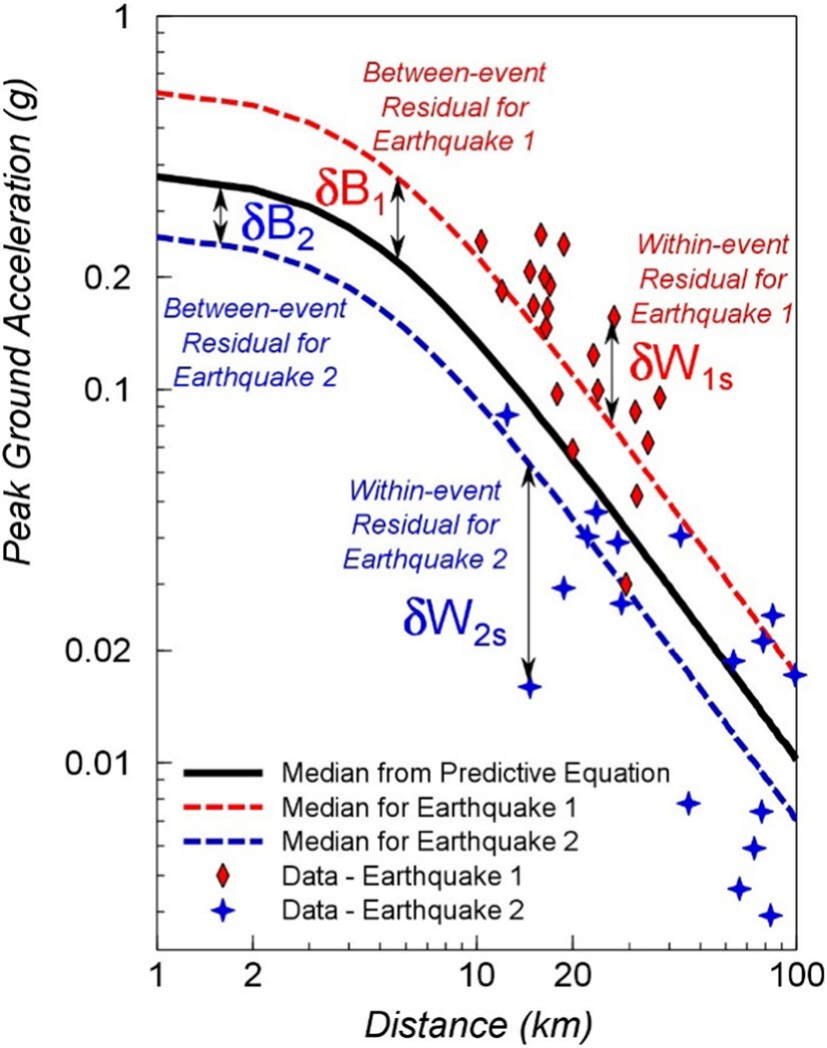
Conceptual illustration of between-event and within-event residuals (Al Atik et al. 2010)

The first term corresponds to how the average level of the ground motions varies from one earthquake of a given magnitude to another, whereas the latter reflects the spatial variability of the motions. The concepts are illustrated schematically in Fig. 18: $\tau$ is the standard deviation of the $\delta B$ residuals and $\phi$ the standard deviation of the $\delta W$ residuals. Additional decomposition of these two terms is then possible, in which it is possible to identify and separate elements that in reality correspond to epistemic uncertainties (i.e., repeatable effects that can be constrained through data acquisition and modelling) rather than aleatory variability; such decomposition of sigma is discussed further in Sect. 5.

Several hundred GMMs, which predict all of the ground-motion parameters described in Sect. 2.2 and are derived for application to many different regions of the world, have been published. Dr John Douglas has provided excellent summaries of these models (Douglas 2003; Douglas and Edwards 2016), and also maintains a very helpful online resource that allows users to identify all currently published GMMs (www.gmpe.org.uk).

### Geotechnical hazards

While the single most important contributor to building damage caused by earthquakes is ground shaking, damage and disruption to transportation networks and utility lifelines is often the result of earthquake-induced landslides and liquefaction (Bird and Bommer 2004).

#### Landslides

**Fig. 19:**
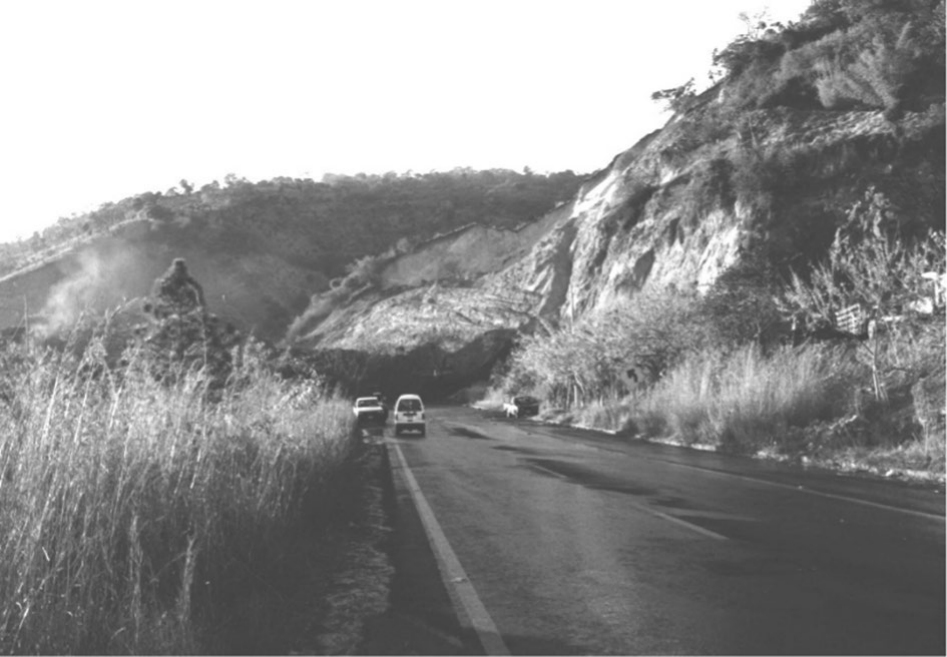
Major landslide triggered by the El Salvador earthquake of January 2001 (Bommer and Rodriguez 2002); another landslide triggered in Las Colinas by this earthquake killed around 500 people

Landslides are frequently observed following earthquakes and can be a major contributor to destruction and loss of life (Fig. 19).

The extent of this collateral hazard depends on the strength of earthquake as reflected by the magnitude (e.g., Keefer 1984; Rodrıguez et al. 1999), but it also depends strongly on environmental factors such as topography, slope geology, and precedent rainfall. Assessment of the hazard due to earthquake-induced landslides begins with assessment of shaking hazard since this is the basic trigger. In a sense, it can be compared with risk assessment as outlined in Sect. 1.1, with the exposure represented by the presence of slopes, and the fragility by the susceptibility of the slopes to become unstable due to earthquakes (which is reflected by their static factor of safety against sliding). Indeed, Jafarian et al. (2021) present fragility functions for seismically induced slope failures characterised by different levels of slope displacement as a function of measures of the ground shaking intensity.

#### Liquefaction

**Fig. 20:**
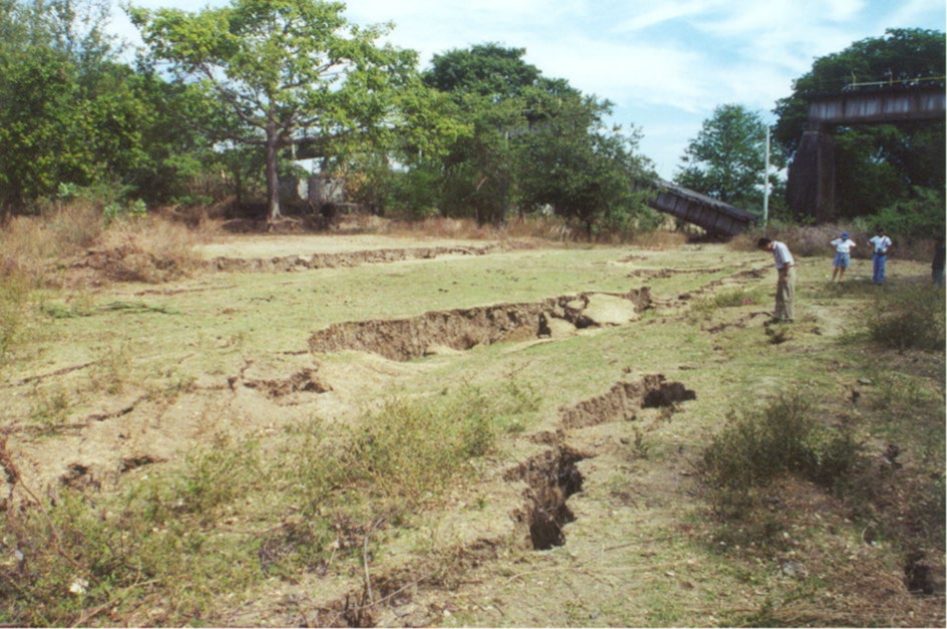
Lateral spreading on the bank of the Lempa River in El Salvador due to liquefaction triggered by the **M** 7.7 subduction-zone earthquake of January 2001; notice the collapsed railway bridge in the background due to the separation of the piers caused by the spreading (Bommer et al. 2002)

Liquefaction triggering is a phenomenon that occurs in saturated sandy soils during earthquake shaking, which involves the transfer of overburden stress from the soil skeleton to the pore fluid, with a consequent increase in pore water pressure and reduction in effective stress. This stress transfer is due to the contractive tendencies of the soil skeleton during earthquake shaking. Once liquefied, the shear resistance of the soil drastically reduces and the soil effectively behaves like a fluid, which can result in structures sinking into the ground. Where there is a free face such as a river or shoreline, liquefaction can lead to lateral spreading (Fig. 20). Liquefaction can result in buildings becoming uninhabitable and can also cause extensive disruption, especially to port and harbour facilities. However, there are no documented cases of fatalities resulting from soil liquefaction, unless one includes flow liquefaction (e.g., de Lima et al. 2020;).

As with landslide hazard assessment, the assessment of liquefaction triggering hazard can also be compared to risk analysis, with the shaking once again representing the hazard, the presence of liquefied soils the exposure, and the susceptibility of these deposits to liquefaction the fragility. In the widely used simplified procedures (e.g., Seed and Idriss 1971; Whitman 1971; Idriss and Boulanger 2008; Boulanger and Idriss 2014), the ground motion is represented by PGA and a magnitude scaling factor, MSF, which is a proxy for the number of cycles of motion.

Geyin and Maurer (2020) present fragility functions for the severity of liquefaction effects as a function of a parameter that quantifies the degree of liquefaction triggering. Structural fragility functions can be derived in terms of the resulting soil displacement (Bird et al. 2006) or another measure of the liquefaction severity (Di Ludovico et al. 2020), so that liquefaction effects can be incorporated into seismic risk analyses although this requires* in situ* geotechnical data and information regarding the foundations of buildings in the area of interest (Bird et al. 2004).

## Seismic hazard and risk analysis

In this section, I present a brief overview of seismic hazard assessment, focusing exclusively on the hazard of ground shaking, highlighting what I view to be an inextricable link between hazard and risk, and also emphasising the issue of uncertainty, which is a central theme of this paper. For reasons of space, the description of hazard and risk analysis is necessarily condensed, and I would urge the genuinely interested reader to consider three textbooks for more expansive discussions of the fundamentals. *Earthquake Hazard Analysis: Issues and Insights* by Reiter (1990) remains a very readable and engaging overview of the topic and as such is an ideal starting point. The monograph *Seismic Hazard and Risk Analysis* by McGuire (2004) provides a succinct and very clear overview of these topics. For an up-to-date and in-depth treatment of these topics, I strongly recommend the book *Seismic Hazard and Risk Analysis* by Baker et al. (2021)—I have publicly praised this tome in a published review (Bommer 2021) and I stand by everything stated therein.

### Seismic hazard analysis

The purpose of a seismic hazard assessment is to determine the ground motions to be considered in structural design or in risk estimation. Any earthquake hazard assessment consists of two basic components: a model for the source of future earthquakes and a model to estimate the ground motions at the site due to each hypothetical earthquake scenario. Much has been made over the years of the choice between deterministic and probabilistic approaches to seismic hazard assessment. In a paper written some 20 years ago (Bommer 2002), I described the vociferous exchanges between the proponents of deterministic seismic hazard analysis (DSHA) and probabilistic seismic hazard analysis (PSHA) as “*an exaggerated and obstructive dichotomy*”. While I would probably change many features of that article if it were being written today, I think this characterisation remains valid for the simple reason that it is practically impossible to avoid probability in seismic hazard analysis. Consider the following case: imagine an important structure very close (< 1 km) to a major geological fault that has been found to generate earthquakes of **M** 7 on average every ~ 600 years (this is actually the situation for the new Pacific locks on the Panama Canal, as described in Sect. 7.2). Assuming the structure has a nominal design life in excess of 100 years, it would be reasonable to assume that the fault will generate a new earthquake during the operational lifetime (especially if the last earthquake on the fault occurred a few centuries ago, as is the case in Panama) and therefore the design basis would be a magnitude 7 earthquake at a distance of 1 km. However, to calculate the design response spectrum a decision needs to be made regarding the exceedance level at which the selected GMM should be applied: if the median motions are adopted (setting $\varepsilon =0$), then in the event of the earthquake occurring, there is a 50% probability that the design accelerations will be exceeded. If instead the 84-percentile motions are used (mean plus one standard deviation), there will be a 1-in-6 chance of the design accelerations being exceeded. The owner of the structure would need to choose the level commensurate with the desired degree of safety, and this may require more than one standard deviation on the GMM. Whatever the final decision, the hazard assessment now includes a probabilistic element (ignoring the variability in the GMM and treating it as a deterministic model, which implies a 50% probability of exceedance, does not make the variability disappear).

If a probabilistic framework is adopted, the decision regarding the value of $\varepsilon$ would take into account the recurrence interval of the design earthquake (in this case, 600 years) to choose the appropriate GMM exceedance level: the median level of acceleration would have a return period of 1,200 (600/0.5) years, whereas for the 84-percentile motions, the return period would be 3,600 years. If the target return period were selected as 10,000 years, say, then the response spectrum would need to be obtained by including 1.55 standard deviations of the GMM, yielding accelerations at least 2.5 times larger than the median spectral ordinates.

**Fig. 21:**
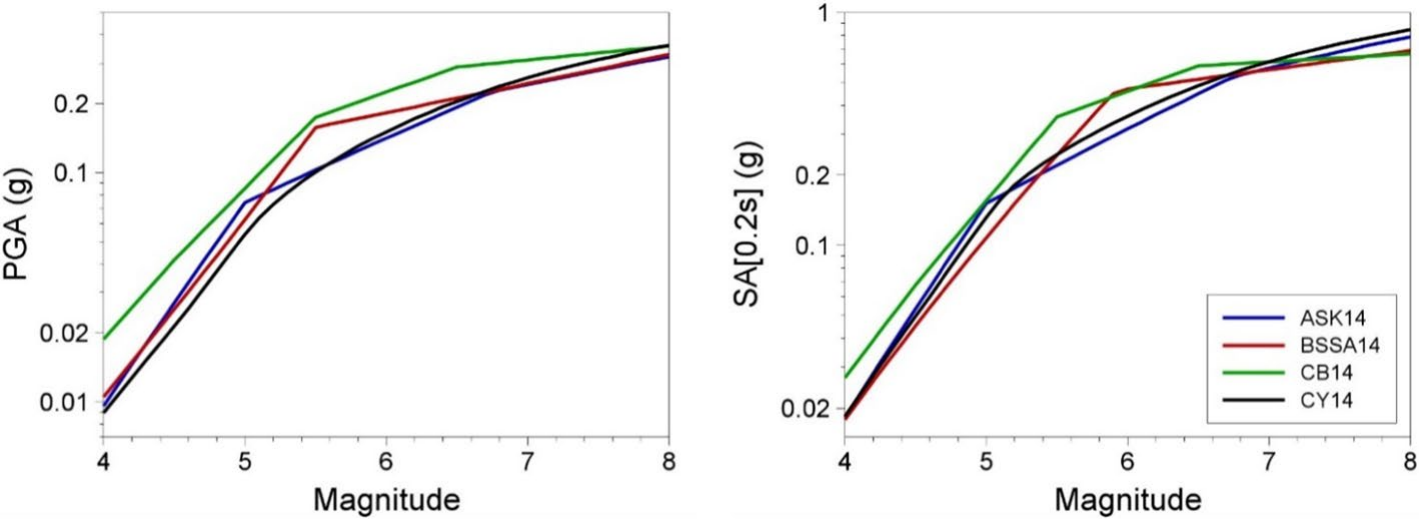
Scaling of PGA (left) and spectral acceleration at 0.2 s (right) with magnitude for a rock (V_S30_ = 760 m/s) site at 10 km using four NGA-West2 GMMs: Abrahamson et al. (2014), Boore et al. (2014), Campbell and Bozorgnia (2014) and Chiou and Youngs (2014)

In practice, most seismic design situations are considerably more complex in terms of the seismic sources and the earthquakes contributing to the hazard than the simple case described above. For example, the site hazard could be still be dominated by a single geological fault, located a few kilometres away from the site at its closest approach, but of considerable length (such that individual earthquakes do not rupture the full length of the fault and will thus not necessarily occur on the section of the fault closest to the site), and which is capable of generating earthquakes of different magnitudes, the larger earthquakes occurring less frequently (i.e., having longer average recurrence intervals) than the smaller events. A deterministic approach might propose to assign the largest magnitude that the fault is considered capable of producing to a rupture adjacent to the target site. However, this would ignore two important considerations, the first is that the smaller earthquakes are more frequent (as a rule-of-thumb, there is a tenfold increase in the earthquake rate for every unit reduction in magnitude) and more frequent earthquakes can be expected to sample higher values of $\varepsilon$, or expressed another way, the more earthquakes of a particular size that occur, the more likely they are to generate higher-than-average levels of ground shaking. The second consideration is that ground-motion amplitudes do not increase linearly with increasing earthquake magnitude, as shown in Fig. 21. Consequently, more frequent scenarios of **M** 6, sampling higher $\varepsilon$ values, could result in higher motions at the site than scenarios of **M** 7. Of course, the rate could simply be ignored, and a decision could be taken to base the design on the largest earthquake, but the rationale—which is sometimes invoked by proponents of DSHA—would be that by estimating the hazard associated with the worst-case scenario one effectively envelopes the various possibilities. However, for this to be true, the scenario would need to correspond to the genuine upper bound of all scenarios, which would mean placing the largest earthquake the fault could possibly produce at the least favourable location, and then calculating the ground motions at least 3 or 4 standard deviations above the median. In most cases, such design motions would be prohibitive and in practice seismic hazard assessment always backs away from such extreme scenarios.

The scenario of a single active fault dominating all hazard contributions is a gross simplification in most cases since there will usually be several potential sources of future earthquakes that can influence the hazard at the site. Envisage, for example, a site in a region with several seismogenic faults, including smaller ones close to the site and a large major structure at greater distance, all having different slip rates. A classical DSHA would simply estimate the largest earthquake that could occur on each fault (thus defining the magnitude, M) and associate it with a rupture located as close to the site as possible (which then determines the distance R); for each M-R pair, the motions at the site would then be calculated with an arbitrarily chosen value of $\varepsilon$ and the final design basis would be the largest accelerations (although for different ground-motion parameters, including response spectral ordinates at different periods, different sources may dominate). In early practice, $\varepsilon$ was often set to zero, whereas more recently it became standard practice to adopt a value of 1. If one recognises that the appropriate value of this parameter should reflect the recurrence rate of the earthquakes, and also takes account of the highly non-linear scaling of accelerations with magnitude (Fig. 21), identifying the dominant scenario that should control the hazard becomes considerably more challenging.

**Fig. 22:**
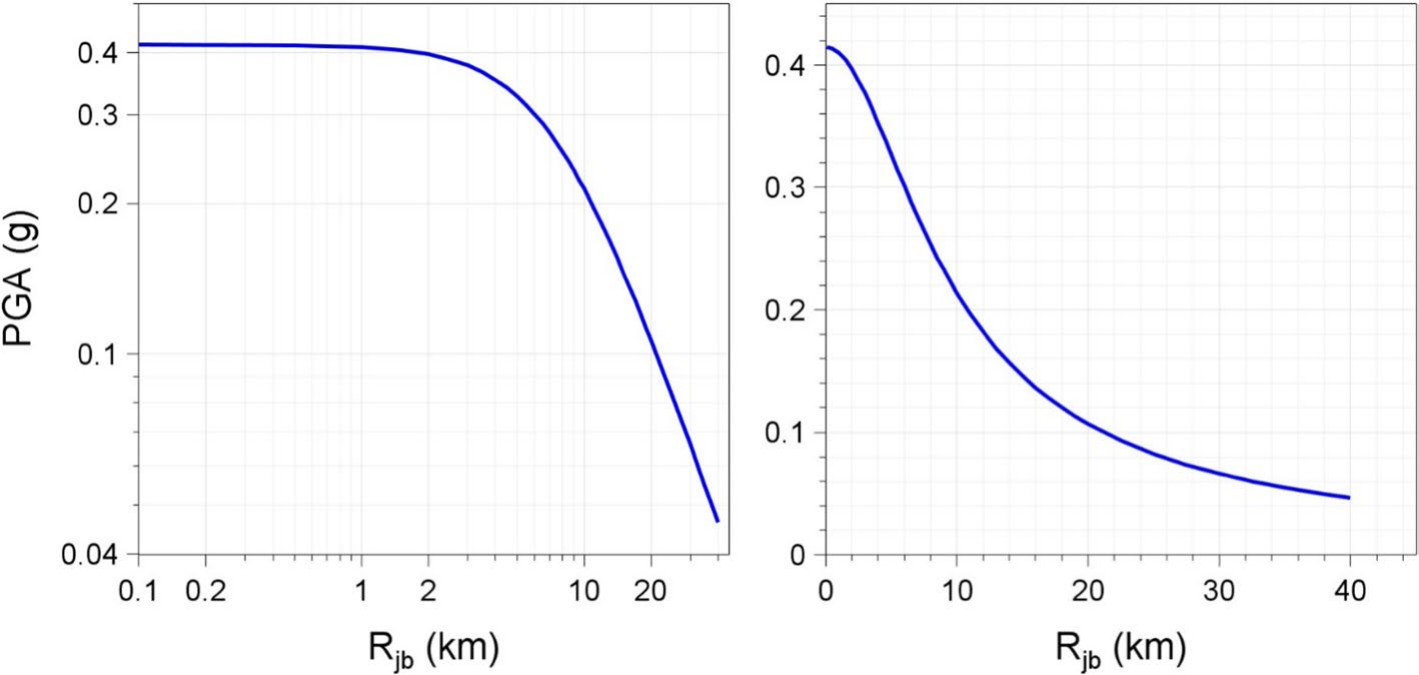
Median PGA values predicted by the European GMM of Akkar et al. (2014) at rock sites (V_S30_ = 760 m/s) plotted against distance for a magnitude **M** 6.5 strike-slip earthquake; both plots show exactly the same information but the left-hand frame uses the conventional logarithmic axes whereas the right-hand frame used linear axes and perhaps conveys more clearly how swiftly the amplitudes decay with distance

An additional complication that arises in practice is that it is usually impossible to assign all observed seismicity to mapped geological faults, even though every seismic event can be assumed to have originated from rupture of a geological fault. This situation arises both because of the inherent uncertainty in the location of earthquake hypocentres and the fact that not all faults are detected, especially smaller ones and those embedded in the crust that do not reach the Earth’s surface. Consequently, some sources of potential future seismicity are modelled simply as areas of ‘floating’ earthquakes that can occur at any location within a defined region. The definition of both the location and the magnitude of the controlling earthquake in DSHA then becomes an additional challenge: if the approach genuinely is intended to define the worst-case scenario, in many cases this will mean that the largest earthquake that could occur in the area would be placed directly below the site, but this is rarely, if ever, done in practice. Instead, the design earthquake is placed at some arbitrarily selected distance (in the US, where DSHA was used to define the design basis for most existing NPPs, this was sometimes referred to as the ‘shortest negotiated distance’), to which the hazard estimate can be very sensitive because of the swift decay of ground motions with distance from the earthquake source (Fig. 22).

**Fig. 23:**
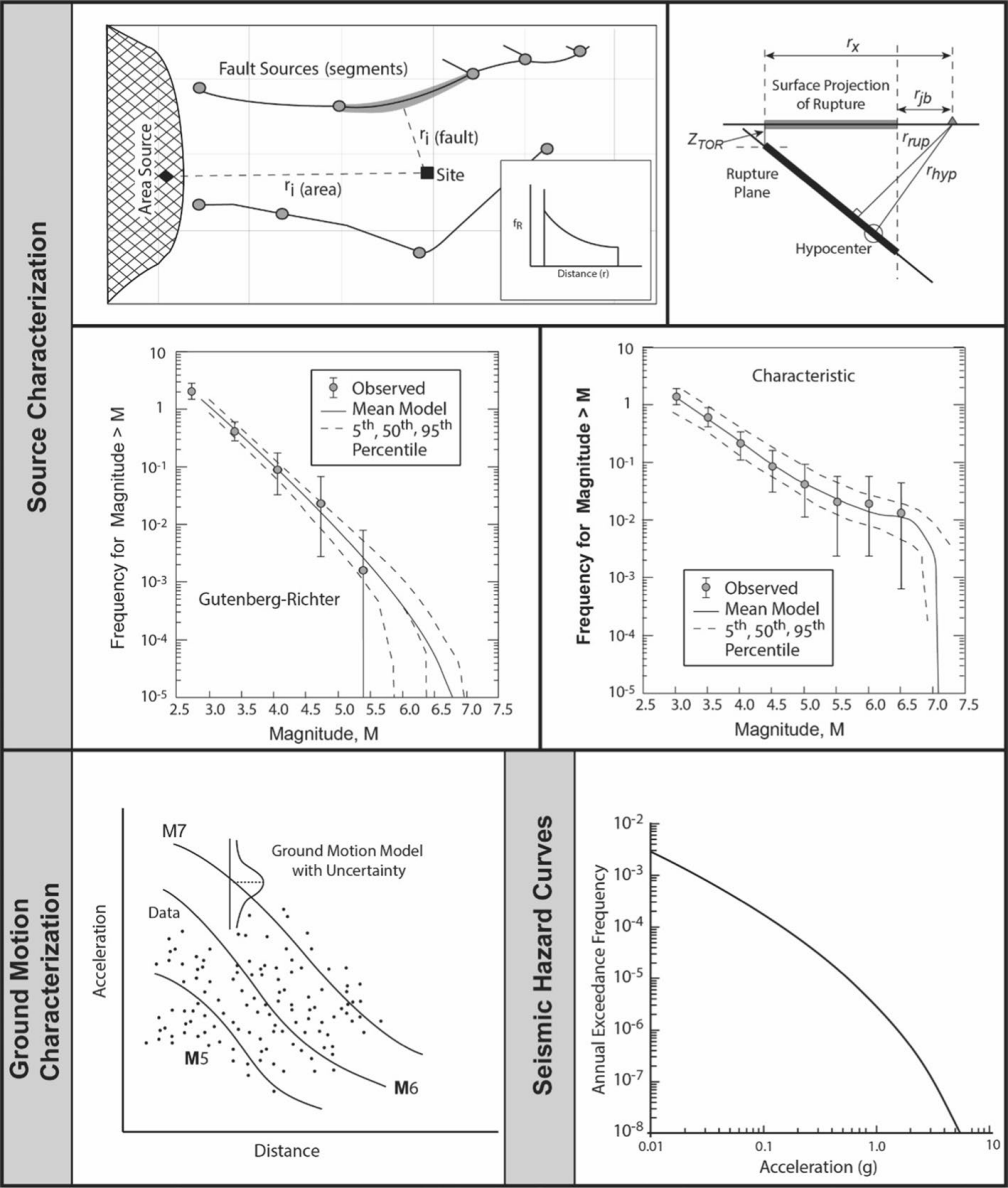
Illustration of the steps involved in a PSHA (adapted from USNRC 2018)

The inspired insight of Allin C. Cornell and Luis Esteva was to propose an approach to seismic hazard analysis, now known as PSHA, that embraced the inherent randomness in the magnitude and location of future earthquakes by treating both M and R as random variables (Esteva 1968; Cornell 1968). The steps involved in executing a PSHA are illustrated schematically in Fig. 23.

A key feature of PSHA is a model for the average rate of earthquakes of different magnitudes, generally adopting the recurrence relationship of Gutenberg and Richter (1944):

3 $$\mathrm{log}\left(N\right)=a-bM$$

where N is the average number of earthquakes of magnitude ≥ M per year, and *a* and *b* are coefficients found using maximum likelihood method (e.g., Weichert 1980); least squares fitting is not appropriate since for a cumulative measure such as N, the data points are not independent. The coefficient *a* is the activity rate and is higher in regions with greater seismicity, whereas *b* reflects the relative proportions of small and large earthquakes (and often, but not always, takes a value close to 1.0). The recurrence relation is truncated at an upper limit, Mmax, which is the largest earthquake considered to be physically possible within the source of interest. The estimation of Mmax is discussed further in Sect. 9.2.

Rather than an abrupt truncation of the recurrence relationship at Mmax, it is common to use a form of the recurrence relationship that produces a gradual transition to the limiting magnitude:

4 $$N\left(M\right)=\nu ({M}_{lower})\left[\frac{{e}^{-\beta (M-{M}_{lower})}-{e}^{-\beta (Mmax-{M}_{lower})}}{1-{e}^{-\beta (Mmax-{M}_{lower})}}\right]$$

where M_lower_ is the lower magnitude limit, $\nu ({M}_{lower})$ is the annual rate of earthquakes with that magnitude, and $\beta =b.\mathrm{ln}(10)$. For faults, it is common to adopt instead a characteristic recurrence model, since it has been observed that large faults tend to generate large earthquakes with an average recurrence rate that is far higher than what would be predicted from extrapolation of the recurrence statistics of smaller earthquakes (e.g., Wesnousky et al. 1983; Schwartz and Coppersmith 1984; Youngs and Coppersmith 1985). Whereas the Gutenberg-Richter recurrence parameters are generally determined from analysis of the earthquake catalogue for a region, the parameterisation of the characteristic model is generally based on geological evidence.

In publications that followed the landmark paper of Cornell (1968), the variability in the GMM was also added as another random variable in PSHA calculations (see McGuire 2008). Consequently, PSHA is an integration over three variables: M, R and $\varepsilon$. Rather than identifying a single scenario to characterise the earthquake hazard, PSHA considers all possible scenarios that could affect the site in question, calculating the consequent rate at which different levels of ground motion would be exceeded at the site of interest as a result. For a given value of the ground-motion parameter of interest (say, PGA = 0.2* g*), earthquakes of all possible magnitudes are considered at all possible locations within the seismic sources, and the value of $\varepsilon$ required to produce a PGA of 0.2* g* at the site is calculated in each case. The annual frequency at which this PGA is produced at the site due to each earthquake is the frequency of events of this magnitude (determined from the recurrence relationship) multiplied by the probability associated with the $\varepsilon$ value (obtained from the standard normal distribution). By assuming that all the earthquake scenarios are independent—for which reason foreshocks and aftershocks are removed from the earthquake catalogue before calculating the recurrence parameters, a process known as de-clustering—the frequencies can be summed to obtain the total frequency of exceedance of 0.2* g*. Repeating the exercise for different values of PGA, a hazard curve can be constructed, as in the lower right-hand side of Fig. 23. The hazard curve allows rational selection of appropriate design levels on the basis of the annual exceedance frequency (or its reciprocal, the return period): return periods used to define the design motions for normal buildings are usually in the range from 475 to 2,475 years, whereas for NPPs the return periods are in the range 10,000 to 100,000 years.

**Fig. 24:**
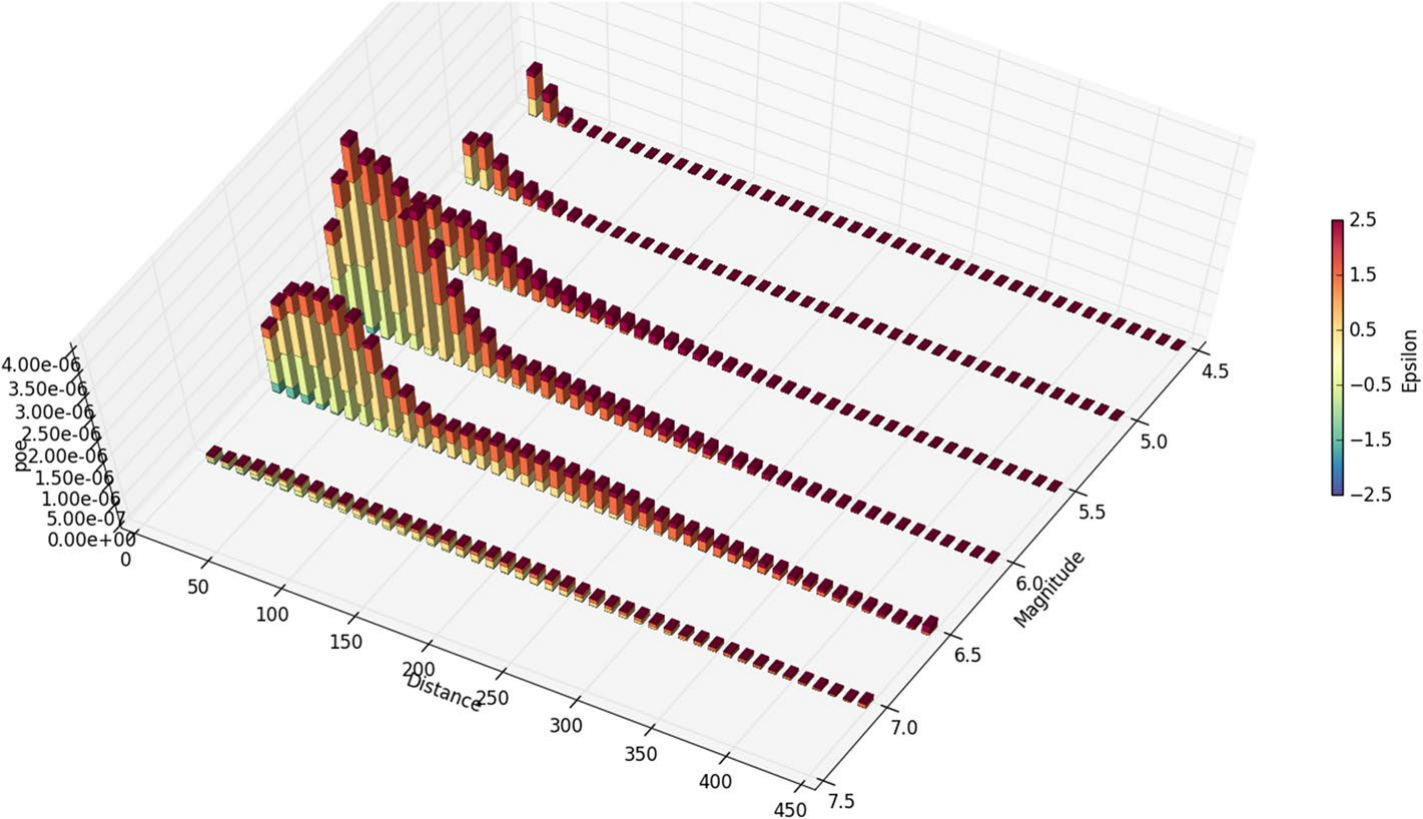
Disaggregation of the hazard in terms of spectral accelerations at 1.0 s for an annual exceedance frequency of 10^–4^ showing the relative contributions of different M-R-$\upvarepsilon$ combinations (Almeida et al. 2019)

Since PSHA calculations are effectively a book-keeping exercise that sums the contributions of multiple M-R-$\varepsilon$ triplets to the site hazard, for a selected annual exceedance frequency the process can be reversed to identify the scenarios that dominate the hazard estimates, a process that is referred to as disaggregation (e.g., McGuire 1995; Bazzurro and Cornell 1999). An example of a hazard disaggregation is shown in Fig. 24; to represent this information in a single scenario, one can use the modal or mean values of the variables, each of which has its own merits and shortcomings (Harmsen and Frankel 2001).

**Fig. 25:**
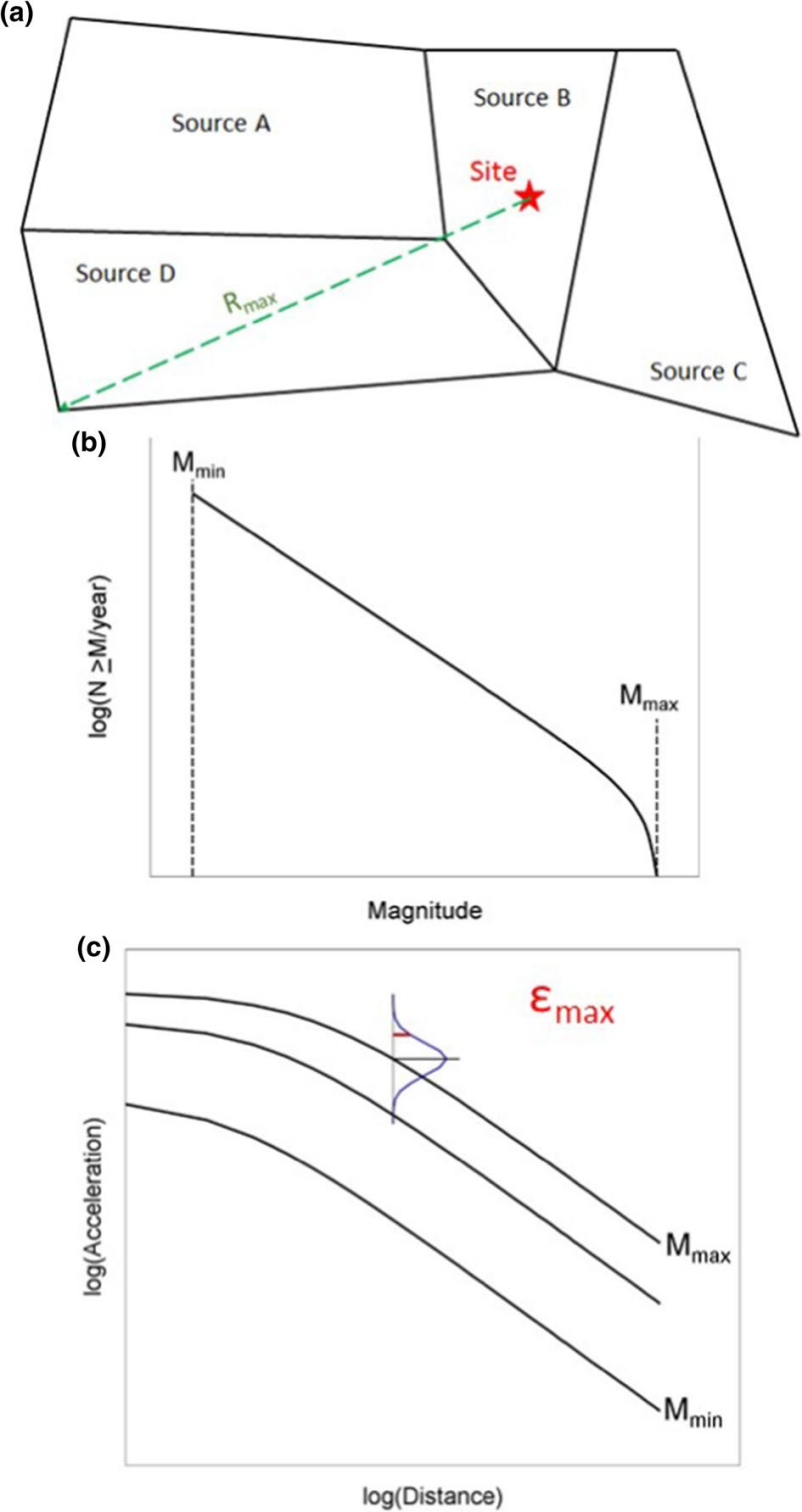
Illustration of integration limits in PSHA in terms of **a** seismic source zones, **b** recurrence relations, and **c** GMMs (Bommer and Crowley 2017)

**Fig. 26:**
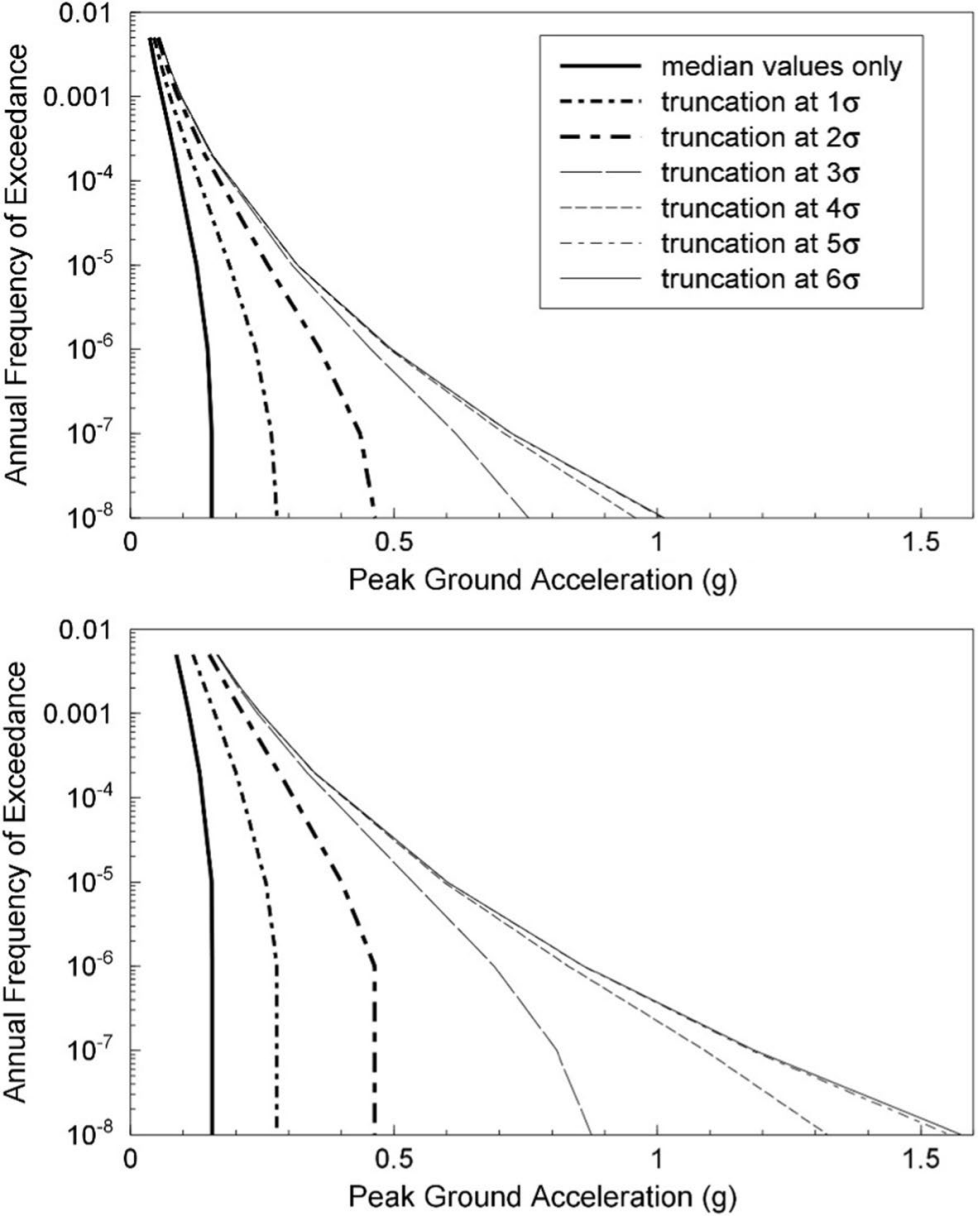
Illustration of the effect of truncating the distribution of ground-motion residuals by imposing different values of ${\upvarepsilon }_{\mathrm{max}}$ in PSHA calculations for regions of low (upper) and high (lower) seismicity rates (Bommer et al. 2004)

Since PSHA is an integration over three random variables, it is necessary to define upper and lower limits on each of these, as indicated in Fig. 25. The upper limit on magnitude has already been discussed; the lower limit on magnitude, M_min_, is discussed in Sect. 3.2. For distance, the minimum value will usually correspond to an earthquake directly below the site (unlike the upper left-hand panel in Fig. 23, the site is nearly always located within a seismic source zone, referred to as the host zone), whereas the upper limit, usually on the order of 200–300 km, is controlled by the farthest sources that contribute materially to the hazard (and can be longer if the site region is relatively quiet and there is a very active seismic source, such as a major fault or a subduction zone, at greater distance). Standard practice is to truncate the residual distribution at a limit such as 3 standard deviations; the lower limit on $\varepsilon$ is unimportant. There is neither a physical nor statistical justification for such a truncation (Strasser et al. 2008) but it will generally only impact on the hazard estimates for very long return periods in regions with high seismicity rates (Fig. 26).

### Seismic risk as the context for PSHA

In my view, seismic hazard assessment cannot—and should not—be separated from considerations of seismic risk. Leaving aside hazard sensitivity calculations undertaken for research purposes, all seismic hazard assessments have a risk goal, whether this is explicitly stated or only implicit in the use of the results. When I have made this point in the past, one counter argument given was that one might conduct a PSHA as part of the design of strong-motion recording network, but in that case I would argue that the ‘risk’ would be installing instruments that yield no or few recordings. To be meaningful, hazard must be linked to risk, either directly in risk analysis or through seismic design to mitigate risk. In the previous section I referred to return periods commonly used as the basis for seismic design, but in themselves these return periods do not determine the risk level; the risk target is also controlled by the performance criteria that the structure should meet under the specified loading condition, such as the ‘no collapse’ criterion generally implicit in seismic design codes as a basis for ensuring life safety. For a NPP, the performance target will be much more demanding, usually related to the first onset of inelastic deformation. In effect, the return period defines the hazard, and the performance targets the fragility, both chosen in accordance with the consequences of failure to meet the performance criterion. For NPPs, the structural strength margins (see Fig. 1) mean that the probability of inelastic deformations will be about an order of magnitude lower than the annual exceedance frequency of the design motions, and additional structural capacity provides another order of magnitude against the release of radiation: design against a 10,000-year ground motion will therefore lead to a 1-in-1,000,000 chance of radiation release.

**Fig. 27:**
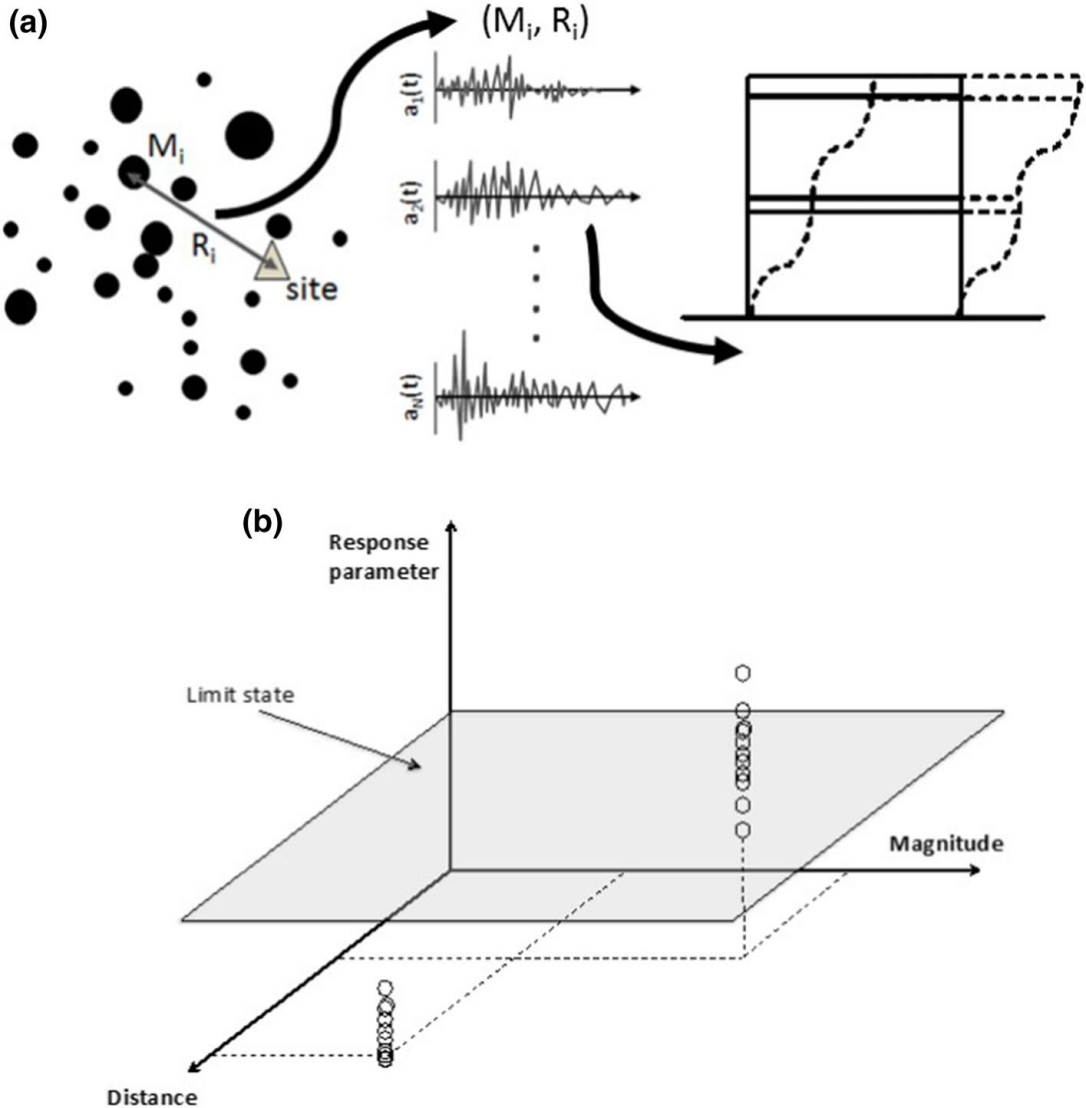
Schematic illustration of rigorous risk assessment for a single structure and a defined response condition or limit state; **a** for each earthquake scenario, a suite of accelerograms is generated and used in dynamic analyses of a structural model, and **b** the results used to determine the rate at which damage occurs (Bommer and Crowley 2017)

One way in which risk considerations are directly linked to PSHA is in the definition of the minimum magnitude, M_min_, considered in the hazard integrations. This is not the same as the smallest magnitude, M_lower_, used in the derivation of the recurrence relation in Eq. (4), but rather it is the smallest earthquake that is considered capable of contributing to the risk (and is therefore application specific). This can be illustrated by considering how seismic risk could be calculated in the most rigorous way possible, for a single structure. For every possible earthquake scenario (defined by its magnitude and location), a suite of acceleration time-histories could be generated or selected from a very large database; collectively, the time-histories would sample the range of possible ground motions for such a scenario in terms of amplitude, frequency content, and duration or number of cycles. Non-linear structural analyses would then be performed using all these records, and the procedure repeated for all possible scenarios. For a given risk metric, such as a specified level of damage, the rate can be determined by the proportion of analyses leading to structural damage above the defined threshold, which can then be combined with the recurrence rate of the earthquake scenarios to estimate annual rates of exceeding the specified damage level (Fig. 27).

**Fig. 28:**
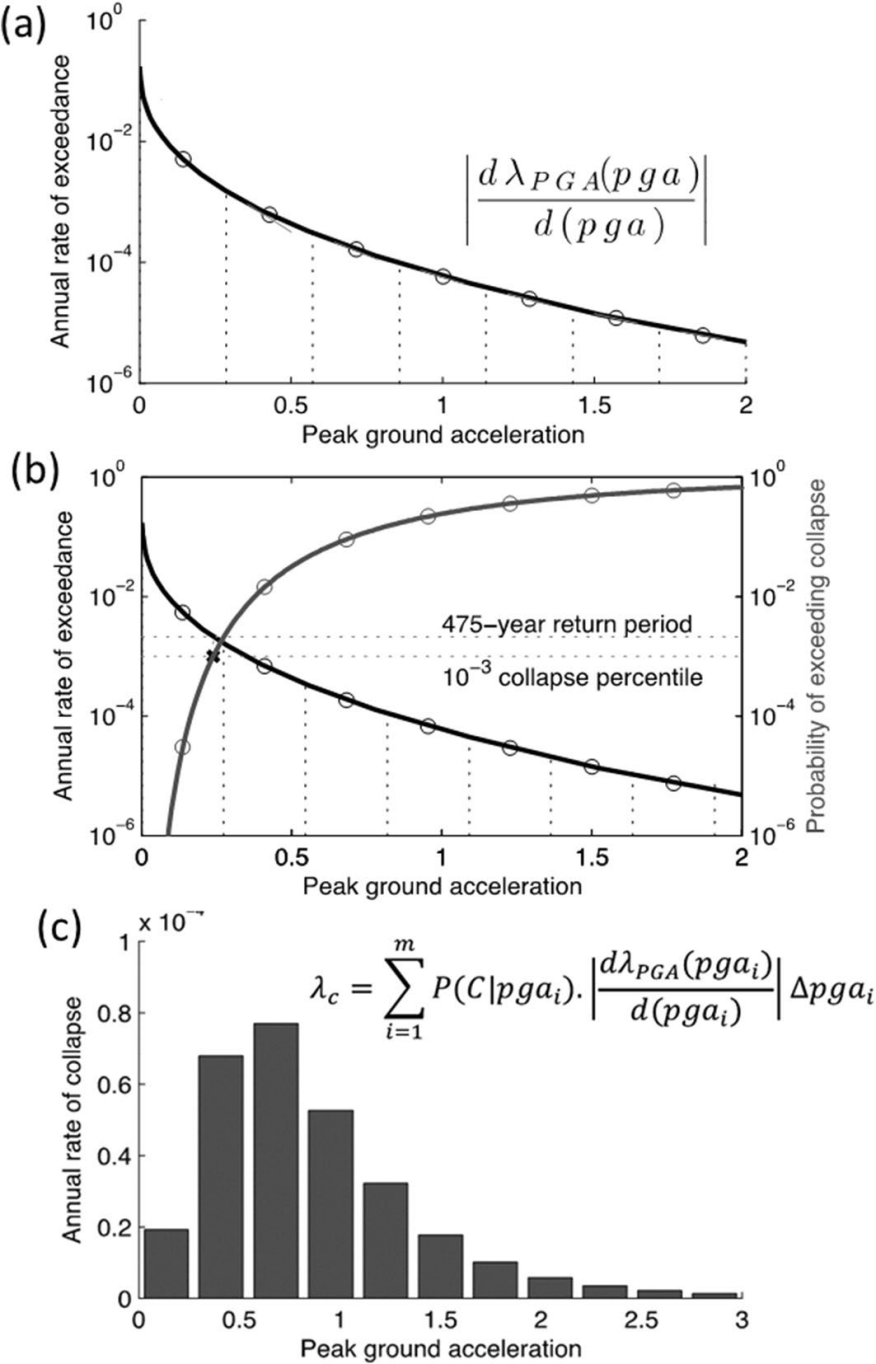
Illustration of seismic risk assessment starting with **a** a seismic hazard curve in terms of PGA and then **b** combining this hazard curve with a fragility function so that **c** the convolution of the two yields the total probability of collapse (Bommer and Crowley 2017)

For any given structure, there will be a magnitude level below which the ground motions never cause damage, regardless of their distance from the site. The usual interpretation of such a result is that the short-duration motions from these smaller earthquakes lack the required energy to cause damage. Now, in practice, such an approach to seismic risk analysis would be prohibitively intensive in terms of computational demand, for which reason several simplifications are made. Firstly, the earthquake scenarios and resulting acceleration time-histories are represented by the results of hazard analyses, and secondly the dynamic analyses are summarised in a fragility function. Usually, the hazard is expressed in terms of a single ground-motion parameter that is found to be sufficient to act as an indicator of the structural response; it is also possible, however, to define the fragility in terms of a vector of ground-motion parameters (e.g., Gehl et al. 2013). In a Monte Carlo approach to risk assessment, individual earthquake scenarios are still generated, but for each one the chosen ground-motion parameter is estimated rather than generating suites of accelerograms. If the hazard is expressed in terms of a simple hazard curve, the risk can be obtained by direct convolution of the hazard and fragility curves (Fig. 28). However, in this simplified approach it is necessary to avoid inflation of the risk through inclusion of hazard contributions from the small-magnitude events that are effectively screened out in the more rigorous approach. This is the purpose of the lower magnitude limit, M_min_, imposed on the hazard integral, although there has been a great deal of confusion regarding the purpose and intent of this parameter (Bommer and Crowley 2017). In an attempt to address these misunderstandings, Bommer and Crowley (2017) proposed the following definition: “*M*_*min*_* is the lower limit of integration over earthquake magnitudes such that using a smaller value would not alter the estimated risk to the exposure under consideration*.” The imposition of M_min_ can modify the hazard—in fact, if it did not, it would be pointless—but it should not change the intended risk quantification. For NPP, typical values for M_min_ are on the order of 5.0 (e.g., McCann and Reed 1990).

The key point being made here is that M_min_ is really intended to filter out motions that are insufficiently energetic to be damaging, so it could also be defined as vector of magnitude and distance (the magnitude threshold increasing with distance from the site), or in terms of a ground-motion parameter. This has been done through the use of a CAV (cumulative absolute velocity, which is the integral of the absolute acceleration values over time) filter, which prevents ground motions of low energy from contributing to the hazard estimate. The original purpose of CAV was to inform decision-making following safe shutdown of NPPs and re-start following earthquake shaking (EPRI 1988). However, CAV filters have been proposed as an alternative to M_min_ (EPRI 2006a; Watson-Lamprey and Abrahamson 2007) and these have prompted the development of new GMMs for the conditional prediction of CAV (Campbell and Bozorgnia 2010). Other ground-motion parameters or vectors of parameters might serve the same purpose equally well. In practice, different parameters may perform better in different applications, depending on which measures of ground-motion intensity are found to be most efficient for defining the fragility functions of the exposure elements for which risk is directly or indirectly being assessed or mitigated.

The parameter M_min_ is a very clear indicator of the risk relevance of PSHA, but other hazard inputs should also be defined cognisant of the intended risk application, starting with the ground-motion parameters used to quantify the shaking hazard. This includes the subtle issue of how the horizonal component of motion is defined from the two recorded components of each accelerogram. Early GMMs tended to use the larger of the two components but there has subsequently been a trend towards using the geometric mean of the parameters from each horizontal component and numerous variations of this convention, all of which seek to approximate a randomly oriented component (Boore et al. 2006; Watson-Lamprey and Boore 2007; Boore 2010). There is no basis to identify an optimal or most appropriate definition, but it is very important that the component definition employed in the hazard analysis is consistent with the way the horizontal earthquake loading is applied in the structural analyses related to the risk mitigation or analysis. For example, if the geometric mean component is adopted in the hazard analysis but a single, arbitrarily selected horizontal component of the accelerograms is used to derive the fragility functions, then there is an inconsistency that requires accommodation of the additional component-to-component variability (Baker and Cornell 2006). For an interesting discussion of the consistency between horizontal component definitions used in GMMs and hazard analysis, load application in structural analysis, and risk goals of seismic design, see Stewart et al. (2011).

The issue of deterministic vs probabilistic approaches can also arise in the context of risk assessment. A purely deterministic quantification of potential earthquake impacts that gives no indication of the likelihood of such outcomes is of very limited value since it does not provide any basis for comparison with other risks or evaluation against safety standards. In this sense, the context of risk provides strong motivation for adopting probabilistic approaches to seismic hazard assessment. Here it is useful to consider what are the key features that distinguish PSHA and DSHA. The first is that PSHA explicitly includes consideration of earthquake rates and the frequency or probability of the resulting ground motions, whereas DSHA generally ignores the former and only accommodates the latter implicitly. Another important difference is that PSHA considers all possible earthquake scenarios (that could contribute to the risk) whereas DSHA considers only a single scenario. Estimation of the total risk to a structure or portfolio of buildings clearly needs to consider all potential sources of earthquake-induced damage, and informed decisions regarding the mitigation or transfer of the risk clearly require information regarding the probability of different levels of loss. There are situations, however, in which the estimation of risk due to a single specified earthquake scenario can be very useful, including for emergency planning purposes, and for non-specialists understanding risk estimates for a single scenario can be much more accessible than a complete probabilistic risk assessment. A risk assessment for a single scenario does not need to be fully deterministic: the scenario can be selected from disaggregation of PSHA and even if it is selected on another basis, its recurrence interval can be estimated from the relevant recurrence relationship. Furthermore, the variability in the predictions of ground shaking levels can be fully accounted for through the generation of multiple ground-motion fields, sampling from the between-event variability once for each realisation and from the within-event variability for each location. The sampling from the within-event variability can also account for spatial correlation (e.g., Jayaram and Baker 2009) which creates pockets of higher and lower ground motions that influence the resulting hazard estimates when they coincide with clusters of exposure (e.g., Crowley et al. 2008)).

### Uncertainty in Seismic Hazard and Risk Assessments

**Fig. 29:**
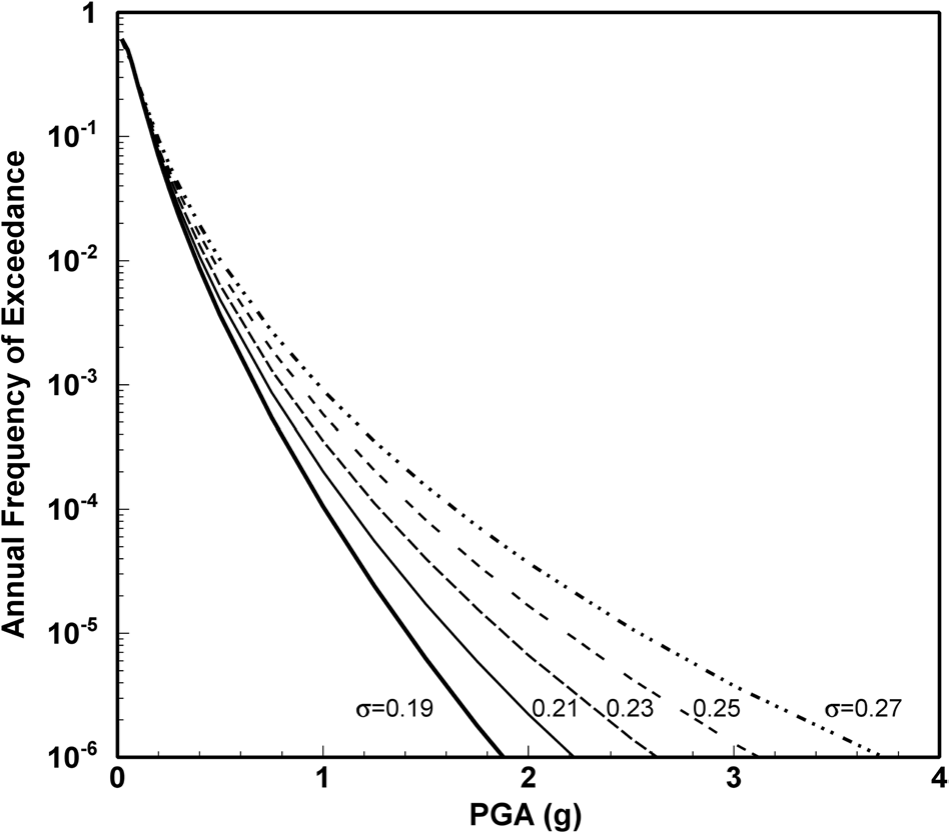
Sensitivity of seismic hazard curves to the standard deviation of the residuals in the GMM (Bommer and Abrahamson 2006)

The basic premise of PSHA is to take into account the apparently random nature of earthquake occurrence and ground-motion generation by integrating over the random variables of M, R and $\varepsilon$ (as a minimum: other random variables can include focal depth distributions and styles-of-faulting, for example). The consequence of the random variability is to influence the shape of the seismic hazard curve, which can be clearly illustrated by looking at the impact of different values of the GMM variability $\sigma$ (Fig. 29).

**Fig. 30:**
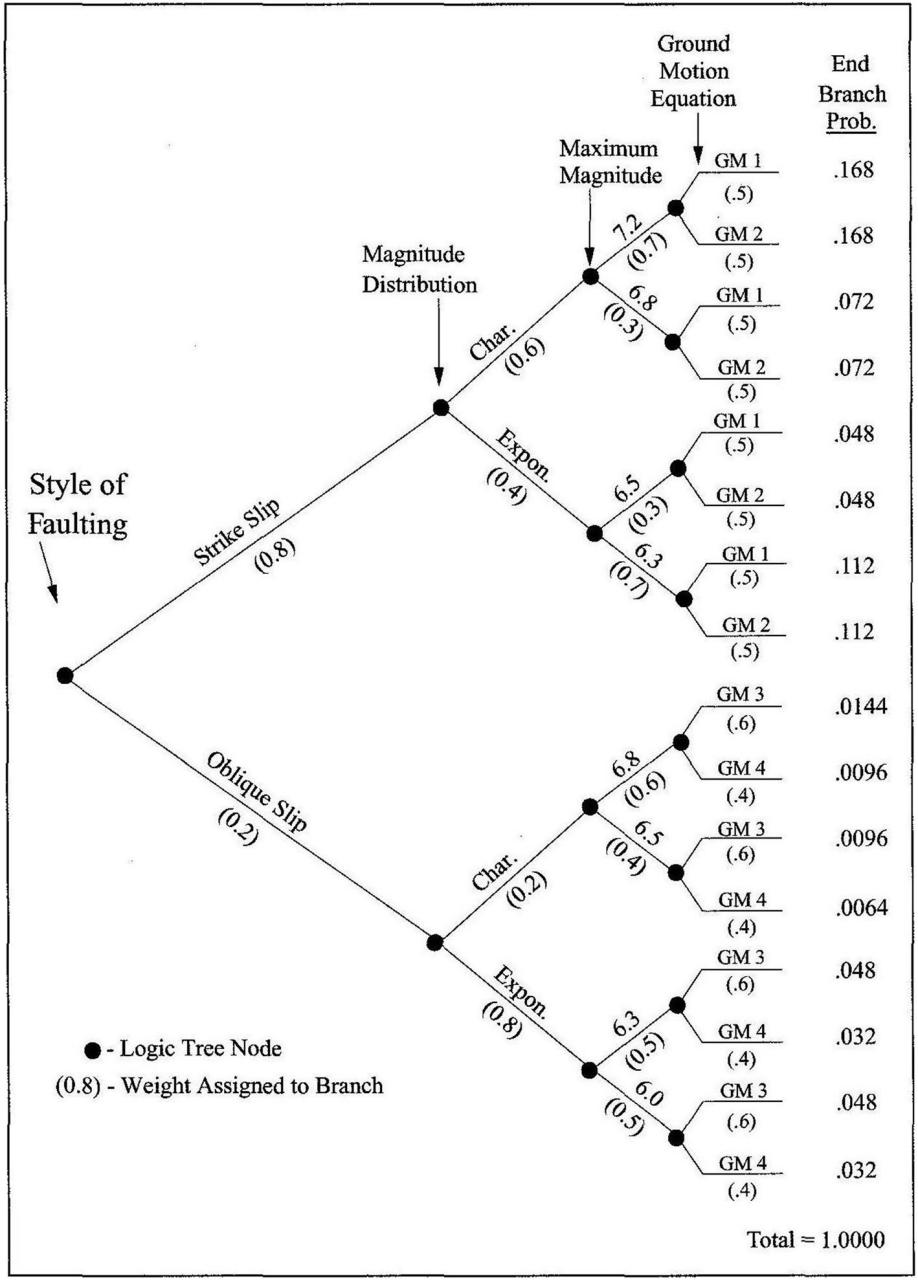
Example of a fault logic tree for PSHA (McGuire 2004)

In defining the seismic source characterisation (SSC) and ground motion characterisation (GMC) models that define the inputs to PSHA, decisions have to be made regarding models and parameter values for which a single ‘correct’ choice is almost never unambiguously defined. The nature of the available data in terms of geological information regarding seismogenic faults, the earthquake catalogue for the region, and strong-motion recordings from the area, is such that it will never cover all of the scenarios that need to be considered in the hazard integrations, so there is inevitably extrapolation beyond the data. Moreover, different experts are likely to derive distinct models from the same data, each reflecting valid but divergent interpretations. Consequently, there is uncertainty in most elements of a PSHA model including the seismic source boundaries, the temporal completeness of the catalogue (which in turn influences the calculated recurrence rates), the value of Mmax, and the choice of GMM. These are all examples of epistemic uncertainty, as introduced in Sect. 1.2. Aleatory variabilities are characterised by distributions based on observational data, and they are then incorporated directly into the hazard integrations, influencing, as shown above, the shape of the hazard curve. Epistemic uncertainties are incorporated into PSHA through the use of logic trees, which were first introduced by Kulkarni et al. (1984) and Coppersmith and Youngs (1986) and have now become a key element of PSHA practice. For each element of the PSHA input models for which there is epistemic uncertainty, a node is established on the logic tree from which branches emerge that carry alternative models or alternative parameter values. Each branch is assigned a weight that reflects the relative degree of belief in that particular model or parameter value as being the most appropriate; the weights on the branches at each node must sum to 1.0 (Fig. 30).

**Fig. 31:**
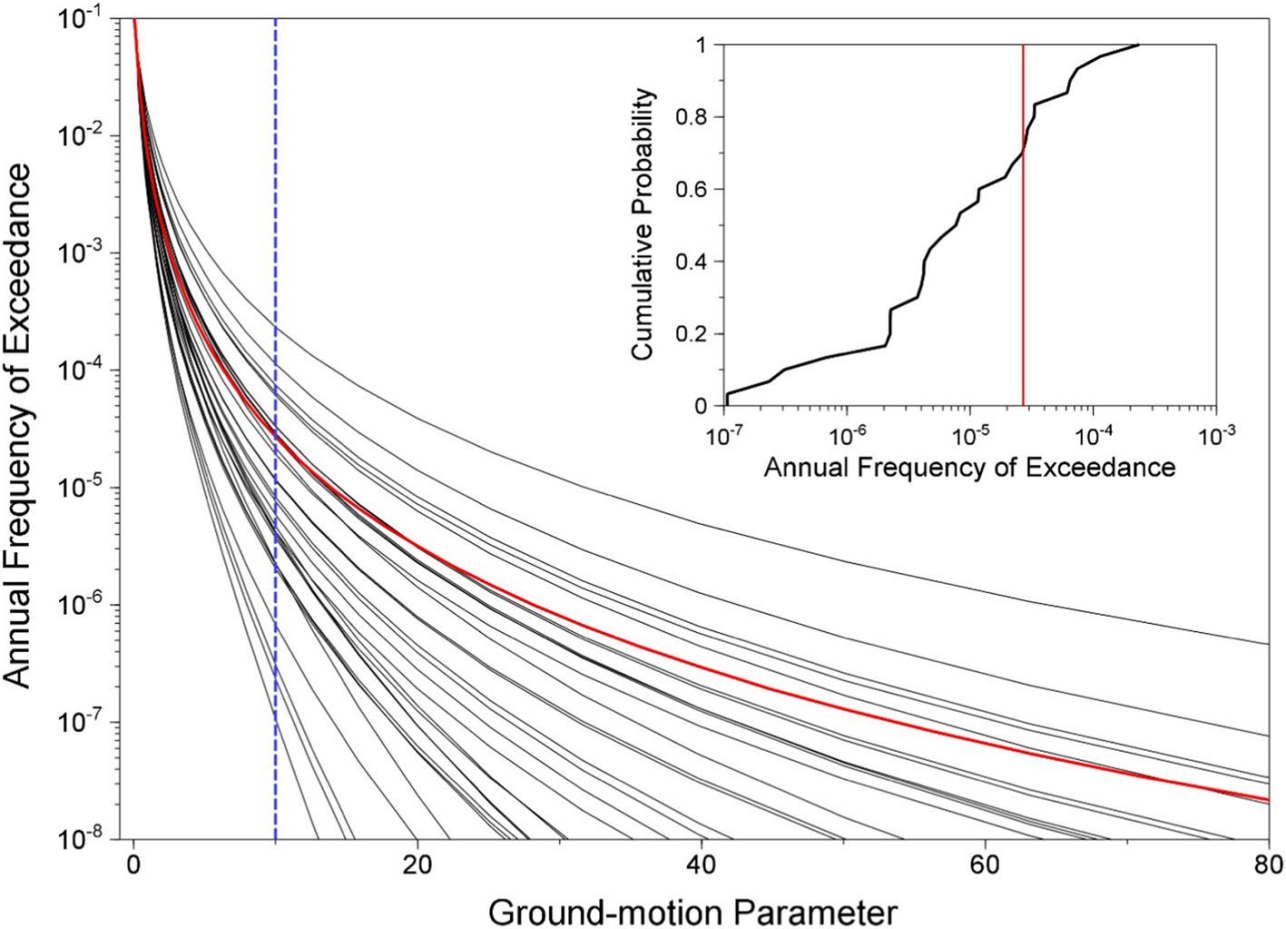
In the main plot the grey lines are hazard curves corresponding to different branch combinations from a logic tree and the red curve is the mean hazard; the inset figure shows the cumulative weights associated with the AFEs for a specific ground-motion level, indicated by blue dashed line in main plot

The logic tree in Fig. 30 has just four nodes and two branches on each node, which is much simpler than most logic trees used in practice but serves to illustrate the basic concept. The PSHA calculations are repeated for every possible path through the logic tree, each combination of branches yielding a seismic hazard curve; the total weight associated with each hazard curve is the product on the weights on the individual branches. The logic-tree in Fig. 30 would result in a total of 16 separate hazard curves, which would be associated with the weights indicated on the right-hand side of the diagram. Whereas aleatory variability determines the shape of the hazard curve, the inclusion of epistemic uncertainty leads to multiple hazard curves. The output from a PSHA performed within a logic-tree framework is used to summarise the statistics of the hazard—the annual frequency of exceedance or AFE—for each ground motion level, calculating the mean AFE (Fig. 31). For seismic design rather than risk analysis, it could be argued that since the starting point is the selected AFE, the mean ground-motion amplitude at each AFE should be determined instead (Bommer and Scherbaum 2008). Such an approach would yield appreciably different results, but this is not standard practice, and the mean hazard curve should be calculated as illustrated in Fig. 31.

**Fig. 32:**
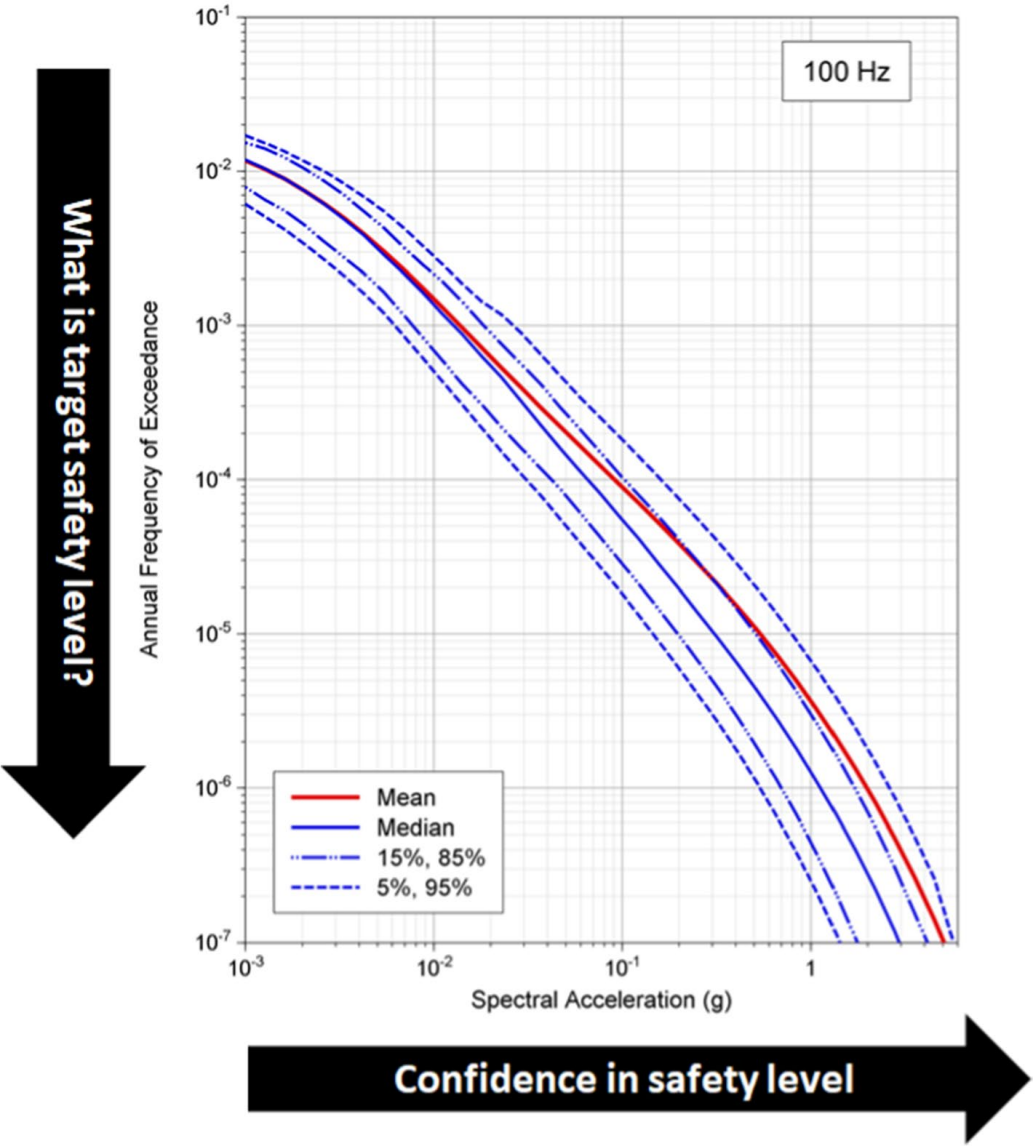
Decision-making for seismic safety using a distribution of site-specific hazard estimates; hazard curves from Almeida et al. (2019)

As well as the mean hazard, it is possible to calculate the median and other fractiles of the hazard. The output from a PSHA thus moves from a single hazard curve to a distribution of hazard curves, allowing two choices to be addressed: the level of motion corresponding to the target safety level (which is determined by the AFE and the associated performance targets, as explained in the previous section) and the confidence level required that this safety level is achieved (Fig. 32). The second decision can be stated in terms of the following question: in light of the unavoidable uncertainty associated with the estimation of the seismic hazard, what degree of confidence is required that the hazard assessment has captured the hazard levels? This is a critical question, and it is the reason that capturing the epistemic uncertainty is one of the most important features of seismic hazard analysis.

A distribution of hazard curves such as shown in Fig. 32 conveys the overall level of epistemic uncertainty in the hazard estimates, both from the spread of the fractiles and also from the separation of the median and mean hazard curves. In practice, the most commonly used output is the mean hazard curve. Just as there is epistemic uncertainty in hazard assessment, there is also epistemic uncertainty in most of the other elements of risk analysis (e.g., Crowley et al. 2005; Kalakonas et al. 2020). Fully probabilistic risk analysis, as applied for example to NPPs, considers the full distribution of both hazard and fragility curves, but the mean risk can be obtained by simply convolving the mean hazard with the mean fragility.

A key challenge in PSHA, and in seismic risk analysis, is the separation and quantification of aleatory variability and epistemic uncertainty; Sect. 5 is focused on this challenge in conducting PSHA. The distinction between variability and uncertainty is not always very clear and some have argued that the distinction is unimportant (e.g., Veneziano et al. 2009). If the only required output is the mean hazard, then whether uncertainties are treated as random or epistemic is immaterial, provided that all uncertainties are neither excluded nor double counted. However, if the fractiles are required, then the distinction does become important. In the UK, for example, the expectation of the Office for Nuclear Regulation is that the seismic hazard at NPP sites will be characterised by the motions with an 84-percentile AFE of 10^–4^; if epistemic uncertainties are treated as aleatory variabilities, this quantity will likely be underestimated.

## Good practice in PSHA

The rational management of seismic risk necessarily begins with broad acceptance amongst relevant stakeholders of robust estimates of the seismic hazard. In this section, I briefly summarise what I would suggest are the minimum requirements that a site-specific PSHA should fulfil to increase the chances of the results being accepted.

In an overview of the state of practice two decades ago, Abrahamson (2000) stated that “*The actual practice of seismic hazard analysis varies tremendously from poor to very good*.” I agree that variation in practice is very large and would even suggest that even stronger adjectives might apply to the end members. I would propose that the best practice, usually exemplified in large projects for nuclear sites, is excellent, and moreover that it frequently defines the state of the art. At the lower end, the practice can indeed be very poor although there are reasons to be optimistic about the situation improving, especially with the comprehensive and clear guidance that is now becoming available in the textbook by Baker et al. (2021) referred to previously. International ventures like GSHAP (Global Seismic Hazard Assessment Project; Giardini 1999; Danciu and Giardini 2015) and GEM (Global Earthquake Model; Crowley et al. 2013; Pagani et al. 2015; Pagani et al. 2020) have done a fantastic job in promoting good practice PSHA practice around the world, especially in developing countries. Much of the poor practice that persists is related to studies conducted for engineering projects that are conducted on compressed schedules and with very small budgets, and which are of questionable value.

In Sect. 4.1, I highlight some of the common errors that are observed in practice and which could be easily eliminated. The following sections then present features of PSHA studies that I believe enhance hazard assessments.

### Internal consistency

The objective in conducting a PSHA should be to achieve acceptance of the outcome by all stakeholders, including regulators. If the study makes fundamental errors, then all confidence in the results is undermined and the assessment can be easily dismissed. I am assuming here that the PSHA calculations are at least performed correctly in terms of integration over the full ranges of M, R and $\varepsilon$; there have been cases of studies, for example, that fix $\varepsilon$ to a constant value (such as zero, thus treating the GMM as a deterministic prediction, or 1), which simply does not constitute PSHA.

The major pitfalls, in my view, are related to performing hazard calculations that are not internally consistent. In Sect. 3.2, I already discussed the importance of consistency between the hazard study and the downstream structural analyses or risk calculations, but there are also issues of consistency within the PSHA. Firstly, there needs to be consistency between the SSC and GMC models, with the latter explicitly considering and accommodating the full range of independent variables defined in the former and* vice versa*. Consistent definitions of independent variables are also important. For example, if the magnitude scale adopted in the homogenised earthquake catalogue used to derive the recurrence parameters is different from the scale used in the GMMs, an adjustment is required. The easiest option is to use an appropriate empirical relationship between the two magnitude scales to transform the GMM to the same scale as the earthquake catalogue, but it is important to also propagate the variability in the magnitude conversion into the sigma value of the GMM (e.g., Bommer et al. 2005). Fortunately, these days such conversions are not often required because most GMMs and most earthquake catalogues are expressed in terms of moment magnitude, **M** (or M_w_).

**Fig. 33:**
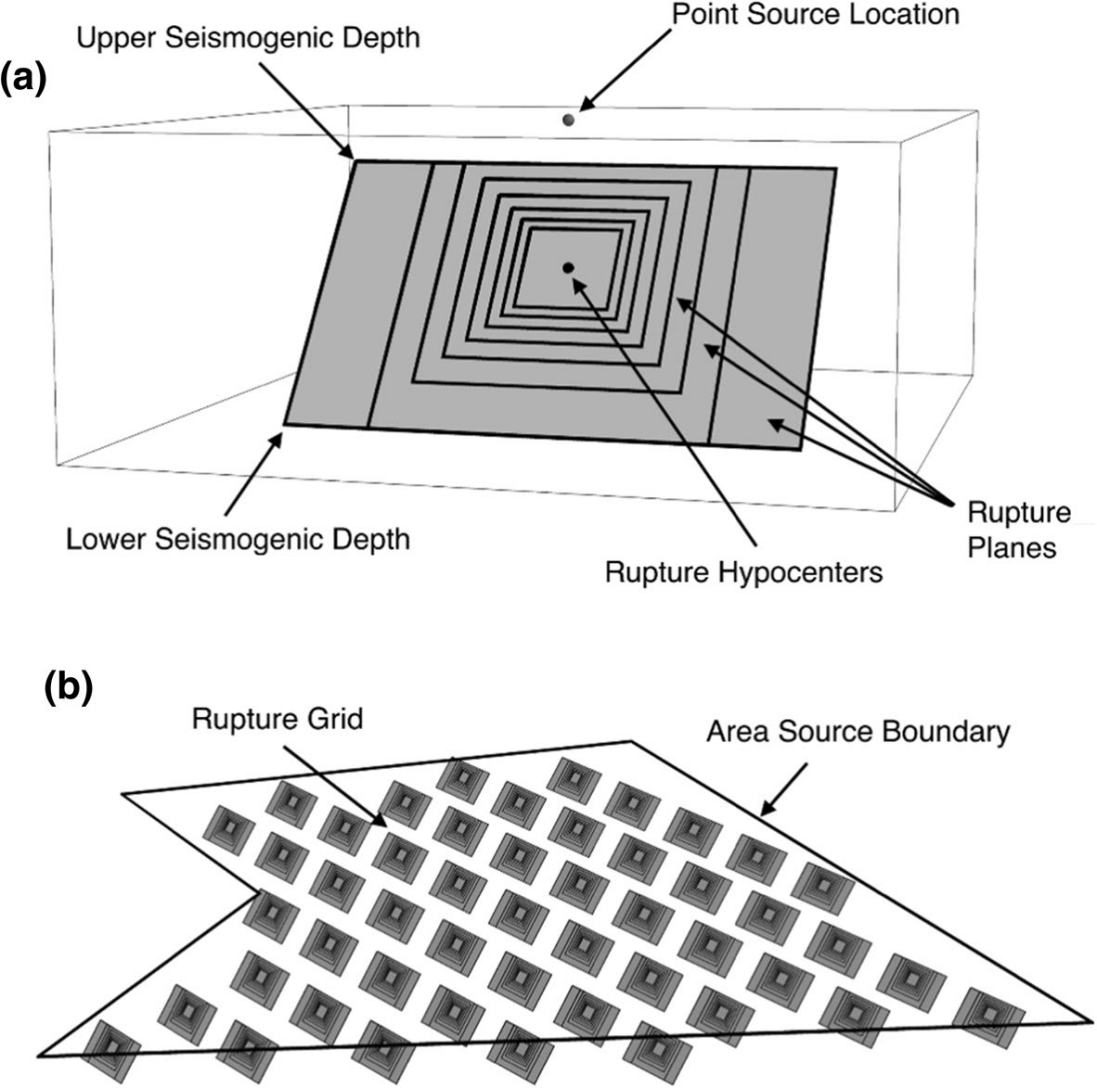
**a** Illustration of virtual ruptures for earthquake of different magnitudes for a single point source; **b** virtual ruptures generated within a source zone, which in practice could also have different orientations, dips and depths (Monelli et al. 2014)

Another important issue of consistency arises for SSC models that include area source zones because most modern GMMs used distance metrics such as R_rup_ or R_jb_ that are defined relative to extended fault ruptures. The easiest way to integrate over a seismic source zone is to discretise the area into small elements, effectively defining the distance to the site as R_epi_ or R_hyp_, which then creates an inconsistency with the distance metric used in the GMMs. Some freely available software packages for performing PSHA integrate over areal sources in this way, leading to consistent underestimation of the hazard when deployed with GMMs using R_rup_ or R_jb_ (Bommer and Akkar 2012). In this case, converting the GMM from a finite rupture distance metric to a point-source metric is not advisable since the variability associated with such conversions is very large (e.g., Scherbaum et al. 2004a), although it should also vary with both magnitude and distance (e.g., Thompson and Worden 2018). The approach generally used is to generate virtual fault ruptures within the source zone, the dimensions of which are consistent with the magnitude of each scenario (Monelli et al. 2014; Campbell and Gupta 2018; Fig. 33). The availability of PSHA software packages such as OpenQuake (Pagani et al. 2014) with the facility to generate such virtual ruptures facilitates avoidance of this incompatibility in hazard calculations. The specification of the geometry and orientation of the virtual ruptures creates considerable additional work in the construction of the SSC model and the generation of the ruptures also adds a computational burden to the calculations. Bommer and Montaldo-Falero (2020) demonstrated that for source zones that are somewhat remote from the site, it is an acceptable approximation to simply use point-source representations of the earthquake scenarios.

Within the GMC model, a potential inconsistency can arise if multiple GMMs are used with different definitions of the horizontal component of motion. Several studies have presented empirically derived conversions between different pairs of definitions (e.g., Beyer and Bommer 2006; Shahi and Baker 2014; Bradley and Baker 2015; Boore and Kishida 2017), making it relatively easy to adjust all the GMMs to a common definition. However, since some of these conversions apply both to the medians and the sigma values, they should be applied prior to the hazard calculations rather than as a post-processing adjustment.

When site effects are modelled separately from the ground-motion prediction—which should always be the case for site-specific PSHA—then important challenges arise to ensure compatibility between the prediction of motions in rock and the modelling of site response. These issues are discussed in detail in Sect. 5.3.

### Inclusion of epistemic uncertainty

Epistemic uncertainties in PSHA are unavoidable and frequently quite large. Consequently, it is indispensable that they should be identified, quantified, and incorporated into the hazard analysis. For any PSHA to be considered robust and reliable, it must have taken account of the uncertainties in the SSC and GMC models. Beyond performing hazard calculations that are mathematically correct and internally consistent, this is probably the single most important feature in determining whether or not a hazard assessment is considered acceptable or not.

Every PSHA should therefore make a concerted effort to properly characterise and incorporate epistemic uncertainties. This is of paramount importance and is the reason that all PSHA studies now include a logic tree *de rigueur*. However, simply including a logic tree for the key inputs to the hazard calculations does not guarantee an appropriate representation of the epistemic uncertainty, although this may not always be immediately obvious. Reflecting the primordial importance of this issue, the next two complete sections of the paper are devoted to the identification and quantification of epistemic uncertainty in PSHA: Sect. 5 discusses technical aspects of ensuring that epistemic uncertainty is adequately captured in the hazard input models; Sect. 6 discusses procedural guidelines that have been developed specifically for this process.

Before discussing the technical and procedural frameworks for capturing uncertainty in PSHA, it is important to emphasise that this is not the only goal of a successful PSHA. Equally important objectives are to build the best possible SSC and GMC models—which could be interpreted as the best constrained models—and also to reduce as much as possible the associated uncertainty through the compilation of existing data and collection of new data from the site and region. The task then remains to ensure adequate representation of the remaining epistemic uncertainty that cannot be reduced or eliminated during the course of the project, but the construction of the logic tree should never be a substitute for gathering data to constrain the input models.

### Peer review and quality assurance

Appropriately conducted peer review and quality assurance (QA) can both contribute significantly to the likelihood of a PSHA study being accepted as the basis for decision making regarding risk mitigation measures, by increasing confidence in the execution of the hazard assessment and in the reliability of the results. Peer review and QA are discussed together in this section because the two processes are complementary.

Peer review consists of one or more suitably qualified and experienced individuals providing impartial feedback and technical challenge to the team conducting the hazard assessment. While it can be viewed as a relatively easy task (compared to building the hazard input models and performing the PSHA calculations), effective peer review requires considerable discipline since the reviewers must be impartial and remain detached from the model building. The focus of the peer review must always be on whether the team conducting the study has considered all of the available information and models (and the peer reviewers can and should bring to their attention any important information that has been overlooked) and the technical justifications given for all of the decisions made to develop the models, including the weights on the logic-tree branches. The peer review should interrogate and, when necessary, challenge the work undertaken, without falling into the trap of prescribing what should be done or pushing the modelling teams into building the models the peer reviewer would have constructed if they had been conducting the study. If this degree of detachment is achieved, then the peer review process can bring great value in providing an impartial and independent perspective for the teams that are fully immersed in the processes of data interpretation and model development.

Late-stage peer review, in which the first genuine engagement of the reviewers is to review a draft report on the PSHA, is largely pointless. At that stage, it is very unlikely that the model building and hazard calculations will be repeated in the case that the peer review identifies flaws, in which case the outcome is either unresolved objections from the peer reviewers or rubber stamping of an inadequate study. Peer reviewers should be engaged from the very outset and be given the opportunity to provide feedback at all stages of the work, including the database assembly and the model building process from the conceptual phase to finalisation. The hazard calculations should only begin after all issues raised by the peer review have been resolved. If the peer review process is managed intelligently, the review of the draft final PSHA report should be focused exclusively on presentation and not on any technical details of the SSC and GMC models.

For peer review to enhance the likelihood of acceptance of a PSHA study, a number of factors are worth considering. The first is the selection of the peer reviewers, since the confidence the review adds will obviously be enhanced if those assigned to this role are clearly recognised experts in the field with demonstrable and extensive experience. Secondly, it is of great value to include as part of the project documentation a written record of the main review comments and how they were resolved. Inclusion of a final closing letter from the peer reviewers giving overall endorsement of the study—if that is indeed their consensus view—is a useful way to convey to regulators and other stakeholders the successful conclusion of the peer review process.

The value of the peer review process, both in terms of technical feedback to the team undertaking the PSHA and in terms of providing assurance, can be further enhanced when the study includes formal working meetings or workshops that the reviewers can attend as observers, especially if regulators and other stakeholders are also present to observe the process. This is discussed further in Sect. 6.

**Fig. 34:**
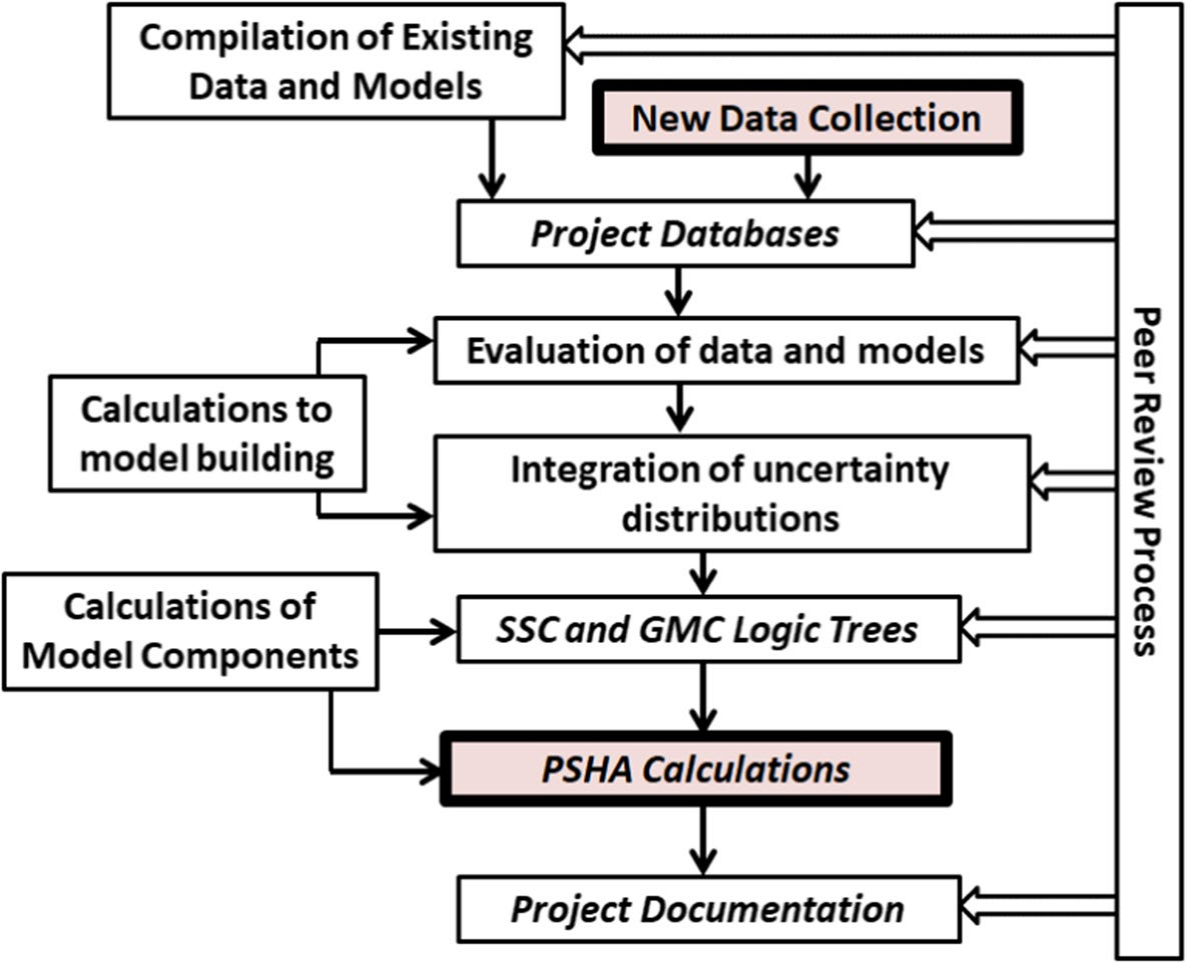
Schematic illustration of the complementary roles of peer review and QA in PSHA projects; the highlighted boxes representing the two stages of the process where formal QA requirements are appropriate; adapted from Bommer et al. (2013) and USNRC (2018)

Quality assurance essentially adds value to a PSHA study by increasing confidence in the numerical values of the final hazard estimates. At the same time, it is important not to impose formal QA requirements on every single step of the project, since this can place an unnecessary and unhelpful burden on the technical teams. Excessive QA requirements will tend to discourage exploratory and sensitivity analyses being performed to inform the model development process, which would be very detrimental. Figure 34 schematically illustrates the complementary nature of QA and peer review, emphasising that while all calculations should be checked and reviewed, formal QA should only be required on new data collection and on the final hazard calculations.

**Fig. 35:**
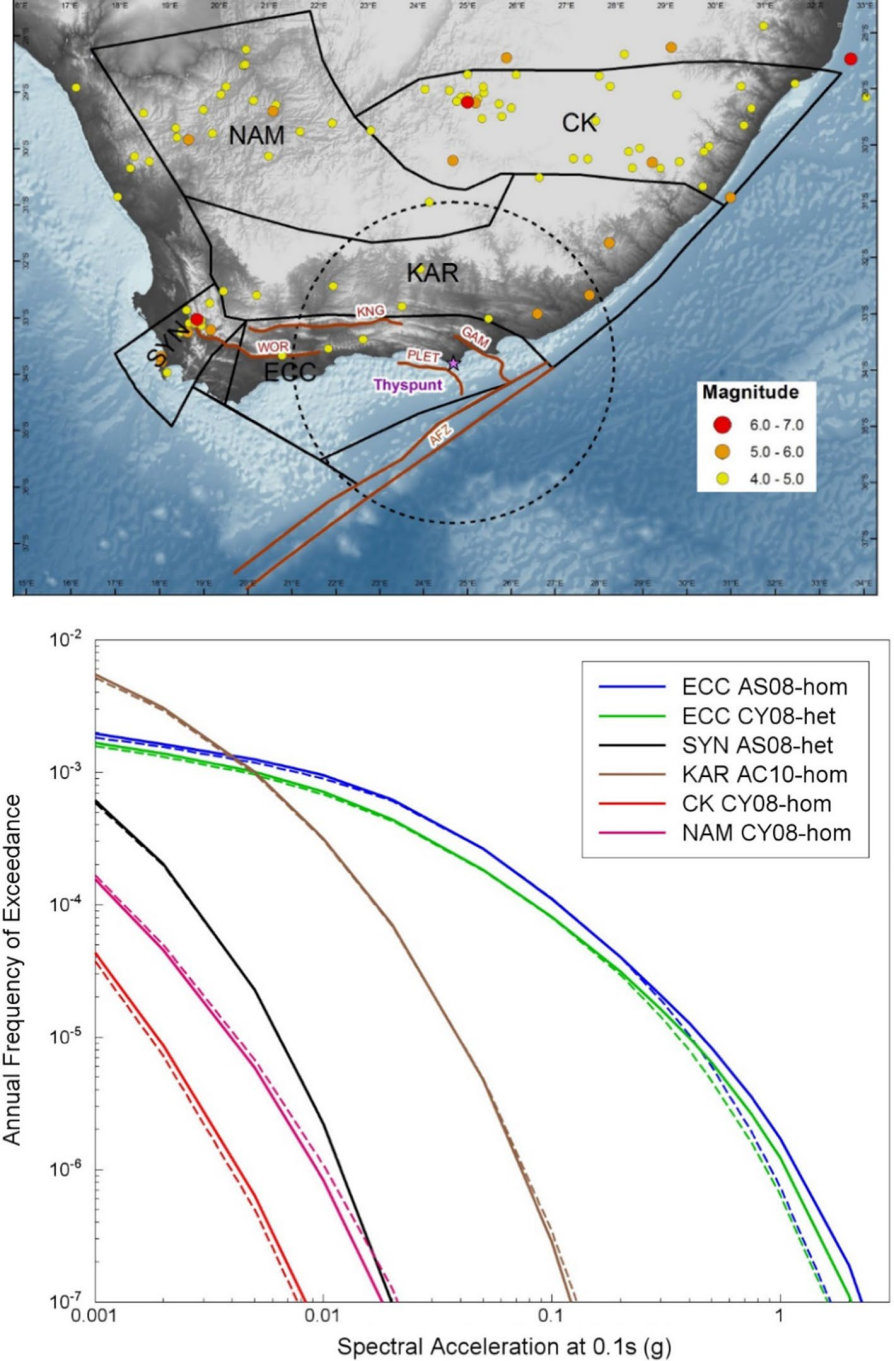
Upper: Seismic sources zones defined for the Thyspunt PSHA (Bommer et al. 2015b); lower: hazard curves obtained from parallel implementations in the FRISK88 (solid curves) and OpenQuake (dashed curves) software packages of the full SSC logic tree for each source zones in combination with a single branch from the GMC model (Bommer et al. 2013)

Formal QA on the PSHA calculations can include two separate elements. The first is that the code being used for the calculation has undergone a process of verification to confirm that it executes the calculations accurately. Valuable resources to this end are the hazard code validation and comparison exercises that have been conducted by the Pacific Earthquake Engineering Research (PEER) Center in California (Thomas et al. 2010; Hale et al. 2018). The second is confirmation that the SSC and GMC models have been correctly entered into the hazard calculation code, which is an important consideration for the logic trees developed for site-specific assessments at the sites of safety–critical structures such as NPPs, which will often have several hundred or even thousands of branch combinations. The GMC model can usually be checked exactly by predicting the median and 84-percentile ground-motion amplitudes for a large number of M-R combinations. For the PSHA for the Thyspunt nuclear site in South Africa (Bommer et al. 2015b), we performed such a check on the GMC model implementation with two independent implementations external to the main hazard code. For the SSC model, the full logic trees for individual sources were implemented, in combination with a selected branch from the GMC model, in two separate hazard codes by different teams of hazard analysts. The results were compared graphically (Fig. 35); the differences were seen to be small and not systematic, with higher hazard estimates being yielded by one code or the other for each source, suggesting that within the tolerance defined by the differences in the algorithms embedded in the codes (and in particular the generation of virtual ruptures), the results could be considered consistent and therefore confirmed the model implementation. While this is more rigorous than the approaches generally applied in PSHA studies, it does provide a robust check; a similar approach was implemented in the PSHA for the Hinkley Point C NPP site in the UK (Tromans et al. 2019).

### Documentation

The documentation of a PSHA study that fulfils all the objectives outlined above should do justice to the depth and rigour of the hazard assessment, and there can be little doubt that this will further enhance the likelihood of the study being accepted. The documentation should be complete and clear, explaining the evaluation of the data and models (including those that were not subsequently used), and providing technical justifications for all the final decisions, including the weights on the logic-tree branches. At the same time, the report should not be padded out with extraneous information that is subsequently not used in the model development (such as a long and detailed description of the entire geological history of the region, most of which is not invoked in the definition of the seismic sources). The one exception to this might be an overview of previous hazard studies for the site or region, which may not be used in the development of the current model but provide useful background and context for the reader.

As well as providing detailed information on the construction of the SSC and GMC models, the documentation should also enable others to reproduce the study. One element that assists with meeting this objective is to include what is referred to as Hazard Input Document (HID), which provides a summary of the models, including all details required for their implementation, but without any explanations or justifications. In major PSHA projects, the HID is usually passed to the hazard analysts for implementation in the code, and it also forms the basis for the QA checks summarised in the previous section. Tables of values and coefficients, and also of hazard results, can be usefully provided as electronic supplements to the PSHA report. There is value in the report also summarising the process that was followed and, in particular, the peer review and QA processes, pointing to separate documentation (ideally in appendices) providing more details.

**Fig. 36:**
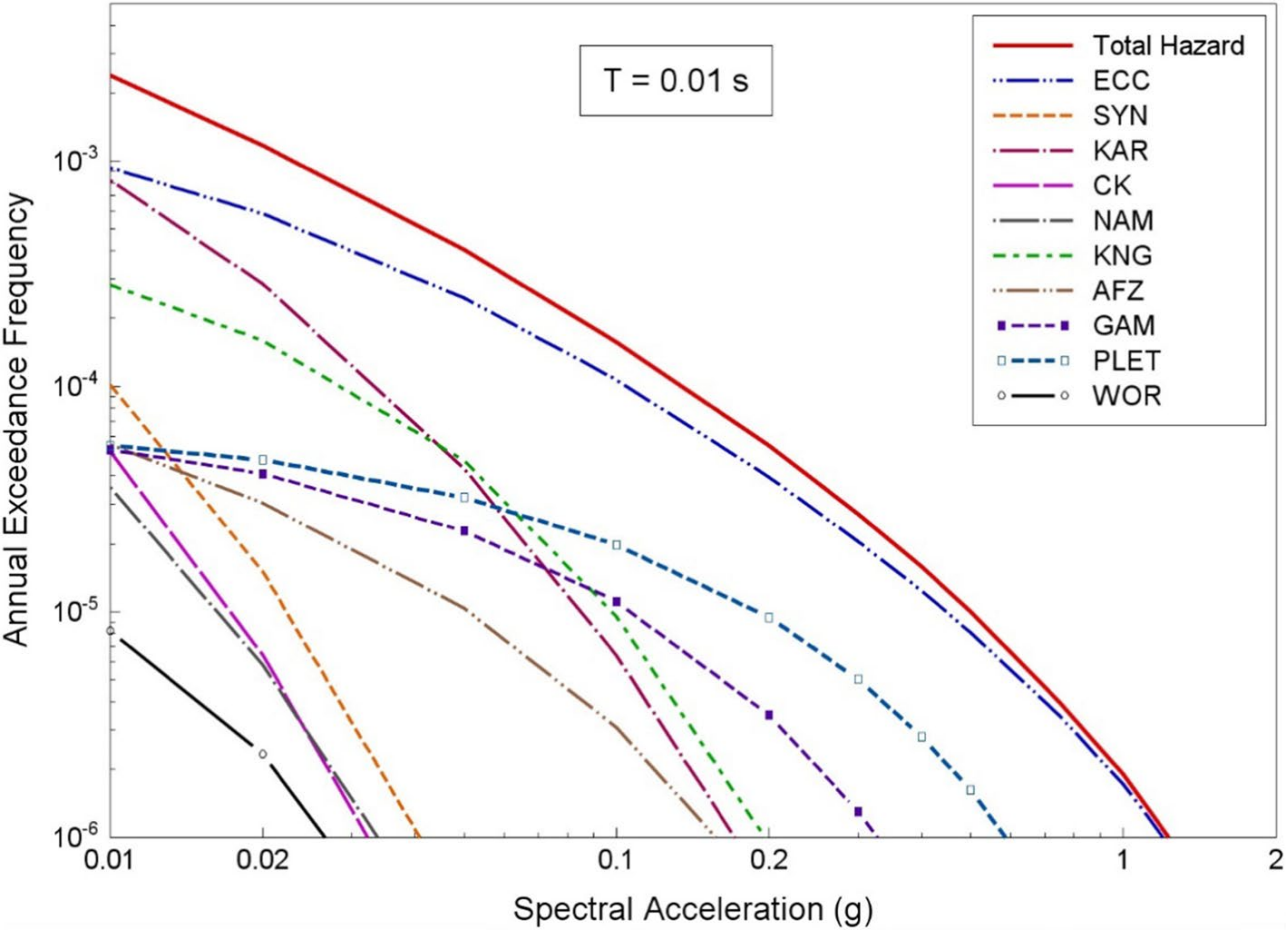
Contributions by individual seismic sources (see upper plot in Fig. 35) to the total hazard at the Thyspunt nuclear site in terms of the spectral acceleration at 0.01 s (Bommer et al. 2015b)

The hazard results will always be presented in the form of mean and fractile hazard curves, and for AFEs of relevance, it is common to also present uniform hazard response spectra (UHRS). For selected combinations of AFE and oscillator period, it is useful to show M-R-$\varepsilon$ disaggregation plots (see Fig. 24). There are several other ways of displaying disaggregation of the results that can afford useful insights into the PSHA results, including the hazard curves corresponding to individual seismic sources (Fig. 36).

**Fig. 37:**
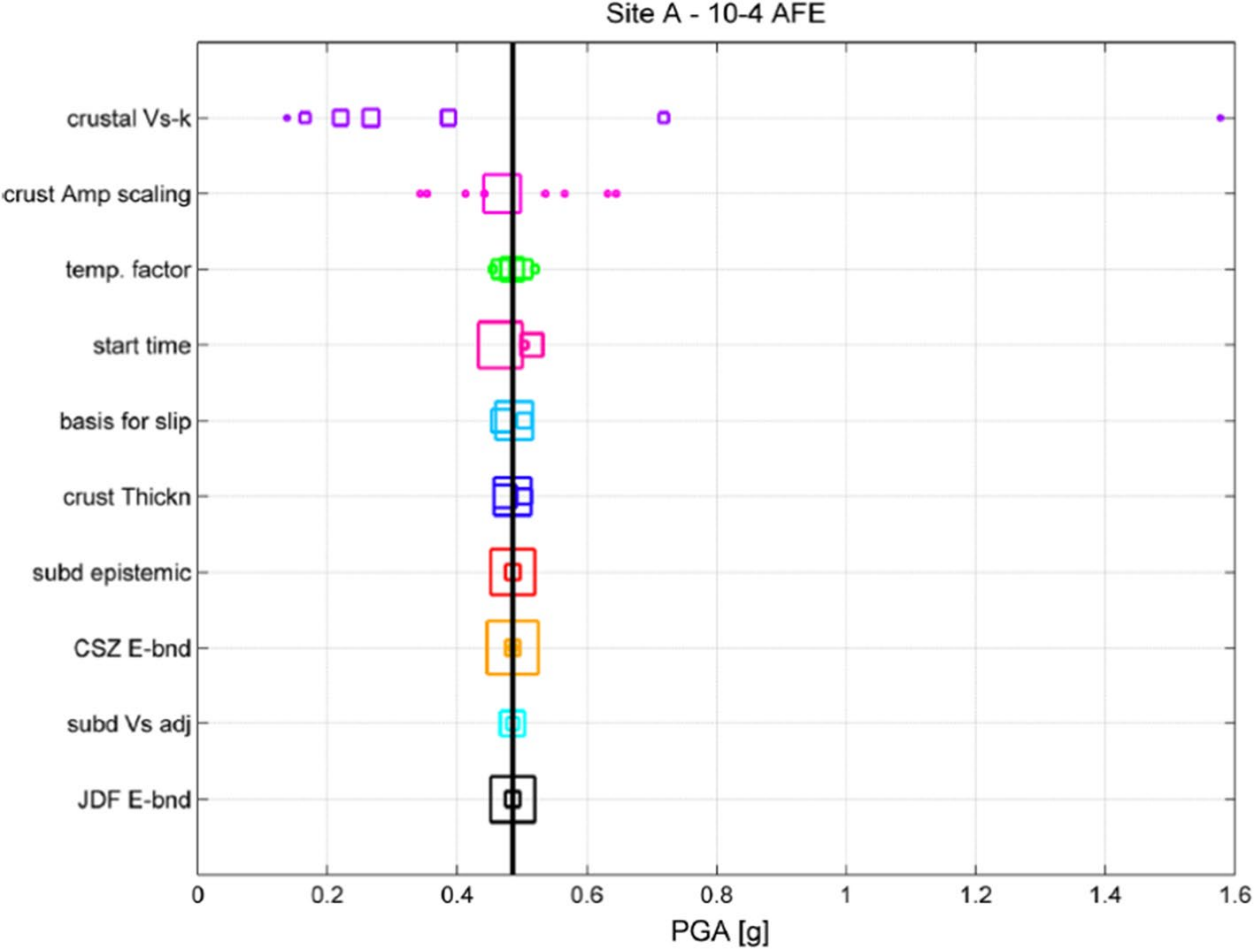
Tornado plot for the 10^–4^ AFE hazard estimate in terms of PGA at site A obtained in the Hanford site-wide PSHA (PNNL 2014); the black line corresponds to the mean hazard and the size of each symbol corresponds to the weight on the individual logic-tree branch

**Fig. 38:**
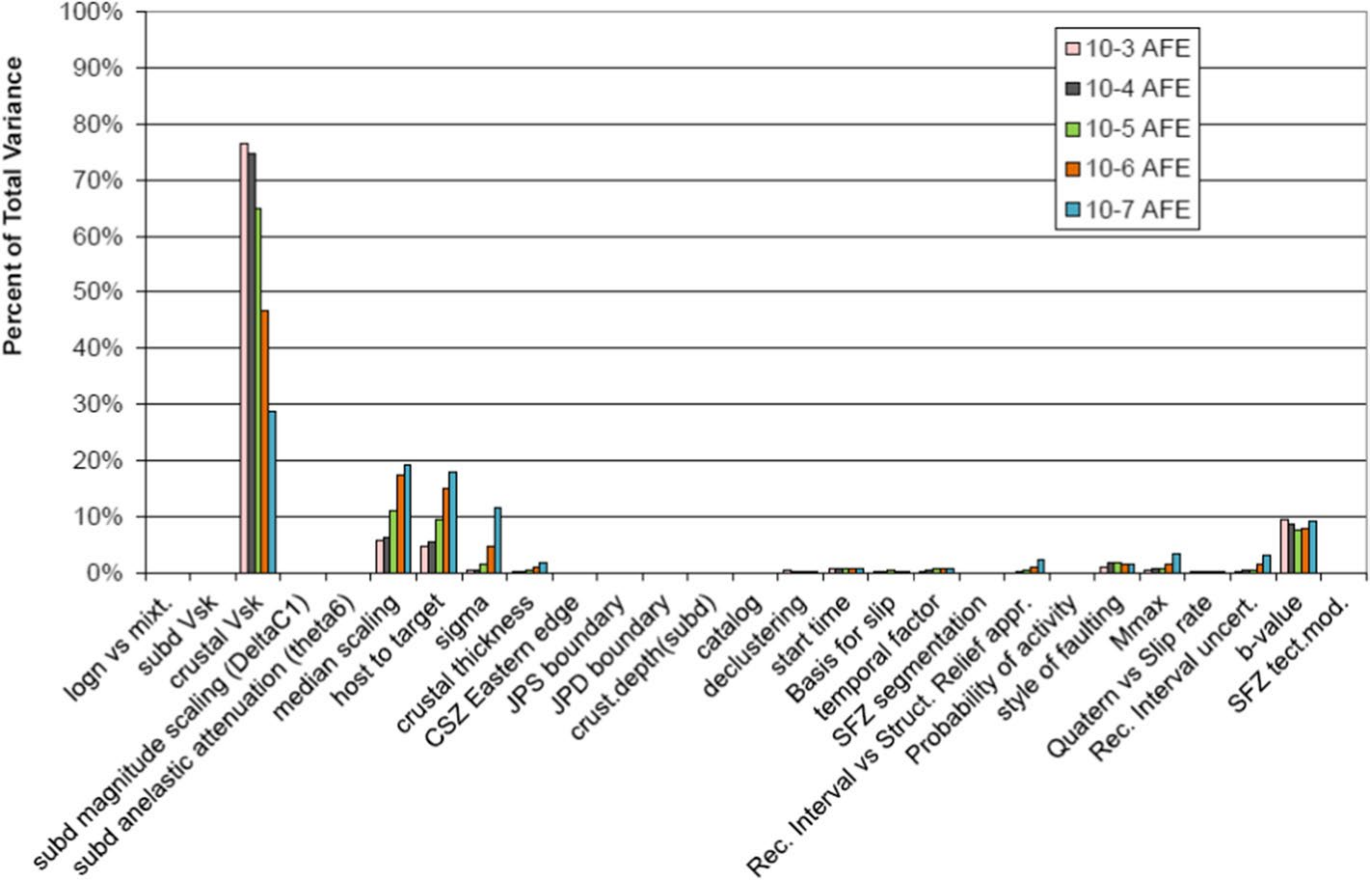
Variance plot for the hazard estimates in terms of PGA at site A for various AFEs as obtained in the Hanford site-wide PSHA (PNNL 2014)

There are also diagrams that can be included to display the individual contributions of different nodes of the logic tree to the total uncertainty in the final hazard estimates for any given ground-motion parameter and AFE. One of these is a tornado plot, which shows the deviations from the ground-motion value corresponding to the mean hazard associated with individual nodes (Fig. 37), and another is the variance plot, which shows nodal contributions to the overall uncertainty (Fig. 38).

Making PSHA reports publicly available can also be beneficial to the objective of obtaining broad acceptance for the hazard estimates, countering any accusations of secrecy or concealment of information, although in such cases, publication together with the final endorsement from the peer reviewers is advisable. In the United States, it is common practice to make site-specific PSHA studies for nuclear sites freely available (for example, the Hanford PSHA can be downloaded from https://www.hanford.gov/page.cfm/OfficialDocuments/HSPSHA). In other locations, public dissemination of site-specific PSHA reports is less common, but similar value in terms of demonstrating openness can be achieved through publication in the scientific literature of papers describing the studies, as has been done, very encouragingly, for recent hazard assessments at nuclear new-build sites in the UK (Tromans et al. 2019; Villani et al. 2020). Such articles can also contribute to the assurance associated with the study by virtue of having undergone peer review by the journal prior to publication. I would also note that dissemination of high-level PSHA studies, whether by release of the full reports or through publications in the literature, can also contribute to the improvement of the state of practice.

## Constructing input models for PSHA

From the preceding discussions, it should now be clear that the construction of SSC and GMC logic trees is clearly central to the execution of a successful PSHA. In this section, I discuss the development of such logic trees for site-specific hazard assessment. This is not intended as a comprehensive guide on how to construct SSC and GMC models, which would require the full length of this paper. The focus is very specifically on recent developments, most of which have arisen from experience on high-level PSHA projects for nuclear sites, which assist in the construction of logic trees that fulfil their intended purpose. The first sub-section discusses and defines exactly what is the purpose of logic trees, and then their application is discussed for ground-motion predictions in rock, for adjustments for local site effects, and for seismic source characterisation models. The order may seem somewhat illogical since the SSC model would normally be the starting point for a PSHA. The reason for reversing the order here is that recent innovations in GMC modelling have made the construction of logic trees much more clearly aligned with their purpose, and these improvements have also now been adapted to site response modelling; the final sub-section discusses the possibility, and indeed the necessity, of adapting the same approaches to SSC modelling.

### The purpose of logic trees

As noted in sub-Sect. 4.2, all PSHA studies now employ logic trees but this is often done without a clear appreciation of the purpose of this tool. In many cases, one is left with the impression that the logic tree constructed for the inputs to the hazard calculations is simply a gesture to acknowledge the existence of epistemic uncertainty and to demonstrate that more than one model or parameter value has been considered for each of the key elements of the SSC and GMC models.

**Fig. 39:**
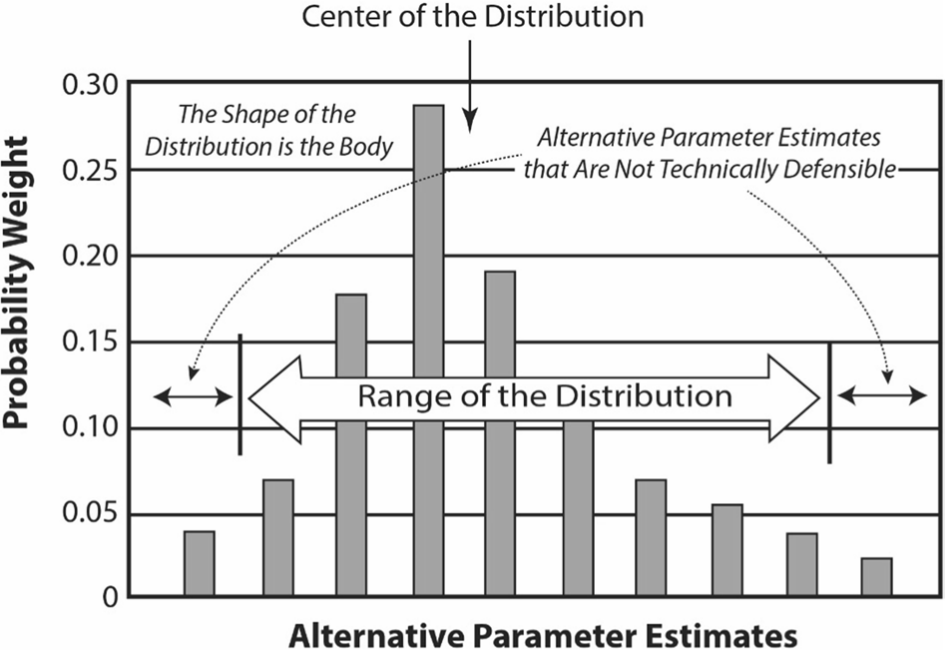
Schematic illustration of the concepts of centre, body, and range in relation to the distribution of a specific parameter implied by a node or set of nodes on a logic tree (USNRC 2018)

The purpose of a logic tree in PSHA is to ensure that the hazard results reflect the full distribution of epistemic uncertainty, capturing the best estimate of the site hazard as constrained by the available data and the associated range of possible alternative estimates due to the epistemic uncertainty in the SSC and GMC models. The purpose of the SSC and GMC logic trees has been stated as representing the centre, the body, and the range of technically defensible interpretations of the available data, methods, and models, which is often abbreviated as the CBR of TDI (USNRC 2018). The ‘centre’ could be understood as the model or parameter value considered to be the best estimate or choice for the region or site based on the modeller’s interpretation of the currently available data. The ‘body’ could be understood as the alternative interpretations that could be made of the same data, and the ‘range’ as the possibilities that lie beyond the currently available data (but which must be physically realisable). Figure 39 illustrates these three concepts in relation to the distribution of a single parameter in the SSC or GMC logic tree.

A point to be stressed very strongly is that the distributions implied by the logic tree are intended to represent the CBR of TDI of the factors that drive the hazard estimates at the site. For the SSC model, these factors are the location (and hence distance) and recurrence rate of earthquakes of different magnitude, and the maximum magnitude, Mmax. For the GMC model, the factor is the amplitude—defined by the median predictions and the associated sigma values—of the selected ground-motion parameter at the site due to each magnitude-distance pair defined by the SSC model. The logic tree is not intended to be a display and ranking, like a beauty contest, of available models. All available data and models that may be relevant to the characterisation of the hazard at the site should be considered in the development of the logic tree, but there is absolutely no requirement to include all the available models in the final logic tree. Models that are not included in the logic tree are not really being assigned a zero weight, which could be interpreted to imply that the model has been evaluated as irrelevant (possibly by virtue of being very similar to another model that is already included) or unreliable; the model may simply not be needed for the logic tree to capture the full CBR of the variables of interest: earthquake locations and recurrence rates, Mmax, median ground-motion predictions, and sigma in the ground-motion prediction. All models considered should appear in the PSHA documentation but none of them needs to feature in the logic trees, especially if it is finally decided to construct new models instead of using existing ones.

There has been much debate in the literature regarding the nature and meaning of the weights assigned to the branches of logic trees (Abrahamson and Bommer 2005; McGuire et al. 2005; Musson 2005, 2012a; Scherbaum and Kuehn 2011; Bommer 2012). The weights are assigned as relative indicators of the perceived merit of each alternative model or parameter value; the absolute value of the weights is not the critical feature but rather the ratios of the weights on the branches at each node: a branch assigned a weight of 0.3 is considered three times more likely to be the optimal model or value than a branch with a weight of 0.1. A potential pitfall in debates that focus on the interpretation of logic-tree branch weights is that we can lose sight of the fact that all that matters in the end is the full distribution that results from the combination of the branches and their associated weights (i.e., both axes of the histogram in Fig. 39). Moreover, for logic trees with any appreciable number of branches, the hazard results are generally found to be far more sensitive to the branches themselves (i.e., models or parameter values) than to the weights (e.g., Sabetta et al. 2005).

Regardless of how the weights are assigned, in generating the outputs from the PSHA (mean hazard and fractiles) they are treated as probabilities. Since this is the case, it is desirable that the branches satisfy the MECE (mutually exclusive and collectively exhaustive) criterion; the latter should always be achieved since no viable option should be omitted from the logic tree, but it can be challenging in some cases to develop logic-tree branches that are mutually exclusive.

### Ground motion models

As stated above, the objective of a GMC logic tree is to define the CBR of predicted ground-motion amplitudes for any combination of magnitude, distance and other independent variables defined in the SSC model for a PSHA. The amplitudes are a function of the median predictions from the GMMs and their associated sigma values.

#### Median predictions: multiple GMM vs backbone GMM

**Fig. 40:**
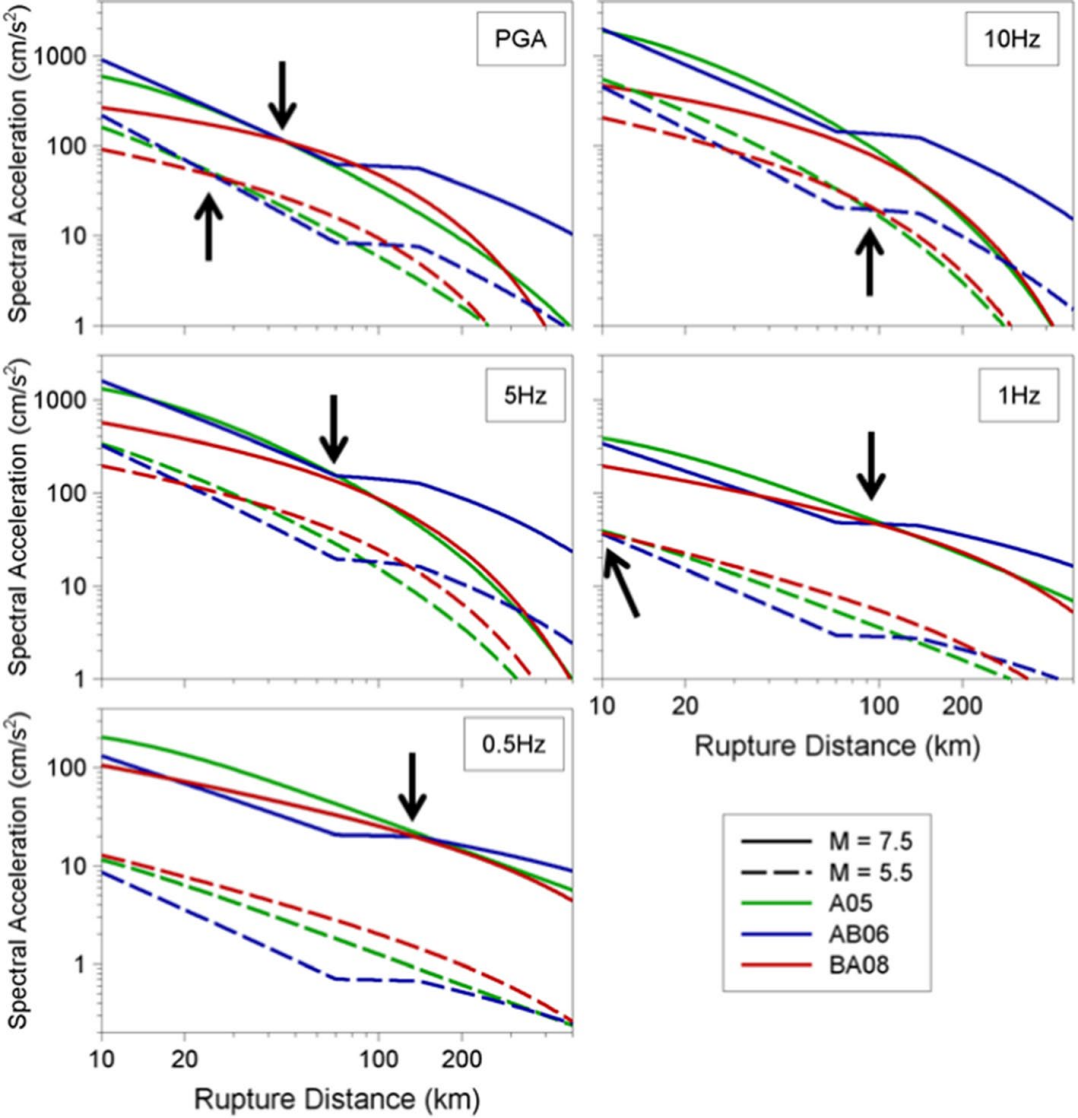
Median predictions of PGA and spectral accelerations at different oscillator frequencies from the GMMs of Atkinson (2005), Atkinson and Boore (2006), and Boore and Atkinson (2008) (for **M** 5.5 and **M** 7.5 plotted against distance; the arrows indicate magnitude-distance combinations for which the three median predictions converge

The first logic tree to include a node for the GMC model, to my knowledge, was presented by Coppersmith and Youngs (1986): the logic tree included a single GMC-related node with two equally weighted branches carrying published GMMs. The practice of building GMC logic trees evolved over the ensuing years, but the basic approach was maintained: the branches were populated with published GMMs (or occasionally with new GMMs derived specifically for the project in question), and relative weights assigned to each branch. There are several pitfalls and shortcomings in this approach, one of which is illustrated in Fig. 40.

The plots in Fig. 40 show median predictions from the three GMMs that populated the logic tree defined for a PSHA conducted for major infrastructure in North America, located in the transition region between the active tectonics of the west and the stable continental interior of the east. The arrows highlight several magnitude-distance combinations for which the predictions from the three GMMs converge to almost exactly the same value. Consequently, for these M-R pairs, the logic tree is effectively communicating that there is no epistemic uncertainty in the predictions of response spectral acceleration, which cannot be the case. One might think that the solution is to increase the number of branches, but this can actually result in very peaked distributions since many GMMs are derived from common databases.

**Fig. 41:**
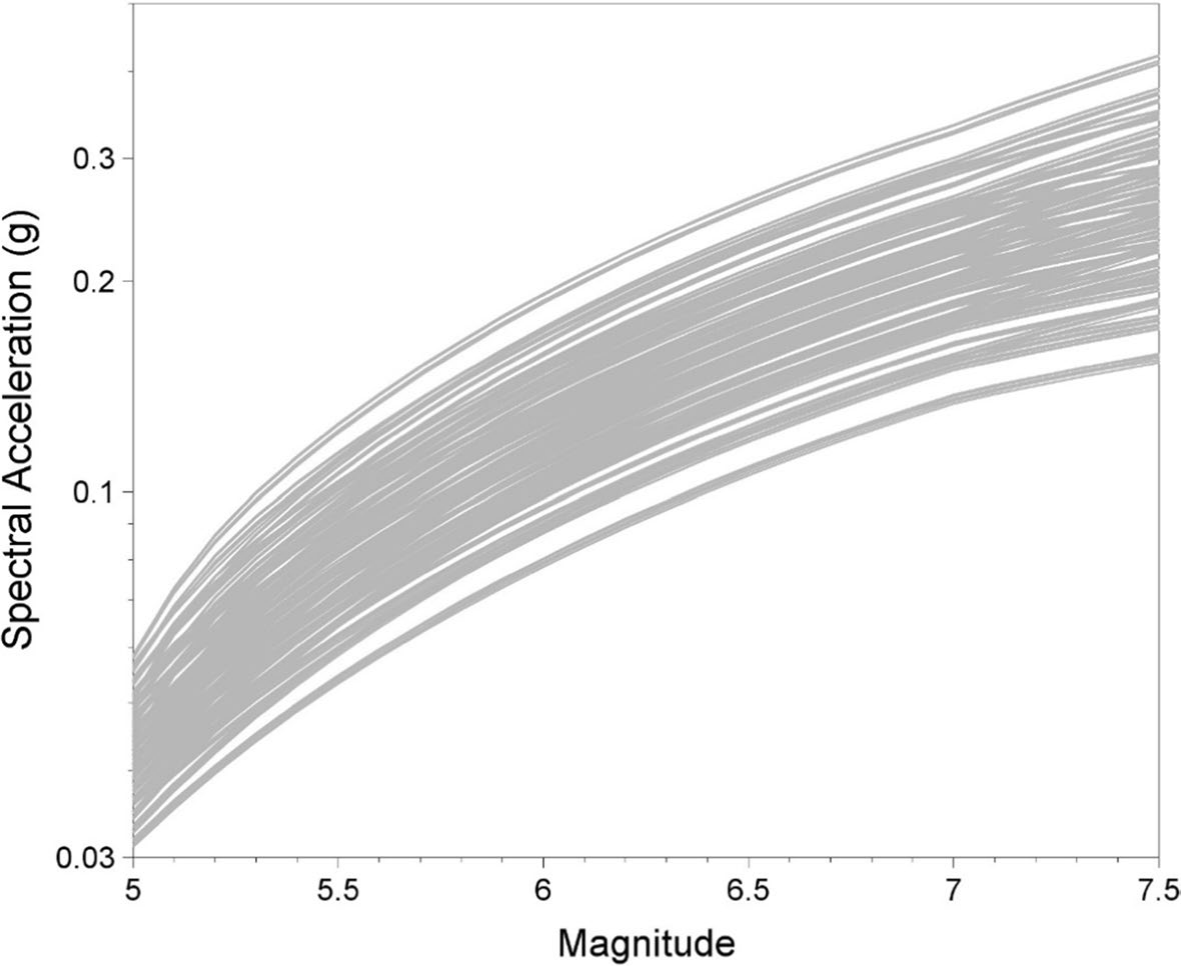
Predicted median spectral accelerations at a given period obtained from a logic tree constructed using a backbone approach, for a fixed distance and V_S30_, as a function of magnitude

The fundamental problem with the multiple GMM approach to constructing logic trees is that the target distribution of ground-motion amplitudes that results from several weighted models is largely unknown. Different tools have been proposed to enable visualisation of the resulting ground-motion distribution, including composite models (Scherbaum et al. 2005) and Sammons maps (Scherbaum et al. 2010). Such tools are generally not required, however, if the GMC logic tree is constructed by populating the branches with alternative scaled models of a single GMM, which has been given the name of a backbone GMM approach (Bommer 2012). In its simplest form, the backbone GMM is simply scaled by constant factors, but many more sophisticated variations are possible, with the scaling varying with magnitude and/or distance. In the example shown in Fig. 41, it can be appreciated that the spread of the predictions increases with magnitude, reflecting the larger epistemic uncertainty where data are sparser. What can also be clearly appreciated is that the relationship between the branch weights and the resulting distribution of predicted accelerations is much more transparent than in the case where the logic tree is constructed using a number of different published GMMs.

**Fig. 42:**
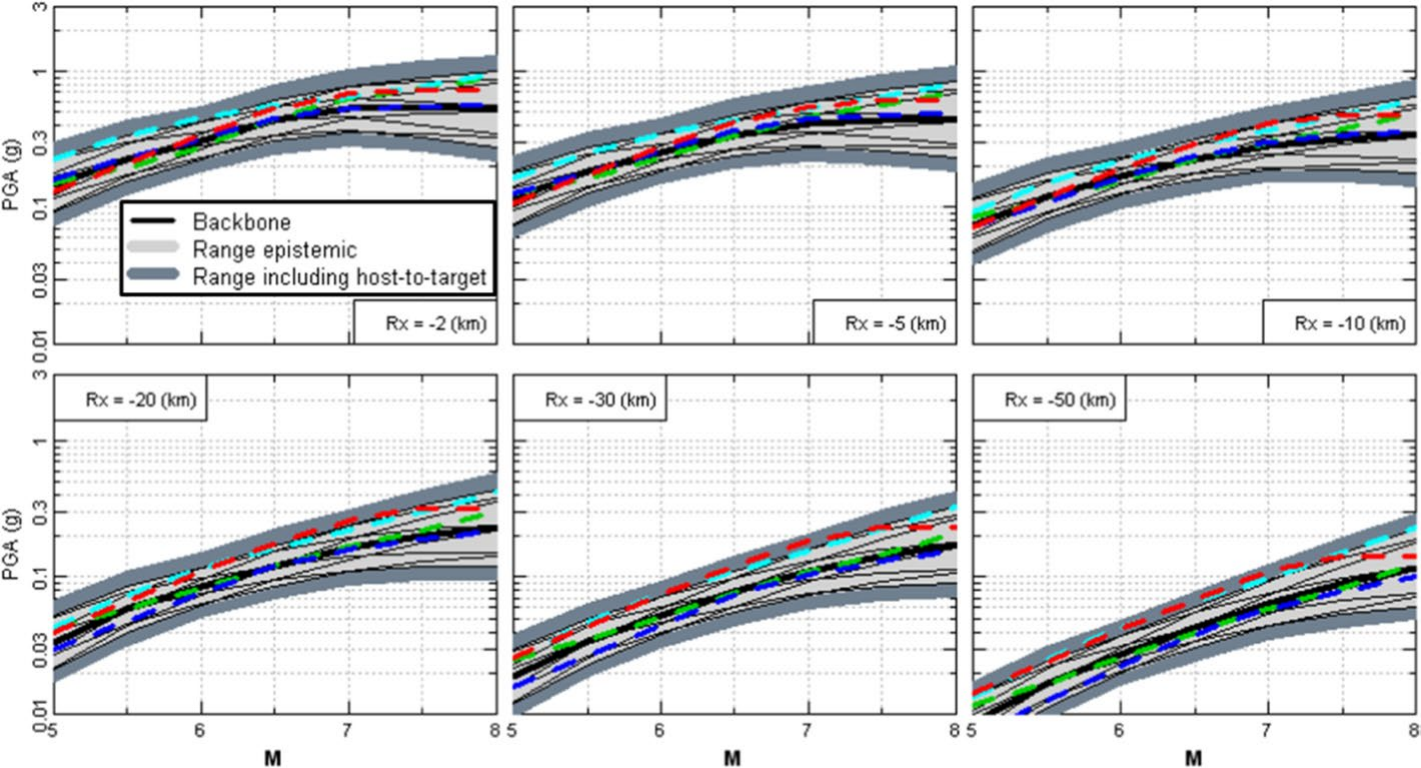
Predicted median PGA values from the Hanford GMC logic tree, as a function of magnitude for different distances. The solid black line is the backbone GMM, and the thin black lines the other models from the same host region, which collectively define the inherent uncertainty (light grey shading); the dark grey shading corresponds to the additional uncertainty associated with adjusting the backbone GMM to the characteristics of the target region and site; the dashed, coloured curves are other GMMs not used in the model development but plotted for comparative purposes (PNNL 2014)

In addition to the clearer relationship between the logic tree branches and the resulting ground-motion distribution, and the consistent width of the distribution that avoids the ‘pinching’ seen in Fig. 40, there are other advantages of the backbone approach, each of which really highlights a shortcoming in the multiple GMM approach. One of these is the fact that in using the latter approach, there is an implicit assumption that the range of predictions from the available GMMs that happen to have been published covers the range of epistemic uncertainty. In practice, this is very unlikely to be the case, and even in regions with abundant ground-motion data, such as California, it is recognised that the range of predicted values from local GMMs, like the NGA-West2 models (Gregor et al. 2014) does not capture the full range of epistemic uncertainty in ground-motion predictions for that region (Al Atik and Youngs 2014). If the same models are used to populate a GMC logic tree for application to another region (with less abundant ground-motion data), an even broader additional range of epistemic uncertainty is likely to be required. Figure 42 illustrates the backbone GMM model developed in the Hanford PSHA project (PNNL 2014), in which the total range of epistemic uncertainty comes from the inherent uncertainty associated with the backbone GMM in its host region (light grey shading) and the additional uncertainty associated with adjusting the backbone GMM for applicability to source and path characteristics in the target region and to the rock profile at the Hanford site (dark grey shading).

The backbone GMM approach has already been widely applied, in various different forms, and its use predates the introduction of the term backbone now used to describe it (Bommer 2012; Atkinson et al. 2014). The backbone approach is fast becoming standard practice in high-level PSHA studies for critical sites (e.g. Douglas 2018), and I would argue that in the light of the shortcomings it has highlighted in the multiple GMM approach, rather than there being a need to make the case for using the backbone approach, it would actually be challenging to justify the continued use of the multiple GMM approach.

#### Median predictions: adjustments to regional and local conditions

A legacy of the widely used approach of constructing GMC logic trees by populating the branches with published GMMs has been a focus on approaches to selecting GMMs that are applicable to the target region. Many studies have looked into the use of locally recorded ground-motion data to test and rank the applicability of candidate GMMs (Scherbaum et al. 2004b, 2009; Arango et al. 2012; Kale and Akkar 2013; Mak et al. 2017; Cremen et al. 2020; Sunny et al. 2022). In many applications, the only data available for such testing are recordings from small-magnitude earthquakes, which may not provide reliable indications of the GMM performance in the larger magnitude ranges relevant to hazard assessment (Beauval et al. 2012).

In parallel with the focus on selection on the basis of inferred applicability to the target region, work also developed to make adjustments to GMMs from one region, usually referred to as the host region, to make them more applicable to the target region where the hazard is being assessed. I believe that this approach should be strongly preferred since the degree to which two regions can be identical in terms of ground-motion characteristics is obviously open to question: if the selection is based on testing that simply identifies the most applicable models (in terms of how well they replicate local data), it does not necessarily mean that these GMMs are genuinely applicable to the target region without further adjustment. Moreover, even if the source and site characteristics of the host and target regions are genuinely similar, it is unlikely that the generic site amplification in any GMM will match the target site characteristics (an issue discussed further in sub-Sect. 5.3). With these considerations in mind, Cotton et al. (2006) proposed a list of selection criteria, all of which were designed to exclude poorly derived GMMs that are unlikely to extrapolate well to larger magnitudes and all the distances covered by hazard integrations, and also to exclude models from clearly inappropriate settings (i.e., subduction-region GMMs for crustal seismic sources). The selected models were adjusted for parameter compatibility, and then adjusted to match the target source, path, and site conditions.

The general approach proposed by Cotton et al. (2006) has continued to evolve since first proposed, with Bommer et al. (2010) formalising the list of exclusion criteria and making them more specific. The most important developments, however, have been in how to adjust the selected GMMs to the target region and site. Atkinson (2008) proposed adjusting empirical GMMs to better fit local data, starting with inspection of the residuals of the local data with respect to the model predictions. This so-called referenced empirical approach is relatively simple to implement but suffers from important drawbacks: if the local data are from predominantly small-magnitude earthquakes, the approach is not well suited to capturing source characteristics in the target region, and for a site-specific study, unless the local database includes a large number of recordings from the target site, it will not help to better match the target site conditions. Another approach is to use local recordings, even from small-magnitude events, to infer source, path, and site parameters for the target region. The main parameters of interest are as follows:

- The stress drop, or more correctly, the stress parameter, $\Delta \sigma$, which is a measure of the intensity of the high-frequency radiation in an earthquake
- The geometric spreading pattern, which describes the elastic process of diminishing energy over distance as the wavefront becomes larger
- The quality factor, $Q$, which is a measure of the anelastic attenuation in the region, with higher values implying lower rates of attenuation with distance
- The site damping parameter, ${\kappa }_{0}$, which is a measure of the high-frequency attenuation that occurs at the site; contrary to the parameter $Q$, a higher value of ${\kappa }_{0}$ means greater attenuation

**Fig. 43:**
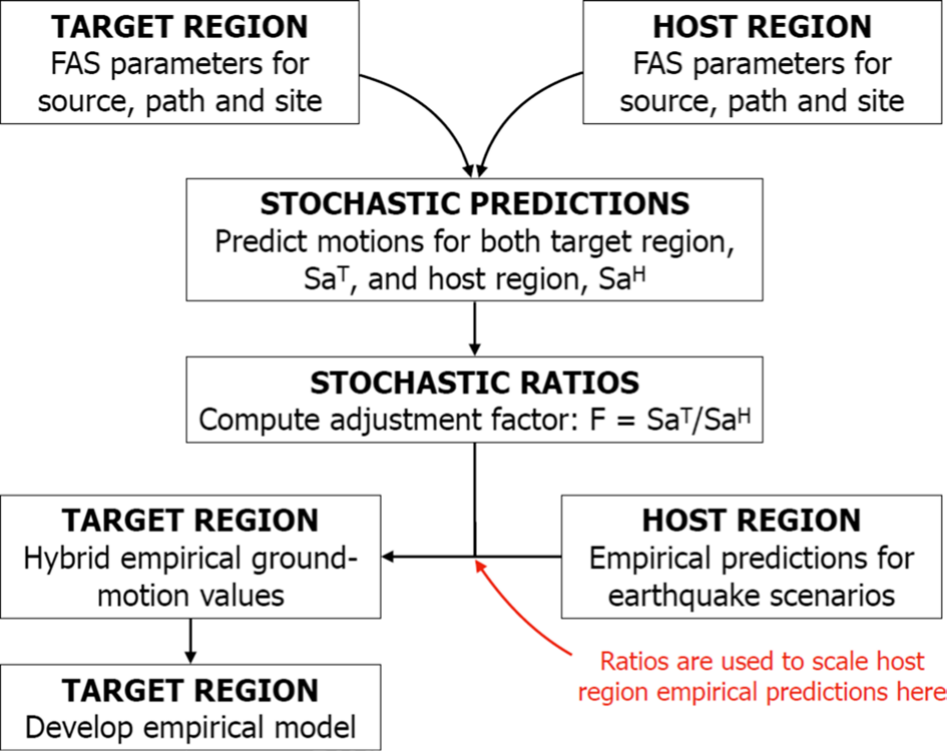
Illustration of hybrid-empirical adjustments to transform a GMM from its host (H) region to the target (T) region where the PSHA is being conducted; FAS is Fourier amplitude spectrum and Sa is spectral acceleration (Bommer and Stafford 2020)

Boore (2003) provides a very clear overview of how these parameters can be determined, and then used to generate Fourier amplitude spectra (FAS), which can then be transformed to response spectra by making some assumptions regarding signal durations. Once a suite of such parameters is available, they can be used to generate GMMs through stochastic simulations. Hassani and Atkinson (2018) performed very large numbers of such simulations to generate stochastic GMMs that could be locally calibrated by specifying local values of $\Delta \sigma$, $Q$, and ${\kappa }_{0}$. While this is a very convenient tool, the simulations are based on a point-source model of earthquakes, hence finite rupture effects in the near field are not well captured. There is consequently strong motivation to retain the advantages offered by empirical GMMs, which prompted Campbell (2003) to propose the hybrid-empirical method to adjust empirical GMMs from one region to another. The basis of the hybrid empirical method is to determine suites of source, path, and site parameters (i.e., $\Delta \sigma$, $Q$, and ${\kappa }_{0}$) for both the host and target regions, and then to use these, via FAS-based simulations, to derive ratios of the spectral accelerations in the host and target regions, which are then used to make the adjustments (Fig. 43). This is essentially the approach that was used by Cotton et al. (2006) to adjust the selected GMMs to the target region.

**Fig. 44:**
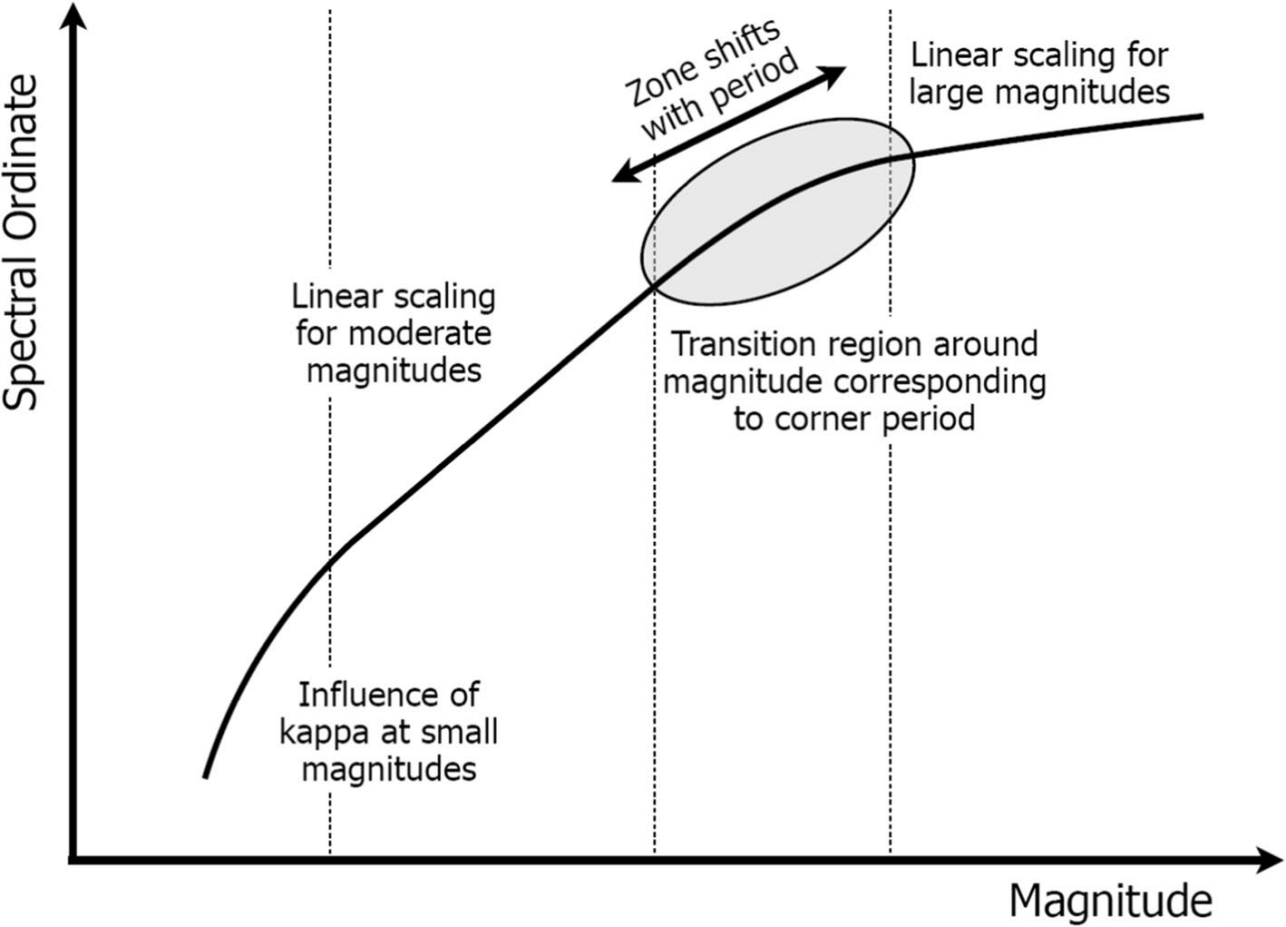
Theoretical scaling of Sa with magnitude arising from consideration of a point- source FAS (Bommer and Stafford 2020); the magnitude at which the transition from moderate-magnitude scaling to large-magnitude scaling occurs varies with oscillator period

Within the general framework in which selected GMMs are adjusted to be applicable to the target region and site, it clearly becomes less important to try to identify models that are approximately applicable to the target region, unless one perceives benefits in minimising the degree of modification required. An alternative approach is to select GMMs on the basis of how well suited they are to being modified. As Fig. 43 shows, at the core of the hybrid-empirical adjustments is the assumption that ratios of FAS can serve as a proxy for scaling of response spectral accelerations, Sa. Since the relationship between Sa and FAS is complex (Bora et al. 2016), especially at higher frequencies, the method works better if the scaling of Sa implicit in the empirical GMM is consistent with the scaling of FAS from seismological theory. This applies, in particular, to the scaling with magnitude (Fig. 44).

Another refinement that has been proposed is to make the adjustments for host-to-target region differences separately for each factor rather than collectively as in the original method of Campbell (2003). This has the advantage that the uncertainty in the estimates of the parameters such as $\Delta \sigma$, $Q$, and ${\kappa }_{0}$ can be modelled explicitly, thus creating a more tractable representation of the epistemic uncertainty. For this to be possible, the selected GMM should have a functional form that isolates the influence of individual factors such as $\Delta \sigma$, $Q$, and ${\kappa }_{0}$. If such a model can be identified, then the backbone and hybrid-empirical approaches can be combined to construct the logic tree. The adjustable GMM is selected as the backbone and then the GMC logic tree is constructed through a series of nodes for host-to-target region adjustments. The NGA-West2 model of Chiou and Youngs (2014) has been identified as the most adaptable of all current GMMs for active crustal seismicity, having a functional form that both conforms to the scaling illustrated in Fig. 44 and also isolates the influence of $\Delta \sigma$ and $Q$ in individual terms of the model (Bommer and Stafford 2020). The Chiou and Youngs (2014) GMM also has the added advantage of magnitude-dependent anelastic attenuation, which allows a reliable host-to-target region adjustment for path effects to be made even if only recordings of small-magnitude earthquakes are available. For the stress parameter adjustment, however, the magnitude scaling of stress drop would need to be accounted for in the uncertainty bounds on that node of the logic tree.

In addition to scaling consistent with seismological theory and the isolated influence of individual parameters, a third criterion required for an adaptable GMM is a good characterisation of source, path, and site properties of the host region. This is not straightforward because determination of the required parameters for the host region would need to have been made assuming geometric spreading consistent with that implicit in the GMM. Moreover, there may be no clearly defined host region, even for a nominally Californian model such as Chiou and Youngs (2014), since many of the accelerograms in their database, especially for larger magnitudes, were recorded in other parts of the world. Therefore, rather than seeking a suite of source, path, and site parameters for the host region of the backbone GMM, inversions can be performed that define a suite of parameters (for a virtual host region) that are fully consistent with the backbone model (Scherbaum et al. 2006). Al Atik and Abrahamson (2021) have inverted several GMMs, including Chiou and Youngs (2014), hereafter CY14, to obtain model-consistent site profiles of shear-wave velocity, V_S_, and ${\kappa }_{0}$; Stafford et al. (2022) then used these to invert CY14 for source and path properties. The suites of parameters obtained by Al Atik and Abrahamson (2021) and by Stafford et al. (2022) fully define the host region of CY14; inversion of ground-motion FAS in the target region then allows the construction of a GMC logic tree consisting of successive nodes for source, path, and site adjustments (although, as discussed in sub-Sect. 5.3, the site adjustment should generally be made separately).

In closing, it is important to highlight that this should not be interpreted to mean that CY14 is a perfect GMM or that all other GMMs cease to be of any use. With regards to the first point, it is worth noting that only 8% of the earthquakes in the CY14 database were associated with normal ruptures, so for applications to seismic sources dominated by normal-faulting earthquakes, this might be viewed as an additional source of epistemic uncertainty. Additionally, the derivation of CY14, in line with the earlier Chiou and Youngs (2008) models, assumed that the records with usable spectral ordinates at long periods represented a biased sample of high-amplitude motions; their adjustment for this inference resulted in appreciably lower predicted spectral accelerations at long periods than are obtained from the other NGA-West2 models, and this divergence might also be considered an epistemic uncertainty since both approaches can be considered to be technically defensible interpretations.

#### Sigma values

As was made clear in sub-Sect. 2.2.3, ground-motion prediction models predict distributions of ground-motion amplitudes rather than unique values for an M-R combination, hence sigma is as much part of a GMM as the coefficients that define the median values, and therefore must also be included in the GMC logic tree. In early practice, each published GMM included in the logic tree was accompanied by its own sigma value, but it has become more common practice now to have a separate node for sigma values. This has been motivated primarily by the recognition of adjustments that need to be made to these sigma values when local site amplification effects are rigorously incorporated into PSHA (as described in the next section).

Empirical models for ground-motion variability invoke what is known as the ergodic assumption (Anderson and Brune 1999), which means that spatial variations are used as a proxy for temporal variation. The required information is how much ground motions vary at a single location over time, or in other words over many different earthquakes occurring in the surrounding region. In practice, strong-motion databases tend to include, at most, records obtained over a few decades, and consequently the variation of the ground-motion amplitudes from site to site is used as a proxy for the variation over time at a single location. However, for accelerograph stations that have generated large numbers of recordings, it is observed that the variability of the motions is appreciably smaller than predicted by the ergodic sigmas associated with GMMs (Atkinson 2006). The reason that this is the case is that a component of the observed spatial variability in ground-motion residuals actually corresponds to repeatable amplification effects at individual sites. The decomposition of the variability presented in Eq. (2) can now be further broken down as follows:

5 $$\sigma = \sqrt{{\tau }^{2}+{\phi }^{2}}=\sqrt{{\tau }^{2}+{\phi }_{ss}^{2}+{\phi }_{S2S}^{2}}$$

where ${\phi }_{S2S}$ is the site-to-site variability (or the contribution to total variability due to the differences in systematic site effects at individual locations) and ${\phi }_{ss}$ is the variability at a single location. If the systematic site amplification effect at a specific location can be constrained by large numbers of recordings of earthquakes covering a range of magnitude and distance combinations, then the last term in Eq. (5) can be removed, and we can define a single-station or partially non-ergodic sigma:

6 $${\sigma }_{ss}=\sqrt{{\tau }^{2}+{\phi }_{ss}^{2}}$$

In practice, it would be rather unlikely that at the site of major engineering project (for which a PSHA is to be conducted), we have a large number of ground-motion recordings. However, if such information were available, then it would constrain the systematic site effect, hence the absence of this knowledge implies that for the target site ${\phi }_{S2S}$ actually represents an epistemic uncertainty. If, as should always be the case, the site-specific PSHA includes modelling of local site amplification factors, capturing the epistemic uncertainty in the amplifications, then it is necessary to invoke single-station sigma, to avoid double counting the site-to-site contribution. Using datasets from recording sites yielding large numbers of accelerograms in many locations around the world, Rodriguez-Marek et al. (2013) found that estimates of single-station variability, ${\phi }_{ss}$, are remarkably stable, and these estimates therefore can be adopted in PSHA studies.

The concept of non-ergodic sigma has been extended to also include repeatable site and path effects, such that for ground motions recorded at a single location due to earthquakes occurring in a single seismic source, even lower variability is observed (e.g., Lin et al. 2011). Using these concepts, fully non-ergodic GMMs have been developed (e.g., Landwehr et al. 2016) and used in PSHA (Abrahamson et al. 2019). The advantage that these developments bring is a more accurate separation of aleatory variability and epistemic uncertainty, allowing identification of the elements of uncertainty that have the potential to be reduced through new data collection and analysis.

Reflecting the marked influence that sigma has on seismic hazard estimates, especially at the low AFEs relevant to safety–critical facilities, several studies have explored additional refinements of sigma models. Using their model for spatial correlation of ground-motion residuals (Jayaram and Baker 2009), Jayaram and Baker (2010) showed that accounting for this correlation in the regressions to derive GMMs results in smaller values of between-earthquake variability and greater values of within-earthquake variability. The net effect tends to be an increase in single-station sigma for larger magnitudes and longer periods, but the impact is modest and would only need be accounted for in PSHA studies in very active regions that are targeting small AFEs (i.e., hazard analyses that will sample large values of $\varepsilon$).

**Fig. 45:**
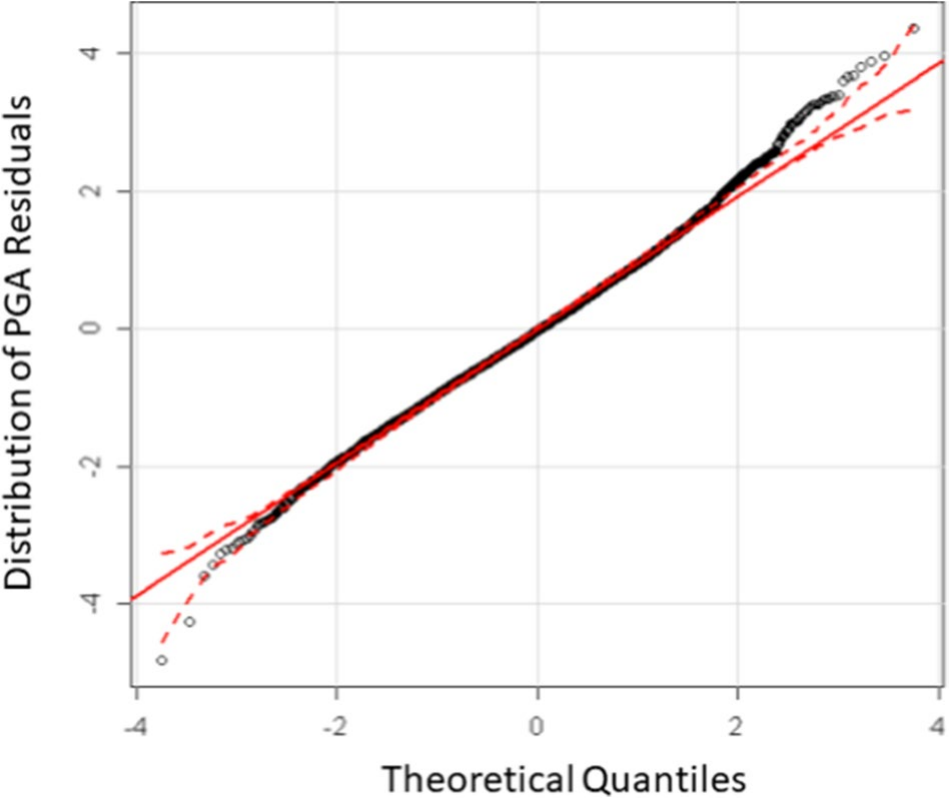
Event- and site-corrected residuals of PGA from the Abrahamson et al. (2014) GMM plotted against theoretical quartiles for a log-normal distribution. If the residuals conformed to a log-normal distribution, they would lie on the solid red line; the dashed red lines show the 95% confidence interval (modified from PNNL 2014)

Another subtle refinement that has been investigated is the nature of the tails of the residual distributions. Early studies (e.g., Bommer et al. 2004b) showed that ground-motion residuals conformed well to the log-normal distribution at least to ± 2 $\sigma$ and deviations beyond these limits were interpreted to be due to insufficient sampling of the higher quantiles by the relatively small datasets available at the time. Subsequently, as much larger ground-motion datasets became available, it became apparent that the deviations may well be systematic and indicate higher probabilities of these higher residuals than predicted by the log-normal distribution (Fig. 45). In some projects, this has been accommodated by using a mixture model that defines a weighted combination of two log-normal distributions in order to mimic the ‘heavy tails.’ Again, this is a refinement that is only likely to impact on the hazard results at low AFEs and in regions of high activity.

### Incorporating site response into PSHA

**Fig. 46:**
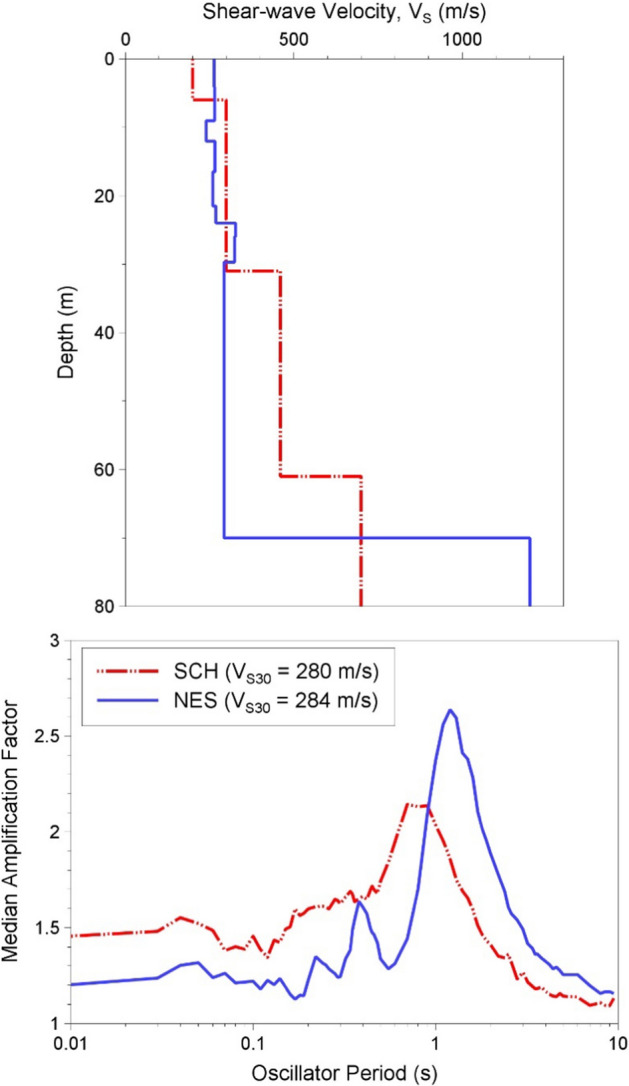
Upper: V_S_ profiles for the sandy SCH site and the clayey NES site, which have almost identical V_S30_ values; lower: median amplification factors for the two sites obtained from site response analyses (adapted from Papaspiliou et al. 2012)

The presence of layers of different stiffness in the near-surface site profile can have a profound effect on the surface motions, hence incorporating such local amplification effects is essential in any site-specific seismic hazard assessment. As noted in sub-Sect. 2.2.3, modern ground-motion prediction models always include a term for site amplification, usually expressed in terms of V_S30_. For an empirically constrained site amplification term, the frequency and amplitude characteristics of the V_S30_-dependence will correspond to an average site amplification of the recording sites contributing to the database from which the GMM was derived. The amplification factors for individual sites may differ appreciably from this average site effect as a result of different layering in the uppermost 30 m and to differences in the V_S_ profiles at greater depth (Fig. 46). For a site-specific PSHA, therefore, it would be difficult to defend reliance on the generic amplification factors in the GMM or GMMs adopted for the study, even if this also include additional parameters such as Z_1.0_ or Z_2.5_. Site amplification effects can be modelled using measured site profiles and this is the only component of a GMC model for which the collection of new data to provide better constraint and to reduce epistemic uncertainty does not depend on the occurrence of new earthquakes. Borehole and non-invasive techniques can be used to measure V_S_ profiles at the site and such measurements should be considered an indispensable part of any site-specific PSHA, as should site response analyses to determine the dynamic effect of the near-surface layers at the site.

#### PSHA and site response analyses

The last two decades have seen very significant developments in terms of how site amplification effects are incorporated into seismic hazard analyses. Previously, site response analyses were conducted for the uppermost part of the site profile, and the resulting amplification factors (AFs) applied deterministically to the hazard calculated at the horizon that defined the base of the site response analyses (SRA). A major step forward came when Bazzurro and Cornell (2004a, 2004b) developed a framework for probabilistic characterisation of the AFs and convolution of these probabilistic AFs with the PSHA results obtained at the rock horizon above which the SRA is applied.

**Fig. 47:**
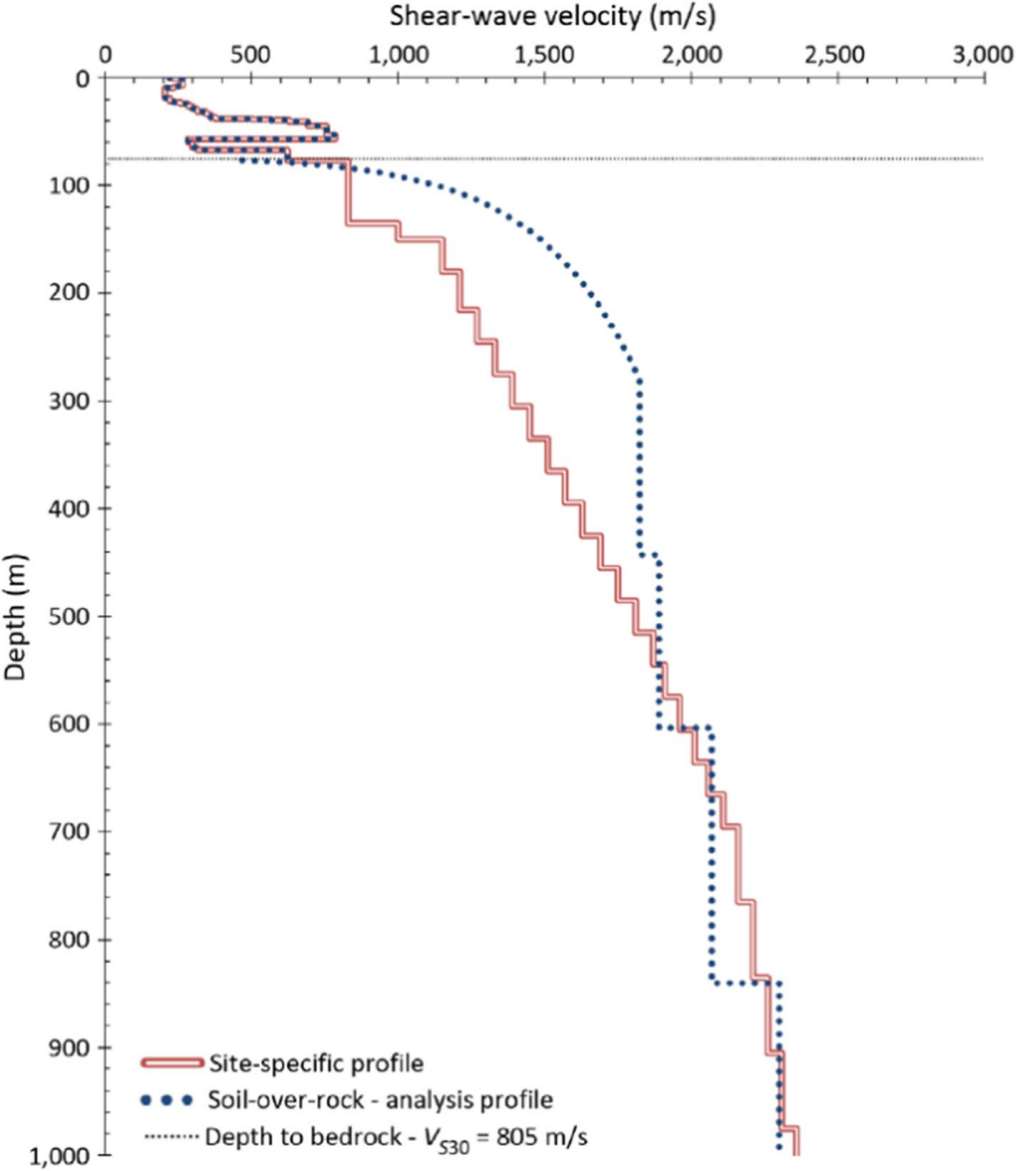
V_S_ profiles of underlying bedrock and overlying layers for which site response analysis is performed; the red line is the actual site profile, the dotted line the profile associated with the GMM (Williams and Abrahamson 2021)

**Fig. 48:**
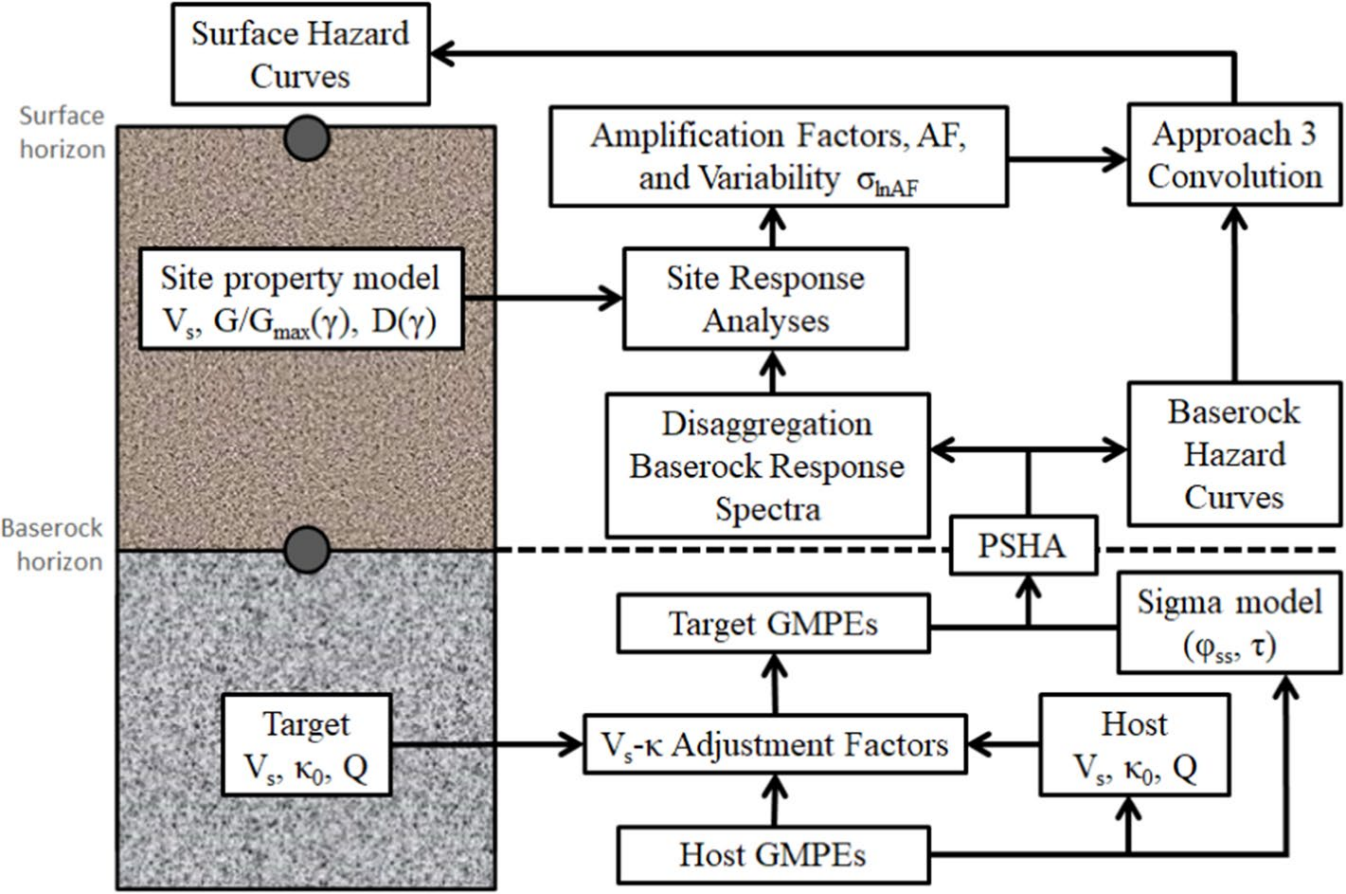
Scheme for applying host-to-target region adjustments to calculate rock hazard and then to convolve the rock hazard with AFs for the overlying layers (Rodriguez-Marek et al. 2014); G/G_max_ and D are the strain-dependent soil stiffness and damping, $\upgamma$ is the strain

An issue that was not always clearly recognised in this approach was the need to also capture correctly the AF associated with the V_S_ profile below the rock horizon at which the hazard is calculated and where the dynamic inputs to the site response calculations are defined. If the site-specific V_S_ profile is appreciably different from the profile implicit in the GMM used to predict the rock motions, there is an inconsistency for which an adjustment should be made (Williams and Abrahamson 2021; Fig. 47). In a number of site-specific PSHA studies, this has been addressed by making an adjustment for differences between both the GMM and target V_S_ profiles and between the damping associated with these profiles, in order to obtain the rock hazard, before convolving this with the AFs obtained from SRA for the overlying layers. Such host-to-target V_S_-$\kappa$ adjustments (e.g., Al Atik et al. 2014) became part of standard practice in site-specific PSHA studies, especially at nuclear sites (e.g., Biro and Renault 2012; PNNL 2014; Bommer et al. 2015b; Tromans et al. 2019). The scheme for including such adjustments to obtain hazard estimates calibrated to the target rock profile and then convolving the rock hazard with the AFs for overlying layers is illustrated in Fig. 48.

**Fig. 49:**
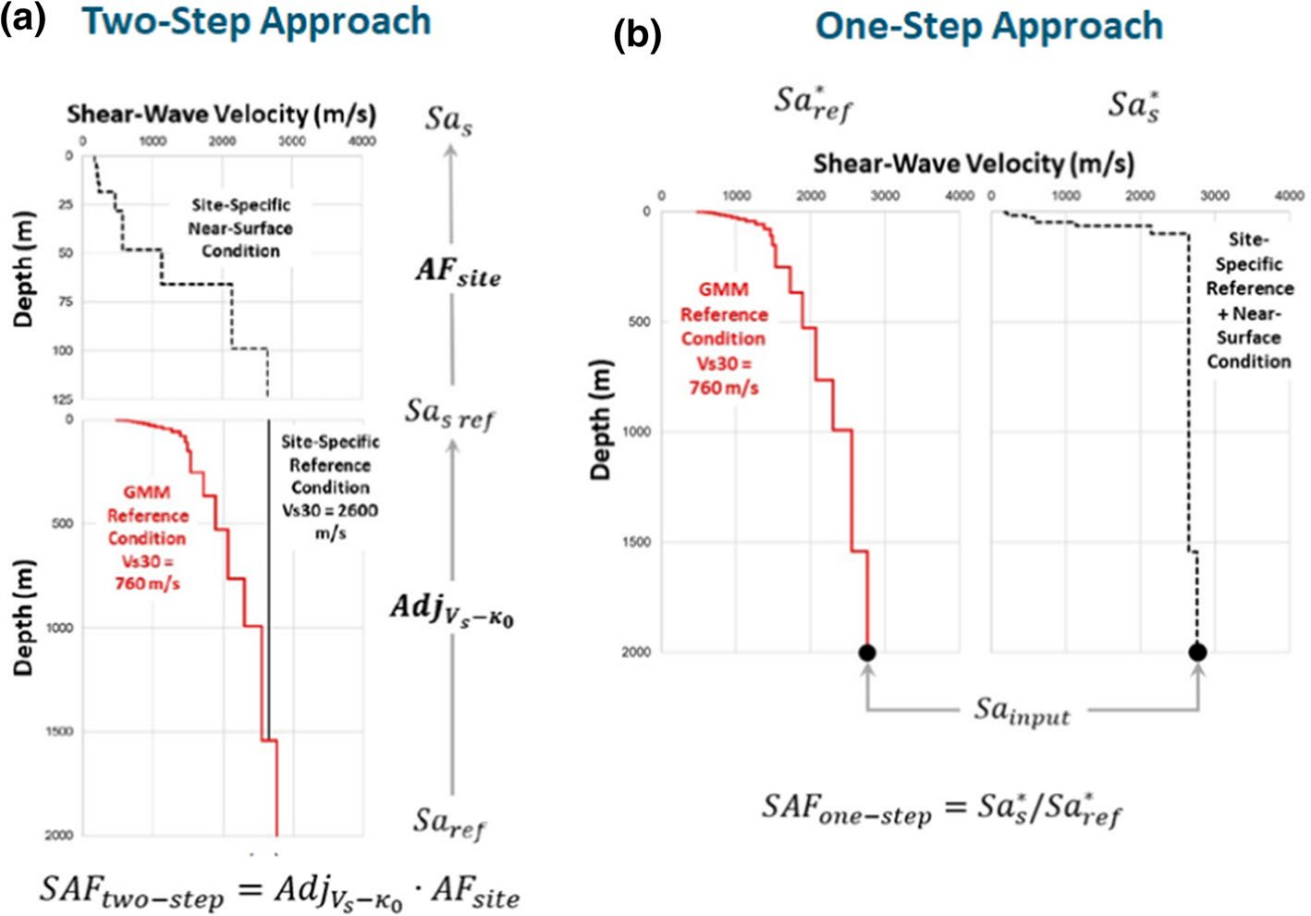
**a** Two-step site adjustment approach as in Fig. 48, and **b** one-step site adjustment; the subscript *s* refers to surface motions and the subscript ref to the reference rock profile (Rodriguez-Marek et al. 2021b)

The sequence of steps illustrated in Fig. 48 enables capture of the variability and uncertainty in both the rock hazard and site amplification factors, while also reflecting the characteristics of the full target site profile. However, there are practical challenges in the implementation of this approach, the first of which is that neither the GMC model for the baserock horizon nor the site response analyses for the overlying layers can be built until the baserock elevation is selected and characterised. Therefore, the development of the GMC model cannot begin until the site profile has been determined, possibly to considerable depth. Once the baserock is determined, then it is necessary to obtain estimates for the ${\kappa }_{0}$ parameter at a buried horizon, which is challenging unless there are recordings from borehole instruments at that horizon or from an accelerograph installed on an outcrop of the same rock (which even then may be more weathered than the buried rock horizon). Several studies have proposed empirical relationships between V_S30_ and ${\kappa }_{0}$ (Van Houtte et al. 2011; Edwards and Fäh 2013b; Laurendeau et al. 2013), but these tend to include very few values from very hard rock sites that would be analogous to many deeply buried rock profiles (Ktenidou and Abrahamson 2016). Consequently, there has been a move towards making the site adjustment in a single step rather in the two consecutive steps illustrated in Fig. 48. In the two-step approach, there is first an adjustment to the deeper part of the target site profile, through the V_S_-$\kappa$ correction, and then an adjustment to the upper part of the profile through the AFs obtained from SRA. In the one-step approach, the adjustment for the full profiles—extended down to a depth at which the host and target V_S_ values converge—is through ratios of AFs obtained from full resonance site response analyses of both profiles (Fig. 49); for the V_S_-$\kappa$ adjustments in the two-step approach, it is common to use quarter-wavelength methods (Joyner et al. 1981).

The one-step approach is not without its own challenges, including defining dynamic inputs at great depth. If the target profile is also hard rock and only linear SRA is to be conducted, the inputs can be obtained from stochastic simulations for scenarios identified from disaggregation of preliminary hazard analyses. Alternatively, surface motions at the reference rock profile can be generated from the GMM, since the profile is consistent with the model, and then deconvolved to the base of the profile to define the input to the target profile. The sensitivity to the input motions is likely to be less pronounced that in the two-step case since the site adjustment factors applied are the ratio of the AFs of the host and target profiles. The approach does, however, bring several advantages, including the fact that the reference rock model and the site adjustment factors can be developed in parallel and independently. If the convolution approach—often referred to as Approach 3, as in Fig. 48, after the classification of methods by McGuire et al. (2021)—is used, then the entire PSHA for the reference rock profile can be conducted independently of the target site characterisation. The GMC logic-tree is constructed by applying host-to-target region source and path adjustments to the backbone GMM, creating a logic tree that predicts motions calibrated to the target region but still for the reference rock profile associated with the GMM. The reference rock hazard therefore does not correspond to a real situation, but this reference rock hazard can then be easily transformed to surface hazard at any target profile. This can be enormously beneficial when hazard estimates are required at several locations with a region, as discussed further in sub-Sect. 6.5.

As an alternative to performing a convolution of the reference rock hazard with site adjustment factors, it is also possible to embed the adjustment factors directly in the hazard integral. This approach is computationally more demanding but can be advantageous when the site adjustment factors depend on the amplitude of the rock motions, for the case of non-linear site response, or depend on magnitude and distance, as has been found to be the case for short-period linear site amplification factors for soft sites (Stafford et al. 2017). The fractiles of the surface hazard are also obtained more accurately with this direct integration approach.

#### Epistemic uncertainty in site response analyses

**Fig. 50:**
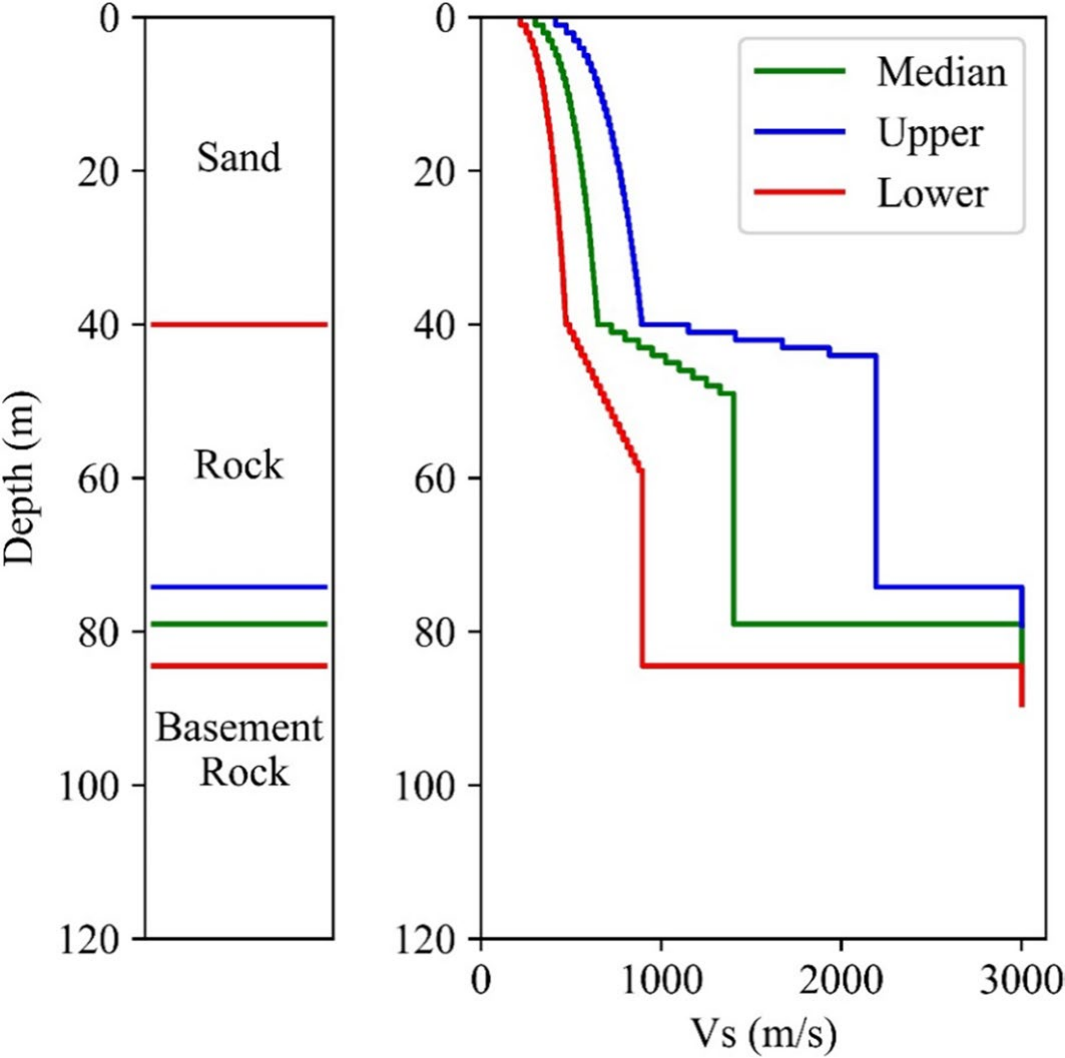
Stratigraphic profile for a hypothetical site (left) and V_S_ profiles (right) representing the range of epistemic uncertainty (Rodriguez-Marek et al. 2021a)

**Fig. 51:**
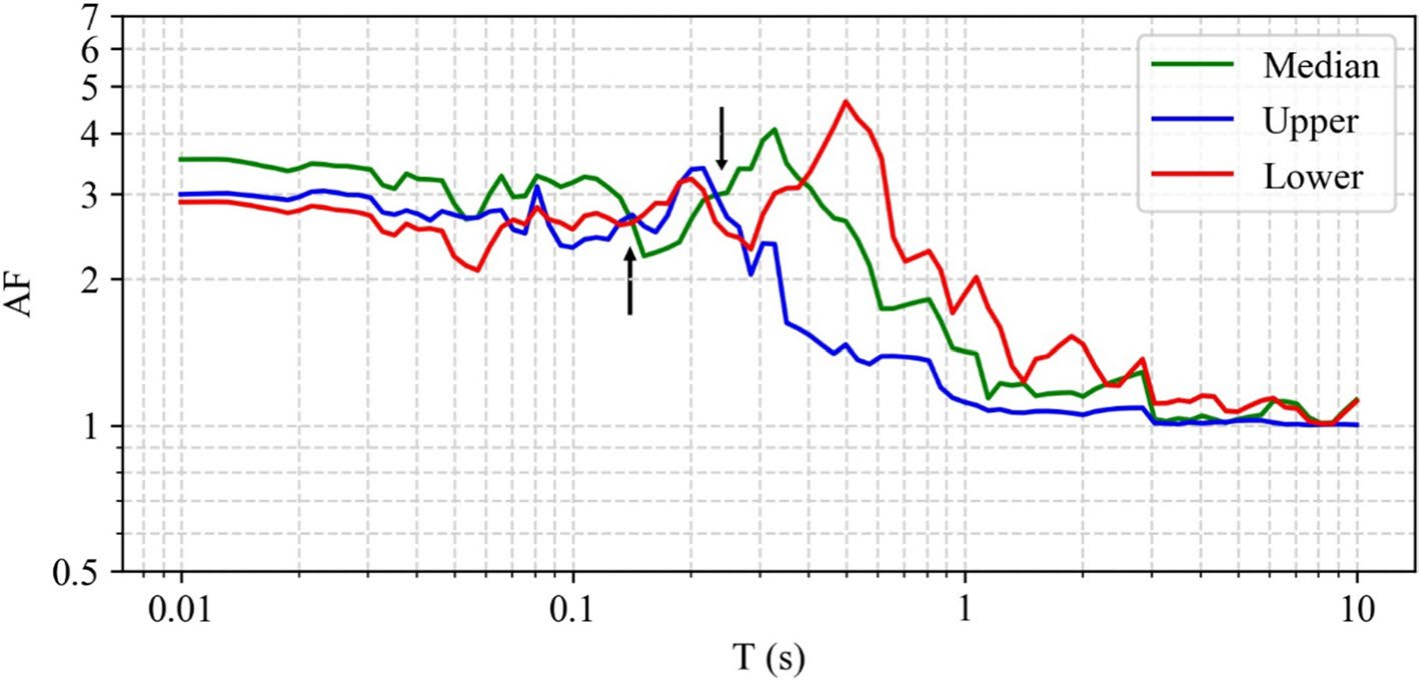
Amplification factors for the three V_S_ profiles in Fig. 50; the arrows indicate oscillator periods at which the three functions converge, suggesting that there is no epistemic uncertainty (Rodriguez-Marek et al. 2021a)

The basic components of an SRA model are profiles of V_S_, mass density, and damping, and for non-linear or equivalent linear analyses, modulus reduction and damping (MRD) curves that describe the decrease of stiffness and increase of damping with increasing shear strain in the soil. Uncertainty is usually modelled in the V_S_ profile, as a minimum. Common practice for a long time was to define the V_S_ profile and associated measure of its uncertainty defined as standard deviation of *ln*(V_S_). Profiles were then generated to by randomly sampling from the distribution defined by this standard deviation, superimposing a layer-to-layer correlation structure; the profiles could also include randomisations of the layer thicknesses and also the MRD curves. This procedure, however, treated all of the uncertainty in the site profiles as aleatory variability whereas in fact at least part of this uncertainty is epistemic. Consequently, there has been a move towards adopting logic trees for SRA, a common procedure being to define the best estimate profile and upper and lower alternatives, inferred from * in situ* measurements (Fig. 50). EPRI (2013a) provides guidance on appropriate ranges to be covered by the upper and lower bounds as a function of degree of site information that is available. Assigning weights to V_S_ profiles in a logic tree, however, is in many ways directly akin to assigning weights to alternative GMMs in a GMC logic tree, and the same pitfalls are often encountered. Figure 51 shows the AFs obtained from the three V_S_ profiles in Fig. 50, from which it can appreciated that at some oscillator frequencies, the three curves converge, suggesting, unintentionally, that there is no epistemic uncertainty in the site amplification at these frequencies. This is the same issue depicted in Fig. 40 and results from constructing a logic tree that does not allow easy visualisation of the resulting distribution of the quantity of interest, in this case the AFs at different frequencies. These observations have prompted the development of what could be considered a ‘backbone’ approach to SRA, although it is implemented rather differently.

**Fig. 52:**
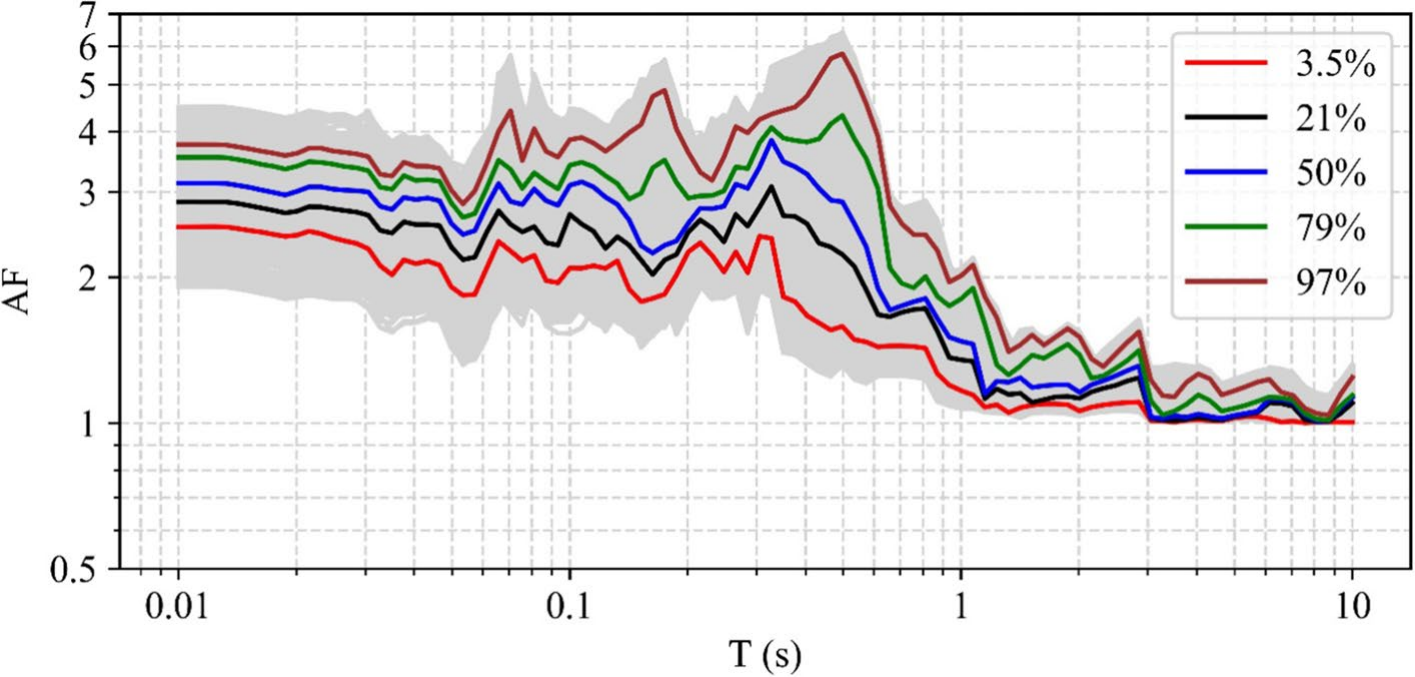
AFs obtained using multiple branch combinations from a complete logic tree for the site profiles and properties (grey curves) and the final AFs obtained by re-sampling this distribution (coloured curves), which correspond to the percentiles indicated in the legend and which are associated with the following weights: 0.101, 0.244, 0.31, 0.244, 0.101 (Rodriguez-Marek et al. 2021a)

The approach proposed by Rodriguez-Marek et al. (2021a) is to build a complete logic tree with nodes for each of the factors that influence the site response, such as the soil V_S_ profile, the bedrock V_S_, the depth of the weathered layer at the top of rock, and the low-strain damping in the soil. Site response analyses are then performed for all combinations of branches, which can imply an appreciable computational burden. The output will be a large number of weighted AFs, which are then re-sampled at each oscillator frequency, using a procedure such as that proposed by Miller and Rice (1983) to obtain an equivalent discrete distribution (Fig. 52).

The computational demand of the required SRA calculations in this approach is significant, although sensitivity analyses can be performed to identify nodes that have little effect on the results, which can then be dropped, and by using simplified schemes to map the influence of the variability in some elements of the model into the distribution directly (e.g., Bahrampouri et al. 2019).

Most SRA is performed assuming 1D vertical propagation of the seismic waves, which is a reasonable assumption given that at most sites V_S_ values reduce with depth (leading to refraction of the waves into increasingly vertical paths), but it is also an idealised approximation. For oscillator periods much longer than the fundamental period of the site, 1D SRA methods will tend to yield AFs close to unity in all cases. The method proposed allows a minimum level of epistemic uncertainty, reflecting the modelling error, to be imposed, in order to avoid underestimation of the epistemic uncertainty at longer periods.

### Seismic source models

In terms of their outputs that drive seismic hazard estimates, GMC and site response logic trees both define a single variable: at a given oscillator period, for a reference rock GMC model, it is the response spectral acceleration, and for the site adjustment logic tree, it is the relative amplification factor. For the case of SSC models, the outputs that directly influence the hazard estimates are many: the locations and depths of future earthquakes (which determines the source-to-site distance), the rates of earthquakes of different magnitude, the largest possible magnitude (Mmax), the style-of-faulting, and the orientation of fault ruptures. Distinguishing between elements of aleatory variability (which should be included directly in the hazard integrations) and elements of epistemic uncertainty (that are included in the logic tree) is generally quite straightforward for most components of SSC models: for a given source zonation, locations are an aleatory variable, whereas alternative zonations occupy branches of the logic tree; similarly, the hazard calculations integrate over the distribution of focal depths, but alternative depth distributions are included as a node in the logic tree.

In the following sub-sections I discuss the construction of elements of an SSC model from the same perspective as the preceding discussions of models for rock motions and site amplification factors: how can the best estimate model be constrained, and how can the associated epistemic uncertainty be most clearly represented. I make no attempt to provide a comprehensive guide to SSC model development, which, as noted previously, would require the full length of this paper (and would be better written by others who specialise specifically in this area). Rather I offer a few insights obtained from my experience in site-specific PSHA projects, and I also point the reader to references that define what I would consider to be very good current practice.

#### Finding faults

Since all earthquakes—with the exception of some volcanic tremors and very deep earthquakes in subduction zones—are the result of fault rupture, an SSC model would ideally consist only of clearly mapped fault sources, each defined by the geometry of the fault plane, the average slip rate, and the characteristic earthquake magnitude. While we know that this is practically impossible, every effort should be made to locate and characterise seismogenic faults whenever possible. In the Eighth Mallet-Milne lecture, James Jackson counselled that to make robust estimates of earthquake hazard and risk one should “*know your faults*” (Jackson 2001). Jackson (2001) provides an excellent overview of how faults develop and rupture, and how to interpret their influence on landscapes, as well as technological advances—in particular satellite-based InSAR techniques—that have advanced the ability to detect active faults. Most of the examples in Jackson (2001) are from relatively arid regions, particularly in the Mediterranean and Middle East regions. There are other environments in which detection of faults, even if these break the surface in strong earthquakes, can be much more challenging, particularly in densely vegetated tropical regions. For example, the fault associated with the earthquake in Mozambique in 2006 (Fig. 8), which produced a rupture with a maximum surface offset of ~ 2 m, was previously unknown. The earthquake occurred in an active flood plain overlain by thick layers of young alluvial deposits and there was nothing in the landscape to indicate the presence on a major seismogenic fault (Fenton and Bommer 2006).

**Fig. 53:**
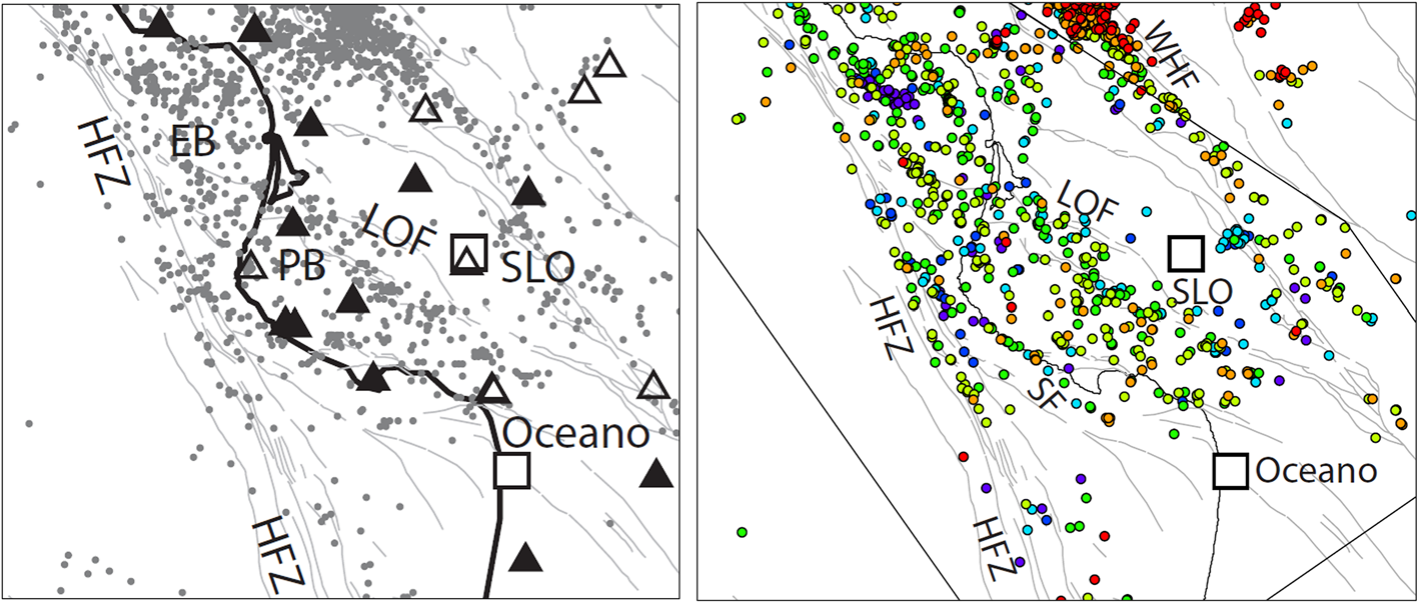
Seismicity in central California from the USGS catalogue (left) and after relocations using a new region-specific crustal velocity model (Hardebeck 2010). The triangles are seismograph stations (SLO is San Luis Obispo); the DCPP is located where there are two overlapping black triangles; HFZ is the Hosgri fault zone, SF is the newly identified Shoreline Fault (Hardebeck 2010)

**Fig. 54:**
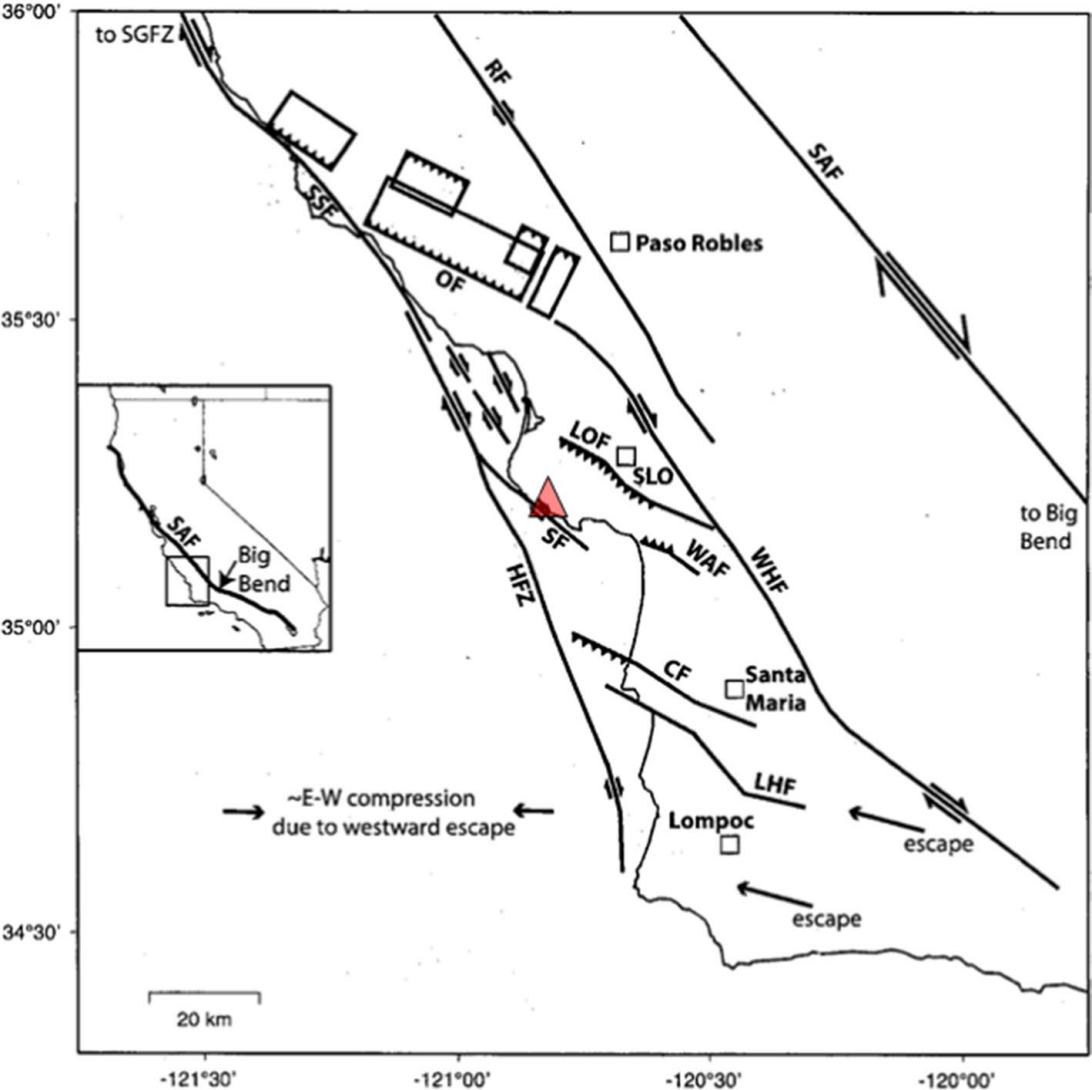
Faults in central California, including the Hosgri fault (HFZ) which defined the seismic design basis for the DCPP (red triangle) and the Shoreline fault (SF) (modified from Hardebeck 2010)

Another interesting example of a fault that was difficult to find was revealed through extensive studies undertaken for the Diablo Canyon NPP (DCPP) on the coast of California. I served for several years on the Seismic Advisory Board for the DCPP, for which the license conditions imposed by the US Nuclear Regulatory Commission (USNRC) included long-term studies to improve the knowledge of the seismicity and geology of the region surrounding the site, and to re-evaluate both the site hazard and the consequent seismic risk in the light of the new information obtained. The location of the DCPP, near San Luis Obispo, on the coast of central California, is in a region that had been studied far less than areas to the north and south, which had been the focus of extensive research by the University of California at Berkeley and UCLA, respectively. The operator of the DCPP, Pacific Gas and Electricity (PG&E), funded major research efforts in central California, many of them through the US Geological Survey (USGS), including installation of new seismograph networks, re-location of earthquake hypocentres, and extensive geophysical surveys. I distinctly recall working with Norm Abrahamson (on another project) in San Francisco one day when PG&E seismologist Marcia McLaren walked in to show Dr Abrahamson a plot of earthquake epicentres, obtained with a new crustal velocity model and advanced location procedures that consider multiple events simultaneously, which appeared to form a straight line adjacent to the shoreline, about 600 m from the NPP (Fig. 53). The revelation caused some consternation initially because there was no mapped fault at this location, the seismic design basis for the DCPP being controlled mainly by the scenario of a magnitude **M** 7.2 earthquake on the Hosgri fault, located about 4.5 km from the power plant (Fig. 54); consistent with other NPPs licensed in the USA in the same era, the design basis was deterministic.

**Fig. 55:**
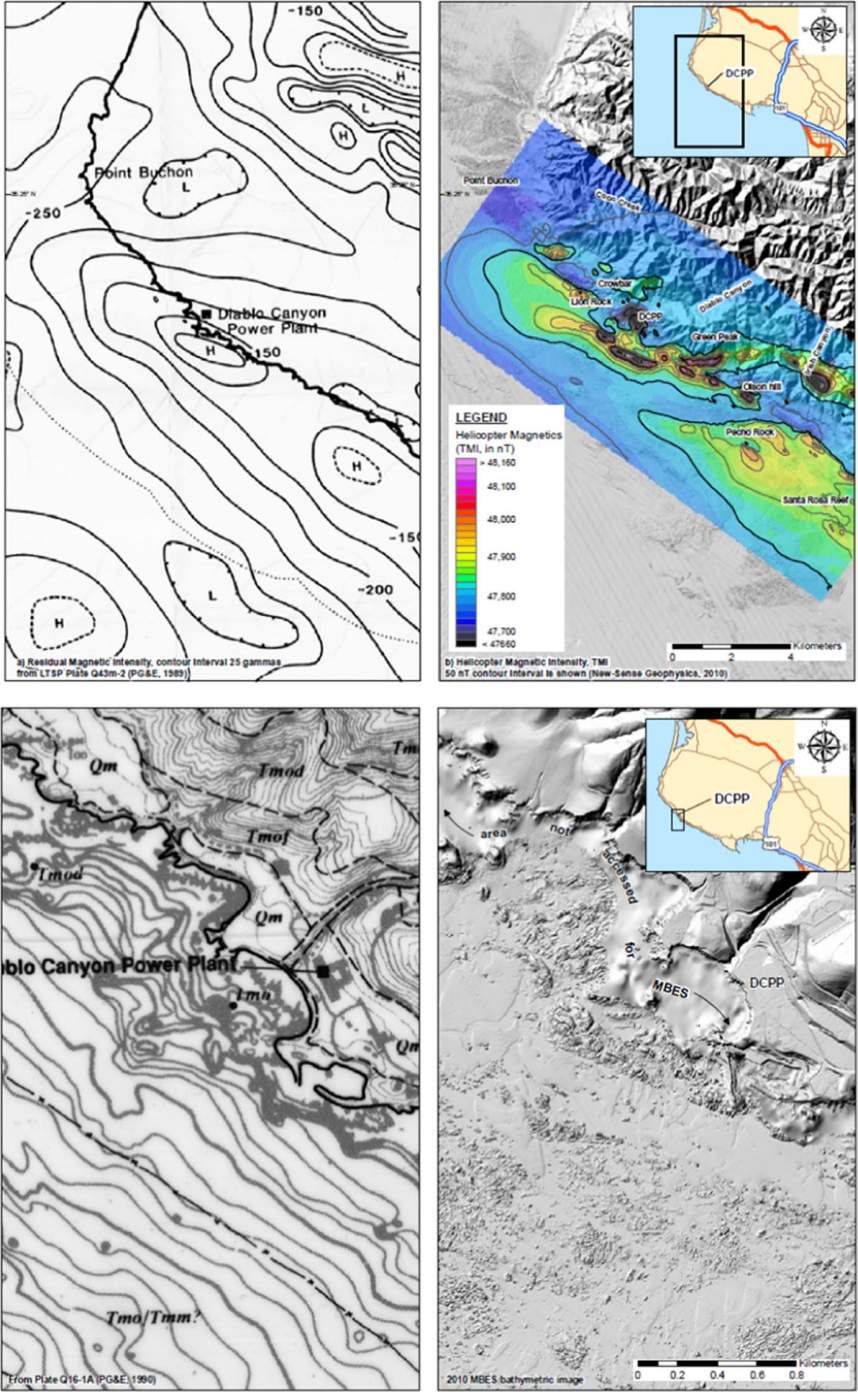
Contrasting geophysical measurements in the vicinity of the DCPP from 1989/1990 (left) and 2009 (right); upper: helicopter magnetics, lower: bathymetry (PG&E 2011)

Identification of seismogenic faults through locations of small-magnitude earthquakes is actually rather unusual in practice, but this case showed the potential of very accurate hypocentre location techniques. The presence of a right-lateral strike-slip fault along the coastline, given the name of Shoreline Fault, was confirmed by fault plane solutions (aka ‘beachballs’) showing a consistent orientation and slip direction. The reason that the extensive geophysical surveys had not identified the Shoreline Fault is its location within the shallow surf zones and the resolution of geophysical measurements originally made in the late 1980s. High-resolution magnetic and bathymetric surveys undertaken subsequent to the discovery of the aligned epicentres confirmed the clear presence of this structure (Fig. 55). The Shoreline Fault itself is not a very large structure but a scenario was presented wherein a major earthquake on the Hosgri fault would continue along the Shoreline fault, situating an event as large as **M** 7.5 a few hundred metres from the plant (Hardebeck 2013). Subsequent studies showed the Shoreline Fault to have a very low slip rate and that it did not present heightened risk to the plant (the design basis response spectrum for the DCPP was anchored at a PGA of 0.75* g*).

The characteristic model for earthquake recurrence on faults combines large magnitude quasi-periodic events with smaller events that follow a Gutenberg–Richter recurrence relationship (Youngs and Coppersmith 1985; see the middle right-hand panel of Fig. 23). There are other cases, however, where there is little or no earthquake activity of smaller magnitude between the large-magnitude characteristic earthquakes, sometimes referred to as an Mmax model (Wesnousky 1986). In such cases, especially if a fault is late in its seismic cycle and the last major event pre-dated any reliable earthquake records, seismicity data will be of little value in identifying active faults. A clear example of this is the Pedro Miguel fault in central Panama, which was discovered through geological investigations undertaken as part of the expansion programme to build the new post-Panamax locks that began operation in 2016; I was privileged to witness this work as it unfolded as a member of the Seismic Advisory Board for the Panama Canal Authority (ACP).

**Fig. 56:**
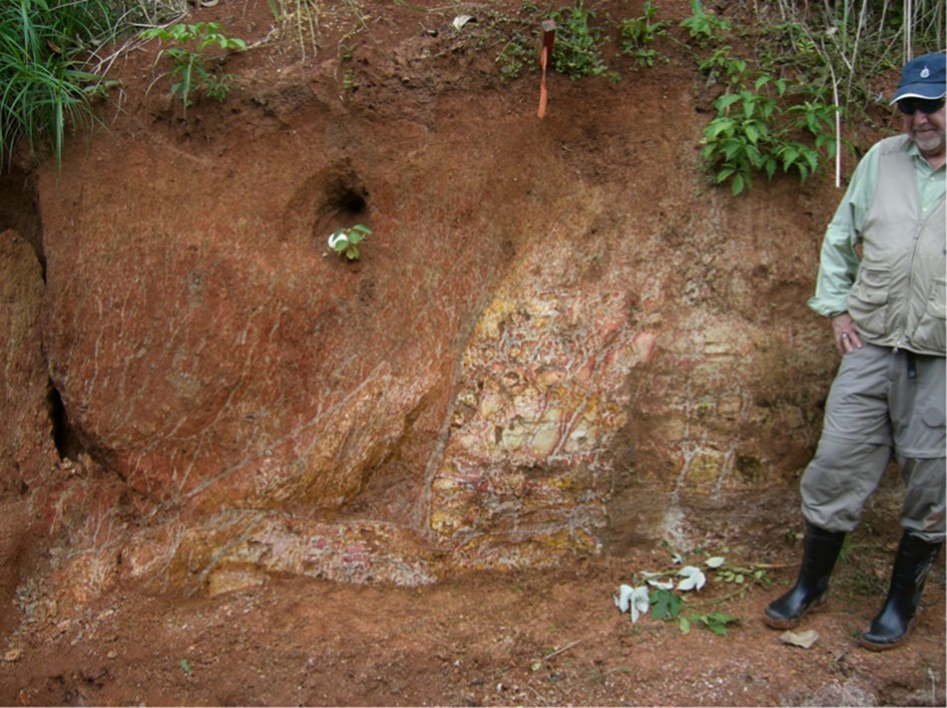
Exposure of the Pedro Miguel fault in a trench in central Panama

The work undertaken for the ACP identified several large strike-slip faults in central Panama, the most important of which turned out to be the Pedro Miguel fault, which runs approximately north–south and in very close proximity to the new Pacific locks. The fault was identified initially from surface offsets of streams and other geomorphological expressions, followed by an extensive programme of trenching (Fig. 56). The evidence all pointed consistently to a long, strike-slip fault that had last undergone major right-lateral slip a few hundred years ago, with evidence for earlier movements of comparable size. Here an interesting side note is in order: when the first trenches were opened and logged, there was some discussion of whether some observed fault displacements had occurred as the result of two large earthquakes at different times or one very large earthquake. Although the latter scenario may appear to be the more extreme scenario, it would actually result in lower hazard than the former interpretation, which may seem counter intuitive to some. The single large earthquake would have very long recurrence interval, whereas the somewhat smaller (but still very substantial) earthquakes imply a higher recurrence rate. Due to the non-linear scaling of ground motions with magnitude (Figs. 21 and 44), the larger magnitude of the less frequent characteristic earthquake would not compensate for the longer recurrence interval, hence in PSHA calculations, higher hazard results from the interpretation of the displacements being due to multiple events.

**Fig. 57:**
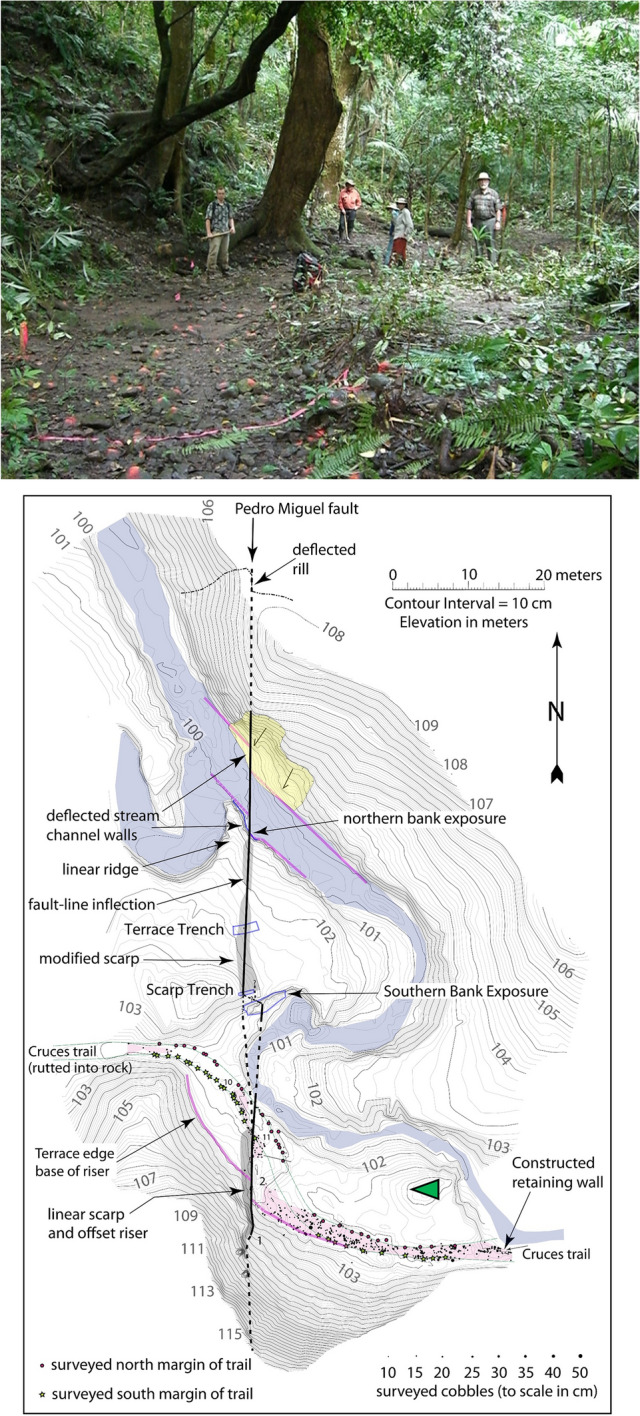
Upper: photograph of Camino de Cruces, in which the author (left) and previous Mallet-Milne lecturer Lloyd Cluff (right) are either side of offset; lower: map of the Pedro Miguel fault where it offsets the Camino de Cruces and adjacent stream banks; green triangle indicates approximate position and direction of photo (modified from Rockwell et al. 2010a)

After the geomorphological studies and paleoseismological investigations in the trenches had revealed the clear presence of an active fault with relatively recent movements, an additional discovery was made that provided compelling evidence both for the presence of the fault and the date of its most recent movement. The *Camino de Cruces* was a cobblestone road, built in 1527, that extended from the Pacific coast of Panama almost half-way across the isthmus to the source of the Chagres River. During the sixteenth and seventeenth centuries, the Spanish *conquistadores* transported gold, silver, spices and textiles plundered from South America to Panama via ship. The precious cargo was then transported by mule along the *Camino de Cruces* and then by boat along the Chagres to join ships on the Caribbean coast that would sail the booty to Europe. Exploration of the *Camino de Cruces*, which is now embedded in the jungle and requires a few hours of hiking to be reached from the nearest road, revealed a 3 m offset of the cobblestones, which aligned perfectly with the orientation and slip direction of the Pedro Miguel fault identified from the trenches (Fig. 57). Adjacent stream banks were also displaced by the same amount. Historically, the few damaging earthquakes known to have occurred in Panama were assigned to sources in the ocean to the north or south of the isthmus, which are zones of active tectonic deformation. An earthquake in 1621 was reported to have caused damage, particularly to the old Panama City (located to the east of today’s capital) and had been located by different researchers in both the northern and southern offshore deformation zones. However, through careful re-evaluation of the historical accounts of the earthquake effects, Víquez and Camacho (1994) had concluded that the 1621 earthquake was located on land, probably in close proximity to *Panamá Vieja*. This led to the conclusion that the 1621 earthquake had occurred on the Pedro Miguel fault, an earthquake of magnitude ~ 7 along the route of the Panama Canal. The implications of these findings, and the resistance these conclusions have encountered, are discussed further in Sect. 7.2.

The two examples above from California and Panama both correspond to cases of finding previously unknown faults, which will generally lead to increased hazard estimates. There are also many cases of geological investigations leading to reduced hazard estimates by demonstrating that a fault has a low slip rate and/or low seismogenic potential. Such studies will generally require a well-established geological framework for the region with clear dating of formations or features of the landscape. A good example is the GAM and PLET faults close to the Thyspunt NPP site in South Africa (Fig. 35), which were assigned probabilities of only 20% of being seismogenic on the basis of lack of displacements in well-defined marine terraces (Bommer et al. 2015b). The effect of assigning such a probability is to effectively reduce the recurrence rate of earthquakes on these structures by a factor of five.

**Fig. 58:**
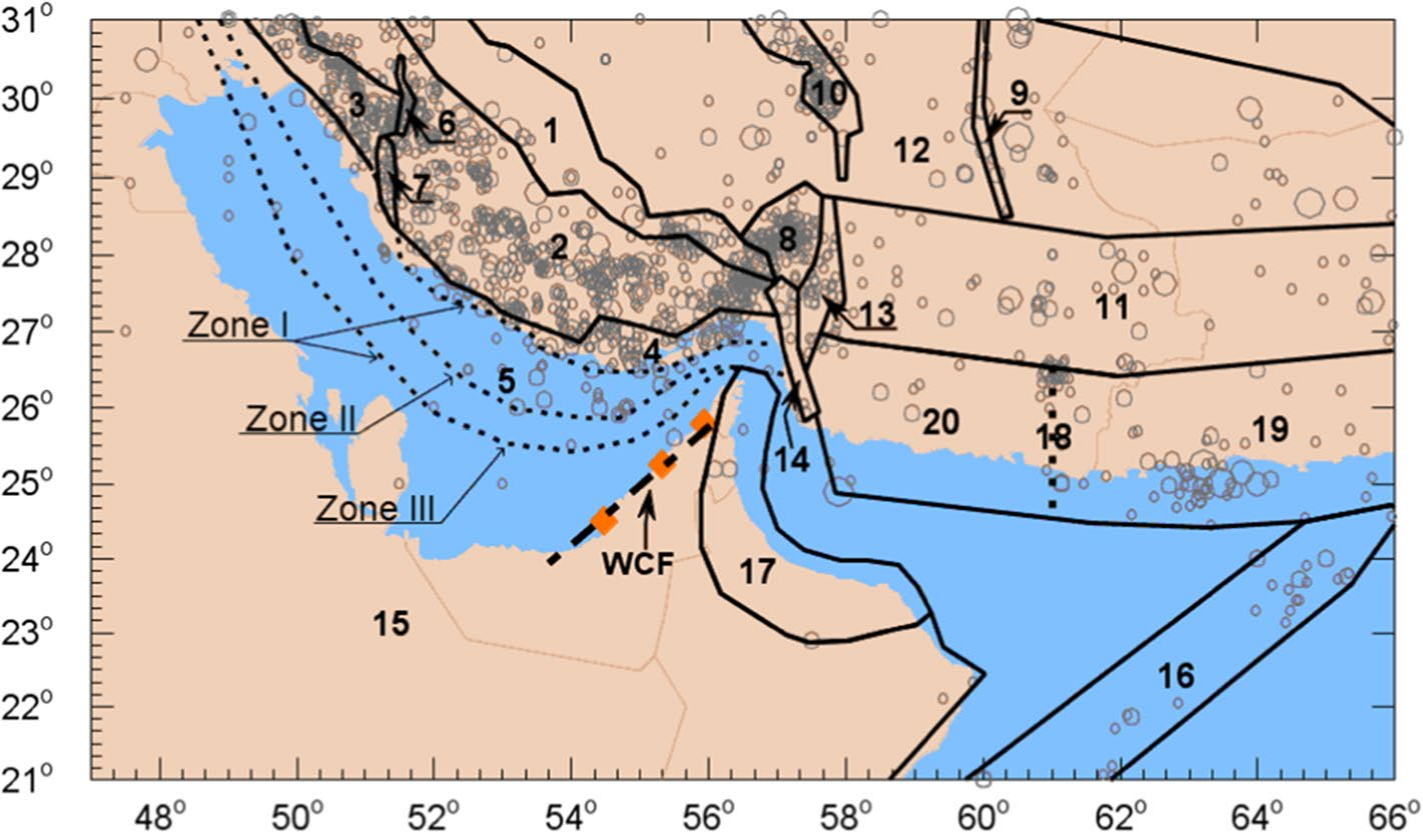
Seismic source zones defined for PSHA of Abu Dhabi, Dubai and Ra’s Al Khaymah (red diamonds, left to right) in the UAE (Aldama-Bustos et al. 2009); WCF is the West Coast Fault

Another example comes from the United Arab Emirates, for which we undertook a PSHA prompted by requests for input to numerous engineering projects in Dubai and Abu Dhabi (Aldama-Bustos et al. 2009). Our results closely agreed with other studies for the region, such as Peiris et al. (2006), but the 2475-year hazard estimates of Sigbjornsson and Elnashai (2006) for Dubai were very significantly higher. The distinguishing feature of the latter study is the inclusion of the West Coast Fault (WCF) as an active seismic source (Fig. 58). The seismic hazard studies that include the WCF as an active seismic source have generally done so based on the Tectonic Map of Saudi Arabia and Adjacent Areas by Johnson (1998), which drew heavily on the work of Brown (1972) which, according to Johnson (1998), presented ‘‘*selected tectonic elements of Saudi Arabia and, in lesser details, elements in adjacent parts of the Arabian Peninsula*’’. Among several publications on the geology of this region that we reviewed, only Hancock et al. (1984) refer to a fault along the coast of the Emirates, but their mapped trace is annotated with a question mark indicating doubts regarding its presence.

Assigning activity rates to the WCF is difficult due to the lack of any instrumental seismicity that could be directly associated with this structure, and the historical record for the UAE is almost null because of the very sparse population and the absence of major towns and cities where earthquake damage could have been recorded. To perform a sensitivity analysis, we assumed the fault to behave as a characteristic earthquake source and the slip rate was estimated indirectly from the maximum rate that could pass undetected based on the available information. To infer this limiting slip rate, we employed contours of the base of the Tertiary and the approximate base of the Mesozoic rocks that are overlain by sediments known as *sabkhas*; the latter are composed of sand, silt or clay covered by a crust of halite (salt), deposits that were formed by post-glacial flooding between 10 and 15 Ma ago, hence we conservatively assumed an age of 10 Ma. The Brown (1972) map is at a scale of 1:4,000,000 and it was assumed that any offset in the contours resulting from accumulated slip on the fault would be discernible if at least 1 mm in length on the map, implying a total slip of 4 km and a slip rate of 0.4 mm/year. Additional constraint on the slip rate was inferred from the GPS measurements obtained at two stations in Oman (Vernant et al. 2004); making the highly conservative assumption that all the relative displacement is accommodated on the WCF yields a slip rate of 2.06 mm/year, although in reality most of this displacement is actually owing to the rotational behaviour of the Arabian plate. We then assumed a characteristic earthquake magnitude of **M** 7 ± 0.5; the relationship of Wells and Coppersmith (1994) indicates **M** 8 if the entire fault ruptures, but such events would be difficult to reconcile with the lack of observed offset. With the slip rate of 0.4 mm/year, the hazard was re-calculated for Dubai: the inclusion of the WCF increased the hazard estimates but even for an AFE of 10^–6^, the increase in the ground-motion amplitude is less than a factor of two. To produce a 475-year PGA for Dubai that would match that obtained by Sigbjornsson and Elnashai (2006), a slip rate on the fault of 6.0 mm/year would be required.

**Fig. 59:**
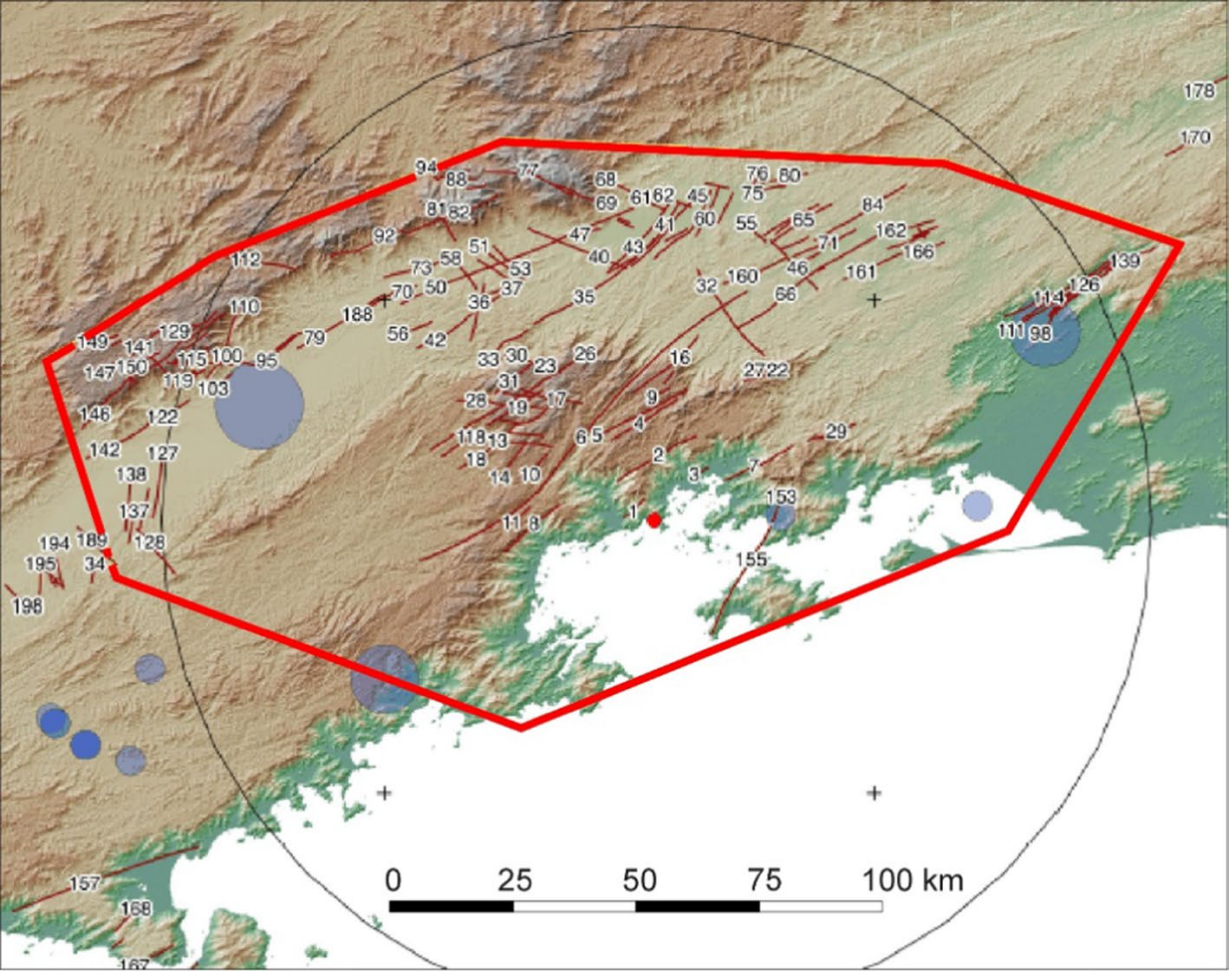
Mapped faults in the region surrounding the Angra dos Reis NPP site (red dot) in southeast Brazil; the red polygon is the equivalent source area defined to model the potential seismicity associated with these faults (Almeida et al. 2019)

In the case of WCF, constraints on the possible slip rate were obtained indirectly, whereas it is possible that field investigations might reveal that this lineament is not an active fault at all. An inescapable fact is that geological field work, especially when it involves trenching and laboratory dating of rock samples, is time consuming and can incur substantial costs, but for major infrastructure projects, the investment is fully justified. If geological field work is not undertaken to characterise known or suspected faults, then a price must be paid in terms of increased epistemic uncertainty. This principle was invoked in a site-specific PSHA for the Angra dos Reis NPP in southeast Brazil (Almeida et al. 2019). A number of faults have been mapped in the region of the site (Fig. 59) and for some of these structures, displacements are visible in exposures at road cuttings, which in itself points to possible seismogenic activity of these structures.

**Fig. 60:**
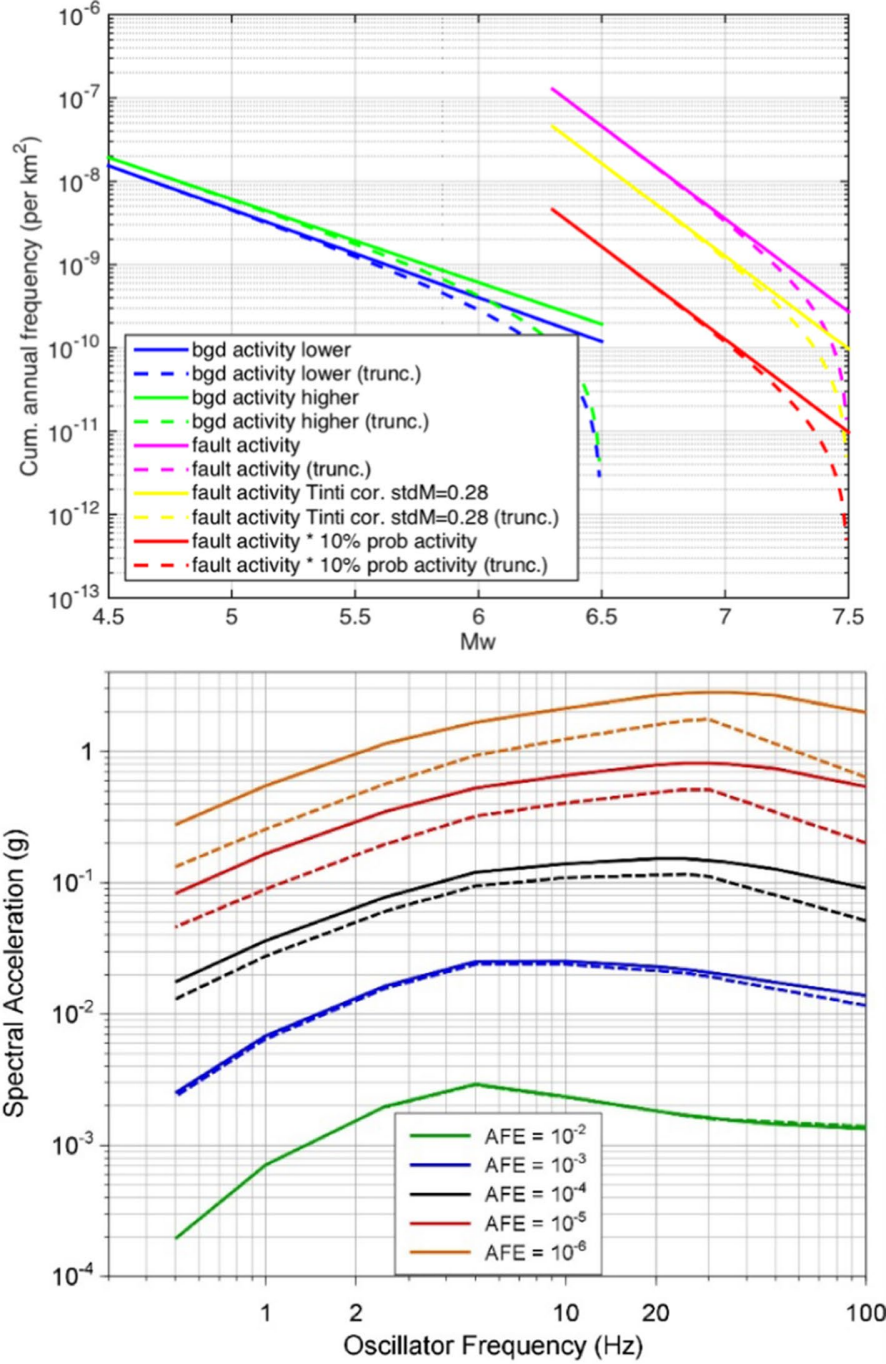
Upper: recurrence relationships for host source zone (blue and green) and for the equivalent source for potentially active faults (purple curve from the data, red curve is the effective recurrence after applying a 10% probability of the faults being seismogenic), defined for the Angra dos Reis PSHA; lower: uniform hazard response spectra for the Angra dos Reis NPP site in Brazil obtained without (dashed lines) and with (solid lines) the contributions from the potentially active faults (Almeida et al. 2019)

At the same time, the Quaternary sequence of the region is still in development and reliable geochronology data for the formations displaced by the local offsets are very limited to date. There is also a lack of clear and persistent geomorphological expression of most of the faults for which displacements have been logged. Rather than modelling all of these structures as individual sources, with logic-tree branches for uncertainty in their probability of being seismogenic, slip rates and characteristics magnitudes, their collective impact on the hazard was modelled through an equivalent source zone (red polygon in Fig. 59) imposed on top of the other area source zones defined for the PSHA. Each fault was assigned a slip rate, dependent on its length, which would not be inconsistent with the lack of strong expressions in the landscape, and a maximum magnitude inferred from its length. These parameters were then used to define magnitude-recurrence pairs that generated an equivalent catalogue of larger events, for which a recurrence model was derived (Fig. 60). This source was then added to the areal source zones and included in the hazard integrations with an M_min_ of 6.5 and an Mmax corresponding to the largest value assigned. This conservative approach led to appreciable increase in the hazard estimates at low AFEs (Fig. 60) but it provided a computationally efficient way of including the epistemic uncertainty associated with these faults. If the resulting site hazard were to have proved challenging for the safety case of the plant, geological and geochronological investigations could be commissioned to provide better constraint on the seismogenic potential of these faults, which would most likely lead to a reduction in their impact.

#### Source zones and zoneless models

**Fig. 61:**
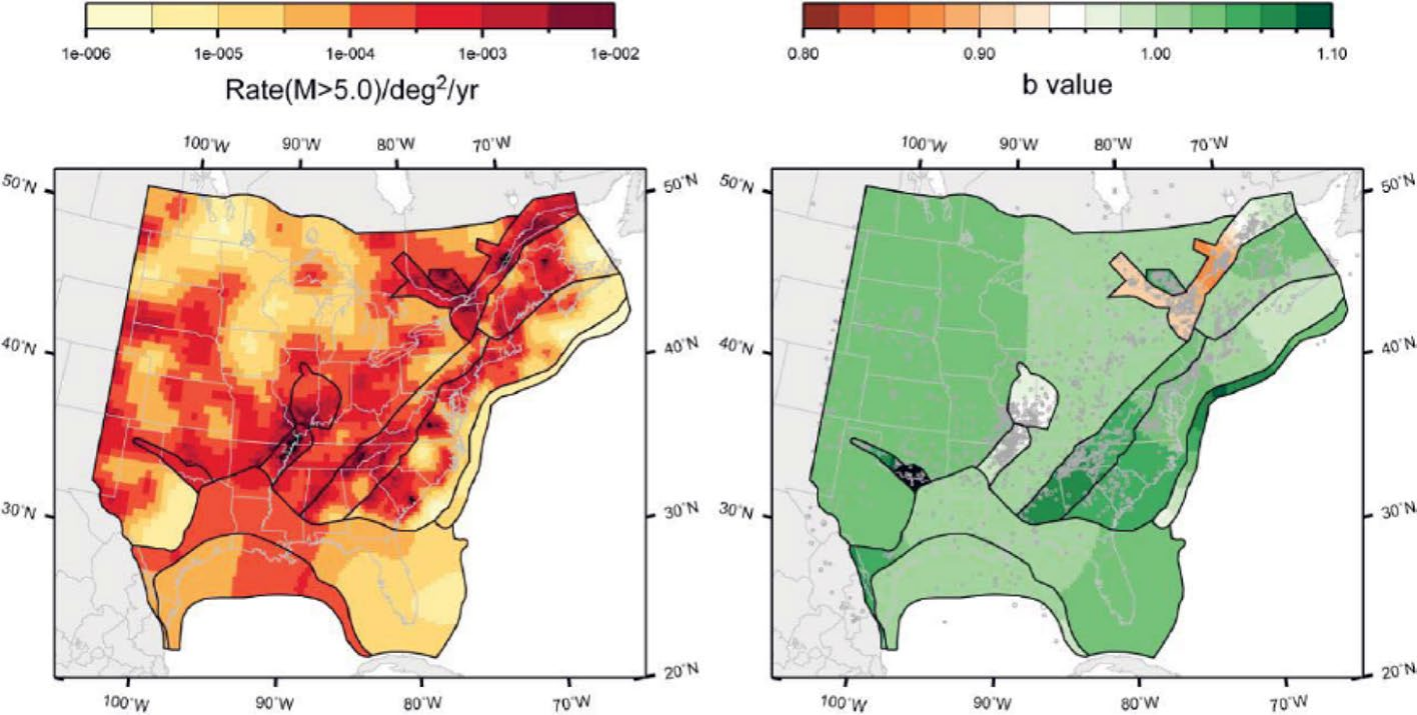
Spatially smoothed activity rates (left) and the* b*-value (right) within the broad source zones defined for the SSC model of the Central and Eastern United States (USNRC 2012a)

Since not all earthquakes can be assigned to mapped geological faults, seismic source zones are a ubiquitous feature of SSC models for PSHA. Source zones are generally defined as polygons, within which specified characteristics of the seismicity are assumed to be uniform. One of the common assumptions is that the seismicity is spatially uniform, and earthquakes can therefore occur at any location within the source zone with equal probability. This has often led to the suggestion (by reviewers) that the SSC logic tree should also include a branch for zoneless models, in which the locations of future seismicity are essentially based on epicentres in the earthquake catalogue for the region (e.g., Frankel 1995; Woo 1996). For a region in which the spatial distribution of seismicity is tightly clustered, the zoneless approaches are likely to yield distinctly different hazard distributions compared to hazard estimates obtained with source zones (e.g., Bommer et al. 1998). In my view, however, there should be no automatic imperative to include both source zones and zoneless approaches, because such an admonition places the focus in the construction of the SSC logic tree on selecting and weighting models rather than on the distributions of magnitude, distance and recurrence rate that drive the hazard. There is, in any case, a third option between zoneless approaches and areal source zones, namely zones with smoothed seismicity: source zones can be defined in which certain characteristics are uniform throughout (such as Mmax, style-of-faulting, and focal depth distributions) but with the *a*- and *b*-value of the Gutenberg-Richter recurrence relationship varying spatially (Fig. 61). The spatial smoothing is based on the earthquake catalogue but with the degree of smoothing controlled by user-defined parameters (which is also true of the zoneless approaches).

**Fig. 62:**
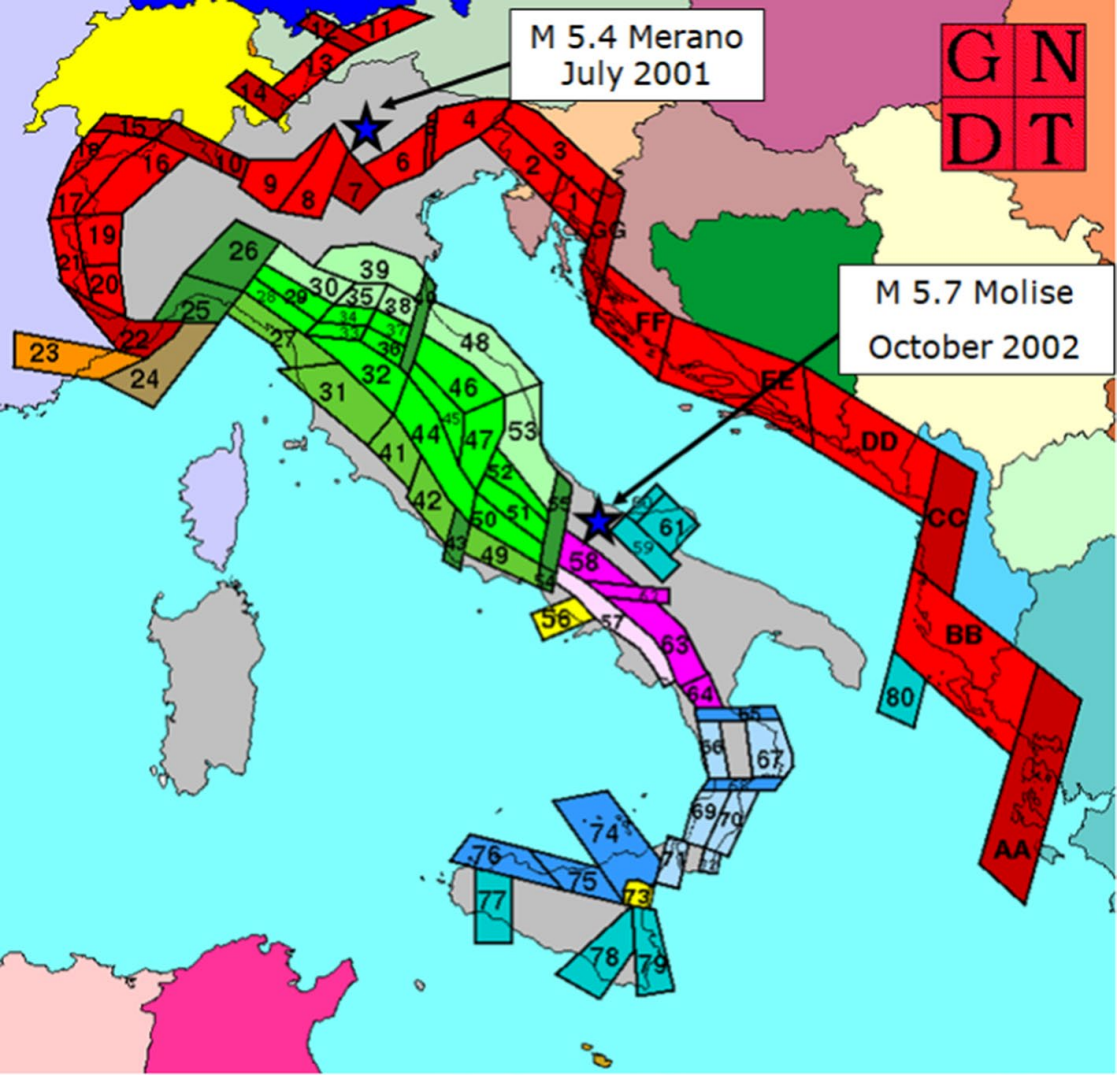
The 1996 seismic source zonation (ZS4; Meletti et al. 2000) underlying the seismic hazard map of Italy, showing locations of two destructive earthquakes that occurred outside the boundaries of the zones (adapted from figure in Meletti et al. 2008)

The questions being addressed in the construction of a seismic source zonation or a zoneless source modelling approach is the same: where will future earthquakes occur and what will be their characteristics in terms of Mmax, style-of-faulting and focal depth distribution? When these questions are not answered by the localising structures of active geological faults, the question then arises to what degree is the earthquake catalogue spatially complete? Or expressed another way, can the observed spatial distribution of seismicity be assumed to be stationary for the forthcoming decades covering the design life of the facility under consideration? Spatial completeness can be a particularly important issue in mapping of seismic hazard. In 2004, I served on a panel to review the development of a new seismic hazard map for Italy (Meletti et al. 2008), an endeavour that was triggered in large part by two earthquakes of **M** 5.7 earthquake of 31 October and 1 November 2002, which caused the collapse of a school building in San Giuliano and the deaths of 25 children. The earthquake occurred in an area classified as not requiring seismic design in the seismic design code of 1984. The earthquake was the second destructive earthquake to occur outside of the seismic source zones defined for the hazard mapping, following an **M** 5.4 in Merano in July 2001, which also led to loss of life (Fig. 62). The purpose of the new hazard map was to serve as the basis for a revised seismic design code (Montaldo et al. 2007; Stucchi et al. 2011) and also as the starting point for an endeavour to seismically retrofit school buildings at risk (e.g., Grant et al. 2007).

The definition of seismic source zones is often poorly justified in PSHA studies, with different criteria being invoked for different boundaries and evidence cited as a determining factor for one zone ignored in another. There can be no prescription for how source zones should be defined because the process will necessarily have to adapt to the specific characteristics and data availability in any given application. However, some simple guidelines can assist in creating a more transparent and defensible seismic source zonation, which is fundamental to achieving acceptance of the resulting hazard assessment. Firstly, the study should clearly explain the definition of a seismic source zone being adopted in the study, which needs to be more specific than a bland statement regarding uniform seismicity. The definition should list the earthquake characteristics that are common across a source zone, and those which are allowed to vary, whether through spatial smoothing (for recurrence parameters) or through aleatory distributions (for style-of-faulting, for example). Boundaries between source zones will then logically correspond to distinct changes in one or more of the common characteristics. Secondly, the criteria for defining boundaries should also be clearly specified, together with the data to be used in implementing each criterion. To the extent possible, evidence should be given that demonstrates the role of each criterion in controlling the location, size, and rate of seismicity, either in general or in the region where the study is being performed. These criteria should then be consistently and systematically applied to develop the source zonation model. A good example of both clear definition of source zone characteristics and the application of consistent criteria for their definition can be found in the SSC study for the Central and Eastern United States (CEUS-SSC) project (USNRC 2012a).

The discussion of criteria for defining source boundaries and using data to apply these criteria should not give the impression that the process, once defined, can be somehow automated. Inevitably, expert judgement plays a significant role, as discussed further in Sect. 6. The boundaries of seismic source zones are a clear example of epistemic uncertainty, and this is often reflected in the definition of multiple source zonation models with alternative boundaries, especially in site-specific studies for which the configuration of the host zone (containing the site) and its immediate neighbours can exert a strong influence on the hazard results.

As previously noted in Sect. 4.1, for compatibility with the distance metrics used in current GMMs, hazard calculations need to generate virtual fault ruptures within area source zones. The geometry of these virtual ruptures should reflect the geological structure and stress orientations in the region, and their dimensions should be related to the magnitude of the earthquake; for the latter, several empirical scaling relationships are available, including those of Stafford (2014), which were specifically derived for application in PSHA. Careful consideration needs to be given to the physical characteristics of these virtual ruptures, since they are not only a tool of convenience required because of the use of R_jb_ and R_rup_ in GMMs; the ruptures should correspond to physically realisable events. Rupture dimensions are often defined by the total rupture area and source models will generally define the thickness of the seismogenic layer of the crust; consequently, for the largest magnitudes considered, the length may be very considerable, exceeding the dimensions of the source zone within which the rupture initiates. This is usually accommodated by allowing the source zones to have ‘leaking boundaries’, which means that the ruptures can extend outside the limits of the source zone. This makes it even more important to clearly define the meaning of a source zone since in effect it implies the presence of seismogenic faults that may straddle two or more source zones, but rupture initiations are specified separately within each zone. Particular caution is needed if the host zone is relatively quiet and there are much higher seismicity rates in more remote sources, especially if the specified orientations allow virtual ruptures to propagate towards the site. In one project in which I participated, the preliminary hazard analyses showed major hazard contributions coming from a source zone whose closest boundary was a considerable distance from the site. Disaggregating the contributions from this source in isolation, it became apparent that the ruptures associated with the largest earthquakes in this source were almost reaching the site. The recommendation of Bommer and Montaldo-Falero (2020) to use only point-source representations rather than virtual ruptures in remote source zones eliminates this potential pitfall.

In some site-specific PSHAs that I have reviewed, very small seismic source zones are sometimes defined, usually to enclose a cluster of relatively high seismic activity. This becomes akin to a zoneless seismicity model or smoothed seismicity with limited spatial smoothing, which should be justified through a geologic or tectonic explanation for why higher seismic activity is localised in that area. Such technical justifications are particularly needed when the consequence of such small source zones is to maintain the observed seismicity at a certain distance from the site under study. Another issue that needs to be addressed with very small seismic source zones is that for many of the virtual ruptures, the majority of their length may lie outside the source boundaries. This could partially be addressed by assigning smaller Mmax values, but this would also need a robust and independent technical basis rather than simply being an expeditious measure to accommodate the decision to define a source zone of small area.

#### Recurrence rate estimates

The recurrence rates of moderate and large magnitude earthquakes in an SSC model are the basic driver of seismic hazard estimates. For a single seismic source zone, the hazard curve obtained at a site scales directly with the exponent of the activity rate (*a*-value) of the Gutenberg-Richter recurrence relationship. The rates of future earthquakes are generally inferred from the rates of past earthquakes, both for fault source and area sources, hence the reliability of the hazard assessment will depend on the data available to constrain the rate and the assessment of the associated uncertainty. Focusing on source zones rather than fault sources, the recurrence model relies on the earthquake catalogue for the region. As already noted in Sect. 3.3, instrumental monitoring of earthquakes has been operating for at most a few decades in many parts of the world, which is a very short period of observation to serve as a basis for establishing long-term rates. The catalogue can usually be extended through retrieval and interpretation of historical accounts of earthquake effects; the very first Mallet-Milne lecture by Nick Ambraseys was largely devoted to the historical seismicity of Turkey (Ambraseys 1988). This work revealed that the 20^th^ Century had been an unusual quiescent period for seismicity in southeast Turkey, for which reason the instrumental earthquake catalogue was a poor indicator of the long-term seismic hazard in the region, where several large earthquakes has occurred in the nineteenth Century and earlier (Ambraseys 1989).

As with geological investigations of faults, historical seismicity studies will often unearth previously unknown earthquakes that will impact significantly on hazard estimates, but in some cases such studies can serve to constrain low hazard estimates. In the PSHA for the Thyspunt nuclear site in South Africa (Bommer et al. 2015a, b), the hazard was largely controlled, at least at shorter oscillator periods, by the seismicity rates in the host ECC source zone (Fig. 35). The earthquake catalogue for this region was very sparse but investigations were undertaken that established that this was not the result of absence of evidence for seismic activity. By identifying the locations at which newspapers and other records were available over different historical periods and noting that these did include reports of other natural phenomena (Albini et al. 2014), the absence of seismic events was confirmed, thus corroborating the low recurrence rates inferred from the catalogue. Without this evidence for the absence of earthquake activity, broad uncertainty bands on the recurrence model would have been required, inevitably leading to increased seismic hazard estimates.

Developing an earthquake catalogue for PSHA involves retrieving and merging information from many sources, both instrument and historical, as often as possible using primary sources of information, and eliminating duplicated events. Listed events that are actually of anthropogenic origin, such as quarry blasts, must also be removed (e.g., Gulia and Gasperini 2021). The earthquake magnitudes must then be homogenised to a uniform scale, which is usually moment magnitude; as noted below, the variability in such empirical adjustments should be accounted for in the calculation of recurrence rates. Since PSHA assumes that all earthquakes are independent—in order to sum their hazard contributions—the homogenised catalogue is then declustered to remove foreshocks and aftershocks (e.g., Gardner and Knopoff 1974; Grünthal 1985; Reasenberg 1985).

To calculate recurrence rates, the number of earthquakes in each magnitude bin is divided by the time of observation, but this requires an estimate of the period for which the catalogue is complete, which will generally increase with magnitude. The estimation of completeness periods is a key source of epistemic uncertainty in the derivation of recurrence rates, but this uncertainty can be constrained by establishing probabilities of earthquake detection over different time periods based on the operational characteristics of seismograph networks and the availability of historical records. The uncertainty in magnitude values, whether the standard error of instrumentally determined estimates or the standard deviation in empirical relations to convert other magnitudes to moment magnitude (or to convert intensities for the case of historical events), should also be taken into account. These uncertainties are usually assumed to be symmetrical (normally distributed) but they lead to errors because of the exponential nature of earthquake recurrence statistics (i.e., because there are more earthquakes at smaller magnitudes). The effect of this uncertainty is to alter the activity rate—upwards or downwards—but it does not alter the *b*-value (Musson 2012b); however, if the magnitude uncertainties are not constant, which will often be the case, then the *b*-value is also affected (Rhoades 1996). Tinti and Mulargia (1985) proposed a method to adjust the magnitude values to correct for this uncertainty; in the CEUS-SSC project, Bob Youngs developed an alternative approach that adjusts the effective rates (USNRC 2012a).

**Fig. 63:**
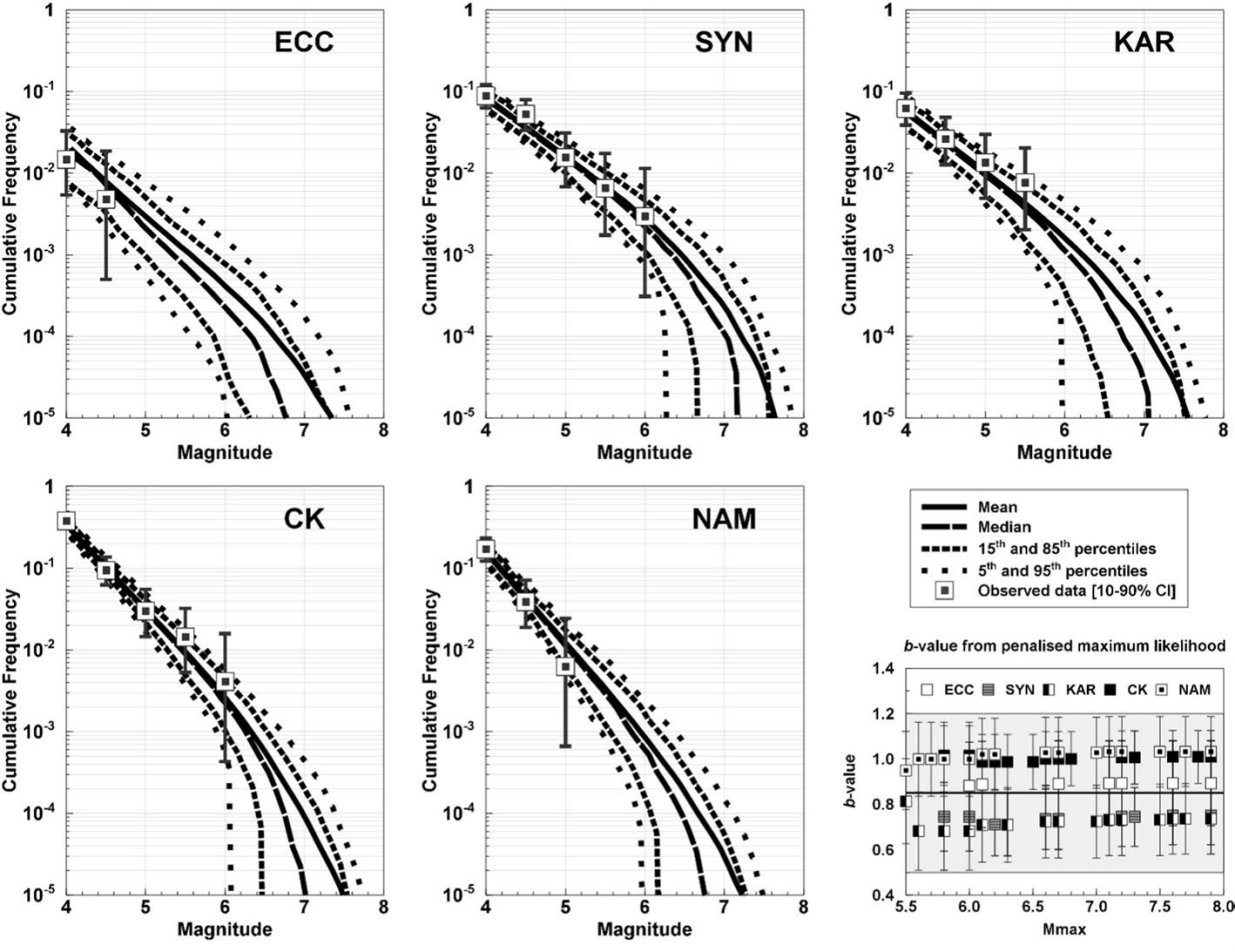
Fitting of recurrence relationships to catalogue data for the five area source zones defined for the Thyspunt site (Fig. 35) using the penalised maximum likelihood approach (Bommer et al. 2015b); the panel at the lower right-hand side shows the* b*-values determined for each source zone using the prior distribution based on the regional* b*-value (grey shading)

As was noted previously (Sect. 3.1), once the recurrence data are prepared, the parameters of the Gutenberg-Richter relationship should be obtained using a maximum likelihood approach (e.g., Weichert 1980). Veneziano and Van Dyke (1985) extended this approach into a penalised maximum likelihood method, in which the *b*-values are conditioned on the estimates of Mmax and also constrained by a prior estimate for the *b*-value, which is useful where data are sparse. Figure 63 shows the fitting of recurrence relationships to the data for the five source zones defined for the Thyspunt PSHA using the penalised maximum likelihood approach.

A final point to make concerns the construction of the logic-tree branches for recurrence parameters. The key message that it is important to ensure that the resulting range of uncertainty (on recurrence rates of earthquakes of different magnitude) is not unintentionally too broad. The *a*- and *b*-values should always be kept together on a single node rather than split as two separate nodes (a practice in some early studies for UK NPP sites, for example) since they are jointly determined, and their separation would lead to combinations that are not consistent with the data. Ideally, the recurrence parameters should also be coupled with Mmax values, which will generally be the case when the penalised maximum likelihood approach is used. Checks should always be made to ensure that the final branches imply seismic activity levels that can be reconciled with the data available for the region, especially on the upper end. Do the higher branches predict recurrence rates of moderate magnitude earthquakes that would be difficult to reconcile with the paucity or even absence of such events in the catalogue? Is the implied rate of moment release with the nature of the region and any estimates, from geological data or remote sensing measurements, of crustal deformation rates?

#### A backbone approach for SSC models?

In the light of the preceding discussions, we can pose the question of whether there is the possibility of adapting the backbone approach to SSC modelling? The key to the backbone approach is a more transparent relationship between the models and weights on the logic-tree branches and the resulting distribution of parameters that move the needle in the hazard calculations. For a given source configuration, a backbone approach is easily envisaged. Stromeyer and Grünthal (2015) actually proposed an approach that would qualify as a backbone approach: in the first step, the uncertainty in the *a*- and *b*-values is propagated, through their covariance matrix, to the estimates of rate at any fixed value of magnitude. The one-dimensional distributions of rates are then re-sampled at each magnitude into an equivalent distribution following Miller and Rice (1983); this is directly comparable to the way that the distribution of AFs is re-sampled at each oscillator frequency in the approach of Rodriguez-Marek et al. (2021a; Sect. 5.3).

**Fig. 64:**
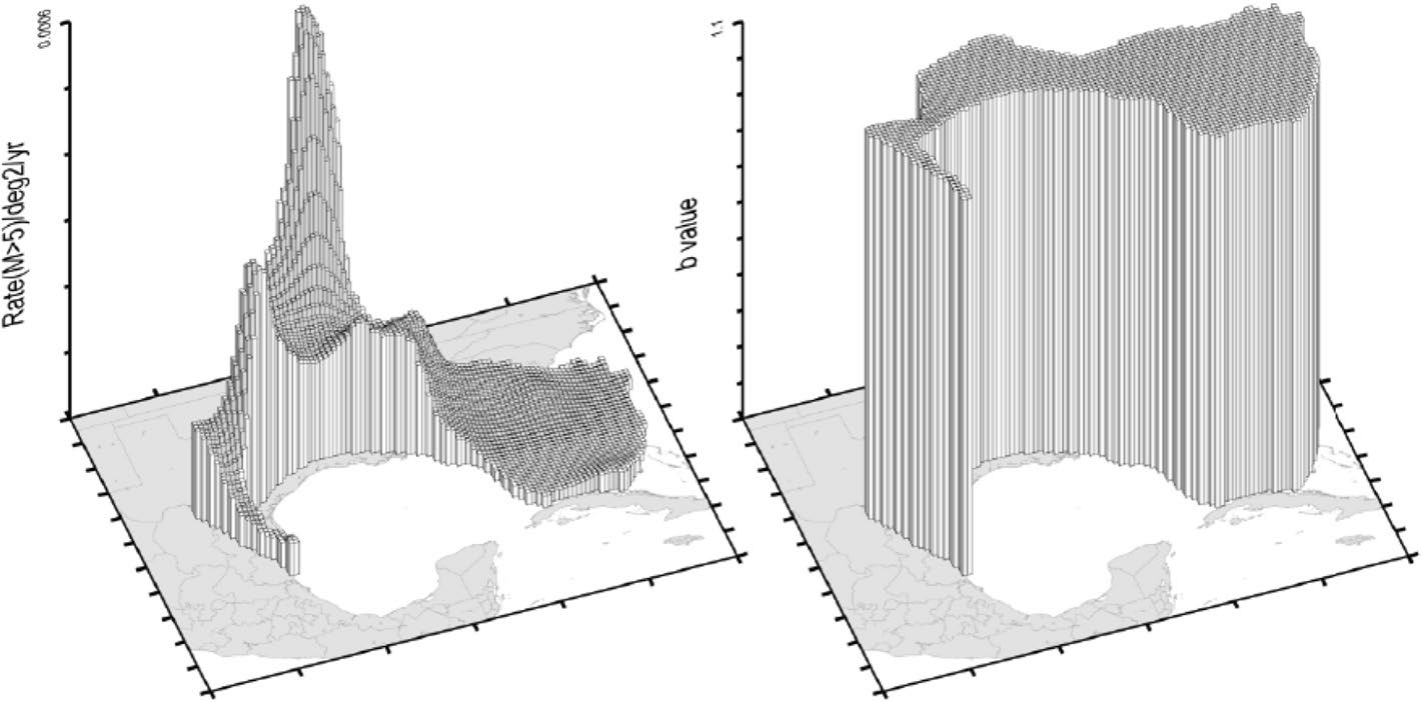
Distribution of activity rates (left) and* b*-values (right) for one seismic source in the CEUS-SSC model (USNRC 2012a)

When the spatial distribution of future seismicity is also included as an epistemic uncertainty through alternative zonations or alternative smoothing operators, the situation becomes complicated. Since the alternative zonations will automatically overlap one another, the logic tree is unlikely to satisfy the MECE criterion. With multiple source zone configurations, it also becomes more difficult to visualise the distributions of location and recurrence rates simultaneously. Maps could be generated that depict the effective rate of earthquakes of a specified magnitude over a spatial grid (Fig. 64), but it would be challenging to represent this information for the full range of magnitudes simultaneously. Herein may lie an interesting challenge for researchers working in the field of seismic source modelling: to develop visualisation techniques that would enable the full implications of an SSC logic tree, in terms of space and rate over the full range of magnitudes from M_min_ to Mmax, to be visualised.

## Uncertainty and expert judgement in PSHA

By this point, I hope that I will have persuaded the reader that the identification, quantification, and clear incorporation of epistemic uncertainty into seismic hazard assessments are fundamental to increasing the chances of the results of such studies being accepted and thus adopted as the starting point for seismic risk mitigation, which is always the ultimate objective. In Sect. 5, I have discussed current approaches to the construction of logic trees, the tool ubiquitously employed in site-specific PSHA projects to manage epistemic uncertainty. In this section I briefly discuss the role of expert judgement in constructing these logic trees and current best practice in terms of procedures for making these judgements.

### The Inevitability of expert judgement

As I have stressed several times, the importance of gathering and analysing data in seismic hazard assessment cannot be overemphasised. The compilation and assessment of existing data is a non-negotiable part of any seismic hazard study, and the collection of new data, particularly for site-specific studies for important facilities, is strongly recommended. However, it is also important to be conscious of the fact that the data will never be sufficient—at least not in any foreseeable future—to allow the unambiguous definition of the unique models for the characteristics and rates of potential future earthquakes and for the ground motions that such events could generate. Consequently, there is always epistemic uncertainty, and the full distribution of epistemic uncertainty cannot be objectively measured. For some practitioners and researchers, this seems to be difficult to accept. Examining the performance of GMMs against local ground-motion data may usefully inform the process of constructing a GMC logic-tree but any quest for a fully objective and data-driven process to select and assign weights to models to occupy the branches is futile. Similarly, procedures to check the consistency of source models with the available earthquake catalogue may also be usefully informative—subject to various assumptions regarding the completeness of the catalogue—but I would argue that at most such techniques can demonstrate that a source model is not invalid (which is not the same as validating the model); this seems to be reflected in the change from “*objective validation*” to “*objective assessment*” in the titles of the papers proposing such testing of source models by Musson (2004) and Musson and Winter (2012).

If the centre, body, and range of epistemic uncertainty cannot be measured from observations, the objective of assessing the CBR of TDI cannot be met without invoking expert judgement. In their proposal for an entirely objective approach to populating the branches of a GMC logic-tree, Roselli et at. (2016) dismiss the application of expert judgement on the basis that “…*. a set of GMPEs is implemented (more or less arbitrarily) in a logic-tree structure, in which each GMPE is weighted by experts, mostly according to gut feeling*.” This is a misrepresentation since what is sought is a judgement, in which there is a clear line of reasoning from evidence to claim, rather than an unsubstantiated or intuitive opinion. The judgements require technical justification and the expert making the judgement should be able to defend the judgement if challenged.

In this context, it is also helpful to clarify exactly what is implied by the term ‘expert’, the meaning of which is two-fold. Firstly, the person making the judgement, or assessment, must be appropriately qualified in the relevant subjects and preferably also experienced in the interpretation of data and models in this field; ideally, the individual will have also received some training in the concepts of cognitive bias and how such bias can influence technical decisions. Secondly, by the time the person is making their judgement, they are expected to have become an expert in the specific application—the seismicity or ground-motion characteristics of the region and the dynamic properties of the site—through study and evaluation of the relevant literature, data, and models. This is quite distinct from classical ‘expert elicitation’ where the objective is usually to extract only the probabilities associated with specified events assuming that this information already exists in the mind of the expert (e.g., O’Hagan et al. 2006).

### Multiple expert judgements

In classical expert elicitation, several experts are usually assembled but the objective is to identify among them the ‘best’ experts, chosen on the basis of their responses to related questions for which the responses are known. As applied to seismic hazard assessment, the purpose of assembling multiple experts is quite different. The intention is to bring different perspectives to the interpretation of the available data, methods, and models, precisely because the objective is not to find the ‘right’ answer but rather to capture the centre, the body, and the range of technically defensible interpretations. Experts with different training and experience are likely to make distinct inferences from the same information and hence increase the chances of capturing the full CBR of TDI.

At the same time, it is important to point out that the intention of engaging multiple experts in a seismic hazard assessment is not intended to increase the chances of constructing a logic tree that represents the views of the broad technical community in the field. Put bluntly, multiple expert hazard assessments should not be conducted as a plebiscite or referendum. Some confusion around this issue arose because of an unfortunate use of words in the original SSHAC guidelines—discussed below—which stated the goal to be capture of the centre, body, and range of the informed technical community (or CBR of the ITC; Budnitz et al. 1997). The intent of this wording was to imply that the study should capture the full distribution of uncertainty that would be determined by any group of appropriately qualified and experienced subject-matter experts who became informed about the seismicity of the region and seismic hazard of the site through participation in the assessment. Regrettably, this intent was often overlooked and the objective of capturing the CBR of the ITC was interpreted as meaning that the views of all experts in the field should be reflected in the logic tree. Such a view may be admirably inclusive and democratic but is unlikely to lead to a robust scientific characterisation. This is important in the context of this paper that is focused on achieving acceptance of the results of seismic hazard assessments, since one could easily lean toward favouring an approach that ensured that many views and models from the broad technical community were included on the basis that this might lead to broader acceptance (if one assumes that all the experts whose views were included would look positively on their preferred model being part of a broad distribution rather than clearly identified as the best model). My view is that we should always make the best possible scientific assessments, and that we should conduct these assessments and document them in ways that are conducive to their acceptance, but the scientific assessment should never be compromised by the desire to achieve acceptance.

**Fig. 65:**
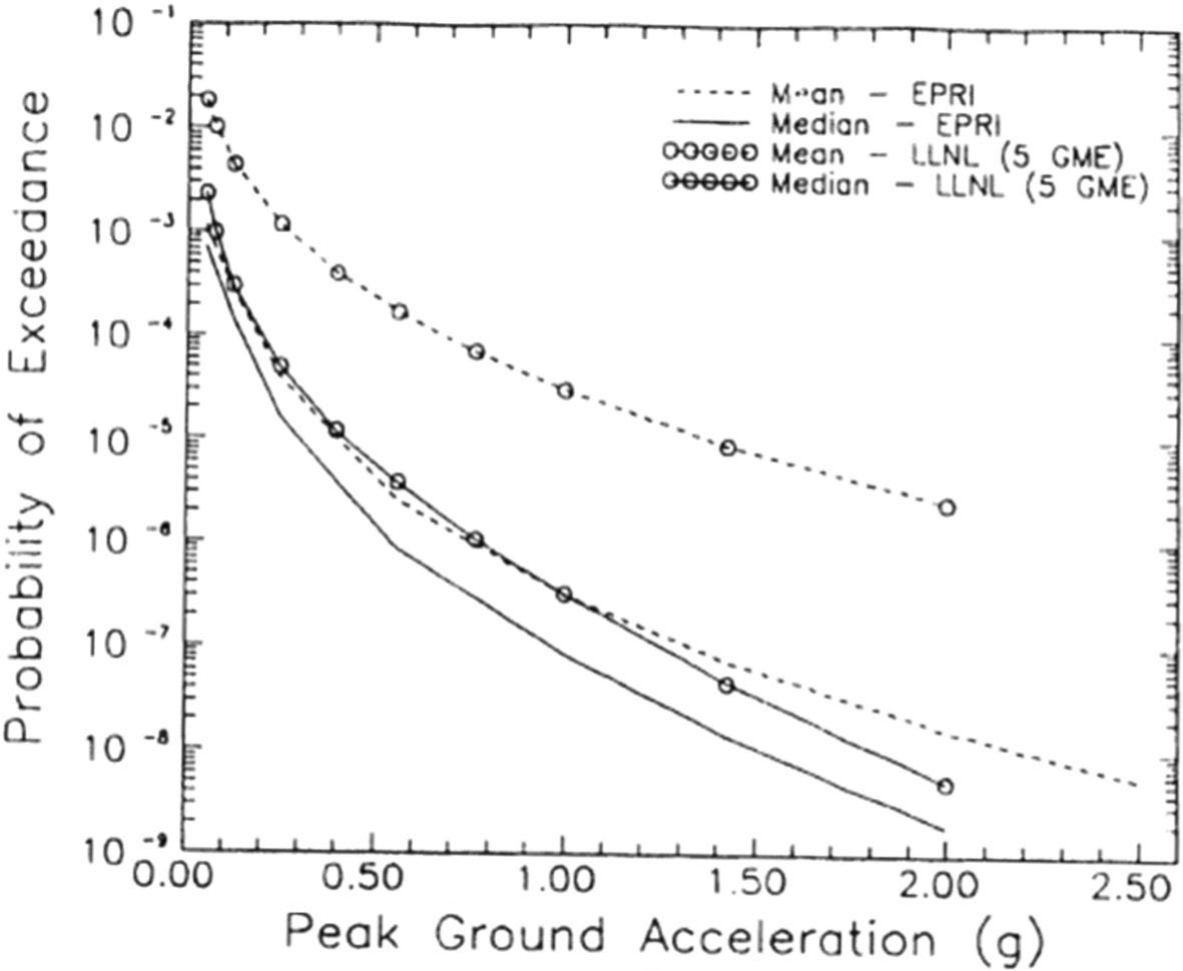
Mean and median hazard curves for PGA at an NPP site in Central and Eastern United States obtained from the EPRI and LLNL PSHA studies (Bernreuter et al. 1987)

The benefits of engaging multiple experts in the assessment of seismic hazard have been recognised for a long time, especially for regions where uncertainties are large as a result of earthquakes occurring relatively infrequently. In the 1980s, two major PSHA studies were conducted for NPPs in the Central and Eastern United States by the Electric Power Research Institute (EPRI) and Lawrence Livermore National Laboratory (LLNL). Both studies engaged multiple experts but conducted the studies in different ways in terms of how the experts interacted. The hazard estimates produced by the two studies for individual sites were very different both in terms of the expected (mean) hazard curves and the implied ranges of epistemic uncertainty (Fig. 65). In response to these divergent outcomes, EPRI, the US Nuclear Regulatory Commission (USNRC), and the US Department of Energy (DOE) commissioned a panel of experts—given the designation of the Senior Seismic Hazard Assessment Committee, or SSHAC—to explore and reconcile the differences between the EPRI and LLNL studies.

Whereas the original expectation was that the SSHAC review might find a technical basis for reconciling the results from the EPRI and LLNL studies, they concluded that the differences arose primarily from differences in the way the two studies had been conducted: “*In the course of our review, we concluded that many of the major potential pitfalls in executing a successful PSHA are procedural rather than technical in character. ….. This conclusion, in turn, explains our heavy emphasis on procedural guidance*” (Budnitz et al. 1997). The outcome of the work of the SSHAC was a report that provided guidelines for conducting multiple expert seismic hazard studies, which became known as the SSHAC guidelines (Budnitz et al. 1997).

### The SSHAC process

Mention of SSHAC or the SSHAC process sometimes provokes a heated response of the kind that is normally reserved for controversial political or religious ideologies. Such reactions are presumably prompted by perceptions or experience of specific implementations of the SSHAC process (see Sect. 7.2) rather than any impartial perusal of the guidelines. The SSHAC guidelines are simply a coherent proposal, based on experience, for how to effectively organise a seismic hazard study involving multiple experts. The essence of the SSHAC process can be summarised in five key characteristics:

1. *Clearly defined roles* Each participant in a SSHAC process has a designated role, and for each role there are specific attributes that the participant must possess and specific responsibilities that they are expected to assume. The clear definition of the roles and responsibilities is the foundation of productive interactions within the project.
2. *Evaluation of data, methods, and models* Databases of all available data, methods, and models are compiled, and supplemented, where possible, by new data collection and analyses. These databases are made available to all participants in the project and the TI Teams (see below) are charged with conducting an impartial assessment of the data, methods, and models for their potential applicability to the region and site under study.
3. *Integration* On the basis of the evaluation, the TI Teams are charged with integrating their assessments into distributions (invariably represented by logic trees) that capture the CBR of TDI.
4. *Documentation* consistent with the description given in Sect. 4.4, the study needs to be summarised in a report that provides sufficient detail to enable the study to be reproduced by others.
5. *Participatory peer review* As discussed in Sect. 4.3, peer review is critical. In a SSHAC process, the peer reviewers are charged with conducting rigorous technical review and to also review the process through which the study has been conducted, which to a large extent means ensuring that the roles and responsibilities are adhered to by all participants throughout the project. The adjective ‘participatory’ is used in SSHAC terminology to distinguish the recommended approach from late-stage review; while the term does reflect the fact that the peer reviewers are present in meetings and workshops throughout the project, it should not be interpreted to mean that they actually engage in the development of the SSC and GMC logic trees—detachment and independence from that activity is essential.

When rigid opposition to the notion of SSHAC is expressed, it has been suggested that those militating against the SSHAC process could be asked which of these five characteristics they find most unpalatable and would not wish to see in a site-specific seismic hazard study. Views regarding specific details of how SSHAC studies are organised are entirely reasonable—the guidelines have evolved iteratively, as discussed in Sect. 6.4—but wholescale rejection of these basic concepts is difficult to understand. There can be little doubt that clear demonstration that a seismic hazard assessment complied with all five of these basic stipulations should be conducive to securing acceptance of the outcomes of the study.

**Fig. 66:**
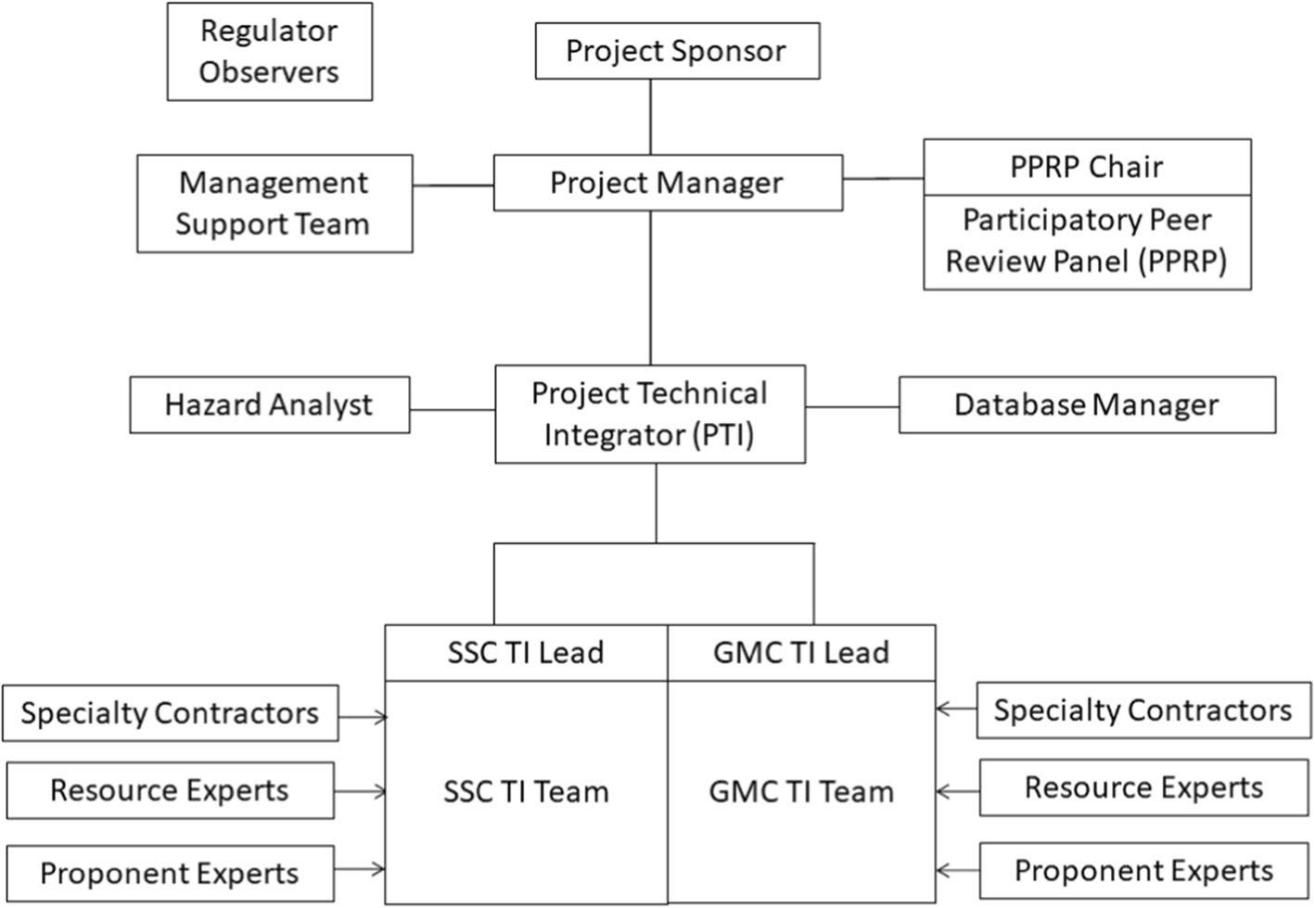
Role and interactions in SSHAC seismic hazard study (USNRC 2018)

Figure 66 illustrates the interactions among the key participants in a SSHAC study. The TI (Technical Integration) Teams are responsible for the processes of evaluation and integration, and ultimately assume intellectual ownership of the SSC and GMC models. Each TI Team has a nominated lead, responsible for coordinating the work of the Team and the interfaces with other parts of the project. Additionally, there is an overall technical lead, called the Project Technical Integrator (PTI); in practice, this position is often filled by one of the TI Leads. The evaluations by the TI Team are informed by Specialty Contractors, who collect new data or undertake analyses on behalf of the TI Teams, and by Resource Experts, who are individuals with knowledge of a specific dataset or region or method that the TI Teams wish to evaluate. The TI Teams also engage with Proponent Experts, who advocate a particular model without any requirement for impartiality. Details of the required attributes and the attendant responsibilities corresponding to each role are provided in USNRC (2018).

From the perspective of acceptance of the results of a PSHA study, the roles of Resource Expert and Proponent Expert are particularly important since they provide a vehicle for the participation by members of the interested technical community, and especially those who have worked on the seismicity, geology or ground-motion characteristics of the region. Their participation can bring very valuable technical insights and information to the attention of the TI Teams, and at the same time give these same experts insight into and knowledge of the hazard assessment project. In many settings, the technical community includes individuals with strong and sometimes even controversial views of the earthquake potential of a particular fault or the location of particular historical events. Dismissing the views of such researchers would be unscientific and also give them ammunition to criticise the project and its findings, but it would also be inappropriate to include their models without due scrutiny purely on the basis of appeasing the proponent. The SSHAC process provides a framework to invite such experts to participate in a workshop—with remuneration for their time and expenses—to allow them to present their views and to then respond to the questions from the TI Teams, all under the observation of the PPRP, thus facilitating an objective evaluation of the model.

The selection of appropriate individuals to perform the specified roles in a SSHAC study is very important and the selection criteria extend beyond consideration of academic qualifications and professional experience. For members of the TI Teams, willingness to work within a team and to be impartial is vital. All the key participants must be able and willing to commit significant time and effort to the project, and the TI Leads and PTI need to be prepared to be engaged very frequently and to be able to respond swiftly and effectively to any questions or difficulties that may (and usually will) arise.

In many ways, the most critical role is that of the participatory peer review panel (PPRP). A final closure letter from the PPRP indicating concurrence that the technical bases of the PSHA input models have been satisfactorily justified and documented, that the hazard calculations have been correctly performed, and that the project was executed in accordance with the requirements of the SSHAC process, is generally viewed as the key indicator of success. Since the PPRP is, in effect, the arbiter for adherence to process, there is very serious onus on the PPRP to diligently fulfil the requirement of their role, always maintaining the delicate balance between engagement with the project and independence from the technical work. The role of the PPRP Chair, who is charged with steering the review panel along this narrow path, is possibly the most challenging, and in some ways most important, position in a SSHAC hazard study.

### SSHAC study levels

The original SSHAC guidelines (Budnitz et al. 1997) defined four different levels for the conduct of hazard studies, increasing in complexity and numbers of participants from Level 1 to Level 4, with the highest level of study being intended for important safety–critical infrastructure or applications that were surrounded by controversy. The intent was that the greater investment of time and resources at the higher study levels would lead to an enhanced probability of regulatory assurance (which, for NPP sites, is the essential level of acceptance of a site-specific PSHA). The enhanced assurance is assumed to be attained by virtue of the higher-level studies being more likely to capture the CBR of TDI, although this remains the basic objective at all study levels.

**Fig. 67:**
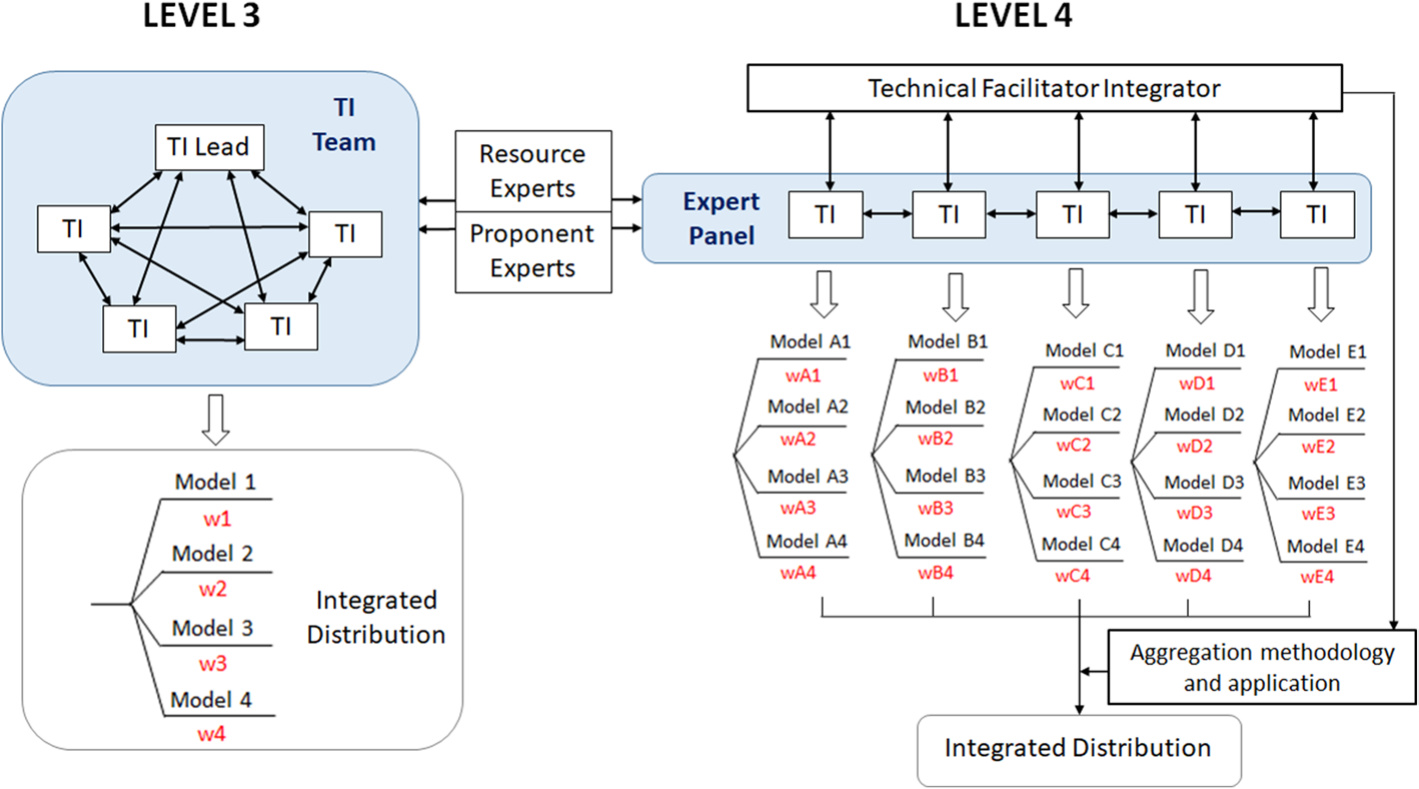
Schematic illustration of the key organisational differences between SSHAC Level 3 and Level 4 studies (modified from USNRC 2018)

Although Budnitz et al. (1997) defined four study levels, detailed implementation guidance was provided only for Level 4, which was implemented in seismic hazard studies for the Yucca Mountain nuclear waste repository in Nevada (Stepp et al. 2001) and the PEGASOS project for NPP sites in Switzerland (Abrahamson et al. 2002). A decade after the original guidelines were issued, USNRC convened, through the USGS, a series of workshops to review the experience of implementing the guidelines in practice. The outcome of these workshops was a series of recommendations (Hanks et al. 2009), the most important of which was that detailed guidelines were also required for Level 3 studies. This led the drafting of NUREG-2117 (USNRC 2012b), which provided clear guidance and checklists for the execution of both Level 3 and Level 4 seismic hazard studies. A very significant development was that in NUREG-2117, the USNRC made no distinction between Level 3 and Level 4 studies in terms of regulatory assurance, viewing the two approaches as alternative but equally valid options for reaching the same objective. The key difference between Level 3 and 4 studies is illustrated in Fig. 67: in a Level 4 study, each evaluator/integrator expert, which may be an individual or a small team, develops their own logic tree for the SSC or GMC model, whereas in a Level 3 study the evaluator/integrators work as a team to produce a single logic tree. In a Level 4 study, there are interactions among the evaluator experts but also with a Technical Integrator Facilitator (TFI), sometimes individually and sometimes collectively.

From a logistical point of view, the Level 4 process is rather cumbersome and Level 3 studies have been shown to be considerably more agile. Moreover, the role of TFI is exceptionally demanding, considerably more so than that of the TI Leads or even the PTI in a Level 3 study. In my view, the Level 3 process offers two very significant advantages over Level 4, in addition to the points just noted. Firstly, if the final logic tree in a Level 4 is generated by simply combining the logic trees of the individual evaluator experts, then it can become enormous: in the PEGASOS project, the total number of branch combinations in the full logic tree was on the order of 10^26^. Such wildly dendritic logic trees pose enormous challenges from a computational perspective, but their size does not mean that they are more effectively capturing the epistemic uncertainty. Indeed, such an unwieldy model probably makes it more difficult to visualise the resulting distributions and inevitably limits the options for performing sensitivity analyses that can provide very valuable insights. The second advantage of Level 3 studies is the heightened degree of interaction among the evaluator experts. In a Level 4 study, there is ample opportunity for interaction among the experts including questions and technical challenges, but ultimately each expert is likely to feel responsibility for her or his own model, leaving the burden of robust technical challenge to the TFI. In a Level 3 study, where the experts are charged to collectively construct a model that they are all prepared to assume ownership of and to defend, the process of technical challenge and defence is envigorated. Provided the interactions among the experts take place in an environment of mutual respect and without dominance by any individual, the animated exchanges and lively debates that will usually ensue can add great value to the process. In this regard, however, it is important to populate the TI Teams with individuals with diverse viewpoints who are prepared to openly debate the technical issues to be resolved during the course of the project. If the majority of the TI Team members are selected from a single organisation, for example, this can result in a less dynamic process of technical challenge and defence, especially if one of the TI Team members, or indeed the TI Lead, is senior to the others within their organisation.

A new update of the SSHAC guidelines was issued in the form of NUREG-2213 (USNRC 2018), which superseded NUREG-2117 and now serves as the standalone reference document for the SSHAC process. The SSHAC Level 3 process has been widely applied in studies for nuclear sites in various countries as well as for hydroelectric dams in British Columbia, and a valuable body of practical experience has thus been accumulated. The insights and lessons learned from these applications led to the drafting of NUREG-2213, which includes detailed guidance on all four study levels, including Level 1, for which the requirements may surprise some people since there seemed to have been a view in many quarters that any PSHA not specifically characterised as SSHAC Level 2, 3 or 4, would, by default, be a SSHAC Level 1, which is very much not the case.

One of the motivations for including guidance on Level 1 and 2 studies, apart from completeness, was the fact that following the Fukushima Daiichi accident in 2011, the USNRC required all NPP operators to re-evaluate their site hazard through a SSHAC Level 3 PSHA. For plants east of the Rocky Mountains, the studies were based on the CEUS-SSC model, which was the outcome of a regional SSHAC Level 3 study, and regional GMMs for hard rock (EPRI 2013b). The application and adaptation of these regional SSC and GMC models to each site were carried out as Level 2 studies, generally focusing on the modification from the reference hard rock condition of the GMMs to local site conditions. This highlighted the need to provide clear guidance on how to conduct Level 2 studies, which is now provided in NUREG-2213. More recently, USNRC commissioned a study to explore the application of the SSHAC Level 2 procedures to site response analyses for PSHA, the findings of which are summarised in a very useful and informative report (Rodriguez-Marek et al. 2021b).

Another important feature of NUREG-2213 is the recognition that the biggest step in the sequence from Level 1 to Level 4 is the jump from Level 2 to Level 3. In order to bridge this gap, the current SSHAC implementation guidelines allow for enhanced Level 2 studies in order to provide recognition for studies conducted fulfilling all of the requirements of a Level 2 study but also availing themselves of some the additional benefits to be accrued by including elements of a Level 3 study. Prior to the issue of NUREG-2213, a number of PSHA projects made the claim to be a Level 2 + or Level 2–3 study, but there was no basis for such qualifications. The augmentations might include enlarged TI Teams, PPRP observation (by one or two representatives of the panel) at some working meetings, and one or more workshops (a Level 3 study is required to conduct three formal workshops with very specific scopes and objectives). While a Level 3 study should continue to be viewed as the optimal choice to achieve regulatory assurance for a site-specific PSHA at a nuclear site, encouragement should be given to all studies that can move closer to this target, and in that regard the option of an augmented or enhanced Level 2 study is a positive development. In effect, this is the approach that has been applied at some UK new-build nuclear sites (Aldama-Bustos et al. 2019).

**Fig. 68:**
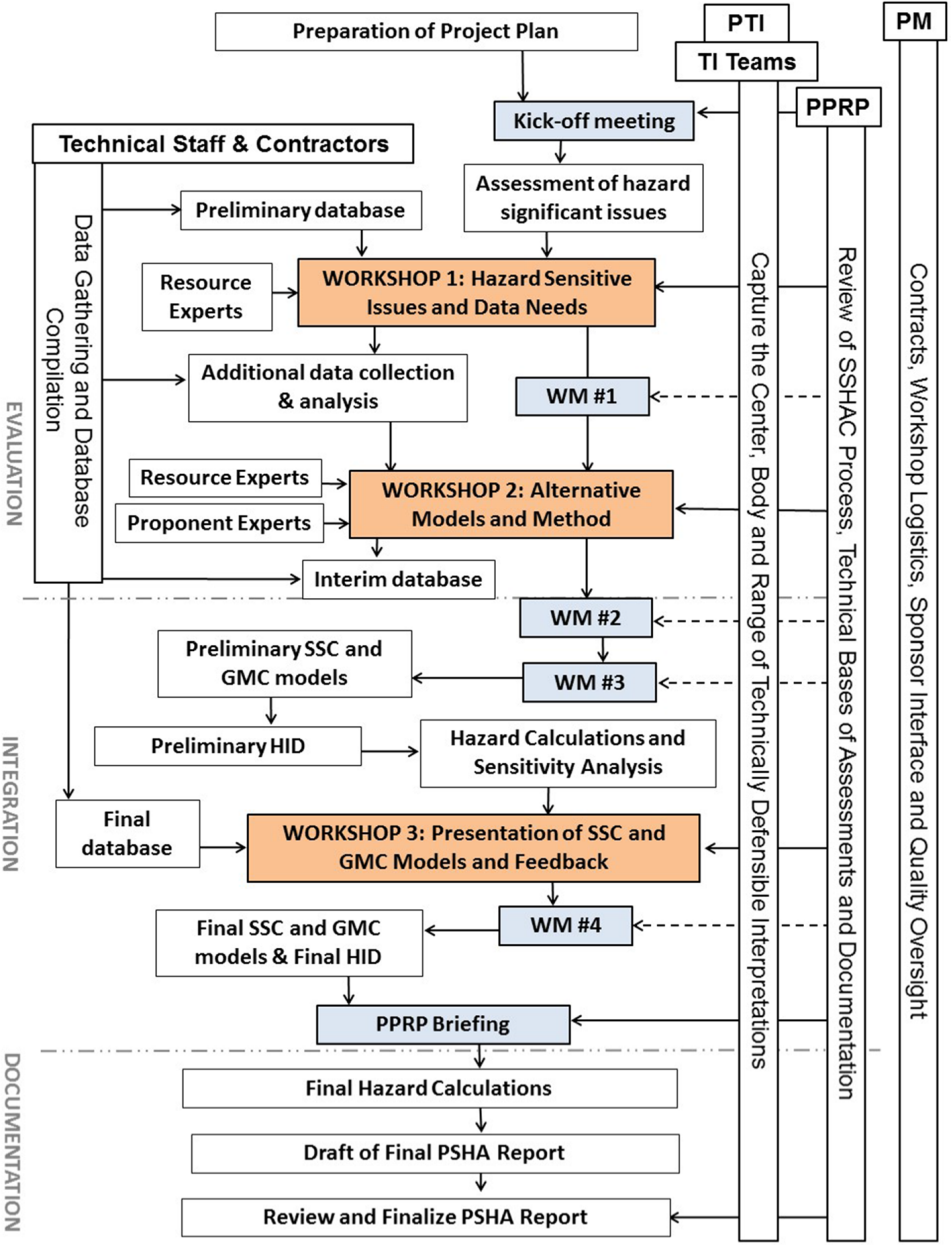
Flowchart identifying the steps involved in conducting a SSHAC Level 3 hazard study, with time running from top to bottom of the diagram (USNRC 2018)

With some precaution, I would like to close this section with a personal view. I am cautious because I would not want this to be invoked as a justification for any company or utility that simply wants to minimise investment in the seismic hazard study for their site, but I will assume that if these suggestions are taken up in practice, it would be for the technical reasons I am laying out. The SSHAC Level 3 process is built around three formal workshops (Fig. 68); the normal format is for the SSC and GMC workshops to be held back-to-back, which has logistical advantages in terms of mobilisation of the PPRP, overlapping for joint sessions at Workshops 1 and 3. These common days for both teams are designed to facilitate identification of interfaces between the two components of the PSHA input models and to discuss hazard sensitivities. I would strongly favour maintaining these two workshops in any study, although it should be possible in many circumstances to combine the kick-off meeting and Workshop 1. Within this general framework, however, I think there could be significant benefits in structuring the main body of the process in different ways because of the very different nature of SSC and GMC model building. The SSC process tends to be data driven, with the TI Team evaluating geological maps, fault studies and geochronology data, geophysical databases (elevation, gravity, magnetism, etc.), and the historical and instrumental earthquake catalogues, as well as models proposed for regional tectonic processes and seismogenic potential of key structures. On the GMC side, the database is generally limited to ground-motion recordings and site characterisation, and much of the work lies in developing the framework for how to build the models for reference rock motions and for site amplifications. I would argue that advances made in these areas in recent years are beginning to reach a kind of plateau in terms of establishing an appropriate basic framework (as presented in Sects. 5.2 and 5.3), which will be refined but possibly not fundamentally changed.

The framework that has evolved through several SSHAC projects, supplemented by research published in many papers, can now be adopted, I believe, for site-specific hazard assessments, with minor adjustments being made as required for each application. If this is the case, the work of the GMC TI Team will focus on using the available ground-motion data and site characterisation (V_S_ and lithology profiles, local recordings to infer kappa, and, in some cases, dynamic laboratory tests on borehole samples to constrain MRD curves). Such endeavours may not be particularly assisted by the conduct of a formal GMC Workshop 2 and are generally better advanced through formal and informal working meetings (with PPRP observers present at the former). At the same time, for key issues on the SSC side, workshops that extend beyond the usual three days may be very useful, especially if there is the flexibility to break out from the formality of these workshops. Imagine a case, for example, where one or two faults close to the site potentially exert a controlling influence on the hazard but their seismogenic potential is highly uncertain. In such a situation, an alternative format could be ‘workshop’ that began with a day of presentations on what is known about the structures, followed by a one- or two-day field trip to visit the structures in the field, possibly including what geologists sometimes refer to as a ‘trench party’, and then another day or two of working meeting in which the observations could be discussed by the SSC TI Team and several Resource and Proponent Experts. This more flexible approach might lead to the GMC sub-project being classified as an augmented Level 2 study, whereas the SSC sub-project could effectively exceed the basic requirements for a Level 3 study. The classification that would then be assigned to the whole process is not clear although it would perhaps be discouraging for a study organised in this way to only be given Level 2 status. There may be a case, in the next iteration of the SSHAC guidelines, to provide more flexibility for how the central phase of a Level 3 study is configured, allowing for differences in how the SSC and GMC sub-project navigate the route between Workshops 1 and 3.

### Regional versus site-specific studies

**Fig. 69:**
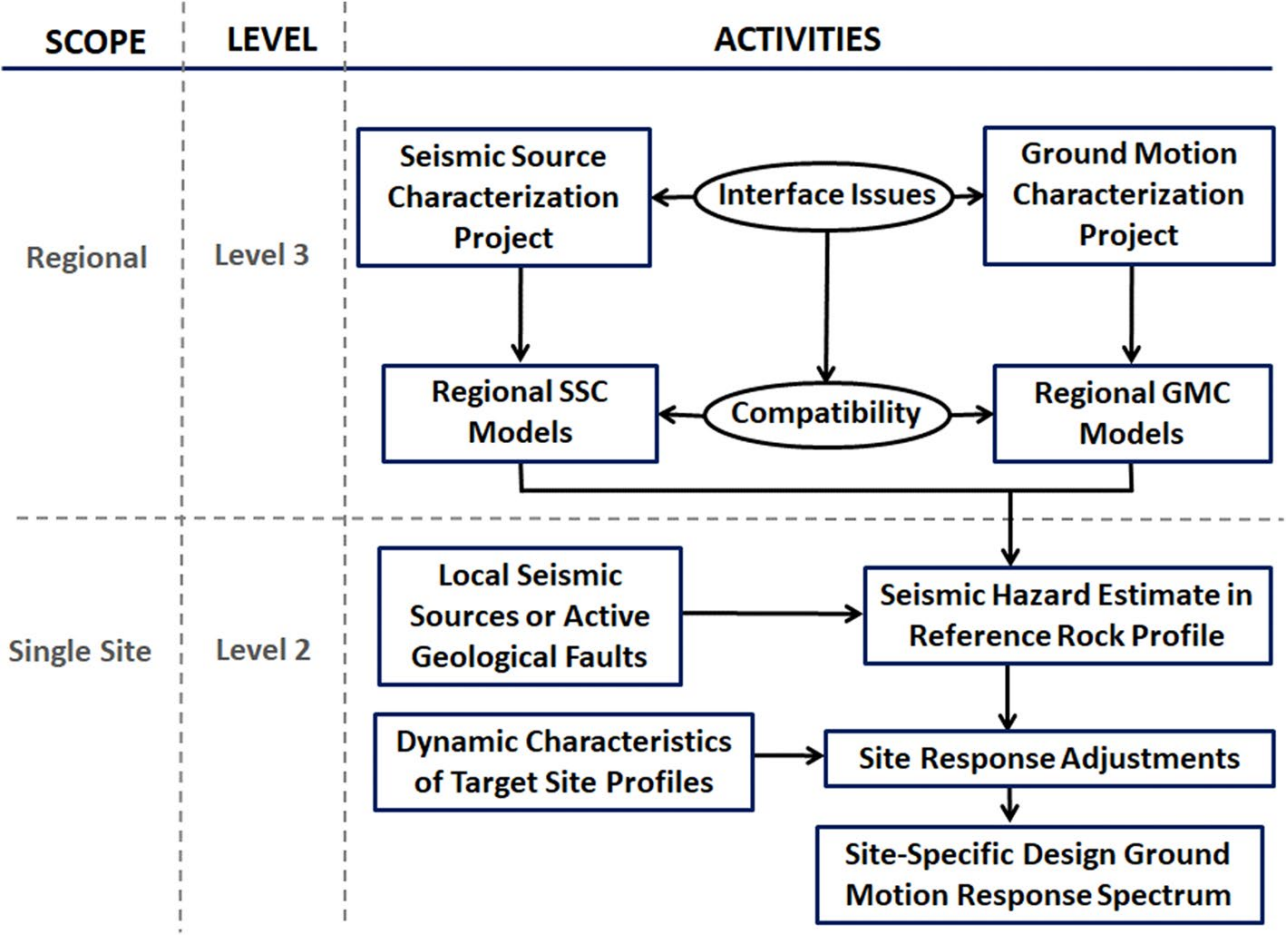
Scheme for regional SSC and GMC model development through Level 3 studies and local updating through Level 2 studies (modified from USNRC 2018)

In the previous section, mention was made of the use of two regional models as the basis for re-evaluations of seismic hazard at NPP sites in the Central and Eastern United States following the Tōhoku earthquake of March 2011 and the nuclear accident at the Fukushima Daiichi plant (as the first stage of a screening process to re-evaluate the seismic safety of the plants). The CEUS-SSC model (USNRC 2012a) was produced through a SSHAC Level 3 project and the EPRI (2013b) GMC model was generated through a SSHAC Level 2 update of GMMs that had been produced in an earlier Level 3 study (EPRI 2004) and then refined in a Level 2 update (EPRI 2006b). The EPRI (2013a) GMC model has since been superseded by the SSHAC Level 3 NGA-East project (Goulet et al. 2021; Youngs et al. 2021). In view of the large number of NPP sites east of the Rocky Mountains, the use of regional SSC and GMC SSHAC Level 3 studies, locally updated through Level 2 projects, was clearly an efficient way to obtain reliable hazard assessments in a relatively short period of time. Such a use of regional SSC and GMC models developed through Level 3 studies to be updated by local Level 2 studies is illustrated in Fig. 69. An alternative scheme is for the seismic hazard at all the sites in a region to be evaluated simultaneously in a single project, an example of which is the recently completed SSHAC Level 3 PSHA that was conducted for the six NPP sites in Spain; this was made possible because the study was commissioned by an umbrella organisation representing all the utilities who own and operate the different plants.

**Fig. 70:**
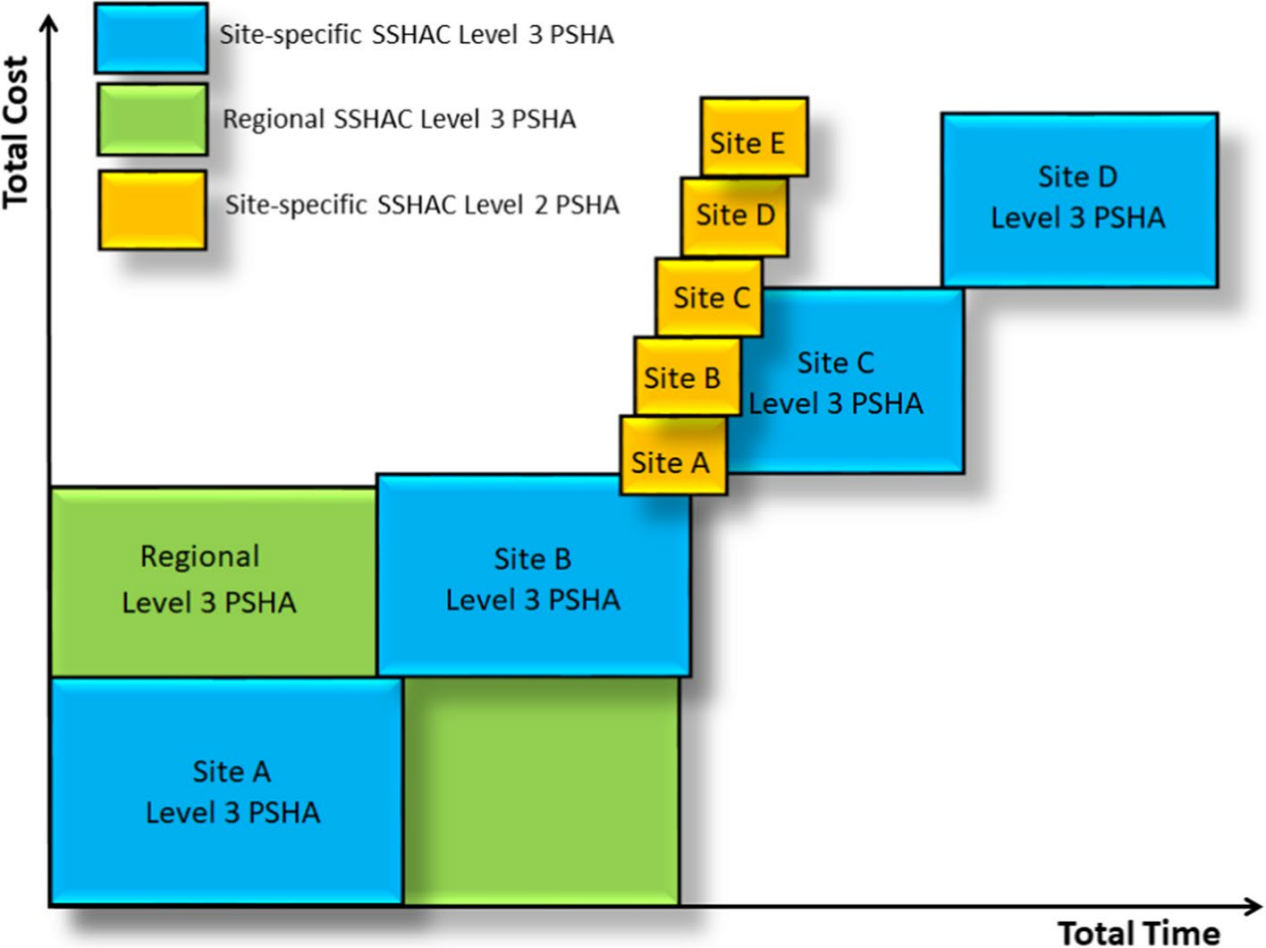
Schematic illustration of cost and time of alternatives for conducting SSHAC PSHA studies at multiple sites in a region (Coppersmith and Bommer 2012)

There are compelling pragmatic reasons for following this path when seismic hazard assessments are required at multiple locations within a region, including the fact that it offers appreciable cost savings once assessments are required for two or more sites. Moreover, since the pool of available experts to conduct these studies remains relatively small, it also allows streamlining of schedule since the local Level 2 updates require fewer participants. Both of these practical benefits are illustrated schematically in Fig. 70.

There is also, however, another potential benefit, especially for the case when two or more nuclear sites are closely located to one another in a given region. If completely parallel studies are undertaken by different teams, then there is a real possibility of inconsistent hazard results (after accounting for differences in site conditions), which could highlight fundamental differences in SSC and/or GMC modelling. This would present a headache for the regulatory authority and do nothing to foster confidence in the studies towards the goal of broad acceptance of the resulting hazard estimates.

If the traditional approach of hazard analysis at a buried rock horizon followed by site response analysis for the overlying layers (Fig. 48) is followed, the multiple-site approach relies on the assumption that a good analogue for the reference rock profile can be encountered at all target sites. Since this will often not be the case, the alternative one-step site adjustment approach (Fig. 49) lends itself perfectly to the development of a regional GCM model that can be applied to target locations and then the hazard adjusted for the differences between the host rock profile of the backbone GMM and the complete upper crustal profile at the target site.

In a region of low seismicity like the UK, where SSC models are dominated by seismic source zones with seismicity rates inferred from the earthquake catalogue, the regional scheme depicted in Fig. 69 would seem like a very attractive option, especially given the small number of specialists in this field based in the UK. More than a decade ago, I proposed that such an approach be adopted as the nuclear new-build renaissance was beginning (Bommer 2010). Since then, site-specific assessments at five nuclear sites, conducted by different groups, have been initiated, which can only be viewed as a lost opportunity, especially in view of the small geographical extent of the UK and the reliance of all these studies on the earthquake catalogue of the British Geological Survey, and the fact that it would be very difficult to justify a regionalised ground-motion model for different parts of this small country.

### How much uncertainty is enough?

A misconception in some quarters is that application of the SSHAC process leads to broad uncertainty in hazard assessments, the implication being that had the hazard been assessed without following an alternative procedure, the uncertainty would somehow have been absent. As McGuire (1993) stated: “*The large uncertainties in seismic hazard are not a defect of the method. They result from lack of knowledge about earthquake causes, characteristics, and ground motions. The seismic hazard only reports the effects of these uncertainties, it does not create or expand them*”. The starting point for any seismic hazard study should be a recognition that there are epistemic uncertainties, and the study should then proceed to identify and quantify these uncertainties, and then propagate them into the hazard estimates. But the objective is always to first build the best possible input models for PSHA and then to estimate the associated uncertainty (in other words, all three letters of the acronym CBR are equally important). The purpose of the SSHAC process is not only to capture uncertainties, and it is certainly not the case that one should automatically expect broader uncertainty bands when applying higher SSHAC study levels. In the not-too-distant past, the indications are that many seismic hazard assessments were rather optimistic about the state of knowledge and how much was truly known about the seismicity and ground-motion amplitudes in a given region. Attachment to those optimistic views regarding epistemic uncertainty have prompted some of the opposition to the SSHAC process, as discussed in Sect. 7.2.

**Fig. 71:**
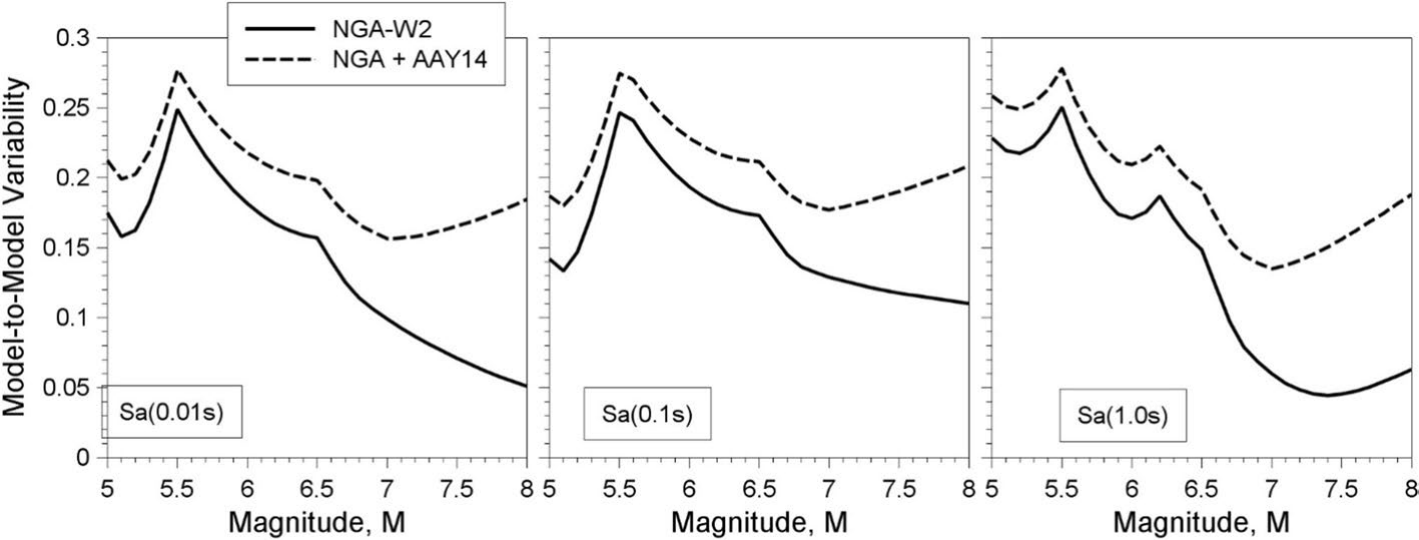
Model-to-model variability of median predictions at a site with V_S30_ = 760 m/s from four NGA-West2 models (see Fig. 21) with and without the additional epistemic uncertainty intervals proposed by Al Atik and Youngs (2014), for strike-slip earthquakes of different magnitude on a vertically dipping fault

A question that often arises when undertaking a PSHA, is whether there is a way to ascertain that sufficient epistemic uncertainty has been captured. The required range of epistemic uncertainty cannot be measured, since the range of the epistemic uncertainty, by definition, lies beyond the available data. For individual components of the hazard input models, comparisons may be made with the epistemic uncertainty in other models. For example, for the GMC model, one might look at the range of epistemic uncertainty in the NGA-West models, as measured by the model-to-model variability (rather than their range of predicted values), and then make the inference that since these models were derived from a data-rich region, their uncertainty range should define the lower bound on uncertainty for the target region. However, there are many reasons why such an approach may not be straightforward. Firstly, the uncertainty defined by the NGA-West2 GMMs displays a trend of decreasing in the magnitude ranges where the data are sparser, although this is improved with application of the Al Atik and Youngs (2014) additional uncertainty penalty (Fig. 71). Secondly, the site-specific PSHA might be focused on a region that is much smaller than the state of California for which the NGA-West2 models were developed (using a dataset dominated by other regions in the upper range of magnitudes). The dynamic characterisation of the target site is also likely to be considerably better constrained than the site conditions at the recording stations contributing to the NGA-West2 database, for which just over half have V_S30_ values inferred from proxies rather than measured directly (Seyhan et al. 2014).

Another option is to compare the epistemic uncertainty in the final hazard estimates, measured for example by the ratio of spectral accelerations at the 85^th^ percentile to those at the 15^th^ percentile (Douglas et al. 2014b), obtained in other studies. In general, such comparisons are not likely to provide a particular useful basis for assessing the degree of uncertainty in a site-specific study, and certainly it would be discouraging to suggest that the uncertainty captured in hazard estimates for other sites should define the minimum threshold, unless one were able to access such information for a study in which there was abundant seismological and excellent site characterisation information, whence the uncertainty might then be taken as a minimum threshold. Otherwise, an expectation of matching some threshold level of uncertainty might remove the motivation to collecting new data and performing analyses that would help to constrain the model and reduce the uncertainty. At the end of the day, the onus lies with the PPRP to make the judgement as to whether the uncertainty bounds defined are consistent with the quality and quantity of the information available for the hazard assessment. In site-specific PSHA studies in which I have participated, there have been occasions when the PPRP has questioned uncertainty ranges for potentially being too broad as well as the more commonly expected case of challenging uncertainty intervals viewed as being too narrow.

## The assessment and acceptance of seismic hazard estimates

Important technical (Sect. 5) and procedural (Sect. 6) advances that have been made to facilitate and render more transparent the process of capturing uncertainties in PSHA, which is foundational to achieving regulatory assurance. However, even seismic hazard studies performed with great rigour can sometimes encounter vehement opposition rather than general acceptance. This section discusses some of the motivations for the rejection of hazard estimates, which, more often than not, lie in objection to the amplitude of the ground motions that result from PSHA. However, as discussed in Sect. 7.4, there are a few cases where hazard estimates have been exaggerated—sometimes with far-reaching consequences for infrastructure projects—and opposition to the hazard estimates was fully justified.

### The diehard determinists

According to some researchers and practitioners, all PSHA studies should be rejected because the approach is fundamentally flawed and PSHA should be discarded in favour of deterministic hazard assessments. There are important differences between PSHA and DSHA but turning the choice between the two approaches into an issue that takes on almost ideological overtones does nothing to promote seismic risk mitigation, as discussed in Sect. 3.1. McGuire (2001), a pioneer and proponent of PSHA, presents a very balanced discussion of how both deterministic and probabilistic approaches to seismic hazard and risk analysis can be useful for different types and scales of application. Articles by the advocates of DSHA have tended to adopt a less constructive attitude towards probabilistic approach and have generally tried to utterly discredit PSHA (e.g., Krinitzsky 1995a, 1995b, 1998, 2002; Paul 2002; Castaños and Lomnitz 2002; Wang et al. 2003; Peresan and Panza 2012; Stein et al. 2012; Wyss et al. 2012; Bela 2014; Mulargia et al. 2017). While some of these articles are amusing to read, none of them take us any closer to seismic hazard assessments that enable risk-informed decision making that optimises the use of limited resources. For the reader with time to spare, I would particularly recommend the paper by Panza and Bela (2020) and its 105-page supplement, which offers very interesting insights.

The views of the diehard determinists were perhaps most clearly expressed in a statement by an organisation calling itself the International Seismic Safety Organisation (ISSO), which issued a statement that only DSHA or NDSHA (Neo-deterministic seismic hazard assessment; Peresan and Panza 2012) “*should be used for public safety policy and determining design loads*” (www.issoquake.org/isso/). Signatories to the statement included Ellis Krinitzsky and Giuliano Panza, both of whom are cited above for their anti-PSHA essays and who also provided forums, as former editors of *Engineering Geology* and *Pure and Applied Geophysics*, respectively, for many other articles along similar lines. The ISSO statement included the following observations on PSHA and DSHA that are worth citing in full:

*“The current Probabilistic Seismic Hazard Analysis (PSHA) approach is unacceptable for public safety policy and determining design loads for the following reasons:*

*(1) Many recent destructive earthquakes have exceeded the levels of ground motion estimates based on PSHA and shown on the current global seismic hazard map. Seismic hazards have been underestimated here.*

*(2) In contrast, ground motion estimates based on the highest level of PSHA application for nuclear facilities (e.g., the Yucca Mountain site in USA and sites in Europe for the PEGASOS project) are unrealistically high as is well known. Seismic hazards have been overestimated here.*

*(3) Several recent publications have identified the fundamental flaws (i.e., incorrect mathematics and invalid assumptions) in PSHA, and have shown that the result is just a numerical creation with no physical reality. That is, seismic hazards have been incorrectly estimated.*

*The above points are inherent problems with PSHA indicating that the result is not reliable, not consistent, and not meaningful physically. The DSHA produces realistic, consistent and meaningful results established by its long practice and therefore, it is essential that DSHA and its enhanced NDSHA should be adopted for public safety policy and for determining design loads.”*

The third bullet is not substantiated in the statement and the mathematical errors in PSHA often alluded to by opponents of PSHA have never been demonstrated—the error seems to reside in their understanding of PSHA. The first two bullets, which respectively claim that PSHA underestimates and overestimates the hazard, warrant some brief discussion. Regarding the first bullet, the accusation is essentially that PSHA is unsafe whereas DSHA somehow provides a greater level of assurance. In some cases, earthquakes have occurred that exceed the size and location of potential future events defined in seismic hazard models; examples of this are highlighted in Fig. 62. Another example of this is the March 2011 Tōhoku earthquake in Japan, which exceeded the magnitude of the earthquake defined as the design basis for the Fukushima Daiichi NPP, which resulted in the tsunami defences being inadequate (although, as explained in Sect. 1, the resistance to ground shaking was not exceeded). These are, however, examples of shortcomings in how the hazard has been estimated—and perhaps in particular how uncertainties have not been adequately characterised—rather than an inherent failure of the PSHA approach (Geller 2011; Stein et al. 2011; Hanks et al. 2012). Other examples cited in the ISSO statement refer to cases of recorded ground motions exceeding ground motions specified in probabilistic hazard maps. Such comparisons overlook the nature of probabilistic seismic hazard maps—which are not predictions much less upper bound predictions—and are not a meaningful way to validate or invalidate a PSHA-based hazard map (e.g., Iervolino 2013; Sect. 12.3 of Baker et al. 2021). The only meaningful comparison between recorded motions and probabilistic hazard maps would be that proposed by Ward (1995): if the map represents motions with a 10% probability of exceedance in 50 years (i.e., a return period of 475 years), then one should expect motions in 10% of the area to exceed the mapped values during an observational period of 50 years. The misleading claim by the proponents of DSHA is that it leads to seismic safety by establishing the worst-case ground motions, something which is clearly not the case, although its application will also be very conservative in many situations (only the degree of conservatism will be unknown).

The second bullet in the ISSO statement quoted above, interestingly, makes the opposite accusation, namely that PSHA sometimes overestimates the hazard. Two specific cases are mentioned, PEGASOS and Yucca Mountain, and these are both discussed below in Sect. 7.2.1 and 7.3 respectively.

Any rigid attachment to DSHA is an increasingly anachronistic stance and the continued attacks on PSHA are an unhelpful distraction: I would propose that society is better served by improving the practice of PSHA rather than declaring it a heresy. Indeed, while scenario-based hazard assessments have their place (see Sect. 9), it is high time that the use of DSHA as the basis for establishing design ground motions, especially for safety–critical structures, should be abandoned. In this regard, the International Atomic Energy Agency (IAEA) could play an important role. IAEA guidelines on seismic hazard assessment for nuclear sites still allow DSHA, which is unavoidable for as long as this is viewed as an acceptable approach by nuclear regulators in any member country. However, the current guidelines also encourage comparison of the results obtained with the two approaches: “*The ground motion hazard should preferably be evaluated by using both probabilistic and deterministic methods of seismic hazard analysis. When both deterministic and probabilistic results are obtained, deterministic assessments can be used as a check against probabilistic assessments in terms of the reasonableness of the results, particularly when small annual frequencies of exceedance are considered*” (IAEA 2010). Exactly what is meant by the term ‘reasonableness’ is not clarified but it would seem more appropriate to specify that the PSHA results should be disaggregated (which is mentioned only in an Appendix of SSG-9) and to evaluate the M-R-$\varepsilon$ triplets controlling the hazard, rather than to compare the PSHA results with the ground motions that would have been obtained by arbitrarily selected values of these three parameters. Nuclear safety goals should ultimately be defined in probabilistic terms and probabilistic estimates of risk cannot be obtained using the outputs from DSHA. And in terms of safety goals, PSHA offers a rational framework to select appropriate safety targets and the level of confidence that the selected target is being reached (Fig. 32).

### Resistance to exceeded expectations

The most energised crusades that I have witnessed against the outcomes from PSHA studies have been in cases where the resulting design ground motions significantly exceeded earlier hazard estimates or preconceptions regarding the general hazard level of a region. As has been discussed earlier in the paper, new information can be found that will challenge existing hazard estimates, but this new data can be acknowledged and assessed impartially, as was the case for the Shoreline Fault adjacent to the Diablo Canyon NPP in California (Sect. 5.4). In this section, I recount two case histories where, for very distinct reasons, new hazard estimates were not received with such equanimity.

#### The PEGASOS project

**Fig. 72:**
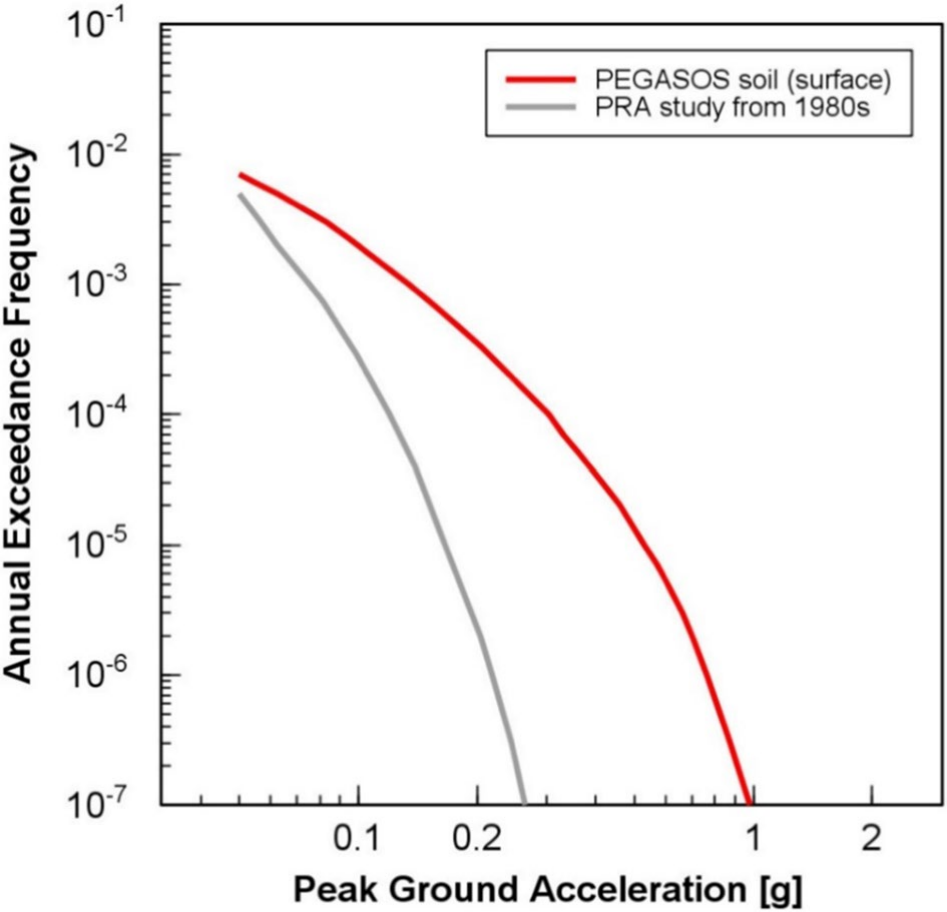
Comparison of median hazard curve for a Swiss NPP site from PEGASOS with the hazard curve obtained from an earlier PSHA in the 1980s (adapted from Bommer and Abrahamson 2006)

The PEGASOS project was a SSHAC Level 4 PSHA for NPP sites in Switzerland that ran from 2000 to 2004, organised with sub-projects for the SSC model, the GMC model for rock, and the local site response (Abrahamson et al. 2002). As noted in Sect. 6.4, the final logic tree resulted in branch combinations exceeding Avagadro’s number, which created severe computational challenges. When the results were released, they met with stern and sustained opposition led by Dr Jens-Uwe Klügel (Klügel 2005, 2007, 2008, 2011), representative of one of the Swiss NPPs (and, coincidentally, a signatory to the ISSO statement discussed in Sect. 7.1). The basic motivation for Dr Klügel’s crusade was very clear: the PEGASOS results represented a very appreciable increase in the existing seismic hazard assessment for the Swiss plants (Fig. 72). The plants were originally designed using deterministic hazard estimates but in the 1980s, PSHAs were performed to provide the input to probabilistic risk analyses (PRA); the PEGASOS results were significantly higher.

**Fig. 73:**
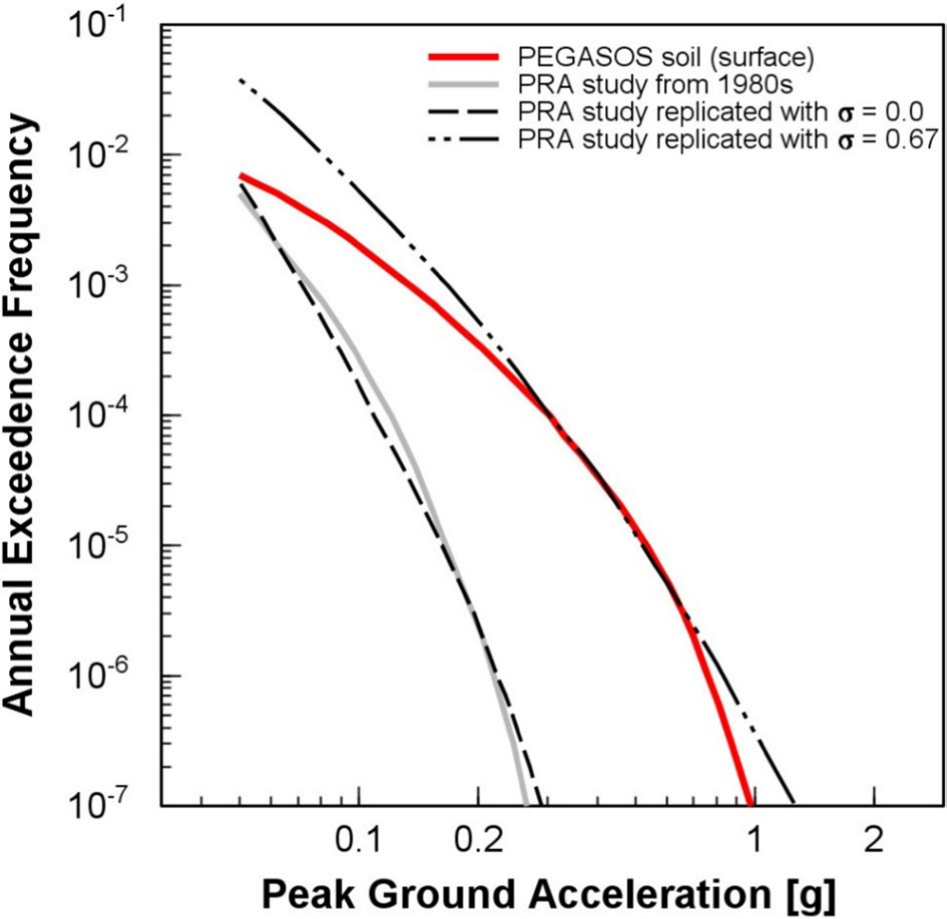
The same as Fig. 72 but with hazard curves from the 1980s PSHA model reproduced with and without sigma (adapted from Bommer and Abrahamson 2006)

Responses to the original assault on PEGASOS by Klügel (2005) were published, focusing on defence of PSHA and the SSHAC process (Budnitz et al. 2005), as well as pointing out flaws in the ‘validation’ exercises presented in Dr Klügel’s paper (Musson et al. 2005), while others—coincidentally another core member of ISSO—rallied to support Dr Klügel’s position (Wang 2005). However, none of these exchanges touched the core issue: the old hazard results being defended were incorrectly calculated. As shown in Fig. 73, it was possible to reproduce the hazard curve from the 1980s PSHA, based on the available documentation, but only by neglecting the sigma in the GMM—which does not, by any modern standard, constitute a PSHA. When the hazard calculations were repeated assigning an appropriate sigma value, the median hazard curve at the surface was slightly higher than that obtained from the PEGASOS calculations. This information was shared with Dr Klügel but had no effect on his campaign to invalidate the hazard results from PEGASOS.

The curves in Figs. 72 and 73 do not, however, tell the entire story because these plots show only the median hazard. The mean hazard from the PEGASOS study was higher than the correctly calculated (i.e., including sigma) mean hazard from the 1980s PSHA, indicating greater epistemic uncertainty. In large part, this was the result of a very optimistic view of how much was known by those conducting the earlier hazard study. However, in fairness there was also avoidable uncertainty included in the PEGASOS model, primarily because of a decision to undertake no new data collection, including no site characterisation measurements—although, interestingly, this was not a criticism included in Klügel (2005).

The controversy created by Dr Klügel’s campaign resulted in long delays to the hazard results being adopted in risk assessments for the Swiss plants and also succeeded in tarnishing not only the PEGASOS project but also the SSHAC process, fuelling numerous criticisms of the process (e.g., Aspinall 2010). The final outcome was a new PSHA study, the PEGASOS Refinement Project (PRP; Renault et al. 2010), which began in 2008 and ended in 2013. While there were clearly very major improvements made during the PRP and important lessons were certainly learned, the fact remains that an individual was able to launch a campaign that stopped the adoption of a major PSHA study, involving experts from the United States and throughout Europe, prompted by objection to the results on the basis that they exceeded previously hazard estimates that had been incorrectly calculated.

#### The Panama Canal expansion

**Fig. 74:**
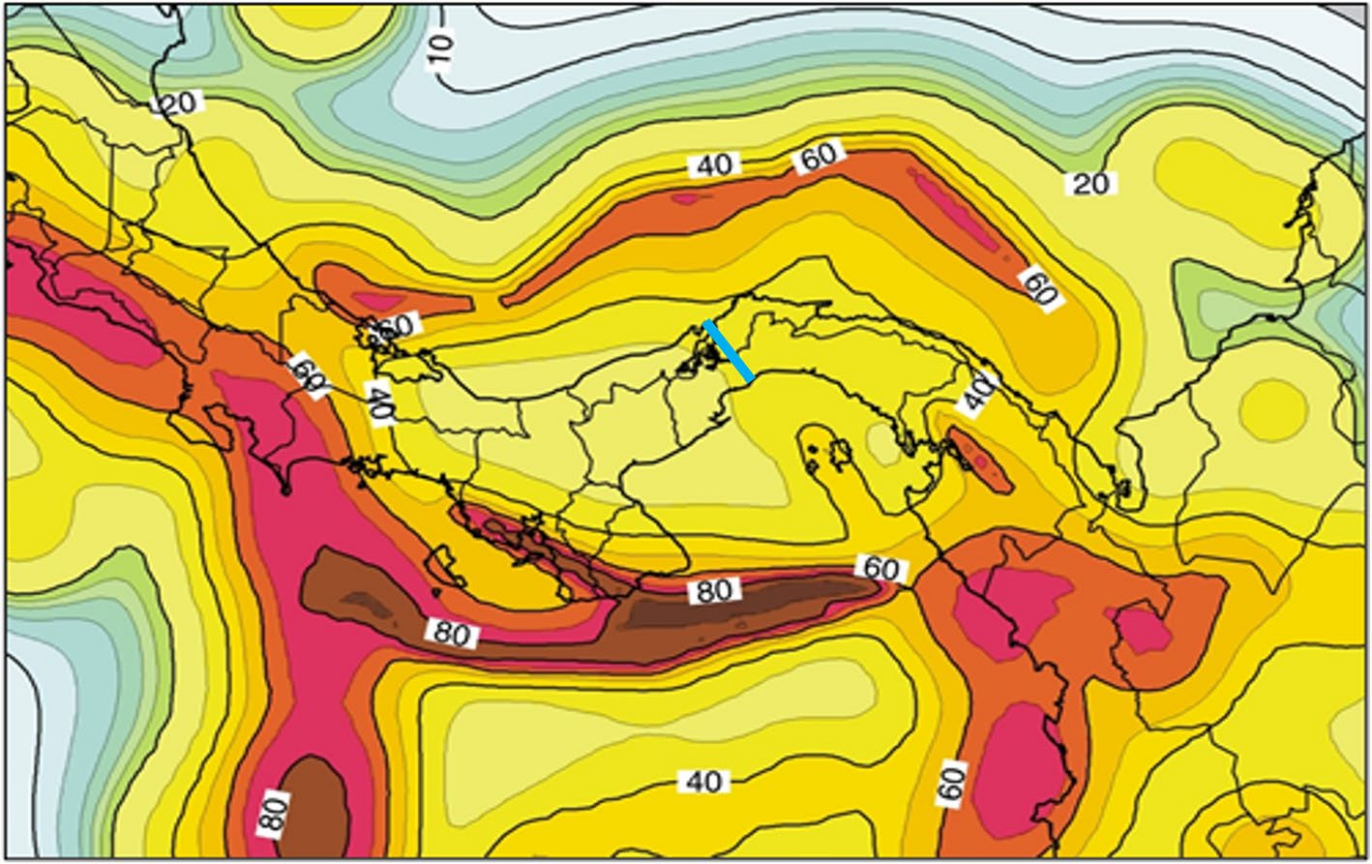
USGS 2003 hazard map of Panama in terms of PGA (%g) for a return period of 2,500 years; the light blue line shows the approximate route of the canal

In Sect. 5.4, I described the discovery of the Pedro Miguel fault as a result of investigations undertaken as part of the Panama Canal expansion project. The identification of this active fault in central Panama, striking sub-parallel to the Pacific side of the canal and approaching the route very closely near the new locks, resulted in a radical change of the estimated seismic hazard. Prior estimates of seismic hazard in central Panama were based primarily on active sources of earthquakes offshore to south and north of the isthmus, the latter being the location of a well-documented earthquake on 7 September 1882 (Fig. 74). The inclusion of the 48 km-long Pedro Miguel fault, and other active structures identified during the same studies, increased the 2,500-year PGA at the Pacific (Miraflores) locks by a factor of 2.5 from 0.40* g* to 1.02* g*.

**Fig. 75:**
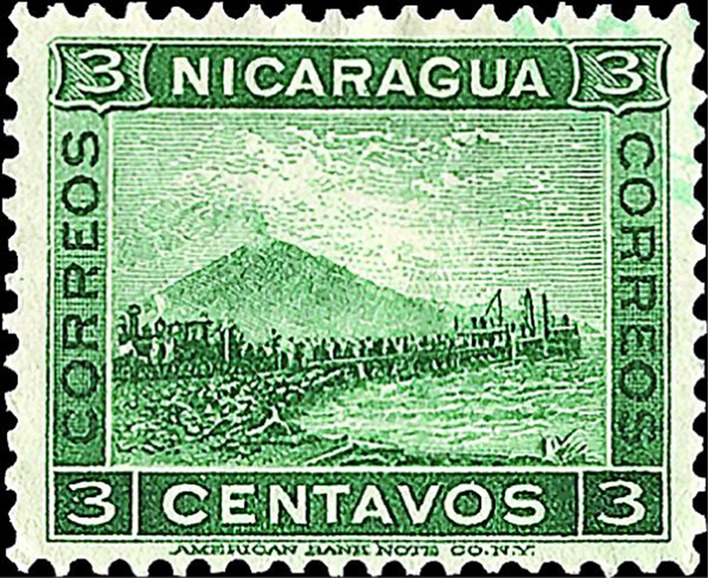
Postage stamp from Nicaragua depicting the active Momotombo stratovolcano. (https://www.linns.com/news/us-stamps-postal-history/)

Unsurprisingly, the news of this huge increase in the estimated hazard came as a shock for the ACP. To fully appreciate the challenge that this new data presented, it is helpful to understand the historical context. Following the failure of the French project to build the Panama Canal, the canal was eventually built by the United States, in what was truly a colossal engineering project that involved the creation of a new country (prior to November 1903, Panama was a province of Colombia) and the effective annexation of part of that country by the US (the Panama Canal zone). Before embarking on the project, two separate groups had lobbied for different routes for an inter-oceanic canal through the isthmus of Central America, one in Panama and the other in Nicaragua. On the day that the US Senate finally came to vote on which route to adopt, the Panamanian option was selected by 42 to 34 votes. On the morning of the vote, senators had received postcards with Nicaraguan postage stamps depicting active volcanoes (Fig. 75), which is believed to have swayed several undecided lawmakers to vote in favour of the Panama option. For the history of how the Panama Canal came into being, I strongly recommend David McCullough’s excellent book (McCullough 1977).

**Fig. 76:**
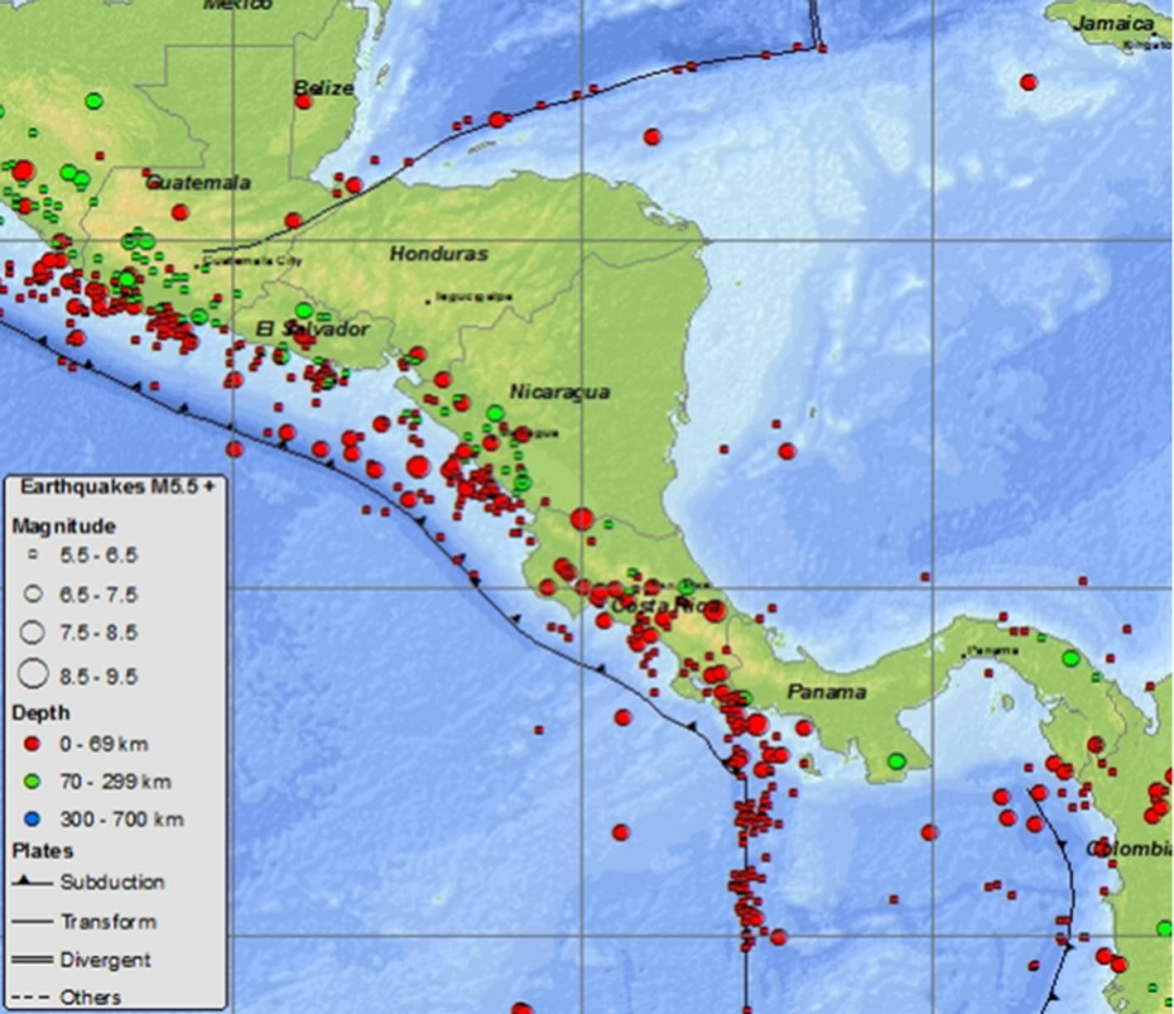
Epicentres of earthquakes of magnitude ≥ 5.5 in Central America since 1990. Source: http://earthquake.usgs.gov/earthquakes/world/central_america/seismicity.php

There is no doubt that the Central American republics to the north of Panama are tectonically very active: destructive earthquakes are frequent occurrences in Costa Rica, Nicaragua, El Salvador, and Guatemala, and the official crests of all these nations depict volcanoes. By contrast, seismicity during the instrumental period has been very much lower in Panama (Fig. 76). However, the choice of Panama over Nicaragua as the canal route seems to have established in the Panamanian psyche not so much that Panama is of lower hazard—or, more accurately, that destructive earthquakes in Panama are less frequent—than its neighbours, but rather that it is actually aseismic. During one of my visits, I encountered a magazine in my hotel room extolling the benefits of Panama as an ideal location for holidays or retirement, in which one of the headline claims was as follows: “*Panama has no hurricanes or major earthquakes. Panama is even blessed by nature. It is the only country in Central America that is absolutely hurricane-free. Panama also has none of the destructive earthquakes that plague its Central American neighbors. Your Panama vacation will never have to be re-scheduled due to natural events. Your property investment will always be safe*.” In light of this widely held view in Panama, it is perhaps not surprising that the implications of the paleoseismological studies were met with disbelief and denial.

The revised hazard estimates led to design motions for the new locks that posed a significant engineering challenge, and more than one of the consortia posed to bid for the expansion work withdrew when the seismic design criteria were revealed. Some people within the ACP were reluctant to accept the results and engineering consultants were engaged to obtain information to counter the findings of the geological and paleoseismological investigations, but these efforts were largely unsuccessful: one of the claims made was related to the lack of paleoliquefaction features (e.g., Tuttle et al. 2019), but the notion that such evidence would be preserved in a tropical environment with very high precipitation rates is naïvely optimistic.

**Fig. 77:**
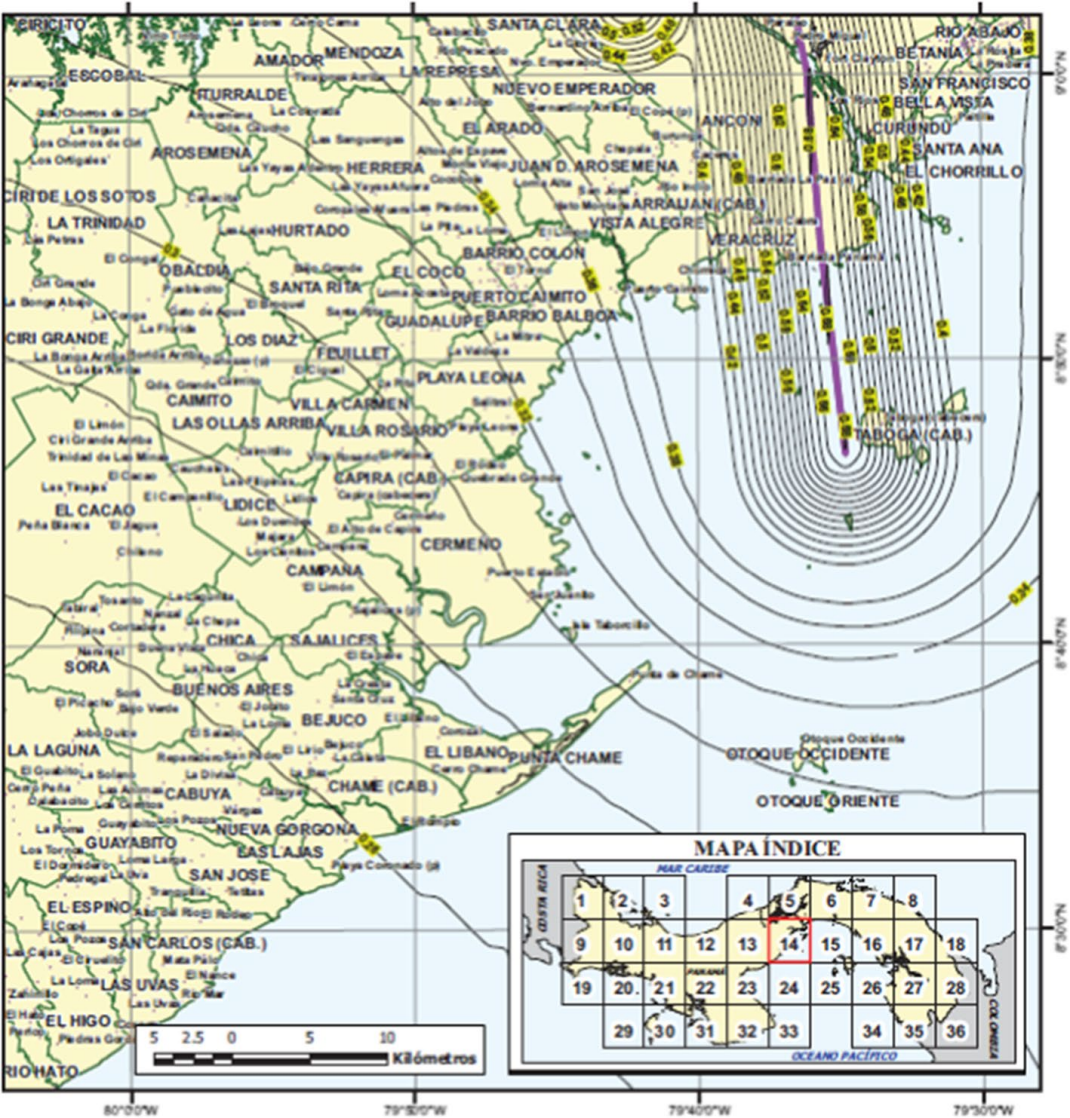
Map of 1-s spectral accelerations for south-central Panama from the REP-2014 structural design code; the purple line is the Pedro Miguel fault

The concerns about the implications of the Pedro Miguel fault extended beyond the canal because the fault is located only about 5 km from Panama City, a rapidly growing city with many high-rise buildings. Thanks to the efforts of some engineers from the ACP, the 2004 building code for Panama was revised in 2014 with a hazard map generated taking full account of this active structure (Fig. 77).

Nonetheless, the controversy persists. A paper by Schug et al. (2018) documented observations in the major excavations created for the approach channel for the new Pacific locks, and concluded that the Pedro Miguel fault was not present, countering the recommendation to design the dam that would contain the channel for up to 3 m of fault displacement. This has been taken up by some in Panama to call for a new revision of the hazard map and building code without the Pedro Miguel fault as a seismic source. However, while there may be uncertainty about the structure and location of the Pedro Miguel fault and its splays (which could question the fault slip specified for the dam design), the evidence from many other locations for the existence and recent activity of this fault is compelling and has important implications for seismic hazard; this impressive body of evidence is difficult to discount on the basis of observations at one location. The evidence that supports the existence of the fault is also consistent with an updated understanding of the tectonics of Panama, which rather than being a rigid microplate bounded by active offshore regions (e.g., Adamek et al. 1988), is now understood to be undergoing extensive internal deformation (Rockwell et al. 2010b), which could be expected to produce faults with multiple splays, some of which may have been exposed in the excavations studied by Schug et al. (2018). The debate regarding the Pedro Miguel is likely to continue for a while yet but with several major engineering projects underway in central Panama—including another bridge crossing the canal and the westward extension of the Metro system—it is an issue with far-reaching consequences.

### Testing PSHA

**Fig. 78:**
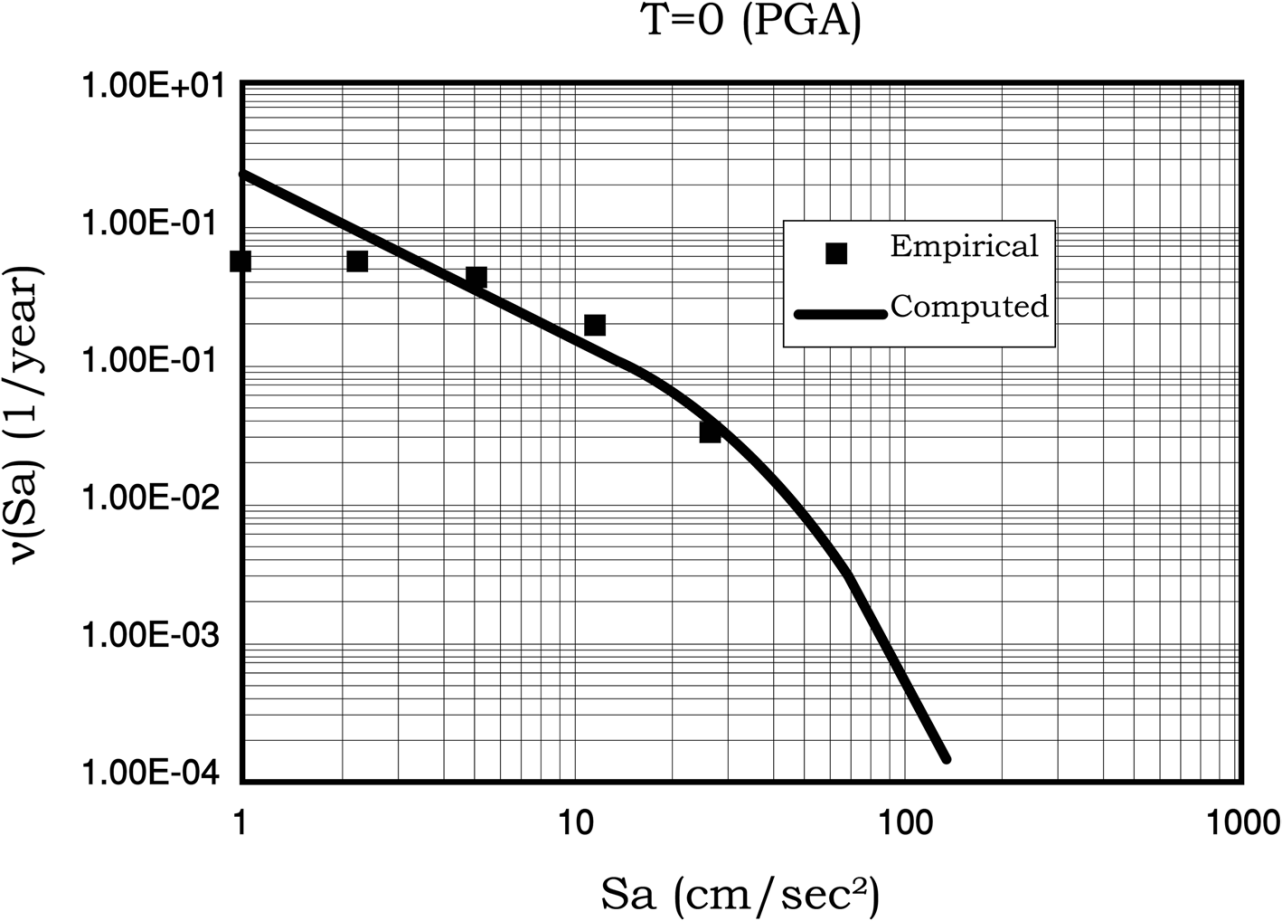
Comparison of hazard curve for PGA obtained from PSHA with empirical estimates of exceedance rates of PGA obtained from recordings at the same location (redrawn from Ordaz and Reyes 1999)

If our objective is to achieve acceptance of seismic hazard estimates, independent validation of the results by testing against data is clearly an attractive option. The most straightforward and unambiguous test is direct comparison of the hazard curve with the recurrence frequencies of different levels of ground motion calculated from recordings obtained at the same site over many years. Such empirical hazard curves have been generated for the CU accelerograph station in Mexico City by Ordaz and Reyes (1999), as shown in Fig. 78. The agreement between the empirical and calculated hazard is reassuring but it can be immediately noticed that the hazard curve is only tested in this way for return periods up to about 35 years, reflecting the time for which the CU station, installed in 1962, had been in operation. Fujiwara et al. (2009) and Mak and Schorlemmer (2016) applied similar approaches to test national hazard maps, rather than site-specific estimates, in Japan and the US, respectively.

In practice, statistically stable estimates of the return periods of different levels of motion require observation periods that are much longer than the target return period: Beauval et al. (2008) conclude that robust constraint of the 475-year hazard would require about 12,000 years of recordings at the site of interest. For the return periods of interest to safety–critical infrastructure—which for NPPs is on the order of 10,000 years or more—it becomes even more unlikely that sufficient data are available. Moreover, for genuine validation the recordings would need to have been obtained at the same site, which would require incredible foresight or extremely good luck to have had an accelerograph installed at the site several decades before the facility was designed and constructed.

Many researchers have tried to extend the period for which empirical observations are available by using intensities rather than ground-motion recordings to test seismic hazard estimates. While much longer periods of macroseismic observation are available in many regions of the world, the approach either requires the intensities to be transformed to ground-motion parameters using empirical relationships (e.g., Mezcua et al. 2013), which introduce large uncertainties, or by performing PSHA in terms of intensity (e.g., Mucciarelli et al. 2000). Hazard calculated in terms of intensity is of little use as engineering input and it is also difficult to establish whether intensity-based hazard is consistent with hazard in terms of response spectral accelerations, not least because the variability associated with intensity predictions is generally normal rather than the log-normal distribution of ground-motion residuals (which are therefore skewed towards larger absolute values). The simple fact is that we will likely never have the required data to genuinely validate seismic hazard estimates—and if we did, we could dispense with PSHA and simply employ the data directly. Testing of individual components of the hazard input models is often worth pursuing—see, for example, the proposal by Schorlemmer et al. (2007) for testing earthquake likelihood models—but our expectations regarding the degree of validation that is obtainable should be kept low. Oreskes et al. (1994) provide a sobering discussion of verification and validation of models in the Earth sciences, concluding that “*what typically passes for validation and verification is at best confirmation, with all the limitations that this term suggests*.” Oreskes et al. (1994) define confirmation as agreement between observation and prediction and note that “*confirmation is only possible to the extent that we have access to natural phenomena, but complete access is never possible, not in the present and certainly not in the future. If it were, it would obviate the need for modelling*.”

In the light of the preceding discussion, it is interesting—and to me, somewhat disturbing—that there has been a trend in recent years to use observational data not just to test PSHA results but also to modify them (and the adjustment, unsurprisingly, is generally downwards). The proposals are to use Bayesian updating to modify the hazard models—essentially to change the weights on logic-tree branches—using observational data (e.g., OECD 2015; Secanell et al. 2018). I should clarify that I have no fundamental objection to Bayesian methods or to their application to engineering seismology. Based on experience as an expert witness in a dispute involving extensive damage to a power plant caused by a large earthquake in southern Peru in 2001, where the closest ground-motion recording was obtained at 70 km, I have proposed a Bayesian approach to estimating the ground shaking levels at the site of interest from multiple datasets and modelling (Bommer and Stafford 2012). Without over-extending this discussion, I would raise two objections to Bayesian updating of PSHA input models: (1) the same data should not be used to develop and to test a model, so to apply such techniques requires a conscious decision to leave some data aside when developing the SSC and/or GMC models, which runs contrary to the principle of establishing the best-constrained models possible; (2) down-weighting or even removing logic-tree branches based on short-term observations will influence the long-term hazard estimates in ways that are difficult to justify. Bayesian modification of PSHA input models has been largely proposed and promoted by the French nuclear industry and may well be a response to regulatory transition in that country from being one of the last bastions of DSHA to a gradual adoption of probabilistic approaches. Fortunately, the approach has gained little traction globally and has not been widely adopted.

**Fig. 79:**
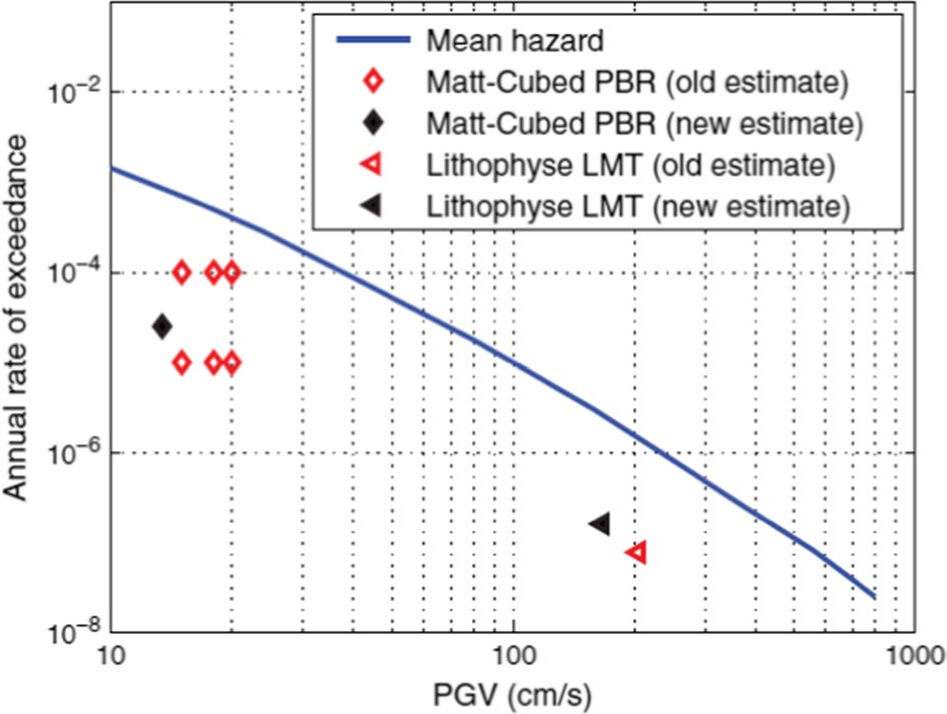
Mean hazard curve at Yucca Mountain in terms of PGV, compared with unexceeded ground motions inferred from precariously balanced rocks (PBR) and lithophyse (LMT is lower mean tuff properties for these fragile geological features) using different approaches for calculating their fragility (Baker et al. 2013)

There is, however, one perfectly legitimate use of empirical data to limit hazard estimates in PSHA, and it corresponds, paradoxically, to cases of ground motions at very long return periods and very high amplitudes of shaking. During the early decades of strong-motion recording (from 1933 to the mid-1960s), expectations of the largest possible motions were strongly correlated with the maximum recorded amplitudes (Strasser and Bommer 2009). Nowadays, large-amplitude recordings (PGA > 1* g*, PGV > 100 cm/s) are no longer a surprise—and due to spatial variability, we should probably expect to see even larger amplitudes. However, there are likely to be physical bounds on the levels of motion that can be recorded in earthquakes, due to three factors: (1) the most intense seismic radiation that can emanate from the source of the earthquake; (2) the interaction of radiation from different parts of the source and from different travel paths; and (3) the limits on the strongest motion that can be transmitted to the surface by shallow geological materials (Bommer et al. 2004b). The need to impose physical constraint on very low probability hazard estimates was highlighted by the PSHA for the Yucca Mountain nuclear waste repository in Nevada (Stepp et al. 2001). Due to the long design life of the post-closure facility and the need for very low probability of failure, the hazard calculations were extended to annual exceedance frequencies of 10^–8^, leading to ground motion levels that very likely exceed physical limits (Andrews et al. 2007). Physical limits on the levels of ground shaking that could occur at Yucca Mountain were estimated from the accelerations that would have toppled precariously balanced rocks (e.g., Brune 1999) and other fragile geological features that can be reliably aged, thus allowing the hazard estimates to be capped (Baker et al. 2013; Fig. 79). Such geological indicators of limiting ground-motion amplitudes have since been used in seismic hazard assessments for the Diablo Canyon NPP in California (Rood et al. 2020) and other facilities (Stirling et al. 2021). Physical limits on ground motions related to the limited strength of near-surface deposits have been explored from the perspective of site response analyses and the maximum accelerations that can be transmitted (e.g., Pecker 2005).

### Inflated hazard assessments

In the discussions thus far, the primary concern has been with underestimations—deliberate or otherwise—of the seismic hazard, since this has obvious safety implications. However, severe overestimation of the seismic hazard at a given location can also have serious consequences, including rendering design and construction very challenging and even economically unviable in extreme cases. The case of high hazard estimates for Dubai and Abu Dhabi resulting due to the unproven West Coast fault was already discussed in Sect. 5.4. There have also been cases of inflated hazard estimates, where the resulting ground motions are not especially onerous but nonetheless there have been important consequences.

The first case concerns the Concud fault in Aragón, Spain (located a little over 200 km east of Madrid). In 2012, the Aragón government announced a project to build a new public hospital in the city of Teruel. Due to the location in the lowest hazard region of Spain (PGA < 0.04 g), the NCSE-02 building code did not require seismic design. However, Simón et al. (2016) published a study of the Concud fault, which is located some 400 m from the hospital site, from which it was inferred that it undergoes alternating periods of fast (0.53 mm/year) and slow (0.13 mm/year) slip, currently being in a fast slip phase. Following a very unconventional procedure, Simón et al. (2016) developed a linear recurrence relationship combining their geological data with regional seismicity data at lower magnitudes—referring to the concept of characteristic earthquakes but completely ignoring the model formulation proposed by Youngs and Coppersmith (1985)—and then used this to determine the earthquake magnitude with a 500-year recurrence interval (a completely erroneous attempt to determine the hazard for the 475-year return period specified in the Spanish building code), yielding a result of magnitude 5.33. Empirical prediction equations are then used to estimate an intensity of VII (actually 7.4) and this was then transformed to a PGA via an outdated empirical correlation model between these two parameters (Simón Gómez et al. 2014). This updated hazard assessment caused the hospital construction to be suspended.

**Fig. 80:**
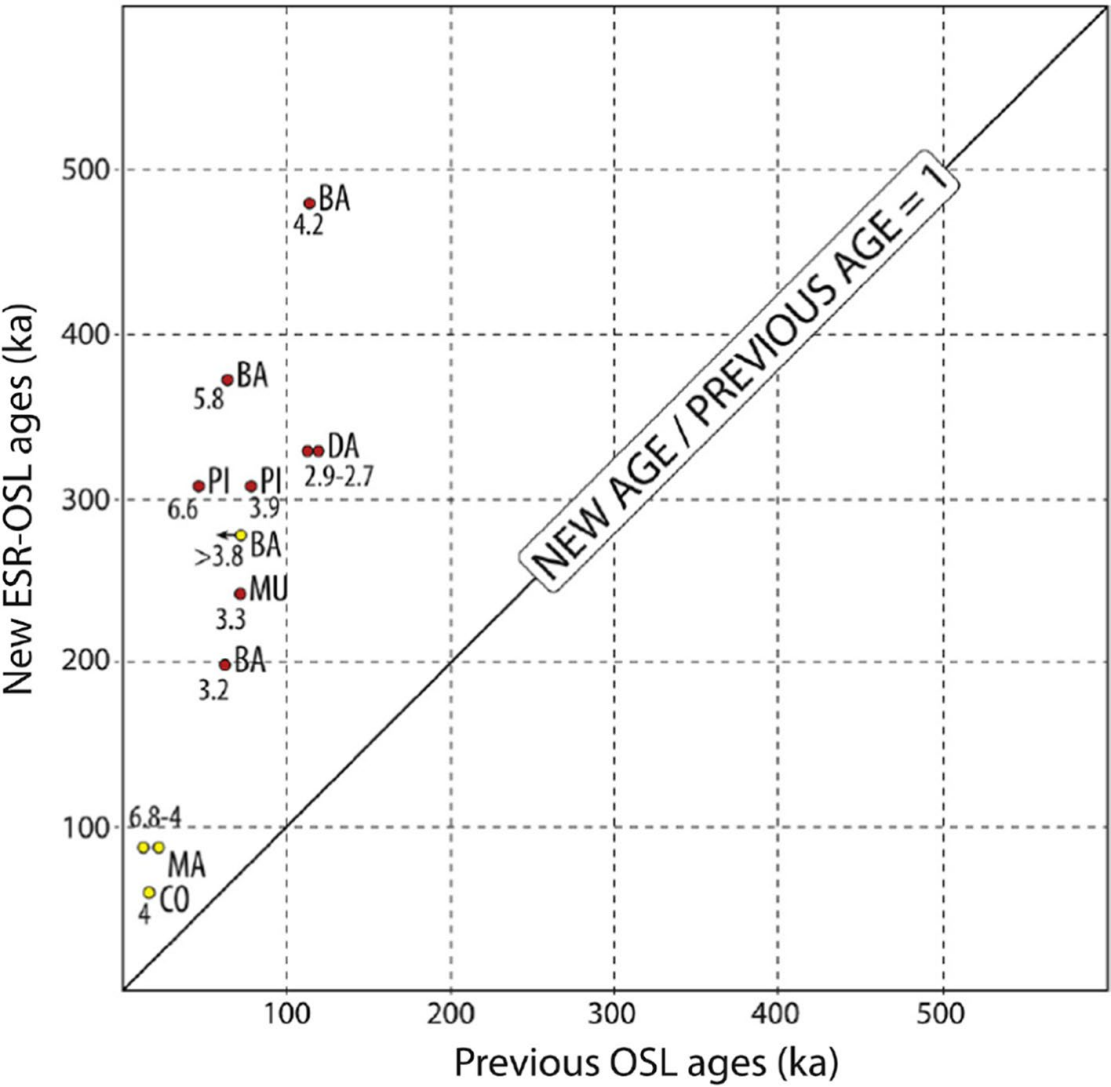
Comparison of new OSL ages for samples along the Concud-Teruel fault system compared with those from the laboratory that provided the results underpinning the Simón et al. (2016) study; the numbers indicate how much longer are the new ages (Gutiérrez et al. 2020)

Subsequent paleoseismological investigations, conducted for the Trillo NPP site as part of the SSHAC Level 3 PSHA for all nuclear power plants in Spain (Sect. 6.5), concluded that the slip rate and seismogenic potential of the Concud fault were significantly lower than inferred by Simón et al. (2016). The key contributing factor to the exaggerated hazard estimate were results of OSL (optically stimulated luminescence) dating performed by a laboratory in Madrid that were found to yield vastly underestimated ages for the deposits displaced by the Concud fault (Fig. 80). The design basis for the Teruel hospital was finally based on the 475-year PGA of 0.05* g* based on the most recent seismic hazard map for Spain (IGN 2013a) and without explicit consideration of the Concud fault, but the start of construction was delayed until 2019 due to the exaggerated hazard estimate.

**Fig. 81:**
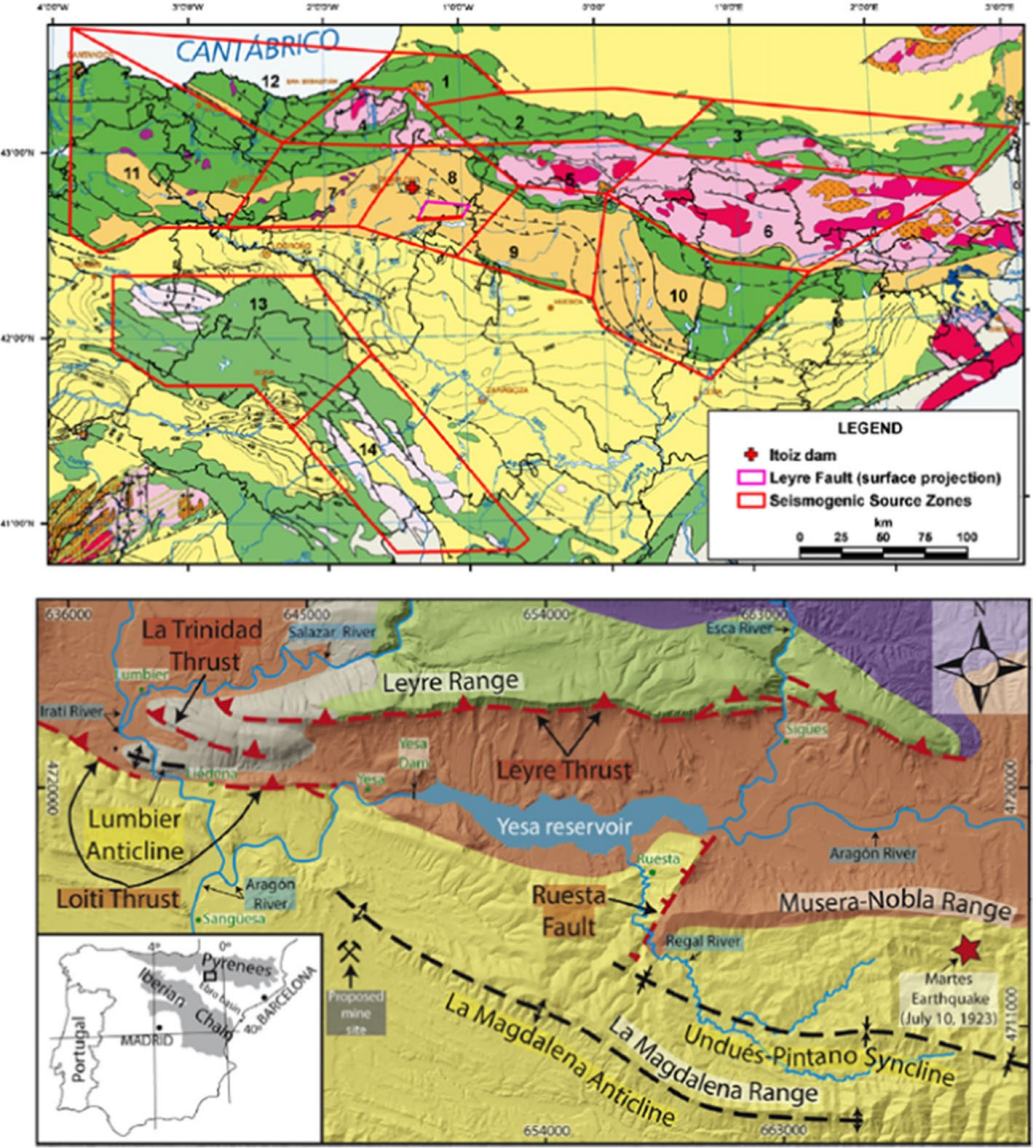
Upper: Seismic sources defined in the PSHA for the Itoíz dam (red cross) by García-Mayordomo and Insua-Arٞvalo (2011), the red polygons showing seismic source zones and the pink quadrilateral showing the surface projection of the Leyre fault; lower: faults, including the Leyre thrust, in the vicinity of the Yesa reservoir (Carbonel et al. 2019)

Another case of overestimated fault activity impacting on engineering projects concerns the Leyre fault in the western Pyrenees. In September 2004, during filling of the Itoíz reservoir located about 20 km north of the fault, a sequence of moderate earthquakes occurred, prompting a request from the Spanish Ministry of Environment for a PSHA for the Itoíz dam site, which was carried out by the Spanish geological survey (IGME). Field work undertaken by IGME concluded that none of the faults in the region of the dam showed evidence of Quaternary displacements with the exception of the Leyre fault, which was considered capable of producing earthquakes as large as **M** 6.6 ± 0.26 with a recurrence interval of 6,000 years. García-Mayordomo and Insua-Arévalo (2011) conducted a PSHA with area source zones and the Leyre fault as a distinct source (Fig. 81), noting that “*Even though the recent activity of the fault is still under investigation, it was decided to take a conservative approach and consider it in the hazard calculations*”. The result of the PSHA was a 1,000-year PGA at the Itoíz dam site that was twice the acceleration specified in the NCSE-02 building code. However, the new hazard model for the Itoíz dam site had a collateral impact regarding the design of the Yesa dam, located just 2.5 km south of the fault (Fig. 81), which at the time was being raised from a height of 78 m to 108 m to double the capacity of the reservoir. The indication of a highly active fault so close to the dam raised doubts regarding the project to increase the dam height. However, subsequent investigations of the thrust (i.e., shallow-dipping reverse) fault by Carbonel et al. (2019) demonstrated that the Leyre fault is not active, highlighting the fact that offsets on faults are not necessarily indicators of seismogenic activity since they can also result from non-seismogenic processes such as evaporite dissolution, salt movement, and landslides. Moreover, fault plane solutions for earthquakes in the region—including the Martes earthquake of July 1923 (Fig. 81b)—consistently show normal-faulting mechanisms rather than reverse (Stich et al. 2018). Another controversial Spanish fault features prominently in the case history presented in Sect. 12.3.

**Fig. 82:**
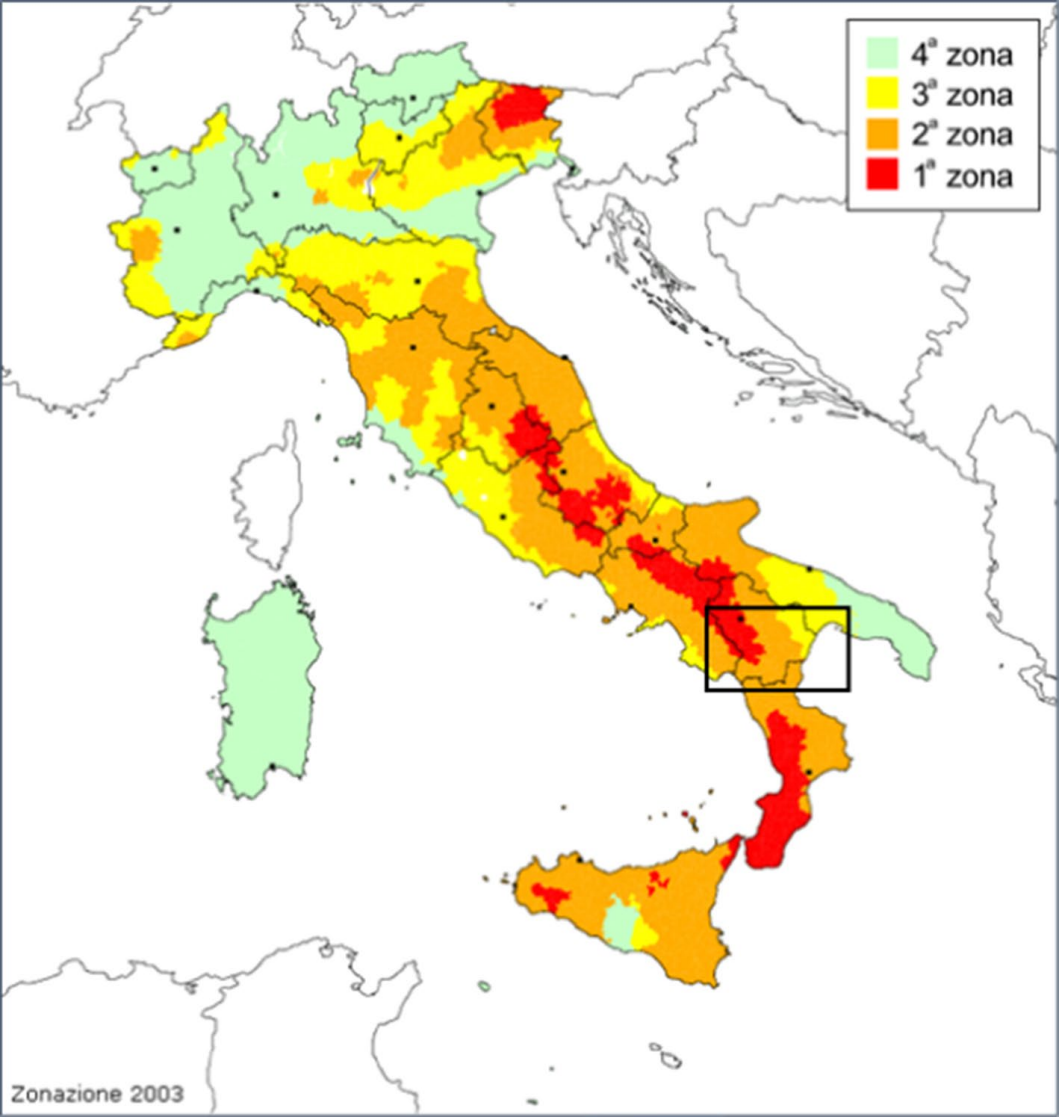
Proposed national hazard zonation of Italy based on the April 2004 (courtesy of Max Stucchi and Valentina Montaldo); the rectangle shows the area of Fig. 83

**Fig. 83:**
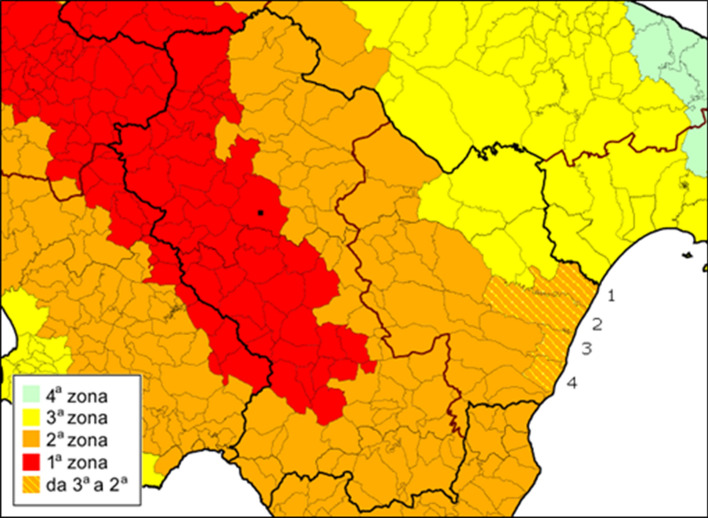
Detail of the revised national hazard zonation showing the region of Basilicata and the four municipalities upgraded from Zone 2 to Zone 3 (courtesy of Max Stucchi and Valentina Montaldo); municipality no. 3 is Scanzano Jonico

The final case concerns the new Italian hazard map discussed earlier in Sect. 5.4.2. The final zonations—from Zone 4 to Zone 1 in order of increasing hazard—were assigned at the level of municipalities, requiring that for any municipality crossed by a PGA contour defining the boundaries between one zone and another, a choice was made regarding which zone to assign. A national zonation was proposed (Fig. 82) but under legislation that devolves a degree of power to the regions of Italy, each region could move municipalities into an adjacent zone at their own discretion. Several municipalities were consequently downgraded to lower hazard: 63 in the Province of Trento were moved from Zone 3 to Zone 4 and six in Sicily were assigned to Zone 2 instead of Zone 1. In the region of Basilicata, however, just before the deadline for finalising the national hazard zonation, four municipalities were raised from Zone 2 to Zone 3 (Fig. 83). One of these was the municipality of Scanzano Jonico, which had been designated by the Council of Ministers as the selected site for a national repository for high and intermediate nuclear waste (Peruzza and Pessina 2016). Legislation regarding the waste repository forbid the construction of such a facility in hazard Zones 3 and 4, hence the deft upgrading of Scanzano Junico resulted in the automatic cancellation of the waste repository project.

Part II: Induced Seismicity

In Part I, I have attempted to demonstrate that the state of practice in seismic hazard analysis has undergone significant evolution, particularly with regards to handling uncertainty. Technical developments have increased our ability to build well-constrained seismic source and ground-motion models, and to incorporate the associated uncertainties in a transparent and tractable manner. Procedures have also been proposed, and iteratively refined through lessons learned from practical implementation, for conducting multiple-expert hazard assessments to capture the centre, body, and range of technically defensible interpretations of the available data and models. In this second part of the paper, my objective is to explore how these technical and procedural developments can be adapted to induced seismicity.

Part I has also shown that despite the significant advances made in seismic hazard analysis, acceptance of hazard assessments by all stakeholders (regulators, owners, operators, and the general public) is by no means automatically assured. The challenge of achieving acceptance of earthquake hazard and risk assessments for induced seismicity is much greater, because the risk is viewed as an imposed rather than natural threat by those affected, and also because the industrial processes causing induced seismicity are often the subject of controversy in themselves. However, for rational management of induced seismic risk that balances the potential dangers with the benefits of the industrial processes causing the seismicity, such acceptance is vital. The degree to which objective assessment of induced seismic risk is being both achieved and effectively communicated is a key focus of the ensuing discussions.

## Earthquakes of anthropogenic origin

**Fig. 84:**
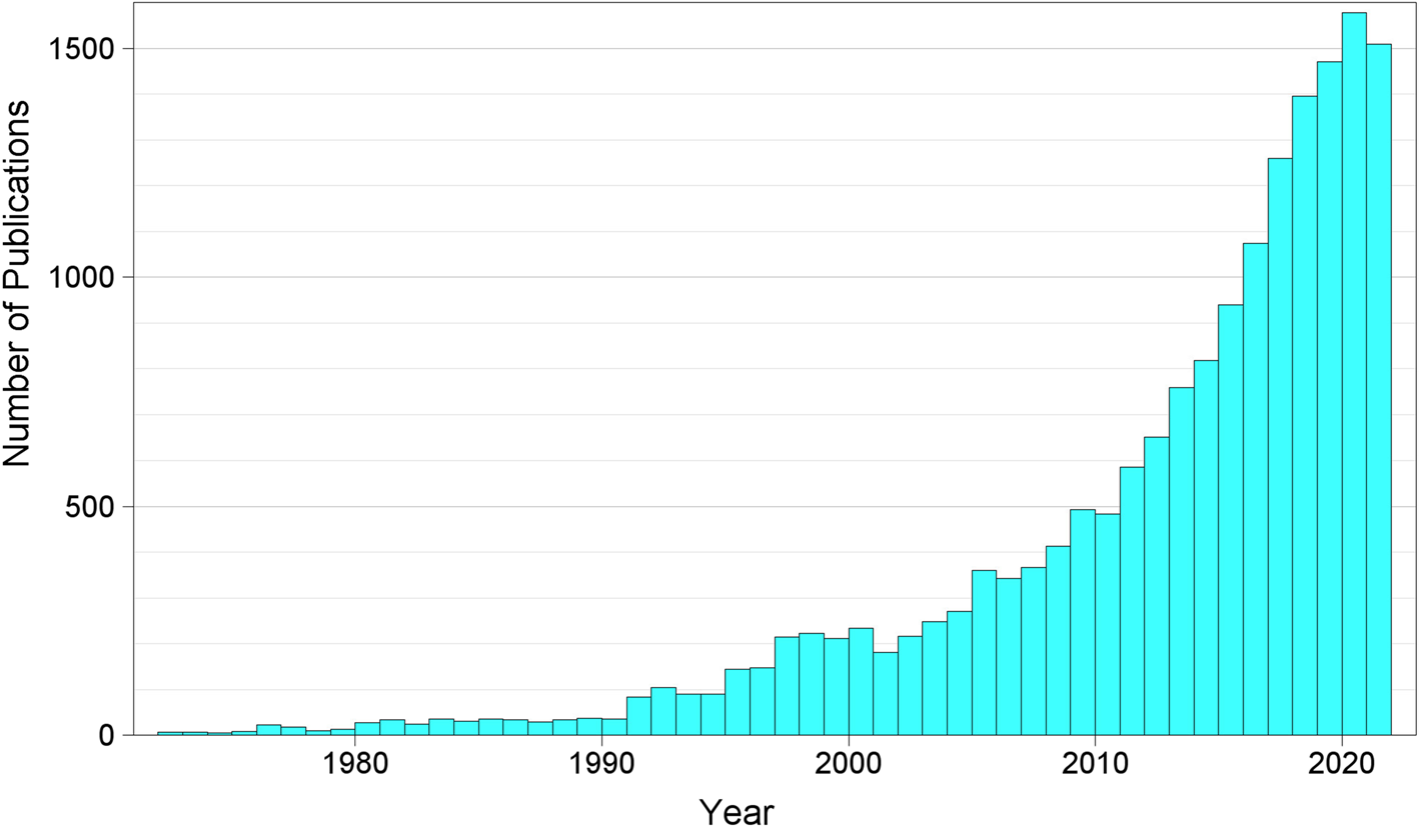
Number of publications per year from 1972 to 2021 listed on Web of Science with topic ‘induced seismicity’ or ‘induced earthquakes’; the data for 2021 may not be complete

Earthquakes associated with human activities are not a very recent phenomenon, but induced seismicity has attracted a great deal of attention in recent years, both in the media and in academic research (Fig. 84). The interest has been driven in large part by significant increases in seismic activity in certain regions of the world—in particular in Oklahoma and neighbouring states (Keranen et al. 2014; McNamara et al. 2015) and in the Western Canadian Sedimentary Basin (WCSB; Atkinson et al. 2016a)—that have been linked to hydrocarbon production. There can be little doubt that the general controversy that surrounds the process of hydraulic fracturing, or fracking, has also served to raise the profile of induced seismicity in general, even though fracking has not been the major contributor to induced seismicity.

My focus in this paper is to address induced seismicity from the perspective of seismic risk, exploring how advances in the treatment of natural seismicity can be adopted and adapted to induced earthquakes. Before entering into discussions of the assessment (Sect. 9) and mitigation (Sect. 10) of induced seismic risk, this section provides a brief introduction to the basic concepts and definitions, as well as discussing the very important question of how induced and natural earthquakes can be distinguished.

In view of Fig. 84, which was inspired by a similar image presented by Professor Stefan Wiemer at the Third Schatzalp Workshop on Induced Seismicity held in Davos in March 2019 (the presentations and posters from which can be accessed at www.seismo.ethz.ch/en/research-and-teaching/schatzalp-workshop/), I need to clarify that in this paper I make no attempt to undertake a comprehensive review of the vast literature that now exists on the topic (to keep up with all the literature would now require one to read four or five papers a day, only resting on Sundays!). I do refer to many of the landmark papers that have been published in this field—and a number of my own papers too since I am presenting my own perspectives on this topic—but several readers are likely to consider that I have missed some key citations, for which I can only apologise. I would, however, point the reader to excellent overview and review papers that have been published and which help one to navigate through the enormous body of published literature (e.g., Suckale 2009; Ellsworth 2013; Davies et al. 2013; Keranen and Weingarten 2018; Foulger et al. 2018), and I trust that new overview papers will appear in due course to maintain and update the condensed road maps for those seeking to extract the essence from the ongoing research in this field.

### Induced and triggered earthquakes

Seismographs record the passage of waves travelling through the Earth’s crust and the seismograms of these signals can be used to locate the source of the waves and the energy released at the source, as measured by magnitude scales. The recorded waves may originate from sources other than earthquakes, including natural phenomena such as volcanic activity and landslides (e.g., Hibert et al. 2014a, 2014b) and artificial energy sources such as explosions, sonic booms (e.g., Cates and Sturtevant 2002) and even light aeroplane crashes (Aspinall and Morgan 1983). As mentioned in the opening paragraph of this paper, seismograph monitoring of nuclear explosions is a key element in maintaining treaties banning the testing of nuclear weapons. The explosions most commonly recorded are quarry blasts, which need to be removed from the earthquake catalogue before calculating recurrence parameters (e.g., Gulia and Gasperini 2021). All such sources of seismic waves fall outside the focus of this paper, which is about earthquakes that occur due to abrupt slip of geological faults, in the same way as the natural or tectonic earthquakes discussed in Part I.

Mining has long been recognised as an anthropogenic source of seismicity (e.g., Cook 1976; Klose 2013), especially in regions of deep mining such as South Africa. However, the seismic signals generated by mining activity are often the result of collapses and rock bursts rather than the rupture of pre-existing geological faults. Another long-recognised source of seismicity is the impounding of deep reservoirs (e.g., Simpson 1976; Simpson et al. 1988). In the case of reservoir-induced seismicity, the earthquakes occur in the same way as tectonic events through fault rupture, the primary mechanism triggering the fault slip being an increase in pore pressure due to infiltration of water driven by the hydraulic gradient created by the reservoir.

**Fig. 85:**
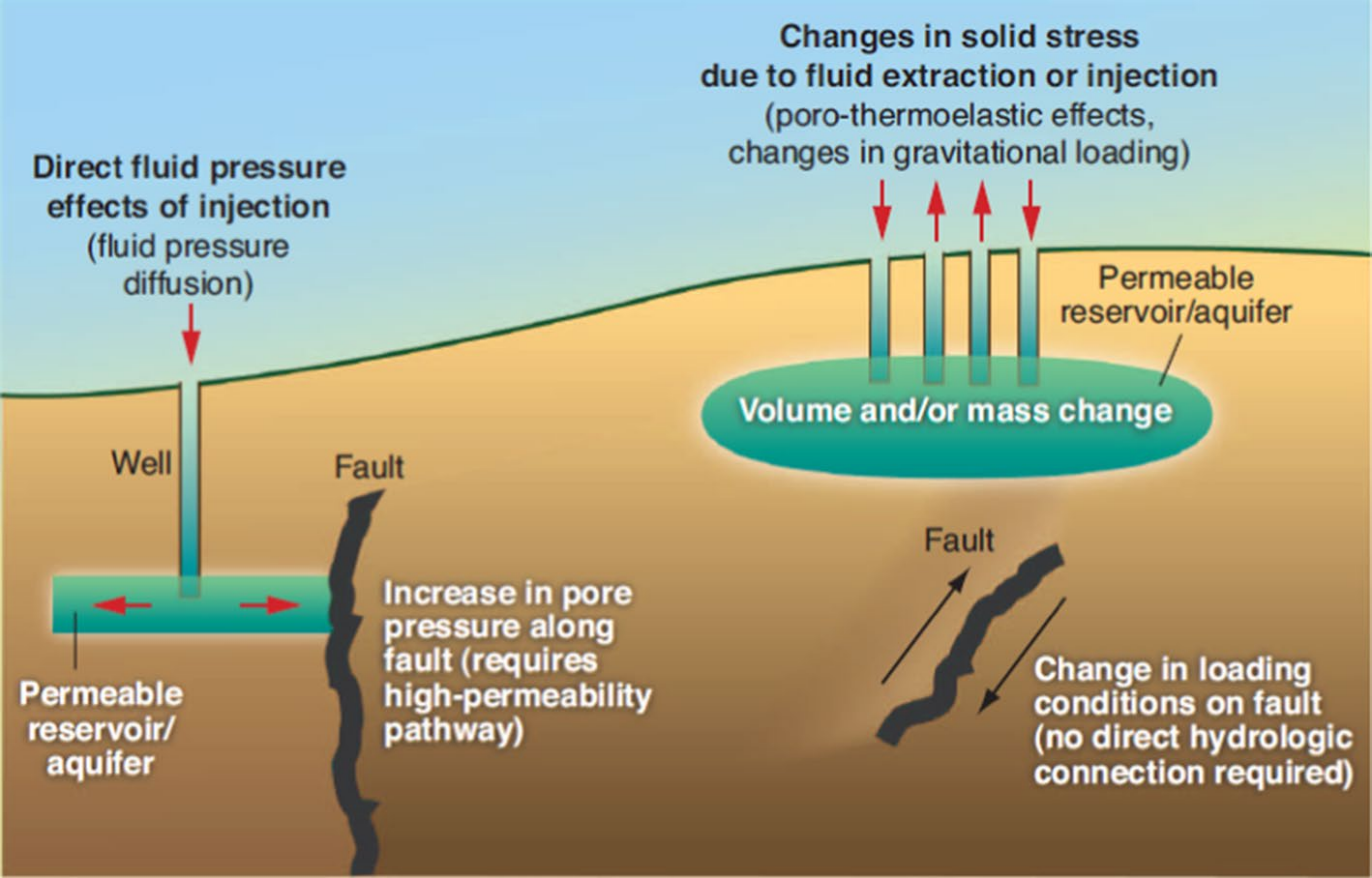
Illustration of the mechanisms of inducing seismicity through fluid injection leading to increased pore pressure on a fault (left) and by fluid injection or extraction changing the shear and normal stresses on a fault (right) (Ellsworth 2013)

The primary focus in recent years has been related to seismicity induced by the injection or extraction of fluids (Fig. 85), which includes a wide range of industrial processes, nearly all of which are related, in one way or another, to energy supply (NRC 2013). The fluid extraction and injection processes that have been associated with earthquakes include the following: conventional hydrocarbon production (e.g., Suckale 2010); wastewater injection (e.g., Ellsworth 2013); hydraulic fracturing for production of unconventional hydrocarbon reservoirs (e.g., Atkinson et al. 2020; Schultz et al. 2020a); enhanced geothermal systems (e.g., Majer et al. 2007); and carbon capture and storage (e.g., Verdon and Stork 2016).

There are cases where seismicity has clearly been associated with fluid extraction, including conventional gas extraction, such as in the Lacq field in southwest France (Bardainne et al. 2008), but the associations have not always been unambiguous. The destructive **M** 5.1 2011 earthquake that struck Lorca in southeast Spain has been attributed to extraction of groundwater (González et al. 2012). McGarr (1991) postulated that three major earthquakes in California—**M** 6.5 Coalinga in 1983, **M** 6.1 Kettleman North Dome in 1985, and **M** 5.9 Whittier Narrows in 1987—were all due to oil extraction, following the mechanism illustrated on the right-hand side of Fig. 85. However, this hypothesis has not been widely accepted and those earthquakes are not generally viewed as induced events.

Cases of induced seismicity associated with fluid injection are far more common and the association of the earthquakes with the injections is frequently unambiguous. The first very clearly identified case of seismicity induced by fluid injection was at the Rocky Mountain Arsenal in Denver, Colorado, where waste fluid from weapons production was injected in a 3.6 km disposal well. The injections began in March 1962 and within a few months gave rise to numerous seismic events, the larger of which were felt by local residents (Healy et al. 1968). The injections were finally suspended in February of 1966, but seismicity continued for some time afterwards, the largest event (**M** 4.8) occurring in August 1967. This prompted an experiment conducted between 1969 and 1980 in the Rangley oilfield in northwest Colorado as a collaboration between the USGS and Chevron, to explore the relationship between* in situ* stress, fluid injections, and fault slip potential based on friction coefficients measured on laboratory tests of rock samples (Raleigh et al. 1976). The experiments confirmed that the faults slipped when the pore pressure reached the estimated level required to overcome the shearing resistance.

**Fig. 86:**
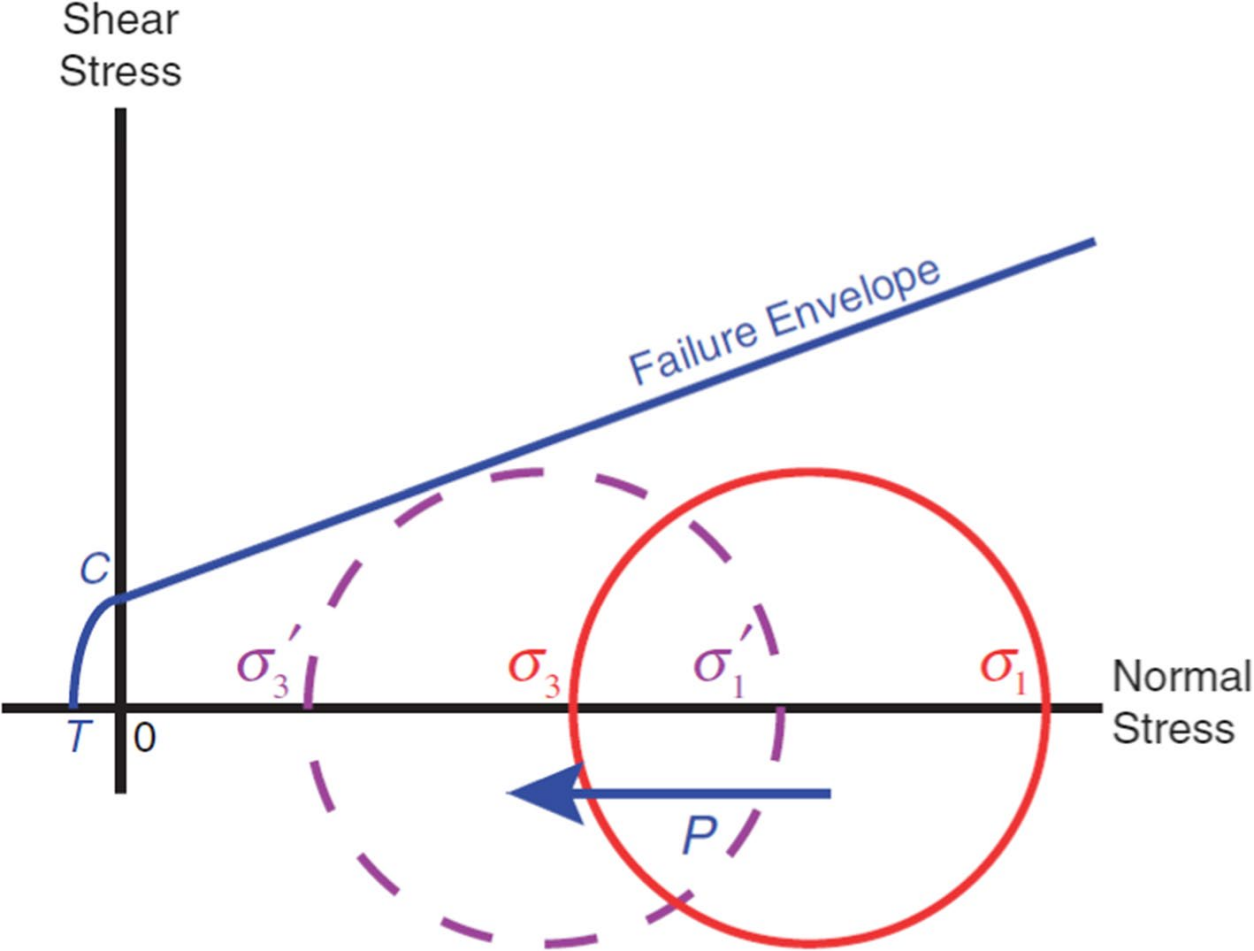
Mohr’s circle diagram illustrated how elevation of pore pressure, leading to a reduction in effective stresses, can bring a fault to failure (Rubinstein and Babaie Mahani 2015); ${\upsigma }_{1}$ and ${\upsigma }_{3}$ are the maximum and minimum normal stresses, and the symbols with primes correspond to the effective stresses

The increase in pore pressure on a fault that can result from fluid injection reduces the effective normal stress acting on the fault, which in turn lowers the resistance to shearing. This is illustrated by the Mohr’s circle diagram in Fig. 86. There are several mechanisms through which the pore pressure within the fault can be raised, the most rapid being direct injection into the fault plane itself, as is believed to have happened in the Pohang enhanced geothermal project that has been linked to a destructive earthquake of **M** 5.5 (Lee et al. 2019). The injected fluid can also migrate through existing networks of fractures connecting the well to the fault (Igonin et al. 2021). Stresses can also be transferred statically through poro-elastic deformations; this mechanism can act in unison with dynamic fluid pressure transfer (Kettlety and Verdon 2021). Another mechanism that has been identified for stress transfer is through aseismic fault slip resulting in increased stress on another fault (Bhattacharya and Viesca 2019).

Regardless of the specific mechanism, the changes in pore pressure or stress due to the injections are generally small in comparison with existing stresses within the Earth’s crust. Consequently, earthquakes will generally only occur on faults that are already critically stressed, meaning that they are already close to rupture as a result of tectonic stresses and the fact that the fault is favourably orientated with respect to the existing stress field. Viewed from this perspective, the timing of the earthquakes may be controlled by the anthropogenic activities, but it would not be correct to say that the earthquakes are caused by the injections since it is the existing state of stress on the fault that is ultimately responsible for producing an earthquake. Very small-magnitude events, which are usually referred to as micro-seisimicity and are only be detected by sensitive downhole seismic instruments (e.g., Maxwell et al. 2010), may be properly referred to an induced seismicity, but the larger events—and particularly those that are felt, and which generate societal and regulatory concern—are more correctly described as triggered earthquakes. Dahm et al. (2013) defined triggered earthquakes as follows: “*Triggered earthquakes occur on favourably oriented faults in agreement with the existing regional or local background stress field and geological structure. Their magnitude is not controlled by human-induced stress changes, which only cause the event nucleation. However, the human-induced stress changes have the potential to advance failure on an active fault that is prone to natural failure in the future*.” However, it is common practice to refer to such earthquakes as induced seismicity, and this convention is also followed herein. One argument in favour of using the terminology of induced seismicity, as pointed out by Rubinstein and Babaie Mahani (2015), is that the term triggered earthquakes is already used in seismology to describe earthquakes that result from stress transfer caused by one fault rupture to another fault (e.g., Stein et al. 1997).

In closing this discussion, a point to stress is that induced seismicity can be caused by a variety of anthropogenic processes. While fracking is one such process, on a global scale it is neither the primary cause of induced earthquakes nor the cause of the largest induced earthquakes, even though the media often portrays it as the main cause of induced seismicity. Schultz et al. (2020a, b) note that barely 1% of hydraulic fracturing wells around the world have caused induced seismicity. Hydraulic fracturing appears to be the primary cause of induced earthquakes in the WCSB, but elsewhere this is not the case. In the Oklahoma, Kansas and Texas, for example, induced seismicity is mostly the result of saltwater injection—when crude oil is extracted from the ground it is generally accompanied by saltwater, sometimes in even larger quantities than the oil itself such as in the Rubiales and Quifa fields in Colombia (Molina et al. 2020), which is separated and usually injected into disposal wells. Rubinstein and Babaie Mahani (2015) report that only 10% of the saltwater injected in Oklahoma is produced by hydraulic fracturing. However, the media still insists on making direct or insinuated connections to fracking even when it is not remotely involved. By way of illustration, following the 2018 Newdigate earthquakes in southern England—discussed further in Sect. 8.2—Richard Selley, Emeritus Professor of Petroleum Geology at Imperial College London and resident of the affected area—was interviewed on site for television news. Professor Selley’s opening statement was to clarify that there were no hydraulic fracturing operations in the area and therefore no connection of the seismicity with fracking; the interview was broadcast in the evening news in its entirety, minus this opening statement.

### Distinguishing induced from natural earthquakes

The importance of discriminating between natural and induced earthquakes cannot be overstated, for three reasons. Firstly, for the science of understanding the processes by which earthquakes are induced and the factors that influence these processes to advance, the starting point must be the clear identification—to the extent possible, since ambiguity will exist in some cases—of earthquakes whose occurrence is related to an anthropogenic activity. Analyses that correlate tectonic earthquakes with industrial processes would only serve to create confusion. Secondly, reliable identification of induced earthquakes is fundamental to developing confidence in the management of the associated risk: classifying induced seismicity as natural will aggravate public mistrust if the classification is subsequently proven wrong, and incorrectly classifying earthquakes as induced will lead to unwarranted concern. Finally, if measures are to be taken to mitigate the risk due to induced seismicity through control of the hazard (see Sect. 10.1), the efforts are likely to be in vain if the earthquakes are, in fact, of tectonic origin. And the inevitable failure of the mitigation measures would thus undermine confidence in the possibility of controlling induced seismicity.

There are many cases in which the induced nature of observed seismicity is unambiguous, especially when a large number of earthquakes suddenly occur in a region of little or no tectonic seismicity, such as the case of the Groningen gas field in the Netherlands (see Sect. 12.4). Another very clear case is the observed seismicity in the Quifa and Rubiales oilfields in Colombia mentioned above, which are located in a region of very low natural seismicity and where there are very pronounced spatial and temporal correlations of the observed earthquakes with the massive saline water re-injections (Gómez Alba et al. 2020; Molina et al. 2020). When the earthquakes occur in a region where tectonic seismicity is also observed, distinguishing induced events can become more challenging and it becomes necessary to identify clear correlations between the observed seismicity and parameters that characterise the injections and, in some cases, hydrological and/or geological factors (e.g., Oprsal and Eisner 2014; Goebel et al. 2015; McClure et al. 2017; Hincks et al. 2018; Grigoratos et al. 2020).

**Fig. 87:**
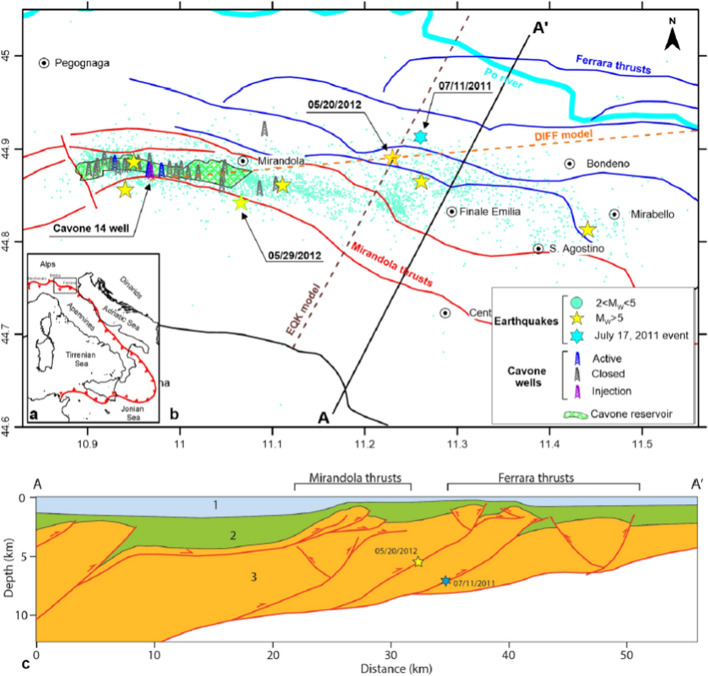
**a** Location maps showing main thrust alignment in Italy; **b** map of the area of the 2012 Emilia-Romagna earthquakes, with epicentres shown by light turquoise circles (M < 5) and stars for events of M ≥ 5, the largest two events outlined and with date labels, and the location of Cavone oil field and the production and injection wells; **c** cross-section showing the thrust faults corresponding to blue and red lines in (**b**). The blue star in (**b**) is an event of **M** 4.5 that occurred in July 2011 (Albano et al. 2017a)

Ultimately, the goal would be to determine whether the pore pressure and/or stress changes on the fault or faults that produced the earthquakes could have been caused by the fluid injections. Since pressure measurements on the faults are generally not available, the determinations usually require the use of hydrological and geomechanical models to represent the fluid pressure propagation and the response of the crustal rocks to the pressure changes. Dahm et al. (2015) developed an approach that is based on calculation of the geomechnical perturbation due to oil extraction in order to determine whether the location and mechanism of an induced earthquake is consistent with the pressure changes associated with hydrocarbon production. By comparing these stress changes with the long-term rate of stress increase due to tectonic processes, the approach of Dahm et al. (2015) allows the probability of the earthquake being induced to be calculated. The method was applied to three earthquakes that occurred close to hydrocarbon fields, the largest of which was the Emilia-Romagna earthquake of 20 May 2012. This **M** 6.1 earthquake was followed by several aftershocks, the largest of which occurred on 29 May 2012 with **M** 5.9, these two largest events resulting in extensive damage and 27 fatalities. The main aftershock and several of the smaller aftershocks occurred close to the Cavone oil field (Fig. 87).

Pezzo et al. (2013) concluded that the earthquake sequence was consistent with the long-term seismicity of the region and Caputo et al. (2012) excavated paleoseismological trenches following the earthquake, confirming that previous sequences of large earthquakes had occurred in the same area. The location of the 29 May event close to the Cavone oil field seems to have been the result of stress transfer due to the main shock on 20 May (Ganas et al. 2012; Pezzo et al. 2013); the main shock was located about 18 km away from the field. Although the western part of the aftershock distribution partially coincided with the Cavone field, none of the early papers on the source characteristics and rupture mechanism of the earthquakes even mentioned the oil field let alone a possible causative relationship of the earthquakes with hydrocarbon production. Nonetheless, in December 2012, the Italian Civil Protection Department formed, at the request of the President of the Emilia-Romagna region, an international panel of experts to investigate a possible connection between the oil fields and the seismic sequence. Given that the sequence began with a mainshock at an appreciable distance from the oil field and triggered a sequence of aftershocks that propagated towards the oil field, it may seem rather strange that the question was even asked. Dahm et al. (2015), who considered only the depletion of the reservoir and not the re-injection of salt water, concluded that there was a less than 1% probability that the earthquakes were triggered by hydrocarbon production. In a separate study, Albano et al. (2017a, b) modelled the impact of wastewater injections in the Cavone field and concluded that these would have caused stress changes on the fault associated with the mainshock rupture that would have been less than 10% of the stress transfer from the **M** 4.5 earthquake that occurred on an adjacent fault about 10 months earlier (Fig. 87c). Both Dahm et al. (2015) and Albano et al. (2017a) conclude, therefore, that the earthquake sequence was of tectonic origin and unrelated to the activities in the oil field.

From the perspective of seeking objective assessment of the hazard and risk due to induced seismicity, the story of the investigation by the international panel set up to investigate the possibility of the Emilia-Romagna earthquakes having been triggered by activities in the Cavone oil field is worthy of some brief discussion. The panel (ICHESE, International Commission on Hydrocarbon Exploration and Seismicity in the Emilia Region), issued its report in February 2014, concluding that “*the seismic process that began before May 20*^*th*^*, 2012 and continued with the sequence of earthquakes in May–June 2012 is statistically correlated with increases in production and injection in the Cavone oil field.*” The report states less emphatically that the mainshock of 20 May 2012 could have been triggered by fluid extraction and injection, and then makes several recommendations about the need for data to be provided by the operators and research that should be undertaken (and presumably funded). The report did lead to media reports that the earthquakes could have been caused by the operations in the oil field (e.g., https://www.thelocal.it/20140415/oil-drilling-may-have-triggered-deadly-italy-quakes/) and led the region of Emilia Romagna to impose a ban on all drilling. The subsequent scientific studies published by Dahm et al. (2015) and Albano et al. (2017a) have not vindicated the conclusions of ICHESE. Exactly how ICHESE came into being is not entirely clear but in a letter from the Italian Department of Energy (part of the Ministry of Economic Development) referring to the work of the Commission, it refers to the panel of experts by the name it was originally assigned: *Commissione Internazionale sull’esplorazione di idrocarburo e l’aumento della sismicità in Emilia del 2012* (International Commission on Hydrocarbon Exploration and Seismicity Increase in Emilia), which would seem to imply that the conclusion of the panel’s work was already foreseen in its initial title.

Detailed statistical, hydrological, and geomechanical analyses require extensive and detailed datasets, and require considerable time and effort to be executed. In many cases, an assessment of whether or not earthquakes are induced needs to be made rapidly and without recourse to such advanced approaches, for which reason simplified question-based approaches have a useful role to play. Such a screening scheme was proposed by Davis and Frohlich (1993) and this has been very widely applied in practice. The Davis and Frohlich (1993) approach consists of seven questions regarding the observed events and their relationship to the anthropogenic activity and the natural seismicity, if any, in the region:

1. Are these events the first known earthquakes of this character in the region?
2. Is there a clear correlation between injection/abstraction and seismicity?
3. Are epicentres near wells (within 5 km)?
4. Do some earthquakes occur at or near injection/abstraction depths?
5. If not, can known geologic structures channel flow to sites of earthquakes?
6. Are changes in fluid pressures at well bottoms sufficient to generate seismicity?
7. Are changes in fluid pressures at hypocentral distances sufficient to generate seismicity?

Each question is answered ‘yes’ or ‘no’, with five or more positive responses being interpreted as strong evidence for the earthquakes being induced; four positive answers suggests that there is a correlation, but it is ambiguous, whereas three or fewer ‘yes’ responses indicate that the earthquakes are unlikely to be induced. The scheme has undergone adaptation and improvements, the first being modifications by Davis et al. (1995) for application to fluid extraction processes. When considering historical cases, for which detailed pressure data will generally not be available, Frohlich et al. (2016) proposed modified questions and assigned values of 1 for ‘yes’ and 0 for ‘no’ plus 0.5 for ‘possibly’, with the assessment then based on the final sum of responses.

In April 2018, an earthquake sequence began close to the village of Newdigate in Surrey, UK, to the south of London, with several events reported by the British Geological Survey with M_L_ > 2 and the largest reaching M_L_ 3.2. The earthquake sequence, which continued into 2019, occurred a few kilometres away from two small oil fields, Brockham and Horse Hill. Concerns were raised by a small group of UK academics regarding a possible connection between the hydrocarbon fields and the seismicity. The forum selected by this group to share this view was a letter in *The Times* newspaper on 6 August 2018 arguing that a “*moratorium on drilling, re-injection and flow testing should be put in place immediately and remain in force until the records of fluid injection and local faulting activity have been comprehensively surveyed and interpreted, and the triggering mechanism for this quake cluster properly understood*.” By throwing this cat among the pigeons, the authors of the letter created a serious dilemma for the Oil and Gas Authority (OGA) that regulates hydrocarbon production in the UK—as well as potentially threatening the livelihoods of employees of the small companies operating these oil fields. The OGA convened a workshop with 40 invited participants, including the authors of the letter (www.ogauthority.co.uk/news-publications/news/2018/oga-newdigate-seismicity-workshop-3-october-2018/), the workshop report stating that, with one exception* “The workshop participants concluded that, based on the evidence presented, there was no causal link between the seismic events and oil and gas activity”* (the exception being the lead author of the letter). A study published subsequently in a mainstream seismological journal concluded that it was indeed unlikely that the earthquakes had been induced (Hicks et al. 2019), although dissenting views have been expressed in a chapter of a slightly obscure book (Westaway 2020) and on a blog (www.geosierra.com/news.html); the prevailing scientific view remains that there was no causative link between the oil fields and the earthquake sequence. The case raises an interesting question of the weight that should be given to different sources when classifying earthquakes as induced. The Human-Induced Earthquake Database (HiQuake; https://inducedearthquakes.org/; Foulger et al. 2018) lists the Newdigate earthquakes as induced; while the database acknowledges the conclusion of the OGA workshop on 3 October 2018, it cites three references in support of the events being induced: the lead letter writer’s presentation at that workshop and missives from the same individual, and colleagues, sent to the UK parliament and to Surrey County Council in 2019; the Hicks et al. (2019) paper is not cited.^1^ To my mind, any catalogue of induced earthquakes needs to indicate the relative confidence with which the classification is made, which in cases of controversy should clearly reflect when this is a minority view—and especially if the view is not supported by peer-reviewed publication. I was very surprised to find the 2007 M_L_ 4.3 Folkestone earthquake on the south coast of the UK (Sargeant et al. 2008)—very likely a similar event to the 1580 Dover Straits earthquake that many believe Shakespeare alluded to in *Romeo and Juliet*—is also classified as induced, the cause being attributed to coastal engineering. The Internet facilitates the dissemination of unfounded claims of anthropogenic causes for seismicity, particularly by those who already oppose the industrial activity in question, and if these are then picked up by mainstream media, can rapidly gain traction. A case in point was a tectonic **M** 6.5 earthquake in Botswana in April 2017, which was attributed the extraction of gas from coal (e.g., www.thegazette.news/latest-news/ckgr-gas-mining-linked-to-earthquakes/) although its natural origin has been clearly confirmed (Albano et al. 2017b).

The purpose of the apparent detour in the previous paragraph is related to the simplified discrimination scheme of Davis and Frohlich (1993). At the OGA workshop on the Newdigate earthquakes, the scheme was used by different speakers both to make the case for the earthquakes being induced and to demonstrate that they were most likely of natural origin. This prompted three participants at the workshop, including myself, to undertake a critical assessment of the Davis and Frohlich (1993) approach and to propose some modifications. The key shortcomings identified were as follows: (1) the scheme assigns zero whether there is no information to enable a response or whether the information available strongly suggests that the earthquakes are of natural origin; (2) the scheme gives equal weight to all questions even though some pieces of evidence may be much stronger indicators than others; (3) the final ‘score’ is not easily interpreted. In the proposed update of the scheme, Verdon et al. (2019) addressed issue (1) by assigning negative points for evidence supporting a conclusion of natural seismicity, issue (2) by allowing different maximum numbers of negative or positive points for the response of each question in accordance with how persuasive each item of evidence is perceived to be, and issue (3) by expressing the final outcome—the Induced Assessment Ratio (IAR)—as a percentage of the maximum possible score. To facilitate the interpretation of the IAR, Verdon et al. (2019) also defined a second index, the Evidence Strength Ratio (ESR), to reflect the information available for the assessment as a proportion of the information that would be ideally available. Applied to the Newdigate sequence with the information available in June 2018, the ESR scores for the Brockham and Horse Hill oil fields were 46% and 20% respectively, yielding IAR values of − 8% and 15% for the two fields. By October 2018, the ESR for both fields had increased to 87% and the IAR values were − 33% and − 79%, supporting the conclusion that the earthquakes were of natural origin.

### Identifying the true cause of induced earthquakes

Distinguishing induced from natural earthquakes is very important, but it is also important—for the same reasons expounded at the beginning of Sect. 8.2—to ensure that seismicity identified as being induced is attributed to the correct cause. This may not be straightforward in cases where several anthropogenic activities are underway in the same region, or indeed even at the same location. For example, in Sect. 8.1 it was mentioned that seismicity has been linked to the Lacq gas field in France, but Grasso et al. (2021) have recently demonstrated that the seismicity may have been due to injection of wastewater rather than the extraction of gas.

Another interesting case concerns hydraulic fracturing for shale gas in the vast reserves of the Sichuan basin in China, where there has been a great deal of seismicity associated with these operations. Tan et al. (2020), for example, identified a close spatial and temporal correlation between the hydraulic fracturing wells and the observed seismicity. The seismicity attributed to hydraulic fracturing in the Sichuan basin has included events of M_L_ 5.7 (**M** 5.3) in December 2018 and M_L_ 5.3 in January 2019 (Lei et al. 2019), which are the largest events that have been linked to hydraulic fracturing globally (Schultz et al. 2020a, b). On 17 June 2019 there was another earthquake, some 15 km to the north, with magnitude **M** 5.8. Jia et al. (2020) recognised the correlation between the overall intensity of the injections and the elevated seismicity in the region, but they conclude that this large event was likely due to water injections related to salt mining in the region, a conclusion also supported by Wang et al. (2020) and Li et al. (2021).

**Fig. 88:**
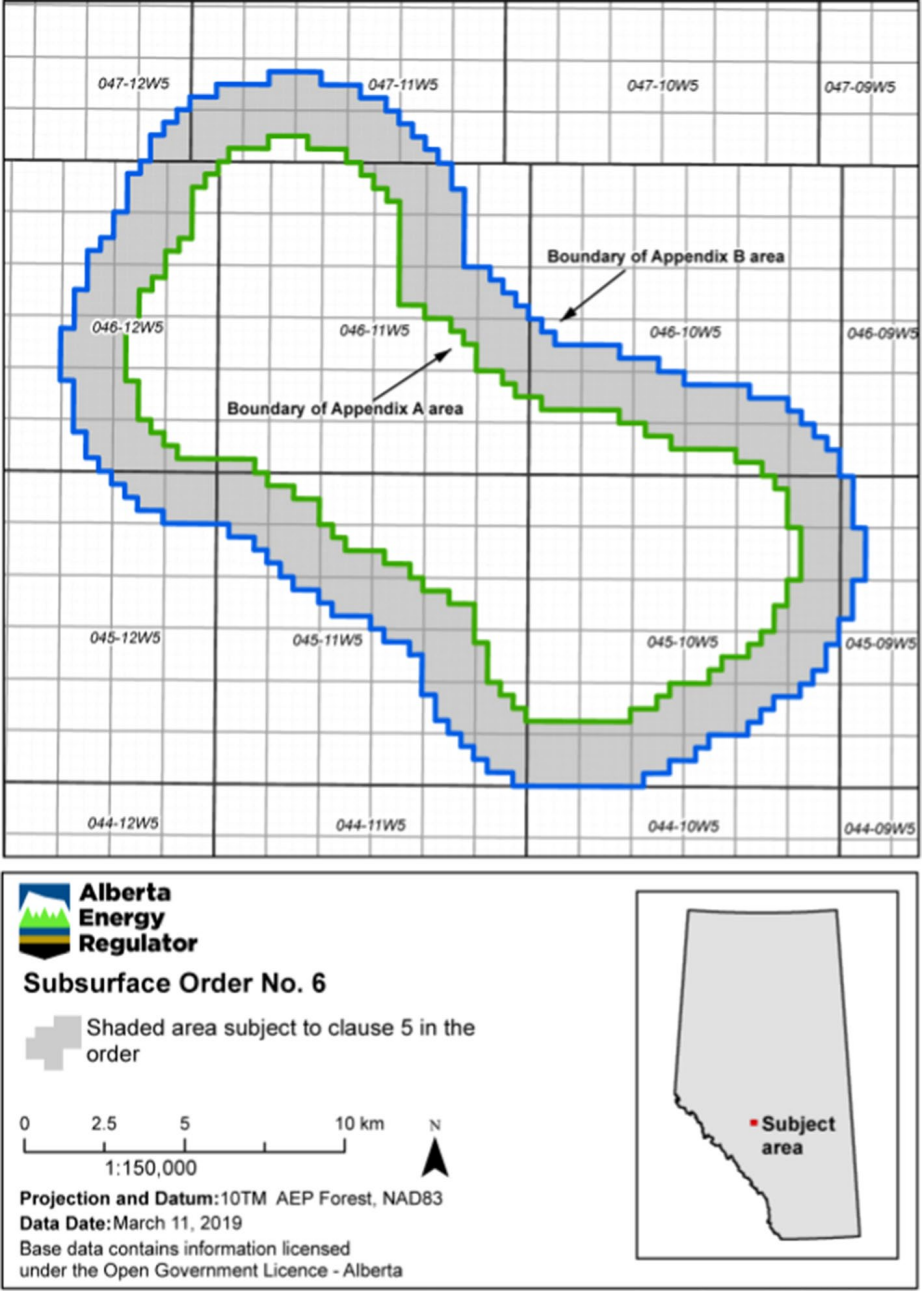
Exclusion zones for hydraulic fracturing around the Brazeau dam: no wells are permitted within the green boundary and no wells in the deep Duvernay formation are permitted within the blue boundary (AER 2019)

A final case is one with very immediate practical consequences. The Alberta Energy Regulator (AER) imposed restrictions in Subsurface Order no. 6 (SSO6) on hydraulic fracturing around the Brazeau hydroelectric dam in Canada, which forbid any wells within 3 km of the dam and its appurtenant structures, and additionally prohibited wells in the deep Duvernay shale formation within 5 km (Fig. 88). The specifications of SSO6 recognise the extensive induced seismicity that has been observed due to hydraulic fracturing in the Duvernay formation (e.g., Bao and Eaton 2016) and simultaneously the lower tendency for induced earthquakes in the shallower formations above the Duvernay. Applications for hydraulic fracturing wells targeting relatively shallow Cretaceous formations in the grey shaded area of Fig. 88 were opposed by the owner of the Brazeau dam, leading to regulatory hearings convened by the AER to determine whether the proposed wells would pose a seismic risk to the Brazeau dam facility. Ghofrani and Atkinson (2020) published a study that associated earthquakes in the WCSB with hydraulic fracturing wells in these Cretaceous formations, which then served as the starting point for the hazard and risk assessments to support the dam owner’s position. The method of Ghofrani and Atkinson (2020) was to calculate weights specifying the temporal and spatial correlation of earthquakes in the regional catalogue to hydraulic fracturing (HF) operations, considering wells in the different formations separately. The weights are assigned as 1.0 for a separation distance of 3 km or less and for a time interval between HF operations and the earthquakes of 5 days or less; with increasing distance and time, the two weight functions decay, the final weight, W, being simply the arithmetic mean of the two. A value of 0.35 for W is described by Ghofrani and Atkinson (2020) “*as passing a reasonable threshold for association*”; this value could be obtained by an earthquake occurring at 20 km from a HF well within 10 days of stimulation or by an earthquake occurring at 4.5 km from a well within 90 days of stimulation. The application of the method results in a small number of M ≥ 3.0 events that are assigned to HF wells in the Cretaceous formations, although these events were not listed in the paper. Verdon and Bommer (2021b) applied the Ghofrani and Atkinson (2020) algorithm using the same earthquake catalogue and database of wells in the region, and then individually examined the cases found to score above the threshold value of W. All of the earthquakes were found to be much more clearly associated with HF wells in the deeper Duvernay or Montney formations or else with wastewater injections in deeper formations. In their reply, Ghofrani and Atkinson (2021) supplied a list of the identified events, in which they include a single earthquake of magnitude greater than 3 associated with the Mannville and Cardium formations that were the subject of the AER hearings, namely the **M** 3.8 Ferrier earthquake of 10 March 2019, which, as Ghofrani and Atkinson (2021) acknowledge, is most likely of natural origin, given its reliably determined focal depth of 14 km. The implications of the erroneous associations for hazard and risk estimation are discussed further in Sect. 9.

## Seismic hazard and risk analysis for induced earthquakes

In Part I, I presented the view that approaches to the quantitative assessment of seismic hazard and risk have evolved greatly and there are well established practices in these fields that can also be applied to induced seismicity. However, several adjustments are required to adapt hazard and risk assessment to induced earthquakes.

### Seismic source models

As explained in Sects. 3.1 and 5.4, an SSC model defines the locations and average recurrence intervals of earthquakes of different magnitude. In PSHA studies for natural seismicity, the earthquake rates are inferred from past observations of earthquakes as reflected in the instrumental and historical earthquake catalogues. The same approach can be applied to include induced seismicity in hazard assessments: for example, in the United States, one-year hazard forecasts have been formulated based on observed induced seismicity during the previous year (e.g., Petersen et al. 2017). Such an approach requires the assumption that the seismicity will remain stationary—and implicitly, therefore, that the industrial operations will also not change—and only provides a short-term assessment. Whereas natural seismicity is characterised by observing the average numbers of earthquakes per year (resulting from continuous tectonic processes), the equivalent observational metric for induced seismicity should be related to the operations. The capacity to estimate the hazard for future operational scenarios is enhanced by relating the observations of induced earthquakes to a characteristic of the fluid injections, such as estimating the seismicity rates per well, for example, which can then be converted to rates per year on the basis of the foreseen number of wells per year. The seismogenic index, $\Sigma$, proposed by Shapiro et al. (2010), relates the seismic activity rate to the total volume of injected fluid, Q_c_, such that the Gutenberg-Richter recurrence relationship presented in Eq. (3) becomes:

7 $$\mathrm{log}\left(N\right)=\mathrm{log}\left[{Q}_{c}\left(t\right)\right]+\Sigma - bM$$

**Fig. 89:**
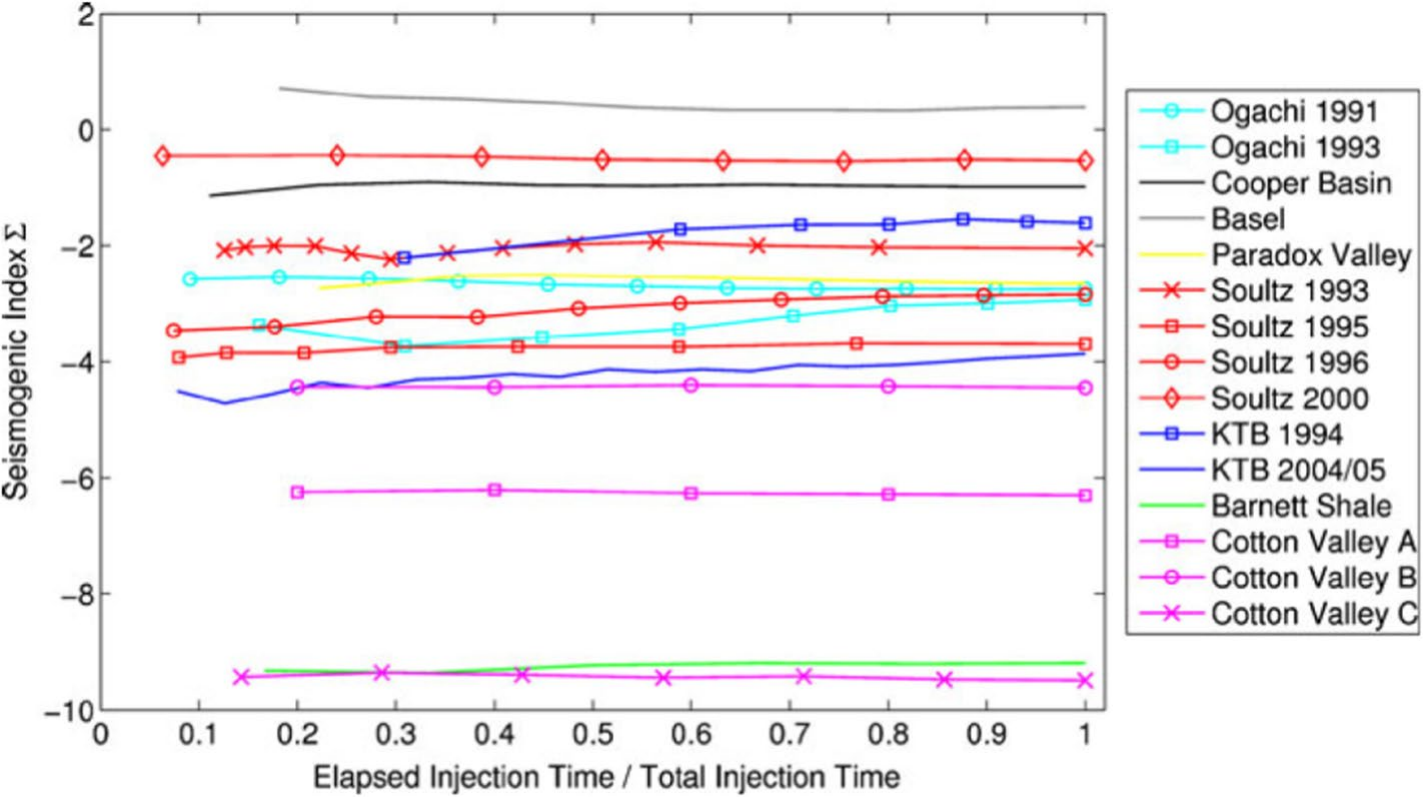
Values of the seismogenic index determined from fluid injections for experimental research, hydraulic fracturing, wastewater injection, and an enhanced geothermal project (Dinske and Shapiro 2013)

The first two terms replace the activity rate (the *a*-value) in the original equation, making the level of seismicity a function of the intensity of the injections and the seismic sensitivity of the local crust to these injections. The value of the seismogenic index is found to vary enormously from one formation to another (Fig. 89), reflecting the fact that fluid injections of the same volume can lead to very different seismic responses in different formations, including an effectively null response (such as the lowest values of $\Sigma$ depicted in Fig. 89).

The seismogenic index is a powerful tool for modelling induced seismicity, but it requires injections to have already taken place in the formation for which future hazard and risk estimates are required. The estimation of hazard for future operations that do not have precedent in the region and formation under consideration is extremely challenging. Although understanding of the geological and operational factors that influence induced seismicity is continually improving (e.g., Hincks et al. 2018; Keranen and Weingarten 2018; Ries et al. 2020), we are still a long way from being able to predict* a priori* the seismic response to fluid injections. Hydrological modelling of fluid pressure migrations can estimate pore pressure increase on known faults, albeit that this will require assumptions regarding rock permeabilities. This information can be combined with evaluation of the slip tendency of faults—based on their orientation and the tectonic stress field (e.g., Morris et al. 1996)—to estimate the likelihood of the injections leading to activation of mapped faults. However, since only the larger faults are likely to be identified and since the uncertainties associated with such models will usually be considerable, such analyses cannot be relied on as a basis for estimating induced seismicity characteristics from future operations in the absence of any empirical data.

If no prior injections have taken place and the seismogenic index has not been measured, a PSHA based on this parameter would need to assume a range of values, informed by values obtained for formations that might be considered potential analogues. Silva et al. (2021) performed a probabilistic risk analysis for possible future hydraulic fracturing in Manaus, Brazil, and captured the uncertainty in rates of induced seismicity through logic-tree nodes for $\Sigma$ (taking values between − 0.5 and − 2.5) and the Gutenberg-Richter *b*-value (taking values between 0.7 and 1.6). These logic-tree branches do reflect the epistemic uncertainty in these parameters, but for induced earthquakes of magnitude 5 and larger the ratio of the highest to lowest recurrence rates is greater than 3 million. Moreover, their logic tree also includes a node to capture the possibility that the hydraulic fracturing injections do not cause any induced seismicity, assigned a weight of 0.997. As noted earlier, Schultz et al. (2020a) report that globally only about 1% of hydraulic fracturing wells have caused earthquakes, so unless there is a basis to adjust this probability, perhaps based on factors such as lithology or depth of the formation, the first node of the hazard logic-tree would always assign a probability of ~ 0.99 to there being no induced seismicity. From the perspective of risk management, however, hazard estimates covering such a wide range of possibilities may not be particularly informative. In such circumstances, I would argue that a scenario-based approach is preferable, considering earthquakes with a range of magnitudes (see Sect. 9.2 for a discussion of maximum magnitude) and estimating the impact that each of these would have on the exposed building stock in the region, were they to occur. Such analyses could provide insights into the risks that induced earthquakes could pose and also identify the magnitude thresholds at which these risks would be unacceptable, thereby informing the design of mitigation measures (see Sect. 10).

In terms of the spatial distribution of potential induced seismicity, the model should reflect observed patterns in terms of separation between the injection well and induced events. If the hazard model considers a large number of wells distributed over a region, an area source zone encompassing all of the wells may be a suitable model, but for individual wells due consideration should be given to the tendency for fluid pressures to dissipate with distance. Injection-induced earthquakes have occurred at distances of several kilometres from the wells, and have also occurred at greater depth than the wells (and occasionally at shallower depths as well), but for many operations, induced earthquakes tend to occur in close proximity to the injection wells: Schultz et al. (2020a) state that for cases with well-constrained locations, the maximum distance of induced earthquakes from hydraulic fracturing wells has been on the order of 1.5 km.

As with natural seismicity, future earthquake sources can be represented by source zones of uniform seismicity or directly by earthquake catalogues. For induced seismicity in the Groningen gas field—discussed in detail in Sect. 12.4—the induced seismicity is found to be closely correlated with the reservoir compaction (Bourne et al. 2014). The seismicity model developed for hazard and risk calculations in Groningen uses Monte Carlo simulations, generating earthquakes in proportion to the compaction (Bourne et al. 2015).

### Maximum magnitudes

**Fig. 90:**
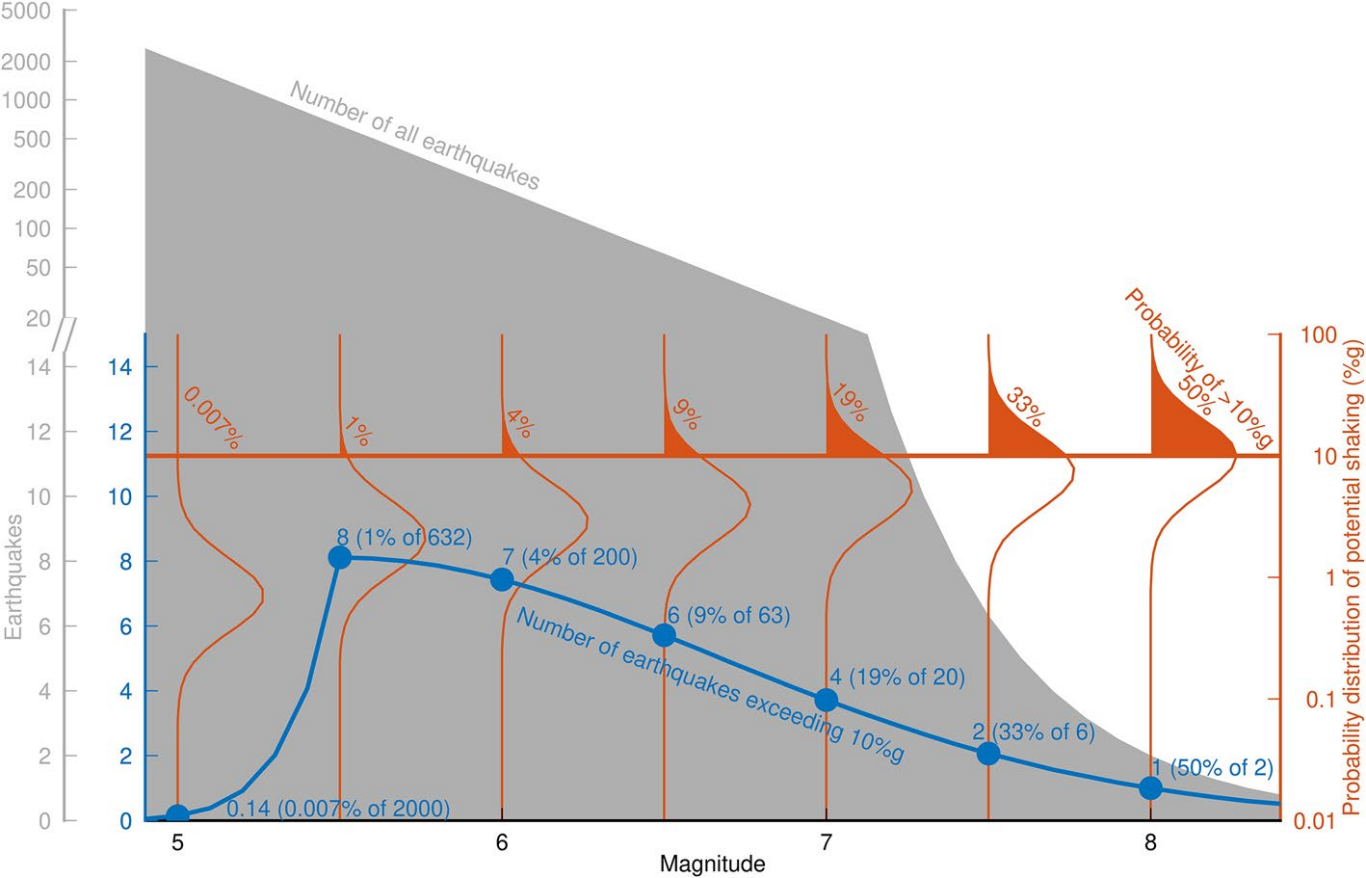
Schematic illustration of contributions to the hazard of a PGA value of 0.1 g as a function of the total number of earthquakes of different magnitude (grey) and the probability of exceedance related to the number of standard deviations required to reach that level of acceleration (orange); the hazard contributions by magnitude (blue) are the product of the two (Minson et al. 2021)

The maximum magnitude, Mmax, is the largest earthquake considered in hazard (and risk) calculations. In PSHA for natural earthquakes, it is generally the response to the question: what is the largest earthquake that could occur in this source under the current tectonic conditions? For fault sources, Mmax can be estimated from assumptions about how much of the fault could rupture in a single earthquake and empirical scaling relationships between magnitude and rupture dimensions. For cases where seismicity cannot be associated with known geological faults, estimation of Mmax is more challenging and a variety of approaches have been proposed that include extreme value statistics applied to the earthquake catalogue (Kijko 2004) and regional analogues (e.g., Wheeler 2016). Interestingly, although considerable effort has been expended on constraining models for Mmax, in PSHA for natural seismicity, usually defined by a range of possible values, it is a parameter that typically exerts a modest impact on hazard estimates (Fig. 90); hazard estimates are most often dominated by earthquakes of moderate magnitude (Minson et al. 2021). Due to the very low recurrence rates of the largest earthquakes (close to Mmax), combined with the non-linear scaling of ground motions with magnitude (Fig. 21) that requires more standard deviations to reach high amplitudes of motion, the scenarios close to Mmax tend not to contribute significantly to the hazard, except for very low annual exceedance frequencies and long oscillator periods. Consequently, Mmax values are often assigned rather conservatively in PSHA, which provides assurance against an earthquake occurrence contradicting the model, and there is no strong motivation to challenge large Mmax estimates since they have a modest impact on the resulting hazard estimates.

**Fig. 91:**
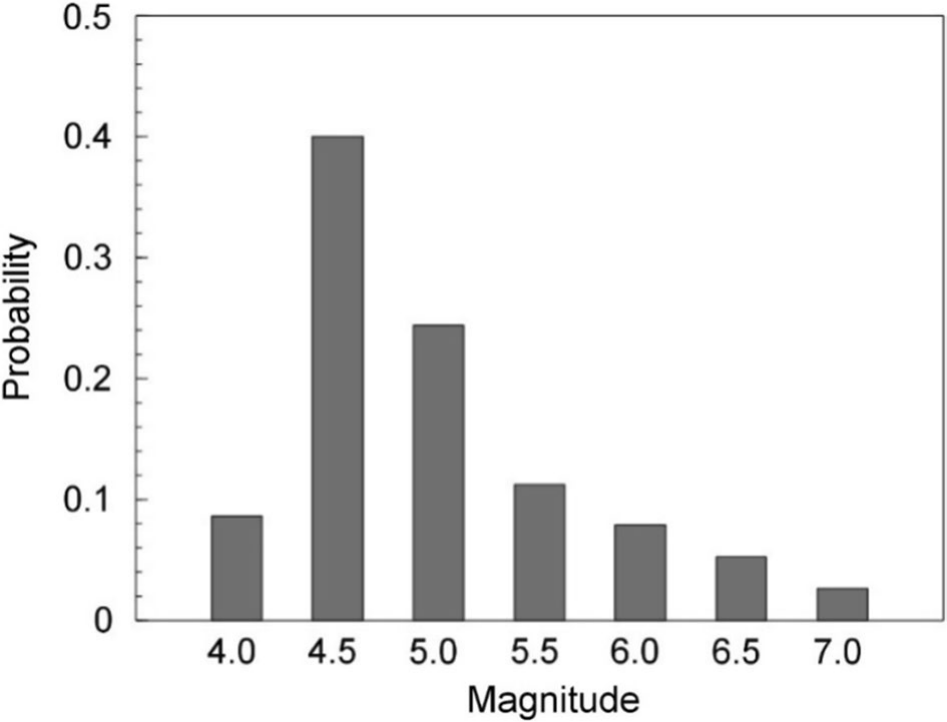
Mmax distribution for induced seismicity in the Groningen gas field (Bommer and van Elk 2017)

For induced seismicity, however, the choice of Mmax can be critical. At a workshop convened by the USGS to discuss the incorporation of induced seismicity into US national seismic hazard mapping, there was a majority view that the same Mmax values should be adopted as for natural seismicity (Petersen et al. 2015). For the case of wastewater injection-induced seismicity in Oklahoma and neighbouring states, where induced events have reached **M** 5.7 (e.g., Keranen et al. 2013), this may be a reasonable assumption, but for many other applications it could be grossly conservative. In the Groningen gas field, for example, the largest earthquake that has occurred was of magnitude M_L_ 3.6 (**M** 3.5), whereas regional seismic hazard assessments for natural earthquakes have assigned values of Mmax ≥ 6.5 (Woessner et al. 2015). The distribution of Mmax estimates defined by a specialist panel engaged specifically to address this issue, includes a long tail to cover the range of possibilities in terms of triggered tectonic earthquakes—and also influenced by the possibly spurious analogue of the magnitude 7 Gazli, Uzbekistan, earthquakes of 1976 and 1984 that have been tentatively linked to gas production (Simpson and Leith 1985)—but the lower end of the distribution was only fractionally above the largest observed event, and the highest weight assigned to a magnitude just one unit greater than the largest observed event (Fig. 91). If the approach of adopting the same Mmax distribution defined for tectonic seismicity had been followed, all the risk calculations would have included the impact of earthquakes of magnitudes from 4.5 up to 6.5, even though there is a very clear possibility that earthquakes of this size will never—and indeed, could not—occur in relation to the gas extraction.^2^ In my view, consideration should always be given to a distribution of Mmax values with the lower bound close to the size of the largest earthquakes that have actually been observed, rather than to suggest that the lower bound estimate of Mmax is two or three magnitude units greater than the largest observed event.

In terms of the upper bound on Mmax, several studies have proposed approaches for its estimation (e.g., Shapiro et al. 2011; Hallo et al. 2014). The approach of McGarr (2014), which has been widely adopted, relates the largest earthquake that can be induced by injections to the total volume of injected fluid. This hypothesis has been contested by van der Elst et al. (2016), who propose that the largest earthquake is essentially controlled by the tectonics of the region rather than characteristics of the operation—which is consistent with the concept of triggered seismicity. However, van der Elst et al. (2016) postulate that the maximum earthquake is also statistically controlled and increases with the number of earthquakes—which in turn increases with the volume of injected fluid.

There are at least two reasons why smaller Mmax values could be justified for induced seismic hazard and risk analysis than those used in PSHA for natural seismicity. Firstly, the operations—particularly in the case of hydraulic fracturing for shale gas recovery or enhanced geothermal systems—may be short lived, so the question should change from what is the largest earthquake that could occur during the present tectonic regime, to what is the largest event that could occur during these injections and the ensuing period of pressure equalisation? The response to such a question might be better provided by the concept of the maximum expected earthquake rather than the maximum possible earthquake (Holschneider et al. 2011). Secondly, most injections occur at relatively shallow depths compared to the mid-crustal depths at which large tectonic earthquakes tend to initiate, with the fault rupture propagating mainly upwards (e.g., Mai and Thingbaijam 2014). This is not to say that downward propagating fault ruptures do not exist: for example, several of the larger earthquakes that occur in the ancient crust of western Australia have very shallow focal depths (Leonard 2008). The 1968 M 6.5 Meckering earthquake is believed to have been associated with a downward propagating fault rupture (Vogfjörd and Langston 1987) and the **M** 6.0 2016 Petermann Ranges earthquake was associated with a rupture 20 km in length confined to the top 3 km of the crust (Wang et al. 2019). In California, Lomax (2020) calculated a focal depth of just 4 km for the **M** 7.1 Ridgecrest earthquake, “*implying nucleation in a zone not conducive to spontaneous, large earthquake rupture nucleation and growth*.” However, Lomax (2020) argued that this shallow hypocentre resulted from stress transfer due to a deeper (12 km) foreshock of **M** 6.4, without which rupture initiation of a large event at such shallow depth would not have occurred. Such cases remain the exception rather than the rule: in the database of more than 50 finite rupture models for both strike-slip and dip-slip earthquakes of Mai et al. (2005), in only six of the cases is the hypocentre located in the upper third of the rupture width and none in the top 15% of the rupture width. Therefore, in most settings it would seem that triggering large earthquakes by initiating faults ruptures at shallow depth would be rather unlikely.

One other compelling reason that smaller Mmax values may be appropriate for some operations that could potentially induce earthquakes is if there is a traffic light protocol (TLP) in place to control the seismicity levels. Such protocols are discussed in Sect. 10, but for now suffice to note that their primary objective is to limit the size of the largest induced earthquake—and if the implementation of a TLP does not result in a leftward shift of the Mmax distribution, then it is not really fulfilling its purpose.

### Ground Motion Models

Hazard and risk assessments often require the prediction of ground-motion amplitudes for earthquakes of very shallow focal depth and of smaller magnitude than might normally be considered when dealing with natural seismicity. For many years, GMMs were generally developed for application to earthquakes of magnitude 4.5 to 5.0 or greater, reflecting the widely used values of M_min_ (see Sect. 3.2). Using the Euro-Mediterranean ground-motion database to derive GMMs for magnitudes 5.0–7.6 and then for magnitude 3.0–7.6, Bommer et al. (2007) demonstrated that extrapolation of the equations derived from regression on larger magnitude overestimate the ground motions not only for smaller magnitudes but also at the lower limit of the upper magnitude range (M ~ 5). Chiou et al. (2010) made a similar finding by extending the Chiou and Youngs (2008) NGA-West2 using recordings from smaller magnitude events in California, and also finding differences between northern and southern Californian data that did not persist at larger magnitudes. The overestimation is now understood in terms of non-linear magnitude scaling of ground motions, already shown in Fig. 21, which also persists in the smaller magnitude range (Douglas and Jousset 2011; Baltay and Hanks 2014). The NGA-West2 GMMs accommodated these lessons through extension to much lower magnitudes (3.0–3.5), making them more suitable for such applications.

Douglas et al. (2013) developed GMMs for application to induced earthquakes associated with geothermal projects using a global database of recordings from such earthquakes as well as some induced earthquakes related to other processes. The highly heterogeneous database and poor characterisation of most of the recording sites resulted in models with very large sigma values. For the Groningen gas field, we identified the need for application-specific GMMs given that the recorded motions—probably due to specific features of the uppermost crustal structure—displayed systematic differences even with respect to induced earthquakes in other Dutch gas fields (Bommer et al. 2016). Ground-motion models for induced seismicity in other specific regions have been developed by several researchers, particularly for Oklahoma (Yenier et al. 2017; Novakovic et al. 2018; Zalachoris and Rathje 2019) or the Central and Eastern United States in general (Farajpour and Pezeshk 2021).

**Fig. 92:**
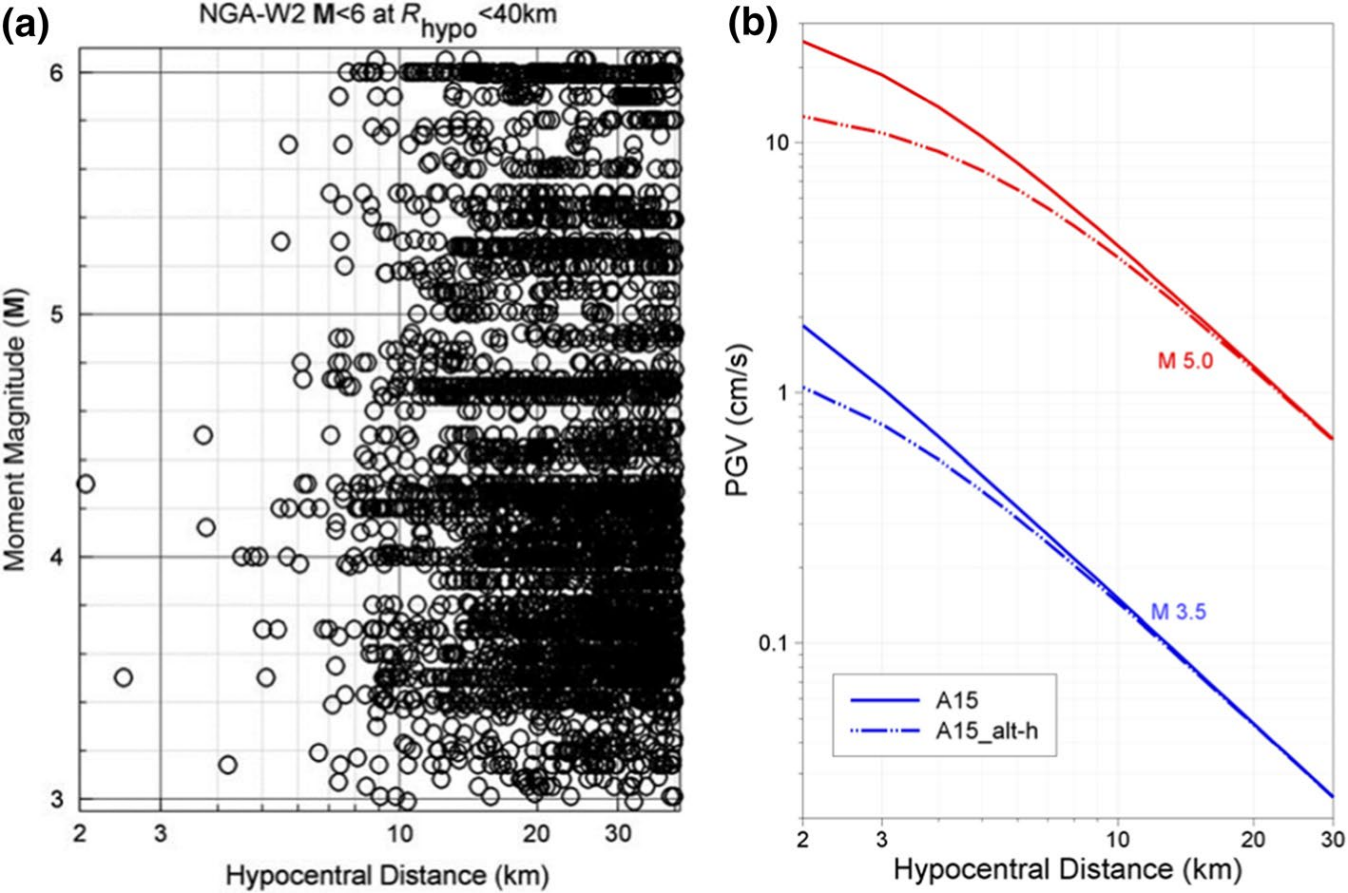
**a** Magnitude-distribution of the dataset used to derive the GMM of Atkinson (2015); **b** comparison of median predicted PGV values on rock for two magnitudes using the main equation (A15) and the alternative saturation term (A15_alt-h)

Atkinson (2015) developed an empirical GMM specifically for application to induced earthquakes but using recordings from tectonic earthquakes. The model was derived using recordings from the NGA-West2 database (Ancheta et al. 2014) obtained at hypocentral distance of less than 40 km from earthquakes of magnitude **M** 3 to **M** 6. These data offered the advantage of consistent and reliable metadata, including recording site characterisations. However, the data were sparse at very short distances, and this lack of constraint on the epicentral motions results in large uncertainty regarding the median predictions of epicentral motions, which was reflected in two alternative models for the degree of near-source saturation (Fig. 92). The difference between the two models at the epicentre of shallow-focus events is about a factor of 2. In a subsequent study, Atkinson et al. (2016b) performed analyses that indicated that the alt-h model was to be preferred and Atkinson and Assatourians (2017) explicitly recommended use of the model with the alternative saturation term.

A potential shortcoming of the Atkinson (2015) GMM is that it does not account for the relationship between stress drop and focal depth; the stress drop, or stress parameter, is a measure of the strength of the high-frequency radiation from an earthquake (see Sect. 5.2). Several studies have found that it is correlated with depth, such that deeper crustal earthquakes have higher stress parameters (e.g., Hardebeck and Aron 2009; Trugman and Shearer 2017). Abercrombie et al. (2021) have recently concluded that these findings arise from not modelling the depth-dependence of wave attenuation, but for models that do include depth-dependent attenuation, the use of depth-dependent stress drop is a proxy for capturing this effect. From this perspective, the A15 model uses data from mid-crustal tectonic earthquakes as the basis for prediction of motions from shallower induced earthquakes, without an adjustment for the reduced stress parameter. The application of the model to induced earthquakes in Central and Eastern United States has been justified on the basis of average stress drops in that region being higher than in California, from where the data were obtained (e.g., Allmann and Shearer 2009; Boyd et al. 2017; Huang et al. 2017). This rationale, however, does mean that the application of the Atkinson (2015) GMM to induced earthquakes in other regions, where median stress drops might be comparable to those in California, would be conservative.

**Fig. 93:**
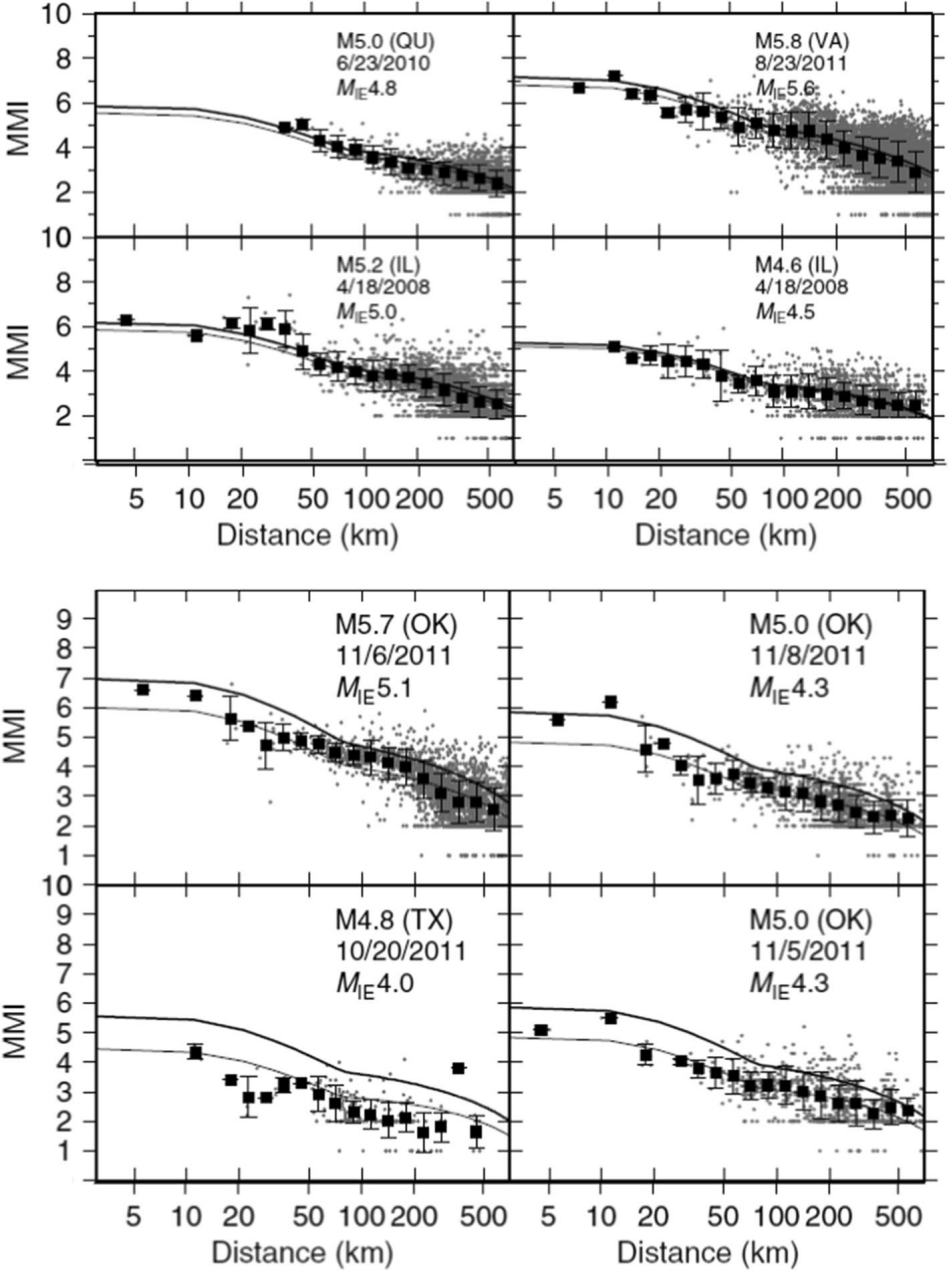
Intensity data from four tectonic (upper) and four induced (lower) earthquakes in the Central and Eastern US. The thin lines are the best fit to the data, the thicker grey line the predicted intensities from Atkinson and Wald (2007); adapted from Hough (2014)

A critical question that this raises is whether induced earthquakes, by virtue of their shallower focal depths, generate stronger motions in the epicentral region than tectonic earthquakes of the same magnitude, or whether the apparently lower stress drops of shallow events counterbalance the reduced travel paths. Hough (2015) analysed intensity data from natural and induced earthquakes in the Central and Eastern United States, from which she made two observations: (1) the motions from shallow, induced events are generally lower, and (2) that the motions are comparable in the epicentral region (Fig. 93). This was interpreted as being the result of lower stress drops for the shallow-focus, induced earthquakes with this effect being offset by the shorter travel paths to the surface close to the epicentre. Atkinson et al. (2018) also analyse Did-You-Feel-It (DYFI) intensity data from induced and natural earthquakes in Central and Eastern United States and arrive at very similar conclusions to those reached by Hough (2014). Atkinson et al. (2018) find that “*natural and induced events have similar average intensities within 10 km of the epicenter…… a consequence of two focal-depth effects that have offsetting impacts on the strength of ground motion: (1) the epicenter is near the source for shallow events, and (2) the stress parameter scales with focal depth.*” Whether the effect is due to depth dependence of the stress parameter or has another physical explanation, the concept of ground motions being weaker for shallower events seems to be a common observation. Indeed, such an effect is captured in several of the NGA-West2 GMMs with terms that predict higher amplitudes of motion with increasing depth, through positive coefficients on either the depth-to-top-of-rupture, Z_TOR_ (Abrahamson et al. 2014; Chiou and Youngs 2014) or on the hypocentral depth (Campbell and Bozorgnia 2014).

In summary, for induced seismic hazard and risk assessment, GMMs are required that are calibrated for application to the appropriate range of magnitudes and also to the focal depths typical of induced events. Provided that a model captures the non-linear scaling over the full range of magnitudes and the depth dependence of the ground-motion amplitude, the same GMM should be applicable to both tectonic and induced earthquakes in a given region. The applicability of models derived from induced earthquakes in one region should not, however, automatically be assumed to apply to induced seismicity in another region.

### Minimum magnitude

The purpose and definition of the lower bound magnitude in PSHA, M_min_, was discussed in some detail in Sect. 3.2. It is interesting to note that some practitioners argue for the same Mmax values as used for natural earthquakes (which, in Sect. 9.2, I have suggested will often not be appropriate) and lower M_min_ values when dealing with induced seismicity. The minimum magnitude is a proxy for ground motions that are not expected to be damaging, but in light of the conclusions drawn in the previous section—namely that epicentral motions from induced and natural earthquakes in a given region should be comparable—there is no reason to use different minimum thresholds when assessing hazard and risk due to induced and natural earthquakes. Indeed, if the impact of induced seismicity is to be evaluated through comparison of the relative hazard contribution compared to that from tectonic earthquakes in a region, the use of different M_min_ values could lead to a distorted view, since this would not be a like-with-like comparison. The same does not hold for using different values of Mmax, if there are reasons—as there often will be—for a different distribution of upper bound magnitudes for induced earthquakes.

The values of M_min_ used in hazard and risk assessments for induced earthquakes may well be lower than those used in standard PSHA studies that are performed to determine seismic design loads considering tectonic earthquake activity. The reason for this is that the exposed building stock may be of low seismic resistance due to deterioration and lack of maintenance, and moreover induced seismicity can occur in regions with very low levels of natural seismicity, whence there may be no requirements for earthquake-resistant design considerations in applicable building codes. But given what appears to be the current consensus that in any given region shallow induced earthquakes and deeper tectonic earthquakes of the same magnitude are expected to generate similar levels of ground shaking at the epicentre, the values of M_min_ used in hazard and risk assessments should be controlled only by the fragility of the exposed infrastructure and buildings (and the damage levels of interest in the risk assessment), regardless of whether we are dealing with induced or natural seismicity. The magnitude thresholds at which earthquake damage may be expected are discussed further in Sect. 11.

### Risk Analyses for induced seismicity

In the Introduction of this article, I argued that hazard should not be separated from risk, and this holds as much, if not more, for induced seismicity as it does for tectonic earthquakes. Assessment of the seismic hazard due to potential induced earthquakes is insufficient to make rational decisions that balance risks and benefits; as discussed further in Sect. 10, risk management of induced seismicity should be informed by quantitative risk assessments.

From this perspective, it is encouraging to see that several risk assessments have been published for cases of induced seismicity. Mignan et al. (2015) performed an intensity-based risk assessment for the Basel enhanced geothermal system in Switzerland, a case history explored in greater depth in Sect. 12.1. Langenbruch et al. (2020) perform a risk analysis in terms of economic loss due to low-probability, large impact earthquakes, based on the Pohang geothermal project in South Korea.

Gupta and Baker (2019) evaluate induced seismic risk in Oklahoma and Chase et al. (2019) for Central and Eastern US in general, both related to wastewater injection. An elaborate seismic risk model has been developed for induced seismicity in the Groningen gas field, which is described in Sect. 12.4.

**Fig. 94:**
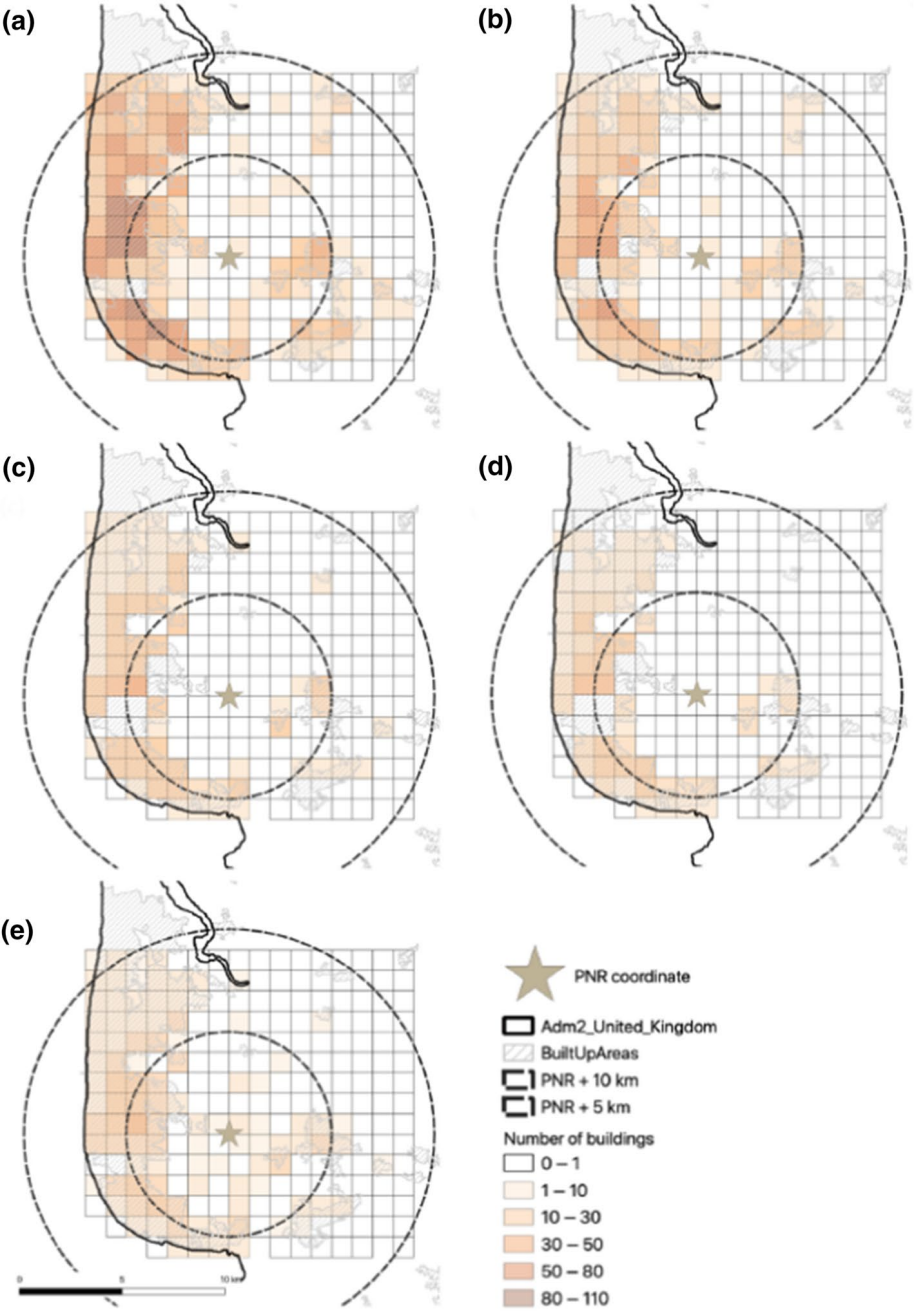
Risk analysis results for an induced earthquake scenario of M_L_ 4.5 associated with hydraulic fracturing in northwest England expressed in terms of percentage of buildings with each 1 km^2^ grid experiencing damage states **a** DS1, **b** DS2, **c** DS3 and **d** DS4, or **e** chimney collapse (Edwards et al. 2021)

Risk studies have been performed for induced seismicity associated with hydraulic fracturing, one example being the study for Manaus by Silva et al. (2021) mentioned earlier. Edwards et al. (2021) estimated the risk associated with hydraulic fracturing for shale gas in the UK (see Sect. 12.2) using a scenario-based approach. Ground-motion recordings from induced earthquakes generated by the operations were used to select GMMs and V_S30_ maps were generated based on surface lithology and multi-channel analysis of surface waves (MASW) measurements conducted in the region. A regional exposure model was constructed using open access databases and on-site inspections, and then risk calculations performed for scenarios of different magnitude. The results obtained for the largest scenario (M_L_ 4.5) are shown in Fig. 94.

In the same way that hazard analyses need to be adapted to the particular characteristics of induced earthquakes, the fragility functions should also be derived from analyses using hazard-consistent motions (see Silva et al. 2019 and Chase et al. 2021 for interesting discussions of selecting ground-motion inputs for the derivation of fragility function). Fragility functions expressed, for example, in terms of PGA and calibrated for moderate-to-large magnitude tectonic earthquakes could be expected to overestimate the impact of induced earthquakes of smaller magnitude. The characteristics of ground motions that influence earthquake damage are briefly discussed in Sect. 11.1.

### Induced seismicity and epistemic uncertainty

The key theme of Part I of this article was the identification and inclusion of uncertainties in seismic hazard assessment, as a contribution towards achieving acceptance of seismic hazard and risk estimates as the starting point for rational decision making with regards to risk management. I also acknowledged how an earnest effort to incorporate uncertainties and to communicate transparently their influence on the calculated risk can have the undesirable consequence of conveying the impression that very little is known or understood and that we are therefore dealing with unquantifiable dangers, which naturally provoke greater concern. Both of these aspects—demonstrating the inclusion of uncertainties in risk estimates and the possibility of this generating more concern rather than assurance—are very relevant when dealing with induced seismicity. Induced seismicity will generally be viewed as an imposed or involuntary peril rather than a natural hazard, leading to lower tolerance. There are numerous examples of how strongly risk perception can be influenced by whether a risk is voluntary or imposed, such as protests against mobile phone transmitter masts being installed close to schools by parents who are happy to allow their children to spend hours every day using mobile phone handsets, even though there is no evidence for the former posing a greater risk (e.g., Wood 2006). Another example would be the news coverage given to major rail accidents in the UK while the death toll can be comparable to the number of fatalities on British roads every week. In dealing with induced seismicity, it is necessary to keep in mind that discussions surrounding induced seismicity are rarely likely to begin from an objective assessment—especially when the anthropogenic process generating the induced seismicity is already steeped in controversy.

In the light of these considerations, the adoption of the SSHAC process (Sect. 6) for the assessment of seismic hazard and risk could be very beneficial. The SSHAC guidelines provide a clear and transparent process through which to conduct hazard and risk assessments, with observation of the process by independent peer reviewers, regulators and other stakeholders. The process also provides a framework for the presentation and discussion of all scientific viewpoints. To date, to my knowledge, there has yet to be a full induced seismic hazard or risk assessment conducted following the SSHAC process. The assessment of Mmax for Groningen followed many of the core SSHAC principles (Bommer and van Elk 2017) but plans to conduct the comprehensive risk assessment for induced earthquakes in Groningen as a SSHAC study were thwarted, as discussed in Sect. 12.4.

Whether or not the SSHAC guidelines are formally adopted, hazard and risk assessments for induced seismicity should still aim for the SSHAC objective of capturing the centre, body, and range of technically defensible interpretations (CBR of TDI). The purpose is to construct the best model that is supported by the current data and state-of-knowledge, and to estimate the ranges of uncertainty associated with this model (i.e., alternative models supported by the data and models that acknowledge the limitations of the data). This should not include any decisions that are deliberately conservative since that is incompatible with the probabilistic approach to risk assessment to inform rational risk management. I would argue that the precautionary principle has no place in the management of induced seismicity. The precautionary principle essentially counsels that in the light of great uncertainty about the impacts of certain actions and the possibility of these impacts being far-reaching and difficult to reverse, precaution should govern, and such actions should consequently be limited or avoided, at least until more knowledge can be acquired. When dealing with new technologies that could have far-reaching consequences for the environment and for public health, such an approach may often be justified (e.g., Read and O’Riordan 2017). However, in the case of induced seismicity, the application of the precautionary principle would reflect an underestimation of our understanding of the phenomena and of the ability of earthquake engineering both to model and to modify seismic risk; it would be to abandon rational risk management.

**Fig. 95:**
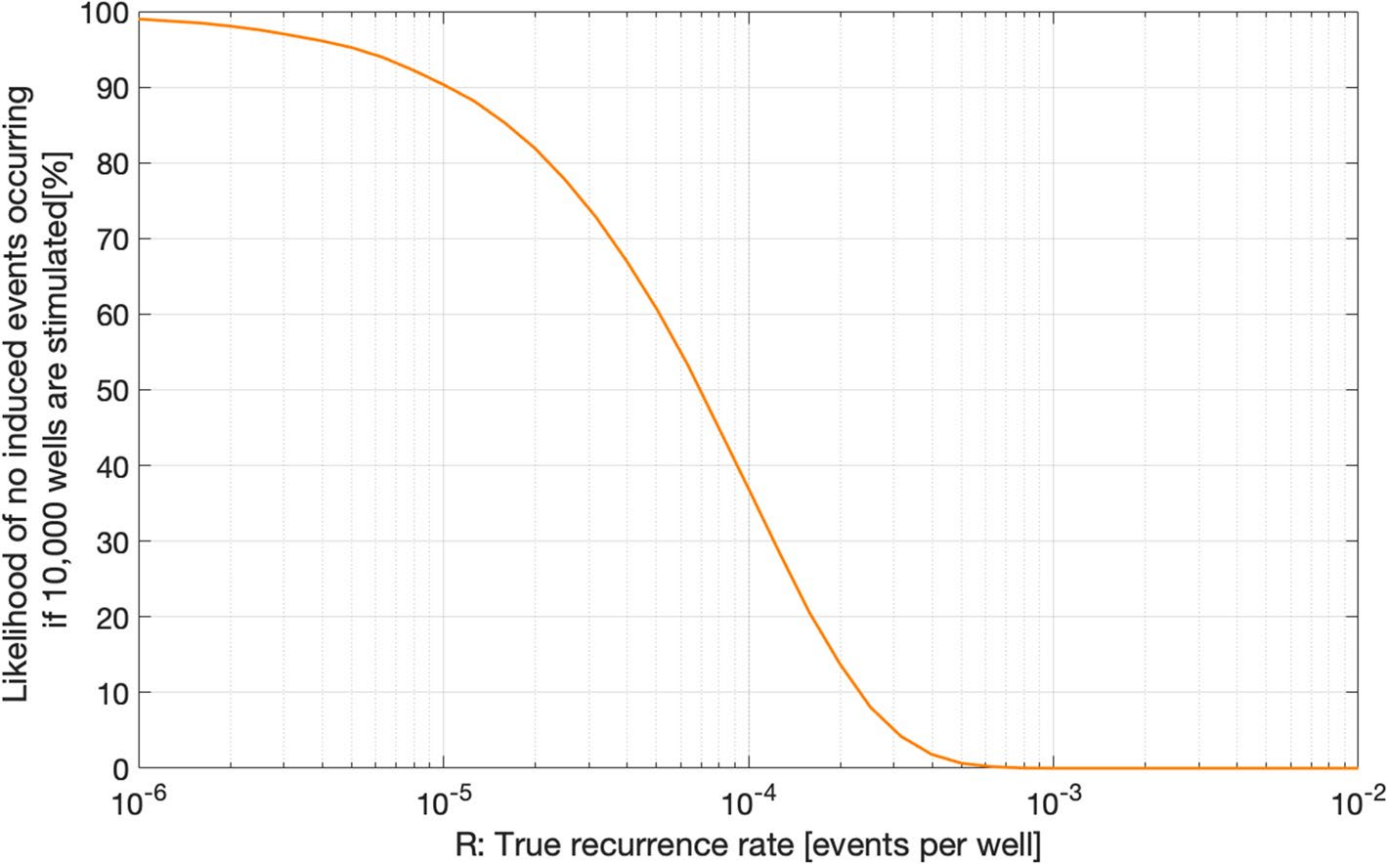
Likelihood of observing no induced earthquakes after 10,000 hydraulic fracturing wells as a function of the unknown true rate of earthquakes (Courtesy of Dr James P Verdon)

These points can be illustrated with a case in point, referring to the applications for hydraulic fracturing licenses for wells in Cretaceous sandstone formations close to Brazeau dam, introduced in Sect. 8.3. The logical starting point to assess the risk that these operations could pose is to evaluate the induced seismicity that has been generated by the ~ 10,000 hydraulic fracturing wells that have already been drilled and injected in these formations in the WCSB. Using loose spatial and temporal correlations that ignore more plausible causes, Ghofrani and Atkinson (2020) associated a small number of M ≥ 3.0 earthquakes with some of these Cretaceous wells. Our analysis, which looked at all potential causes for each of these events (Verdon and Bommer 2021b), demonstrated that it was extremely unlikely that any earthquake of M ≥ 3 had been caused by hydraulic fracturing in Cretaceous formations; although it can be stated with less confidence because of catalogue completeness issues, it is likely that there have been no induced events of M ≥ 2 either (in other words, the formations would appear to have an extremely low seismogenic index). In their rebuttal of our comment, Ghofrani and Atkinson (2021) state their disagreement regarding how associations of seismicity and anthropogenic associations should be made—after we note that they ignored all of the approaches that have been proposed in the literature (see Sect. 8.2)—and then go on to state: “*A second point on which we disagree is an issue that was tangential to our paper: whether a regulator should consider the potential for induced seismicity from HF wells in shallow (Cretaceous) formations to be very low (as implied by GA20) or zero (as implied by VB21)…. In the world of probabilistic seismic hazard analysis (PSHA), the difference between very low probability (i.e., 10*^−4^ *p.a.) and zero is profound. Equally critical in PSHA is the amount of uncertainty in the assessment. VB21 imply that the likelihood of inducing significant seismic events from HF wells in Cretaceous formations is zero, and that there is essentially no uncertainty in this conclusion.*” Verdon and Bommer (2021b) only focused on the science presented in the study of Ghofrani and Atkinson (2020), rather than entering into the hazard and risk implications, but these statements by Ghofrani and Atkinson (2021) are misleading since they extrapolate from our finding that no induced earthquakes have occurred due to hydraulic fracturing in the Cretaceous formations to an assertion that we did not make. The observations associated with ~ 10,000 previous wells in the region (of which several hundred are very close to the proposed operations around Brazeau Dam) is a remarkable database and far richer than the equivalent earthquake catalogues available for most PSHA studies of tectonic seismicity. However, rather than simply inferring a zero probability, the implied ranges of recurrence rates can be explored by performing a Monte-Carlo type analysis. For a given ‘true’ recurrence rate, R, one can generate a population of 10,000 wells and randomly assign induced events at the specified rate R. For each choice of the true rate, R, this iteration is performed 1,000,000 times, and then the resulting population evaluated: out of the 1,000,000 iterations, how likely is it that 10,000 wells would be stimulated without generating any plausible cases of induced seismicity? The results are shown in Fig. 95. If, for example, the true recurrence rate was R = 10^–3^ (1-in-1000), then the likelihood of having a population of 10,000 stimulated wells in the Mannville and Cardium with zero cases of induced seismicity of magnitude ≥ 3 is only 0.005%. Allowing for the possibility that there has been, somewhere in the WCSB, a single case of induced seismicity from stimulation of the Cretaceous formations that was missed (despite the close monitoring, regulatory vigilance and public interest), the likelihood of generating one or fewer cases of induced seismicity from 10,000 wells with a 10^–3^ recurrence rate is still only 0.05%. In this way, a range of recurrence rates could be defined in a logic-tree formulation, with the central branches indicating very low—but non-zero—recurrence rates.

Extending the discussion beyond the science of identifying induced earthquakes and correctly associating these events with anthropogenic operations to a discussion of seismic risk management, Ghofrani and Atkinson (2021) allude to invoking the precautionary principle, stating that: “*The difference between low and zero probability leads to opposing conclusions as to whether it is prudent to conduct HF operations in shallow formations beneath major high-consequence facilities such as dams or nuclear power plants*.” Leaving to one side the fact that their low probability is over-estimated by the erroneous associations, their general position is not consistent with rational risk management. Given the abundant data available regarding hydraulic fracturing, wastewater disposal and induced seismicity in the WCSB, and the clear possibility of quantifying the hazard and estimating the associated uncertainty, why should there be a need to make recourse to the precautionary principle rather than estimating induced seismic hazard and risk and evaluating these on the same basis as for tectonic earthquakes? As noted in Sect. 9.2, the assessment of Mmax for the potential induced seismicity should take account of the observed seismicity, which, following the same logic as applied in Groningen, would in this case lead to a distribution with a peak at quite low magnitudes. Indeed, depending on the M_min_ determined (the smallest earthquakes known to have damaged dams are discussed in Sect. 11.2), it is possible that a good part of the Mmax distribution could be below this threshold, leading to null risk contributions.

## Mitigation of induced seismic risk

Earthquake engineering could be defined as the design and construction of buildings and infrastructure to resist the potentially damaging effects of earthquakes. The practice of earthquake engineering is very well established, and its efficacy has been proven repeatedly by the satisfactory performance of buildings, bridges, and power plants, among others, during strong earthquakes. Considering the four elements of seismic risk illustrated in Fig. 6 (hazard, exposure, fragility and consequences), once a decision is taken to construct a building or facility at a given location, the exposure is determined, and the intended use of the structure determines the consequences of unsatisfactory performance during possible future earthquake. The seismic hazard due to tectonic earthquakes in the region can be quantified in order to determine the shaking levels to be resisted, and then earthquake engineering principles applied to control the remaining factor, the fragility. Through appropriate provision of structural stiffness, strength and ductility, structures can be designed to meet the requisite performance targets—which may range from non-collapse to protect life safety through to complete structural integrity and safe operation for critical installations—under the specified design motions.

In the case of induced seismicity, which occurs as the result of industrial operations, there is the possibility to reduce the risk by modifying the hazard, an option that is not available to conventional earthquake engineering. Systems have been developed and applied to allow these modifications to be made in response to observed indicators of increasing levels of induced seismicity. However, the option to adaptively modify the hazard through adjustments to the operations does not mean that the application of earthquake engineering should not also be included as part of the risk mitigation strategy in some cases. Indeed, the options for modifying all the elements of the risk formula should be considered when managing the potential risk due to induced earthquakes.

### Traffic light protocols

Induced seismicity due to fluid injections occurs as the result of pressure changes in the vicinity of critically stressed geological faults. Reducing the rate or total volume of the injections should therefore lead to a reduction in the level of induced earthquake activity—and suspending the operations completely should lead, once pressures dissipate, to a cessation of induced seismicity. A clear illustration of this principle was the decision in 2016 by the State of Oklahoma to impose a 40% reduction in total injected volume of wastewater from oil production in the regions most susceptible to induced seismicity. Langenbruch and Zoback (2016) predicted that this would lead to significant decrease in seismicity—which has indeed been observed—although they noted that stabilisation would take some time due to the ongoing aftershock sequences following some of the larger induced earthquakes that have occurred in Oklahoma. Dempsey and Riffault (2019) estimated that a 60% reduction in the volume of injected wastewater would be required to bring seismicity levels back down to the natural background levels.

For individual operations, systems have been established to enable modifications to operations (which in practice always means injections) in response to observed increases of induced seismicity activity. The basis for such systems is a dedicated network of sensitive seismographs, sometimes installed in boreholes to improve signal-to-noise ratios, to monitor seismic activity in the immediate vicinity of the injection wells. The system requires the recordings to be telemetered and analysed to provide locations and magnitudes in close to real time. Different thresholds are then defined based on a selected metric, such as the earthquake magnitude, to indicate whether the seismicity is increasing to levels that could become intolerable. These thresholds are assigned colours, with green indicating that seismicity is null or very low and operations may proceed without change, yellow indicating an increase in seismicity that requires remedial action (reduction in the pressure and/or flow rate of the injections), and red indicating that the seismicity has exceeded a pre-determined threshold and the operations need to be suspended; some systems will also define an orange level between yellow and red for a more graded response. The combination of the seismograph network, real-time locations and magnitude estimates, the definition of thresholds, and the defined response actions—which will often also include communications to regulatory and other agencies—is known as a Traffic Light Scheme (TLS) or Traffic Light Protocol (TLP).

The fundamental purpose of a TLS is to avoid levels of ground shaking that would exceed tolerable limits, which would generally mean causing damage to buildings in the vicinity of the operation. Two assumptions are implicit in the design and operation of TLS as an effective risk mitigation tool for induced seismicity. Firstly, it is assumed that induced seismicity will increase gradually during injections such that there are precursor events of smaller magnitude that occur before any event that would exceed the maximum tolerable threshold. Secondly, it is assumed that actions taken to reduce the injections will have the desired effect of preventing further increases in the number or size of induced earthquakes. The validity of both these assumptions will be discussed a little later. However, it has been concluded that TLS are only really suitable for short-term high-pressure injections, such as those associated with enhanced geothermal systems (EGS) and hydraulic fracturing (HF) for unconventional hydrocarbon production (Baisch et al. 2019). The application of TLS to wastewater injection has also been proposed (Zoback 2012) but most implementations to date have been for EGS and HF wells. To my knowledge, there have been no applications of TLS, as described herein, to fluid extraction processes.

**Fig. 96:**
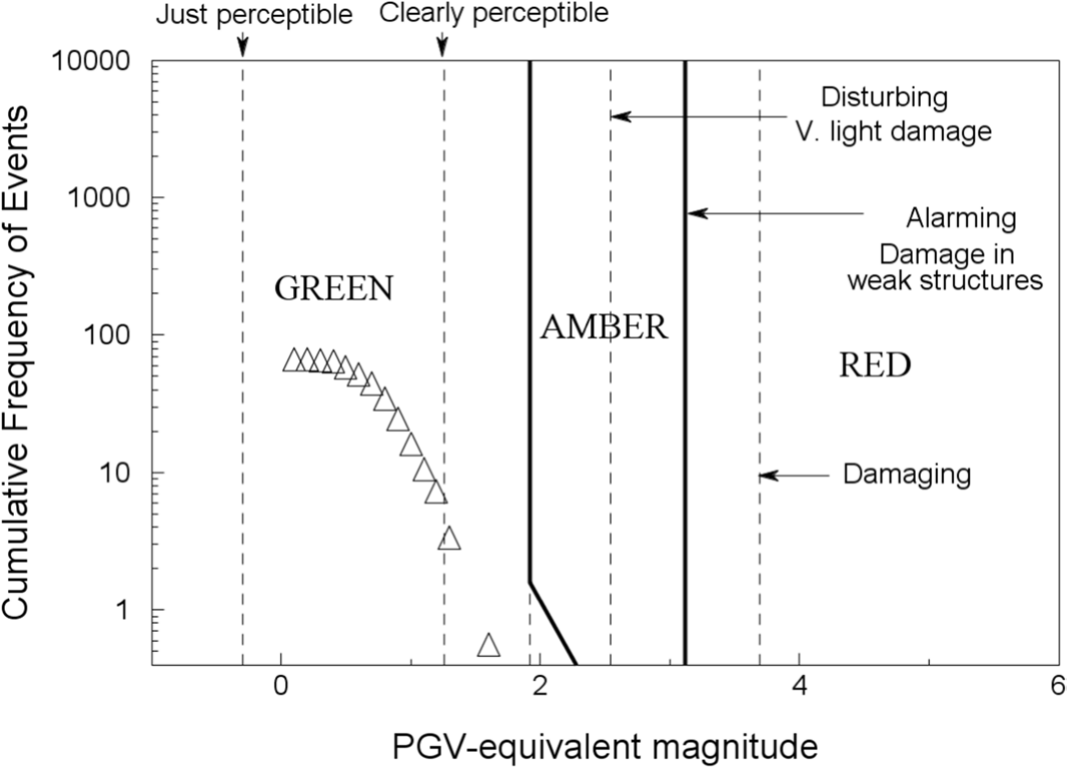
Traffic light thresholds in terms of PGV-equivalent magnitude defined for the Berlín hot fractured rock (HFR) geothermal project in El Salvador; the triangles correspond to the observed background seismicity (Bommer et al. 2006)

Once the instrumentation and near-real time source parameter determination system are in place, the two critical steps in designing a TLS are the selection of the earthquake metric and the definition of the thresholds of this metric that define the green, yellow, (orange) and red-light triggers. Regarding the metric, since it is the intensity of the shaking that determines the impact of an earthquake, it would seem logical to define the threshold in terms of a ground-motion parameter. The peak ground velocity (PGV) is the most widely used parameter, since it can serve as a useful indicator of both perceptibility of the motion to people and of the potential for damage to buildings. However, challenges arise with this parameter since the value of PGV will vary from one location to another, and therefore its use would require the installation of strong-motion instruments at several locations around the injection well, ideally including the locations of exposed buildings. Ader et al. (2019), in designing a TLS for a deep geothermal project in Helsinki, identified two potential pitfalls in using PGV as the TLS metric: firstly, false positives could be triggered by vibrations from other anthropogenic sources close to one of the instruments; secondly, false negatives could be result from the largest PGV occurring at a location where there is no instrument. Ader et al. (2019) addressed these issues by specifying the amber threshold on the basis of either a certain PGV level associated with a minimum magnitude or a larger magnitude in isolation. Magnitude has the advantage of yielding a single value for an earthquake—notwithstanding that there are challenges in reliably determining the magnitudes of small events (e.g., Butcher et al. 2017; Kendall et al. 2019)—and it can be determined very rapidly. Moreover, since the induced earthquakes can be expected to occur close to the well and at depths equal to or slightly greater than the injection depth, for induced seismicity the magnitude can be a reasonable proxy for the epicentral motions. In the TLS developed for the Berlín EGS in El Salvador (Bommer et al. 2006), thresholds were defined in terms of PGV, as described below, but these thresholds were converted to equivalent magnitudes by assuming that these would correspond to median predictions at the epicentre for an earthquake at the depth of the injection well, using a GMM calibrated to recordings of local small-magnitude earthquakes. To additionally account for the rates of seismicity, the thresholds were displayed on a magnitude-frequency recurrence plot, with the limit of the green light corresponding to the observed background seismicity levels prior to the start of the injections (Fig. 96).

Magnitude thresholds defined for yellow and red lights vary considerably from one jurisdiction to another. For example, red light thresholds are set at M_L_ 4.0 in Alberta, British Columbia and Illinois, and at 2.5 in California (Kendall et al. 2019). The red-light threshold should be fixed as the starting point, since this determines the level at which operations will be suspended because the situation is viewed as becoming dangerous. Some researchers have proposed that threshold should be set at the levels that cause nuisance or disturbance to people (e.g., Douglas and Aochi 2014; Cremen and Werner 2020; Schultz et al. 2021a) but such an approach could lead to very low thresholds if these levels of motion determine the trigger for a red light. Motions that might be considered a nuisance could correspond to intensity as low as III, whereas the threshold for even light damage to normal buildings is intensity VI, with very considerable differences in the implied levels of motion between the two: using the empirical relationships of Caprio et al. (2015), these would correspond to median PGV values of 2.77 cm/s and 9.81 cm/s, respectively. If the red light corresponds to such a low threshold, the yellow light is likely to be fixed at a level that leads to frequent interruptions of the operations; excessively low thresholds can be counterproductive, as discussed in Sect. 12.2. The red light, in my view, is better determined by considering the magnitude level that could correspond to the onset of damage; however, as explained below, the threshold for the red light should take into account possible ‘jumps’ in earthquake size. The earthquake magnitudes that might be appropriate thresholds for the onset of damage are discussed below in Sect. 10.3 and are also the entire focus of Sect. 11.

The thresholds selected for the TLS shown in Fig. 96 were informed by several considerations: published thresholds of frequency-dependent PGV levels for tolerable vibration levels due to quarry blasting, traffic and pile driving; fragility curves for local building types, expressed as a function of PGV; and empirical conversions between intensity and PGV. As can be seen in the figure, the red light corresponds to the thresholds of shaking at which damage could occur, a topic discussed further in Sect. 11.1. In this region of relatively high natural seismicity, perceptible levels of shaking were viewed as tolerable and to be handled through engagement with the local inhabitants.

**Fig. 97:**
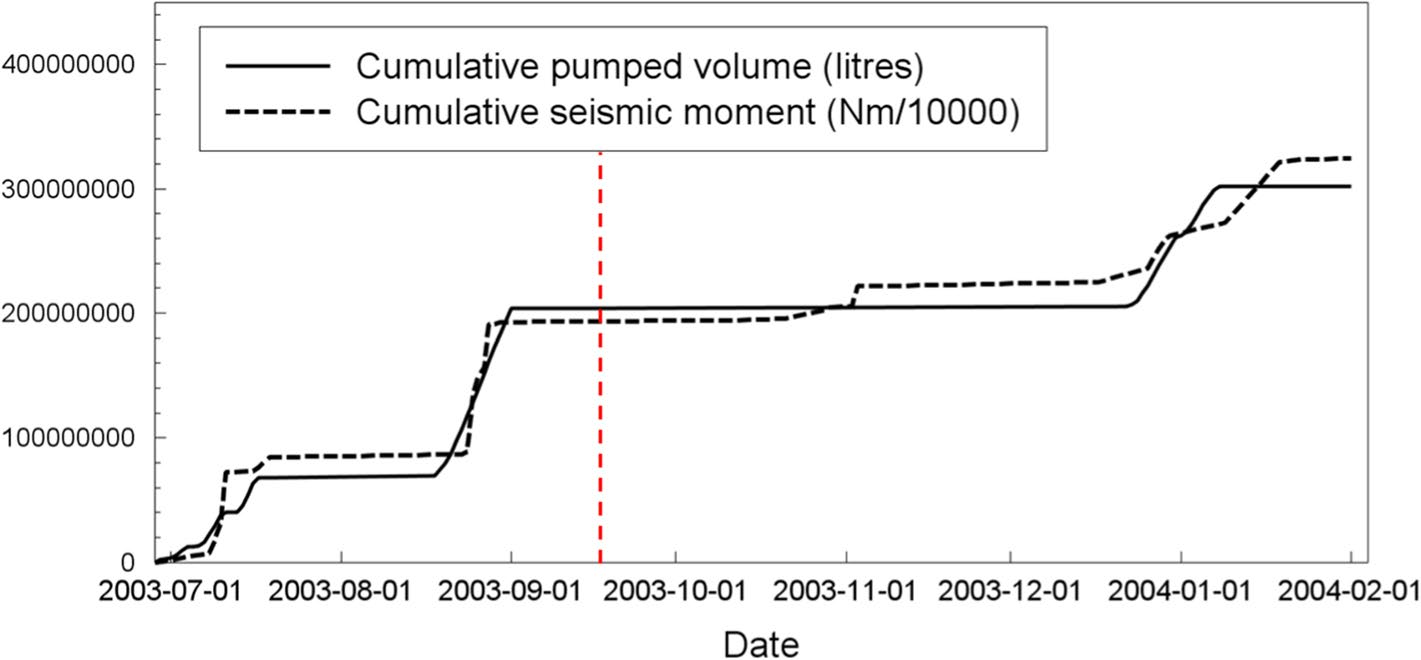
Cumulative seismic moment (dashed line) of seismicity in immediate vicinity of the Berlín HFR injection well and cumulative injected volume of water (solid line) (Bommer et al. 2016); the dashed red line indicates the M_L_ 4.4 event of September 2003, which occurred outside of this cluster; its seismic moment plots off the scale of the y-axis

The TLS for the Berlín EGS was, to my knowledge, the first documented example of a traffic light scheme to control induced seismicity. There was considerable seismic activity of small magnitude in the immediate vicinity of the well, which correlated extremely well with the injected volume of fluid when characterised by cumulative moment release (Fig. 97). As can be seen in Fig. 97, the operations of the HFR involved three periods of hydraulic injections (the first to hydraulically stimulate the formation along the open-hole interval below the casing, the second period to better characterise the shallow reservoir formation accessed below the casing shoe, and the third to stimulate the deeper reservoir level). The TLS was not triggered during the operations but the largest earthquake, of M_L_ 4.4, occurred on 16 September 2003, during the interval between the second and third injection phases. The event was located at about the same depth as the injection well and about 3 km to the south but is assumed to have been caused by the injections. The occurrence of this event two weeks after the shut-in of the second injection phase raised questions regarding the value of the TLS for this project. However, the occurrence of relatively large seismic events after shut-in of pumping, whether because operations are completed or because of a red traffic light, has been observed in many other geothermal projects (e.g., Majer et al. 2007) as well as in several HF injections (e.g., Baisch et al. 2019). Indeed, such ‘trailing’ events, as they are known, are quite common, and their occurrence is entirely consistent with the propagation of increased fluid pressures to a critically stressed fault.

**Fig. 98:**
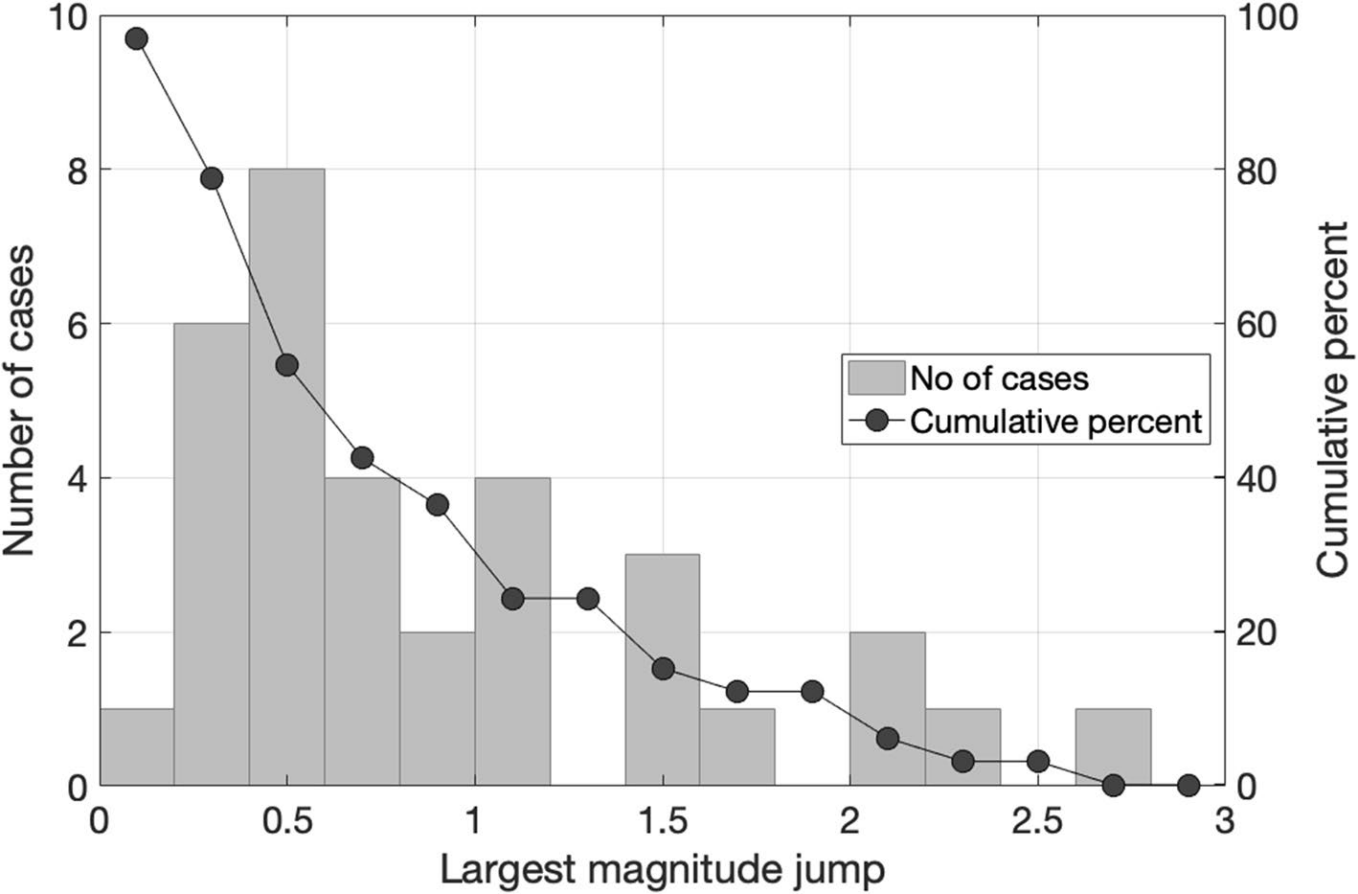
Observed magnitude jumps during induced seismicity sequences caused by hydraulic fracturing (Verdon and Bommer 2021a)

**Fig. 99:**
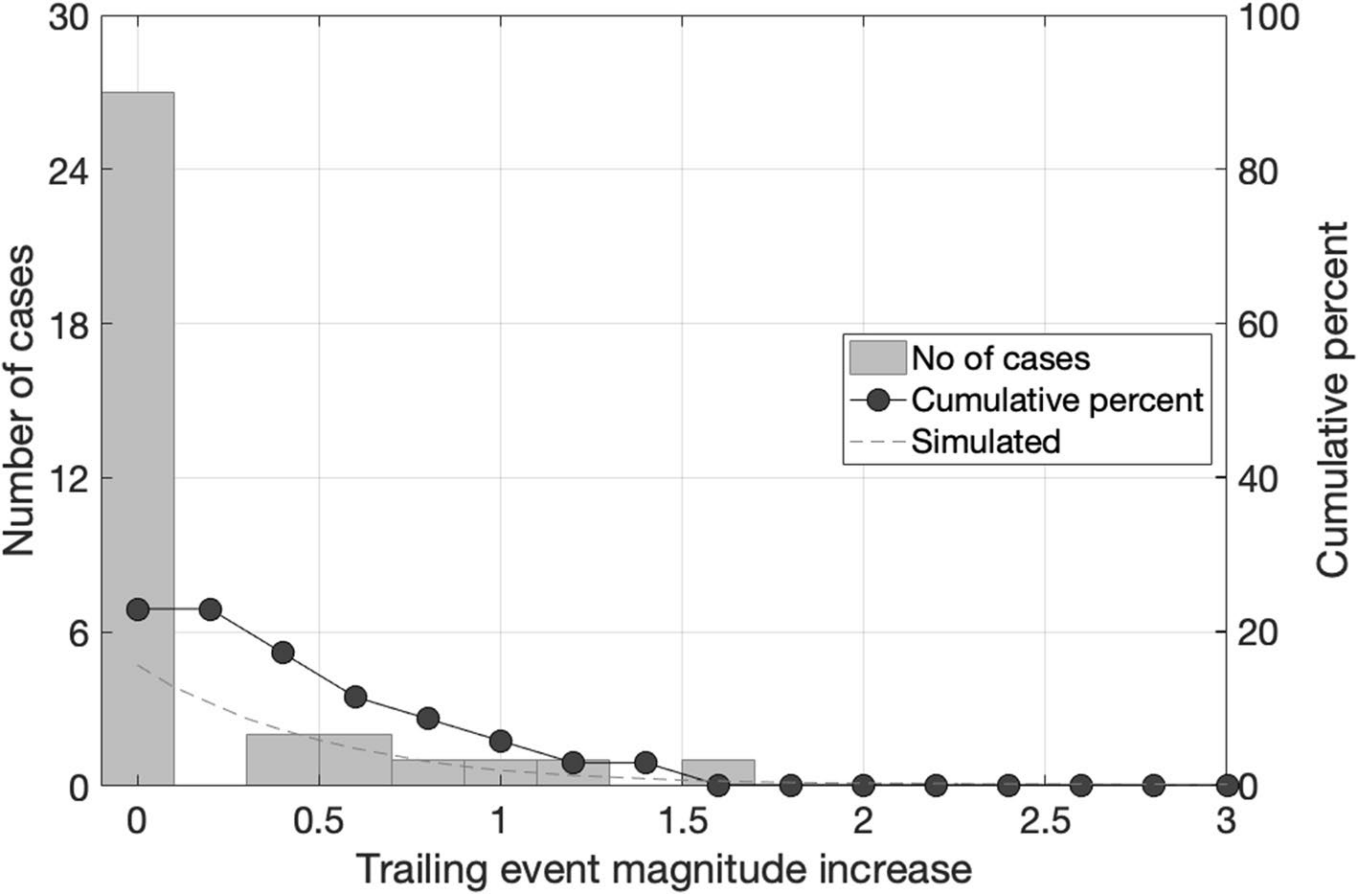
Observed magnitude increases associates with post shut-in trailing events caused by hydraulic fracturing (Verdon and Bommer 2021a); the dashed line shows the theoretical distribution calculated using the approach of Schultz et al. (2020b), assuming a Gutenberg-Richter recurrence relationship with a* b*-value of 1 and that 20% of the population of 1000 earthquakes occur after shut-in

These observations led me to conclude that TLS are not an effective risk mitigation tool for induced seismicity. However, I have since been persuaded that trailing events should not be viewed as invalidation of the concept of TLS but rather as a feature that should be built into the design of these systems. Verdon and Bommer (2021a) compiled data from 35 TLS operations for HF wells in Canada, China, the UK and the US, to study the statistics of the largest magnitude jumps in the induced seismicity sequences (Fig. 98) and the largest magnitude increases of trailing events above the largest events during injections (Fig. 99). The largest observed magnitude jumps are on the order of 2.5 units, but such cases are rare, and may also correspond to cases relying on regional rather than dedicated local seismograph networks, hence there can be some doubt regarding the detection threshold for smaller events. For 60% of the observed cases, the maximum jump in magnitude was of 1 unit or smaller, and for 23% of the cases the jump was between 1 and 2 units (Fig. 98). In terms of trailing events, in three quarters of the cases, there was no post shut-in increase of magnitude, and in a further 17% of the cases, the increase was of 1 unit of magnitude or smaller. The maximum post-injection increase in magnitude was 1.6 units, which occurred in a single case. An important point to note is that there were no cases for which there was both a large jump in magnitude during the injections and a further magnitude increase following shut-in. We concluded, therefore, that it should not be necessary to consider both of these effects in the design of a TLS.

The interpretation of these statistics needs to be made bearing in mind the fact that they correspond to a dataset that is in many respects likely to be a biased sample, because they correspond to cases where there was induced seismicity and moreover the cases with magnitude jumps and trailing events are more likely to have been documented. With this in mind, the observations can inform suitable gaps between the red-light magnitude and the magnitude limit that is to be avoided. The yellow-light threshold then needs to be set to provide a suitable margin for preventative measures to be implemented in the case of escalating seismicity, without setting this value so low that there are repeated interruptions of the injections that render the project untenable.

Verdon and Bommer (2021a) also examined the time delays between shut-in and the occurrence of the largest trailing events. For three-quarters of the cases, the largest events occurred during the injections, and in less than 10% of the cases did the largest event occur more than one week after shut-in; these observations can help to determine for how long a TLS should operate.

Similar data could be gathered from TLS operations for EGS, either to expand the database, if the two datasets are considered to be mutually consistent, or else to separately inform the design of TLS for injections related to enhanced geothermal systems.

### Physical mitigation of seismic risk

**Fig. 100:**
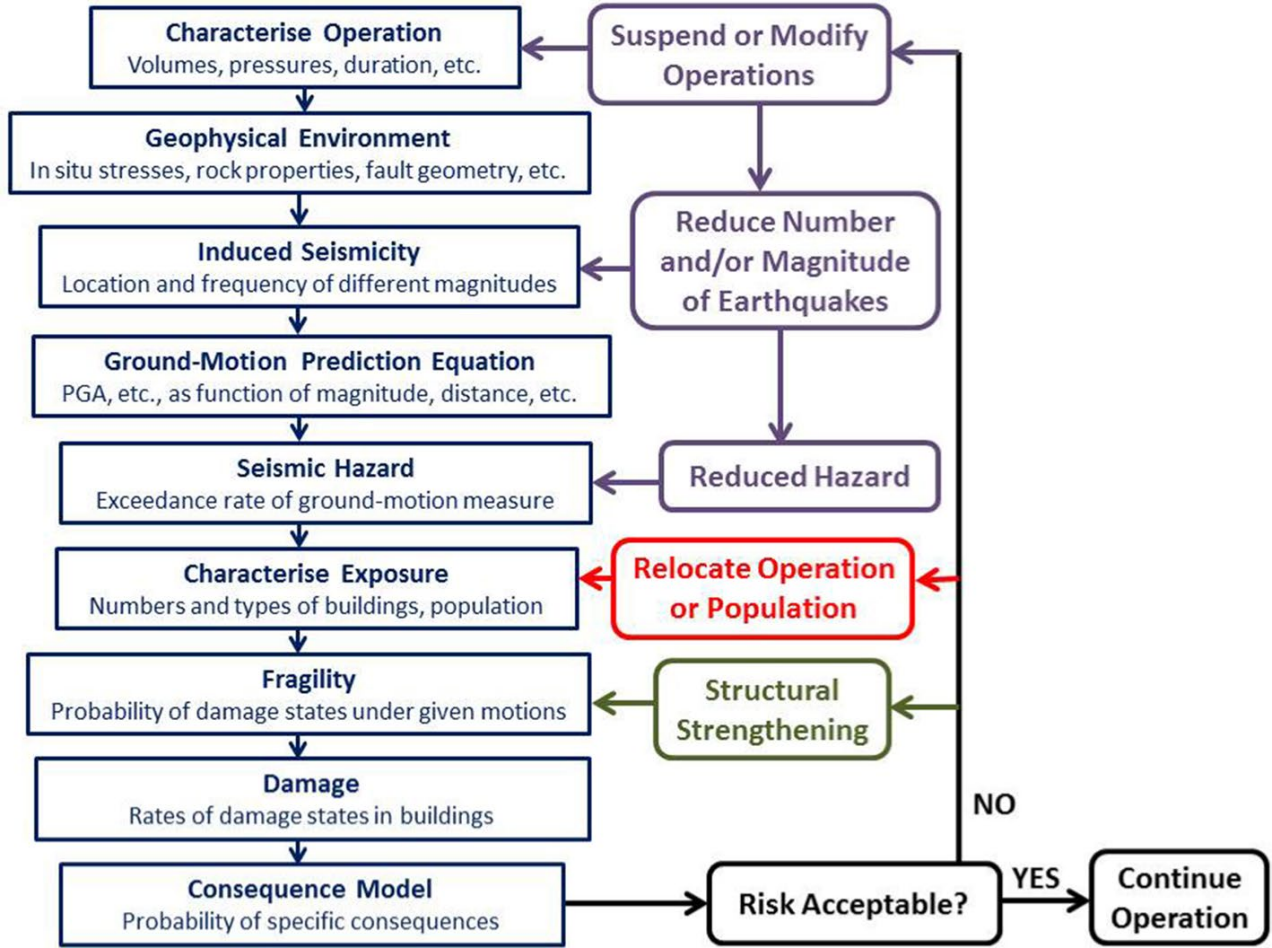
Steps to evaluate seismic risk due to induced earthquakes (blue boxes on left-hand side) and measures that can be taken, individually or in combination, to mitigate the risk (Bommer et al. 2015a)

Although a skilfully designed TLS can be an effective tool for mitigating induced seismic risk, it will generally not provide guarantees of safety (unless the yellow- and red-light thresholds are set to unmanageably low levels). Additionally, there are many anthropogenic operations for which TLS are unlikely to be effective, including reservoir impoundment and conventional hydrocarbon production. Therefore, while the opportunity to modify the hazard is an obvious and attractive option, there is no reason why the application of traditional earthquake engineering should not also be considered as a risk mitigation strategy, if this can be economically justified. Figure 100 illustrates the steps involved in the assessment of induced seismic risk (left-hand column) and the options that are available for mitigation of this risk. Structural strengthening can involve providing additional strength, ductility or both, for resistance of strong shaking; minor damage under lower levels of shaking can be mitigated through increased stiffness. Options for structural interventions, which can be applied globally to the structure or to individual elements, and their relative merits and disadvantages, are discussed in Bommer et al. (2015a) and references therein.

To implement an effective scheme of structural strengthening as a strategy for the mitigation of induced seismic risk, it is necessary to estimate the expected levels of ground shaking due to potential induced earthquakes, estimate the existing risk, and devise a strategy that targets the most at-risk structures with interventions that balance the required enhancement of the seismic resistance of the buildings with the cost of the measures and also with the disruption to the inhabitants. However, it is also important to emphasise that relatively simple structural interventions that would not require detailed dynamic analyses and would require minimal disruption for the inhabitants, could, in many cases, provide adequate protection against damage that could pose a threat. Additional protection can be afforded by simple measures to secure items within a house, such as strapping heavy items to studs and installing latches to prevent items falling (e.g., Greer et al. 2020).

Whenever the benefits of the industrial process are viewed to be highly valuable and extended interruptions to the process to control induced seismicity need to be avoided, the mitigation of risk through earthquake engineering is a logical choice. The use of building strengthening, potentially combined with modifications to operations, as a tool for risk mitigation against induced earthquakes was a key element in the proposed strategy to manage the risk due to induced seismicity in the Groningen gas field in the Netherlands. As discussed in Sect. 12.4, however, this strategy was not advanced sufficiently because of determined political campaigns to close the gas field instead, leading to the loss of a unique opportunity to demonstrate the rational and effective management of risk due to induced seismicity.

### General rules versus application-specific measures

In many jurisdictions, regulators of processes such a hydraulic fracturing have specified that a TLS must be operated, and the specifications generally include the magnitudes that define the yellow and red levels. While this is a reasonable approach, a case could also be made for the regulation to be goal setting rather than prescriptive, establishing tolerable risk levels in terms of consequences of induced earthquakes rather than the characteristics of the earthquakes themselves. Possibly, rather than the regulator choosing between a goal-setting approach and a prescriptive approach, these could be offered as alternatives. To implement a risk-based approach would require a certain degree of technical expertise that operators may need to engage externally, and the assessment of risk-based strategies also places a similar onus on the regulatory authority. However, there are significant potential benefits from such an approach: for operators, it can avoid unnecessarily stringent controls when the risk exposure is minimal and for the public it can encourage a more focused assessment of the elements at risk and the protection that they require.

**Fig. 101:**
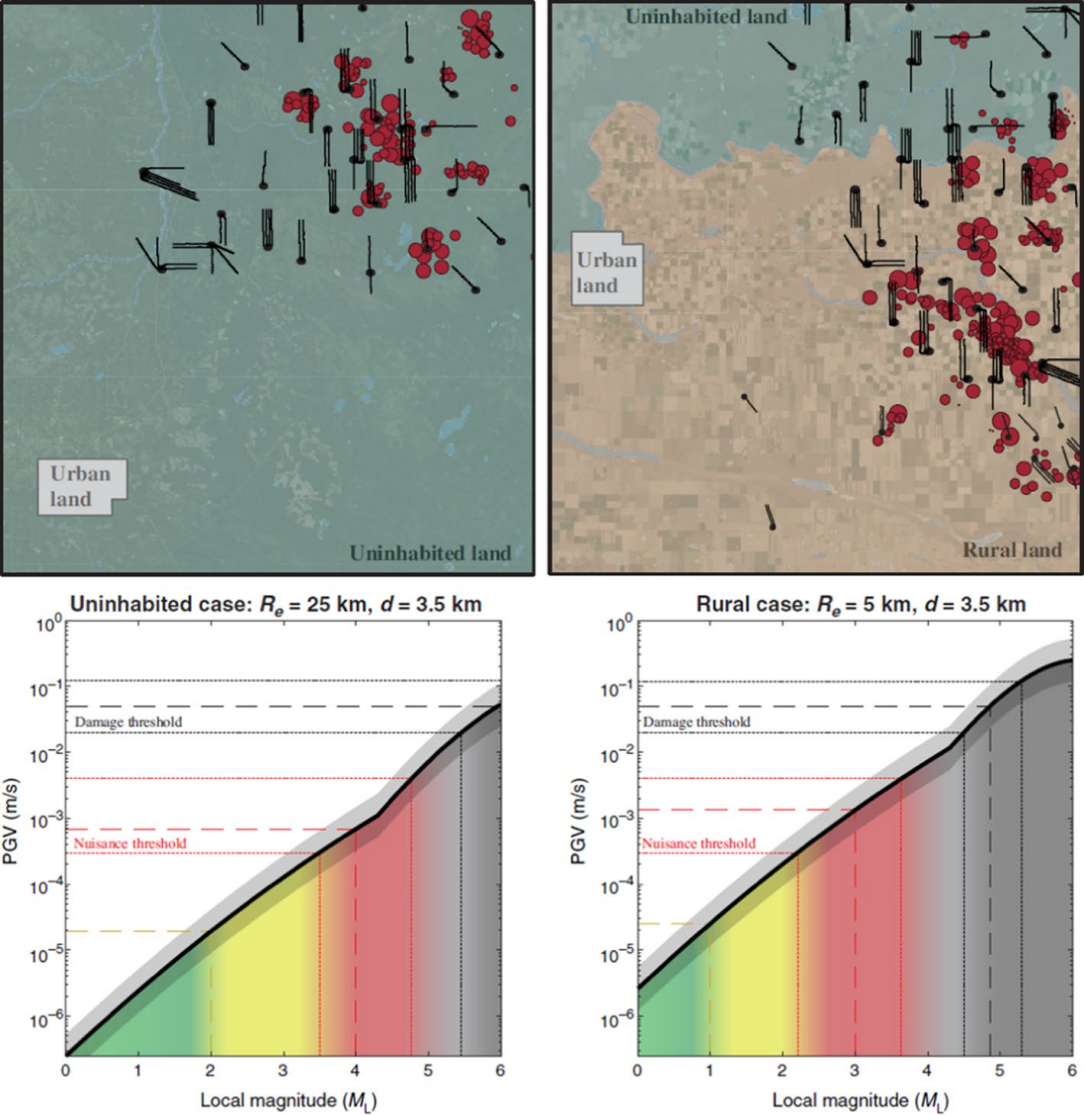
Upper: Hypothetical scenarios considered by Schultz et al. (2020b) considering a largely unpopulated region (left) and a partially settled rural area (right); Lower: Relationships between PGV and M for these same two cases, which are controlled by the R_e_, the equivalent epicentral distance calculated according to the distance to the closest buildings, the average number of inhabitants per building, and the population density; in both cases, it is assumed that the earthquake occur a depth of 3 km. The figure indicates how the magnitudes for the nuisance and damage thresholds are very different for the two cases (Schultz et al. 2020b)

An important consideration in determining a risk management strategy is the seismic fragility of the exposed building stock or infrastructure. Baird et al. (2020) determined magnitude thresholds for potential damage to modern constructions in the US as a function of distance, which could be used to infer the limiting magnitude thresholds that a TLS should be aiming to avoid in an area with this type of building, depending on the location of the structures relative to the injection wells. Schultz et al. (2020b) and Schultz et al. (2021b) proposed that the determination of the red-light magnitude threshold for TLS be based on a full seismic risk assessment considering the exposed building stock and its fragility, as well as local site conditions that could lead to amplification of the ground shaking. The approach leads to different TLS magnitude thresholds depending on the population density in the area and the fragility of the exposed structures (Fig. 101).

The key point is that all elements that contribute to the risk should be considered in the formulation of the mitigation plan, not only the control of the hazard through operation of a TLS or through general limits on injections or extraction rates. Johnson et al. (2021) estimated the risk, in terms of economic losses, due to induced seismicity caused by wastewater injections in Oklahoma. Their study concluded that strategies to limit the seismicity through controls on the injected volumes can be effective in controlling the ground shaking hazard, but that this was not necessarily the most effective way to reduce the losses. They identified the distance between the injection wells and the exposed building stock to be a key factor influencing the losses, leading to the conclusion that one of the most effective options could be to relocate injection wells away from populated areas, even by a few kilometres. This is consistent with the risk modelling approach of Schultz et al. (2020b).

The most effective risk mitigation strategies will depend on the specific characteristics of the industrial operations that might cause induced seismicity and of the exposed building stock. An optimal suite of measures might include location of injection wells as far as possible from dense settlements, strengthening of the most vulnerable exposed buildings (or even replacing these—an option that was followed in Groningen for a particular group of poorly constructed buildings erected by a particular contractor), together with a TLS to monitor seismicity and modify operations as necessary.

**Fig. 102:**
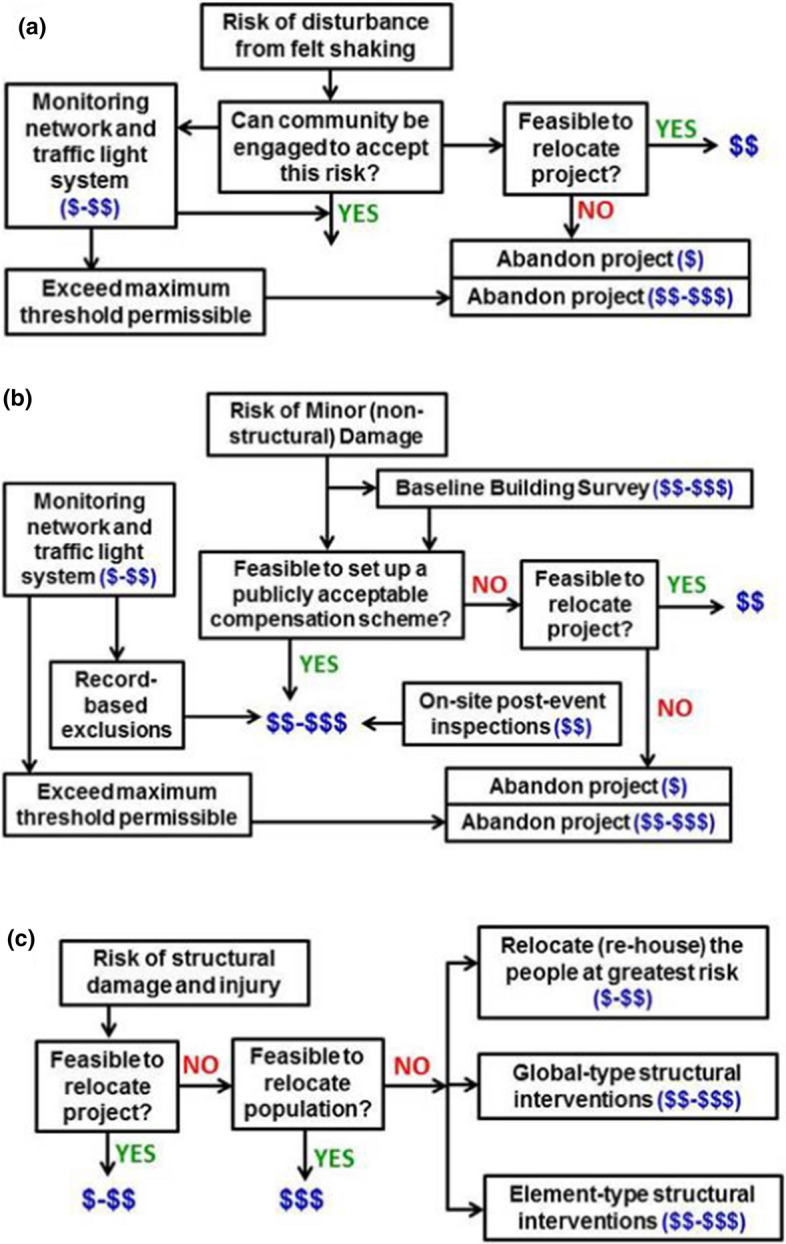
Options for risk mitigation schemes to mitigate **a** felt shaking causing nuisance, **b** non-structural damage incurring repair costs, and **c** structural damage that could pose a threat to the building occupants (Bommer et al. 2015a); the range of relative costs associated with each alternative are indicated ($: low; $$: medium: $$$: high), noting that abandoning a project that is operational is much higher than abandonment following a feasibility assessment

In exploring risk mitigation options for induced seismicity, Bommer et al. (2015a) differentiated potential schemes on the basis of the risk target, depending on whether the objective was to avoid disturbance to the exposed population, prevent minor (non-structural) damage, or only to protect life and limb against structural damage (although it goes without saying that the risk mitigation strategy could address more than one of these objectives). Mitigation options at the higher risk levels could include relocation of the project and/or the most exposed population, or else a programme of building strengthening. At the lower levels the measures could include engagement of the exposed population (likely to be more feasible for ‘green’ energy options such as geothermal than for hydraulic fracturing for hydrocarbons, although local employment opportunities could influence the attitude) and monetary compensation for minor damage (Fig. 102). Although I am not aware of such a scheme ever being implemented in practice, financial incentives could also be used to manage nuisance risk: a threshold magnitude for which it would be expected that the shaking would be felt by many people in the local area (but without causing damage) could be defined, and every household in the exposed area would then receive a nominal, but not trivial, sum for each such occurrence. Such a scheme was proposed for the Groningen gas field by Bal et al. (2019), which might sound somewhat outlandish to some but in practice could have been a much more rational and equitable approach than the damage claim and compensation scheme that has evolved in that situation (see Sect. 12.4).

The final point to make is that the risk mitigation strategy designed prior to the commencement of the injections or other operations should be updated and modified in the light of information gathered during the operations. The data gathered could include earthquake locations and magnitudes, which can be correlated with operational factors (for example, to calculate the seismogenic index, which can then allow projections of future seismicity rates), recorded ground motions, and observed performance of local buildings under the recorded shaking levels. For the TLS developed for the Berlín HFR project, for example, the GMM used to calibrate the model was obtained by adjusting a published equation for PGV to match recordings from small-magnitude volcanic swarm recordings obtained in the region; as the injections proceeded and recordings were obtained from the induced events, residual analyses were conducted in order to make adjustments to the initial GMM, which was found to overestimate the recorded amplitudes (Bommer et al. 2006).

## Can small-magnitude earthquakes cause damage?

As discussed in the previous section, effective mitigation of induced seismic risk through TLS hinges on defining magnitude thresholds that could result in damage to buildings. From the perspective of earthquake-resistant design of new structures, the influence of events of magnitude smaller than 4.5 or 5.0 is usually disregarded through the lower bound magnitude, M_min_, imposed on PSHA calculations (see Sect. 3.2). However, it is acknowledged that for estimating risk to existing building stock, particularly in regions with low levels of natural seismicity, the magnitude thresholds for damaging events could be lower. For the rational management of induced seismicity, determining these magnitude thresholds is of fundamental importance. Even though the levels will depend on the characteristics of the exposed building stock, the local ground conditions, and the distance at which these buildings are situated from the potential locations of induced earthquakes, I believe it can be very useful to make general inferences from observations of small-magnitude earthquakes. To this end, in this section I briefly discuss observations of damage due to small-magnitude natural earthquakes, which can serve as a proxy for induced earthquakes of the same magnitude if one accepts the premise that the two types of event produce comparable ground motions in the epicentral region (see Sect. 9.3). I believe the body of evidence that observations from small-magnitude tectonic earthquakes present should not be ignored, especially since such events are vastly more abundant that their induced counterparts. Case histories of small-magnitude induced earthquakes reported to be damaging are discussed in Sect. 12; one of the purposes of the current section is to provide a point of reference and comparison for the induced case histories. For clarity, I do not believe that the potential for moderate-magnitude triggered events, such as the **M** 5.8 Pawnee earthquake in Oklahoma or the **M** 5.5 Pohang earthquake in Korea, to cause appreciable damage is open to debate; the question being addressed here is whether earthquakes smaller than, say **M** 4.5, can be expected to lead to damage in buildings and infrastructure.

The section begins with a brief discussion of the ground-motion characteristics that influence damage. This is then followed by an overview of empirical observations from small-magnitude tectonic earthquakes and the impact of the ground shaking on buildings and other structures. The section closes with a brief discussion of collateral hazard associated with small-magnitude earthquakes.

### What makes ground motion damaging?

There is no simple answer to this question since it depends on the characteristics of the structure being shaken, both in terms of its linear vibration properties and its non-linear behaviour, and also on the structural response metric used to quantify damage. A literature review of studies that have sought to answer these questions using both analytical and experimental approaches could quite easily occupy the full length of the paper. Nonetheless, I will attempt to offer some general observations and insights on this topic since it has important implications for the damage potential from small-magnitude earthquakes.

**Fig.103:**
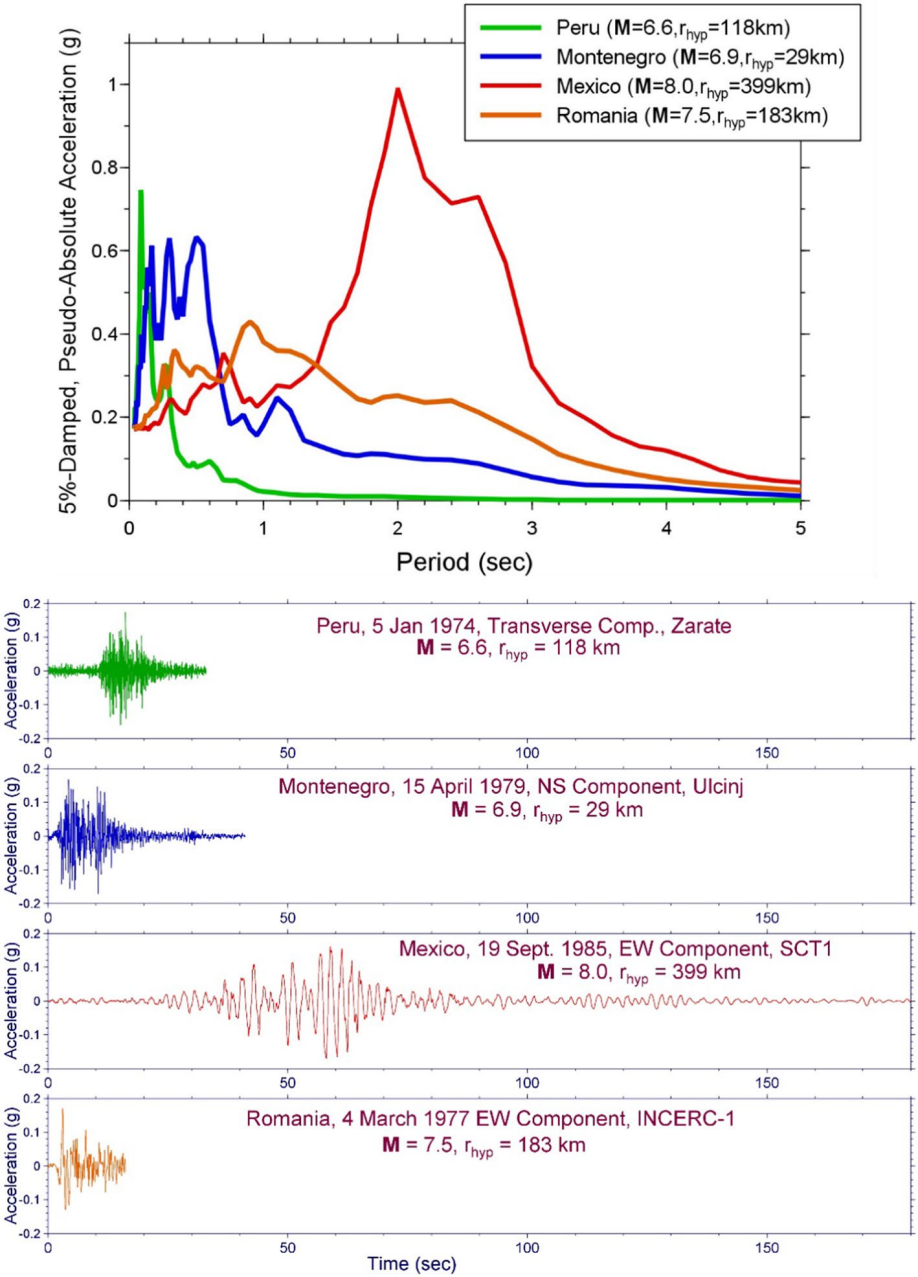
Four horizontal accelerograms with identical PGA values (lower) and their 5%-damped pseudo-acceleration response spectra (upper). Adapted from Bommer and Boore (2005)

As was already mentioned in Sect. 2.2, no single parameter can fully represent the characteristics of a ground-motion recording and its capacity to cause damage. One reason for this is that the response of any structure will be strongly influenced by the relationship between its own natural frequency of vibration and the frequency content of the ground motion. Figure 103 shows four accelerograms with exactly the same value of PGA but very different acceleration response spectra (which all have the same intercept at the PGA value of 0.18* g*). These ground motions had very different impacts: the Peru earthquake was destructive to low-rise housing but had very little impact on high-rise structures, whereas the Michoacán earthquake caused extensive damage to medium- and high-rise buildings in Mexico City, where the motions were amplified by thick deposits of lacustrine clays, but had limited effect on low-rise buildings in the city (e.g., Celebi et al. 1987).

As well as the different frequency contents, as revealed very clearly by their response spectra, the four accelerograms in Fig. 103 also display clear differences in the duration and number of cycles of motion. While the influence of duration on geotechnical effects such as liquefaction is clearly recognised, its influence on structural damage is still very much a matter of debate (e.g., Hancock and Bommer 2006). The influence of duration is more apparent in structures that have degrading inelastic properties (i.e., stiffness and/or strength that reduces with increasing cycles of motion), such as unreinforced masonry. For example, Bommer et al. (2004a) found a clear influence of duration on damage to masonry when the damage was measured in terms of loss of strength and the primary characteristic of the motion was the average spectral acceleration over an interval from the initial natural vibration period to a period about three times longer. However, it was also noted that the duration and the averaged spectral acceleration of the records were correlated, which could partially mask the influence of the duration. In order to isolate the influence of duration, Hancock and Bommer (2007) used a suite of records spectrally matched to the same response spectrum but with a wide range of durations, an approach which has subsequently been adopted by others (e.g., Chandramohan et al. 2016). Spectral matching uses wavelets to adjust an accelerogram such that its response spectrum matches a defined spectral shape, with minimal changes to the acceleration time-histories (Hancock et al. 2006), which can reduce significantly the number of dynamic analyses required to obtain stable estimates of non-linear structural response (Hancock et al. 2008). Hancock and Bommer (2007) used the spectrally matched records to analyse the response of an 8-storey reinforced concrete building, finding that peak response metrics, such as maximum drift, were unaffected by duration, but that cumulative damage metrics were influenced by the duration of the motions.

**Fig. 104:**
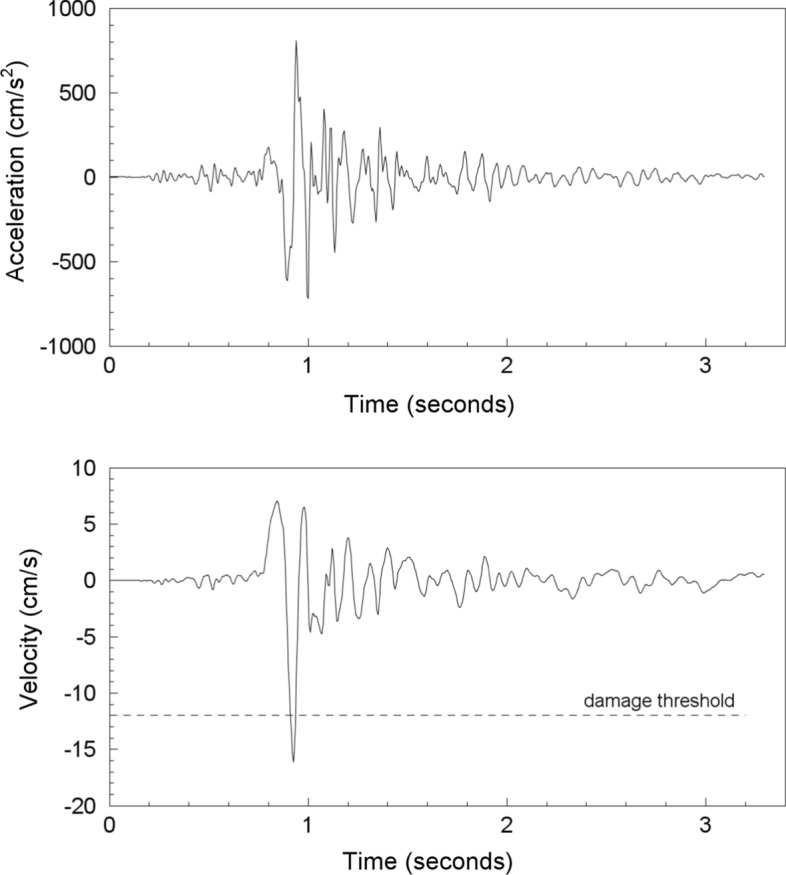
Recorded acceleration and velocity traces from the M_L_ 4.4 induced earthquake associated with an enhanced geothermal project in El Salvador (Bommer et al. 2006)

Although the extent to which duration (combined with some other parameter) influences building damage remains somewhat ambiguous, the length of the strong shaking interval—and consequently the energy that it carries—does provide an explanation for why motions from smaller earthquakes that have high peak amplitudes do not appear to be destructive. By way of illustration, Fig. 104 shows an accelerogram recorded very close to the epicentre of the M_L_ 4.4 earthquake associated with the Berlín HFR geothermal project (Sect. 10.1). The horizonal PGA was on the order of 0.8* g* and the PGV value 16 cm/s, the latter exceeding the damage threshold defined for the TLS. However, no damage occurred as a result of this event, demonstrating that while we can define thresholds for individual ground-motion parameters to becoming damaging—such as 0.2* g* for PGA and 20 cm/s for PGV—these can only be considered as necessary but not sufficient conditions. In other words, the fact that ground motion has a high PGA does not automatically mean that it is damaging. Indeed, this is the very reason why M_min_ needs to be defined in PSHA: if there were no ground motions that had high PGA values but were not damaging, the M_min_ parameter would not be needed.

**Fig. 105:**
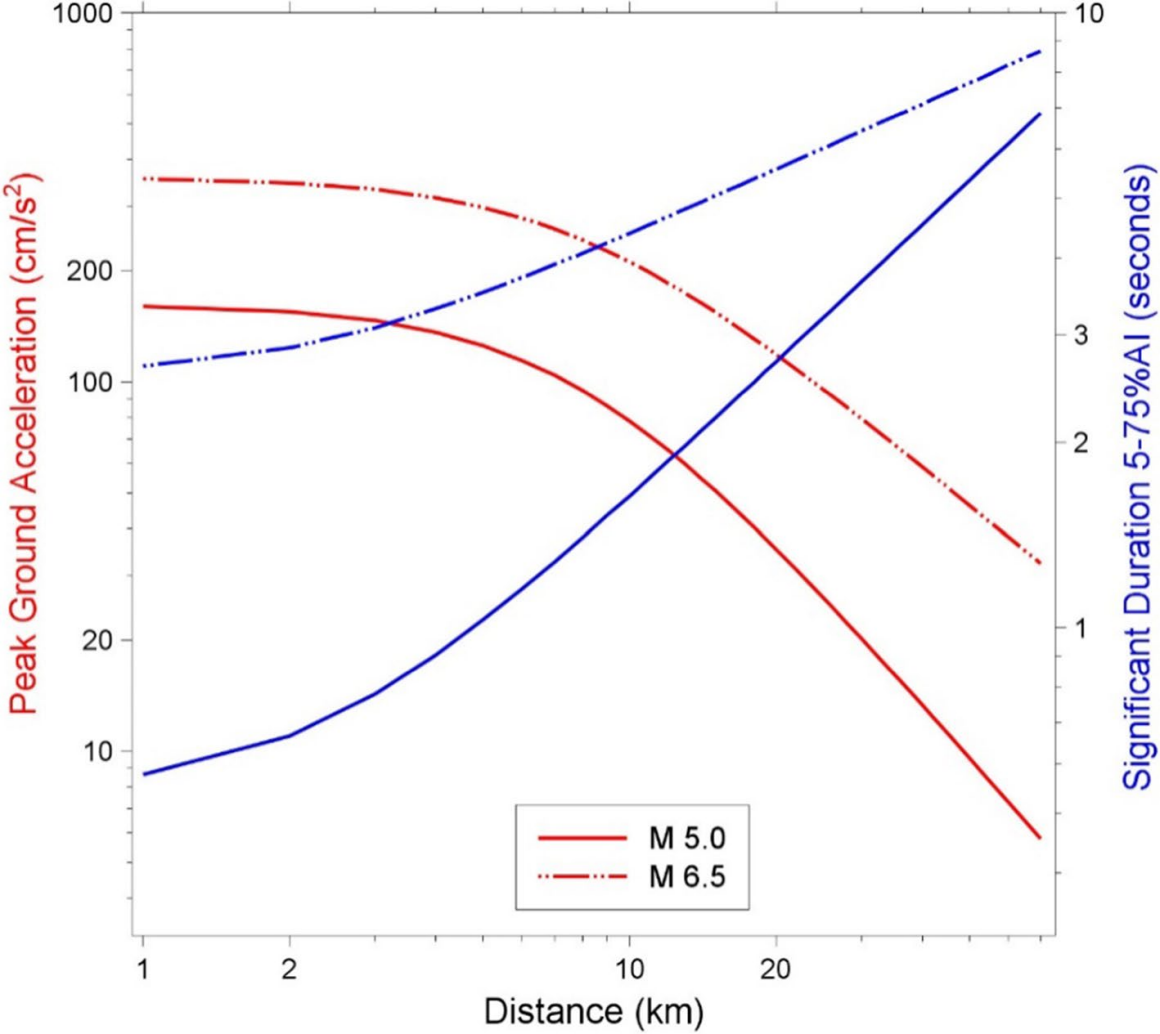
Predicted median PGA values from the GMM of Akkar and Bommer (2010) and significant duration from the GMM of Bommer et al. (2009) for rock sites and strike-slip earthquakes of **M** 5.0 and 6.5 on vertically dipping fault ruptures that extend to the ground surface

If the duration of the signal is an important factor, then it needs to be accounted for in studies based on dynamic analyses of structures. The amplitude and duration of ground motions both scale with the earthquake magnitude but they have opposite trends with distance (Fig. 105). If the target for analysis is the epicentral motions for an induced earthquake of M 4, say, then if records are selected from earthquakes of this size recorded at distances of up to ~ 10 km, an inconsistency can arise. When those motions are scaled up to match the epicentral PGA values, the significant duration will remain the same and the combination of amplitude and duration will actually correspond to a larger earthquake. This is exacerbated by the fact that the residuals in predictions of PGA and duration are negatively correlated (Bradley 2011). This means that if the PGA corresponds to an 84-percentile value (i.e., one standard deviation above the mean prediction), then the associated duration would be expected to be appreciably lower than the mean prediction. This negative correlation simply reflects the finite energy content of the ground motion, which in the epicentral area is controlled mainly by the magnitude of the earthquake; to produce a motion with an exceptionally high amplitude, the signal needs to be compressed in terms of duration. Consequently, if accelerograms from earthquakes of M 4 recorded at distances of ~ 10 km are scaled to match predicted epicentral amplitudes at say the 2-sigma level for an induced earthquake of the same magnitude, the resulting ground motion will have a duration greatly in excess of what would be expected for such a scenario; the impact estimated from such high-energy scaled motions could therefore appreciably overestimate the impact of the scenario earthquake.

### Damage due to small-magnitude natural earthquakes

**Fig. 106:**
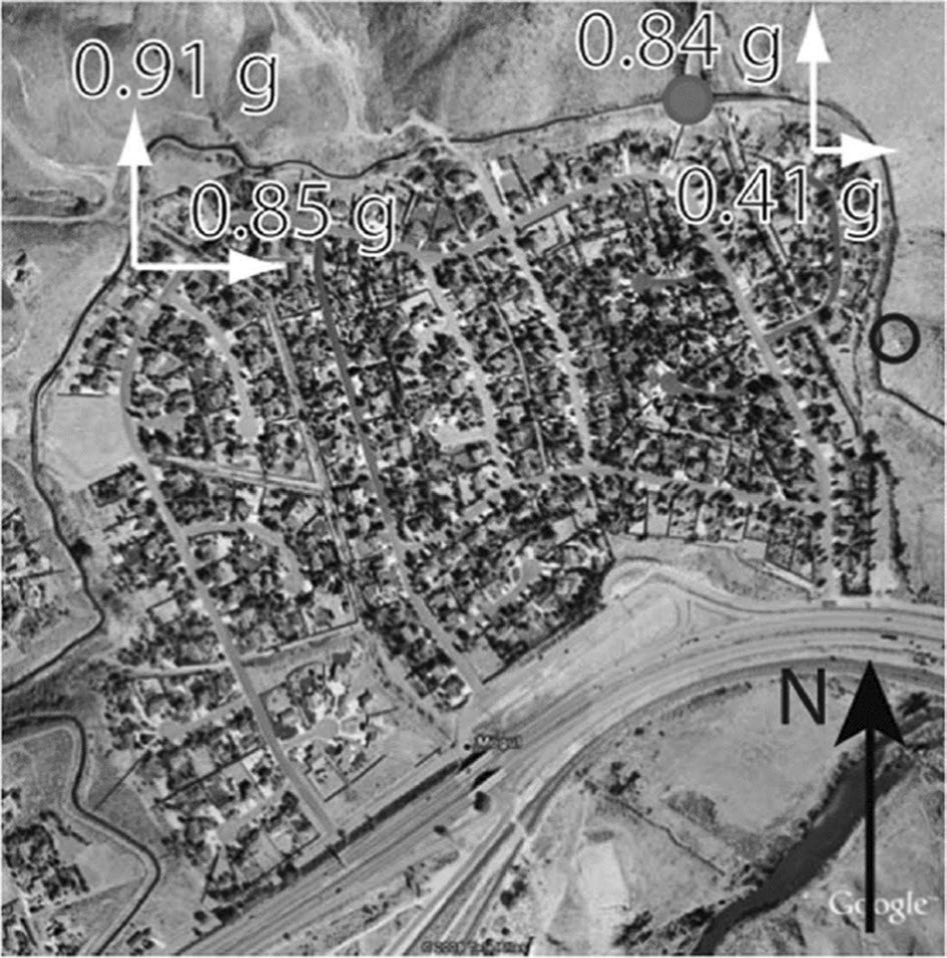
Aerial image of Mogul showing the location of the epicentre (shaded circle) and the horizontal PGA values recorded at the MOGE (east) and MOGL (west) accelerograph stations (Anderson et al. 2009); the open circle on the east side is the location a small rockfall on a steep and heavily fractured granite slope

In my Joyner Memorial Lecture, I defined a small earthquake as being of less than magnitude 5, whereas in the introduction of this section I set the threshold at **M** 4.5—mainly because all of the case histories discussed in Sect. 12 of this article concern earthquakes of magnitude below 4.5 (and in most cases much smaller). An interesting case in point here is the **M** 5.0 earthquake—an event right on the boundary I have proposed for defining small earthquakes—that struck Mogul, a small suburb of Reno, Nevada, on 26 April 2008. The epicentre was located at the northeast limit of the town and the focal depth was calculated as just 3 km. Two accelerographs located within Mogul recorded very large horizontal PGA values (Fig. 106); the vector of the horizontal components at the MOGL station had a PGA of 1.2* g*. There are some 270 houses in Mogul and Anderson et al. (2009) reported the following with regard to their performance in the earthquake: “*There were no deaths in Mogul and no reports of injuries requiring medical treatment. None of the houses experienced damage that prevented continued occupancy. To our knowledge, only two structures, both with living space over the garage, experienced minor (but costly) structural damage. In both cases, the sole plate of the wood frame in a corner of the garage was nailed to a mud sill that was bolted to the stem wall, and during the earthquake, the nails failed.*” In terms of the larger affected area, Anderson et al. (2009) stated that “*Several hundred homes constructed primarily since the 1980s were exposed to shaking in excess of 0.5 g. Very few sustained damage more significant than cracked plaster.*”

The MOGL station is actually located in the back garden of the home of University of Nevada seismology professor John Anderson, lead author of the Anderson et al. (2009) paper on the earthquake. Professor Anderson kindly sent me several photographs of the inside and outside of his house following the earthquake, noting that “*a large fraction of the contents of shelves and cupboards were thrown out onto the floor throughout the neighborhood. Pictures fell to the floor…… a leading engineer in the city, came and looked at the house just to see for himself what this high ground motion had done, and he didn't find any structural damage*” (J.G. Anderson, personal communication, 2020). In summary, the very high-amplitude, short-duration motions generated by this **M** 5.0 earthquake caused very little damage to well-built, code-compliant dwellings.

**Fig. 107:**
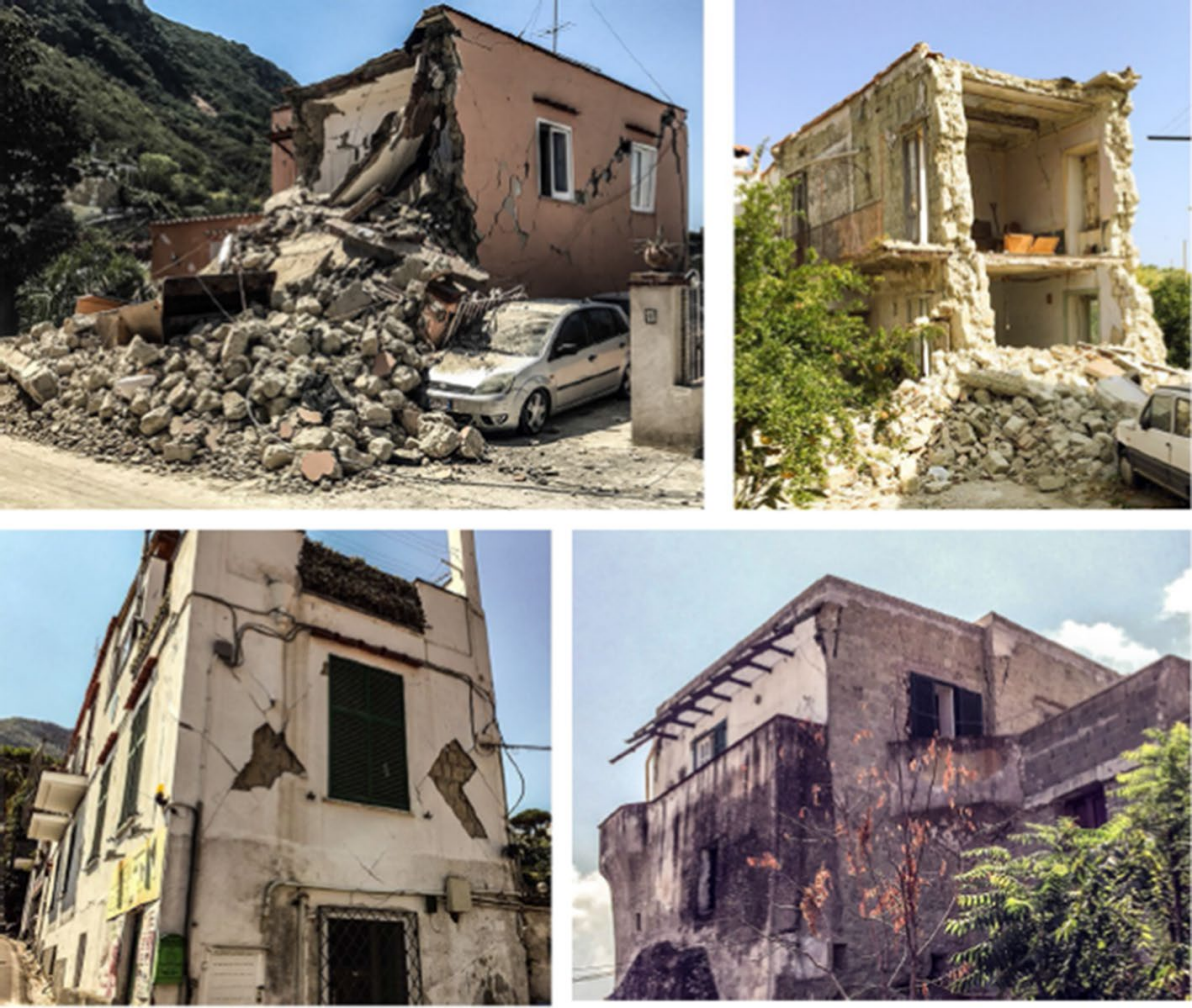
Upper and lower left: Examples of damage to unreinforced masonry buildings in the 2017 M 3.9 Ischia earthquake; lower right: undamaged masonry building with iron tie rods (Brisghella et al. 2019)

A starkly contrasting case is the **M** 3.9 earthquake that occurred on the island of Ischia, offshore from Naples in southern Italy, in August 2017. This volcano-tectonic earthquake had a focal depth of just 1.7 km and occurred directly below the town of Ischia. Several old and heavy unreinforced masonry structures were damaged, leaving two dead and 42 injured (Briseghella et al., 2019). Damage was limited to a small area of about 400 m radius, within which it is suspected that ground motions were possibly amplified by topographic effects since the damage mainly occurred on a hill in the epicentral area. Brisghella et al. (2019) attribute the main cause of damage to the very high building vulnerability, noting that no reinforced concrete structures were damaged and even the presence of iron tie rods in masonry buildings proved sufficient to prevent collapse (Fig. 107). Reports have highlighted that there was little control of construction in the affected area and that Ischia had been identified as an area where illegal construction is rife (https://www.thelocal.it/20170822/shocking-to-die-in-such-low-magnitude-earthquake-says-chief-geologist/).

**Fig. 108:**
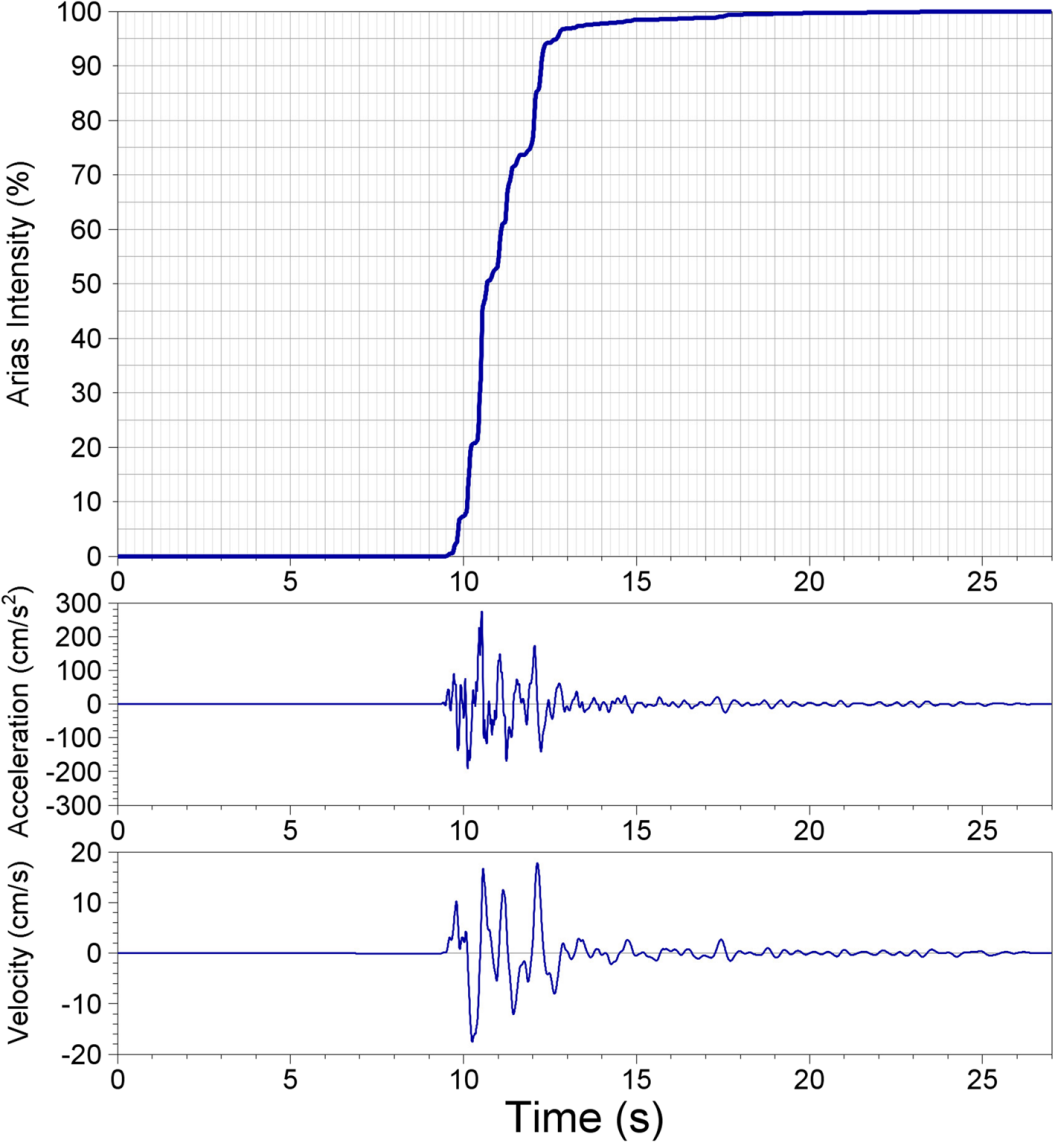
Acceleration and velocity traces of the EW component of the IOCA recording of the Ischia earthquake; the upper frame shows the Husid plot indicating the accumulation of Arias intensity against time; the records were obtained from the Engineering Strong Motion Database hosted by INGV (Lanzano et al. 2019)

**Fig. 109:**
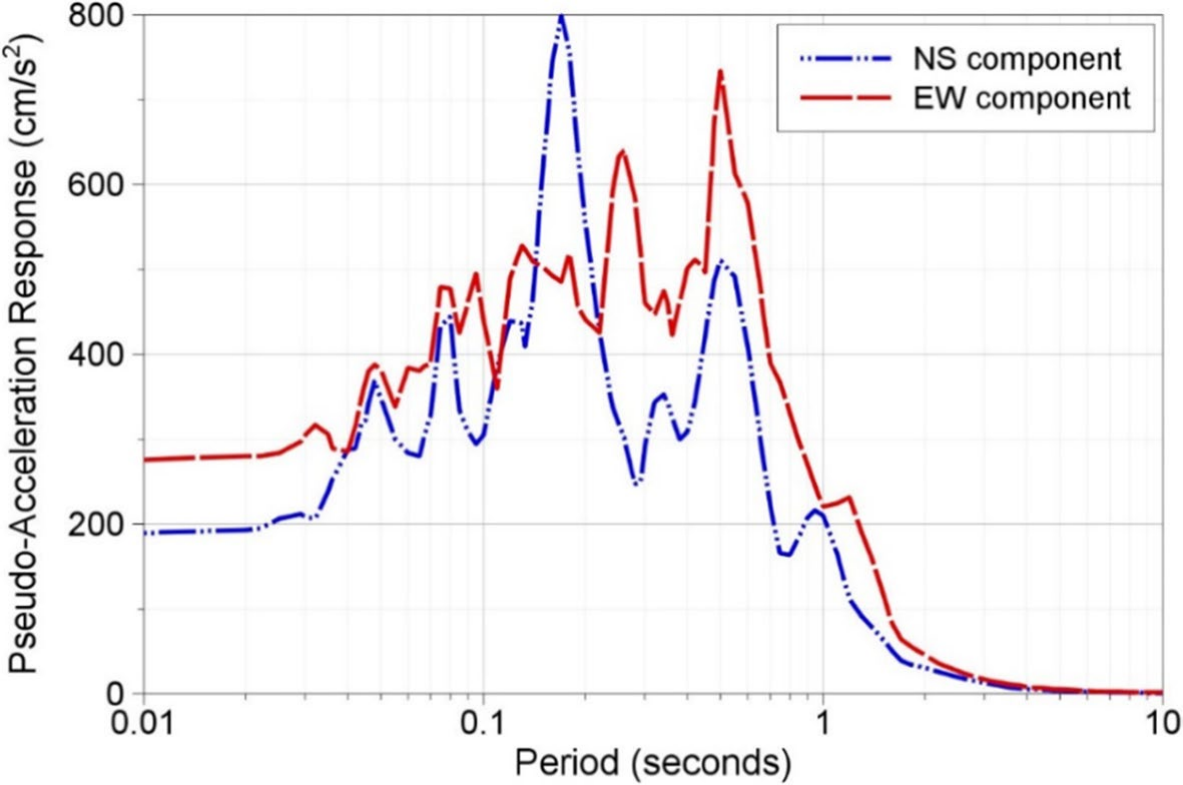
Acceleration response spectra with 5% of critical damping from the horizontal components of the IOCA recording of the Ischia earthquake; the records were obtained from the Engineering Strong Motion Database hosted by INGV (Lanzano et al. 2019)

The strongest recorded motions from this event were obtained at the IOCA accelerograph station located about 0.6 km north of the epicentre, showing moderate amplitudes and a duration (based on 5–75% accumulation of the total Arias intensity) of just over 2 s (Fig. 108). The amplitudes of the motion are not particularly high, but the motion does appear to be of unusually low frequency despite the classification of the IOCA station as Eurocode 8 site type B in the Eurocode 8 (V_S30_ 360–800 m/s); this is also reflected in the broad plateaus of the horizontal response spectra (Fig. 109). However, such low-frequency motions, which are quite distinct from those generated by tectonic earthquakes of similar size, have been identified as being typical of shallow volcano-tectonic earthquakes (e.g., Tusa and Langer 2016). These characteristics of the ground motions may have played a role in the exceptional impact of the Ischia earthquake, which is an outlier in terms of such a small event causing so much damage and even casualties. However, the field reconnaissance report by Briseghella et al. (2019) clearly indicates that the pronounced fragility of the heavy masonry structures that experienced damage was a major contributing factor to the severe impact of this earthquake.

To explore the impact of small-magnitude earthquakes, Nievas et al. (2020a) compiled a database of earthquakes with magnitudes in the range 4.0 to 5.5 for which there were reports of physical damage, economic losses, or injuries or deaths. For the period 1900 to 2017, almost 2000 earthquakes were identified, although the vast majority of these occurred during the twenty-first Century, reflecting the influence of the Internet in disseminating such information.

**Fig. 110:**
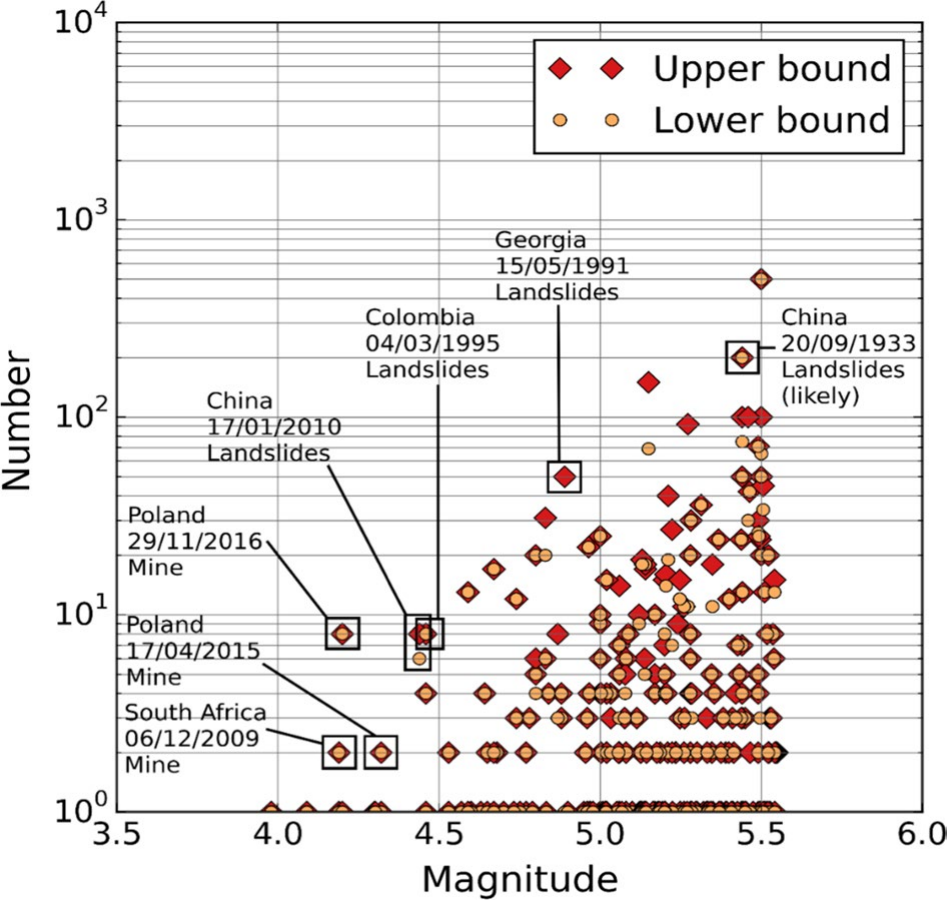
Numbers of reported deaths as a function of earthquake magnitude from the database of Nievas et al. (2020a)

In compiling such a global database, it is inevitable that depth is sacrificed for breadth, with the result that for many of the earthquakes there is very little information available. This raises an important consideration because in empirical science it is common to assert that absence of evidence is not evidence of absence. However, in the age of widespread ownership of smart phones and access to social media platforms, I believe that one could argue that the absence of evidence can, in many cases, be interpreted as evidence of absence. When people are posting images of the most banal occurrences in their lives, a report of ‘damage’ that is not accompanied by photographic evidence (unless it is in a very remote and/or underdeveloped region), could legitimately be treated with some suspicion (a theme that will be re-visited in Sect. 12). Moreover, the descriptors used to characterise the reported damage vary enormously and are rarely expressed in terms of established damage scales such as that defined for the EMS intensity scale (Sect. 2.2). In view of the ambiguity associated with reports of damage and even ‘destruction’ to buildings, the most reliable information in the database might be the reports of deaths caused by earthquakes. However, earthquake deaths attributed to heart attacks were excluded from the database since there are several studies that have demonstrated that rather than causing heart attacks to happen, earthquakes tend to cause heart attacks that were imminent to cluster in time (see Appendix 2 of Nievas et al. 2020a). In terminology that is probably familiar to most following the Covid-19 pandemic, the heart attacks that occur during earthquakes do not contribute to excess mortality relative the background rate when averaged over a longer period of time. Figure 110 shows the numbers of reported deaths for each event with reported casualties (about 14% of the database) as a function of the earthquake magnitude. As can be seen from the annotations in the plot, most of the events of **M** ≤ 4.5 causing more than one death are associated with mine collapses or with landslides. The former are clearly a special case and in some of these cases it is possible that the mine collapse itself was recorded by seismographs and assigned a magnitude, hence the actual cause of the ‘earthquake’ rather than a response to the shaking. The same may hold for some of the landslides but even when the landslides are a consequence of an earthquake, this may reflect cases of very susceptible slopes, especially if the earthquake occurred during a rainy season (these collateral hazards are discussed further in Sect. 11.3).

**Fig. 111:**
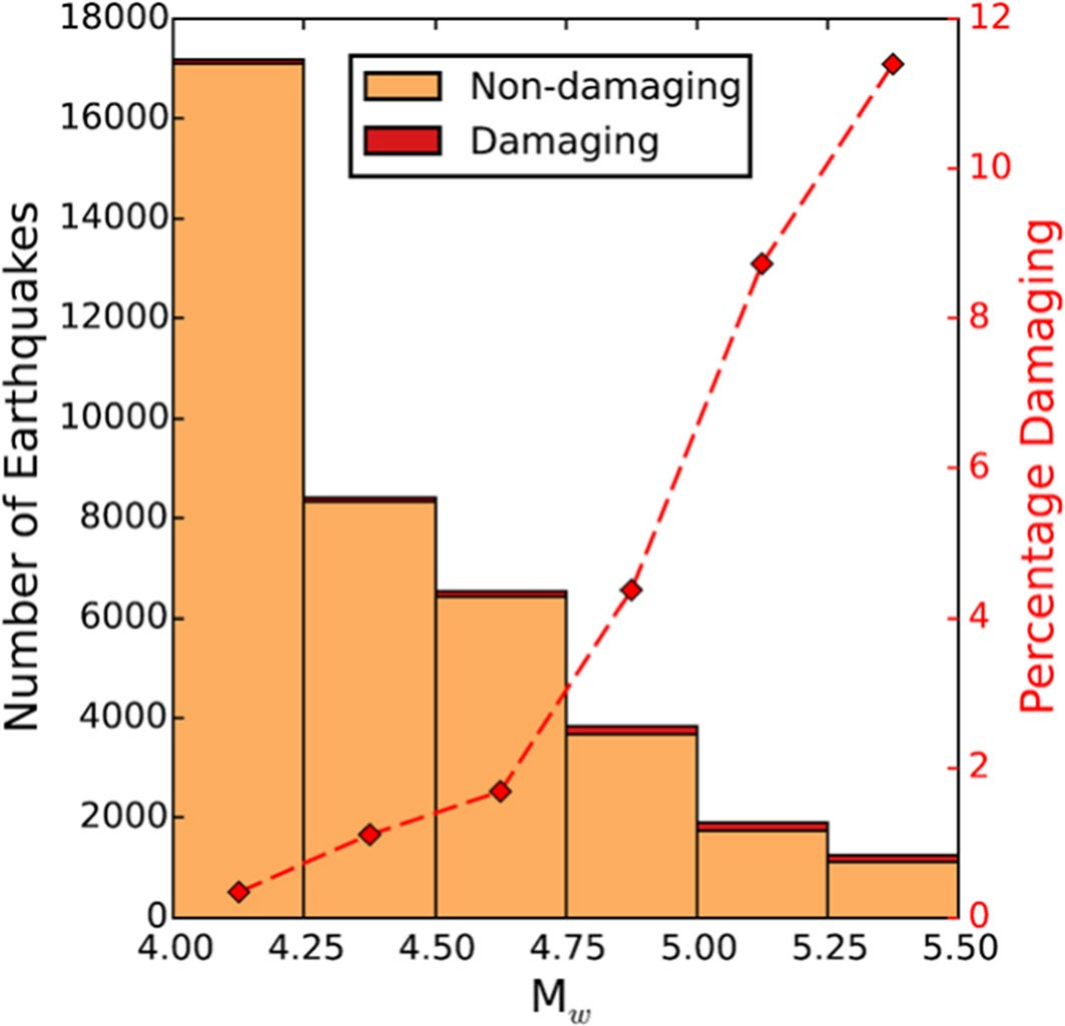
Numbers of potentially damaging earthquakes globally from 2001 to 2015 and those reported as damaging in the database of Nievas et al. (2020a), with the diamonds indicating the percentage of damaging events in each magnitude interval (Nievas et al. 2020b)

In a second study, Nievas et al. (2020b) sought to explore the proportion of earthquakes in this magnitude range that are reported to have caused damage. A global earthquake catalogue of earthquakes with magnitudes from 4.0 to 5.5 was compiled for the period from 2001 to 2015, which is the period during which the database of damaging events is considered to be the most complete. Of course, it is acknowledged that the database is not complete because of events that are not reported and also events reported in languages that we were unable to decipher, so in this sense the database of Nievas et al. (2020a) would define lower bounds on the proportion of small-to-moderate magnitude earthquakes that are damaging (although this may also be partially offset by the presence of ‘false positives’ in the database, corresponding to exaggerated and unsubstantiated reports of earthquake impacts). The global catalogue was then filtered to consist only of events that could potentially have impacted the built environment, eliminating deeper earthquakes, offshore events and those occurring in unpopulated or very sparsely populated regions. Figure 111 shows the distribution of the 39,000 events with respect to magnitude—which is consistent with the Gutenberg-Richter recurrence model—and also highlights the 740 events that are also included in the database of damaging events. Overall, the damaging events constitute just 1.9% of the total number of earthquakes, although if we focus only on 2013–2015, during which time the online Earthquake Impact Database (https://earthquake-report.com) was operating, the proportion increases to 4.3%. However, it is important to bear in mind that this includes events larger than **M** 5; if we focus only on events of magnitude **M** ≤ 4.5, only about 1% of the potentially damaging earthquakes are reported to have caused damage, injury or economic losses. As just noted above, this is most likely to be a lower bound, but it still points to damage from such small earthquakes being very much the exception rather than the rule. Detailed reports are available for very few of the events in this magnitude range, but on the basis of the information that is available it would seem that they generally correspond to cases of extreme vulnerability of the exposed buildings.

**Fig. 112:**
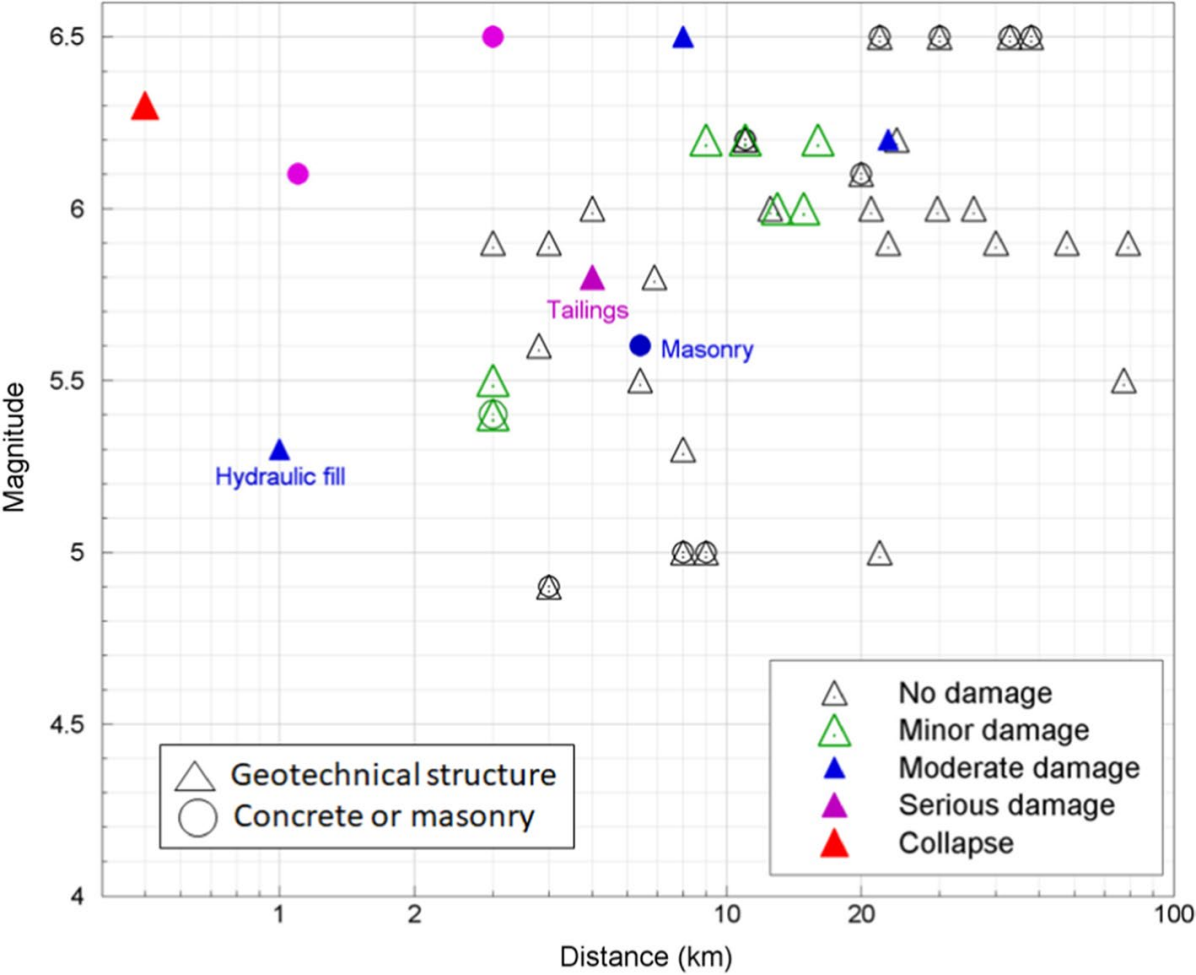
Case histories of dam performance in earthquakes; data retrieved from USCOLD (1992), USCOLD (2000) and USSD (2014) by John W France

Insights into the capacity of small earthquakes to cause damage can also be obtained from observation of structures other than buildings. Figure 112 shows observations of dam performance in earthquakes compiled by the US Committee on Large Dams (USCOLD) and the US Society on Dams (USSD). These data suggest that there are no documented cases of damage to dams in earthquakes smaller than magnitude 5. The smallest earthquake in those listings to have caused damage to a dam was a magnitude 5.3 event and the damage occurred in a dam constructed of hydraulic fill, which would have been a very susceptible structure. Also noteworthy is the observation that the only other two cases of moderate or serious damage caused by events of M < 6 involved a masonry dam and tailings dam, both also likely to be relatively fragile structures.

However, when this information has been presented, two exceptions to the conclusions inferred from the data in Fig. 112 have been noted, the first being the reported failure of the Earlsburn dam in Scotland due to an earthquake in 1839. Hinks (2015) states that the Earlsburn dam failed some 8 h after “*an earthquake thought to have had a magnitude of 4.8*”. This reflects the fact that for an event in the mid-19th Century, estimates of the magnitude will carry a high level of uncertainty. Hinks (2015) reports that the 6 m dam was constructed of earth and peat with a narrow core of silty clay and founded on peat. There is no reason to doubt that the failure of this dam was precipitated by an earthquake, but it would also appear that this was an exceptionally vulnerable structure.

**Fig. 113:**
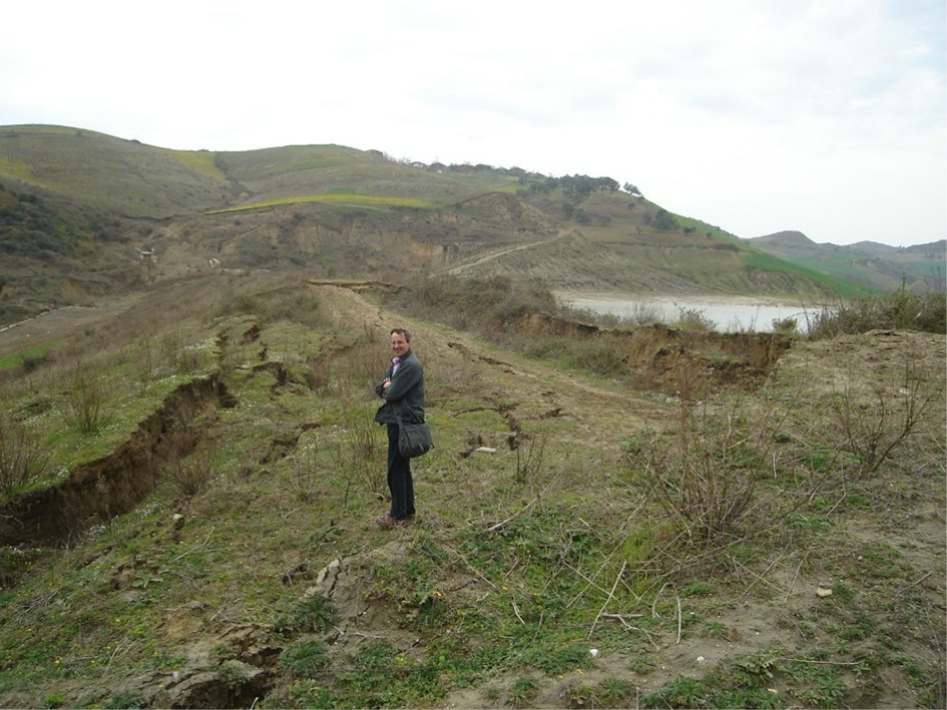
Damage to the Sharredushk dam caused by an earthquake of M 4.1 (Courtesy of Mr Tim Hill)

The second case asserted to invalidate the conclusions drawn from Fig. 112 is the 2009 Sharredushk dam failure in Albania due to an earthquake of magnitude 4.1 (Fig. 113); the earthquake epicentre was located about 1 km from the dam. The Sharredushk dam failure is discussed by Wieland (2019) and Wieland and Ahlehagh (2019) but communication with Dr Martin Wieland confirms that the information presented regarding the Sharredushk dam was provided by Jonathan Hinks (e.g., Hinks et al., 2012; Hinks, 2015). Mr Hinks is a civil engineer specialised in dam safety, previously employed at Halcrow, who has kindly shared information regarding the case that provides very useful insight. Under a World Bank-funded programme, Mr Hinks was engaged in work to assess many dams throughout Albania on behalf of the Albanian government. His report on the Sharredushk dam was issued in February 2004—five years before the earthquake—in which he noted that the dam was experiencing extensive internal erosion, which was manifesting in sink holes in the downstream face. The upstream face was protected by concrete slabs, many of which were broken, and there was also evidence of extensive erosion at the right abutment. The engineering report recommended extensive strengthening works in the form of buttresses at the right abutment and along the downstream face of the 136 m-long dam. These remedial works were costed at $0.7 M and were never implemented, possibly because the risk was considered low in view of the area immediately downstream being largely unpopulated.

**Fig. 114:**
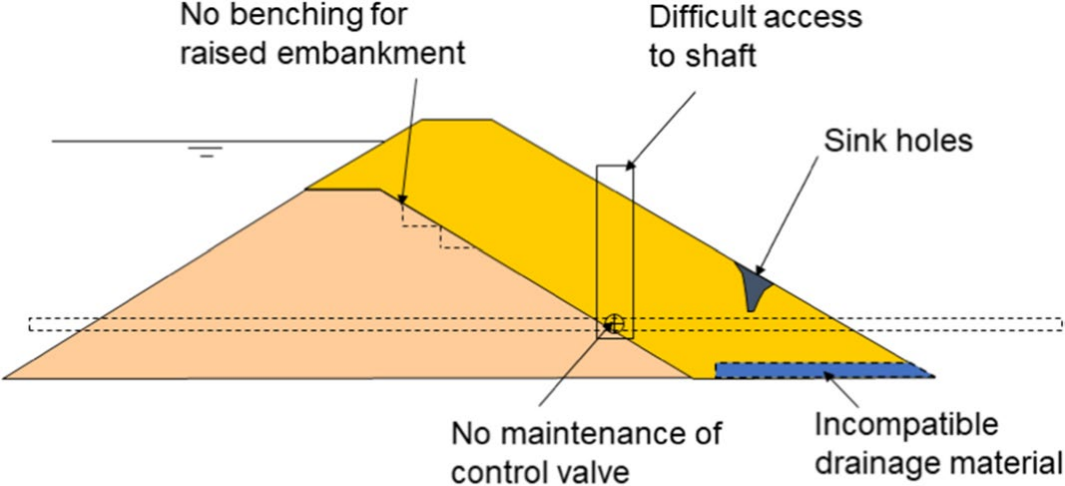
Cross-section of the Sharredushk dam showing original (beige) and raised (yellow) sections and also indicating existing sink holes in the upstream face (Courtesy of Mr Tim Hill)

The work on behalf of the Albanian government for assessing the dams was subsequently taken up by Mr Tim Hill, a dam engineer employed at Mott MacDonald, who was able to provide me with additional information about the dam, including reports from colleagues who visited the dam following the failure. The dam was originally constructed in the 1960s to a height of 60 m, as a homogeneous clay structure (i.e., no separate core) and was subsequently raised by another 6 m (Fig. 114). Mr Hill explained that the topsoil on the upstream slope was not removed prior to the raising of the dam and no benching was created, resulting in a plane of weakness along the interface between the original dam and the raised section.

Another key problem with the design of the dam was identified as incompatible drainage material. The design for the raising works recognised the importance of keeping the phreatic surface as low as possible in the downstream shoulder to maintain structural stability. This objective is usually achieved through a blanket drain placed below the downstream shoulder, which is a horizontal sand layer, generally on the order of about 1 m in thickness. An essential feature of the drain is to have a fine-grained sand layer in contact with the fill material and then a coarse sand layer, which creates compatibility between the materials. In the case of the Sharredushk dam, the drain—which was made up of individual ‘fingers’ rather than a continuous blanket—was composed only of coarse sand. This meant that fine material from the shoulder fill was washed into the filter material and reduced its permeability to the extent that it effectively ceased to function as a drain. The sinkholes observed in the downstream face provided evidence of the washing out of fill material, which created a zone of weakness at the toe of the dam.

**Fig. 115:**
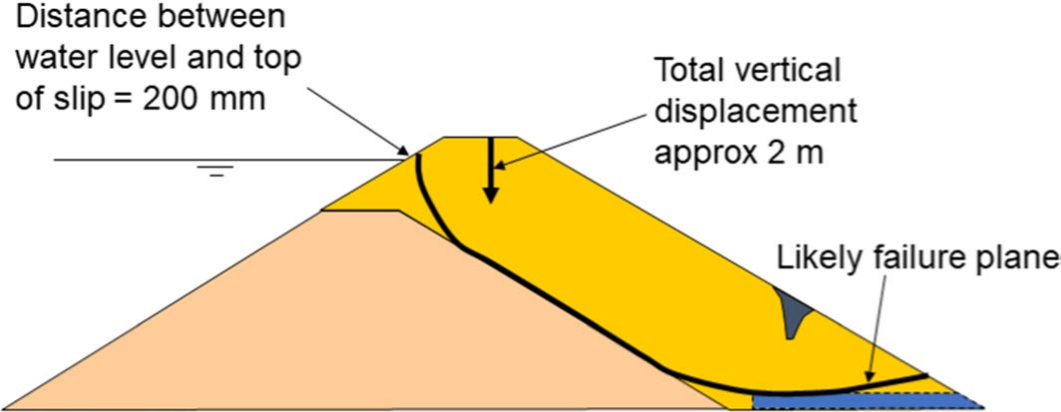
Cross-section of the Sharredushk dam indicating total vertical deformation initiated by the earthquake and the likely failure plane on which the slip occurred (Courtesy of Mr Tim Hill)

The earthquake occurred on 18 March 2009, at the end of a very wet winter when the reservoir was completely full and starting to overtop the spillway. With the drains no longer functioning, the phreatic level within the dam would have been high. With the toe weakened by internal erosion and the plane of weakness between the original and raised sections of the dam, the earthquake shaking was characterised by Mr Hill as simply “*the straw that broke the camel’s back*.” The inferred failure mode is indicated in Fig. 115. Fishermen reported small cracks that appeared in the crest the day after the earthquake, and the slip that resulted in 1.5–2 m vertical deformation happened six days after the earthquake.

In conclusion, this case history corresponds to a case of extensive damage to dam associated with an earthquake of magnitude less than 5. However, it is also clearly a case of an extremely susceptible dam that professional engineers who inspected the site before and after the event concluded would have likely failed even without the seismic shaking.

In summary, leaving aside the exceptional case of the Ischia earthquake and the unusual ground motions associated with shallow-focus volcano-tectonic earthquakes, the overall picture that emerges from this review is that damage from earthquakes of magnitude **M** ≤ 4.5 is rare. In those rare cases for which there appears to have been damage as a result of such small events, it would appear to be generally indicative of very weak and vulnerable structures rather than any inherent capacity for the ground shaking to cause destruction—a conclusion that at least partially applies also to the Ischia case. For adequately built structures, such small earthquakes would generally appear not to pose a threat, even if they do generate peak motions of high amplitude.

### Collateral hazards due to small-magnitude earthquakes

To close this discussion of the potential for small-magnitude earthquakes to cause damage, I wish to briefly discuss collateral hazards other than the direct effects of ground shaking. These collateral hazards were identified in Fig. 7 and discussed in Sect. 2 of the paper. These secondary earthquake hazards are worth discussing in the context of induced earthquakes since I have seen some of them raised as potential threats associated with such events and if we are to achieve rational assessment of induced seismic risk, I believe it is helpful to focus our attention where it matters and not to be side-tracked by distractions. Towards this end, I very briefly discuss each of the four main collateral hazards: surface rupture, tsunamis, landslides, and liquefaction.

Surface rupture for earthquakes of **M** ≤ 4.5 can be confidently dismissed as a credible hazard. In the first instance, there is a probability much smaller than 5% that earthquakes of such magnitude produce ruptures that reach the ground surface (Youngs et al. 2003), although for shallow-focus induced earthquakes, the probability might conceivably be a little higher. However, even if the rupture does reach the surface, the expected maximum displacements on the fault would be on the order of 1–2 cm (e.g., Wells and Coppersmith 1994). Serva et al. (2019) conclude that the smallest earthquakes for which surface rupture hazard needs to be considered is **M** 5.5.

Since small-magnitude earthquakes are unlikely to produce surface rupture, they are also very unlikely to generate tsunamis. The size and destructive potential of a tsunami is determined by the volume of sea water that is displaced by an offshore fault rupture. The rupture dimensions and fault displacement associated with earthquake of **M** ≤ 4.5 are far too small to generate tsunamis that could even be detected. Indeed, magnitude **M** 6.5 (which is ~ 1,000 times more energetic) would generally be considered the minimum threshold for tsunamigenic earthquakes (https://www.usgs.gov/faqs/what-it-about-earthquake-causes-tsunami).

As Fig. 110 indicates, the possibility of landslides due to earthquakes as small as **M** 4.5 cannot be dismissed. However, it must be borne in mind that landslides frequently occur without any external loading, due to earthquake or other sources, the primary trigger being rainfall. Therefore, slopes that are very susceptible to instability as a result of heavy rainfall, erosion, excavation for road construction or deforestation, may fail under very low levels of shaking. However, empirical relations between the distance to the farthest triggered landslides (or the area affected by landsliding) and magnitude, suggest that for magnitude **M** 4.5 landslides would only occur very close to the epicentre (e.g., Rodriguez et al. 1999). Therefore, if in the immediate vicinity of a project that could potentially cause induced seismicity, highly susceptible slopes are identified, the possibility of shaking-triggered instability should be considered as part of a holistic risk assessment.

**Fig. 116:**
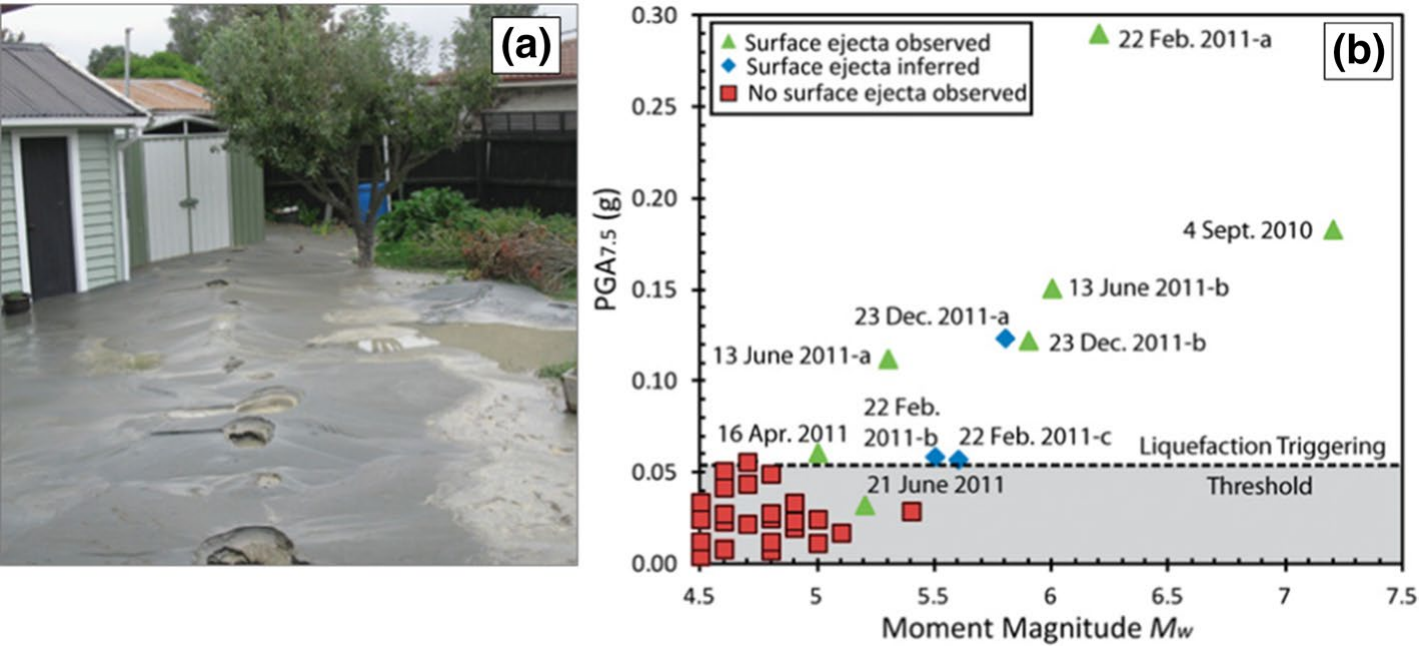
**a** Observed effects of liquefaction triggered in a suburb of Christchurch, New Zealand; **b** earthquake events in the 2010–2011 Christchurch sequence indicating whether liquefaction triggering occurred at the location in (**a**) (adapted from Quigley et al. 2013). The PGA values are the equivalent values adjusted for a magnitude **M** 7.5 earthquake

The final collateral hazard to consider is liquefaction triggering, which depends on both amplitude and duration of the shaking, as discussed in Sect. 2.3, for which reason the magnitude of the earthquake is known to play an important role in determining whether or not liquefaction occurs. Prompted by claims that liquefaction hazard was an important threat associated with induced seismicity in the Groningen gas field (Sect. 12.4), Green and Bommer (2019) undertook a survey of reported cases of liquefaction, which was then supplemented by simple modelling of representative soil profiles that could be considered highly susceptible to liquefaction. Among the field observations reviewed, one that stands out particularly is the study by Quigley et al. (2013) conducted for the Christchurch earthquake sequence in New Zealand. Liquefaction occurred in the backyard of Dr Quigley’s home in the Avonside suburb of eastern Christchurch, and he was able to make on-site inspections following each episode of felt shaking (Fig. 116). The observations, summarised in the right-hand panel of Fig. 116, showed that the smallest earthquake for which liquefaction triggering was observed was of magnitude **M** 5.0. This is consistent with the general conclusions of Green and Bommer (2019): the smallest earthquakes for which liquefaction triggering has been observed were of **M** 4.5 and there is no evidence for liquefaction occurring in smaller earthquakes. However, in all of the cases for which earthquakes of this size produced liquefaction, the phenomenon took place in extremely susceptible ground, such as marshy riverbanks or beach deposits. The smallest earthquakes to have caused liquefaction triggering in ground that could support any type of construction were of **M** 5, which therefore defines the threshold for consideration in risk analyses—as assumed many years earlier by Atkinson et al. (1984).

The one outlier among the cases reviewed by Green and Bommer (2019) was the case of the 1865 Barrow-in-Furness earthquake. Musson (1998) describes dramatic liquefaction effects in this event, for which he estimates a magnitude in the range from 2.5 to 3.5. Whether the problem lies with the estimated magnitude for this earthquake or with the observations attributed to liquefaction effects, Green and Bommer (2019) concluded that the information is unreliable and that it would be unwise to define the threshold magnitude for liquefaction triggering on the basis of such tenuous information, especially since nothing remotely comparable has been reported for any earthquake of comparable size in the century-and-a-half that have since elapsed. Based on global earthquake recurrence rates on land, this case either corresponded to a 1-in-10,000,000 event or else is an unreliable data point. Musson (2020) responded to our conclusion, acknowledging that the case was “*problematic and highly anomalous*” but insisting that “*facts are facts and should not be dismissed no matter how rare and anomalous an occurrence*”; the interested reader may wish to peruse the comment by Musson (2020) and our reply (Green and Bommer 2020) and draw their own conclusions.

## The consequences of induced earthquakes

**Fig. 117:**
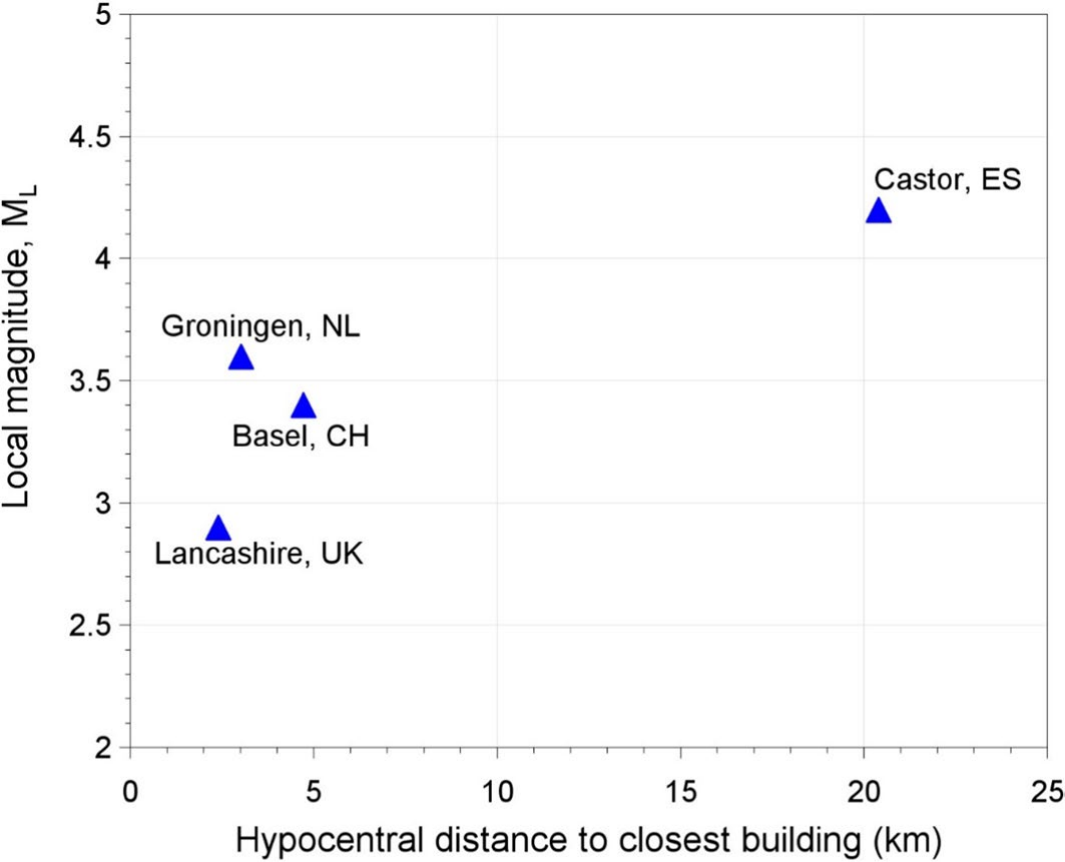
Maximum magnitudes in each of the four case histories discussed in Sect. 12, plotted against the hypocentral distance to the closest exposure

In this section, I discuss four case histories of induced seismicity and the impact that these induced earthquakes have had on the built environment and on the people who experienced the shaking, as well as on the industrial activities that caused them. I have chosen these cases on the basis of all having been reported to have caused damage or otherwise generated notoriety; in all four cases, the induced seismicity resulted in the operations being cancelled. However, such far-reaching consequences arose from earthquakes of rather modest sizes: Fig. 117 shows the maximum magnitude of each seismic sequence and the approximate distance of that event from the closest exposed building. From the discussion in Sect. 11, one could conclude that damage due to earthquakes of magnitude less than 4.5 is very rare (and generally reflects extremely vulnerable buildings), and magnitude 4.0 might define the lower bound for natural tectonic earthquakes that have been reported to have caused damage. Even if earthquakes of magnitudes smaller than 4.5 have caused damage to precarious buildings, the evidence suggests that this would only have occurred very close to the epicentre. Among the case histories that are described in this section, the largest magnitudes in three of the four cases were appreciably smaller than 4; for the one case where the largest magnitude was above 4, the closest buildings were located some 20 km away. Therefore, at face value, these case histories would seem to imply, individually and collectively, that induced earthquakes are more destructive than their tectonic counterparts of the same magnitude—even though comparisons of the ground shaking levels that they produce do not suggest that this is the case (see Sect. 9.3). In most of the cases, it should be recognised, the decision to suspend the operations was also linked not only to the earthquakes that had occurred but also to larger events that it was claimed could occur if the activity continued (emphasising why the estimation of Mmax for induced seismicity can be a critical issue, as discussed in Sect. 9.2).

These are all cases in which I have been directly involved in one way or another, and about which I am at liberty to divulge information, so I hope that I am able to provide some insights beyond what has already been published in the literature.

The final point that I would like to emphasise is that the common theme linking all four cases is energy supply. The first case (Basel) is a geothermal project, an energy source that most would agree is ‘green’. The other three cases are all related to natural gas supply, covering conventional (Groningen) and non-conventional (Lancashire) reservoirs, and storage (Castor). The fact that these three case histories concern a fossil fuel will perhaps incline some readers to view the suspension of the operations as a positive move from an environmental perspective, which I will try to address is Sect. 13. At this point, before discussing these cases, I would like to provide a little context. At the time of writing (late autumn/early winter 2021), gas prices globally have risen dramatically, leading to the collapse of energy supply firms and closure of factories in the UK, highlighting western European dependence on Russian gas. Readers will likely recall the dispute over gas prices between Russia and Ukraine that led to Russia cutting off gas supplies to Ukraine on 1st January 2009, during the middle of winter. On 7 January, the impact was extended when Russian gas supplies stopped flowing through Ukraine for 13 days, cutting off all supplies to several countries in southeastern Europe. In autumn 2021, Russia has threatened to cut gas supply to Moldova, new tensions have arisen regarding approval of the new Nord Stream 2 pipeline to convey gas from Russia into Germany, bypassing both Ukraine and Poland, and Russia has amassed armed forces on the border with Ukraine. How all this will play out remains to be seen, but the stakes are clearly very high when it comes to security of gas supply, and this is the backdrop against which three European countries have taken decisions, in response to these induced earthquakes, that directly impact their own supplies of natural gas.

### Deep Heat Mining, Basel

**Fig. 118:**
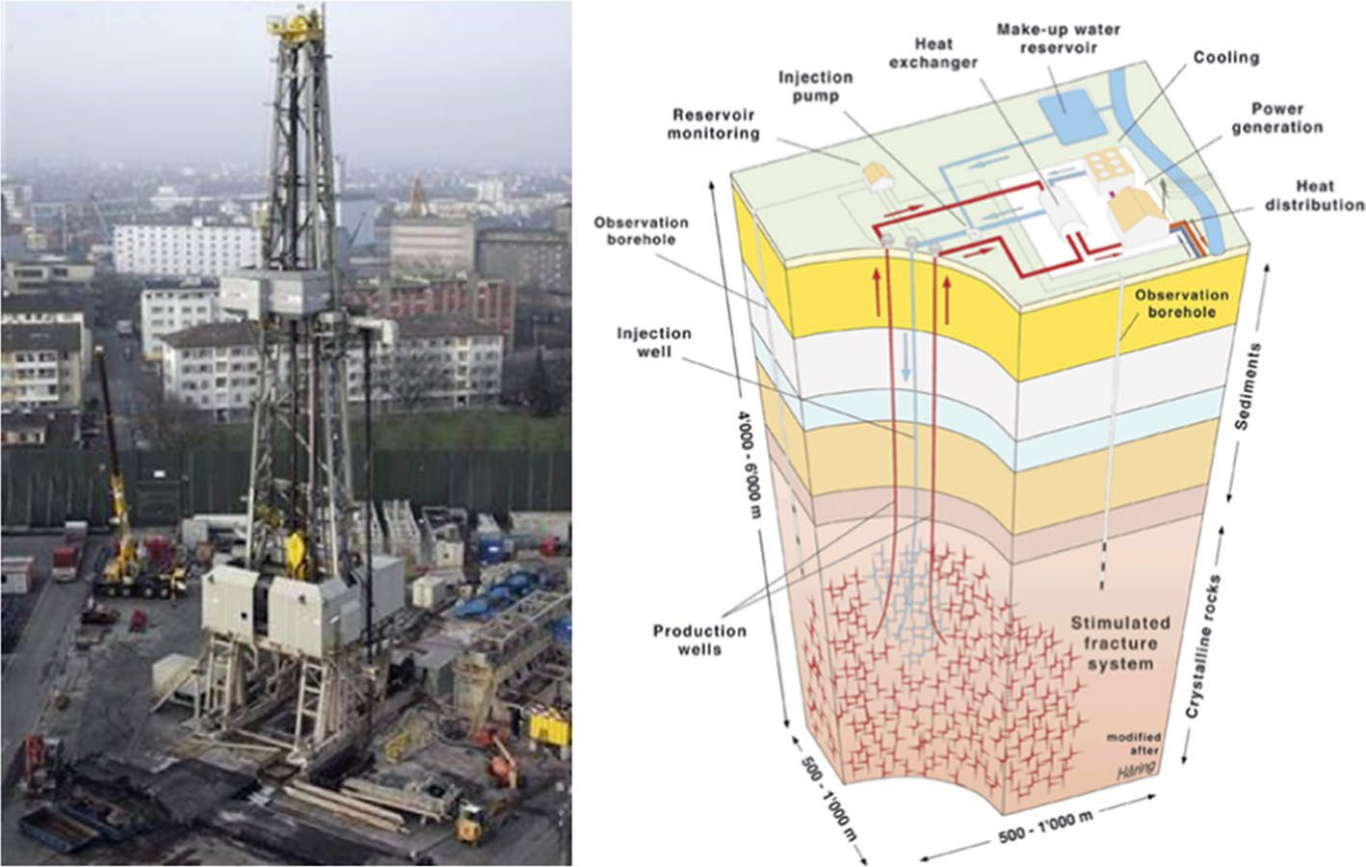
Left: The Deep Heat Mining project in Basel; right: schematic cross-section of the project design (Courtesy of Geothermal Explorers)

The Deep Heat Mining (DHM) project represented a $60 M investment to provide renewable electricity to 10,000 homes in the Swiss city of Basel, located in the northwest of the country very close to the triple junction of borders with France and Germany. In order to be economically viable, the project also needed to provide district heating to 2,700 homes, which required the project to be located in a densely populated location (Fig. 118). As for the HFR project in Berlín, El Salvador (see Sect. 10.1), the objective of the project was to use high-pressure injections of water in order to increase the permeability of hot rocks at a depth of between 4 and 5 km (Fig. 118).

**Fig. 119:**
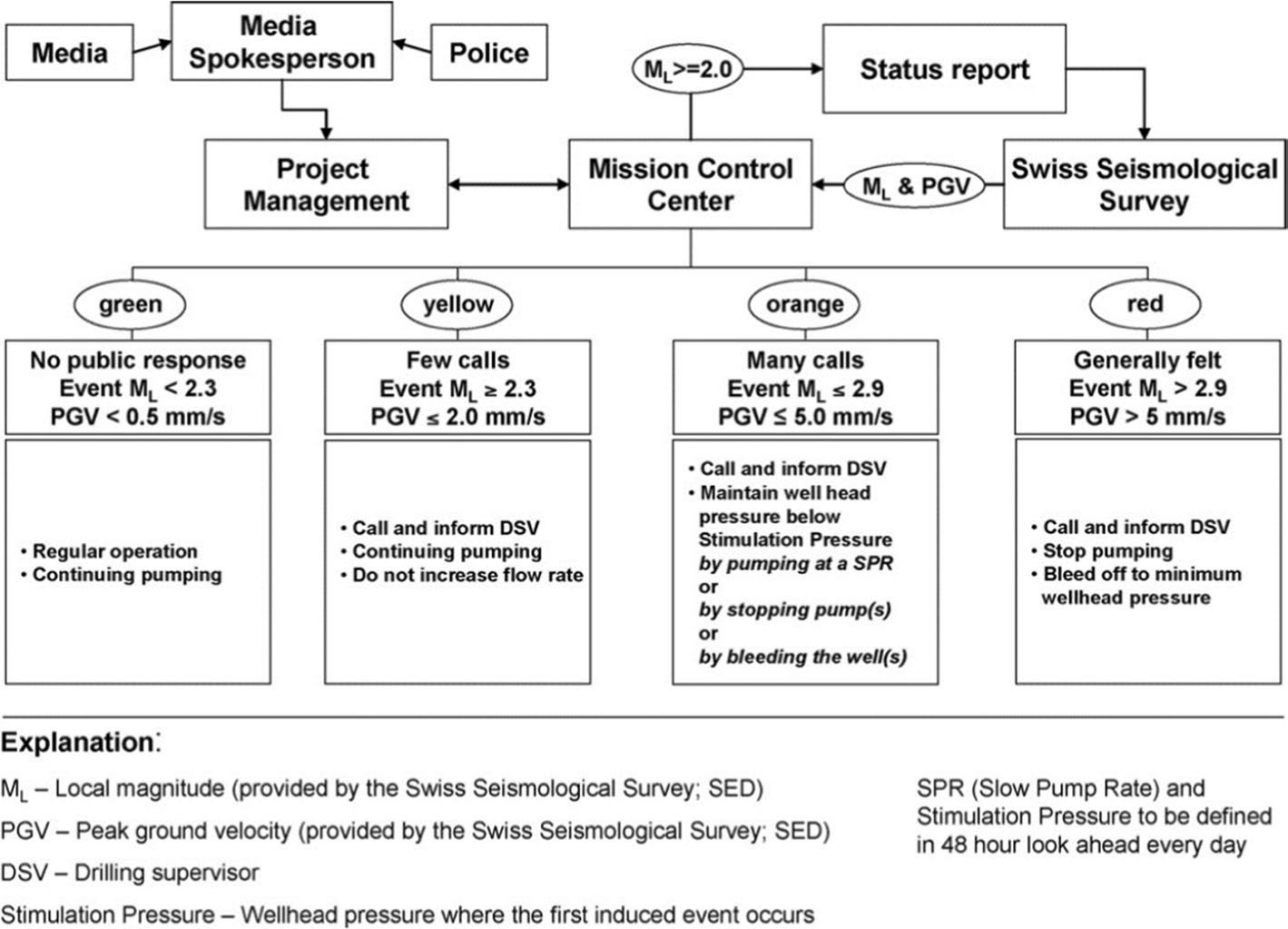
Design of the Traffic Light Scheme for the Basel Deep Heat Mining project (Häring et al. 2008)

The DHM project established a TLS for the control of the induced seismicity, which was adapted from the Berlín HFR traffic light and extended to include four levels and to use both magnitude and PGV as thresholds but with appreciably lower values than had been used in El Salvador (Fig. 119). I participated as a member of the Scientific Board for the project, which was a fairly large and rather loosely organised panel that met to advise Geothermal Explorers, the company undertaking the project under contract to Geopower Basel, which was owned by the City of Basel (the major shareholder) and seven Swiss utilities.

I am not sure to what degree the project achieved buy-in from the inhabitants of the city of Basel, which is perhaps surprising given that the Canton of Basel has a strongly anti-nuclear position (and has tried to close down an NPP in neighbouring France) and there should have been scope to gain popular support for the project. Another factor worth noting is that the time at which the project commenced in December 2006 was perhaps unfortunate since it came a few weeks after the city held commemorative events to mark the 650^th^ anniversary of the earthquake of 1356. While estimates of the magnitude of this historical earthquake range from 6.0 to 7.1 (Meghraoui et al. 2001; Lambert et al. 2005; Fäh et al. 2009), there is irrefutable evidence that a large earthquake struck the city of Basel on 18 October 1356 and caused extensive damage. The DHM project injections began just after the city had conducted commemorative events that will have reminded the inhabitants of Basel that they reside at the location of the largest and most destructive earthquake in the country’s history, which may well have influenced the response to the induced seismicity.

Six days after the high-pressure injections began, early on 8 December, some minor earthquakes were recorded, prompting reductions of the flow rates in response to the yellow traffic light. Later the same day, a magnitude M_L_ 2.6 event occurred, which immediately led to suspension of the injections and bleeding of the injected fluid to reduce the pressure. However, trailing events of M_L_ 2.7 and M_L_ 3.4 (**M** 3.2) occurred after the shut-in and the shaking was felt by many people. Shortly afterwards, the project manager, Markus Häring, was escorted by the police to meet with the crisis management team of the City of Basel.

**Fig. 120:**
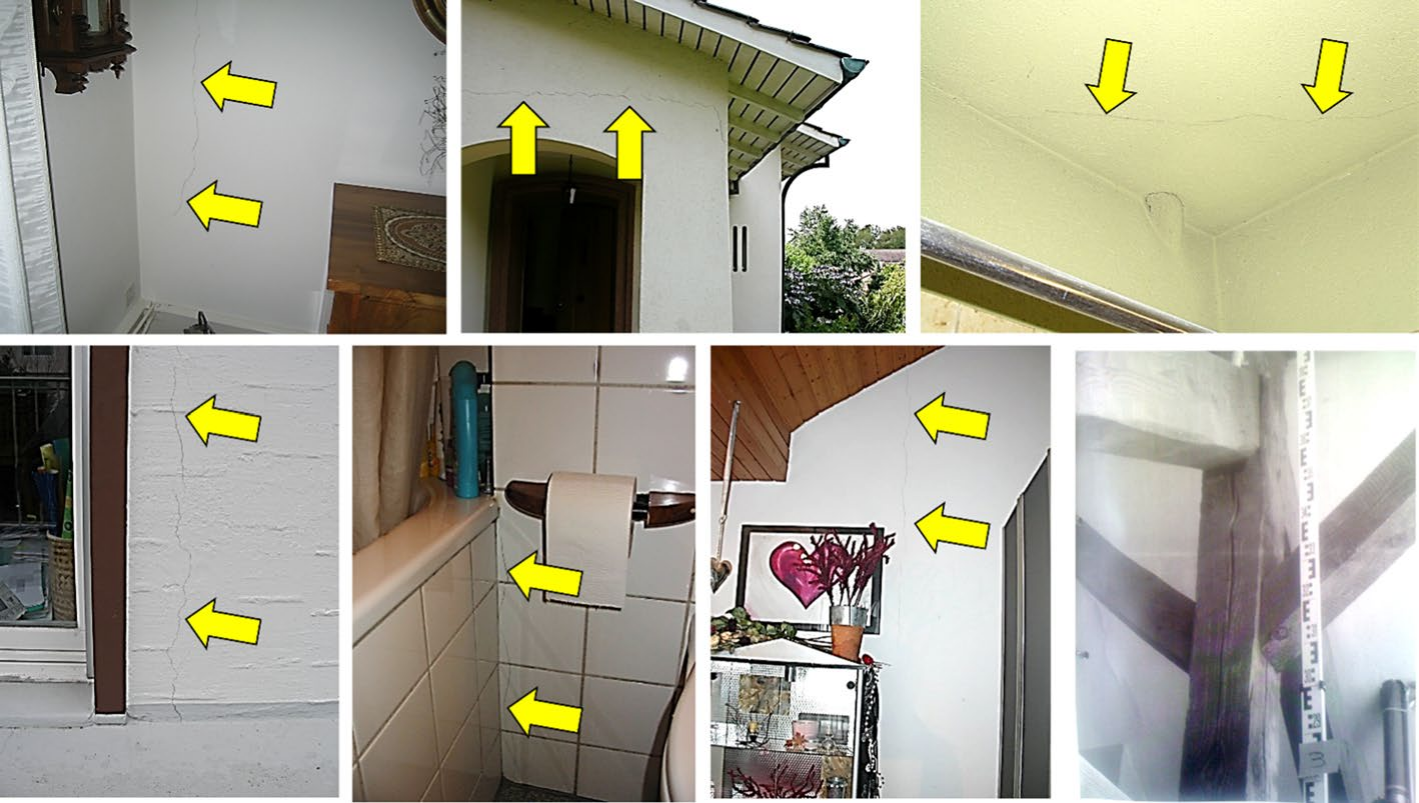
Examples of reported earthquake damage caused by the M_L_ 3.4 Basel earthquake (Courtesy of Geothermal Explorers); in each case, the yellow arrow highlights the crack, except in the bottom right-hand image, where the ‘damage’ is splitting of timber due to drying

The area around the injection well was closely monitored by seismic instruments installed and operated by the Swiss Seismological Service (SED) based at ETH Zürich. The largest recorded PGA from the M_L_ 3.4 event was on the order of 0.1* g* and the largest horizontal PGV a little over 2 cm/s. Deichmann and Giardini (2009) report EMS-98 intensities of IV to V in different parts of the city, and also note that “*very small nonstructural damage was consistently reported for hundreds of buildings, such as hairline cracks to the plaster or damage to the paint at building junctions. Although often difficult to verify, a significant share of the reported instances of damage is presumed to be a direct consequence of the earthquake.*” Examples of the damage attributed to the effects of the earthquake shaking are shown in Fig. 120. Although these are clearly very light levels of damage—and similar to what many of us could find in our own homes after a few years—the claims paid out by insurance companies eventually summed to a total of more than $ 9 million (Giardini 2009). During a full week, the local radio station called for damages to be communicated to a specially installed reporting centre and at the same time Geopower Basel advised insurers not to undertake on-site investigations of claims in order to avoid legal disputes and political controversies. How much physical damage the earthquakes actually caused and what proportion of the total insurance payments corresponded to unverified claims of damage has not been clearly established. Nonetheless, the induced seismicity associated with the Basel DHM project is often referred to as having been damaging, but there is no evidence for any damage that exceeded the kind of hairline cracks shown in Fig. 120.

The project remained suspended while the city of Basel commissioned, by tender, an evaluation of the risk associated with continuing the operations. The risk study was conducted by Baisch et al. (2009), who presented risk results mainly in terms of potential economic losses, considering that continuation of the project could potentially trigger an earthquake of M_L_ 4.5. The risk model was calibrated to reproduce the economic losses generated by the 2006 earthquake as measured by the insurance claims that were settled—as the authors of the study stated: “*Even if it is not proven (and as far as we know, no attempt was done in that sense), that damages were for sure caused by the earthquake, we consider these values as the direct consequences of the 2006 earthquake.*” The estimated potential losses calculated on this basis were very high and led to a decision by the authorities to permanently suspend the project, which was a blow not only for the Basel DHM project but also for enhanced geothermal projects in general.

Three years after the earthquakes, project leader Markus Häring was actually put on trial, the charges being stated as *Vorsätzliches Verursachen eines unterirdischen Bergsturzes* and *Vorsätzliches Verursachen einer unterirdischen Überschwemmung*, which would translate as intentionally causing an underground landslide and intentionally causing an underground flood. While causing landslides and floods are criminal offences under Swiss law, Markus was swiftly acquitted of these nonsensical charges (and the prosecutor who brought the case went into retirement). However, there was never any compensation to the wrongly accused, and it is also not clear why he became the scapegoat for a project effectively owned by the city of the Basel. I find it deeply troubling that somebody could face criminal charges for the consequences of efforts to develop a green energy source, despite putting a system (the TLS) in place to avoid escalation of induced seismicity and implementing the response protocol as specified.

**Fig. 121:**
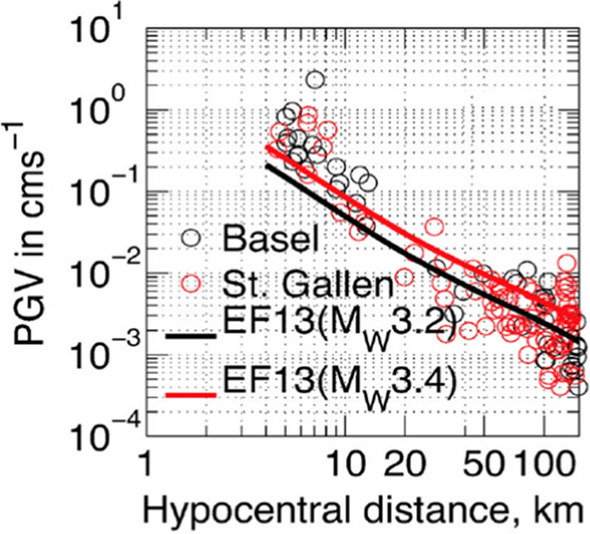
Recorded PGV values from the Basel (black) and St Gallen (red) earthquakes, plotted as a function and hypocentral distance (modified from Edwards et al. (2015); the solid lines are the median predictions from the stochastic GMM of Edwards and Fäh (2013a) for the Swiss foreland, adjusted to a V_S30_ of 620 m/s

The charges brought against Markus Häring are all the more surprising if one considers that it was not clearly established how much damage had actually been caused. Insight into that question was provided a few years later by another Swiss geothermal project, in St Gallen on the eastern side of Switzerland, southwest of Lake Constance. On 30 July 2013 the injections at St Gallen caused an earthquake that was slightly larger than the Basel earthquake (M_L_ 3.5, **M** 3.4; Diehl et al. 2017) and located at a similar focal depth (4.3 km *cf.* 4.7 km for Basel). The ground motions recorded in the two earthquakes were of similar amplitude, as shown in Fig. 121, although there may have been greater site amplification effects in Basel than in St Gallen. The notable fact is that there were no reports of damage due to the St Gallen earthquakes and no claims were made for damage due to this earthquake, which stands in very stark contrast to the enormous damage bill in Basel.

The St. Gallen geothermal project, approved by 80% of the population in a local referendum, was eventually discontinued, but the induced seismicity is reported to have been a minor factor in this decision. Indeed, it is reported that even after the induced earthquake, there was public pressure for the project to continue (Moeck et al. 2015). The main reasons that the project was discontinued were low flow rates, the presence of large volumes of gas (the expansion of which had a cooling effect that reduced temperatures) and financial issues.

### UK shale gas

This case history relates to hydraulic fracturing, or fracking as it is widely referred to, which is a controversial topic regardless of induced seismicity. However, the focus of this paper is exclusively on induced earthquakes; suffice to note here that in the last 15 years, hydraulic fracturing for unconventional hydrocarbon production has expanded enormously on a global scale—and has possibly been a major contributor to delaying ‘peak oil’ (see Sect. 13.1). Hydraulic fracturing is a technology that has been used in the oil and gas industry for several decades, but recent technological advances, including multi-stage horizontal wells, have expanded its application to reservoirs that were previously unexploited, such as shale and tight sandstones.

**Fig. 122:**
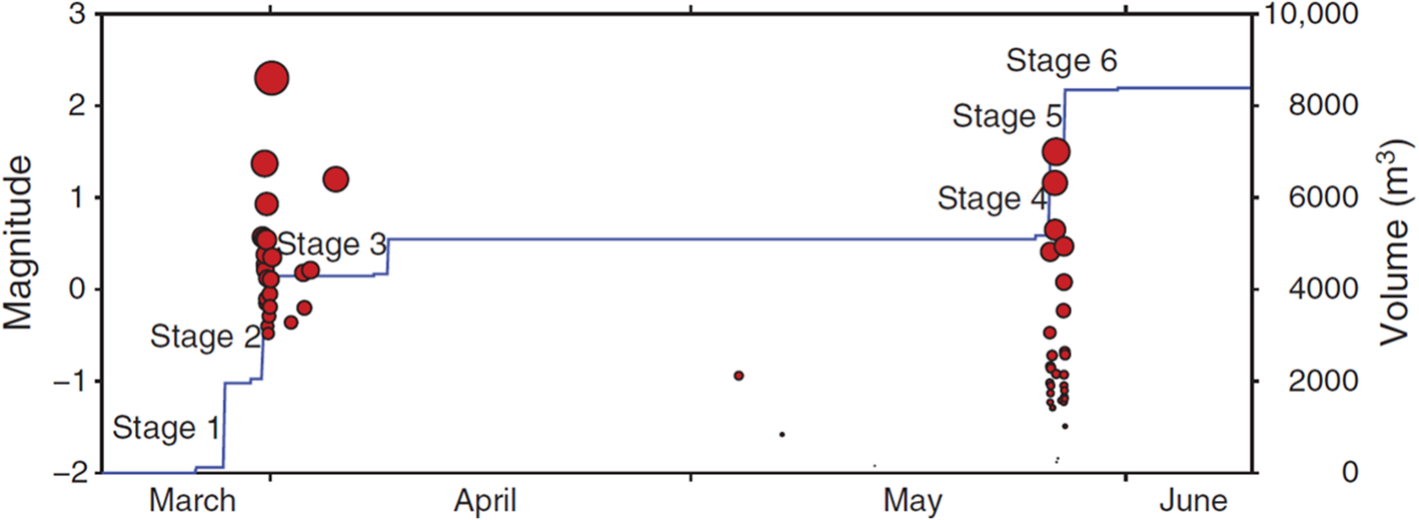
Cumulative injection volume in the Preese Hall well (blue line) and time and magnitude of the induced earthquakes (red dots) (Verdon et al. 2019)

There are potentially large natural gas reserves in shale deposits in the UK that could be produced through hydraulic fracturing (e.g., Selley 2012). In 2011, Cuadrilla Resources began hydraulic fracturing in the Bowland shale in Lancashire at the Preese Hall site. The second stage of hydraulic fracturing was completed on 31 March and a little over 10 h later, on 1^st^ April, an earthquake of M_L_ 2.3 was reported by the British Geological Survey (BGS) located close to the injection well. No seismicity was observed in the following weeks and operations were resumed on 26 May, but the following day, again about 10 h after operations were completed, another earthquake occurred, this one of M_L_ 1.5 (Fig. 122) and better recorded because of the installation of additional seismographs following the first event (Clarke et al. 2014). Both of these earthquakes were reported to have been felt, which is rather surprising in the case of the second event. Following discussion with the UK Department of Energy and Climate Change (DECC), the* de facto* regulator, Cuadrilla suspended the operations and commissioned a specialist geomechanical study.

**Fig. 123:**
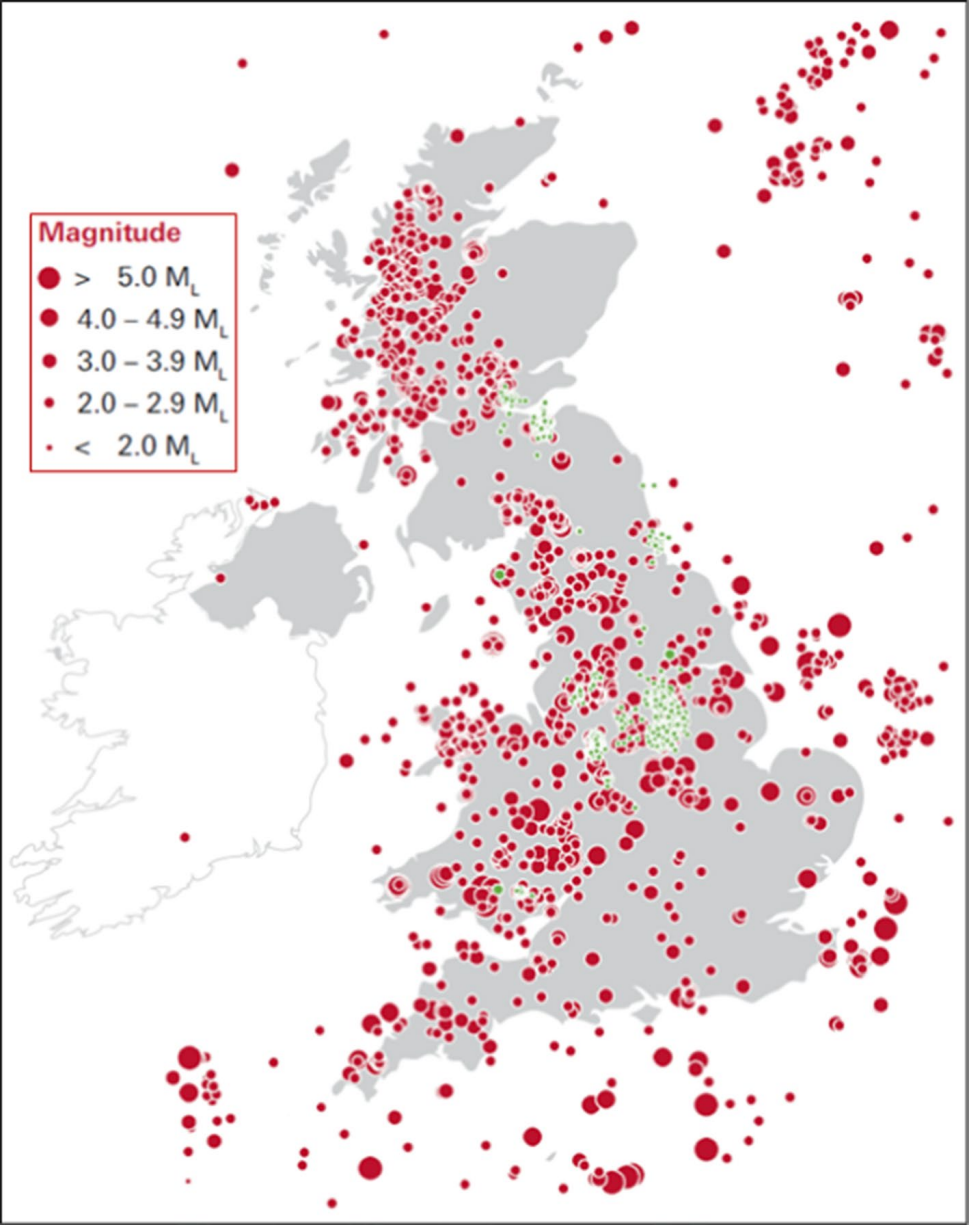
Tectonic (red) and mining-induced (green) earthquakes in the UK from 1382 to 2012 according to the British Geological Survey ( modified from RS and RAEng 2012)

It is worthwhile pointing out that there has never been any real controversy regarding a causal link between the Preese Hall injections and the M_L_ 2.9 earthquake. Applying their new question-based scheme for distinguishing induced from natural earthquakes (see Sect. 8.2), Verdon et al. (2019) obtained an IAR of 75% in favour of an induced earthquake even with the information available in April 2011 (when the ESR was 42%); once all the relevant data became available (increasing the ESR to 82%), the IAR rose to 83%, which implies very high confidence that the event was induced. At the same time, it may be useful for readers who are unfamiliar with the UK to note that while the UK is a region of low seismic activity on a global scale, both natural and anthropogenic earthquakes do occur, the latter having mainly been caused by mining (Fig. 123). The two largest earthquakes unambiguously associated with mining were both of magnitude M_L_ 3.1 (Wilson et al. 2015). In recent decades, mining-induced seismicity has diminished because of the closure of most of the UK coal mines, but to my knowledge when these events did occur, they neither had any serious impact nor generated controversy.

The study commissioned by Cuadrilla concluded that the Preese Hall earthquakes were caused by the injected water entering a small and previously unknown fault, and that if operations continued the maximum magnitude of future events was unlikely to exceed M_L_ 3.0 (de Pater and Baisch 2011). The report also recommended a TLS for control of induced seismicity in future operations, in which it was proposed that green correspond to events smaller than M_L_ 0.0, and that the red-light threshold be set at M_L_ 1.7. For intermediate magnitudes, the yellow-light response proposed by de Pater and Baisch (2011) was just to continue seismic monitoring after each stage for at least two days “*until the seismicity rate falls below one event per day*”, but without any changes to injections during operations already underway. DECC commissioned a separate study to review the report by de Pater and Baisch (2011) and to make recommendations for future operational controls. The report by Green et al. (2012) noted that had the recommended TLS been in place at Preese Hall, no remedial action would have been taken prior to the M_L_ 2.3 event. However, rather than recommend a more effective yellow-light response, the proposal was to lower the red-light threshold from M_L_ 1.7 to M_L_ 0.5. This proposal—which I learned, during an animated debate that took place at a workshop on induced seismicity hosted by the American Association of Petroleum Geologists in London, was claimed by the second author^3^ of the Green et al. (2012), report—was accepted and implemented by DECC. The decision to set the magnitude threshold so low—probably below the limit of event detection by the BGS seismograph network—surprised many because it is clearly excessively conservative, even when accounting for trailing events and magnitude jumps (Sect. 10.1), and probably unworkable as an operational protocol. Indeed, the third author of the Green et al. (2012) report has subsequently been quoted questioning the very low threshold: “*The existing regulations are really quite conservative, they are set at a level of earthquake that is really very unlikely to be felt. So something like 1.5 is a level of earthquake that is not going to be felt widely by people – I think it is something we ought to have a look at*” (Dr Brian Baptie, BGS, quoted on BBC News https://www.bbc.co.uk/news/science-environment-46962472). The joint report on hydraulic fracturing for shale gas issued jointly by the Royal Society and the Royal Academy of Engineering following the Preese Hall events noted the following: “*Given average background noise conditions in mainland UK, a realistic detection limit of BGS’ network is magnitude 1.5 M*_L_.* For regions with more background noise, the detection limit may be closer to magnitude 2–2.5 M*_L_.* Vibrations from a seismic event of magnitude 2.5 M*_L_* are broadly equivalent to the general traffic, industrial and other noise experienced daily.*”

Several years later, the UK government lifted the moratorium on fracking imposed after Preese Hall and Cuadrilla were granted permission to resume hydraulic fracturing operations in Lancashire. At this stage, the operations were regulated by three UK government agencies—the Environment Agency, the Health and Safety Executive, and the Oil and Gas Authority (OGA)—although DECC remained involved in terms of setting policy; induced seismicity came under the auspices of OGA. At that time, I was engaged by OGA to advise on tolerable shaking levels from induced earthquakes, expressed in terms of PGV, which were then adopted as a secondary level of the TLS (the triggers were still based on magnitude but recorded PGV levels would be a factor in determining the response). I argued energetically, as had others, for an increase of the red-light threshold magnitude, but while there seemed to be a general understanding that such a change would be appropriate, there was not the political will in government to be seen to be relaxing the rules. In the UK there would appear to be considerable opposition to fracking and there are several very active and very vocal groups that have campaigned against the application of this technology, and the media by and large portray hydraulic fracturing in a very unfavourable light.

**Fig. 124:**
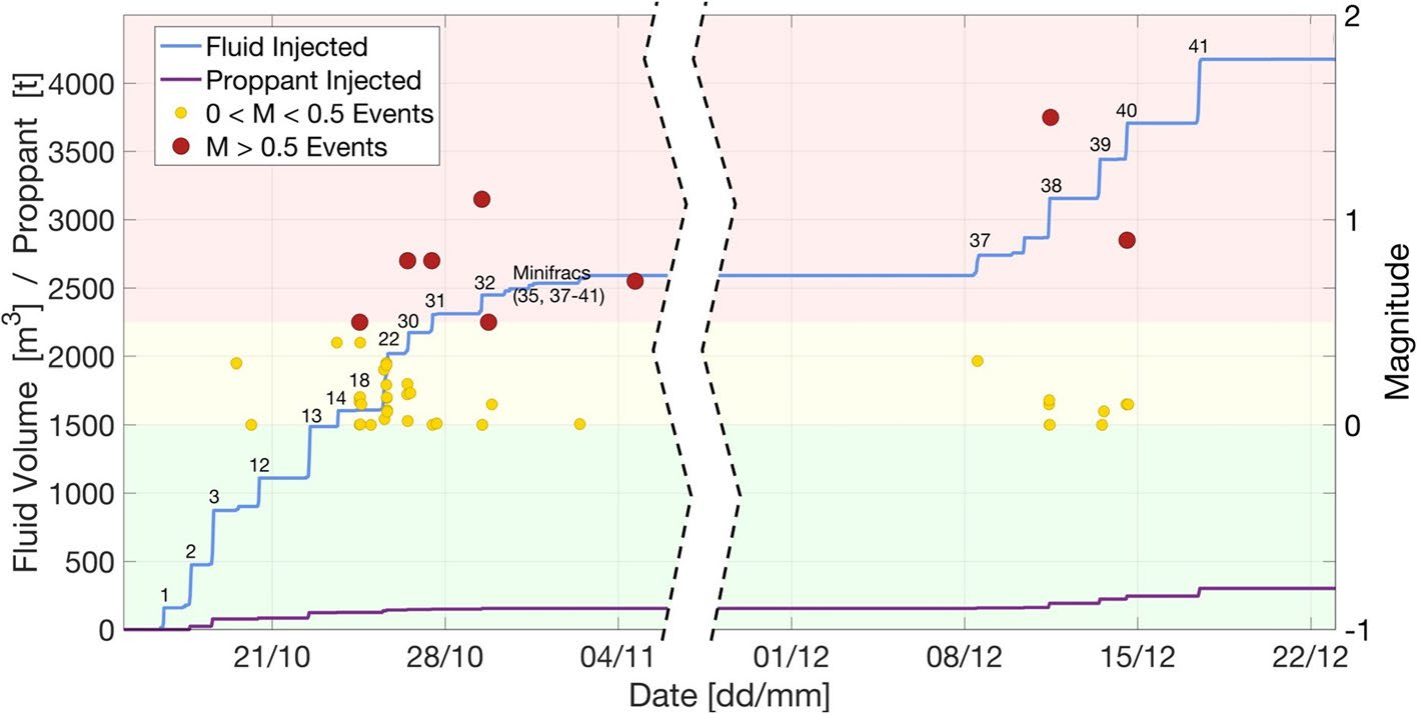
Injected fluid volume (blue line) and weight of proppant (purple line) at the PNR-1z well, showing the induced events that corresponded to yellow or red lights on the TLS (Clarke et al. 2019)

The new Cuadrilla operations were undertaken at Preston New Road (PNR) and injections in the PNR-1z well began in October 2018. As can be seen in Fig. 124, several red-light earthquakes occurred in the first two weeks, causing the operations to be interrupted (for at least 18 h while the situation was reviewed with OGA) on numerous occasions. The interruptions resulted in frequent news reports of the hydraulic fracturing operations being suspended because of earthquakes, even though the events were too small to be felt (the largest was M_L_ 1.1). When injections resumed in December 2018, two more red-light events occurred, the larger with M_L_ 1.5. Apart from the M_L_ 0.5 threshold making it practically impossible to advance the operations, it also resulted in public perception that something of concern was happening at PNR even though these were events of size that occur many hundreds of times across the UK every year. If the objective of the extremely conservative TLS was to make the public feel safe, it seems to have had the opposite effect.

**Fig. 125:**
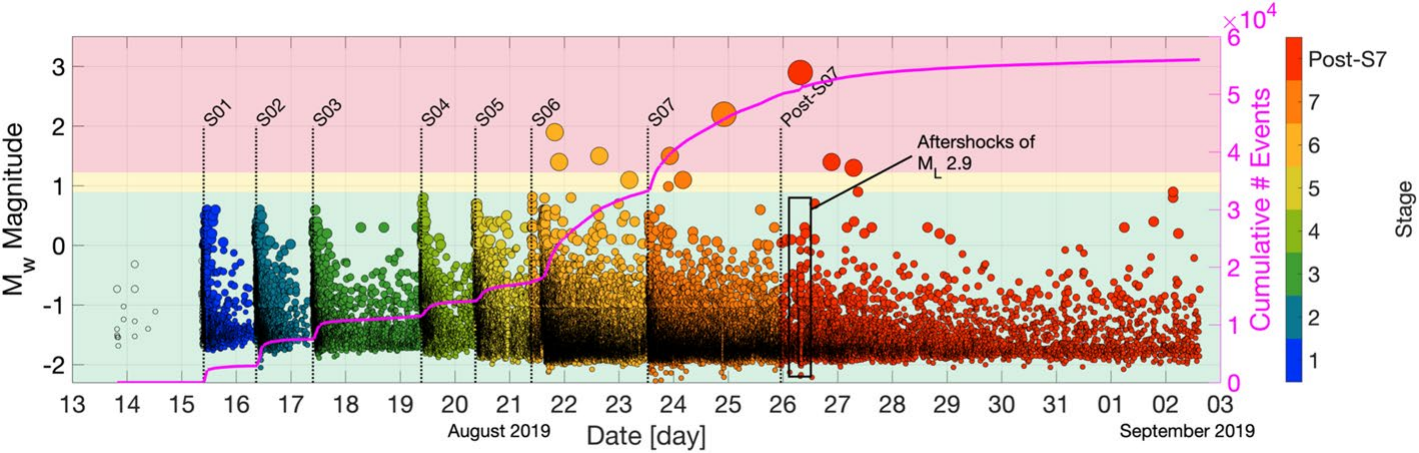
Timeline of hydraulic fracturing stages and induced seismicity for the PNR-2 well (Kettlety et al. 2021); the magnitude thresholds of the TLS have been transformed to moment magnitude using an empirical relationship derived from the data previously acquired at PNR

After the PNR-1z operations closed, the OGA commissioned a series of independent scientific studies of the induced earthquakes and the potential future patterns and impact of induced seismicity. The following year, Cuadrilla began injections in a second well, PNR-2. Once again, as the project advanced, a number of events in the red-light occurred, particular after stage 6 (S06; Fig. 125). The largest event actually occurred about 60 h after stage 7 of the frack had been completed and reached magnitude M_L_ 2.9 (Karamzadeh et al. 2021).

The M_L_ 2.9 occurred on 26 August 2019 and led to a new government moratorium on hydraulic fracturing pending the outcome of new studies. There were 2,266 responses submitted to the BGS ‘*Did You Feel It?*’ online questionnaire for macroseismic observations, on the basis of which a maximum intensity for the event was reported as VI on the EMS, which corresponds to the onset of light damage. However, such reports may be treated with a little caution since they do not reflect on-site assessments by suitably qualified professionals but rather self-reporting by people who have felt the shaking, and therefore will naturally tend to be biased towards the higher indicators rather than the modal observation that should be the basis for assigning an intensity. Moreover, there may be multiple reports of damage for the same structure and in view of the heightened emotions surrounding the operations and the technology, some reports may have been exaggerated. For instance, the reports include one instance of a “collapsed wall” and one of a “collapsed house wall” but these were not supported by the actual damage descriptions and no photographic evidence was provided for the collapses. In Sect. 11 I made the point that in the age of the smart phone, absence of evidence may well be evidence of absence. Photographs of damage attributed to the earthquake have been posted online but most of these could easily be related to settlement: https://drillordrop.com/2019/09/26/cuadrilla-sent-office-staff-to-check-property-damage-from-uks-biggest-fracking-earth-tremor/. The largest recorded horizontal PGV, obtained at ~ 1.8 km from the epicentre, was 0.89 cm/s and the largest PGA was 0.077* g*, which are not levels of motion that would be expected to cause any significant damage.

Following the suspension of operations at PNR-2, OGA commissioned updates of the geomechanical, seismological and seismic risk studies undertaken for the PNR-1z events to be updated using the PNR-2 data. All these studies are available online at https://www.ogauthority.co.uk/exploration-production/onshore/onshore-reports-and-data/preston-new-road-well-pnr2-data-studies/. The seismic risk evaluation, summarised in Edwards et al. (2021), estimated the impact of possible future earthquake scenarios of M_L_ 3.0, 3.5, 4.0 and 4.5; the largest magnitude is considered very unlikely but was estimated to potentially cause non-trivial impacts if it did occur. The overall conclusion of the studies, as summarised somewhat ambiguously by OGA, was that significant uncertainties remained regarding the potential for induced seismicity associated with hydraulic fracturing for shale gas in this region although there was also the possibility to provide improved control of the induced seismicity, and the studies could have provided the starting point to formulate better risk mitigation strategies going forward.

On the same day that OGA published the initial reports based on the PNR-1z data on their website, the UK government announced a permanent moratorium on hydraulic fracturing in the UK, pointing to the reports as the justification—effectively making the induced seismicity the reason for permanently shutting down shale gas recovery in the UK (unless and until this decision is reversed). That the announcement came in the run-up to a general election in the UK (in December 2019), where the most hotly contested parliamentary seats were in the north of England and knowing that there is a great deal of public opposition to the technology, could raise questions about the motivation behind the announcement. Two years later, gas bills in the UK have risen very sharply and many gas-distribution firms have closed down; as noted in the introduction to this section, there is also considerable uncertainty regarding future gas supplies from overseas—while potentially very significant UK gas reserves remain untouched.

A question that may be interesting to ask here is whether the same story would have unfolded had a more rationally designed TLS been deployed at Preston New Road, perhaps with a red light set at M_L_ 2.5? The UK shale gas story may have been even more different had such a traffic light system been in place at Preese Hall in 2011.

### Castor gas storage project

In many parts of the world, including much of Europe, natural gas is an important part of the current energy mix, both for direct consumption and for electricity generation. Gas storage is considered an important component of secure gas supply, the primary motivation being the ability to balance supply and demand, creating additional capacity for periods of extreme cold, for example. Gas storage can also be important for ensuring pressure maintenance in the distribution system and also to provide insurance against unforeseen accidents. During the huge gas price rises of 2021, gas storage capacity has come into sharp focus in many European countries, including the UK, where the Rough facility, located off the Yorkshire coast, which used to account for 70% of the national storage capacity, was closed in 2017. Other European countries, notably the Netherlands and Germany, have far greater storage capacity.

Gas storage is an important issue in Spain since it is country with limited natural gas reserves and therefore relies heavily on imports, which arrive in the form of liquefied natural gas (LNG) by ship or through pipelines from gas-producing nations in North Africa. Security and continuity of supply in Spain consequently depends on gas storage capacity to a greater degree than in many other European countries. The gas grid in Spain is operated by ENAGAS (originally *Empresa Nacional de Gas*), established by the Spanish government in 1972 to develop and operate the distribution grid; ENAGAS was privatised in 1994, the state now holding only a 5% share. ENAGAS operates two onshore subterranean gas storage facilities at Serrablo and Yela, plus the Gaviota facility offshore from northwest Spain. A storage facility at Marismas in southern Spain has, until recently, been operated in conjunction with two small gas fields by the company Gas Natural; plans to expand the storage capacity at this facility were thwarted by strong public and political opposition.

Against this backdrop, the Castor project was intended to add significant additional gas storage capacity. The Amposta oilfield, located about 20 km offshore from the Spanish mainland in the Gulf of Valencia (at a latitude just north of the Balearic Islands), was discovered in 1970 by Shell and entered production in 1972 until the reserves were largely depleted. The company ESCAL conceived a plan to use the space created by the oil extraction to develop a new gas storage facility, which would have had a capacity of about 1.3 Bcm (billion cubic metres), with an output capacity equivalent to about one-quarter of daily consumption in Spain.

**Fig. 126:**
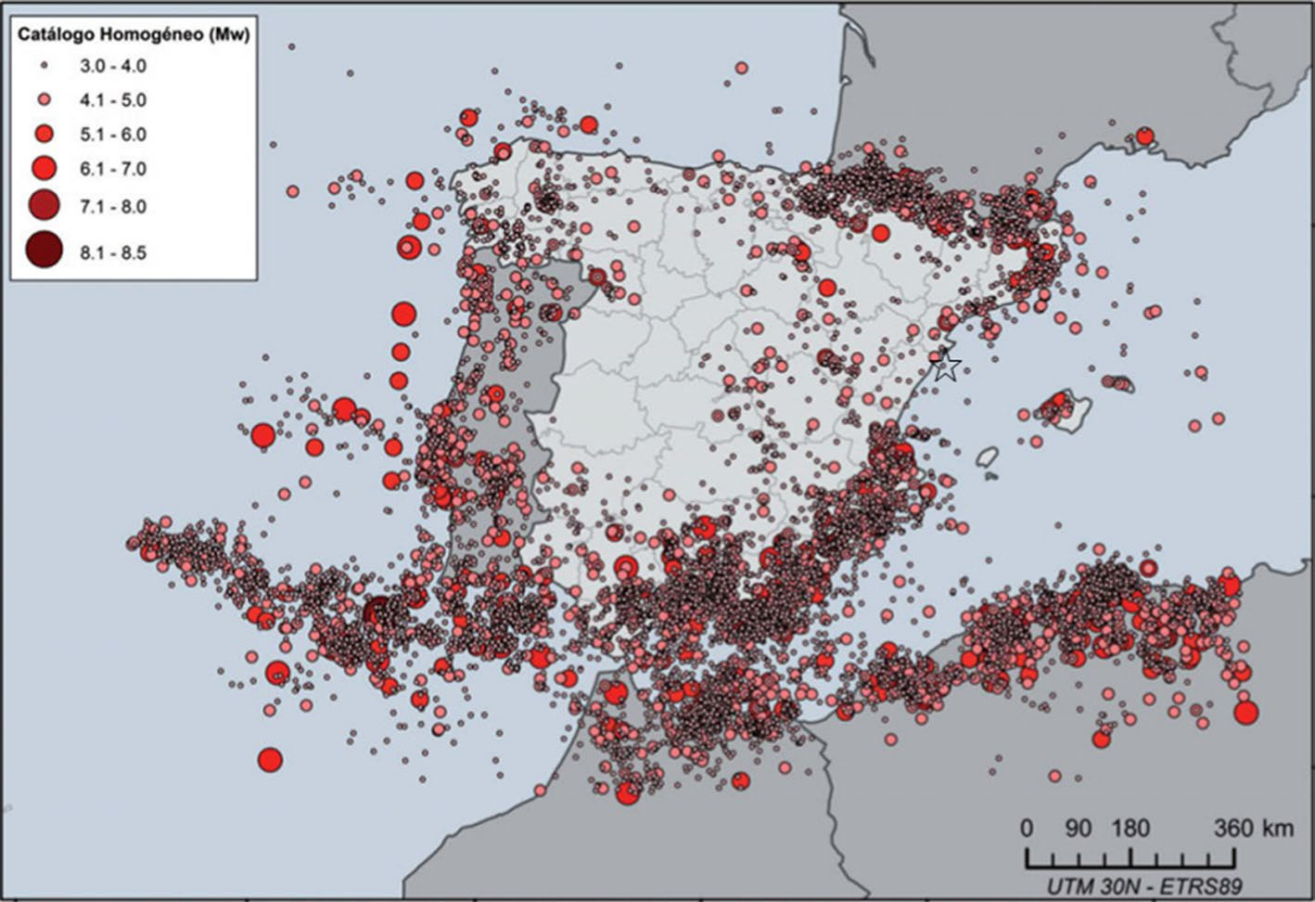
Catalogue of natural earthquakes used in the national seismic hazard mapping of Spain (modified from IGN 2013a); the black star indicates the location of the Castor gas storage project

The Castor gas storage facility is located in a region of relatively low natural seismicity (Fig. 126) and in one of the lowest seismic hazard regions of Spain: the 475-year PGA for this location on the official hazard map for Spain produced by IGN (*Instituto Geográfico Nacional*) is 0.05* g* (IGN 2013a).

**Fig. 127:**
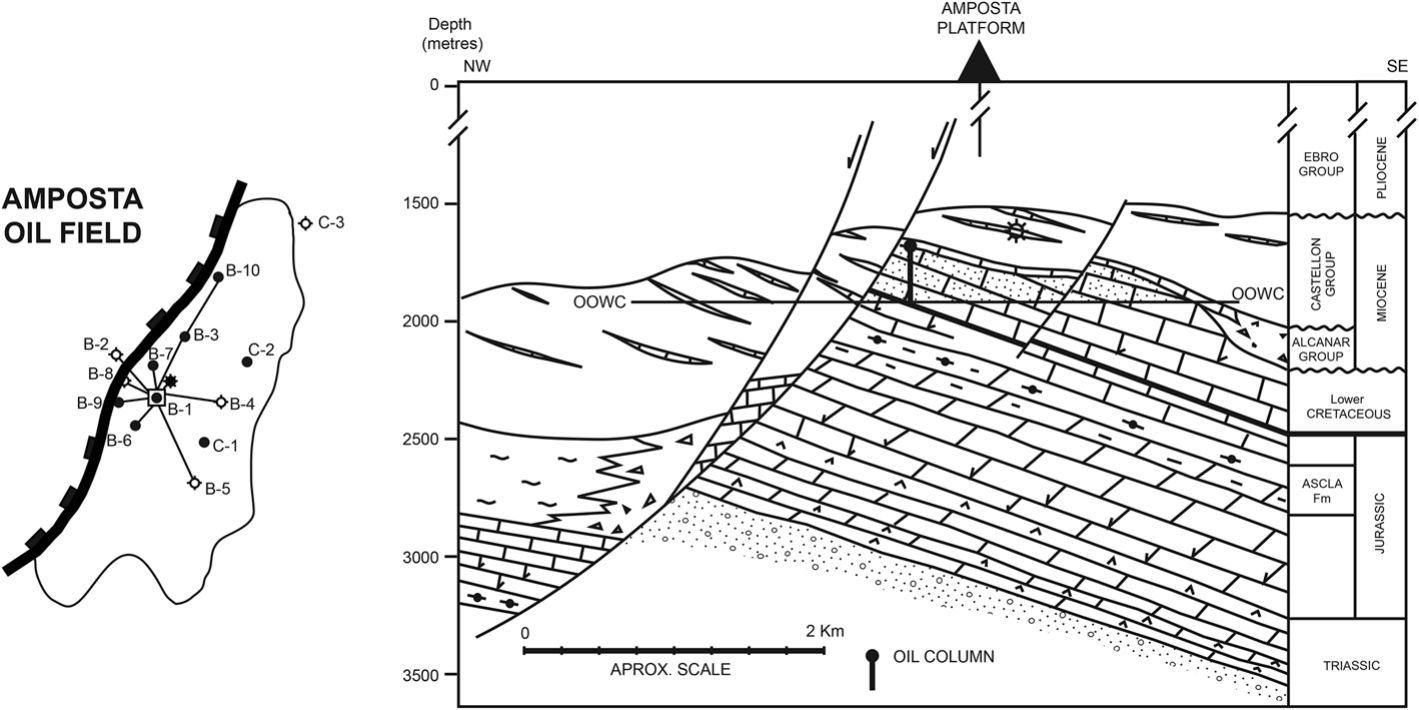
Left: Well locations in the Amposta oil field, which is bounded to the west by the WNW-dipping Amposta fault; right: cross-section showing the location of the oil column (Playà et al. 2010)

**Fig. 128:**
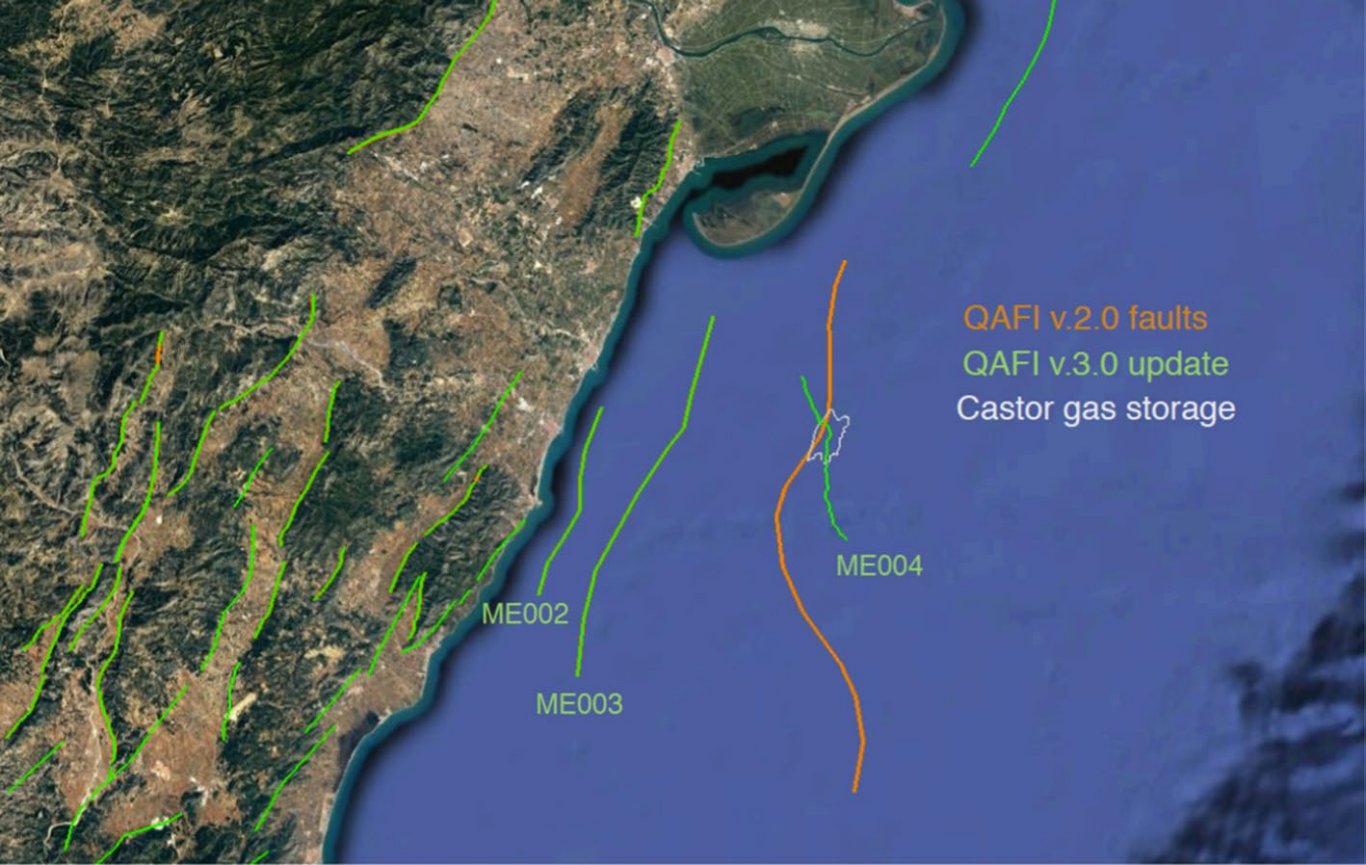
Location of faults in the QAFI database; MEE04 is the Amposta fault (Courtesy of Rodrigo del Potro)

The oil reservoir that became the gas storage facility was located within a rotated block of a horst structure, bounded on the west by the Amposta fault (Fig. 127). The dimensions, geometry and seismogenic capacity of the Amposta fault became critical questions in the Castor story. The *Instituto Geológico y Minero de España* (IGME), the Spanish geological survey, maintains a database of active Quaternary faults, QAFI (Quaternary Active Faults Database of Iberia, http://info.igme.es/qafi/), the compilation of which is explained by García-Mayordomo et al. (2012). The QAFI database is compiled from existing information that is incorporated at face value, thus facing the same tension between breadth and depth discussed in the context of the database of small-magnitude earthquakes reported to have caused damage (Sect. 11.2). The Amposta fault appeared in QAFI v. 2.0 with a total length of 51 km, a dip of 60° and a depth of 15 km, characteristics obtained from the PhD thesis of Roca (1992), which inferred the presence of the fault from a single seismic profile. In one map in that thesis, which was a study of the entire Valencian Trough, the fault identified from the seismic profile was erroneously linked with other faults (with inconsistent dips), resulting in the appearance of a structure 51 km in length. This map was subsequently used by Perea (2006) who inferred the seismogenic potential of the Amposta fault from this exaggerated length and a slip rate inferred from the same seismic profile that had been used by Roca (1992). IGME estimated a maximum magnitude of **M** 7.1, assuming that the entire fault would rupture in a single earthquake. Extensive geophysical investigations were carried out as part of the Castor project, including the interpretation of a large number of seismic lines in the area. The conclusion of these studies was the Amposta fault was a much smaller structure than indicated in QAFI v.2.0 and this new information led to an update of the fault characteristics in QAFI v.3.0 (García-Mayordomo et al. 2017), as indicated in Fig. 128.

Even with the updated length of the Amposta fault, the QAFI database still indicated an active fault with appreciable seismogenic potential, including a maximum magnitude of **M** 6.6, which would require rupture of the fault along its entire length (despite evidence for segmentation) and its entire width. As shown in the cross-section in Fig. 127, the fault is actually a listric structure, becoming horizontal at a depth of a little more than 3 km. Such a structure is unlikely to generate a large earthquake and indeed it is possible that the Amposta is actually a growth fault, linked to salt tectonics, and not a seismogenic structure at all.

In addition to the geophysical investigations of the geological structures around the gas storage reservoir, ESCAL also commissioned independent geomechanical studies by the IFP (French Petroleum Institute) to calculate the possibility of causing displacement on the Amposta fault as a result of the gas injections; these studies concluded that the pressure increases due to the gas injections would fall well short of the pressure required to induce slip on the fault (which could also threaten the integrity of the gas storage). ESCAL also contracted the Ebro Observatory to install additional seismographs in the region surrounding the gas storage facility and to monitor local seismicity in near-real time. There was not a formal traffic light protocol, but the foundation of any TLS is enhanced seismic monitoring and rapid communication of observations, so in practice there was a system in place—and, as explained below, remedial actions were taken in response to observed seismicity, thus making it a TLS in effect if not in name.

An important point to note here is that there were very few precedents for induced seismicity associated with subterranean gas storage projects that would have been the basis of serious concerns for the Castor project. Induced seismicity has been observed in conjunction with gas storage at Bergemeer, Grijpskerk and Norg in the Netherlands (TNO 2015) and in the Czech Republic (Zedník et al. 2001; Benetatos et al. 2013), but the largest earthquakes in these locations did not exceed magnitude 1.5. Tang et al. (2015) report a series of about 200 earthquakes in 2013–2014 that occurred close to the injection well and gas storage facility at Hutubi in China, the largest event reaching magnitude 3.6. However, Tang et al. (2015) acknowledge that it is not clear whether this event was associated with the gas injections or if it was associated with the previous period of gas production from 1998 to 2013.

Another important point to emphasise is that neither regulatory and state organisations in Spain, including IGME and IGN, nor any of the entities engaged to advise on the development of the Castor project, raised concerns or objections related to the possibility of induced seismicity.

**Fig. 129:**
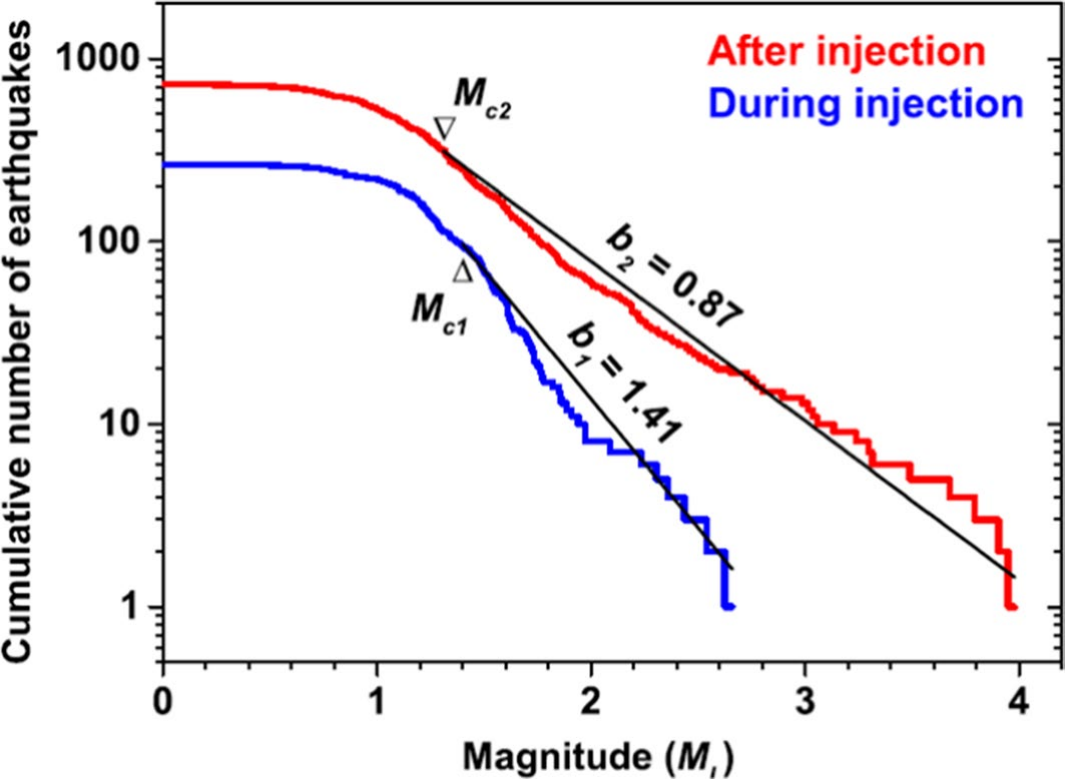
Recurrence relationships for the seismicity that occurred during (blue) and after (red) the gas injections (Cesca et al. 2014)

The first stage of gas injections occurred in June 2013 and was followed by a brief second stage in late August. No seismicity was observed during these operations, leading to an increase of the injection rate during the third phase, which began on 2 September. On 5 September, the first earthquakes occurred, the largest of which reached magnitude 1.5. During the following days, the number of seismic events increased, reaching as many as 20 per day. The largest event was of magnitude 2.7, following which the flow rate was reduced until the end of the third phase on 17 September, with the most intense activity occurring between 29 September and 4 October; the largest event, assigned **M** 4.2, occurred on 1^st^ October. In total, three earthquakes of magnitude greater than 4 occurred. The characteristics of the seismicity that occurred during the injections and after the injections were quite distinct (Fig. 129).

Several studies have since been published in the scientific literature presenting locations of the induced events (e.g., Gaite et al. 2016) and exploring the relationship between the gas injections and the observed seismicity (e.g., Ruiz-Barajas et al 2017). Cesca et al. (2014) note that although it cannot be stated with certainty that the events were not of natural origin rather than being triggered earthquakes, the close temporal and spatial correlations between the operations and the events point strongly to a causal relationship, which seems to be universally accepted. However, the mechanism by which the injected gas led to the earthquakes remains a topic of debate (Cesca et al. 2014; Saló et al. 2017; Villaseñor et al. 2020; Vilarrasa et al. 2021; Cesca et al. 2021), with the more recent studies indicating that the larger earthquakes probably occurred on small faults located below the gas reservoir. The one point on which all of the published studies agree is that the Amposta fault was not the source of the earthquakes.

**Fig. 130:**
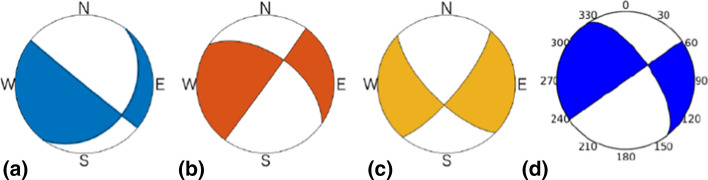
Fault plane solutions for the largest Castor earthquake by **a** Cesca et al. (2014), **b** IGN (2013b), **c** Saló et al. (2017), **d** Juanes et al. (2017); adapted from Juanes et al. (2017)

One study, however, did identify the Amposta fault as the source of the seismicity—and also speculated that if the gas injections were to continue, much larger earthquakes could occur as the result of the activation of this structure. The study by Juanes et al. (2017), authored by academics from MIT and Harvard, was commissioned by ENAGAS, and was seen by many as the ‘official’ study of the Castor earthquakes. The report, which has not been summarised in a peer-reviewed paper, identifies a NW–SE trending fault as the origin of the earthquake, concluding that this is consistent with the Amposta fault. Juanes et al. (2017) perform a moment tensor analysis, the results of which are compared with the fault plane solutions obtained in other studies (Fig. 130). These are lower hemisphere projections, which means that the convex side of the fault plane indicates the direction of dip of the fault plane. Therefore, the favoured fault plane (each diagram indicates two possible, and perpendicular, fault planes) of Juanes et al. (2017) is dipping to the northeast, the opposite direction of the known geometry of the Amposta fault.

The report by Juanes et al. (2017) ended with conclusions regarding the possibility of resuming operations at the Castor facility: (i) the occurrence of events of M ~ 4 was likely to have moved the Amposta fault system closer to failure; (ii) given the fault structures and the history of destabilisation, there was a possibility of earthquakes of larger magnitude, noting that a complete rupture of the Amposta fault system could produce an event of magnitude 6.8; and (iii) defining safe operation injection limits (in terms of pressures, rates and volumes) was difficult. In view of the fact that there is absolutely no evidence for the Amposta fault being the source of the seismicity—indeed, there is evidence to contrary, including the incompatible fault rupture mechanism determined by Juanes et al. (2017)—these conclusions have very little technical basis, but have had very far-reaching consequences for the Castor project.

The earthquakes were followed by vocal protests from communities along the coast and many claims for damages. Recalling the point already made more than once that in this modern era of smart phones, absence of evidence may be interpreted as evidence of absence, the web sites of groups formed to push the case for these claims do not show any images of damage (e.g., http://afectadoscastor.com/); the only ‘evidence’ of damage that has been presented are invoices for building repairs. The absence of any damage is entirely consistent with the magnitude (**M** 4.2) of the event and its location more than 20 km from the closest coastal community. The IGN (IGN 2013b), the official seismological service for Spain, estimated the maximum intensity of shaking along the coast to be III on the EMS-98 scale, the description for which is “*The earthquake is felt indoors by a few. People at rest feel a swaying or light trembling. Hanging objects swing slightly. No damage*.” (Grünthal 1998).

Despite the lack of any material impact of the earthquakes, charges were brought against two of the directors of ESCAL making them responsible not only for what happened but also for what could have happened, the meaning of which is unclear unless one accepts the unfounded speculations of the Juanes et al. (2017) study. The charges would have carried a maximum penalty of 7 years of imprisonment, which would have been a remarkable outcome for two individuals who were part of an imaginative venture to increase energy supply security for Spain and who followed all due diligence in the preparation and design of the project, which went ahead with full regulatory approval. During the writing of this paper, in November 2021, I was one of several expert witnesses who participated in the trial held in Castellón, in which one of the most interesting developments was that the morning after Professor Juanes had appeared as witness (and before the witnesses for ESCAL had taken the stand), a local newspaper ran the headline “*Experts dismiss the Amposta fault as the cause of the Castor earthquakes*” (*El Periódico Mediterráneo*, Tuesday 9 November 2021). I am very pleased to record here that on 1^st^ December the judges issued their verdict, absolving the accused of all charges. While any other outcome from the trial would have been outrageous and while this may seem like a victory for rationality, the fact remains that the Castor gas storage is now permanently closed, with all the injected gas now inaccessible. These consequences have been brought about by a series of small earthquakes that occur from time to time in this region offshore of eastern Spain, and which caused no damage whatsoever. The situation seems to have been created through a combination of the displeasure of some residents of the nearby coastal towns (although it is worth noting that the most distant claims came from locations to north, 90 km from the epicentre) and the self-contradictory and speculative report of Juanes et al. (2017).

### The Groningen gas field

This case history could fill the entire length of this article and my summary and interpretation of the Groningen story is inevitably much longer than the previous three cases. The Groningen story warrants this attention for several reasons, including the fact that it is possibly the single most studied case of induced seismicity, especially in terms of investment in data acquisition and analysis. Groningen could also have been a remarkable demonstration of the rational management of induced seismic risk; sadly, it has become instead a triumph of politics over science. The value in reviewing how this came to pass is not in proportioning blame—although this will be an inevitable by-product of any honest attempt to dissect any of these case histories—but rather to highlight the lessons that can be learnt from this spectacular failure of excellent scientific work to exert any influence on policy decisions with very far-reaching implications.

#### Gas production and induced earthquakes

**Fig. 131:**
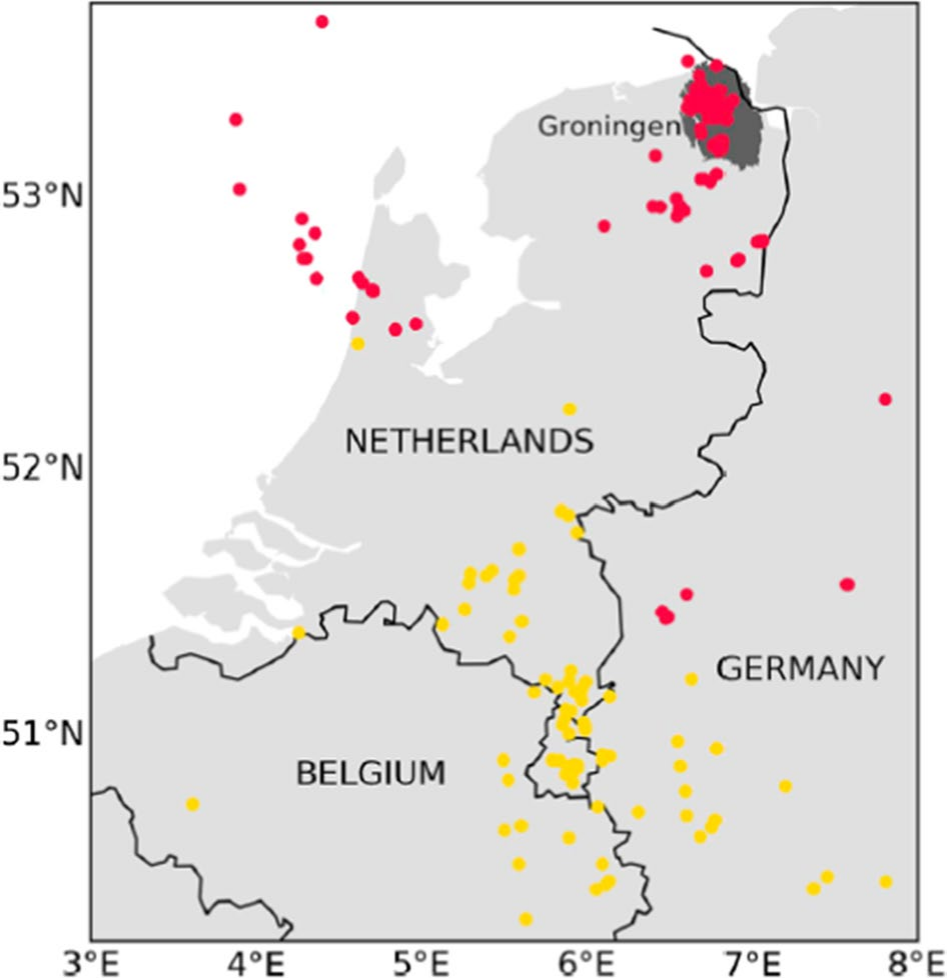
Natural (yellow) and induced (red) earthquakes in and around the Netherlands (Bourne et al. 2014); the grey shaded area in the northeast of the Netherlands is the Groningen gas field

**Fig. 132:**
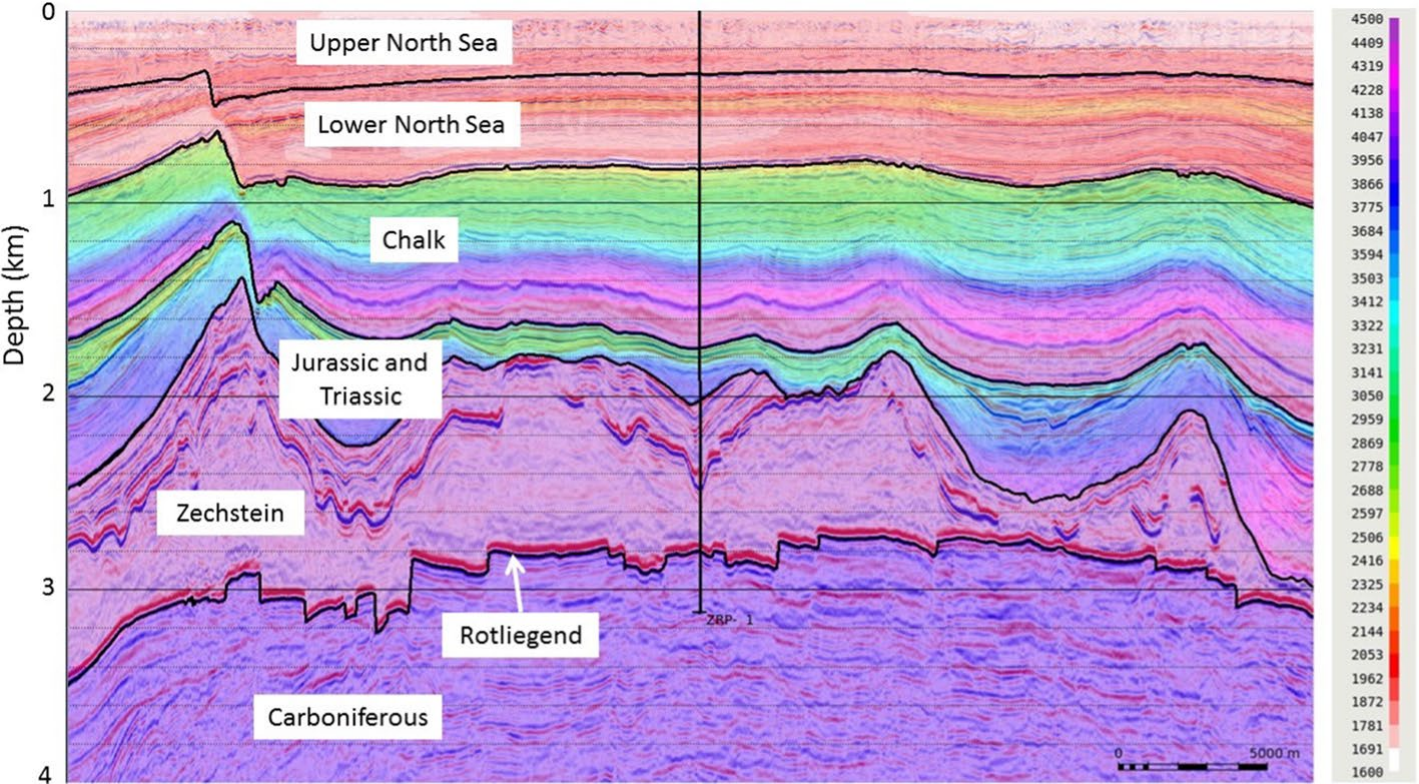
Cross-section through northern part of the Groningen field, intersecting the deep ZRP1 well (vertical black line), indicating the main stratigraphic intervals marked by black lines; colours indicate P-wave velocities in m/s, shown in the legend (van Elk et al. 2019)

**Fig. 133:**
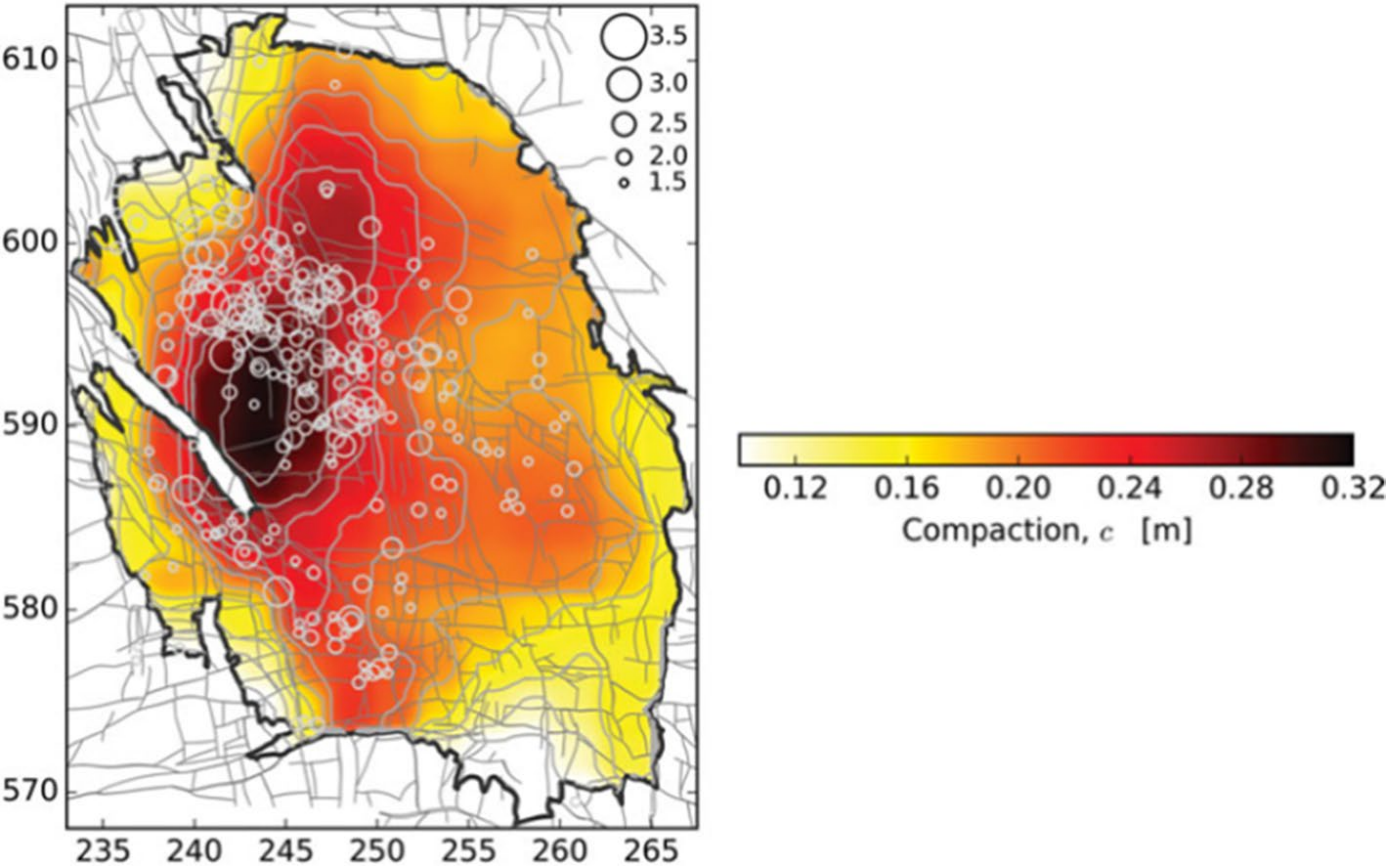
Map of the Groningen field showing reservoir compaction; grey lines are faults and circles are earthquake epicentres (Bourne and Oates 2017)

The Groningen gas field is located in the northeast of the Netherlands, a region apparently devoid of natural earthquakes according to both the instrumental and historical catalogues (Fig. 131). The gas reservoir is contained within the Rotliegend-Slochteren formation, a sandstone unit 150–300 m thickness located about 3 km below the surface (Fig. 132). The gas-bearing sandstone overlies the Carboniferous basement and is overlain by the Zechstein salt, which in turn is overlain by a chalk layer, above which is the North Sea group, consisting primarily of marine clays and sands. There are numerous faults, mostly trending NNE-SSW with some smaller faults trending E-W and N-S, throughout the field, which offset different portions of the gas reservoir by up to several tens of metres, as can be appreciated from the profile shown in Fig. 132; these faults are believed to have formed about 100 million years ago and, prior to the gas production, there was no evidence for geologically recent movement on these structures. Gas is produced from clusters of wells throughout the field, which leads, logically, to a reduction in the reservoir pressure, which in turn results in compaction of the reservoir (Fig. 133) and manifests at the ground surface in the form of regional subsidence, which now has a maximum value of about 35 cm.

**Fig. 134:**
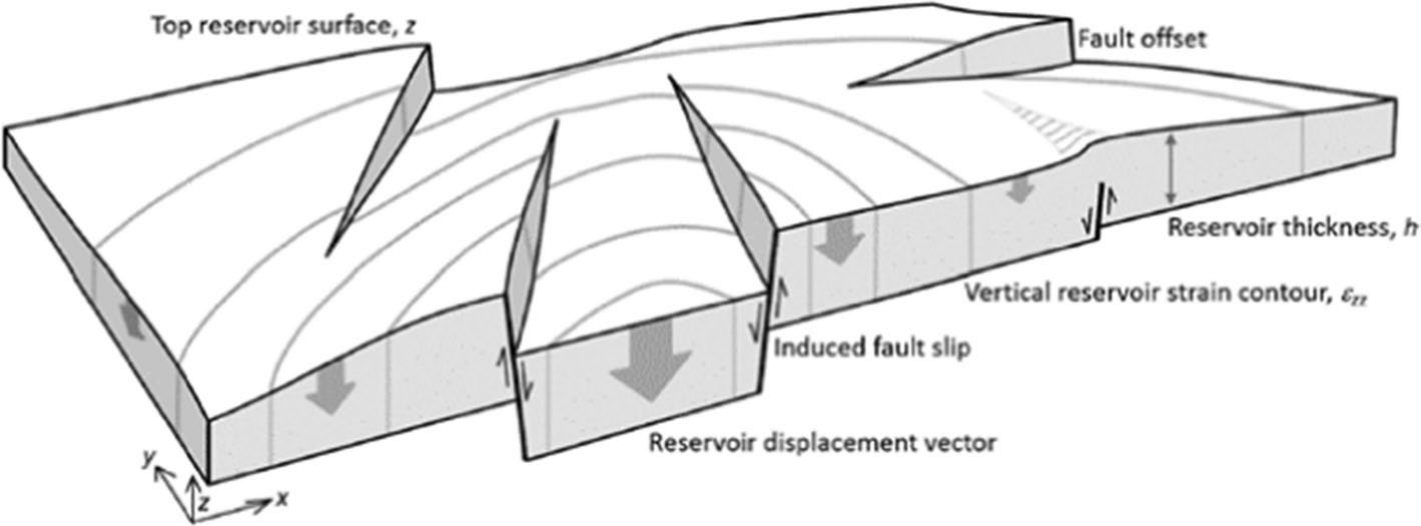
Schematic illustration of how reservoir compaction generates stress of the faults offsetting the Rotliegend and inducing slip on the ancient faults (Bourne et al. 2018)

**Fig. 135:**
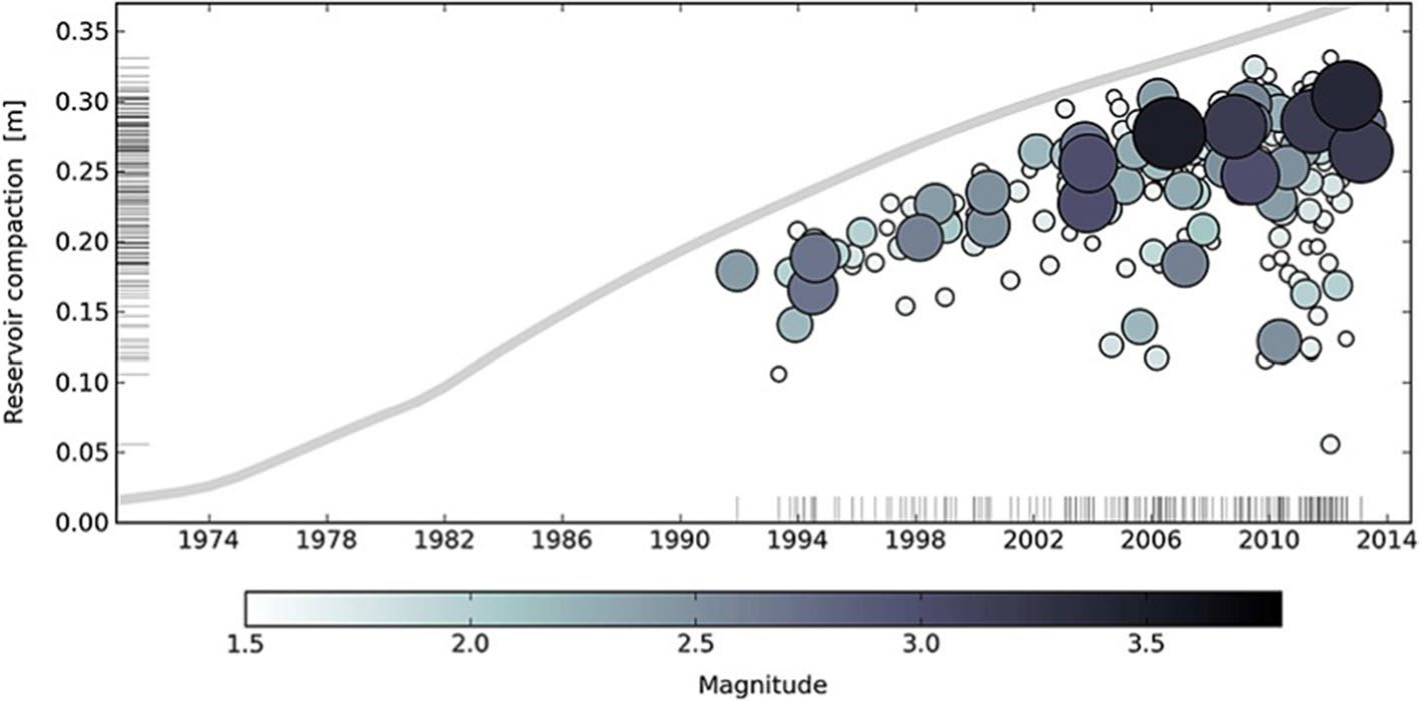
Reservoir compaction and induced seismicity in the Groningen field as a function of data; the light grey curve shows the increase in maximum compaction with time and the circles indicate earthquakes, plotted against the date of their occurrence and at the local compaction level at the time of the earthquake; the size and shading of the circles indicate the magnitude of the earthquake (Bourne et al. 2014)

**Fig. 136:**
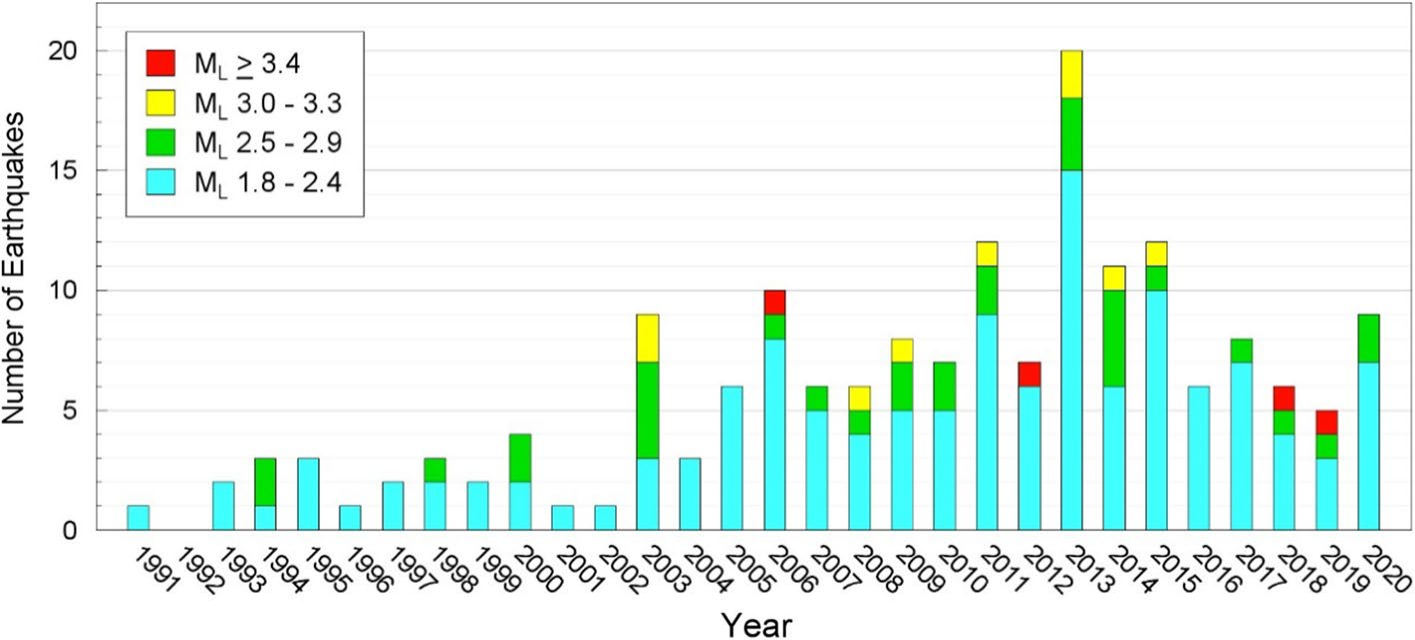
Histogram showing numbers of earthquakes of M_L_ ≥ 1.8 per year up to July 2020

The mechanism by which the Groningen earthquakes are induced (and these earthquakes are genuinely induced as opposed to triggered) is quite distinct from all the cases related to fluid injection that have been discussed. Due to their offsets, the compaction of the reservoir on either side of the fault creates a shearing stress that eventually has led to re-activation of some of the faults through sudden slip (Fig. 134), leading to the small-magnitude earthquakes that have occurred in the field (e.g., Buijze et al. 2017; Bourne et al. 2018). Gas production in the field began in 1963, peaking in 1976 at 88 bcm. The first recorded earthquake, with magnitude M_L_ 2.4, occurred in December 1991; it appears that a critical level of compaction was required for the onset of the seismicity (Fig. 135). In the following three decades, more than 50 earthquakes of the same magnitude or larger have occurred (Fig. 136), and the seismic activity continues to this date, with an event of M_L_ 3.2 occurring on 16 November 2021, which is significant for reasons discussed in Sect. 12.4.6. The four largest earthquakes (of M_L_ ≥ 3.4) have all occurred within or close to the area of maximum reservoir compaction (Fig. 133).

Induced seismicity has occurred in several Dutch gas fields (van Eijs et al. 2006), although prior to the first Groningen earthquake there had only been a few induced earthquakes in Dutch gas fields, the largest being a magnitude M_L_ 2.8 event in the small Eleveld field to the south of Groningen in December 1986. Regrettably, the Groningen field operator, NAM (Nederlandse Aardolie Maatschappij BV, a joint venture of Shell and ExxonMobil), initially claimed that there was no connection between the earthquakes and hydrocarbon production. While this period of misguided and unfounded denial was short lived (by 1993 NAM had acknowledged gas production as the likely cause of the earthquakes), it did lasting damage to public trust.

#### The Huizinge earthquake of August 2012

**Fig. 137:**
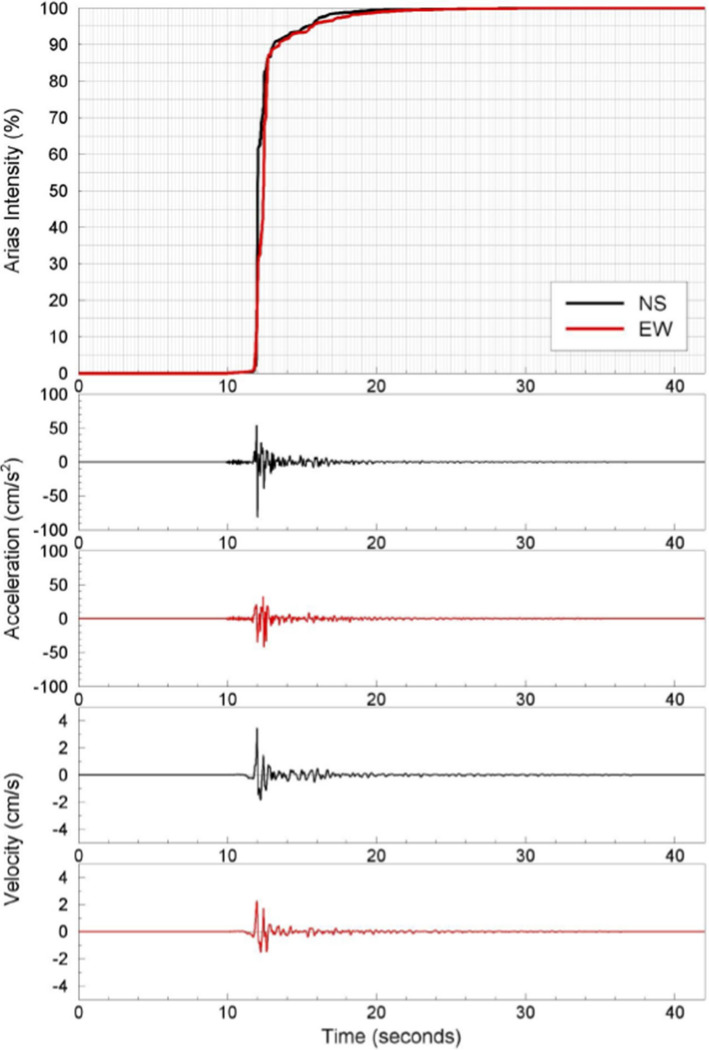
Acceleration and velocity time-series of the horizontal components of the MID1 recording of the Huizinge earthquake; upper plot shows the accumulation of Arias intensity

**Fig. 138:**
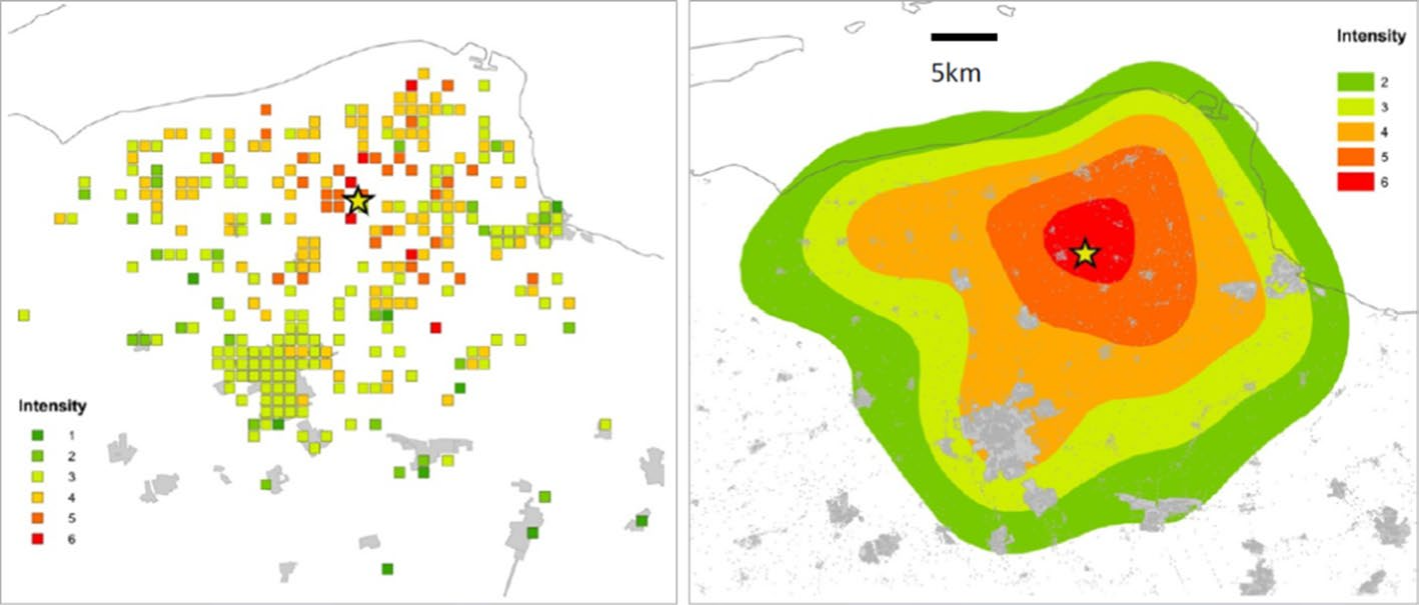
Left: Community Internet-based intensities for the 2012 Huizinge earthquake (epicentre marked by a star); communities are based on the Dutch zip code system and averaged over 1 km^2^ areas and populated areas shown grey; right: KNMI isoseismal map for the Huizinge earthquake (adapted from Dost and Kraaijpoel 2013); note that the scale on the two frames is not the same

The largest earthquake that has occurred in the Groningen field was the Huizinge earthquake of 16 August 2012. The earthquake was assigned a local magnitude of M_L_ 3.6 by KNMI, the Dutch seismological service; the moment magnitude was **M** 3.5. The ground-motion recording network in the Groningen field was rather sparse at that time (the earthquake prompted an upgrade and expansion of the strong-motion network—see Sect. 12.4.4) but a record was obtained at just less than 2 km from the epicentre at the MID1 station: the stronger horizontal component had a PGA of 0.083* g* and a PGV of 3.46 cm/s, and a duration (based on 5–75% accumulation of the Arias intensity) of 0.52 s (Fig. 137). The earthquake was strongly felt in the northern part of the field; from online questionnaires, KNMI determined a maximum EMS intensity of VI—which is consistent with the median predictions from the empirical relationships of Caprio et al. (2015)—over an area of radius of ~ 3–3.5 km (Fig. 138); intensity VI is defined as follows: “*Felt by most indoors and many outdoors. A few persons lose their balance. Many people are frightened and run outdoors. Small objects of ordinary stability may fall and furniture may be shifted. In a few instances dishes and glassware may break. Farm animals (even outdoors) may be frightened. Damage of grade 1 (no structural damage, slight non-structural damage) is sustained by many buildings of vulnerability class A and B; a few of class A and B suffer damage of grade 2 (slight structural damage, moderate non-structural damage); a few of class C suffer damage of grade 1*” (Grünthal 1998). Vulnerability class A refers to rubble or fieldstone masonry and adobe, which are not encountered in the Netherlands. Consequently, the damage would have been expected to be mostly grade 1 (hairline cracks, fall of small pieces of plaster) and possibly a few cases of grade 2 (cracks in many walls, fall of fairly large pieces of plaster).

The Huizinge earthquake is viewed as a turning point in the Groningen story and is often described as the game changer. The obvious explanation for the pivotal impact of the Huizinge event would be that it was larger than any previous earthquake in the Groningen field and caused damage—albeit generally minor—in a relatively large number of houses. In 2003, there had been two earthquakes of M_L_ 3.0 (the Hoeksmeer event of 24 October and the Stedum event of 10 November 2003), which had modest impact: Roos et al. (2009) report that these two events prompted 14 and 82 damage claims, respectively, of which 5 and 43 were accepted and paid. Discussing early induced earthquakes in the Dutch gas fields, van Eijs et al. (2006) had noted that “*The expected damage from these quakes could be described….as ranging from none to, in the worst case, very little light structural damage. However, these quakes have caused significant social anxiety.*” At that time, earthquakes as large as M_L_ 3.4 had occurred in the Roswinkel field at shallower depths of 2.4 km, above the Zechstein salt formation; the M_L_ 3.4 earthquakes in 1997 prompted 235 damage claims, of which 204 were settled (Roos et al. 2009). An event of particular note in this discussion is the Westeremden earthquake of 8 August 2006, which had a magnitude of M_L_ 3.5 and an epicentre less than 2 km to the ENE of the epicentre of the Huizinge event. Roos et al. (2009) report that the Westeremden earthquake led to 410 damage claims, of which 275 were settled.

**Fig. 139:**
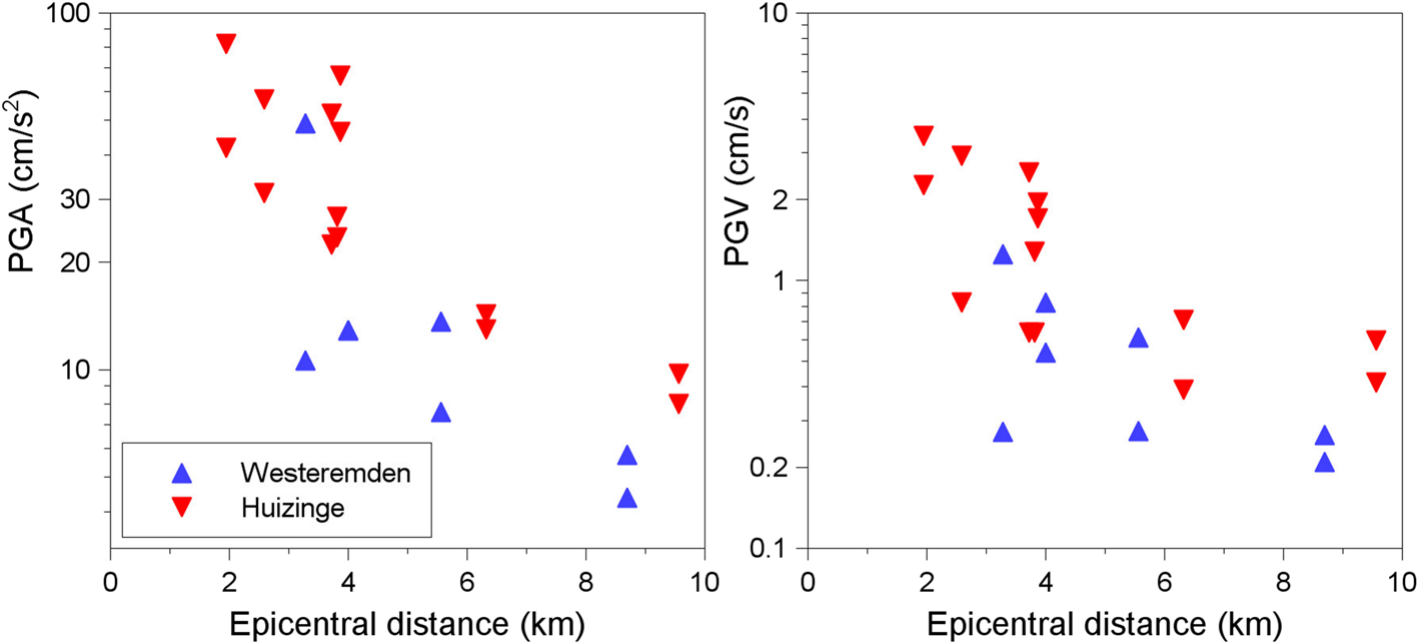
Comparison of recorded horizontal values of PGA (left) and PGV (right) from the 2006 M_L_ 3.5 Westerendem and 2012 M_L_ 3.6 Huizinge earthquakes

Interestingly, recorded motions, especially their PGA values, were generally much stronger in the Roswinkel field (reaching 0.3* g*), which was actually the motivation for developing a bespoke GMM for the Groningen field (see Sect. 12.4.5). Figure 139 compares the recorded horizontal PGA and PGV values from the 2006 and 2012 earthquakes, which does show that the Huizinge motion amplitudes were appreciably higher in general (although still rather low compared to the levels of ground shaking usually associated with structural damage). Using the moment magnitudes calculated for these two earthquakes—**M** 3.38 and **M** 3.52 (Dost et al. 2018, 2019)—the Huizinge earthquake would have released almost 70% more seismic energy than the Westeremden earthquake. The higher energy and higher ground-motion amplitudes of the Huizinge earthquake would certainly explain why it had a greater impact than previous earthquakes in the field, but the extent to which the Huizinge event changed the course of the Groningen story is nonetheless surprising—and perhaps far exceeds the increment of seismic energy and ground-motion amplitudes relative to the previous largest event. In a paper authored by staff members from the regulator of the Groningen field (see Sect. 12.4.7), it was stated that prior to the Huizinge earthquake, models had suggested that the largest earthquake that could occur in the field would be on the order of M_L_ 3.3 to 3.5 and that during such events “*structural damage to buildings and personal risks would not occur…. Based on these outcomes induced seismicity was considered a nuisance, causing damage without posing a safety risk*” (de Waal et al. 2017). The same paper goes on to note that “*The magnitude 3.6 Huizinge event in August (2012) …. led to an unprecedented number of damage claims, involving thousands of homes. It was followed by an independent investigation by the regulator which showed that significantly stronger earthquakes, potentially with magnitudes up to 5.0, could not be excluded and that seismic risk levels in Groningen could be considerable.*” This quote highlights two key issues, one of which is that the Huizinge earthquake raised the prospect of the possibility of even larger events, as highlighted in the study by the regulator, which is discussed in Sect. 12.4.7. The other issue is that the impact was reported not in terms of thousands of damaged homes but rather in terms of thousands of damage claims, an issue explored a little further in Sect. 12.4.3.

A final point worthy of note is that the magnitude of the Huizinge earthquakes was originally reported by KNMI as M_L_ 3.4, slightly smaller than the M_L_ 3.5 of the 2006 Westerendem event. This was only updated to a magnitude of 3.6 in a report issued by KNMI in January 2013 (Dost and Kraaijpoel 2013).

#### Damage and damage claims

**Fig. 140:**
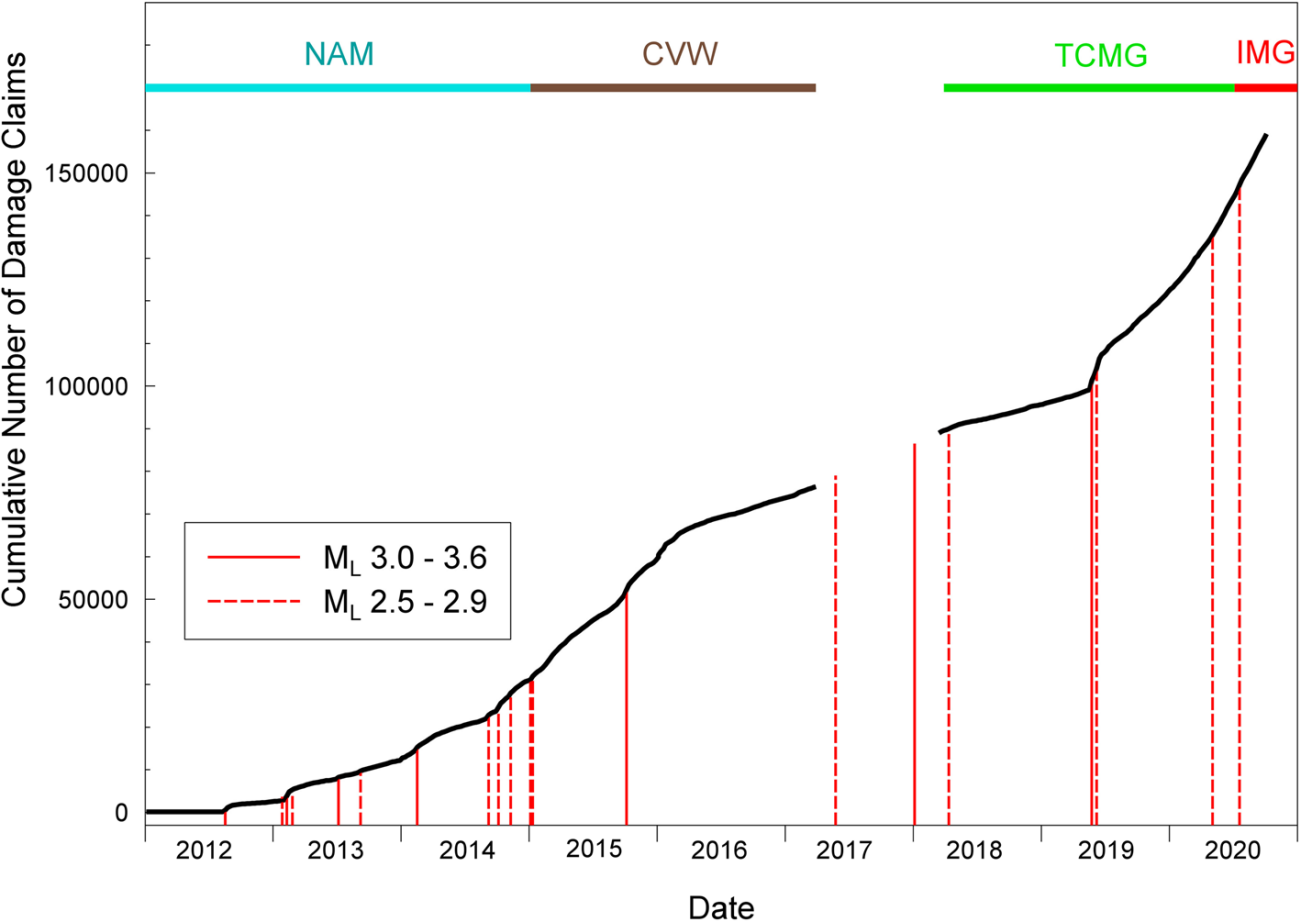
Cumulative number of damage claims paid against time; red lines show the dates of earthquakes of M_L_ ≥ 2.5 and the colour bars at the top indicate the agency responsible for handling the claims

There is no doubt that the Huizinge earthquake caused cosmetic damage in many houses and possibly light structural damage (such as cracks in walls) in a few. Some of the other larger Groningen earthquakes, such as the 2006 M_L_ 3.5 event and other events of M_L_ ≥ 3 that have occurred since, will also have caused similar damage to smaller numbers of houses. However, the claims for damage that have been submitted to the operator of the gas field, NAM, suggest that Huizinge and other earthquakes have had a much greater impact on the built environment over and around the Groningen gas field. Figure 140 illustrates that cumulative number of damage claims that have been submitted since 2012. The figure also shows the dates of earthquakes of at least M_L_ 2.5, as well as indicating the organisation responsible for managing the claims, which for several years has been taken out of the hands of the field operator.

**Fig. 141:**
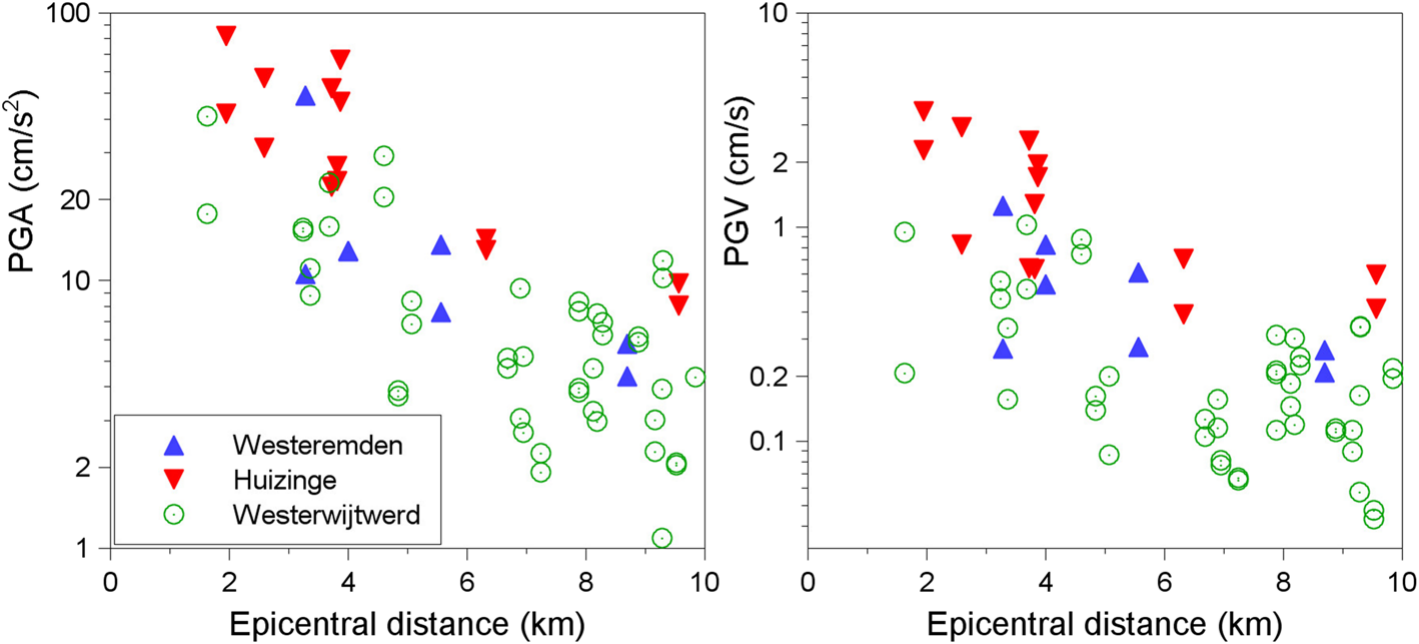
As for Fig. 139 but also showing peak motions from the 2019 M_L_ 3.4 Westerwijtwerd earthquake

A number of important observations can be made regarding Fig. 140, the first of which is that there was a notable but not disproportionate jump in the cumulative number of claims following the Huizinge earthquake, followed by a very gradual increase over the remainder of 2012. The next jump occurred in February 2013, when two M_L_ 2.7 and one M_L_ 3.2 earthquakes took place, resulting in another jump but also an increasing rate of claims submissions thereafter. From then onwards, until May 2019, it is difficult to discern any strong correlation between changes in the slope of the curve and the occurrence of earthquakes. There is a distinct increase in the gradient starting in mid-2014, but this coincided with a government policy for ‘energy efficiency measures’, which obliged NAM to install solar panels in houses for which damage claims were settled; this policy was suspended around the end of 2015. There is a very pronounced increase in the number of claims submitted following the M_L_ 3.4 Westerwijtwerd earthquake of 22 May 2019, starting with a jump much larger than that which followed Huizinge, and then continuing with what appears to be an exponential increase. Figure 141 is the same as Fig. 139 but with the near-source (< 10 km) recordings of the Westerwijtwerd earthquake added in, showing that there was nothing exceptional about the motions from this event (located about 2.3 km SSW of Huizinge)—and certainly no reason for it to cause greater damage than the Huizinge earthquake. The reason for the increased rate of damage claims in recent times is much more likely to be related to be the way that the claims are now handled.

Following the M_L_ 3.4 Zeerijp earthquake of 8 January 2018, the Dutch government introduced legislation that opened up the possibility of submitting claims for compensation of physical damage caused by the induced earthquakes in the Groningen field region to the Temporary Committee on Mining Damage (*Tijdelijke Commissie Mijnbouwschade Groningen*, TCMG) in Groningen. From 19 March 2018, a new damage protocol was thus introduced retroactively for all damage reports, and claims were handled by the TCMG. In the current arrangement, claims are settled by the state-appointed IMG (*Instituut Mijnbouwschade Groningen*), which, like its predecessor TCMG, then invoices NAM for the cost of settled claims. The claims do not have to correspond to recent earthquakes, and it is still possible for claims to be submitted now for damage attributed to the Huizinge earthquake. By the end of 2012 (ignoring all claims prior to Huizinge), the value of the claims paid summed to 37.2 MEuros. These values could be compared with the losses report by EM-DAT (https://public.emdat.be/data) for the 1983 magnitude 5.1 earthquake in Liège, Belgium, and the 1992 magnitude 5.2 Roermond earthquake in the southern Netherlands, which, adjusted to 2020 values, are 130 and 184 million USD respectively; those earthquakes would have released approximately 200 times more energy than the Huizinge earthquake. However, although there has been no earthquake equal in size or larger than the Huizinge event since 2012 (Fig. 136), the total that has now been paid for damage claims exceeds 660 MEuros.

There are many images available of buildings in the Groningen field showing signs of distress. Much of this damage is very likely due to differential settlements; settlement-related damage to buildings is common in many parts of the Netherlands (e.g., Peduto et al. 2017), especially where the near-surface geology includes peats and soft clays, deposits that abound in the Groningen region. In the Groningen region, settlement effects could have been exacerbated by seasonal variations in ground water levels, especially during droughts that have occurred in recent years. Many of the more severe cases of damage reflect patterns that are indicative of differential settlement, but the lighter damage is often very difficult to assign to either shaking or settlement on the basis of its appearance. At the end of 2016, the Dutch government introduced a policy named ‘evidence presumption’, which essentially meant that unless NAM could demonstrate that observed damage could be unambiguously attributed to another cause, it would be assumed to be due to ground shaking resulting from induced earthquakes.

To close this discussion, I just note that the only way that the production-related earthquakes in Groningen could cause damage to buildings is through the inertial loads imposed by ground shaking. The subsidence due to reservoir compactions occurs over such a wide area that the resulting rotation of any individual building would be far too small to be a cause of damage. The Groningen earthquakes have also not caused soil liquefaction (see Sect. 11.3) and the shaking levels have been far too low to cause dynamic deformations of the foundations. Close to the epicentres of the larger earthquakes, there will inevitably be some ambiguity between damage due to shaking and damage due to differential settlement, and indeed interaction between the two (e.g., Bal et al. 2021). With increasing distances from these small earthquakes, it becomes increasingly likely that any observed damage is the result of static settlements rather than earthquake shaking.

#### Data acquisition and analysis

In Sect. 12.4.5 below, I will briefly summarise the development of the model for the estimation of seismic risk in the Groningen field due to the induced seismicity. Before doing so, it is fitting to provide an overview of the data acquisition and analysis activities undertaken by NAM, directly, through contracts and via open sharing of the acquired data with research groups, to underpin the risk model. For reasons of space, I only provide a condensed summary of some of the main research activities, but my hope is that this will convey to the reader the unprecedented scale of the efforts made to characterise all the elements from the risk model from the cause, gas production, through to reservoir compaction and the final effects of ground shaking on building response.

From the perspective of understanding the mechanics of the reservoir depletion and compaction, in addition to pressure measurements in wells and a field-wide gravity survey, a fibre optic cable has been installed over the reservoir section of a deep observation well and new* in situ* compaction measurements have been obtained. At the surface, NAM has commissioned levelling surveys, installed continuous GPS at selected locations, and established a network of 28 marker monuments over the field, as well as acquiring monthly InSAR surveys. To obtain information regarding the rupture processes associated with the earthquakes, geophones have been installed in three existing observation wells that extend to the reservoir and also in two new wells drilled as part of the new data acquisition. Rock cores recovered from the reservoir and the underlying Carboniferous formation were tested in laboratories at the University of Utrecht in the Netherlands and at the NIED laboratory in Tsukuba, Japan (e.g., Hunfeld et al. 2017; Spiers et al. 2017; Pijnenburg et al. 2018, 2019; Pijnenburg and Spiers 2020; Buijze et al. 2020).

In terms of seismic monitoring at the surface, KNMI has operated some borehole seismometers. With support from NAM, four broadband seismographs were installed in 120 m boreholes to improve the monitoring capacity. Extensive work has also been undertaken on analysis and refinement of the earthquake catalogue, including work undertaken directly by NAM (Willacy et al. 2019) and in collaboration with independent researchers (Smith et al. 2020), which has complemented work undertaken by KNMI (Spetzler and Dost 2017). Work undertaken in collaboration with KNMI derived empirical relationships between moment magnitude and local magnitude for Groningen earthquakes (Dost et al. 2018).

KNMI has operated a network of 10 accelerogaphs in the northern part of the field (called the B-network), which was expanded (to 18) and upgraded following the Huizinge earthquake (Dost et al. 2017). NAM funded the installation of network of 80 additional stations with the same instruments (called the G-network), 70 of which are co-located with boreholes housing geophones at depths of 50, 100, 150 and 200 m (Dost et al. 2017). NAM also funded the installation of additional 350 accelerographs, some in public buildings but most in private homes, for which the owners were able to request such an instrument (Ntinalexis et al. 2019). New processing procedures were developed to optimise the information retrieved from the recordings obtained of the small-magnitude Groningen earthquakes (Edwards and Ntinalexis 2021). Additionally, very dense networks of surface geophones were deployed for limited periods at different locations of the field to monitor ambient noise levels in order to estimate V_S_ of the shallowest layers (Spica et al. 2018a); earthquake recordings from these dense arrays were also used to constrain models for the spatial correlation of ground motions (Stafford et al. 2019). The dynamic characteristics of the B-network strong-motion stations were determined through * in situ* V_S_ measurements using a variety of techniques (Noorlandt et al.^2018^), from which seismic CPT (cone penetration test) was identified as a suitable method that was subsequently applied to nearly all the G-network stations. Analysis of horizontal-to-vertical spectral ratios was also used to verify the site characterisations (Spica et al. 2018b). To provide additional constraint on the ground motion modelling, including the effect of the high-velocity Zechstein salt layer overlying the reservoir on seismic wave propagation (Kraaijpoel and Dost 2013), numerical simulations were performed to determine the geometrical spreading characteristics (Edwards et al. 2019).

The surface deposits over the Groningen field consist of soft clays, peats and sands, which can have a pronounced effect on the surface ground motions. To provide the basis for a field-wide site response model, a V_S_ model from the surface to the selected reference rock horizon at the ~ 800 m depth (the base of the North Sea formation) was constructed (Kruiver et al. 2017). The uppermost part of the profiles was based on the GeoTop geological model, applying empirical relationships to assign V_S_ values to the different lithological layers at different depths (Kruiver et al. 2021a). The deep part of the profiles was based on direct measurements made in the new deep wells. To bridge the gap between the geology-based shallow V_S_ profiles and the deep well logs (from about 50 to 150 m), an inversion was performed of surface waves recorded (and considered noise at the time) during the deep seismic reflection profiling of the reservoir in the 1980s—in effect, MASW on a very large scale. Laboratory work was also undertaken to determine the dynamic properties of Holocene peats in Groningen (Zwanenburg et al. 2020). A special study was also undertaken to determine the dynamic characteristics of the dwelling mounds (known in Groningen as *wierden*) on which a small proportion of the building stock is situated (Kruiver et al. 2021b). The complete dataset of processed ground-motion recordings and shear-wave velocity profiles, both at the recording stations and over the entire field, are now being made available for download by any groups interested in using the data for general research or indeed for specific applications to Groningen (Ntinalexis et al. 2022).

To develop a risk model, a key step in the work was the development of an exposure model for the ~ 250,000 buildings in the area defined for the risk study by the field boundary and a 5 km buffer on land. Since the primary focus of the risk model is the risk of injury, the focus has been on the occupied buildings, which account for about one half of the total; the remainder are bicycle and garden sheds, garages, etc. The buildings have been classified by their construction type and materials, height, age, and purpose, from external observations and examination of drawings available at municipality offices.

**Fig. 142:**
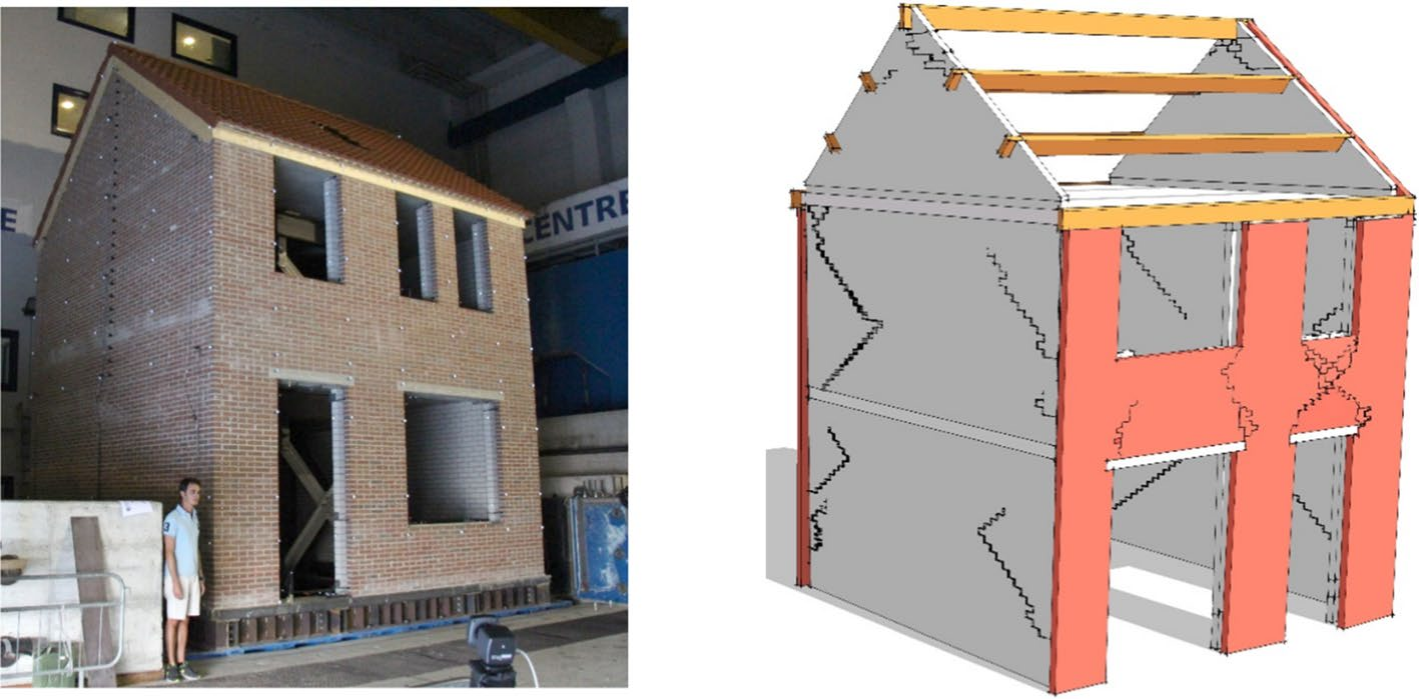
Left: Full-scale masonry structure built for shake table test in Pavia; right: observed damage pattern under strong dynamic loading (van Elk et al. 2019)

Rather than adopt fragility functions based on inferred analogies for the Groningen building types (which differ in many respects from the building stock in other regions, particularly seismically active regions for which most fragility functions have been developed), a very extensive programme of work was undertaken to determine the dynamic response and strength characteristics of the main building classes. This work has included* in situ* testing on many masonry buildings and laboratory tests on both extracted and constructed building elements (Graziotti et al. 2019). The pinnacle of these investigations were dynamic shake table tests on full-scale buildings, which have served to calibrate the advanced structural analyses performed to derive the fragility functions (Graziotti et al. 2016, 2017; Brunesi et al. 2019; Tomassetti et al. 2019; Malomo et al. 2020a, 2020b, 2020c). The tests, conducted in Pavia (Italy) and Lisbon (Portugal), involved the transportation of Groningen building material and builders to these locations to construct full-scale models (Fig. 142) that were then subjected to cyclic and fully dynamic testing. To account for the presence of soft soils throughout most of the field, extensive soil-structure interaction analyses were also performed (e.g., Cavalieri et al 2020a, 2020b).

#### Modelling seismic hazard and risk

**Fig. 143:**
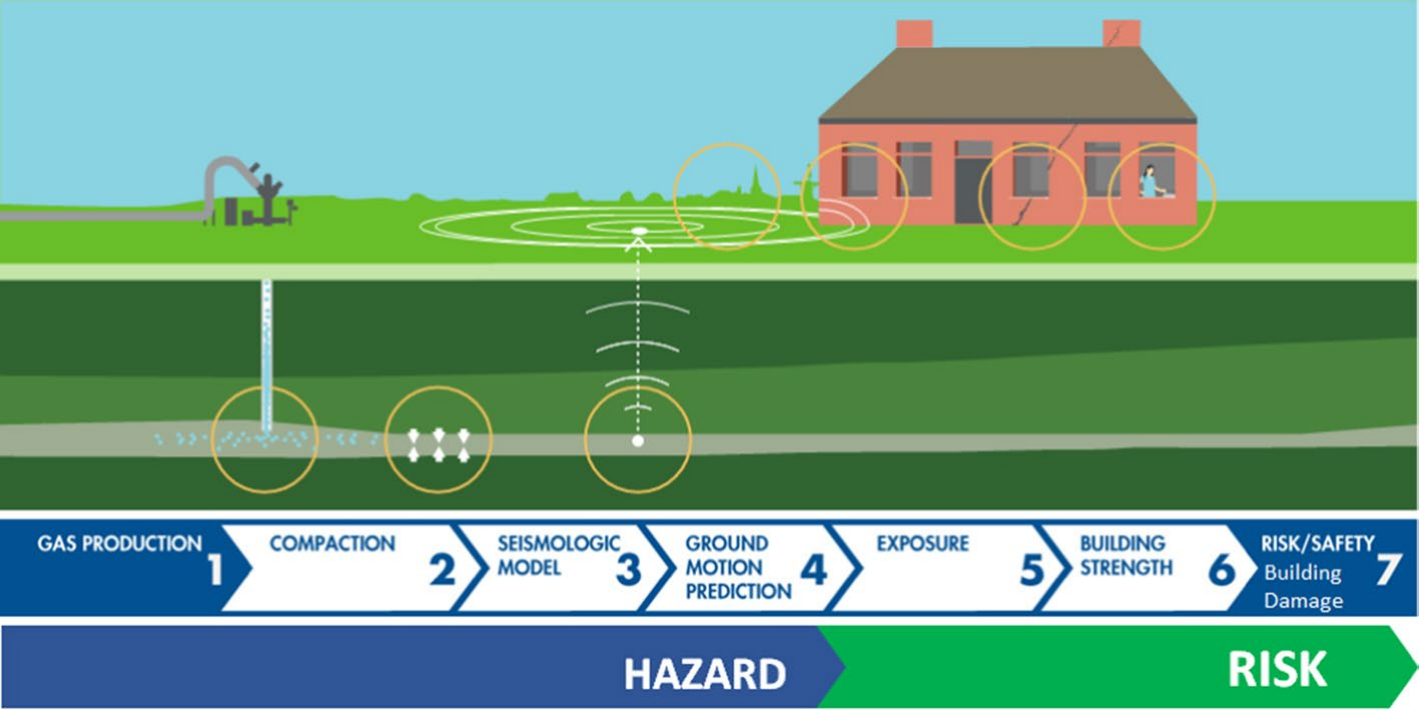
Schematic illustration of the steps in the Groningen seismic risk model (Courtesy of NAM)

A comprehensive seismic risk model has been constructed for the induced seismicity in the Groningen field (Fig. 143), which is performed in a Monte Carlo framework, which is computationally intensive but brings many advantages (Bourne et al. 2015). The first part of the risk model is a seismicity model that defines rates and locations of future earthquakes of different magnitudes on the basis of predicted reservoir compaction for the projected gas production levels (Bourne et al, 2014, 2018; Bourne and Oates 2017); the hazard and risk estimates are therefore always tied to a particular period and the planned production rates during that period. The starting point for the risk modelling is a prediction of the reservoir compaction. The field operator already had a mature dynamic model for the reservoir pressure based on gas withdrawal, which had been matched to observational data over the long production history. The reservoir compaction could then be calculated from the pressure changes, and the predictions of compaction have also been checked against measurements of surface subsidence obtained from levelling and remote sensing measurements.

The next element of the model is a GMM derived specifically for the field, which predicts response spectral ordinates at a reference rock horizon at ~ 800 m depth and then transfers the predicted motions to the ground surface through frequency-dependent non-linear site amplification factors (Bommer et al. 2017). These amplification factors are defined for ~ 160 zones that cover the entire area for which the risk calculations are made (Rodriguez-Marek et al. 2017). The final elements of the model are the exposure database, the fragility functions derived for each building typology, and consequence functions to estimate the impact of different degrees of structural damage (Crowley et al. 2017a, 2017b, 2019; Grant et al. 2021).

**Fig. 144:**
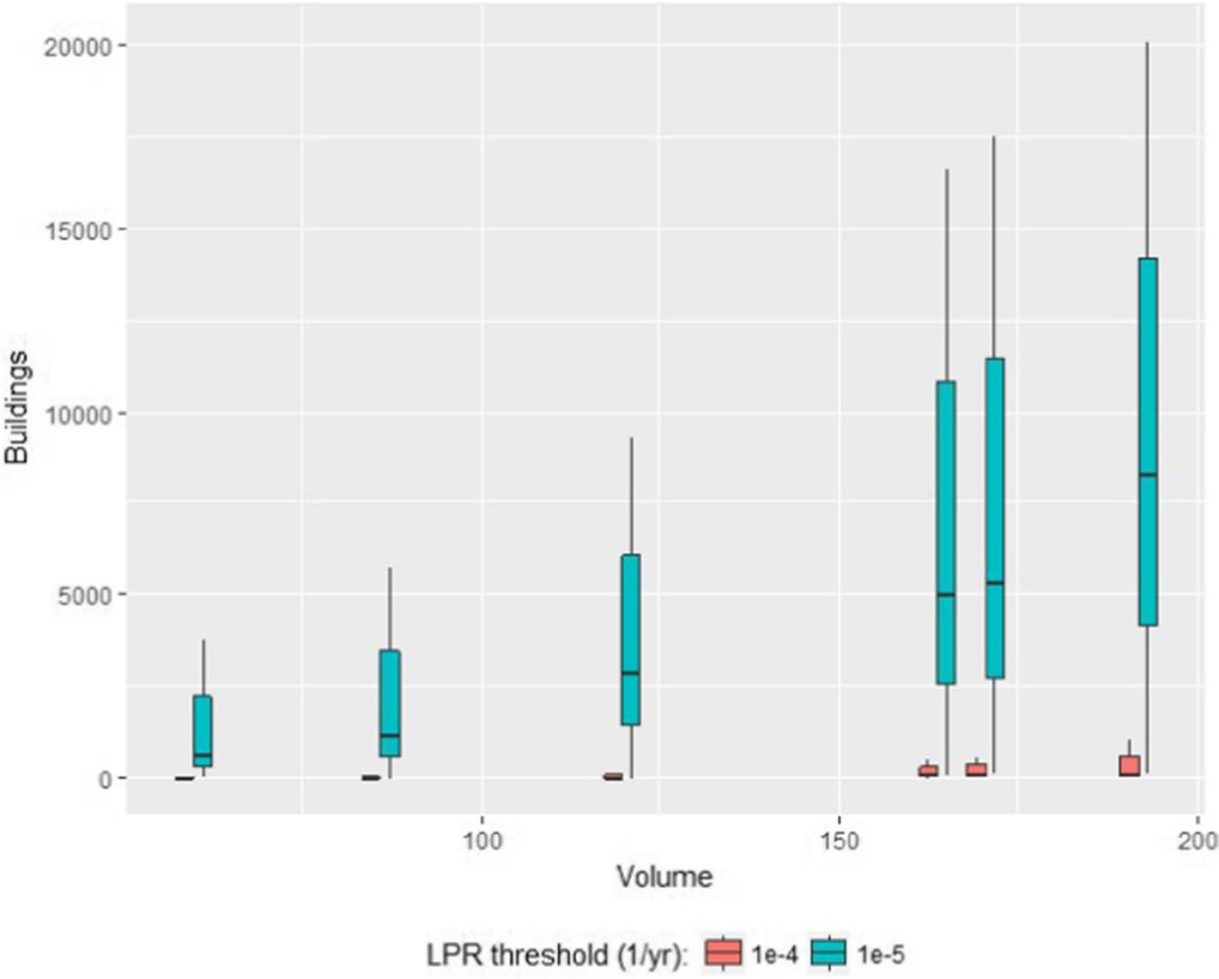
Risk estimates expressed in terms of numbers of buildings failing the LPR criterion at annual probabilities of 10^–4^ (red) and 10^–5^ (green) as a function of the total volume of gas production (in bcm) for the period 2018–2022. The boxes represent plus and minus one standard deviation, and the lines indicate the minimum and maximum values (van Elk et al. 2019)

The main risk metric employed is the Local Personal Risk (LPR), which is the probability of injury to a person permanently situated at a given location. The model output can be expressed in a variety of ways, including spatial distribution of LPR estimates and estimates of the number of buildings exceeding defined thresholds for the LPR as defined in Dutch safety regulations (Fig. 144). The model is also able to calculate Group Risk.

The development of the risk model underwent extensive peer review, both through the process of publication in journals and through the appointment of international panels of experts who were engaged in workshops and for remote review of the documentation of different elements of the model. By way of illustration, the panel engaged to review the development of ground motion and site response models included the following renowned researchers and practitioners in this field: Norm Abrahamson, Gail Atkinson, Hilmar Bungum, Fabrice Cotton, John Douglas, Jonathan Stewart (chair), Ivan Wong and Bob Youngs. For the exposure and fragility model development the review panel consisted of Jack Baker (chair), Matjaz Dolsek, Paolo Franchin, Michael Griffith, Ron Hamburger, Curt Haselton, Jason Ingham, Nico Luco, Marco Schotanus and Dimitrios Vamvatsikos. To provide quality assurance on the risk engine, the complete risk model was implemented independently in two coding languages (Python and C) and only accepted when they yielded both intermediate (hazard) and final (risk) results that agreed within very narrow tolerances.

**Fig. 145:**
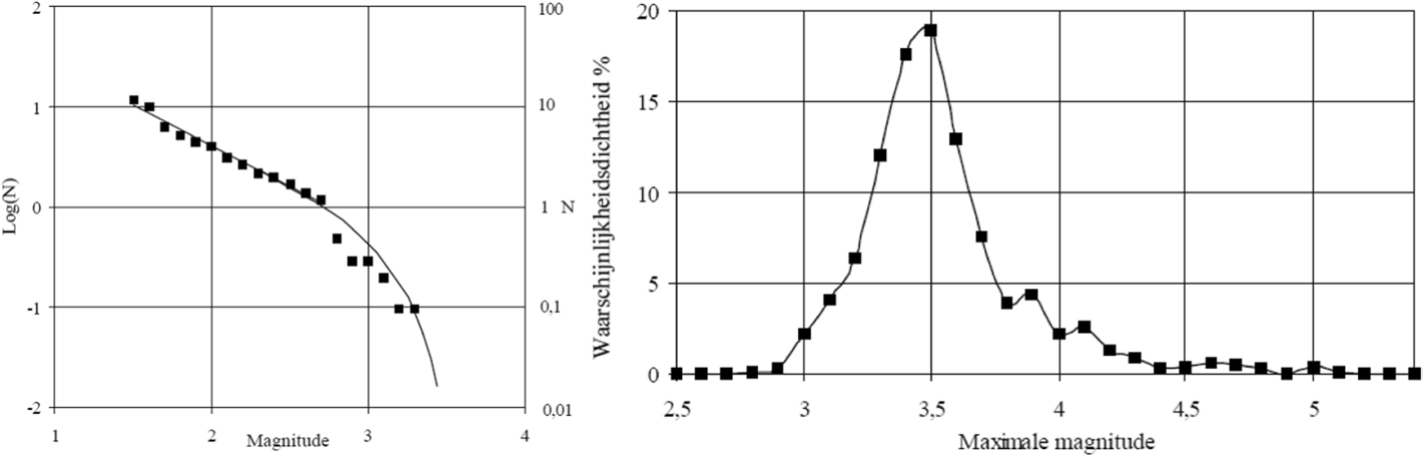
Left: Bounded Gutenberg-Richter recurrence relationship for the northern Netherlands; right: probability density for different Mmax estimates from 1000 Monte Carlo simulations (de Crook et al., 1998)

As can be appreciated from Fig. 144, the risk estimates included epistemic uncertainty. Logic-tree nodes were developed for each element of the model with the intention of capturing the epistemic uncertainties. The reason that the range of uncertainty is quite large for the higher production rate scenarios, despite all of the data collection activities and analyses described in the previous section, is mainly the extrapolation to magnitudes far larger than the maximum of M_L_ 3.6 for which data are available. This reinforces the view expressed in Sect. 9.2 that for induced seismicity, the estimation of Mmax is critically important. The history of Mmax estimates for the Groningen field is worth briefly summarising. The earliest estimate was made by a body called *Begeleidingscommissie Onderzoek Aardbevingen* (BOA, Advisory Committee on Earthquake Investigation), which in 1993 issued a report that estimated Mmax as being in the range 2.9 to 3.3 (de Waal et al., 2017), although it should be noted that this was not specifically for the Groningen field but rather for earthquakes around Assen, south of the Groningen field. KNMI subsequently issued new estimates in 1995, for which two approaches were used: the first was based on the cumulative trend of released seismic energy, which yielded an Mmax of 3.3; the second was based on the dimensions of geological faults, which gave an Mmax of 3.5. These estimates were revised by KNMI in 1998 (de Crook et al. 1998), the two approaches now yielding values of 3.7 and 3.5 respectively. A third approach was also implemented, which involved Monte-Carlo simulations for Bayesian updating of the cumulative magnitude-frequency relation using a bounded Gutenberg-Richter equation (Fig. 145). This final method yielded the highest estimate, based on the median-plus-one-standard-deviation result, of M_L_ 3.8 for Mmax. The same Bayesian approach was applied a few years later by van Eck et al. (2006), leading a slightly modified 84-percentile estimate of 3.9 for Mmax. This value was not revised again prior to the 2012 Huizinge earthquake, so it may be concluded that the prevailing view on the expected largest magnitude of earthquake in the field in August 2012 was M_L_ 3.9.

A related question is how likely these largest possible earthquakes were thought to be, which is not easy to ascertain since the recurrence model adopted for the KNMI studies is the doubly truncated exponential model adapted from the standard Gutenberg-Richter relationship, in which the annual frequency of an earthquake with Mmax is vanishingly small. Moreover, Mmax is an estimate of the largest earthquake that is considered feasible, but that does not mean that it is necessarily expected to occur. With regard to the early KNMI estimate of 3.5 for Mmax, Roos et al. (2009) state that this had a 1% probability of being exceeded.

For the initial hazard model prepared in 2015, Mmax was set very conservatively to 6.5, based on the assumption of the reservoir compaction from full depletion of the reservoir being released seismically in a single event. As the influence of this parameter became apparent, it was clear that such a conservative approach could have far-reaching (and unintended) consequences. In order to estimate the distribution of Mmax, and in view of the potential controversy associated with this parameter, NAM commissioned an independent panel of experts to make the assessment, informed by presentations and discussions at a three-day workshop hosted in Amsterdam in March 2016 (Bommer and van Elk 2017). The resulting distribution of Mmax values was shown in Fig. 91, with a peak at magnitude 4.5 but a tail extending out to just above magnitude 7; the expert panel effectively defined events of magnitude greater than 5 as triggered events that would necessarily rupture outside of the reservoir. Even though the weights assigned to the highest magnitudes in the distribution are small, the uncertainties associated with the ground motions from such scenarios are obviously very large. Indeed, we do not even know what the fault ruptures of such earthquakes would look like: they would presumably initiate inside the reservoir and propagate downwards into the Carboniferous basement. As was noted in Sect. 9.2, the Groningen Mmax distribution will be re-visited in the near future. If the risk calculations were to be performed only for induced earthquakes (therefore not exceeding magnitude 5), the uncertainties would be very considerably smaller given the more modest extrapolation beyond the data and the unparalleled wealth of data available for the Groningen field.

The Mmax workshop was organised following the principles (but not, it is acknowledged, the strict requirements) of a SSHAC process (see Sect. 6). As was noted in Sect. 9.6, there had been both the desire and intention to conduct the entire seismic risk assessment for Groningen as a SSHAC Level 3 study, which would have been, to my knowledge, the first application of the process to induced seismicity and also the first application to a risk study for buildings (there has been an application to fragility functions for dams in the US). I am convinced that this would also have an ideal vehicle to structure discussions of the uncertainties and controversies surrounding the induced seismicity in Groningen in a transparent manner that could have been closely followed by the regulator and other stakeholders. However, for this to have been feasible, it would have been necessary to avoid a parallel review process and periodic updates of the risk estimate during the execution of the SSHAC study, and these conditions were deemed unacceptable to the regulator hence this option could not be pursued.

In passing, it can also be noted that the risk modelling effort also addressed the hazard of liquefaction triggering (Green et al. 2019, 2020). The analyses were not extended to risk since it was found that even for the most susceptible area of the Groningen field, the probability of liquefaction triggering was very low, and even this very small hazard was driven by the upper end of the Mmax distribution.

#### Risk mitigation strategies

**Fig. 146:**
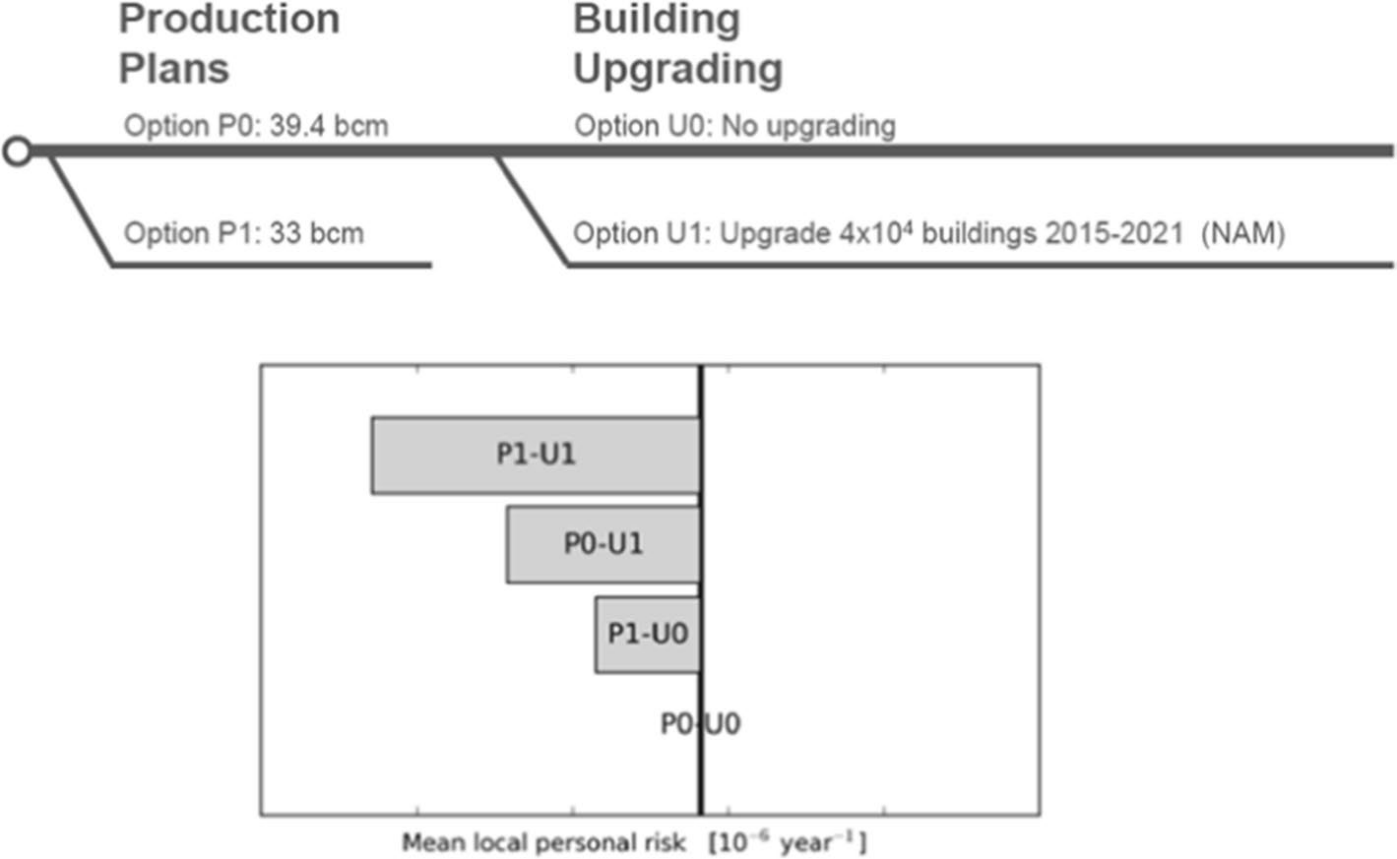
Upper: Logic tree for risk mitigation options based on reduced production (P) and structural upgrading (U); lower: impact of mitigation strategies relative to baseline case (solid line) for an early proof-of-concept model (NAM 2015)

The express purpose of the Groningen seismic risk model was to inform decision making regarding mitigation measures to reduce the impact of induced seismicity. As demonstrated by Fig. 144, the model can estimate the impact of changes in the gas production levels on the resulting risk. However, the model can also estimate the reduction in risk from targeted structural strengthening interventions on selected buildings (Fig. 146). The risk model can identify both the areas and the structural typologies contributing most to the risk estimates, which can in turn prioritise and guide field inspections to develop an inventory of buildings to be strengthened (e.g., Palmieri et al. 2020). Modified fragility functions were then developed for structures that had undergone strengthening, in order to calculate the risk reduction achieved with these measures. The model effectively allowed exploration of multiple mitigation strategies that combine both reductions in gas production and house strengthening interventions, which would allow optimal choices to be made regarding the balance between reduced risk and maintenance of gas supply.

In a paper published in *The Leading Edge*, staff from the field regulator (Muntendam-Bos et al. 2015) made the following statements: “*Risk management depends on the ability to apply control measures. For seismic risk resulting from gas production, there are preliminary indications that seismic activity can be reduced by reducing gas-production rates. In addition, the consequences of earthquakes can be mitigated to a certain extent by adopting a preventive strengthening program aimed at strengthening the most vulnerable buildings and infrastructure to an acceptable level*.” While this acknowledged that building strengthening could contribute to risk mitigation, the implication is that it is less reliable and less effective than changing the production. At that time, restrictions on the gas production levels had already been imposed, and the authors cite van Thienen-Visser and Breunese (2015) as showing that this was already leading to reductions in the earthquake activity. However, I would contend that house strengthening is the more robust approach to risk mitigation, since there is uncertainty related to the future seismicity levels and how they will respond to reductions in production—although an ‘experiment’ is now being conducted that will provide insight on this issue (see Sect. 12.4.8)—whereas the application of established earthquake engineering retrofitting techniques can yield enhanced seismic reduction, with a consequent reduction to the seismic risk, with high confidence. In a subsequent publication by staff from the regulator, no reference at all was made to the option of building strengthening, the article focusing exclusively on the observed reductions in seismicity as a result of production restrictions that had been imposed. The article concluded with a very interesting statement: “*Along with the decrease in seismic activity, the public commotion related to the seismic risk has also declined. Currently, public displeasure is focused mainly on the process of damage handling and compensation*” (Muntendam-Bos et al. 2017). These words almost seem to indicate that with the production limits that had been imposed, the problem was largely resolved, provided the payment of damage claims would be accelerated—which Fig. 140 suggests did indeed happen. However, the apparently optimistic outlook expressed in 2017 did not persist, possibly because earthquakes continued to occur, including the M_L_ 3.4 2018 Zeerijp and 2019 Westerwijtwerd earthquakes, both mentioned previously, regardless of the reduced production levels. This interpretation would seem to be consistent with the following statements from a later paper co-authored by staff from the regulatory body: “*Risk assessment is only the first step toward risk management. Several production-reducing measures have been imposed on the Groningen gas field, with the aim of reducing seismic activity. This aim has been achieved, at least for the short term (2014–2017). A recent earthquake (January 8th 2018, magnitude 3.4) may change this assessment. The attainability of managing seismic activity in the small gas fields (e.g. by a traffic light system) has yet to be demonstrated. Whether operational measures to limit the number and strength of induced events exist remains highly uncertain, especially for fields at the end of their lifecycle. This is currently being investigated*.” (van Thienen-Visser et al. 2018). A focus on physical risk mitigation through the reduction of fragility in the buildings contributing most to the risk estimates rather than only on hazard control through production limitations would have provided a more robust approach—as had been proposed by Bommer et al. (2015a). A house strengthening programme is underway in Groningen, responsibility for which, like the claims handling, has now been taken away from NAM, but limited progress has been made, and now the opportunity to implement a concerted programme of structural upgrades in order to manage the seismic risk has been lost.

Before closing this discussion, I note, for completeness, that there have been serious discussions over many years regarding the possibility of injecting large volumes of nitrogen into the reservoir in order to maintain the pressure and prevent further compaction. While the simplicity of the concept is attractive, the idea was not implemented since there are numerous challenges including very high costs, the potential of the injection of gas having unforeseen effects (including induced earthquakes), and the fact that over time the nitrogen would mix with the remaining gas reserves.

I should also mention once again the idea that was floated by Bal et al. (2019)—see Sect. 10.3—of NAM paying out financial compensation following every episode of felt shaking to those affected (i.e., shaken). While this would have had no impact in terms of reducing the physical risk, it would possibly have addressed the displeasure referred to Muntendam-Bos et al. (2017) in the quote cited above.

#### Dysregulated regulation

The regulatory body referred to in the preceding sections is SodM (*Staatstoezicht op de Mijnen*, the State Supervision of Mines). The role of SodM, in the case of the Groningen gas field, is actually advisory rather than regulatory since the gas production levels in the Groningen field, for reasons related to security of energy supply, are set by the Ministry of Economic Affairs and Climate Policy (EZK), informed by advice from SodM. The role of SodM is also not exclusive since during recent years EZK has also sought scientific advice regarding the induced seismicity in Groningen from other bodies, including the Science Advisory Committee (SAC), chaired by Dr Lucia van Genus (President of the Royal Geological and Mining Society of the Netherlands, KNGMG), which was active during 2015 and 2016 in reviewing the development of the NAM seismic risk model and reporting to the Minister of EZK.

As will be discussed in Sect. 13.4, I believe that effective regulation is probably the single most important factor in achieving rational management of the potential risks presented by induced seismicity. I also believe that much can be learnt from the regulation of nuclear facilities, for which there is a tremendous body of experience to draw upon. As well as engaging with nuclear regulators in several countries through work on seismic hazard studies for nuclear sites, I have worked directly for the UK Office for Nuclear Regulation (ONR) and the US Nuclear Regulatory Commission (USNRC), and I think that both these agencies provide exemplary models for how regulation may be conducted. Regulation can be prescriptive, where the licensee is provided with clear guidelines to follow regarding the quantification of risk (which is the USNRC approach) or non-prescriptive, where the regulator establishes the goals to be met but leaves it to the licensee to determine how compliance with these goals is demonstrated (which is the approach used by ONR). In practice, the distinction can be exaggerated because USNRC does allow licensees to adopt alternative procedures (but counsels that this is likely to delay the assessment of license applications) and because the guidelines produced by ONR for its own inspectors are generally viewed as requirements by licensees. In either case, however, a basic principle is that the licensee is expected to undertake the seismic characterisation of the site and calculate the consequent risk to the plant, and the regulator interrogates and challenges the technical bases for these assessments to inform their judgement as to whether the assessments are reliable. In other words, it is essentially the role of peer reviewer, which is not to specify what the results of the study should be but to determine whether the study has been conducted correctly. I have never seen a nuclear regulator issue its own technical assessments, produced without peer review, and put these in front of a licensee, in effect asking them to accept or disprove the regulator’s own scientific conclusions.

**Fig. 147:**
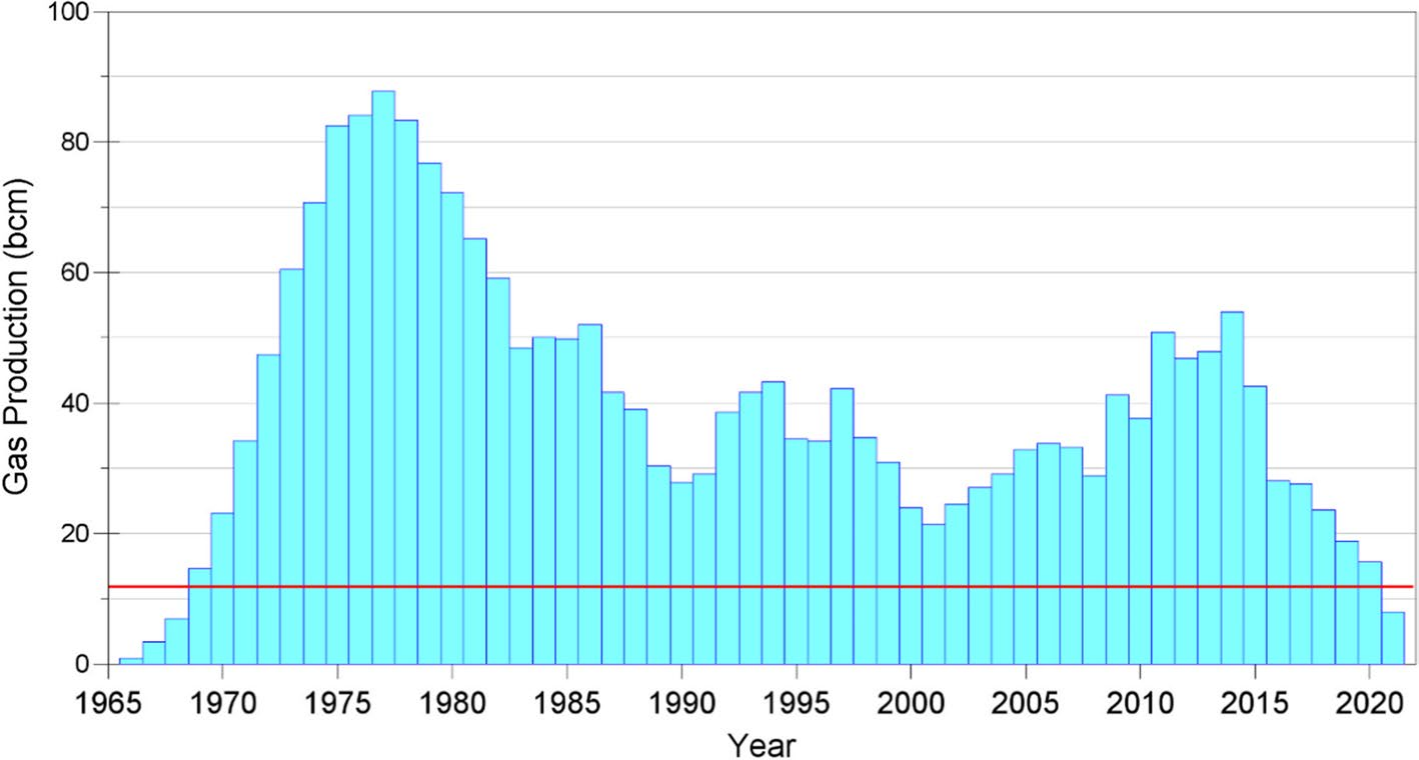
Annual gas production levels up to 2021, showing the decreases since the 2012 Huizinge earthquake; the red line shows the 12 bcm level below which SodM proposed that all induced seismicity of M_L_ ≥ 1.5 would cease. Note that the figure shows production per calendar year rather than per gas year (which starts on 1^st^ October)

However, this is exactly what happened in Groningen. In January 2013, a few months after the Huizinge earthquake, SodM issued a remarkable report (Muntendam-Bos and de Waal 2013). The report presented an analysis of the induced seismicity in Groningen and its correlation with the gas production from the field. One of the report conclusions was that analysis of the seismicity catalogue alone could not constrain the value of Mmax, which could clearly be greater than M_L_ 3.6 and could also be larger than the previous estimate of M_L_ 3.9. This conclusion was uncontroversial and was widely accepted. The report also concluded that the seismicity is driven by both the total volume of gas produced and the production rate, using a model that has been developed by one of the authors of the report (de Waal 1986; de Waal and Smits 1988). On the basis of this model, the report proposed that it would be necessary to reduce the annual production rate to 12 bcm in order to ensure that there would be no earthquakes of M_L_ ≥ 1.5. This gave rise to the slightly bizarre situation in which the field operator, NAM, argued for a lower production rate than the regulator: given that NAM’s own analyses did not support the rate-dependent model, its position was that if the risk control objective was to eliminate all seismicity of magnitude M_L_ ≥ 1.5, the only option would be to end gas production. It is interesting to note that the rate-dependent model has not found much support: KNMI, which was consulted extensively by SodM during their analyses, insisted on including a disclaimer in Muntendam-Bos and de Waal (2013) report to state that the official Dutch seismological service could not support the conclusions based on the model that made the seismicity dependent on the rate of gas production. More recently, de Pater and Berenten (2021), analysing induced seismicity at several gas fields, in and without the Netherlands, concluded that “*compaction dominates seismicity and rate effects are negligible. As yet, no evidence exists for the proposed seismicity-free production rate*”. There is now also strong empirical evidence that the rate-dependent model and the proposed production threshold of 12 bcm are fundamentally flawed. The production rates have been cut drastically as the field moves towards closure (see Sect. 12.4.8), and during the gas production year from 1^st^ October 2020 to 30 September 2021, the rate fell below 12 bcm for the first time (Fig. 147), yet the seismicity continues. Moving into the current gas year, production rates have dropped even lower, and yet just after a full year with production rates 25% lower than the threshold that was proposed to end all earthquakes, an earthquake of M_L_ 2.5 occurred at Zeerijp on 4 October 2021, and a few weeks later, on 16 November 2021, an M_L_ 3.2 earthquake occurred at Garrelsweer.

The 2013 report has also not been an isolated case of the regulator adopting its own scientific positions, as shown by the publications cited in the previous section and others (e.g., Muntendam-Bos 2020,4) that directly relate to the Groningen seismicity and its interpretation, which in turn underpins all hazard and risk modelling. The following is from one of the most recent publications by SodM staff in the open literature, which is worth citing in full:

“*The HRA* [hazard and risk assessment]* used for the Groningen gas field is of high quality and is considered as state of the art by international experts. However, close examination shows that several known and unknown uncertainties are not taken fully into account. In line with ISO 17776 Annex A when dealing with weak knowledge one should apply either stress scenario's* [sic]* or apply a safety factor. Therefor* [sic]* for defining the measures to ensure safety it was decided that a safety margin has to be taken into account. It was decided to base the scope of the strengthening program for buildings on the P90* [90% confidence level]* risk derived from epistemic uncertainties in the logic tree. Although this decision sparked some discussion it has provided the necessary contingency in the housing strengthening program as the PSHRA models are improved and refined and the derived continuously resulting in fluctuations of the calculated risk.*” (van der Zee and Muntendam-Bos 2021)

There are several remarkable features of these declarations, including the effective classification of the state-of-knowledge regarding seismicity, ground motions and structural fragility in the Groningen field as “weak”. If the multi-million Euro, multi-year investment in data collection for Groningen (Sect. 12.4.4), supported by analyses conducted and reviewed by international teams of highly qualified and experienced professionals (Sect. 12.4.5), only results in a ‘weak’ state of knowledge, there is little hope for ever being able to rationally manage the risk from induced seismicity. If peer review by international panels of experts leaves major—but unrecognised and unknown(!)—uncertainties aside, the entire discipline of seismic risk analysis would seem to be in early infancy rather than the mature state I believe to be the case. The fluctuations in the calculated risk alluded to in the quote have mainly been the consequence of the insistence of SodM and the Ministry EZK for full hazard and risk assessments at frequent intervals, which have never allowed the internal iteration of the models prior to implementation (as would have happened had the risk study been conducted as a SSHAC process, as proposed by NAM in 2016). This quote and those in the previous section all allude to the view of the regulator—despite their own bombastic and now disproven declaration in 2013—that the risk could not be reliably modelled or controlled, a view arising from focusing on control of the seismicity as the primary tool for risk mitigation and a lack of appreciation for how earthquake engineering could very effectively diminish the risk. In effect, the regulator’s position has tended towards the precautionary principle, with the inevitable outcome from such an approach to risk management, as discussed below in Sect. 12.4.8.

In closing this discussion, my hypothesis is that while the Huizinge earthquake was the largest to have occurred in the Groningen field, it does not explain the events that have unfolded since either in terms of the uncontrolled payment of damage claims that far exceed the possible consequences of the seismicity or in terms of the decision to the close the gas field (see next section). The turning point in this story, in my opinion, was the SodM report of 2013. In much the same way that the Juanes et al. (2017) report transformed the Castor situation into a crisis (Sect. 12.3), the report by Muntendam-Bos and de Waal (2013) was the first step in the manufacture of a crisis in Groningen.

#### The closure of the Groningen field

The decision has been taken to shut in the Groningen gas field and completely suspend production, the sole motivation for this decision being the induced seismicity. This is clearly a significant loss for Shell and ExxonMobil, the commercial shareholders of NAM, but it is also a major economic loss for the Dutch state. The Groningen gas field is a very major resource: it was the 7^th^ largest gas field in the world when it was discovered in 1959 and about a quarter of the total gas remains today—it is still within the top 15 global reserves. While the field was a lucrative asset for NAM, the main economic beneficiary has been the Dutch government, which through a variety of levies and taxes, is the main recipient (more than 90%) of income from the field (it is estimated that over the life of the field, the income to the Dutch state from Groningen has been on order of 1 trillion Euros).

Exactly when the field will shut in, however, is not entirely clear at the time of writing. During the 2021–2022 gas year, the specified production level is intended to be 3–4 bcm, after which production should cease. However, gas supplies to end users throughout the Netherlands—and in some neighbouring countries—needs to be ensured, which means that gas will be imported, mainly from Russia. An additional complication arises from the fact that the Groningen field produces low-calorific gas by virtue of containing ~ 14% nitrogen. Since all facilities that currently rely on Groningen gas are calibrated to burn this low-calorific gas, GTS (Gasunie Transport Services BV, the company responsible for the gas transmission network in the Netherlands) is constructing, at a cost of around 200 million Euros, a plant that will add nitrogen to the high-quality imported gas before passing it on as low-calorific gas to consumers. The construction of this plant is behind schedule at the time of writing, which will apparently lead to the final production levels in the current gas year being on the order of 7–9 bcm.

The implications of the closure of the Groningen as field may reach far beyond the Netherlands. Holliday (2021) has argued that the huge drop in production leading up to shut-in has been a major contribution to the global increases in gas prices in late 2021, and that it has also changed the balance of power in Europe by empowering Russia.

For completeness, I also need to note that in common with the Basel and Castor case histories, there have also been moves to prosecute NAM in the courts. In September 2015, the campaign group *Groninger Bodem Beweging*​ (https://groninger-bodem-beweging.nl/english/) reported NAM to the police for endangering lives by causing induced earthquakes; until now, however, the prosecutor has yet to make a decision regarding taking this forward.

Muntendam-Bos et al. (2022) stated in a very recent paper on induced seismicity in the Netherlands that “*extensive gathering of subsurface data and adequate seismic monitoring are therefore essential to allow sustainable use of the Dutch subsurface now and over the decades to come*”. However, the Groningen experience suggests that data collection and monitoring, at any scale, will be no match for politicised decision-making. Moreover, responding to public and political pressures, the Dutch government decided in 2018 that NAM would not provide further risk assessments (the last risk assessment by NAM was prepared in March 2020) and the scientific program led and funded by NAM will be closed out. No new study initiatives have been started since 2019. Currently, the last studies are being completed.

The impending closure of the Groningen gas field, with the consequences that this will have in the Netherlands and beyond, has come about because of an earthquake of magnitude M_L_ 3.6 (moment magnitude, **M** 3.5), which did not cause a single injury, let alone fatalities. Even more disturbing is the fact this has happened despite an investment of ~ 200 MEuro in data acquisition and in risk modelling, and despite a clear plan to manage the risk through measures including an extensive building strengthening programme that would have been fully funded by the gas company. Whereas this could have been an extremely valuable demonstration case for the rational management of induced seismic risk, it has been a colossal failure of science and engineering to overcome irrationality.

## Scientific assessment, engineering judgement, public opinion and regulation

In the preceding sections, I have attempted to demonstrate that we have made significant advances in distinguishing induced seismicity from natural earthquakes (Sect. 8) and that well-established procedures developed to quantify the hazard and risk due to natural earthquakes can be adapted to induced seismicity (Sect. 9). I have also tried to show that there are multiple options for mitigating the risk due to induced earthquakes, including both measures to control the hazard and the application of classical earthquake engineering to reduce risk through reduction of fragility (Sect. 10). In addition, I have argued that the global databases of small-to-moderate magnitude earthquakes can provide a framework for understanding the threshold sizes of earthquakes that can pose a threat to people and to the built environment, and also demonstrated how these thresholds are controlled primarily by the fragility of the exposed elements (Sect. 11).

However, in spite of all of these advances, Sect. 12 has painted a rather discouraging outlook, with four major projects related to energy supply being shut down as the result of induced earthquakes, all of which correspond to magnitude-distance scenarios that would generally not be considered a serious threat (Fig. 117). In this section I briefly discuss some of the factors that I believe have contributed to these situations and offer some thoughts on how these might be addressed. I am conscious that there is an extensive literature on risk perception and decision making that I am not drawing upon in these discussions (apart from a few papers specifically related to induced seismicity)—these are simply my own insights from my experience of working on these projects.

### Informing the energy debate

All the cases of induced seismicity that have been discussed in Part II of this article have been caused by operations that are related to energy supply, which is a much-debated topic in itself because of concerns regarding climate change and energy security. In some cases, induced earthquakes simply become another argument for those opposing a particular technology or energy source, which can lead to exaggeration of the impact of the induced seismicity since the intention is generally to portray the operations as sinister in many ways.

I have no doubt that attaining rational assessment of induced seismicity and balanced management of the consequent risks would be greatly assisted by improving the discussion concerning energy supply and consumption, which is often poorly informed, polarised and less than entirely honest. In terms of being poorly informed, there would appear to be a widespread misunderstanding of fundamental concepts. This was brought home to me through teaching at Imperial College London, when I introduced a new module for first-year undergraduates on Energy Supply and Infrastructure. The module began with an open debate on ideas for sustainable energy provision for the future to meet demand and address climate change, in which it became apparent that many students believed, for example, that electricity can be both efficiently stored and efficiently transported over large distances (these are very bright students who had finished their schooling without being taught the fundamentals of energy supply). Ten weeks later, after a couple of lectures on energy supply in general and several specific energy sources, a much more informed and constructive debate took place. Whitmarsh et al. (2015) present an interesting survey of attitudes to different energy technologies in the UK, noting in the first instance how views were largely influenced by factors such as demographics, political leanings, and environmental attitudes. Whitmarsh et al. (2015) also found that attitudes were changed when people were provided with more information, which enabled a more balanced cost–benefit assessment. Understanding the benefits and risk of all energy technologies and sources, and all the implications of both their use and their abandonment, would clearly help.

This brings us to the question of how polarised the energy debate has become, which again was demonstrated by the energy module at Imperial. After two lectures covering fundamentals of energy supply and economics, each of the successive weeks consisted of an invited lecture on a particular energy technology, and we were very fortunate to have excellent speakers give up their time to make presentations on several energy sources (including wind, solar, nuclear, geothermal, biofuels, hydrogen, energy from waste, and oil and gas). While the lectures were very interesting and informative, it was also apparent how many speakers were proponents for a particular technology rather than proponents for a balanced energy mix that included that technology. I think that there are two factors that seemed to contribute to this attitude, one being a perceived need to vilify other energy technologies in order to promote an alternative, and the clear sense that each technology is competing for limited government support in terms of subsidies and tax relief—which in turn would partly explain the tendency to criticise other energy sources.

Which brings us to the final point that the energy debate needs to become more honest, at all levels. On the one hand, proponents and providers of particular energy sources need to be honest about all of the costs, effects and risks of their technology, and opponents of any particular technology also need to be honest about the dangers and the benefits with which it is associated. While there is no doubt that in the past nuclear power plant operators and oil companies have clearly not been candid about their operations and their impacts, it would be naïve to assume that campaigners against these technologies are always open and truthful. The human condition seems to set us to argue to prove that we are right rather than discuss so that together we find the right answer, but the stakes in the energy debate are very high and such dualist outlooks will not solve the challenges. Rather than emotionally charged debate, what is required is a dialectical approach, a discourse among individuals and groups holding different views for the clear purpose of establishing the truth.

Another aspect of the honesty, I believe, is related to the expectation that governments alone can and will solve the issues of energy supply and climate change. Regardless of the source of the energy we use, our long-term survival as a species and as a planet will require us to use less energy, which is more likely to be achieved by radical changes to our lifestyles—particularly in the more affluent countries—rather than by more efficient technologies. There is, I believe, an inherent inconsistency in people expressing the view that it is exclusively the responsibility of governments to solve climate change: if governments were to impose the restrictions on travel and consumption necessary to immediately address increasing global temperatures and the ravaging of nature, it would be met with outrage. Governments, of course, have a critical role to play in determining energy policies and legislating to protect the environment, but the expectation that this can be done to only impact on large corporations without affecting our patterns of consumption is highly unrealistic. An often-stated claim is that we, as a society, are addicted to fossil fuels—I would argue that we are also addicted to very high energy consumption levels. If this is so, then perhaps a holistic solution to the energy issue will also require us to learn from those who have conquered addiction to other substances, in which a key step is a shift from blaming external factors to self-examination. The activist posting endless blogs and videos decrying the harm done by certain industries while ignoring the huge carbon footprint of the Internet^5^ may be as much a part of the problem as the targets of his or her criticism.

On the issue of climate change, there seems to be a general consensus regarding the need to move away from our reliance on fossil fuels, but what is less clear—not least because of the polarised and disingenuous nature of the debate—is how the transition will be made. What does seem to be clear is that a smooth and well-planned transition will be greatly preferable to one for which we are not prepared. An important concept in this respect is peak oil, a term first coined by Marion King Hubbert (Hubbert 1956), which corresponds to the moment in time when production rates of oil start to decline. Since the demand for oil continues to rise inexorably (apart from a brief period at the beginning of the Covid-19 pandemic), driven by growing population, industrialisation, and hypermobility, once peak oil is reached, a rapidly increasing gap would be created between demand and supply. In fact, if demand continues to rise then even a plateau in production rates would suffice to create the gap, which many have predicted would have very ugly economic and social consequences. Predicted dates for when peak oil would be reached have been superseded several times, due to factors including the discovery of new reserves and more effective retrieval technologies. The failure of predictions for when peak oil will happen has probably contributed to complacency regarding this issue, which sooner or later is inevitable. Bardi (2009) discusses the resistance to acceptance of the concept of peak oil, while Chapman (2014) proposes that it remains very relevant. Kerr (2011) argued that a decade ago oil production had already levelled off outside of the OPEC nations. Whether or not peak oil would have happened in the last decade is open to debate, but whether peak oil was averted or whether its due date was simply pushed out even further into the future, it is clear that the expansion of unconventional oil production—including hydraulic fracturing—has been instrumental in changing the panorama.

Some readers, who favour a rapid end to the use of fossil fuels, may have been gratified by the fact that induced seismicity shut down the three projects related to natural gas supply that were related in Sect. 12. Such a view would be, in my opinion, very naïve, since in none of these cases has the response been to replace the use of the natural gas with renewable energy sources such as wind or solar power—for the cases of UK shale gas and the Groningen gas field, it has simply meant a shift to imported natural gas from Russia and other providers. Other consequences have included potential shortages and huge increases of natural gas prices, which in many cases has resulted in increased use of coal and oil to generate electricity (Holliday 2021). For the case of Groningen, a study by Vergeer et al. (2015) forecast a significant increase in greenhouse gas emissions if the gas field were closed and replaced by imported gas from Russia.

Another point worth making is that those who support invoking small-magnitude induced earthquakes as a basis for discontinuing fossil fuel-based projects, need to be aware that the very same arguments have been used to close geothermal energy projects. The potential for induced seismicity and for induced seismicity to cause damage and injury must be taken seriously, as was shown by the Pohang geothermal project in Korea (e.g., Ellsworth et al. 2019), but exaggerating the impact of small-magnitude induced earthquakes as a means to discredit the causative energy technology is not helpful. Decisions regarding the energy mix to support any society need to be informed by reliable and realistic quantification of the costs, the benefits, and the risks (over the entire life cycle from design to decommissioning), including, wherever relevant, the possibility of induced seismicity.

### Preserving the value of scientific assessment

The starting point for dealing with induced seismicity, as I have already stated repeatedly, must be robust scientific assessments of the hazard. I would propose that for any induced seismic hazard assessment to constitute a useful starting point, it must fulfil four basic criteria: (1) the study must be carried out by suitably qualified professionals; (2) the study must be impartial and objective; (3) the hazard characterisation must include an assessment of the associated uncertainties, while also harnessing the constraint provided by the available data; (4) the assessment should be subjected to review and technical challenge. The SSHAC process provides a framework within which all four objectives should be satisfied as a matter of course (see Sect. 6).

I believe that there is also great merit in these assessments being made publicly available. The ideal forum for presenting assessments is authoritative scientific journals for which induced seismicity and seismic hazard are core topics rather than peripheral subjects. Induced hazard assessments should also preferably be presented in journals that publish full-length articles, supported by electronic supplements to share data and codes, rather than the very brief, and sometimes sensationalised, summaries that are characteristic, paradoxically, of the journals that are often viewed as the most prestigious. While I have no illusions that publication in a scientific journal is a guarantee that the study is entirely sound—with the number of journals nowadays and the pressure on academics to publish, the peer review system is severely stretched and frequently unreliable—peer-reviewed publication is still the best option, and the best way to dispel accusations of secrecy. Most journals publish comments and responses on articles, which provides a forum for intense scientific debate, and publication therefore demonstrates willingness to subject one’s hypotheses and analyses to scrutiny and challenge. In general, ideas and models that are published will eventually either find acceptance or meet rejection (whether through direct contradiction or simple neglect), according to their merit.

Articles in scholarly journals often have a limited reach, since the readership is generally limited to other researchers and perhaps a small number of practitioners in the same field. Many scientists seem to find themselves craving greater attention, and of course the Internet provides a simple route to a much broader readership. The problem is that the Internet is largely unregulated and the distinction between facts and fantasy is often difficult to make, especially for the larger non-specialist audience. However, if the claims are being made by someone with a PhD or an academic affiliation, they may appear to be reliable—especially if they resonate with the preconceptions of the reader or viewer. In this regard, fulfilling only the first of the four criteria listed above by a credible scientist disseminating views on the web, has the potential to either provide accessible education on complex topics to the general public, or to add considerably to the confusion and controversy surrounding induced seismicity. If a scientist has published work in the mainstream literature and uses the web to disseminate the findings, this may be very helpful; if the Internet is the only forum on which the proponent in this field is presenting their models and analyses, it is probably a cause for concern.

Even more surprising are the scientists and engineers whose appetite for publicity is so strong that they are perfectly happy to pronounce on topics entirely outside their own field of expertise. In researching the case of the Castor gas storage project (Sect. 12.3), I came across a documentary by Quest TV, which was part of a series entitled *Massive Engineering Disasters*. The short film presents a short history of the Castor project and seismicity that is full of inaccuracies, including statements that the caprock of the reservoir was broken and that the “*massive earthquake*”—also qualifying the seismic sequence incorrectly as “*the first quakes of this magnitude to ever hit the region*”—was caused by the Amposta fault (https://www.youtube.com/watch?v=cRXyUclQpjw). The shocking feature of the documentary for me, however, was that the talking heads speaking to these ‘facts’ and criticising the project operators for not foreseeing the outcomes of the gas injections, included an infrastructure expert, a space physicist and a bioengineer! The more critical viewer might ask how these individuals are qualified to speak to induced seismicity caused by gas storage, but for many observers they would simply come across as technical experts and their pronouncements would have carried authority. For the producers of the documentary, it would not have been difficult to track down the authors of some of the many journal papers published on the Castor seismicity, but their views would probably not have fitted well into the compelling and sensational (albeit largely fictional) narrative.

### Induced seismicity as a challenge for earthquake engineering

In the preceding section, I have emphasised the importance of robust scientific assessments of the hazard of induced seismicity, but the real issue—and a key theme of this paper—is the risk. To transform estimates of hazard into estimates of seismic risk requires the contribution of earthquake engineers. To date, however, it would appear that scientists (seismologists, geophysicists and geomechanics experts) have responded far more energetically to the challenges of induced seismicity than have earthquake engineers. I was impressed, for example, how the participants in the 3^rd^ Schatzalp Workshop on Induced Seismicity (see introduction to Sect. 8) were overwhelmingly scientists and there were no presentations that approached induced seismicity from the perspective of earthquake engineering. Consequently, there is a vast body of research on induced seismicity, of which a large part is motivated by scientific curiosity and by what induced seismicity can teach us about faulting, crustal stresses, and triggering of earthquakes. Such research is clearly worthwhile and enlightening, but its value could be further extended if combined with an engineering focus to seek solutions to the management of induced seismic risk.^6^ To be fair, some earthquake engineering researchers have engaged with the subject of induced seismicity, notably the research groups led by Professor Abbie Liel at the University of Colorado and Professor Jack Baker at Stanford University, and the European earthquake engineers who have been engaged in the risk modelling and house strengthening programmes for Groningen (Sect. 12.4).

The relatively low engagement of earthquake engineering (beyond ground-motion modelling) with induced seismicity might actually reflect the fact that induced seismicity generally does not pose a major engineering challenge, especially compared with the challenges of dealing with natural earthquakes in seismically active regions. However, if we are to achieve a rational assessment of the threat that induced seismicity may pose, we must move beyond hazard to risk, and this requires the active contribution of earthquake engineering.

The other clear benefit that would be obtained from more active participation by earthquake engineers in meeting the challenges of induced seismicity is that engineering solutions would more frequently be added to the menu of risk mitigation options. Currently, it is not at all uncommon for discussions of how to handle induced seismicity to entirely ignore the option of applying earthquake engineering to reduce structural fragility. A typical example is the following text from the paper by de Pater and Berensten (2021), cited in Sect. 12.4, on the factors controlling induced earthquakes in Groningen: “*Since seismicity only depends on compaction, there is little scope for management of seismicity: only pressure maintenance appears to be a viable solution. This can be accomplished by injection to preserve the mass balance or by shutting in gas fields*.” Knoblauch et al. (2019) discuss public preferences regarding the location of enhanced geothermal systems, balancing the benefit of district heating and green electricity with the possibility of induced seismicity; the study provides interesting insights, but the only risk mitigation option put to the participants in the surveys was reduction of the hazard through increased separation of the operations from the exposure. There will be many situations where earthquake engineering solutions are not economically viable, but in many other cases it could be a component of the risk management approach, even if limited to identification and strengthening (or even replacement) of any extremely vulnerable buildings.

### The role of regulation

Let us now assume a situation in which the application of particular energy technology is causing induced seismicity, and the hazard and risk have been robustly quantified through extensive data collection and analyses involving Earth scientists and earthquake engineers. How can the risk assessment be communicated to the public in a way that it will be appreciated, understood, and accepted? I regret that I do not have an answer to this question, but I can see many challenges. As I noted in Sect. 9.6, candid presentation of the risk assessment should include disclosure of the uncertainties, but these may easily be interpreted as indicating that the problem is poorly understood and therefore it could undermine rather than bolster assurance. For a public that is well informed regarding energy supply and the relative benefits (in terms of security of supply, cost, sustainability, and environmental impact) of different energy sources and technologies, there may be scope for objective communication of the seismic risks associated with some technologies. In a polarised situation, where ‘debate’ has been reduced to little more than the mutual vilification of antagonistic groups formed around entrenched ideological positions (who support or oppose issues as part of the ‘package’ that comes with the general political outlook rather than on the basis of any informed assessment), it may be pointless to even try.

At the end of the day, how the message is packaged may be less important than who conveys the message. Some studies have concluded that how messages regarding energy sources are received depend primarily on the degree of trust in those communicating the information. For example, Ryu et al. (2018) found that people living close to NPPs in Korea who trusted the government and the regulatory body were more likely to be accepting of nuclear energy. Tracy and Javernick-Will (2020) looked into attitudes towards induced seismicity related to oil and gas operations in the central United States, finding that people were generally more inclined to trust academics than government agencies. I believe that the responsibility must ultimately lie with an appropriate regulatory authority, and as stated previously, I believe that a great deal could be learnt from regulation in the nuclear industry. Of course, if there is general distrust of government and government agencies, the scope for a regulator to facilitate public assurance regarding the safe management of induced seismicity will be limited, but I remain convinced that this remains the most suitable path to rational assessment and management of induced seismic risk.

For a regulatory body to be effective in ensuring safety of operations with the potential to induce earthquakes and in assuring the public regarding the risk while also facilitating activities that bring societal benefits (especially in terms of energy supply), I would propose that there are several attributes that such an agency should possess:

- The regulator should have very clearly defined responsibility for the management of induced seismicity; in this regard, the regulatory body should have exclusive control over this issue without reference to other authorities or regulatory agencies. However, this authority and autonomy must be balanced by a system of checks and balances, so that complaints regarding any inappropriate conduct by the regulator can be referred to a higher authority, to which the regulator is accountable.
- The regulator should also have the ability, within the national framework for health, safety, and environmental legislation, to determine policy with regard to control of induced seismicity and mitigation of induced seismic risk. The final decisions, however, regarding implementation of energy technologies, will reside elsewhere since several other factors, including security of energy supply, also need to be taken into account.
- The regulator should publish (and update as required) clear guidelines for operators with regard to the expectations in terms of management of induced seismicity; as noted in Sect. 12.4, such guidelines may prescribe a series of steps to be followed or else define goals to be met, in the latter case encouraging licensees to follow relevant good practice to meet those goals.
- The regulatory guidelines or requirements should address the quantification and inclusion of all sources of uncertainty, and define performance targets that incorporate and accommodate the uncertainty; every effort should be made to avoid invoking the precautionary principle.
- The regulator requires the technical and scientific expertise to evaluate the induced seismic hazard and risk assessments. Given the highly specialist nature of this field, it is most likely that the regulator will need to contract external support in this regard, either on the project-specific basis or by appointing expert panels such as those which support the UK Office for Nuclear Regulation in the field of seismic hazard and climate change (https://www.onr.org.uk/external-panels/natural-hazards-panel.htm); the experts engaged should be well regarded within their scientific communities and preferably without any engagements by the industry being regulated. The regulator should also be able to rely on technical support from relevant national scientific bodies such as geological surveys and seismological services.
- Another option for engaging technical expertise for the evaluations is for the regulator to encourage licensees to adopt the SSHAC process and then to rely on the PPRP as the primary technical reviewer, to be supplemented by the regulator’s own assessment; it would not be inappropriate for the regulator to engage with the operator regarding the composition of the PPRP in such cases.
- The regulator should avoid issuing its own scientific positions regarding specific hazard and risk models, especially if these reflect the research of individual staff members, since this creates an unbalanced situation in which the licensee would then be required to adopt or disprove the model; moreover, if such a model is found to be flawed, then the credibility of the regulator is undermined. However, it could be appropriate for a regulatory body to jointly sponsor and endorse industry-wide studies that establish consensus models for elements of the hazard and risk assessments, as the USNRC has done for the development of regional SSC and GMC models to be used in PSHA at NPP sites in the central and eastern United States.
- The regulator’s engagement with licensees should be constructive (regulators and operators should have the common objective of safe operations) but also formal; when the regulator is present in meetings with the licensee or as an observer at workshops, non-binding verbal comments may be made, but all specifications of requirements should be communicated by letter, copied to relevant parties, and forming part of the official record of the assessment. Instructions to licensees should not be issued in telephone conversations, texts, or informal emails. Resolution of disputes between regulators and licensees should not require Freedom of Information requests to recover the paper trail.
- In general, the regulatory staff should adhere to strict codes of professional conduct, which then allows them to demand the same of the licensees. The regulator should have the willingness and authority to challenge any dishonesty or concealment on the part of licensees (and impose sanctions when necessary), but the default starting position should be one of mutual professional respect; experts engaged by the licensees to assist with hazard and risk assessments should be viewed as professionals of integrity rather than hired guns.
- The regulatory agency will inevitably be an instrument of government, but it should be autonomous to the extent that is possible. Equally important is for the regulator to be demonstrably independent from licensees. The regulator needs to be an honest broker, neither sacrificing safety considerations to meet government energy strategies nor allowing operators to fall short of the safety requirements.
- A key question is how regulatory bodies should be funded, which often is in large part from levies imposed on the licensees. Whether funded by industry or government, the arrangements should be designed to avoid the financial support in any way compromising the regulator’s autonomy. I would also argue that the funding should be sufficient to allow staff salaries and consultant fees to be paid at levels comparable to those in the industries being regulated, in order to create a level playing field.
- Finally, the regulator should be prepared to communicate to the public their policy and their decisions, and defend these, when necessary, against attacks from protest groups; pandering to the most vocal sections of civil society is not a basis for effective regulation.

I appreciate that this is an optimistic wish list but none of these suggestions should be unworkable, and without such a regulatory authority any attempts to achieve balanced assessment of induced seismic risks associated with energy technologies are unlikely to succeed.

## Discussion and conclusions

In this paper I have shared my reflections on 35 years of experience in the field of seismic hazard assessment, both as a researcher and a practitioner. For some readers, the content may seem to be lacking in technical detail,^7^ but this reflects my view that the biggest challenges we face may not be of a technical nature. For those interested in more details regarding the science, I hope that the long list of references will prove useful.

I am convinced that seismic hazard is inextricably linked to seismic risk, and hazard assessment finds its meaning when applied to the assessment of risk (which in turn finds its meaning when it becomes the starting point for designing measures to reduce the risk posed by earthquakes, be they natural or anthropogenic). I believe that the practice of seismic hazard and seismic risk analyses has advanced enormously, especially with regards to the data sets now available to us and our ability to make measurements that provide excellent constraint on models for future seismicity and the ground shaking that will be generated. In particular I would emphasise the value of characterising the seismogenic potential of geological faults, which is fundamental to characterising earthquake hazard. Insights into the spatial and temporal patterns of observed seismicity have also improved models for future earthquake distributions.

We have also taken great strides forward in terms of characterising and quantifying uncertainties, including both procedural guidance for organising multiple expert assessments and transparent approaches for incorporating the uncertainties into the hazard and risk estimates. These advances, motivated in large part by the nuclear industry, have provided a basis for greater assurance regarding compliance with seismic safety targets since we are increasingly less likely to be surprised by new events. The capture of epistemic uncertainties in seismic hazard analysis is reaching a stage of maturity that may allow us to focus more on reducing the uncertainty intervals rather than ensuring that sufficient uncertainty has been captured. When we re-visit some seismic hazard studies conducted for NPP sites 30 or more years ago, we are often struck by the remarkably optimistic view of the state of knowledge at the time. However, more recent PSHA studies tend to capture epistemic uncertainty as a matter of course and the task before us now is to demonstrate how uncertainty can be reduced through the acquisition of new data and the conduct of new analyses.

In spite of all these advances in seismic hazard analysis, acceptance of the outcome of seismic hazard studies is not automatic, especially when the results obtained contradict preconceptions or exceed prior estimates that have underpinned the design of existing facilities. The challenge posed by increased seismic hazard estimates is clear and the consternation this can lead to is comprehensible, but neither arbitrary modification of the hazard estimates nor defamation of the new studies are legitimate responses. By the same token, diligence and rigour must be applied if new information that could have such an impact is to be presented. In this regard, academic publication is not always helpful, since a paper is more likely to be published and to attract attention if it paints a dramatic picture of high seismogenic potential, which may tempt authors to downplay the uncertainties and highlight the more extreme part of the distribution.

Induced seismicity is not a new phenomenon, but it has attained much greater prominence in recent years due to increases in anthropogenic earthquakes associated with energy technologies in various parts of the world. The seismological community has responded to this situation with great vigour and generated a remarkable body of literature on this topic that has enormously advanced the state of knowledge (although here again, there is a need to avoid sensationalism by exaggerating the impact of small earthquakes or the possibility of large-magnitude induced events). There is now a need for the earthquake engineering community to also deepen its engagement with the challenge of induced seismicity in order to ensure that the resulting seismic risk, as well as the seismic hazard, are properly quantified in a manner consistent with the assessment of seismic risk due to natural earthquakes. All of the advances that have been made in seismic hazard and risk analysis can be brought to bear—with appropriate adaptations—on the challenges posed by induced seismicity. Earthquake engineering is also needed so that the risk mitigation options for managing induced seismicity include structural upgrade and strengthening rather than focusing exclusively on control of the induced seismicity.

To date, there have been some spectacular failures to achieve rational risk management of induced seismicity. These case histories have all resulted in the closure of operations to provide energy, even though in all cases the impact of the induced seismicity was minor, without serious structural damage in any single case—and in one case, with no damage at all. Exaggeration of the impact, generally in form of damage claims that far exceeded the actual damage, is a common feature of all the case histories. Another common feature seems to be the prospect of larger earthquakes occurring if the industrial activity were to continue, even though in some cases these larger events are very unlikely—and in at least one of the cases, probably physically impossible. This highlights that the estimation of the maximum magnitude of earthquake that can be generated by any specific application of an energy technology is an extremely important topic. I would recommend this as a priority area for research, and that the research include the effects of controls such as traffic light protocols to limit the size of the largest induced earthquake. For as long as claims can be made regarding our inability to preclude large-magnitude earthquakes occurring, stakeholders will seek to invoke the precautionary principle as the basis for shutting down the energy-related activities. Every time that the precautionary principle is invoked in response to induced seismicity, we should consider it a failure of seismology and earthquake engineering since it is not a basis for rational risk management.

Objective evaluation of the risk posed by induced earthquakes, and rational decision-making with regards to options for mitigating this risk and balancing it with the benefits of the causative activity, seem to be somewhat elusive goals at the present time. There are many actions that can be taken to improve the prospects of fulfilling these goals, but at the heart of these must be a risk-based approach to the management of induced seismicity, and an informed, independent, and authoritative regulatory body to ensure that risks are mitigated and balanced with benefits.

***Epilogue***: notes to a young engineering seismologist

I feel very privileged to have worked on many very interesting projects and to have collaborated with some remarkable people, both of which have taught me so much. Although my career began in a very different time (before mobile phones, email, and the Internet), some younger readers, setting out on their own career paths, may be interested in how I came to be involved in these wonderfully interesting enterprises. Let me state at the very outset that it was not the result of executing a carefully conceived career plan. Rather I would say that my good fortune was a combination of creating opportunities for serendipity and then fully engaging with the opportunities that consequently opened up for me. To create opportunities, I travelled a great deal (and learning other languages enhanced both the enjoyment and the benefits of these voyages) and I accepted invitations to participate in interesting ventures without giving too much attention to the terms and conditions being offered. And I did participate rather than being a passive observer: Mark Twain is famously quoted as saying “*It is better to keep your mouth closed and let people think you are a fool than to open it and remove all doubt*”, but you will not be noticed if you do not contribute to discussions. The caveat is needing to be ready to acknowledge being wrong, which I have had to do many times, but by engaging in exchanges and occasionally making a useful contribution to the discussion, new invitations and opportunities arose. An outstanding example of this for me was my appointment to the Seismic Advisory Board (SAB) for the Panama Canal Authority during the early phase of the canal expansion programme, one of the most exciting appointments of my career. In the meetings, in which I was active in the discussions, I developed an excellent rapport with SAB member Dr Lloyd Cluff, head of the geosciences department at the Pacific Gas & Electricity company. On the basis of those interactions, Lloyd subsequently appointed me to the SAB for the Diablo Canyon NPP in central California, which was another amazing opportunity to learn from some of the leading figures in the field. And new opportunities subsequently arose from interactions in the Diablo Canyon SAB meetings.

Professor Nick Ambraseys said in the first ever Mallet-Milne lecture that "*There is little room in Engineering Seismology for 'armchair' seismologists and engineers*" (Ambraseys 1988), and I took this admonition to heart, undertaking several field reconnaissance studies of damaging earthquakes in Algeria, Armenia, California, Colombia, Italy, Japan, Peru and Turkey, among others noted below. The first earthquake I visited was the destructive **M** 5.7 San Salvador, El Salvador, earthquake of October 1986. During the visit, made as part of a small EEFIT team (Bommer and Ledbetter 1987), I met Dr Jon Cortina SJ, a Jesuit priest, structural engineer and professor at the Universidad Centroamericana (UCA), with whom I stayed in touch afterwards. In 1993, after completing my PhD, I went to work at the UCA for two years, in what was a fantastically enjoyable and rewarding experience, even if El Salvador would not have automatically been on most people’s recommended list of destinations to advance an academic career. I stayed engaged with my colleagues at the UCA and other institutions in El Salvador after I returned to London to take up a lecturing position at Imperial, securing EU funding for a digital accelerograph network (Bommer et al. 1997) and continuing research on historical earthquakes (Ambraseys et al. 2001). In 1998 I wrote an article for the SECED Newsletter entitled “A 12-year field mission” explaining all the activities and collaborations that arose from the original visit (Bommer 1998). In the end, my involvement with projects in El Salvador lasted much more than a dozen years, but more about that later.

Despite the ever-increasing possibilities to study earthquakes remotely, I still believe that there is enormous value in going to the field: every earthquake is a full-scale laboratory and the connections that are made can have enduring consequences, as was the case with my study of the San Salvador earthquake. Field reconnaissance missions are frequently organised by EEFIT, EERI and GEER following major earthquakes around the world, and there is great value in joining teams led by experienced individuals and participating in the collective reporting and interpretation of field data that follows. However, there are occasions where a more informal approach can also be appropriate. In May 1995 I was in Athens having dinner with our Geotechnical Engineering MSc students on the last evening of a week-long field trip visiting landslides and tunnels under construction, when the news came in of a large earthquake in the north of the country. Very early the next morning, with two Greek MSc students, we rented a car and drove to the affected area, where we spent a week studying the effects of the earthquake (Bommer et al. 1995). Just over a decade later, I recall receiving an automated email from the USGS with notification of a magnitude **M** 7 earthquake in Mozambique and meeting my colleague Dr Clark Fenton in the corridor as we headed to each other’s offices to propose a field reconnaissance. Less than a week later, we were in the field studying the fault rupture (Fig. 8) and just over four months after the earthquake occurred, we published a paper from our findings (Fenton and Bommer 2006). Our adventures in the field and how we found our way to fault rupture—located in a remote region littered with minefields—are recounted in an article in the SECED Newsletter (Bommer and Fenton 2006).

As well as being willing to travel and to engage with opportunities that arise, I would also say that turning down small opportunities that do not appear particularly attractive at face value may sometimes mean losing wonderful opportunities—or rather, the possibility of creating such opportunities. In 2006, I was approached—on the basis of a colleague’s recommendation—by the Council for Geoscience in South Africa to review the chapters related to seismic hazard assessment of the manual being developed for nuclear site characterisation. Although not a particularly exciting engagement, I accepted and produced a lengthy, and rather critical, report summarising my review. Several months later, I was approached for a follow-up review of all the seismic studies that CGS had conducted on behalf of the energy utility Eskom for potential new-build nuclear sites. The work involved reviewing a large number of reports, and once again I wrote a lengthy review, effectively a gap analysis of the studies conducted. Among my recommendations was that the site-specific hazard assessments should be conducted as SSHAC Level 3 studies. This prompted an invitation to visit South Africa for meetings with CGS and Eskom, the outcome of which—to cut a long story short—was the first ever application of the SSHAC Level 3 process outside of North America (Bommer et al. 2015b). As a direct result of that project, I became involved with drafting the updated SSHAC implementation guidelines in NUREG-2117 and NUREG-2213. My contracts for the work on those USNRC documents essentially covered my travel expenses and a fraction of the time spent on the projects, but this was a perfect example of when it makes sense to be involved in an enterprise regardless of the remuneration.

A very interesting part of my work has been related to induced seismicity, and I will finish with the story of how I came to work in this field, which perfectly illustrates the idea of creating opportunities for serendipity and engaging with the opportunities that arise. In January 2001, a major subduction earthquake occurred offshore El Salvador, and 15 years after the 1986 earthquake that first took me that beautiful country, I headed back as part of a field reconnaissance team. During the visit I went to the offices of the geothermal energy company GESAL (now LaGeo) since I knew that they operated strong-motion accelerographs from which I was interested in obtaining copies of the recordings, to supplement those from the network we had installed 5 years earlier in conjunction with the UCA. The secretary of my contact at GESAL told me that he was in a meeting all day and could not be disturbed, but I pleaded with her to let him know that I was visiting from London and that day was my only possibility to come to his office. This worked and I was actually invited to join the meeting he was in, which was with engineers from Shell to discuss a possible enhanced geothermal project using an abandoned well at the Berlín geothermal field in the eastern province of Usulután. One of the main topics of discussion that day was the control of induced seismicity, and I ended up with a contract to work with Shell geophysicist Dr Steve Oates and others on the design of the traffic light scheme that was deployed on the project (Bommer et al. 2006). As recounted in Sect. 12.1 of the paper, this then led to my engagement on the Basel Deep Heat Mining project, and a few years later, following the Huizinge earthquake (Sect. 12.4.2), Dr Oates recommended me to NAM to participate in the development of the hazard and risk model for the Groningen field.

Beyond creating the conditions for opportunities to present themselves and embracing those opportunities when they appear, my only other advice would be to find and harness your own specific strengths and attributes, and then seek out collaborators with complementary skills. Working in great teams has been the greatest source of learning for me as well as a lot of fun. And when teams work really well—the key seems to be having everyone fully engaged and nobody needing to be the smartest person in the room—the outputs can be remarkable. Watching ideas develop as a problem is raised and possible solutions thrown out, challenged, defended, modified, and then elaborated and fine-tuned, is a uniquely satisfying and rewarding experience^8^—and one for me that would qualify as flow (Csikszentmihalyi 1990). I am also utterly convinced that such interactions—particularly when the participants have individually considered the issues and worked on potential solutions beforehand—produce results that far exceed what any individual, however bright, could achieve working alone. Within your collaborations, do not be afraid to contribute to the process—even seemingly ‘dumb’ questions can often nudge the process in very helpful directions. And never compare your abilities and your contributions with those of others—learn all you can from your collaborators but enjoy bringing your own flavours to the kitchen: the dish will be much richer than if everybody brings the same ingredients.

## Data Availability

All figures are other originals created by the author or from acknowledged sources. The Groningen ground-motion records used in Figs. 139 and 141 can be obtained from KNMI (http://rdsa.knmi.nl/dataportal/) or in processed format from the links in the paper by Ntinalexis et al. (2022). The Groningen damage claims data depicted in Fig. 140 is from NAM (Crowley et al. 2019) for the earlier period and from news items accessible at news items at the following link: https://www.schadedoormijnbouw.nl/nieuws?ss_cid=2000000 for later years. Groningen gas production data in Fig. 147 obtained from the NAM web site: https://www.nam.nl/gas-enolie/gaswinning.html#iframe=L2VtYmVkL2NvbXBvbmVudC8_aWQ9Z2Fzd2lubmluZw.

## Footnotes

1 In fairness, at the time of writing, work is underway to introduce a flag in the HiQuake database to indicate the strength of the evidence to support each earthquake being of anthropogenic origin (Professor Gillian Foulger, personal communication, 2022).

2 At the time of writing this paper, preparations have been made to reconvene the Groningen Mmax panel for a revised evaluation in light of new data and analysis, in particular with relation to the possibility of earthquakes that rupture from the gas reservoir downwards into the Carboniferous rock; the new evidence will be presented and discussed at a workshop to take place (Covid-19 permitting) in Amsterdam the week after this Mallet-Milne lecture is presented in London.

3 Who coincidentally was also the chairman of ICHESE (see Sect. 8.2).

4 In Sect. 13.2, I make a case for the value of publication in scientific journals, but I do not believe that this extends to a regulator publishing its own models and theories, including—as this paper does—critiques of the models developed by the licensee. While comments and responses in peer-reviewed journals can be a wonderful forum for scientific exchanges, but it would be a courageous (or foolhardy) operator that would write a comment demonstrating shortcomings in papers published by their regulator.

5 The carbon cost of digital technology has been brought home, in particular, by the enormous quantities of electricity consumed in the mining of crypto-currencies.

6 I recall the late Dr Bryan Skipp, eminent UK earthquake engineer, polymath and founding member of SECED, saying that what distinguishes engineering from science is the question “So what?”.

7 And some readers may feel inclined to agree with the words of Professor Jenny Suckale of Stanford University, who told me that she often reminds her students that the plural of anecdote is not data.

8 A lesson learned from the pandemic is that this only works, in my opinion, with physical meetings—I have not seen this magic recreated on Zoom or Microsoft Teams.
